# Structural Investigation of Aaptourinamine by a Novel Module-Assembly-Based Calculation

**DOI:** 10.3390/md20100649

**Published:** 2022-10-20

**Authors:** Xing Shi, Zhihui Wu, Tianyun Jin, Cili Wang, Pinglin Li

**Affiliations:** 1 Key Laboratory of Marine Drugs, Chinese Ministry of Education, School of Medicine and Pharmacy, Ocean University of China, Qingdao 266003, China; 2 Laboratory of Marine Drugs and Biological Products, Pilot National Laboratory for Marine Science and Technology (Qingdao), Qingdao 266003, China

**Keywords:** module-assembly, aaptourinamine, new scaffold, meta-structures

## Abstract

Natural products have various and complicated structures, which is still a challenge for elucidating these compounds, especially for those lacking two-dimensional nuclear magnetic resonance (2D NMR) correlations mainly caused by high C/H ratios or proton-deficient and multiple heteroatoms through the conventional structural analytical methods. We reported a novel module-assembly calculation method named Dooerafa, which included constructing the meta-structures by a grafting method based on the crucial and the limited 2D NMR correlations, ring-contraction strategy based on mechanic force field and quantum chemical theory, and self-assemble calculation in Python programming for shaping up the structural candidates along with DFT-GIAO calculation. This new method, verified by a known alkaloid spiroreticulatine with the structure determined by X-ray diffraction, was performed for the structural elucidation of aaptourinamine isolated from marine sponge *Aaptos suberitoides*, showing us a brand new scaffold of imidazo [4,5,1-ij]pyrrolo [3,2-f]quinolin-7(8H)-one, which has a biosynthetic relationship with the bioactive and structurally unique aaptamine alkaloid.

## 1. Introduction

Natural products have highly diverse chemical structures and complicated skeletons, posing a challenge to organic chemistry [1,2,3]. Despite the methods that structure analysis continue to promote, including nuclear magnetic resonance (NMR), X-ray crystallography [4], and biosynthesis-assisted structural elucidation [5], it is still challenging work for those complicated structures originating from natural organisms and especially having high C/H ratios or proton-deficient and multiple heteroatoms. Long-range heteronuclear single quantum multiple bond correlation (LR-HSQMBC) NMR technology, a new NMR pulse sequence technique, has illustrated its practical application for elucidating the structures of proton-deficient molecules. However, it has low sensitivity to natural products with small yields, resulting in limited application [6]. There are many unanticipated structural misassignments such as sclerophytin A [7,8], isoschizogamine [9,10], and calyculaglycoside A [11,12] based on existing elucidating structures methods. Therefore, a new method of accurately determining the structure of natural products needs to be developed.

As an important irreplaceable and potential tool, computer-assisted structural elucidation (CASE) has been providing reliable and advanced solutions to elucidate complex and various structures of natural products [13,14,15,16]. CASE programs imitated the way spectrum experts think about structure analysis through NMR data. So, it is also called artificial intelligence programs or expert programs.

At present, an increasing number of CASE programs have been exploited, such as the ACD Laboratories Structure Elucidator, Bruker CMC-se, Mestrelab MNova Structure Elucidator, and Logic for Structure Determination (LSD). Among them, the ACD/structure elucidator, with enormous data sets and strong data compatibility, is the most popular in the natural products field. It enables us to rapidly deduce the correct structure based on fuzzy and incomplete spectrum information in a short time [16], whereas, the probability of being the correct structure is slightly lower than 100%, which requires further confirmation through DFT NMR calculation. Some CASE programs have relative limitations. For example, Bruker CMC-se, with small structure elucidation, can only perform full automated data analysis when the spectral quality is sufficient [17].

With the increasing speed of computers, the Density Functional Theory (DFT) plays an increasingly important role in accurate chemical shift calculations for even complex natural products, which has been proved to be a very useful tool for natural product structure elucidation [18,19]. Moreover, Python, with legibility and extensibility, has become a popular computer programming language the world over, contrasting with other programming languages such as Java and C++, which has been applied to the DP4-AI method for confirming the molecular structure [20].

Hence, a novel module-assembly-based calculation method called Dooerafa (“doors refer to further”) was exploited to elucidate structures. This method combined the fragments of structures obtained by available two-dimensional (2D) NMR into all candidate structures with various skeletal features. Moreover, the programming code that Dooerafa applied to self-assemble was Python language, and it combined the grafting method to make this method more efficient. Through the molecular energy trend chart, the structures were selected, and the correct structure would be obtained via quantum chemical calculation using the DFT-GIAO method, combined with mathematical statistics parameters, which guaranteed the correctness of the structure. Nevertheless, the remaining structures that conform to the spectral information can also be applied to other appropriate research fields. For example, these structures can provide a reference for designing the synthesis routes.

## 2. Results

### 2.1. A Case Study of Spiroreticulatine

To prove the correctness and feasibility of Dooerafa, spiroreticulatine, a previously reported alkaloid that was isolated from the marine sponge *Fascaplysinopsis reticulata* and unambiguously determined by X-ray diffraction [21], was used as a verification module.

**Figure 1:**
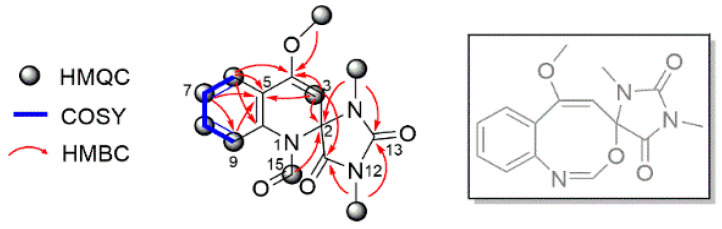
Key 2D NMR correlations of spiroreticulatine and the related possible structure candidate.

**Figure 2:**
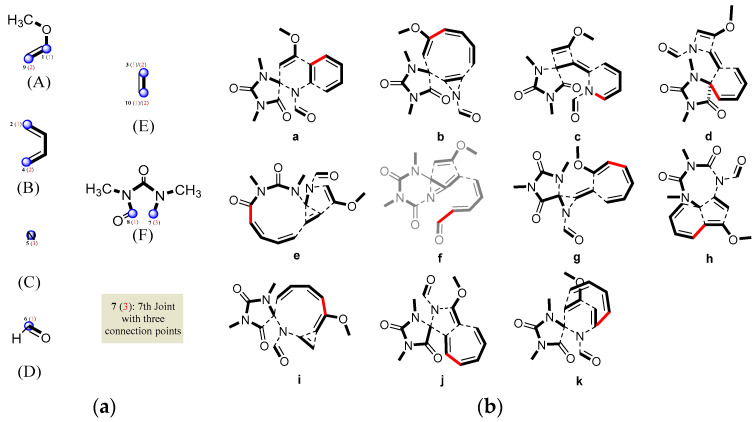
(**a**) The meta-groups of spiroreticulatine A–F. (**b**) The meta-structures of spiroreticulatine a–k (the red bond was grafted on the basis of the meta-groups B, the greys are left out for the unreasonable ^13^C NMR chemical shift of the aldehyde group, and the dashed bond can be freely assembled).

Based on the NMR data of spiroreticulatine, we found that ^2^*J* and ^3^*J* correlations of spiroreticulatine were ambiguous, leading to the structure assignment difficulty and uncertainty. Thus, we can only elucidate the fragments of the structure determined unambiguously by the very certain ^1^H-^1^H COSY correlations and HMBC correlations (Figure 1 and Figure 2a), which caused the structure of spiroreticulatine to not be confirmed based on existing NMR spectrogram information. That is, there was no unique structure having a good relationship with 2D NMR experiments (Figure 1). Furthermore, although the problem of structure conformation can be easily distinguished by DFT-GIAO calculations of ^13^C NMR data, for now, the real problem could be which kind and how many of the structural candidates the compound implied. So, the Dooerafa for spiroreticulatine was performed.

In that case, there were six meta-groups A–F which were the fragments of the structure for spiroreticulatine established by available NMR data (Figure 2a), the remaining 16 connection points joining the meta-groups into a whole structure based on the bond number of atoms (consistent with Supporting Information Section S2). To obtain all possible structures based on the connection points above-mentioned, an original program code in Python was written, which took approximately two weeks to complete the structure assembling calculations, indicating this work was time-consuming.

For efficiency, a novel grafting method extending the remaining meta-groups on the basis of one meta-group within the allowable range of statistics and existing NMR data was studied. This method was applied to construct 11 meta-structures a–k (all possible assembly structures through meta-groups A, C–F segments extending based on B, Figure 2b) which were lowered down to 14 connection points and used for the assembling calculation, taking less than 16 h. Then, 854 group data via Python programming code were obtained and converted into chemical structures.

**Figure 3:**
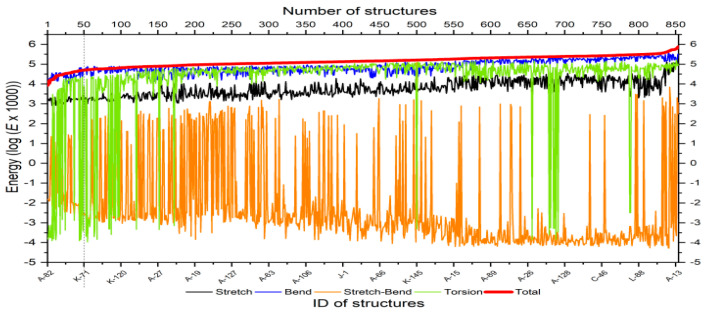
The energy trend of the structures for spiroreticulatine (the values were converted with log_10_(*E* × 1000)).

To narrow down the range of the structure for spiroreticulatine, five types of energies related to the stability of the structures including stretch, bend, stretch-bend, torsion, and total energy were calculated automatically by Python in Chem 3D software using an MM2 molecular mechanic force field [22,23], because the natural products with more stable chemical features tend to be real, especially considering chemically appropriate structures. Then, an inflection point in the most important trend of the total energy trend for spiroreticulatine was found (Figure 3), and there were 50 structures with lower energy before the inflection point, indicating that these structures are more reasonable and stable. On the basis of the tendency toward stability and rationality, these structures before the inflection point were selected to calculate for ^1^H and ^13^C-NMR using the DFT-GIAO method to further confirm the structure of spiroreticulatine.

Furthermore, a related analysis of the fitting between experimental and calculated data using the coefficient of determination (R^2^), as well as the mean absolute error (MAE) and truncated absolute difference (TAD), gave us the correct structure of spiroreticulatine, which was the fifth one in all the 854 structure candidates. Certainly, the selected structure was consistent with the structure determined by X-ray diffraction. Thus, the verification of spiroreticulatine indicated that Dooerafa was suitable for determining the structure of natural products.

Then, the Dooerafa was applied to confirm the structure of an unknown alkaloid called aaptourinamine, which lacked 2D NMR correlations as well. First, the structure of aaptourinamine was preliminarily analyzed by NMR data.

### 2.2. Structural Investigation of Aaptourinamine

Aaptourinamine was isolated from the marine sponge *Aaptos suberitoides*. It had a molecular structure of C_12_H_7_ON_3_ as deduced from HRESIMS data *m*/*z* 210.0661 [M+H]+ (calcd for C_12_H_8_ON_3_, 210.0662) and 232.0481 [M+Na]+ (calcd for C_12_H_7_ON_3_Na, 232.0481) supported by the ^1^H and ^13^C NMR spectral data (see Supporting Information Section S6). It was a potentially new compound with good purity and distinct ^1^H and ^13^C NMR experimental data having no match with the ^13^C NMR database in MICRONMR [24]. However, it had high C/H ratios and a lack of efficient HMBC cross-peaks to be unambiguously determined; neither was it possible for efficient ^15^N NMR due to the insufficient amount of sample and low sensitivity, though the ^15^N NMR has become a great potential tool for elucidating structures [25].

**Figure 4:**
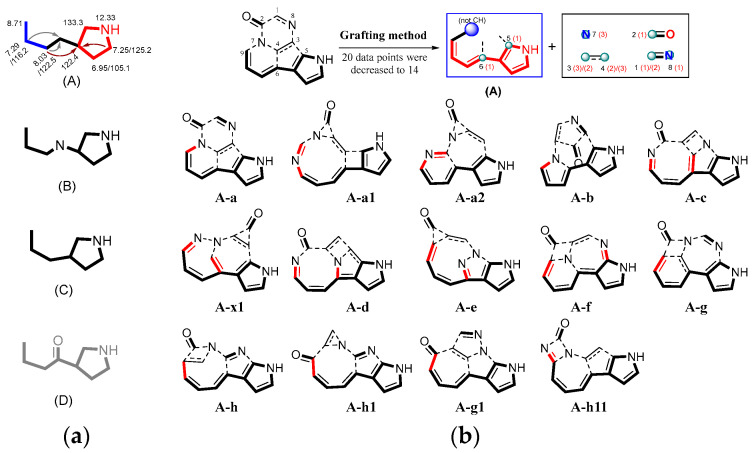
(**a**) The core structure candidates according to the key HMBC, ^1^H-^1^H COSY, and HMQC experimental correlations (the expected correlations in grey were not reasonable). (**b**) Meta-groups and meta-structures based on candidate A (the red bond was grafted on the basis of core structure candidate A, and the dashed bond can be freely assembled).

With the aid of HMQC and ^1^H-^1^H COSY experiments, aaptourinamine displayed one NH proton at *δ*_H_12.31 (1H, s) and six olefinic protons at *δ*_H_8.71 (2H, one doublet *J* = 6.85 Hz and one overlapped singlet), 8.03 (1H, d, *J* = 6.85 Hz), 7.29 (1H, t, *J* = 6.82 Hz), 7.25 (1H, t, *J* = 2.57 Hz), 6.95 (1H, dd, *J* = 2.35, 2.05 Hz) in ^1^H NMR. The profile of an aromatic compound was shown in ^13^C NMR (DEPT), displaying eleven olefinic carbons (five olefinic CH) with chemical shifts around *δ*_C_105–134, as well as one carbonyl group at *δ*_C_168.8. The ^1^H-^1^H COSY and HMBC correlations indicated a dihydropyrrole moiety in which the crucial cross-peaks of *δ*_H_7.25/*δ*_C_122.4 and *δ*_H_8.03/*δ*_C_122.4 were distinguished from a cross-peak group of all the olefinic protons with the adjacent *δ*_C_122.4/122.5. Besides, there must be an imine group, except for five pairs of double bonds and the carbonyl group according to HRESIMS data. Thus, four core structures were deduced from the experimental data of aaptourinamine (Figure 4a). However, the core structure D was not reasonable because of the high deshielded chemical shift of the carbonyl group at more than *δ*_C_180.0, much bigger than the practical *δ*_C_168.8 [26,27], leaving three potential core structure candidates A–C.

**Figure 5:**
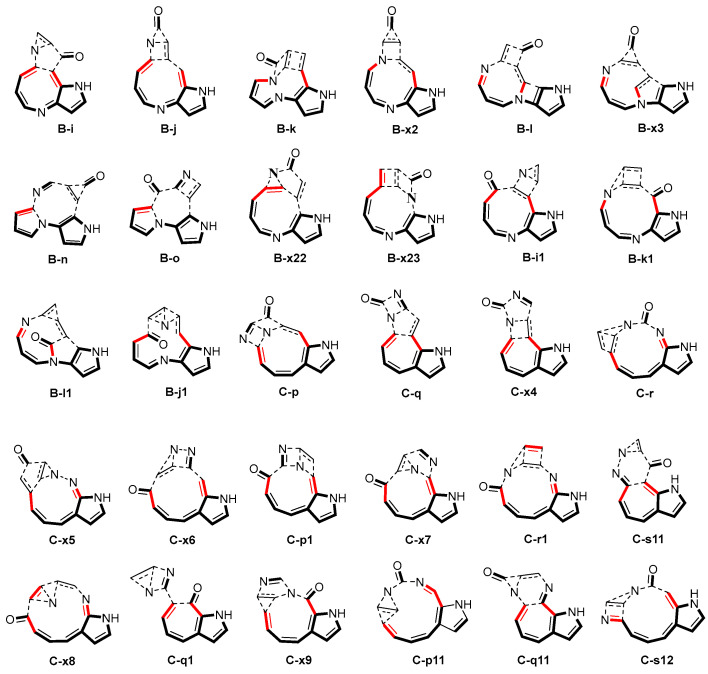
Meta-structures based on candidates B and C (the red bond was grafted on the basis of core structure candidates, and the dashed bond can be freely assembled).

To save calculation time, the grafting method based on the core structure candidates which were the structural fragments conforming to the NMR experiments data was applied to obtain the meta-structures with 14 connection points (Supporting Information Section S2) as in the case of candidate A (Figure 4a). Thus, there were 44 meta-structures, as shown in Figure 4 and Figure 5, which was constructed based on an imine group and a carbonyl group being certain and connected with the core structures A–C or outside the core structures to make certain there were less than 14 connection points in total for each meta-structure.

**Figure 6:**
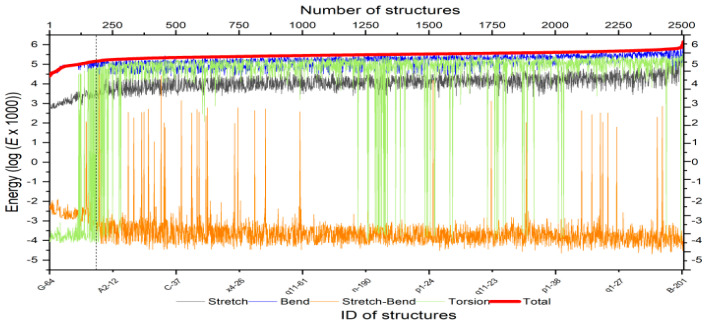
The energy trend of the structures related to aaptourinamine (the values were converted with log_10_(*E* × 1000)).

Subsequently, 2507 structures were drawn manually by the group data obtained through the program code in Python. These structures showed a large diversity with various kinds of assembling. Then, the energies related to molecular stability were calculated by Python controlling Microsoft Excel and Chem 3D. The molecular energy in the mechanic force field displayed an interesting trend for structural change. In the energy trend of the structures related to aaptourinamine, there was an inflection point at which the structures tended to be unstable with a sudden change especially in the torsion, stretch-bend, and total energies (Figure 6, see Supplementary Data S1), which indicated the structures still existed as a chemically unreasonable phenomenon in the case that the main structural moieties of meta-groups have been confirmed based on the crucial experimental NMR data. Meanwhile, the structures before the inflection point were chemically reasonable and stable while structurally variable after that inflection point. So, there must be a sensible reason to shape up the complex and chemically unfair structures existing in nature [28].

**Scheme 1:**
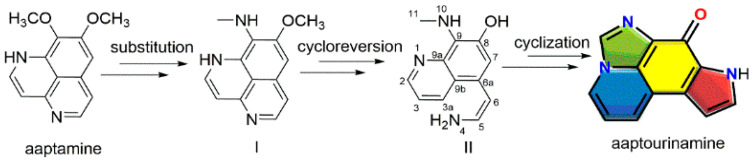
The predicted biosynthetic pathway of aaptourinamine.

Then, 150 structures before the inflection point showing lower energy were calculated for ^1^H and ^13^C NMR using the DFT-GIAO method (Figure 6; Supporting Information Section S4) and analyzed the linear regression parameters which were used to reflect the fitting between experimental and calculated data. Finally, the correct structure of aaptourinamine (A-205, the 9th one in the 2507 candidate structures; Supporting Information, Section S5) with the best match of calculated NMR data was shown as imidazo [4,5,1-ij]pyrrolo [3,2-f]quinolin-7(8H)-one, which was structurally related to aaptamine alkaloid, as predicted in the biosynthesis pathway in Scheme 1.

Therefore, aaptourinamine with a new scaffold of the aaptamine family which had a different type featured the formation of a pyrrolo [3,2-f]quinolone core was first found in nature, indicating an intriguing profile of biosynthesis and bioactivity. The chemical shift of the carbonyl carbon C-8 at *δ*_C_168.8 (Table S2, Supporting Information) in aaptourinamine was greatly upshielded compared to the normal ketone group likely due to the large and favorable conjugated system, as in the case of exiguamine A [27]. Incidentally, there are 18 π-electrons over 16 centers for the four-ring systems of aaptourinamine, which conformed to the 4n + 2 rule by Hückel, causing the structure to possess aromaticity, and the nitrogen atoms play an important role in the unique conjugated aromatic system, which makes for a more novel scaffold [29,30].

## 3. Discussion

To summarize, Dooerafa validated by spiroreticulatine with structure immobilized by X-ray diffraction is successfully used to elucidate the structure of aaptourinamine with an unknown skeleton, providing the credible and accurate result. In this method, first, meta-groups are very important, which were assigned from the believable NMR and HRESIMS experimental data. Then, the grafting method is practical and efficient to obtain the meta-structures to save calculation time. Subsequently, the self-created program code in Python, which shows all group data converting to the structure candidates, is the first to report for determining the structure. Running the code takes less than 16 h, indicating the efficiency of Dooerafa.

On the other hand, the DFT-GIAO calculation of NMR data has been proved to be a reliable method to predict the right structure. ^13^C NMR data, which is almost unchanging in a different solvent in the practical experiments, is especially suitable for the DFT calculation [31]. Nevertheless, ^1^H NMR data is impressible to the solvent and is not a good choice to determine structure as in the cases of spiroreticulatine and aaptourinamine in the present study. Thus, the logical use of a mechanic force field and quantum chemical theory of the ring-contraction strategy is the key point for the study.

**Figure 7:**
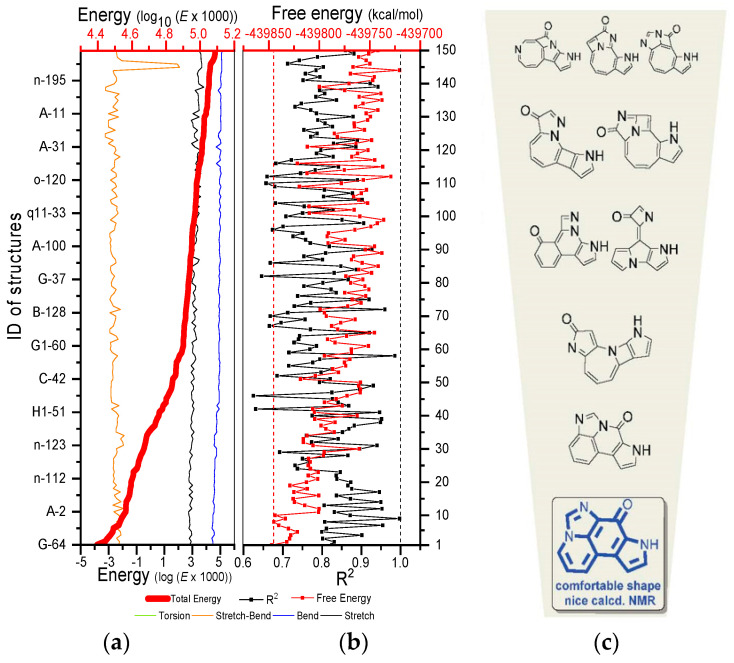
(**a**) The molecule energy trend of the 150 structures before the inflection point related to aaptourinamine in MM2 molecular mechanic force field (the values were converted with log_10_(*E*×1000)). (**b**) The molecule energy trend of the 150 structures in DFT calculated Gibbs free energy, and the related coefficient of determination (R^2^) comparing the experimental and calculated ^13^C NMR data. (**c**) ‘ring-contraction strategy’ calculation for the correct aaptourinamine.

Hence, the molecular energies trending in the mechanic force field showing clear inflection points are suitable to select stable structures with great efficiency (Figure 7), in which the correct structure of aaptourinamine occupied a ninth place in the top 150 structures. The molecular energy with DFT calculation generally displayed a similar trend but showed a closer relationship with the correct structure. Finally, the right skeleton of the natural product was determined according to the NMR calculation showing the best coefficient of determination (R^2^). Thus, the structures which were ticked from the numerous candidates with the lower energy before the inflection point in the energy trend and showed the best match with the experimental data must be the right and unique naturally occurring structure.

## 4. Materials and Methods

### 4.1. General Experimental Procedures

NMR spectra were measured on a Bruker AVANCE NEO 400 (^1^H, 400 MHz; ^13^C, 101 MHz; Bruker, Beijing, China), an Aglient DD2-500 (^1^H, 500 MHz; ^13^C, 126 MHz; Agilent, Beijing, China), or a JEOL JNM-ECP 600 (^1^H, 600 MHz; ^13^C, 151 MHz; JEOL, Beijing, China) spectrometer. The 2.48 and 40.1 ppm resonances of DMSO were used as internal references for ^1^H and ^13^C NMR spectra, respectively. HRESIMS spectra were measured on Micromass Q-Tof Ultima GLOBAL GAA076LC mass spectrometers (Autospec-Ultima-TOF, Waters, Shanghai, China). Semi-preparative HPLC was performed using a Waters 1525 pump (Waters, Singapore) equipped with a 2998 photodiode array detector and a YMC C18 column (YMC, 10 × 250 mm, 5 μmol/L). Preparative HPLC was performed using a Shimadzu LC-20AR (Shimadzu, Shanghai, China) pump equipped with an LC-20A array detector and a SilGreen C18 column (SilGreen, 20 × 250 mm, 5 μm). A Sephadex LH-20 (Amersham Pharmacia Biotech, Buckinghamshire, UK) was used for column chromatography. Silica gel (200–300 mesh, 300–400 mesh and silica gel H; Qingdao Marine Chemical Factory, Qingdao, China) were used for column chromatography, and precoated Silica gel plates (GF254, Qingdao Marine Chemical Factory, Qingdao, China) were used for TLC and spots visualized by heating SiO_2_ plates sprayed with 10% H_2_SO_4_ in EtOH.

### 4.2. Animal Material

The marine sponge *Aaptos suberitoides* was collected from the Yongle Islands of Xisha Islands of the South China Sea in May 2012 and was identified by Leen van Ofwegen, National Museum of Natural History, Netherlands. The voucher specimen (No. XS-2012-30), which was frozen at −20 °C, was deposited at the School of Medicine and Pharmacy, Ocean University of China, P. R. China.

### 4.3. Extraction and Isolation

The frozen sponge *Aaptos suberitoides* (5.4 kg, wet weight) was shredded and extracted with MeOH (five times each time for 3 days). Then, the combined solution was concentrated under a vacuum and desalinated to yield 325.0 g of residue. The residue was subjected to vacuum liquid chromatography (VLC) on a silica gel column by gradient mixtures of petroleum ether/acetone (from 50/1 to 0/1, *v*/*v*) and CH_2_Cl_2_/MeOH (from 10/1 to 0/1, *v*/*v*) to yield nine major fractions (Fr.1−9). Fr.6 (12 g) showed cytotoxic activity against A549 and H1299 tumor cell lines with an inhibition ratio of more than 92% at the concentration of 50 μg/mL. This fraction was divided into eight subfractions (Fr.6-1−6-8) by silica gel CC eluting with petroleum CH_2_Cl_2_/MeOH (50/1−0:1, *v/v*). Fr.6-5 (0.4 g) was separated by Sephadex LH-20 (MeOH) to give six fractions (Fr.6-5-1−6-5-6). Fr.6-5-5 was purified by semipreparative HPLC (YMC C8, 10 × 250 mm, 5 μm; MeOH/H_2_O 40:60, *v/v*; 1.5 mL/min; detection UV: 230 nm) to produce compound aaptourinamine (1.5 mg).

Aaptourinamine: yellow oil; ^1^H NMR and ^13^C NMR data, Table S2 (see Supporting Information); HRESIMS *m*/*z* 210.0661 [M+H]+ (calcd for C_12_H_8_ON_3_, 210.0662) and 232.0481 [M+Na]+ (calcd for C_12_H_7_ON_3_Na, 232.0481).

### 4.4. Procedure for the Dooerafa

In Dooerafa, the meta-groups were deduced on the basis of experimental data, and the meta-structures were assembled via the grafting method to make the connection points less than 14 for the purpose of saving time. Then, a self-assembly procedure written by Python was expected to assemble the meta-groups based on meta-structures to obtain all the structure candidates which were converted from the calculated group data and were drawn manually in Chemoffice 14. InChI, the key of each structure was obtained in Excel embedded with Chemoffice 14. The duplicates were removed according to the InChI code. The molecular energy calculation in a mechanic force field was performed by Python controlling Chem 3D and Microsoft Excel software. A conformational search was performed using the Maestro 11.9 of the Schrödinger software package [32]. The DFT-GIAO calculation of ^1^H and ^13^C NMR data was performed on Gaussian 16 at PCM/mPW1PW91/6-31+G** level. Dooerafa calculations were performed in the Supercomputing Center of the Pilot National Laboratory for Marine Science and Technology (Qingdao) with a standard Linux x64 operating system, while other operations were in a standard notebook running Windows 10 Pro x64.

### 4.5. Quantum Chemical Calculations

The quantum chemical calculations were carried out by Gaussian 16 software using DFT [33]. The input geometries were built using the Chemdraw Pro 14.1 software with an MM2 force field. The lowest energy conformers within 10 kcal/mol were subjected to further DFT calculations at the B3LYP-D3(BJ)/6-31g(d) level in the gas phase, and all minima displayed no imaginary frequencies via vibrational frequency analysis at the same level. Thermal corrections to Gibbs energies were obtained from frequency calculations at 298 K. The population of each conformer and Gibbs energies were calculated by Boltzmann distribution based on Gibbs free energy with the Shermo [34]. Then, conformers with distributions higher than 1% were chosen for the GIAO calculations of NMR shielding constants with spin-spin interactions accomplished using the DFT method at the PCM/mPW1PW91/6-31+G**//B3LYP/6-31G* level in DMSO, and using the PCM model as the optimal method for error analysis. The calculated shielding constants of these conformers were averaged according to the Boltzmann distribution theory and their relative Gibbs free energy. The shielding constants (including C and H) obtained were directly statistically analyzed with experimental chemical shifts.

## 5. Conclusions

In conclusion, Dooerafa which is a novel module-assembly calculation employed by the program package Python and DFT-Gaussian has been developed and gives us a new opportunity to analyze the structure with absent 2D NMR correlations. Besides, marine invertebrates are the main resource of natural molecules with novel structures. Aaptourinamine, elucidated first by Dooerafa and revalidated by a biosynthetic pathway, is shown as a new scaffold of the aaptamine family having a totally different transformation type featured by the formation of a pyrrolo [3,2-f]quinolone core, indicating an intriguing profile of the biosynthesis and bioactivity. Thus, this exploited method provides a novel insight into elucidating the structures of natural products.

## Data Availability

The calculation details, related structure, and NMR spectra are located in Supporting Information. The calculation results for energies related to the stability of the structures can be found in Supplementary Data S1.

## Supplementary Materials

The following supporting information can be downloaded at: https://www.mdpi.com/article/10.3390/md20100649/s1. Figure S1: Core structures A–D for aaptourinamine; Figure S2: The counting principle of the connection site; Figures S3–S5: The meta-structures for the core structure A–C; Figures S6–S12: Spectra for aaptourinamine; Table S1: The Computational details of the 150 structures [35]; Table S2: 1D and key 2D NMR data of aaptourinamine; Supplementary Data S1: Five kinds of energies calculated for aaptourinamine.
