# Supplementary material for: Structural Investigation of Aaptourinamine by a Novel Module-Assembly-Based Calculation
Source: Mar Drugs. 2022 Oct 20;20(10):649. doi: 10.3390/md20100649 (PMC9604825; doi:10.3390/md20100649)
Supplement: Supplementary file 1 [file marinedrugs-20-00649-s001.zip › Supplementary Data 1.pdf]

Five kinds of energies calculated for aaptourinamine

		Stretch	Bend	Stretch-Bend	Torsion	Total Energy	
G-64	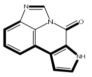 $C_{12}H_7N_3O$	0.6193	29.4203	-0.188	-10.49	24.8857	InChI=1S/C12H7N3O/c16-12-10-7(4-5-13-10)8-2-1-3-9-11(8)15(12)6-14-9/h1-6,13H
G-61	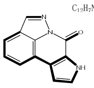 $C_{12}H_7N_3O$	0.8158	29.443	-0.1702	-11.61	27.6437	InChI=1S/C12H7N3O/c16-12-10-8(4-5-13-10)9-3-1-2-7-6-14-15(12)11(7)9/h1-6,13H
H-41	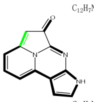 $C_{12}H_7N_3O$	0.5838	24.2838	-0.2751	-6.96	28.6774	InChI=1S/C12H7N3O/c16-10-6-7-2-1-3-9-8-4-5-13-11(8)14-12(10)15(7)9/h1-6,13H
G-77	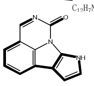 $C_{12}H_7N_3O$	0.8842	36.8875	-0.1325	-11.61	29.8313	InChI=1S/C12H7N3O/c16-12-14-6-7-2-1-3-8-9-4-5-13-11(9)15(12)10(7)8/h1-6,13H
G-72	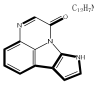 $C_{12}H_7N_3O$	0.8379	35.9095	-0.1491	-10.49	30.7624	InChI=1S/C12H7N3O/c16-10-6-14-9-3-1-2-7-8-4-5-13-12(8)15(10)11(7)9/h1-6,13H
F-36	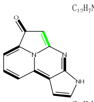 $C_{12}H_7N_3O$	0.5524	24.9718	-0.2552	-3.18	30.9923	InChI=1S/C12H7N3O/c16-10-6-11-14-12-7(4-5-13-12)8-2-1-3-9(10)15(8)11/h1-6H,(H,13,14)
H-89	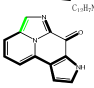 $C_{12}H_7N_3O$	0.6614	27.2712	-0.2188	-7.96	33.3615	InChI=1S/C12H7N3O/c16-11-10-8(4-5-13-10)9-3-1-2-7-6-14-12(11)15(7)9/h1-6,13H
H11-99	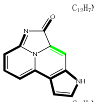 $C_{12}H_7N_3O$	0.6236	28.8637	-0.2704	-7.1	33.9255	InChI=1S/C12H7N3O/c16-12-10-6-8-7(4-5-13-8)9-2-1-3-11(14-12)15(9)10/h1-6,13H
A-205	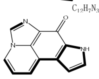 $C_{12}H_7N_3O$	0.6377	28.5779	-0.1138	-7.7000	34.6231	InChI=1S/C12H7N3O/c16-12-9-7(3-4-13-9)8-2-1-5-15-6-14-10(12)11(8)15/h1-6,13H
H11-144	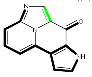 $C_{12}H_7N_3O$	0.552	28.2809	-0.1965	-4.18	35.5961	InChI=1S/C12H7N3O/c16-12-9-6-14-10-3-1-2-8(15(9)10)7-4-5-13-11(7)12/h1-6,13H
A-2	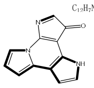 $C_{12}H_7N_3O$	0.5225	35.5435	-0.4186	-5.11	36.5923	InChI=1S/C12H7N3O/c16-9-6-14-12-10(9)11-7(3-4-13-11)8-2-1-5-15(8)12/h1-6,13H
G-13	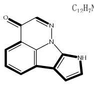 $C_{12}H_7N_3O$	0.8976	35.7206	-0.0883	-8.83	36.7221	InChI=1S/C12H7N3O/c16-10-6-14-15-11-7(2-1-3-9(10)11)8-4-5-13-12(8)15/h1-6,13H
B-15	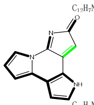 $C_{12}H_7N_3O$	0.6383	37.0653	-0.4954	-6.8300	36.7441	InChI=1S/C12H7N3O/c16-10-6-8-11-7(3-4-13-11)9-2-1-5-15(9)12(8)14-10/h1-6,13H
A-43	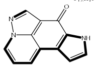 $C_{12}H_7N_3O$	0.7851	29.5535	-0.1306	-5.04	36.8764	InChI=1S/C12H7N3O/c16-12-9-6-14-15-5-1-2-8(11(9)15)7-3-4-13-10(7)12/h1-6,13H
H-58	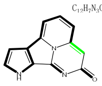 $C_{12}H_7N_3O$	1.0299	33.8094	-0.2722	-8.89	37.8158	InChI=1S/C12H7N3O/c16-10-6-7-2-1-3-9-8-4-5-13-11(8)12(14-10)15(7)9/h1-6,13H
A-7	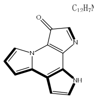 $C_{12}H_7N_3O$	0.569	35.9588	-0.3973	-6.77	38.4748	InChI=1S/C12H7N3O/c16-9-6-14-11-10-7(3-4-13-10)8-2-1-5-15(8)12(9)11/h1-6,13H
G-32	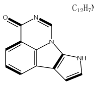 $C_{12}H_7N_3O$	1.0393	37.8963	-0.1479	-8.83	38.7282	InChI=1S/C12H7N3O/c16-12-9-3-1-2-7-8-4-5-13-11(8)15(6-14-12)10(7)9/h1-6,13H
o-249	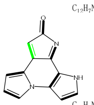 $C_{12}H_7N_3O$	0.5836	37.7969	-0.4667	-6.83	38.925	InChI=1S/C12H7N3O/c16-10-6-7-8-2-1-5-15(8)9-3-4-13-12(9)11(7)14-10/h1-6,13H

H11-107	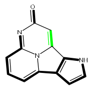 $C_{12}H_7N_3O$	1.0345	35.2572	-0.2758	-7.83	39.4141	InChI=1S/C12H7N3O/c16-11-6-9-12-7(4-5-13-12)8-2-1-3-10(14-11)15(8)9/h1-6,13H
B-36	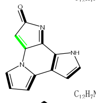 $C_{12}H_7N_3O$	0.5700	37.8115	-0.4635	-6.8300	39.4375	InChI=1S/C12H7N3O/c16-10-6-9-12(14-10)11-7(3-4-13-11)8-2-1-5-15(8)9/h1-6,13H
n-112	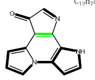 $C_{12}H_7N_3O$	0.5505	36.5458	-0.3962	-4.18	40.0021	InChI=1S/C12H7N3O/c16-9-6-14-12-10(9)7-2-1-5-15(7)8-3-4-13-11(8)12/h1-6,13H
o-246	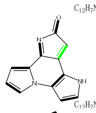 $C_{12}H_7N_3O$	0.6213	37.8819	-0.4702	-4.9	40.4893	InChI=1S/C12H7N3O/c16-10-6-7-11-9(3-4-13-11)15-5-1-2-8(15)12(7)14-10/h1-6,13H
n-116	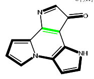 $C_{12}H_7N_3O$	0.5705	36.6427	-0.3974	-3.18	40.6556	InChI=1S/C12H7N3O/c16-9-6-14-12-7-2-1-5-15(7)8-3-4-13-11(8)10(9)12/h1-6,13H
A-77	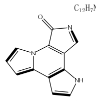 $C_{12}H_7N_3O$	0.7588	39.8004	-0.4695	-6.83	42.5764	InChI=1S/C12H7N3O/c16-12-11-8(6-14-12)10-7(3-4-13-10)9-2-1-5-15(9)11/h1-6,13H
n-188	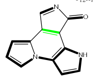 $C_{12}H_7N_3O$	0.6841	40.6443	-0.4488	-5.17	43.2659	InChI=1S/C12H7N3O/c16-12-10-7(6-14-12)8-2-1-5-15(8)9-3-4-13-11(9)10/h1-6,13H
A-79	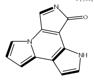 $C_{12}H_7N_3O$	0.6046	40.439	-0.4409	-5.17	43.4986	InChI=1S/C12H7N3O/c16-12-10-9(6-14-12)15-5-1-2-8(15)7-3-4-13-11(7)10/h1-6,13H
n-187	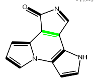 $C_{12}H_7N_3O$	0.711	40.6044	-0.455	-4.24	44.1716	InChI=1S/C12H7N3O/c16-12-10-7(6-14-12)11-9(3-4-13-11)15-5-1-2-8(10)15/h1-6,13H
G1-79	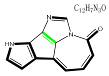 $C_{12}H_7N_3O$	0.981	54.2429	-0.311	-10.49	44.8958	InChI=1S/C12H7N3O/c16-9-3-1-2-8-7-4-5-13-10(7)11-12(8)15(9)6-14-11/h1-6,13H
C-43	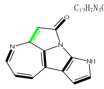 $C_{12}H_7N_3O$	0.6313	54.2300	-0.2471	-5.7000	45.9080	InChI=1S/C12H7N3O/c16-10-6-9-11-7(2-1-4-13-9)8-3-5-14-12(8)15(10)11/h1-6,14H
C-39	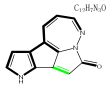 $C_{12}H_7N_3O$	0.8105	54.9447	-0.2788	-10.5500	46.8038	InChI=1S/C12H7N3O/c16-10-6-9-11-7(3-5-13-11)8-2-1-4-14-15(10)12(8)9/h1-6,13H
n-123	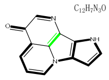 $C_{12}H_7N_3O$	0.8613	35.2223	-0.0886	-2.12	47.2803	InChI=1S/C12H7N3O/c16-9-6-14-11-10-8(3-4-13-10)15-5-1-2-7(9)12(11)15/h1-6,13H
n-198	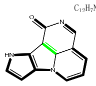 $C_{12}H_7N_3O$	0.917	38.6938	-0.099	-5.17	47.865	InChI=1S/C12H7N3O/c16-12-9-10-8(3-4-13-10)15-5-1-2-7(6-14-12)11(9)15/h1-6,13H
n-210	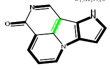 $C_{12}H_7N_3O$	1.0813	37.6015	-0.1408	-4.11	48.1425	InChI=1S/C12H7N3O/c16-12-7-2-1-5-15-9-3-4-13-10(9)8(6-14-12)11(7)15/h1-6,13H
n-113	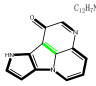 $C_{12}H_7N_3O$	0.8463	37.3669	-0.0777	-2.12	48.6871	InChI=1S/C12H7N3O/c16-9-6-14-7-2-1-5-15-8-3-4-13-11(8)10(9)12(7)15/h1-6,13H
G1-55	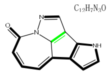 $C_{12}H_7N_3O$	1.3235	55.7794	-0.287	-10.55	49.9772	InChI=1S/C12H7N3O/c16-10-3-1-2-8-7-4-5-13-11(7)9-6-14-15(10)12(8)9/h1-6,13H
A2-61	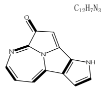 $C_{12}H_7N_3O$	0.8459	51.0599	-0.301	-5.9	51.7907	InChI=1S/C12H7N3O/c16-10-6-9-11-7(3-5-13-11)8-2-1-4-14-12(10)15(8)9/h1-6,13H
D-16	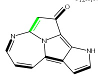 $C_{12}H_7N_3O$	0.7350	52.8473	-0.2477	-3.1800	54.2489	InChI=1S/C12H7N3O/c16-9-6-10-13-4-1-2-8-7-3-5-14-11(7)12(9)15(8)10/h1-6,14H

G1-38	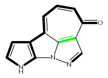 $C_{12}H_7N_3O$	0.9427	56.1479	−0.2363	−5.04	54.8006	InChI=1S/C12H7N3O/c16-10-3-1-2-7-8-4-5-13-12(8)15-11(7)9(10)6-14-15/h1-6,13H
H1-75	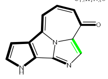 $C_{12}H_7N_3O$	0.7912	54.3054	−0.2516	−4.18	55.8508	InChI=1S/C12H7N3O/c16-10-3-1-2-8-7-4-5-13-11(7)12-14-6-9(10)15(8)12/h1-6,13H
I-65	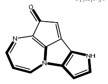 $C_{12}H_7N_3O$	0.9012	52.3096	−0.1927	−3.91	57.1763	InChI=1S/C12H7N3O/c16-9-6-7-10-8(2-4-14-10)15-5-1-3-13-11(9)12(7)15/h1-6,14H
H1-51	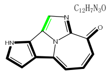 $C_{12}H_7N_3O$	1.0726	56.0431	−0.2987	−6.9	57.5894	InChI=1S/C12H7N3O/c16-10-3-1-2-8-7-4-5-13-11(7)9-6-14-12(10)15(8)9/h1-6,13H
H-148	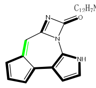 $C_{12}H_7N_3O$	1.2972	83.1531	−0.6288	−13.54	60.1273	InChI=1S/C12H7N3O/c16-12-14-10-6-7-2-1-3-8(7)9-4-5-13-11(9)15(10)12/h1-6,13H
I-66	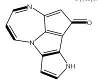	0.9725	55.0755	−0.1842	−1.19	60.6569	InChI=1S/C12H7N3O/c16-9-6-7-12-10(9)11-8(2-4-14-11)15(12)5-1-3-13-7/h1-6,14H
H-149	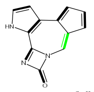 $C_{12}H_7N_3O$	1.2305	83.0582	−0.6034	−13.54	63.052	InChI=1S/C12H7N3O/c16-12-14-11-10-9(4-5-13-10)8-3-1-2-7(8)6-15(11)12/h1-6,13H
H11-68	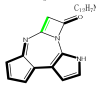 $C_{12}H_7N_3O$	0.8853	81.6821	−0.661	−8.56	63.1592	InChI=1S/C12H7N3O/c16-11-6-10-14-9-3-1-2-7(9)8-4-5-13-12(8)15(10)11/h1-6,13H
F-44	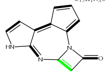 $C_{12}H_7N_3O$	0.8885	81.6812	−0.6786	−8.56	64.7771	InChI=1S/C12H7N3O/c16-11-6-10-14-12-8(4-5-13-12)7-2-1-3-9(7)15(10)11/h1-6H,(H,13,14)
F-39	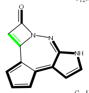 $C_{12}H_7N_3O$	0.854	82.0203	−0.6834	−12.54	66.0692	InChI=1S/C12H7N3O/c16-11-6-10-8-3-1-2-7(8)9-4-5-13-12(9)14-15(10)11/h1-6H,(H,13,14)
G1-108	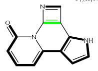 $C_{12}H_7N_3O$	1.2199	75.9162	−0.6734	−7.83	67.6958	InChI=1S/C12H7N3O/c16-10-3-1-2-9-7-4-5-13-11(7)8-6-14-12(8)15(9)10/h1-6,13H
G1-80	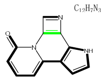 $C_{12}H_7N_3O$	1.1488	75.9844	−0.6595	−6.77	68.3645	InChI=1S/C12H7N3O/c16-10-3-1-2-8-7-4-5-13-11(7)12-9(6-14-12)15(8)10/h1-6,13H
H11-60	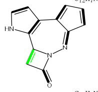 $C_{12}H_7N_3O$	0.787	82.1482	−0.6714	−10.55	68.7169	InChI=1S/C12H7N3O/c16-11-6-10-12-8(4-5-13-12)7-2-1-3-9(7)14-15(10)11/h1-6,13H
C-42	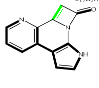 $C_{12}H_7N_3O$	1.3450	78.3761	−0.4861	−5.7600	69.0697	InChI=1S/C12H7N3O/c16-10-6-9-11-7(2-1-4-13-11)8-3-5-14-12(8)15(9)10/h1-6,14H
C-44	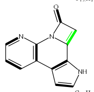 $C_{12}H_7N_3O$	1.1679	78.3801	−0.4984	−6.7600	71.5441	InChI=1S/C12H7N3O/c16-10-6-9-11-7(3-5-13-11)8-2-1-4-14-12(8)15(9)10/h1-6,13H
H-52	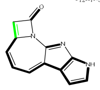	0.8016	87.3188	−0.5391	−7.82	72.1239	InChI=1S/C12H7N3O/c16-10-6-7-2-1-3-9-8-4-5-13-11(8)14-12(9)15(7)10/h1-6,13H
B-138	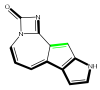 $C_{12}H_7N_3O$	1.2784	92.3785	−0.4982	−12.5400	72.1450	InChI=1S/C12H7N3O/c16-12-14-11-9-6-10-8(3-4-13-10)7(9)2-1-5-15(11)12/h1-6,13H
B-40	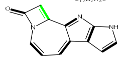 $C_{12}H_7N_3O$	0.7982	87.0260	−0.5086	−5.7600	72.4208	InChI=1S/C12H7N3O/c16-10-6-9-11-7(2-1-5-15(9)10)8-3-4-13-12(8)14-11/h1-6,13H
H11-2	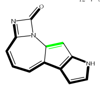 $C_{12}H_7N_3O$	1.2809	92.7054	−0.5258	−12.48	72.6091	InChI=1S/C12H7N3O/c16-12-14-11-3-1-2-8-7-4-5-13-9(7)6-10(8)15(11)12/h1-6,13H

C-66	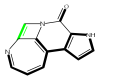 $C_{12}H_7N_3O$	1.7689	79.1033	-0.3733	-5.7000	74.4022	InChI=1S/C12H7N3O/c16-12-10-7(3-5-14-10)8-2-1-4-13-9-6-15(12)11(8)9/h1-6,14H
A-159	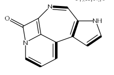 $C_{12}H_7N_3O$	1.5223	82.2859	-0.2297	-7.69	75.4238	InChI=1S/C12H7N3O/c16-12-10-11-8(2-1-5-15(11)12)7-3-4-13-9(7)6-14-10/h1-6,13H
G1-97	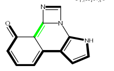 $C_{12}H_7N_3O$	1.0219	78.9743	-0.5496	-6.04	77.8141	InChI=1S/C12H7N3O/c16-9-3-1-2-7-8-4-5-13-11(8)15-6-14-12(15)10(7)9/h1-6,13H
B-13	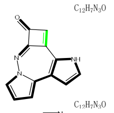 $C_{12}H_7N_3O$	1.0570	79.4923	-0.7905	-6.8300	78.5829	InChI=1S/C12H7N3O/c16-10-6-8-11-7(3-4-13-11)9-2-1-5-15(9)14-12(8)10/h1-6,13H
G1-60	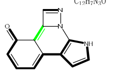 $C_{12}H_7N_3O$	1.1444	77.2593	-0.6814	-7.1	79.5073	InChI=1S/C12H7N3O/c16-10-3-1-2-7-8-4-5-13-12(8)15-9(6-14-15)11(7)10/h1-6,13H
B-30	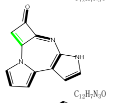 $C_{12}H_7N_3O$	0.9009	77.6844	-0.7592	-4.9700	79.6273	InChI=1S/C12H7N3O/c16-10-6-9-11(10)14-12-7(3-4-13-12)8-2-1-5-15(8)9/h1-6,13H
G-19	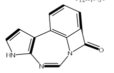 $C_{12}H_7N_3O$	2.0473	89.6073	-0.2082	-7.83	79.7694	InChI=1S/C12H7N3O/c16-12-9-3-1-2-7-8-4-5-13-11(8)14-6-15(12)10(7)9/h1-6,13H
G-18	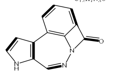 $C_{12}H_7N_3O$	1.7607	86.3927	-0.2874	-9.82	80.1332	InChI=1S/C12H7N3O/c16-12-9-3-1-2-8-7-4-5-13-10(7)6-14-15(12)11(8)9/h1-6,13H
B-190	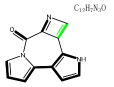 $C_{12}H_7N_3O$	1.5813	82.5811	-0.8318	-7.8300	80.1895	InChI=1S/C12H7N3O/c16-12-11-8(6-14-11)10-7(3-4-13-10)9-2-1-5-15(9)12/h1-6,13H
o-81	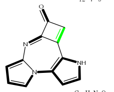 $C_{12}H_7N_3O$	1.244	78.5981	-0.8352	-3.91	80.5002	InChI=1S/C12H7N3O/c16-9-6-7-11-8(3-4-13-11)15-5-1-2-10(15)14-12(7)9/h1-6,13H
H-103	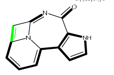 $C_{12}H_7N_3O$	1.7507	72.8837	-0.4563	-7.96	80.6798	InChI=1S/C12H7N3O/c16-12-11-8(4-5-13-11)9-3-1-2-7-6-10(14-12)15(7)9/h1-6,13H
H1-53	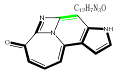 $C_{12}H_7N_3O$	0.912	79.7585	-0.3846	-7.83	81.0946	InChI=1S/C12H7N3O/c16-10-3-1-2-9-7-4-5-13-8(7)6-11-14-12(10)15(9)11/h1-6,13H
C-17	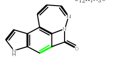 $C_{12}H_7N_3O$	1.4916	88.4519	-0.3243	-9.8200	81.8663	InChI=1S/C12H7N3O/c16-12-9-6-10-7(3-5-13-10)8-2-1-4-14-15(12)11(8)9/h1-6,13H
H11-156	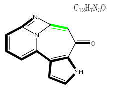 $C_{12}H_7N_3O$	1.5683	79.0011	-0.3076	-5.84	82.1499	InChI=1S/C12H7N3O/c16-9-6-11-14-10-3-1-2-8(15(10)11)7-4-5-13-12(7)9/h1-6,13H
B-128	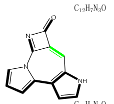 $C_{12}H_7N_3O$	1.1807	82.3227	-0.8142	-7.1000	82.1757	InChI=1S/C12H7N3O/c16-12-8-6-9-7(3-4-13-9)10-2-1-5-15(10)11(8)14-12/h1-6,13H
A-50	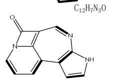 $C_{12}H_7N_3O$	2.0704	89.0358	-0.1434	-4.97	82.4838	InChI=1S/C12H7N3O/c16-12-9-6-14-11-8(3-4-13-11)7-2-1-5-15(12)10(7)9/h1-6,13H
H1-139	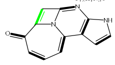 $C_{12}H_7N_3O$	1.1673	75.4039	-0.4689	-2.2499	83.0307	InChI=1S/C12H7N3O/c16-10-3-1-2-8-7-4-5-13-12(7)14-11-6-9(10)15(8)11/h1-6,13H
F-34	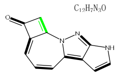 $C_{12}H_7N_3O$	0.9843	85.2549	-0.6054	-5.17	83.3222	InChI=1S/C12H7N3O/c16-11-6-10-7(11)2-1-3-9-8-4-5-13-12(8)14-15(9)10/h1-6H,(H,13,14)
B-177	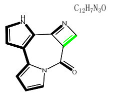 $C_{12}H_7N_3O$	1.5548	85.6303	-0.8151	-5.1100	83.8586	InChI=1S/C12H7N3O/c16-12-8-6-14-11(8)10-7(3-4-13-10)9-2-1-5-15(9)12/h1-6,13H

H-96	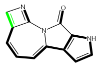 $C_{12}H_7N_3O$	1.445	89.9564	-0.6485	-8.89	84.2638	InChI=1S/C12H7N3O/c16-12-10-8(4-5-13-10)9-3-1-2-7-6-14-11(7)15(9)12/h1-6,13H
A2-65	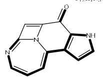 $C_{12}H_7N_3O$	1.1108	75.2129	-0.3873	-2.25	84.631	InChI=1S/C12H7N3O/c16-12-9-6-10-13-4-1-2-8(15(9)10)7-3-5-14-11(7)12/h1-6,14H
I-85	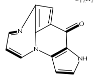 $C_{12}H_7N_3O$	1.6193	72.7872	-0.2913	-3.05	84.9833	InChI=1S/C12H7N3O/c16-12-7-6-8-11(7)15(5-1-3-13-8)9-2-4-14-10(9)12/h1-6,14H
D-15	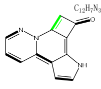 $C_{12}H_7N_3O$	1.6918	79.0777	-0.6035	-5.1700	85.0092	InChI=1S/C12H7N3O/c16-10-6-9-11(10)12-7(3-5-13-12)8-2-1-4-14-15(8)9/h1-6,13H
o-152	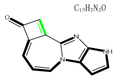 $C_{12}H_7N_3O$	0.9309	86.2884	-0.655	-3.18	85.3147	InChI=1S/C12H7N3O/c16-10-6-8-7(10)2-1-5-15-9-3-4-13-11(9)14-12(8)15/h1-6,13H
G-37	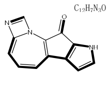 $C_{12}H_7N_3O$	0.9748	90.3349	-0.5259	-7.7	85.7224	InChI=1S/C12H7N3O/c16-12-10-7(4-5-13-10)8-2-1-3-9-14-6-15(9)11(8)12/h1-6,13H
F-32	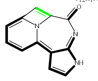 $C_{12}H_7N_3O$	1.3256	77.2465	-0.376	-5.3	86.0534	InChI=1S/C12H7N3O/c16-12-10-6-7-2-1-3-9(15(7)10)8-4-5-13-11(8)14-12/h1-6H,(H,13,14,16)
H11-82	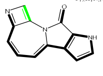 $C_{12}H_7N_3O$	1.3344	91.8828	-0.6199	-7.83	86.6297	InChI=1S/C12H7N3O/c16-12-11-7(4-5-13-11)9-3-1-2-8-10(6-14-8)15(9)12/h1-6,13H
B-175	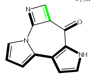 $C_{12}H_7N_3O$	0.9905	82.8896	-0.7618	-3.2500	86.9107	InChI=1S/C12H7N3O/c16-11-8-6-14-12(8)15-5-1-2-9(15)7-3-4-13-10(7)11/h1-6,13H
B-194	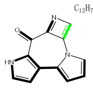 $C_{12}H_7N_3O$	0.9614	81.7212	-0.7108	-5.9700	87.1129	InChI=1S/C12H7N3O/c16-12-10-7(3-4-13-10)8-2-1-5-15(8)9-6-14-11(9)12/h1-6,13H
G-82	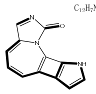 $C_{12}H_7N_3O$	2.284	96.201	-0.297	-12.61	87.436	InChI=1S/C12H7N3O/c16-12-14-6-7-2-1-3-9-8-4-5-13-10(8)11(9)15(7)12/h1-6,13H
x4-8	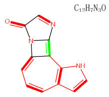 $C_{12}H_7N_3O$	3.0846	101.9716	-0.3211	-8.56	87.5633	InChI=1S/C12H7N3O/c16-9-6-14-12-10-8(15(9)12)3-1-2-7-4-5-13-11(7)10/h1-6,13H
A-193	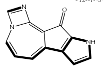 $C_{12}H_7N_3O$	0.8324	90.9855	-0.5203	-6.04	87.7987	InChI=1S/C12H7N3O/c16-11-9-7(8-3-4-13-10(8)11)2-1-5-15-6-14-12(9)15/h1-6,13H
G-36	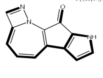 $C_{12}H_7N_3O$	1.2165	88.634	-0.6581	-9.82	88.9444	InChI=1S/C12H7N3O/c16-12-10-8(4-5-13-10)9-3-1-2-7-6-14-15(7)11(9)12/h1-6,13H
n-130	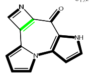 $C_{12}H_7N_3O$	1.3745	83.0542	-0.7384	-5.97	88.9725	InChI=1S/C12H7N3O/c16-12-10-7(6-14-10)8-2-1-5-15(8)9-3-4-13-11(9)12/h1-6,13H
A-100	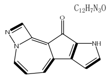 $C_{12}H_7N_3O$	1.0557	89.4209	-0.7002	-7.1	89.1989	InChI=1S/C12H7N3O/c16-12-10-7(8-3-4-13-11(8)12)2-1-5-15-9(10)6-14-15/h1-6,13H
o-212	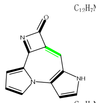 $C_{12}H_7N_3O$	1.686	85.8072	-0.8108	-5.17	89.4196	InChI=1S/C12H7N3O/c16-12-7-6-8-9(3-4-13-8)15-5-1-2-10(15)11(7)14-12/h1-6,13H
I-62	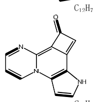 $C_{12}H_7N_3O$	1.9738	79.8974	-0.6684	-2.25	89.4926	InChI=1S/C12H7N3O/c16-9-6-7-10(9)12-14-3-1-5-15(12)8-2-4-13-11(7)8/h1-6,13H
B-212	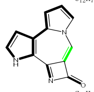 $C_{12}H_7N_3O$	1.6979	86.0119	-0.8287	-6.0400	89.4981	InChI=1S/C12H7N3O/c16-12-8-6-15-5-1-2-9(15)7-3-4-13-10(7)11(8)14-12/h1-6,13H

o-220	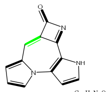 $C_{12}H_7N_3O$	1.9073	86.5404	-0.8223	-6.17	89.8284	InChI=1S/C12H7N3O/c16-12-8-6-7-2-1-5-15(7)9-3-4-13-11(9)10(8)14-12/h1-6,13H
A-93	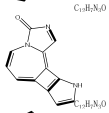 $C_{12}H_7N_3O$	2.4599	97.9078	-0.3783	-12.48	89.9282	InChI=1S/C12H7N3O/c16-12-14-6-9-10-7(2-1-5-15(9)12)8-3-4-13-11(8)10/h1-6,13H
G-74	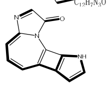 $C_{12}H_7N_3O$	2.3269	97.8878	-0.3522	-10.49	90.5347	InChI=1S/C12H7N3O/c16-10-6-14-9-3-1-2-8-7-4-5-13-11(7)12(8)15(9)10/h1-6,13H
C-13	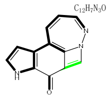 $C_{12}H_7N_3O$	1.6486	83.4726	-0.2891	-5.0400	91.6469	InChI=1S/C12H7N3O/c16-12-9-6-15-11(9)8(2-1-4-14-15)7-3-5-13-10(7)12/h1-6,13H
o-12	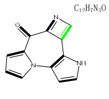 $C_{12}H_7N_3O$	1.7845	83.452	-0.748	-3.25	92.16	InChI=1S/C12H7N3O/c16-12-9-2-1-5-15(9)8-3-4-13-10(8)7-6-14-11(7)12/h1-6,13H
A-29	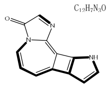 $C_{12}H_7N_3O$	2.3045	98.5626	-0.3666	-9.49	92.7921	InChI=1S/C12H7N3O/c16-9-6-14-12-10-7(2-1-5-15(9)12)8-3-4-13-11(8)10/h1-6,13H
q11-33	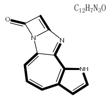 $C_{12}H_7N_3O$	3.0117	105.9451	-0.2067	-9.56	93.2161	InChI=1S/C12H7N3O/c16-10-6-9-14-12-8(15(9)10)3-1-2-7-4-5-13-11(7)12/h1-6,13H
n-168	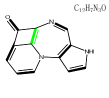 $C_{12}H_7N_3O$	1.679	80.5456	-0.2721	-2.25	93.639	InChI=1S/C12H7N3O/c16-12-7-2-1-5-15-9-3-4-13-8(9)6-14-10(12)11(7)15/h1-6,13H
s11-45	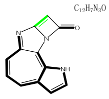 $C_{12}H_7N_3O$	3.0985	106.4455	-0.25	-9.56	93.6818	InChI=1S/C12H7N3O/c16-10-6-9-14-8-3-1-2-7-4-5-13-11(7)12(8)15(9)10/h1-6,13H
n-129	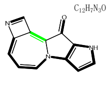 $C_{12}H_7N_3O$	1.2359	89.5579	-0.636	-4.18	94.2099	InChI=1S/C12H7N3O/c16-12-10-9(3-4-13-10)15-5-1-2-8-7(6-14-8)11(12)15/h1-6,13H
o-177	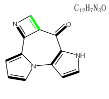 $C_{12}H_7N_3O$	1.5068	86.1471	-0.7589	-1.32	94.5221	InChI=1S/C12H7N3O/c16-12-7-6-14-10(7)8-2-1-5-15(8)9-3-4-13-11(9)12/h1-6,13H
x4-58	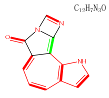 $C_{12}H_7N_3O$	3.9161	108.2528	-0.251	-6.9	94.6147	InChI=1S/C12H7N3O/c16-12-8-3-1-2-7-4-5-13-10(7)9(8)11-14-6-15(11)12/h1-6,13H
I-37	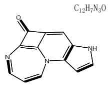 $C_{12}H_7N_3O$	1.466	82.2125	-0.3364	-2.25	94.8563	InChI=1S/C12H7N3O/c16-12-7-6-8-9(2-4-13-8)15-5-1-3-14-10(12)11(7)15/h1-6,13H
n-136	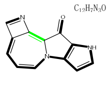 $C_{12}H_7N_3O$	1.3111	89.4414	-0.6109	-5.24	94.9039	InChI=1S/C12H7N3O/c16-12-10-8(3-4-13-10)15-5-1-2-7-6-14-9(7)11(12)15/h1-6,13H
q-65	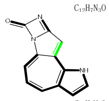 $C_{12}H_7N_3O$	3.8442	110.3677	-0.2714	-11.55	95.068	InChI=1S/C12H7N3O/c16-12-14-10-6-8-9(15(10)12)3-1-2-7-4-5-13-11(7)8/h1-6,13H
F-11	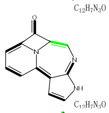 $C_{12}H_7N_3O$	1.5166	84.39	-0.3455	-1.52	95.0843	InChI=1S/C12H7N3O/c16-11-9-3-1-2-8-7-4-5-13-12(7)14-6-10(11)15(8)9/h1-6H,(H,13,14)
o-120	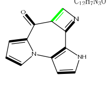 $C_{12}H_7N_3O$	1.818	86.4643	-0.7624	-0.53	95.9977	InChI=1S/C12H7N3O/c16-12-7-6-14-10(7)11-8(3-4-13-11)15-5-1-2-9(12)15/h1-6,13H
G-14	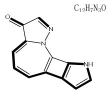 $C_{12}H_7N_3O$	2.2582	95.4504	-0.2845	-8.83	96.8994	InChI=1S/C12H7N3O/c16-10-6-14-15-9(10)3-1-2-8-7-4-5-13-11(7)12(8)15/h1-6,13H
q-66	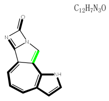 $C_{12}H_7N_3O$	3.9336	112.4572	-0.2893	-11.61	97.2231	InChI=1S/C12H7N3O/c16-12-14-11-8-3-1-2-7-4-5-13-10(7)9(8)6-15(11)12/h1-6,13H

o-165	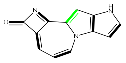	1.5203	94.507	-0.6811	-5.17	97.6925	InChI=1S/C12H7N3O/c16-12-7-2-1-5-15-9-3-4-13-8(9)6-10(15)11(7)14-12/h1-6,13H
A-5	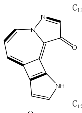	2.3393	96.6582	-0.371	-8.83	98.2585	InChI=1S/C12H7N3O/c16-9-6-14-15-5-1-2-7-8-3-4-13-11(8)10(7)12(9)15/h1-6,13H
A-91	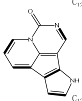	1.7078	52.2594	-0.3866	32.0397	99.635	InChI=1S/C12H7N3O/c16-12-14-6-9-10-7(3-4-13-10)8-2-1-5-15(12)11(8)9/h1-6,13H
G-33	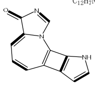	2.3699	99.4261	-0.3702	-8.83	100.6877	InChI=1S/C12H7N3O/c16-12-9-3-1-2-8-7-4-5-13-10(7)11(8)15(9)6-14-12/h1-6,13H
n-169	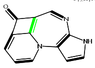	2.2114	87.2917	-0.1542	0.47	100.7267	InChI=1S/C12H7N3O/c16-11-7-2-1-5-15-9-3-4-13-12(9)14-6-8(11)10(7)15/h1-6,13H
A-214	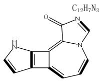	2.3372	100.4604	-0.4130	-8.8300	102.1544	InChI=1S/C12H7N3O/c16-12-11-9-7(8-3-4-13-10(8)9)2-1-5-15(11)6-14-12/h1-6,13H
x4-52	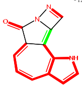	3.959	109.8063	-0.2959	-9.89	102.3922	InChI=1S/C12H7N3O/c16-12-8-3-1-2-7-4-5-13-11(7)10(8)9-6-14-15(9)12/h1-6,13H
A-31	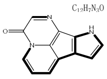	1.4793	50.2142	-0.3625	36.679	102.7852	InChI=1S/C12H7N3O/c16-9-6-14-11-10-7(3-4-13-10)8-2-1-5-15(9)12(8)11/h1-6,13H
n-192	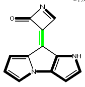	0.8701	107.2225	-1.0434	-6.17	102.8584	InChI=1S/C12H7N3O/c16-12-7(6-14-12)10-8-2-1-5-15(8)9-3-4-13-11(9)10/h1-6,13H/b10-7+
x4-2	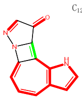	2.3628	103.7329	-0.4647	-8.96	103.0488	InChI=1S/C12H7N3O/c16-9-6-14-15-8-3-1-2-7-4-5-13-11(7)10(8)12(9)15/h1-6,13H
s11-43	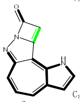	1.3674	73.165	-1.7878	31.5974	104.3785	InChI=1S/C12H7N3O/c16-10-6-9-11-8(14-15(9)10)3-1-2-7-4-5-13-12(7)11/h1-6,13H
q11-37	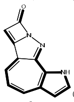	1.3653	73.6564	-1.7615	30.1092	104.7756	InChI=1S/C12H7N3O/c16-10-6-9-8-3-1-2-7-4-5-13-11(7)12(8)14-15(9)10/h1-6,13H
q-6	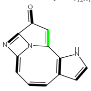	2.3066	108.5089	-0.3431	-7.83	104.9584	InChI=1S/C12H7N3O/c16-9-6-8-11-7(4-5-13-11)2-1-3-10-14-12(9)15(8)10/h1-6,13H/b2-1-,10-3-
A-137	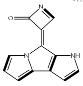	1.1253	108.9979	-1.0393	-6.17	105.2014	InChI=1S/C12H7N3O/c16-12-8(6-14-12)11-10-7(3-4-13-10)9-2-1-5-15(9)11/h1-6,13H/b11-8-
A-53	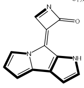	1.0628	108.1536	-1.0467	-5.24	105.2761	InChI=1S/C12H7N3O/c16-12-8(6-14-12)11-10-7(3-4-13-10)9-2-1-5-15(9)11/h1-6,13H/b11-8+
n-110	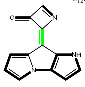	0.8206	105.7112	-1.0454	-3.18	105.3514	InChI=1S/C12H7N3O/c16-9-6-14-12(9)10-7-2-1-5-15(7)8-3-4-13-11(8)10/h1-6,13H/b12-10-
H-56	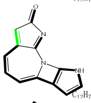	2.9978	106.9247	-0.1965	-7.89	105.8668	InChI=1S/C12H7N3O/c16-10-6-7-2-1-3-9-8-4-5-13-12(8)15(9)11(7)14-10/h1-6,13H
A-11	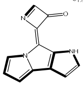	1.1011	107.603	-1.0384	-3.18	108.3786	InChI=1S/C12H7N3O/c16-9-6-14-11(9)12-10-7(3-4-13-10)8-2-1-5-15(8)12/h1-6,13H/b12-11-
H11-108	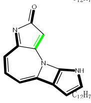	3.0223	107.6649	-0.2252	-6.83	108.4432	InChI=1S/C12H7N3O/c16-11-6-10-8(14-11)2-1-3-9-7-4-5-13-12(7)15(9)10/h1-6,13H

q-60	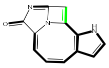	$C_{12}H_7N_3O$	2.9096	106.833	-0.5107	-6.17	109.9364	InChI=1S/C12H7N3O/c16-12-8-3-1-2-7-4-5-13-11(7)9-6-10(14-12)15(8)9/h1-6,13H/b2-1-,8-3-
s11-60	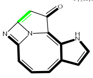	$C_{12}H_7N_3O$	2.7662	111.0263	-0.3239	-5.11	110.0418	InChI=1S/C12H7N3O/c16-8-6-10-14-9-3-1-2-7-4-5-13-11(7)12(8)15(9)10/h1-6,13H/b3-1-,7-2-,12-11+
n-111	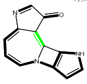	$C_{12}H_7N_3O$	2.5506	103.7403	-0.2097	-3.12	110.2534	InChI=1S/C12H7N3O/c16-9-6-14-7-2-1-5-15-8-3-4-13-11(8)12(15)10(7)9/h1-6,13H
q11-39	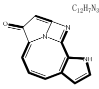	$C_{12}H_7N_3O$	2.689	111.2961	-0.3554	-5.11	110.33	InChI=1S/C12H7N3O/c16-9-6-10-14-12-11-7(4-5-13-11)2-1-3-8(9)15(10)12/h1-6,13H/b2-1-,8-3-
n-124	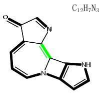	$C_{12}H_7N_3O$	2.5201	103.2878	-0.1805	-2.12	111.0444	InChI=1S/C12H7N3O/c16-9-6-14-10-7(9)2-1-5-15-8-3-4-13-11(8)12(10)15/h1-6,13H
x4-64	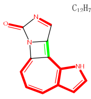	$C_{12}H_7N_3O$	1.5564	67.7639	-1.6827	47.1595	111.0755	InChI=1S/C12H7N3O/c16-12-14-6-9-10-8(15(9)12)3-1-2-7-4-5-13-11(7)10/h1-6,13H
G1-40	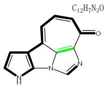	$C_{12}H_7N_3O$	6.5832	107.3583	-1.6272	-7.7	112.324	InChI=1S/C12H7N3O/c16-9-3-1-2-7-8-4-5-13-12(8)15-6-14-10(9)11(7)15/h1-6,13H
x4-48	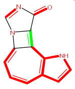	$C_{12}H_7N_3O$	2.869	113.6808	-0.4064	-8.96	112.5396	InChI=1S/C12H7N3O/c16-12-11-9-8(15(11)6-14-12)3-1-2-7-4-5-13-10(7)9/h1-6,13H
n-195	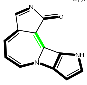	$C_{12}H_7N_3O$	2.6158	106.6636	-0.2669	-4.24	112.7755	InChI=1S/C12H7N3O/c16-12-9-7(6-14-12)2-1-5-15-8-3-4-13-10(8)11(9)15/h1-6,13H
n-209	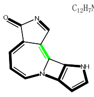	$C_{12}H_7N_3O$	2.6741	106.4948	-0.2824	-4.11	112.8499	InChI=1S/C12H7N3O/c16-12-7-2-1-5-15-9-3-4-13-10(9)11(15)8(7)6-14-12/h1-6,13H
q-13	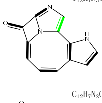	$C_{12}H_7N_3O$	2.2015	110.804	-0.4099	-6.17	112.8842	InChI=1S/C12H7N3O/c16-11-8-3-1-2-7-4-5-13-10(7)9-6-14-12(11)15(8)9/h1-6,13H/b2-1-,8-3-
C-69	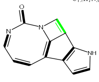	$C_{12}H_7N_3O$	2.7279	128.6412	-0.4780	-10.5500	114.1289	InChI=1S/C12H7N3O/c16-12-14-4-1-2-8-7-3-5-13-10(7)9-6-15(12)11(8)9/h1-6,13H/b2-1-,14-4+
x4-60	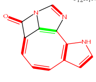	$C_{12}H_7N_3O$	4.6873	124.2552	0.1194	-8.83	114.4048	InChI=1S/C12H7N3O/c16-12-8-3-1-2-7-4-5-13-9(7)10-11(8)15(12)6-14-10/h1-6,13H/b2-1-,3-1-,7-2-,8-3+,10-9-
x4-22	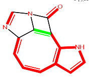	$C_{12}H_7N_3O$	4.6468	125.1605	0.0804	-8.83	114.9034	InChI=1S/C12H7N3O/c16-12-9-10-7(4-5-13-10)2-1-3-8-11(9)15(12)6-14-8/h1-6,13H/b2-1-,3-1-,7-2?,8-3+,10-9-
x4-50	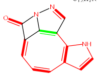	$C_{12}H_7N_3O$	4.1308	125.0374	-0.114	-8.89	118.4166	InChI=1S/C12H7N3O/c16-12-8-3-1-2-7-4-5-13-10(7)9-6-14-15(12)11(8)9/h1-6,13H/b2-1-,3-1-,7-2-,8-3+,10-9+
q11-5	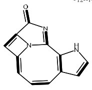	$C_{12}H_7N_3O$	4.0247	117.4642	-0.2412	-7.23	121.1339	InChI=1S/C12H7N3O/c16-12-9-6-8-3-1-2-7-4-5-13-10(7)11(14-12)15(8)9/h1-6,13H/b2-1-,8-3-
s11-29	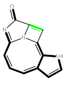	$C_{12}H_7N_3O$	3.9762	118.0425	-0.2667	-6.17	121.948	InChI=1S/C12H7N3O/c16-12-9-6-8-11-7(4-5-13-11)2-1-3-10(14-12)15(8)9/h1-6,13H/b3-1-,7-2-,11-8+
C-21	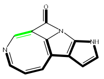	$C_{12}H_7N_3O$	3.0042	133.2379	-0.2830	-4.9700	122.1485	InChI=1S/C12H7N3O/c16-12-9-6-13-4-1-2-7-8-3-5-14-11(8)15(12)10(7)9/h1-6,14H/b2-1-,4-1-,7-2?,9-6+,13-4-,13-6-
H1-140	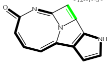	$C_{12}H_7N_3O$	2.5244	119.6951	-0.7109	-6.9	122.8513	InChI=1S/C12H7N3O/c16-11-3-1-2-8-7-4-5-13-12(7)9-6-10(14-11)15(8)9/h1-6,13H/b3-1-,8-2-,14-10-

H-47	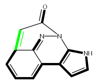 $C_{12}H_7N_3O$	1.6481	28.7932	-0.4814	84.484	124.1106	InChI=1S/C12H7N3O/c16-10-6-7-2-1-3-8-9-4-5-13-12(9)15(10)14-11(7)8/h1-6,13H
F-18	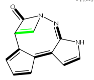 $C_{12}H_7N_3O$	2.0887	77.9619	-1.1284	44.1027	124.3702	InChI=1S/C12H7N3O/c16-12-10-6-15(12)14-11-9(4-5-13-11)7-2-1-3-8(7)10/h1-6H,(H,13,14)/b9-7-
x4-20	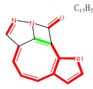 $C_{12}H_7N_3O$	1.1336	84.4457	-1.3435	40.4106	124.7909	InChI=1S/C12H7N3O/c16-12-9-10-7(4-5-13-10)2-1-3-8-6-14-15(12)11(8)9/h1-6,13H/b2-1-,3-1-,7-2?,8-3-,10-9-
A-1	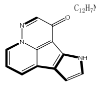 $C_{12}H_7N_3O$	0.9952	54.7149	-0.038	56.4489	126.1308	InChI=1S/C12H7N3O/c16-9-6-14-15-5-1-2-8-7-3-4-13-11(7)10(9)12(8)15/h1-6,13H
A1-4	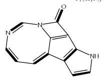 $C_{12}H_7N_3O$	3.2129	138.9044	-0.5068	-7.83	127.0571	InChI=1S/C12H7N3O/c16-12-9-10-7(3-5-14-10)8-2-1-4-13-6-15(12)11(8)9/h1-6,14H/b4-1-,8-2-,13-6-
n-134	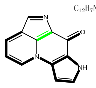 $C_{12}H_7N_3O$	0.8786	46.1517	-0.1079	63.1328	127.2811	InChI=1S/C12H7N3O/c16-12-9-8(3-4-13-9)15-5-1-2-7-6-14-10(12)11(7)15/h1-6,13H
I-102	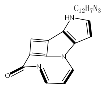 $C_{12}H_7N_3O$	2.7308	121.5186	-0.3719	-5.04	127.7407	InChI=1S/C12H7N3O/c16-12-8-6-7-10-9(2-4-13-10)15(11(7)8)5-1-3-14-12/h1-6,13H/b5-1-,14-3+
D-14	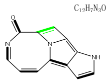 $C_{12}H_7N_3O$	1.6916	122.2259	-0.5548	-4.2400	128.0222	InChI=1S/C12H7N3O/c16-12-10-6-9-11-7(3-5-13-11)8(15(9)10)2-1-4-14-12/h1-6,13H/b2-1-,14-4-
B-105	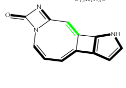 $C_{12}H_7N_3O$	2.8686	148.5867	-0.5111	-12.4799	129.7706	InChI=1S/C12H7N3O/c16-12-14-10-6-9-7(2-1-5-15(10)12)8-3-4-13-11(8)9/h1-6,13H/b5-1-,7-2-,9-6+
q11-25	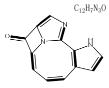 $C_{12}H_7N_3O$	3.6296	124.0211	-0.2448	-3.45	129.9949	InChI=1S/C12H7N3O/c16-11-8-3-1-2-7-4-5-13-10(7)12-14-6-9(11)15(8)12/h1-6,13H/b2-1-,8-3-
s11-8	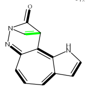 $C_{12}H_7N_3O$	4.068	68.8545	-3.0109	46.9092	130.2112	InChI=1S/C12H7N3O/c16-12-8-6-15(12)14-9-3-1-2-7-4-5-13-11(7)10(8)9/h1-6,13H
n-133	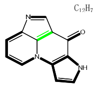 $C_{12}H_7N_3O$	0.8942	47.9496	-0.1088	66.8213	130.2552	InChI=1S/C12H7N3O/c16-12-7-6-14-8-2-1-5-15(11(7)8)9-3-4-13-10(9)12/h1-6,13H
s11-14	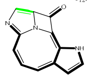 $C_{12}H_7N_3O$	3.5949	124.9767	-0.2794	-3.45	130.4249	InChI=1S/C12H7N3O/c16-12-8-6-14-9-3-1-2-7-4-5-13-10(7)11(12)15(8)9/h1-6,13H/b3-1-,7-2-,11-10+
A-202	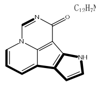 $C_{12}H_7N_3O$	1.0737	56.3958	-0.1513	58.2053	130.6125	InChI=1S/C12H7N3O/c16-12-9-10-7(3-4-13-10)8-2-1-5-15(6-14-12)11(8)9/h1-6,13H
q11-20	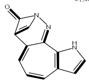 $C_{12}H_7N_3O$	4.1591	69.0939	-3.0512	45.9075	130.6856	InChI=1S/C12H7N3O/c16-12-9-6-15(12)14-11-8(9)3-1-2-7-4-5-13-10(7)11/h1-6,13H
H11-3	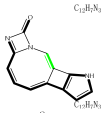 $C_{12}H_7N_3O$	2.9677	149.4584	-0.5274	-12.48	130.7805	InChI=1S/C12H7N3O/c16-12-14-10-3-1-2-7-8-4-5-13-11(8)9(7)6-15(10)12/h1-6,13H/b3-1-,7-2-,9-6+
C-32	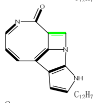 $C_{12}H_7N_3O$	1.9183	128.6555	-0.4576	-5.0400	130.9211	InChI=1S/C12H7N3O/c16-12-9-6-15-10(9)7(2-1-4-14-12)8-3-5-13-11(8)15/h1-6,13H/b2-1-,14-4+
B-57	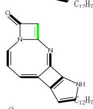 $C_{12}H_7N_3O$	2.6619	147.1573	-0.5574	-8.5600	132.5643	InChI=1S/C12H7N3O/c16-10-6-9-14-12-7(2-1-5-15(9)10)8-3-4-13-11(8)12/h1-6,13H/b5-1-,7-2-,14-12+
B-241	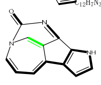 $C_{12}H_7N_3O$	2.9513	44.9555	-0.5605	77.5437	134.7103	InChI=1S/C12H7N3O/c16-12-14-11-9-6-15(12)5-1-2-7(9)8-3-4-13-10(8)11/h1-6,13H

H-51	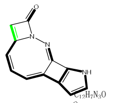 $C_{12}H_7N_3O$	2.6408	148.4282	-0.5944	-11.6099	135.6414	InChI=1S/C12H7N3O/c16-10-6-7-2-1-3-8-9-4-5-13-11(9)12(8)14-15(7)10/h1-6,13H/b2-1-,8-3-,14-12+
C-23	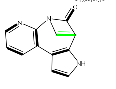 $C_{12}H_7N_3O$	3.2976	61.9535	-1.8659	65.6327	135.9833	InChI=1S/C12H7N3O/c16-12-9-6-15(12)11-8(2-1-4-14-11)7-3-5-13-10(7)9/h1-6,13H
G-54	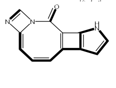 $C_{12}H_7N_3O$	2.6609	154.4301	-0.7693	-8.83	136.2258	InChI=1S/C12H7N3O/c16-12-10-7(8-4-5-13-11(8)10)2-1-3-9-14-6-15(9)12/h1-6,13H/b2-1-,9-3-
H-105	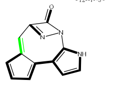 $C_{12}H_7N_3O$	2.9182	76.9794	-1.4983	58.6159	137.5541	InChI=1S/C12H7N3O/c16-12-10-6-7-2-1-3-8(7)9-4-5-13-11(9)15(12)14-10/h1-6,13H/b7-6-
C-20	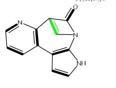 $C_{12}H_7N_3O$	3.1491	65.7909	-1.7024	66.4517	137.7385	InChI=1S/C12H7N3O/c16-12-9-6-15(12)11-8(3-5-14-11)7-2-1-4-13-10(7)9/h1-6,14H
C-45	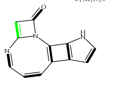 $C_{12}H_7N_3O$	2.6844	152.0928	-0.7452	-8.5600	137.7756	InChI=1S/C12H7N3O/c16-10-6-9-13-4-1-2-8-7-3-5-14-11(7)12(8)15(9)10/h1-6,14H/b2-1-,13-4-
C-40	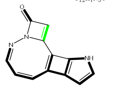 $C_{12}H_7N_3O$	2.5410	151.4197	-0.7800	-11.4800	137.9523	InChI=1S/C12H7N3O/c16-10-6-9-11-7(2-1-4-14-15(9)10)8-3-5-13-12(8)11/h1-6,13H/b2-1-,14-4-
G1-104	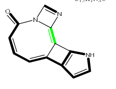 $C_{12}H_7N_3O$	2.7991	156.4763	-0.9461	-9.49	138.6197	InChI=1S/C12H7N3O/c16-9-3-1-2-7-8-4-5-13-11(8)10(7)12-14-6-15(9)12/h1-6,13H/b3-1-,7-2-,12-10+
o-159	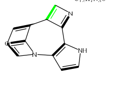 $C_{12}H_7N_3O$	1.0971	77.5375	-0.871	61.5742	138.9759	InChI=1S/C12H7N3O/c16-12-7-2-1-5-15(12)9-3-4-13-11(9)10-8(7)6-14-10/h1-6,13H
D-6	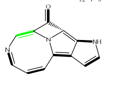 $C_{12}H_7N_3O$	2.2250	132.6663	-0.4987	-1.5200	140.1740	InChI=1S/C12H7N3O/c16-12-9-6-13-4-1-2-8-7-3-5-14-10(7)11(12)15(8)9/h1-6,14H/b2-1-,9-6-,13-4-
H-24	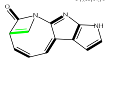 $C_{12}H_7N_3O$	1.7686	84.6723	-1.022	58.9871	140.4791	InChI=1S/C12H7N3O/c16-12-7-2-1-3-9-8-4-5-13-10(8)14-11(9)15(12)6-7/h1-6,13H/b2-1-,9-3-
H-118	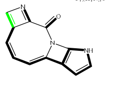 $C_{12}H_7N_3O$	2.3503	156.7711	-0.8374	-8.89	140.8419	InChI=1S/C12H7N3O/c16-12-10-7(6-14-10)2-1-3-9-8-4-5-13-11(8)15(9)12/h1-6,13H/b2-1-,9-3-
H1-77	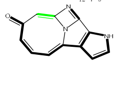 $C_{12}H_7N_3O$	4.7748	139.4743	-1.8664	-4.84	140.9399	InChI=1S/C12H7N3O/c16-7-2-1-3-9-8-4-5-13-11(8)12-14-10(6-7)15(9)12/h1-6,13H/b2-1-,9-3-,10-6-
G-78	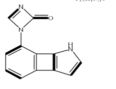 $C_{12}H_7N_3O$	4.4797	157.8209	-1.964	-13.2753	141.703	InChI=1S/C12H7N3O/c16-12-14-6-15(12)9-3-1-2-7-8-4-5-13-11(8)10(7)9/h1-6,13H
H11-11	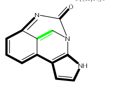 $C_{12}H_7N_3O$	1.994	30.9462	-0.998	99.6897	142.0054	InChI=1S/C12H7N3O/c16-12-14-10-3-1-2-7-8-4-5-13-11(8)15(12)6-9(7)10/h1-6,13H
G-69	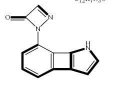 $C_{12}H_7N_3O$	4.379	155.7441	-2.0578	-13.2796	142.86	InChI=1S/C12H7N3O/c16-10-6-14-15(10)9-3-1-2-7-8-4-5-13-12(8)11(7)9/h1-6,13H
q-17	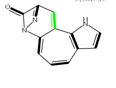 $C_{12}H_7N_3O$	4.2168	68.1477	-3.2131	61.2493	142.9036	InChI=1S/C12H7N3O/c16-12-9-6-8-10(15(12)14-9)3-1-2-7-4-5-13-11(7)8/h1-6,13H
B-216	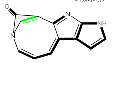 $C_{12}H_7N_3O$	2.4789	82.2492	-1.1737	63.5064	143.2974	InChI=1S/C12H7N3O/c16-12-9-6-15(12)5-1-2-7-8-3-4-13-11(8)14-10(7)9/h1-6,13H/b5-1-,7-2-
A2-62	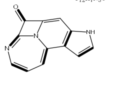 $C_{12}H_7N_3O$	2.5644	86.1721	-1.5585	43.4267	144.0817	InChI=1S/C12H7N3O/c16-11-10-6-8-7(3-5-13-8)9-2-1-4-14-12(11)15(9)10/h1-6,13H

q-19	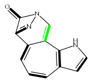 $C_{12}H_7N_3O$	4.5819	69.6001	-3.3022	60.8835	144.5361	InChI=1S/C12H7N3O/c16-12-11-8-3-1-2-7-4-5-13-10(7)9(8)6-15(12)14-11/h1-6,13H
H11-56	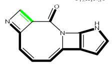 $C_{12}H_7N_3O$	2.3778	159.1064	-0.8929	-5.11	144.5552	InChI=1S/C12H7N3O/c16-12-8-6-14-9(8)2-1-3-10-7-4-5-13-11(7)15(10)12/h1-6,13H/b2-1-,10-3-
G-50	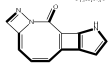 $C_{12}H_7N_3O$	2.6133	154.234	-0.761	-10.9499	144.8616	InChI=1S/C12H7N3O/c16-12-10-8(9-4-5-13-11(9)10)3-1-2-7-6-14-15(7)12/h1-6,13H/b3-1-,7-2-
o-156	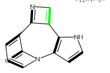 $C_{12}H_7N_3O$	1.1137	78.1529	-0.8374	67.818	145.7529	InChI=1S/C12H7N3O/c16-12-7-2-1-5-15(12)9-3-4-13-11(9)8-6-14-10(7)8/h1-6,13H
B-137	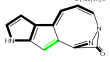 $C_{12}H_7N_3O$	3.2711	84.5124	-1.4540	58.3158	146.0074	InChI=1S/C12H7N3O/c16-12-11-9-6-10-8(3-4-13-10)7(9)2-1-5-15(12)14-11/h1-6,13H/b5-1-,7-2-
G1-66	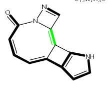 $C_{12}H_7N_3O$	2.8169	156.3919	-0.9602	-12.48	146.442	InChI=1S/C12H7N3O/c16-10-3-1-2-7-8-4-5-13-12(8)11(7)9-6-14-15(9)10/h1-6,13H/b3-1-,7-2-,11-9+
H-94	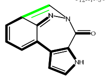 $C_{12}H_7N_3O$	2.3892	32.7235	-0.399	96.9511	147.7955	InChI=1S/C12H7N3O/c16-12-11-9(4-5-13-11)8-3-1-2-7-6-15(12)14-10(7)8/h1-6,13H
H1-66	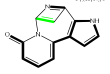 $C_{12}H_7N_3O$	3.2496	48.1462	-1.9478	93.1193	148.1237	InChI=1S/C12H7N3O/c16-11-3-1-2-9-7-4-5-13-12(7)8-6-10(14-8)15(9)11/h1-6,13H
H11-46	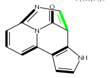 $C_{12}H_7N_3O$	2.8633	29.846	29.846	107.7825	148.1649	InChI=1S/C12H7N3O/c16-12-8-6-14-10-3-1-2-9(15(10)12)7-4-5-13-11(7)8/h1-6,13H
H11-14	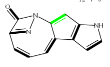 $C_{12}H_7N_3O$	2.7415	87.1542	-1.4825	59.5647	149.0216	InChI=1S/C12H7N3O/c16-12-9-3-1-2-8-7-4-5-13-10(7)6-11(8)15(12)14-9/h1-6,13H/b3-1-,8-2-
p-7	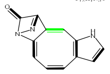 $C_{12}H_7N_3O$	4.5254	91.5109	-4.6569	54.6639	150.7167	InChI=1S/C12H7N3O/c16-12-11-8-6-9-7(4-5-13-9)2-1-3-10(8)15(12)14-11/h1-6,13H/b2-1-,3-1-,7-2-,8-6+,9-6+,10-3+
p-9	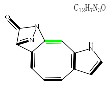 $C_{12}H_7N_3O$	4.5194	91.6308	-4.6656	54.6701	150.8581	InChI=1S/C12H7N3O/c16-12-11-8-3-1-2-7-4-5-13-9(7)6-10(8)15(12)14-11/h1-6,13H/b2-1-,3-1-,7-2-,8-3+,9-6+,10-6+
I-20	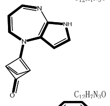 $C_{12}H_7N_3O$	7.3872	59.0496	-7.9195	71.0039	152.7793	InChI=1S/C12H7N3O/c16-11-7-6-8(11)10(7)15-5-1-3-13-12-9(15)2-4-14-12/h1-6,14H
H-37	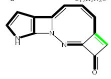 $C_{12}H_7N_3O$	3.4887	155.3092	-0.5153	-7.89	152.992	InChI=1S/C12H7N3O/c16-10-6-7-2-1-3-9-8-4-5-13-12(8)15(9)14-11(7)10/h1-6,13H/b2-1-,9-3-,14-11+
X22-18	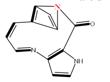 $C_{12}H_7N_3O$	7.0901	68.7746	-7.7869	71.3871	153.5187	InChI=1S/C12H7N3O/c16-12-11-8(3-5-14-11)13-4-1-2-7-9-6-10(7)15(9)12/h1-6,14H/b2-1-,13-4+
r-10	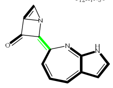 $C_{12}H_7N_3O$	17.3588	123.7754	-14.6496	16.8223	155.1197	InChI=1S/C12H7N3O/c16-11-9-6-15(9)10(11)8-3-1-2-7-4-5-13-12(7)14-8/h1-6H,(H,13,14)/b10-8-
o-155	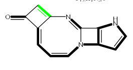 $C_{12}H_7N_3O$	3.106	151.3302	-0.5394	-1.19	156.2356	InChI=1S/C12H7N3O/c16-10-6-8-7(10)2-1-5-15-9-3-4-13-11(9)12(15)14-8/h1-6,13H/b5-1-,7-2+,14-12-
H1-67	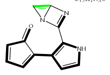 $C_{12}H_7N_3O$	16.7809	126.3095	-14.2019	19.1284	156.8769	InChI=1S/C12H7N3O/c16-9-3-1-2-7(9)8-4-5-13-11(8)12-14-10-6-15(10)12/h1-6,13H
G-43	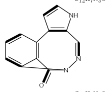 $C_{12}H_7N_3O$	3.3271	90.6008	-1.2005	54.0222	157.0728	InChI=1S/C12H7N3O/c16-12-11-8-2-1-3-10(11)15(12)14-6-9-7(8)4-5-13-9/h1-6,13H/b14-6+

q11-10	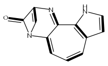 $C_{12}H_7N_3O$	4.2671	65.5292	-3.4197	76.7917	157.2902	InChI=1S/C12H7N3O/c16-12-8-6-15(12)9-3-1-2-7-4-5-13-10(7)11(9)14-8/h1-6,13H
r-53	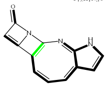 $C_{12}H_7N_3O$	2.7071	167.6187	-1.7583	-7.82	157.3238	InChI=1S/C12H7N3O/c16-10-6-9-8-3-1-2-7-4-5-13-11(7)14-12(8)15(9)10/h1-6H,(H,13,14)
A2-71	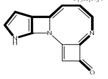 $C_{12}H_7N_3O$	3.2725	158.2689	-0.6628	-4.97	158.8646	InChI=1S/C12H7N3O/c16-10-6-9-11(10)13-4-1-2-8-7-3-5-14-12(7)15(8)9/h1-6,14H/b4-1-,8-2-,13-11+
H-132	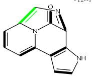 $C_{12}H_7N_3O$	2.798	31.1583	-0.5604	115.6226	159.4333	InChI=1S/C12H7N3O/c16-12-11-10-8(4-5-13-10)9-3-1-2-7(6-14-11)15(9)12/h1-6,13H
C-50	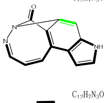 $C_{12}H_7N_3O$	3.1302	94.0206	-1.2338	52.9116	160.0377	InChI=1S/C12H7N3O/c16-12-11-8-2-1-4-14-15(12)10(11)6-9-7(8)3-5-13-9/h1-6,13H/b2-1-,14-4-
X22-46	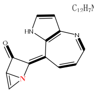 $C_{12}H_7N_3O$	18.0557	125.7998	-14.8688	19.4838	161.0879	InChI=1S/C12H7N3O/c16-12-9-6-15(9)11(12)7-2-1-4-13-8-3-5-14-10(7)8/h1-6,14H/b11-7+
H11-105	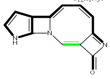 $C_{12}H_7N_3O$	3.6496	160.6467	-0.615	-6.04	161.1676	InChI=1S/C12H7N3O/c16-12-8-6-15-10(3-1-2-9(8)14-12)7-4-5-13-11(7)15/h1-6,13H/b2-1-,8-6+,10-3-
B-192	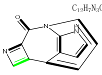 $C_{12}H_7N_3O$	1.5016	80.1299	-1.0333	81.1686	161.6153	InChI=1S/C12H7N3O/c16-12-10-9(6-14-10)7-2-1-5-15(12)11-8(7)3-4-13-11/h1-6,13H
o-78	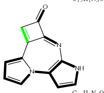 $C_{12}H_7N_3O$	7.8248	148.7515	-1.804	-4.97	162.0732	InChI=1S/C12H7N3O/c16-10-6-7-8-2-1-5-15(8)9-3-4-13-12(9)14-11(7)10/h1-6,13H
o-166	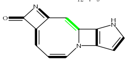 $C_{12}H_7N_3O$	3.2452	158.1813	-0.6059	-5.11	162.2884	InChI=1S/C12H7N3O/c16-12-7-2-1-5-15-9-3-4-13-11(9)10(15)6-8(7)14-12/h1-6,13H/b5-1-,7-2+,10-6-
G1-112	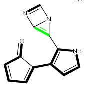 $C_{12}H_7N_3O$	13.9898	127.837	-10.4114	18.1837	162.3633	InChI=1S/C12H7N3O/c16-9-3-1-2-7(9)8-4-5-13-10(8)11-12-14-6-15(11)12/h1-6,13H
s12-29	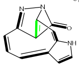 $C_{12}H_7N_3O$	4.3499	87.0169	-4.0771	66.4326	163.1137	InChI=1S/C12H7N3O/c16-12-11-8-3-1-2-7-4-5-13-9(7)6-10(11)15(12)14-8/h1-6,13H/b3-1-,7-2-,9-6+
A-30	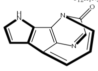 $C_{12}H_7N_3O$	5.4966	50.2811	-1.2698	93.0716	163.2547	InChI=1S/C12H7N3O/c16-9-6-14-11-7-2-1-5-15(9)12(11)10-8(7)3-4-13-10/h1-6,13H
o-119	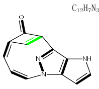 $C_{12}H_7N_3O$	7.531	89.0651	-3.535	164.2332	164.2332	InChI=1S/C12H7N3O/c16-12-7-2-1-5-15-9-3-4-13-11(9)10(14-15)8(12)6-7/h1-6,13H/b5-1-,7-2+
C-9	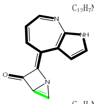 $C_{12}H_7N_3O$	18.7375	127.6179	-14.9205	19.2514	164.7115	InChI=1S/C12H7N3O/c16-11-9-6-15(9)10(11)7-2-1-4-13-12-8(7)3-5-14-12/h1-6,14H/b10-7-
G1-109	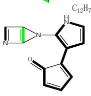 $C_{12}H_7N_3O$	17.6603	128.9041	-11.5244	18.1827	165.9109	InChI=1S/C12H7N3O/c16-10-3-1-2-7(10)8-4-5-13-11(8)15-9-6-14-12(9)15/h1-6,13H
p-29	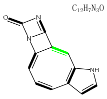 $C_{12}H_7N_3O$	4.0771	106.9416	-4.2777	58.1877	165.9211	InChI=1S/C12H7N3O/c16-12-14-11-8-6-9-7(4-5-13-9)2-1-3-10(8)15(11)12/h1-6,13H/b2-1-,3-1-,7-2-,8-6+,9-6+,10-3+
l-64	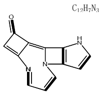 $C_{12}H_7N_3O$	2.9907	157.3911	-0.7352	1.67	165.968	InChI=1S/C12H7N3O/c16-9-6-7-10(9)12-11-8(2-4-14-11)15(12)5-1-3-13-7/h1-6,14H/b5-1-,12-10+,13-3+
l-7	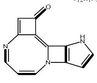 $C_{12}H_7N_3O$	2.9894	157.392	-0.7353	1.67	165.968	InChI=1S/C12H7N3O/c16-9-6-7-10(9)12-11-8(2-4-14-11)15(12)5-1-3-13-7/h1-6,14H/b5-1-,12-10+,13-3-

p-30	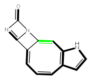 $C_{12}H_7N_3O$	4.075	106.9531	-4.2805	58.1904	166.072	InChI=1S/C12H7N3O/c16-12-14-11-8-3-1-2-7-4-5-13-9(7)6-10(8)15(11)12/h1-6,13H/b2-1-,3-1-,7-2?,8-3+,9-6+,10-6+
p1-41	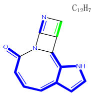 $C_{12}H_7N_3O$	4.5469	176.7374	-1.9815	-7.83	166.2458	InChI=1S/C12H7N3O/c16-9-3-1-2-7-4-5-13-10(7)11-8-6-14-12(8)15(9)11/h1-6,13H/b3-1-,7-2-,11-10+
G1-98	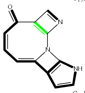 $C_{12}H_7N_3O$	3.4195	165.0788	-0.6594	-3.25	166.8311	InChI=1S/C12H7N3O/c16-10-3-1-2-9-7-4-5-13-11(7)15(9)12-8(10)6-14-12/h1-6,13H/b3-1-,9-2-
i-18	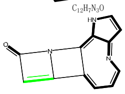 $C_{12}H_7N_3O$	4.1692	108.2632	-4.408	59.6754	167.5354	InChI=1S/C12H7N3O/c16-10-6-9-7-2-1-4-13-8-3-5-14-11(8)12(7)15(9)10/h1-6,14H/b2-1-,4-1-,7-2-,12-11-,13-4+,13-8+
G1-62	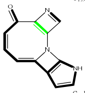 $C_{12}H_7N_3O$	3.5077	164.5886	-0.6669	-5.97	167.7829	InChI=1S/C12H7N3O/c16-10-3-1-2-8-7-4-5-13-12(7)15(8)9-6-14-11(9)10/h1-6,13H/b3-1-,8-2-
H11-7	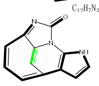 $C_{12}H_7N_3O$	3.0631	37.9922	-0.2656	114.6803	168.173	InChI=1S/C12H7N3O/c16-12-14-9-3-1-2-7-6-10(9)15(12)11-8(7)4-5-13-11/h1-6,13H
q11-27	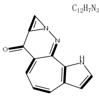 $C_{12}H_7N_3O$	12.137	127.5572	-11.2718	17.3642	168.4159	InChI=1S/C12H7N3O/c16-12-8-3-1-2-7-4-5-13-10(7)11(8)14-15-6-9(12)15/h1-6,13H
B-16	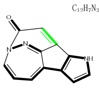 $C_{12}H_7N_3O$	3.2126	50.0954	-1.0409	106.4528	168.4822	InChI=1S/C12H7N3O/c16-10-6-9-11-8(3-4-13-11)7-2-1-5-15(10)14-12(7)9/h1-6,13H
H-152	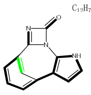 $C_{12}H_7N_3O$	2.5804	84.0007	-1.5158	76.6733	168.9105	InChI=1S/C12H7N3O/c16-12-14-10-8-3-1-2-7(6-8)9-4-5-13-11(9)15(10)12/h1-6,13H
G-79	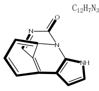 $C_{12}H_7N_3O$	3.8974	33.0044	-1.4275	117.0167	169.0122	InChI=1S/C12H7N3O/c16-12-14-6-9-7-2-1-3-10(9)15(12)11-8(7)4-5-13-11/h1-6,13H
G1-106	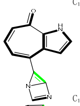 $C_{12}H_7N_3O$	14.3817	126.8967	-10.5145	16.9573	169.0291	InChI=1S/C12H7N3O/c16-9-3-1-2-8(7-4-5-13-10(7)9)11-12-14-6-15(11)12/h1-6,13H
o-252	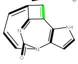 $C_{12}H_7N_3O$	4.6276	65.719	-1.9825	88.4398	169.2713	InChI=1S/C12H7N3O/c16-12-14-10-7-2-1-5-15(12)9-3-4-13-11(9)8(10)6-7/h1-6,13H/b5-1-,7-2-
F-72	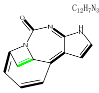 $C_{12}H_7N_3O$	3.1261	53.3931	-2.1278	109.0562	169.8379	InChI=1S/C12H7N3O/c16-12-14-11-9(4-5-13-11)8-3-1-2-7-6-10(8)15(7)12/h1-6H,(H,13,14,16)
H11-1	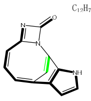 $C_{12}H_7N_3O$	3.3604	106.0349	-0.6828	63.3373	170.2006	InChI=1S/C12H7N3O/c16-12-14-10-3-1-2-7-6-9(15(10)12)11-8(7)4-5-13-11/h1-6,13H/b3-1-,7-2+
x6-37	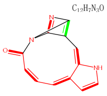 $C_{12}H_7N_3O$	7.5678	63.9245	-7.3201	87.0127	170.6488	InChI=1S/C12H7N3O/c16-10-3-1-2-7-4-5-13-9(7)6-8-11-14-12(8)15(10)11/h1-6,13H/b3-1-,7-2-,9-6+
G-20	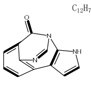 $C_{12}H_7N_3O$	3.3819	31.5376	-1.0286	122.7399	170.708	InChI=1S/C12H7N3O/c16-12-9-3-1-2-7-8-4-5-13-11(8)15(12)6-14-10(7)9/h1-6,13H
q1-48	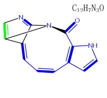 $C_{12}H_7N_3O$	4.7885	58.1365	-4.4497	107.7514	171.0032	InChI=1S/C12H7N3O/c16-12-10-7(4-5-13-10)2-1-3-8-9-6-14-11(8)15(9)12/h1-6,13H/b2-1-,8-3-
q1-49	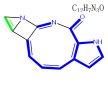 $C_{12}H_7N_3O$	4.7889	58.1364	-4.4501	107.7516	171.0032	InChI=1S/C12H7N3O/c16-12-10-7(4-5-13-10)2-1-3-8-9-6-15(9)11(8)14-12/h1-6,13H/b2-1-,8-3-,14-11+
F-51	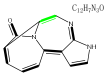 $C_{12}H_7N_3O$	4.5821	67.5758	-1.5187	93.8878	171.0266	InChI=1S/C12H7N3O/c16-12-8-2-1-3-9-7-4-5-13-11(7)14-6-10(8)15(9)12/h1-6H,(H,13,14)

H-53	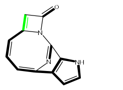 $C_{12}H_7N_3O$	2.5347	102.4615	-0.46	68.7926	171.1681	InChI=1S/C12H7N3O/c16-10-6-7-2-1-3-9-8-4-5-13-11(8)12(14-9)15(7)10/h1-6,13H/b2-1-,9-3-
k-42	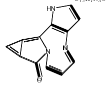 $C_{12}H_7N_3O$	12.1389	131.6509	-12.0904	33.2123	171.6974	InChI=1S/C12H7N3O/c16-12-8-6-7(8)11-10-9(2-4-14-10)13-3-1-5-15(11)12/h1-6,14H/b5-1-,13-3+
B-43	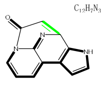 $C_{12}H_7N_3O$	2.4270	26.1226	-0.6467	134.6101	172.0471	InChI=1S/C12H7N3O/c16-10-6-9-11-7(3-4-13-11)8-2-1-5-15(10)12(8)14-9/h1-6,13H
r-16	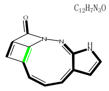 $C_{12}H_7N_3O$	6.1125	176.8878	-2.1651	-9.8995	172.1875	InChI=1S/C12H7N3O/c16-12-9-6-8-3-1-2-7-4-5-13-11(7)14-15(12)10(8)9/h1-6H,(H,13,14)/b3-1-,7-2-
A2-12	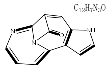 $C_{12}H_7N_3O$	4.6544	67.2507	-1.6348	94.9893	172.7055	InChI=1S/C12H7N3O/c16-12-8-6-9-7(3-5-13-9)10-2-1-4-14-11(8)15(10)12/h1-6,13H
G-44	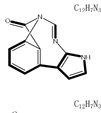 $C_{12}H_7N_3O$	3.3196	93.4663	-1.0526	72.9197	173.1638	InChI=1S/C12H7N3O/c16-12-10-7-2-1-3-9(10)15(12)6-14-11-8(7)4-5-13-11/h1-6,13H/b14-6+
B-42	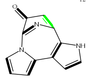 $C_{12}H_7N_3O$	1.9532	32.2233	-1.1398	125.9839	173.3647	InChI=1S/C12H7N3O/c16-10-6-8-11-7(3-4-13-11)9-2-1-5-15(9)12(10)14-8/h1-6,13H
F-68	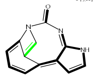 $C_{12}H_7N_3O$	3.8858	90.6198	-0.8942	70.9244	173.4242	InChI=1S/C12H7N3O/c16-12-14-11-8(4-5-13-11)7-2-1-3-10-9(7)6-15(10)12/h1-6H,(H,13,14,16)/b8-7-
I-89	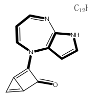 $C_{12}H_7N_3O$	15.1994	123.8905	-14.1899	31.8556	173.8152	InChI=1S/C12H7N3O/c16-11-8-6-7(8)10(11)15-5-1-3-13-12-9(15)2-4-14-12/h1-6,14H
o-89	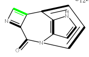 $C_{12}H_7N_3O$	1.6097	81.7025	-1.1105	88.8545	173.8596	InChI=1S/C12H7N3O/c16-12-11-8(6-14-11)7-2-1-5-15(12)9-3-4-13-10(7)9/h1-6,13H
H1-9	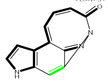 $C_{12}H_7N_3O$	3.8199	93.7507	-0.8348	63.9008	174.5586	InChI=1S/C12H7N3O/c16-11-3-1-2-8-7-4-5-13-9(7)6-10-12(8)14-15(10)11/h1-6,13H/b3-1-,8-2-
s11-5	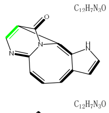 $C_{12}H_7N_3O$	5.5537	71.9083	-2.7465	88.19	174.6283	InChI=1S/C12H7N3O/c16-12-8-6-14-9-3-1-2-7-4-5-13-10(7)11(8)15(9)12/h1-6,13H/b2-1-,3-1-,7-2-,9-3-,11-10+
r-54	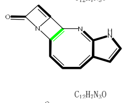 $C_{12}H_7N_3O$	4.3989	108.2	-4.4905	66.513	175.1858	InChI=1S/C12H7N3O/c16-10-6-9-11-8(15(9)10)3-1-2-7-4-5-13-12(7)14-11/h1-6H,(H,13,14)
H-162	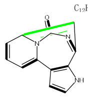 $C_{12}H_7N_3O$	3.9607	29.7555	-1.1196	129.6123	175.4641	InChI=1S/C12H7N3O/c16-12-14-9-6-7-2-1-3-10(15(7)12)8-4-5-13-11(8)9/h1-6,13H
s12-32	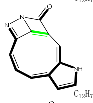 $C_{12}H_7N_3O$	4.5328	110.6982	-4.4613	57.2459	176.0986	InChI=1S/C12H7N3O/c16-12-8-6-10-7(4-5-13-10)2-1-3-9-11(8)15(12)14-9/h1-6,13H/b3-1-,7-2-,10-6+
H11-51	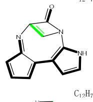 $C_{12}H_7N_3O$	4.7254	125.1139	-1.5559	39.5355	176.3135	InChI=1S/C12H7N3O/c16-12-10-6-15(12)11-8(4-5-13-11)7-2-1-3-9(7)14-10/h1-6,13H/b14-9-
p1-36	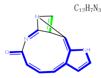 $C_{12}H_7N_3O$	6.1545	75.2028	-6.9142	70.9122	176.6051	InChI=1S/C12H7N3O/c16-9-3-1-2-7-4-5-13-10(7)11-8-6-15(11)12(8)14-9/h1-6,13H/b3-1-,7-2-,11-10-,14-12+
q11-4	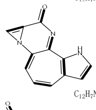 $C_{12}H_7N_3O$	25.5681	141.1312	-17.3474	10.1934	176.7601	InChI=1S/C12H7N3O/c16-12-9-6-15(9)8-3-1-2-7-4-5-13-10(7)11(8)14-12/h1-6,13H
p-10	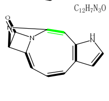 $C_{12}H_7N_3O$	6.1297	87.3151	-3.7965	82.3765	176.7952	InChI=1S/C12H7N3O/c16-12-11-9-3-1-2-7-4-5-13-8(7)6-10(14-11)15(9)12/h1-6,13H/b2-1-,3-1-,7-2-,8-6+,9-3-,10-6-

r-9	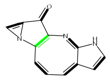 $C_{12}H_7N_3O$	19.2943	146.6623	-14.997	13.7703	177.83	InChI=1S/C12H7N3O/c16-11-9-6-15(9)8-3-1-2-7-4-5-13-12(7)14-10(8)11/h1-6H,(H,13,14)
F-30	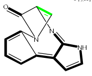 $C_{12}H_7N_3O$	4.8708	125.1208	-1.5796	39.6364	177.8631	InChI=1S/C12H7N3O/c16-12-9-6-15(12)10-3-1-2-7(10)8-4-5-13-11(8)14-9/h1-6H,(H,13,14)/b8-7-
q11-18	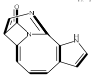 $C_{12}H_7N_3O$	5.7529	74.0277	-2.6645	89.6845	178.7669	InChI=1S/C12H7N3O/c16-12-8-6-14-11-10-7(4-5-13-10)2-1-3-9(8)15(11)12/h1-6,13H/b2-1-,3-1-,7-2-,9-3-,11-10+
H1-84	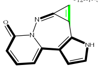 $C_{12}H_7N_3O$	19.8453	127.6083	-15.565	32.3122	178.7784	InChI=1S/C12H7N3O/c16-11-3-1-2-10-7-4-5-13-12(7)8-6-9(8)14-15(10)11/h1-6,13H
q-42	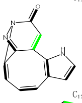 $C_{12}H_7N_3O$	5.1943	72.2889	-3.0381	88.3295	178.9836	InChI=1S/C12H7N3O/c16-10-6-8-11-7(4-5-13-11)2-1-3-9-12(8)14-15(9)10/h1-6,13H/b2-1-,9-3+
H1-27	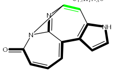 $C_{12}H_7N_3O$	3.625	54.8508	-1.9648	117.2584	179.0005	InChI=1S/C12H7N3O/c16-11-3-1-2-8-7-4-5-13-9(7)6-10-14-12(8)15(10)11/h1-6,13H
B-180	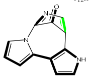 $C_{12}H_7N_3O$	2.3329	31.1598	-1.4478	130.8328	179.1354	InChI=1S/C12H7N3O/c16-11-8-6-14-12(11)15-5-1-2-9(15)7-3-4-13-10(7)8/h1-6,13H
D-8	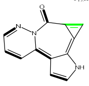 $C_{12}H_7N_3O$	24.7489	122.6888	-16.4964	38.7076	179.1979	InChI=1S/C12H7N3O/c16-12-9-6-8(9)11-7(3-5-13-11)10-2-1-4-14-15(10)12/h1-6,13H
s12-48	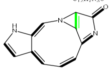 $C_{12}H_7N_3O$	10.3475	135.2974	-6.1342	16.8237	179.6105	InChI=1S/C12H7N3O/c16-12-11-10-8(14-12)3-1-2-7-4-5-13-9(7)6-15(10)11/h1-6,13H/b3-1-,7-2-,9-6+
x9-43	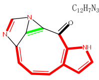 $C_{12}H_7N_3O$	9.7586	139.6265	-7.2259	16.0495	179.882	InChI=1S/C12H7N3O/c16-12-9-7(4-5-13-9)2-1-3-8-10-11(12)15(10)6-14-8/h1-6,13H/b2-1-,8-3+
X23-8	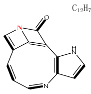 $C_{12}H_7N_3O$	4.4837	184.9719	-1.8971	-4.04	179.9794	InChI=1S/C12H7N3O/c16-12-9-10-8(3-5-14-10)13-4-1-2-7-6-15(12)11(7)9/h1-6,14H/b2-1-,13-4-
X23-17	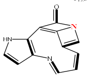 $C_{12}H_7N_3O$	4.4829	184.9713	-1.8969	-4.04	179.9795	InChI=1S/C12H7N3O/c16-12-9-10-8(3-5-14-10)13-4-1-2-7-6-15(12)11(7)9/h1-6,14H/b2-1-,13-4+
B-119	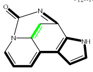 $C_{12}H_7N_3O$	2.0791	24.3021	-0.8850	143.6051	180.0002	InChI=1S/C12H7N3O/c16-12-14-9-6-10-7(2-1-5-15(10)12)8-3-4-13-11(8)9/h1-6,13H
G-71	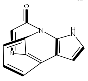 $C_{12}H_7N_3O$	3.5874	32.3843	-1.3496	130.1585	180.2907	InChI=1S/C12H7N3O/c16-10-6-14-11-7-2-1-3-9(11)15(10)12-8(7)4-5-13-12/h1-6,13H
B-77	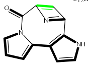 $C_{12}H_7N_3O$	6.5449	76.0963	-2.2443	85.0898	180.3398	InChI=1S/C12H7N3O/c16-12-9-6-8(14-9)11-7(3-4-13-11)10-2-1-5-15(10)12/h1-6,13H
F-21	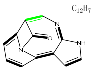 $C_{12}H_7N_3O$	5.0303	76.9726	-1.3805	95.4579	180.3607	InChI=1S/C12H7N3O/c16-12-8-6-14-11-7(4-5-13-11)9-2-1-3-10(8)15(9)12/h1-6H,(H,13,14)
X22-33	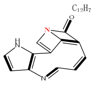 $C_{12}H_7N_3O$	4.4933	184.9202	-1.8946	-2.98	180.5989	InChI=1S/C12H7N3O/c16-12-7-2-1-4-13-9-3-5-14-10(9)8-6-15(12)11(7)8/h1-6,14H/b2-1-,13-4+
B-198	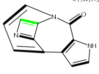 $C_{12}H_7N_3O$	2.1032	86.7524	-1.0825	87.1370	180.6371	InChI=1S/C12H7N3O/c16-12-11-8(3-4-13-11)7-2-1-5-15(12)9-6-14-10(7)9/h1-6,13H
H11-69	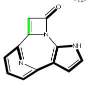 $C_{12}H_7N_3O$	2.4242	89.6617	-0.84	82.6258	180.6661	InChI=1S/C12H7N3O/c16-11-6-10-9-3-1-2-8(14-9)7-4-5-13-12(7)15(10)11/h1-6,13H

H11-73	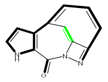 $C_{12}H_7N_3O$	3.2291	82.5713	-0.6564	81.7596	180.8385	InChI=1S/C12H7N3O/c16-12-11-8(4-5-13-11)7-2-1-3-9-10(6-7)15(12)14-9/h1-6,13H
k1-23	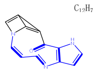 $C_{12}H_7N_3O$	12.6307	124.8361	-10.6346	32.2657	181.0101	InChI=1S/C12H7N3O/c16-12-9-7-6-15(11(7)9)5-1-3-13-8-2-4-14-10(8)12/h1-6,14H/b5-1-,13-3+
H1-135	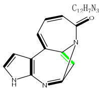 $C_{12}H_7N_3O$	3.1397	54.6767	-1.8121	117.6126	181.9984	InChI=1S/C12H7N3O/c16-11-3-1-2-7-8-4-5-13-12(8)14-10-6-9(7)15(10)11/h1-6,13H
k-14	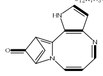	4.6813	59.9744	-5.7698	111.0014	182.4278	InChI=1S/C12H7N3O/c16-12-7-6-9(12)15-5-1-3-13-8-2-4-14-10(8)11(7)15/h1-6,14H/b5-1-,13-3+
B-129	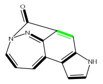 $C_{12}H_7N_3O$	2.7518	47.0601	-0.3761	117.2884	182.5027	InChI=1S/C12H7N3O/c16-12-9-6-10-7(3-4-13-10)8-2-1-5-15(12)14-11(8)9/h1-6,13H
o-217	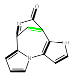 $C_{12}H_7N_3O$	2.4967	32.8873	-1.4343	131.4104	182.6763	InChI=1S/C12H7N3O/c16-12-7-6-8(14-12)9-2-1-5-15(9)10-3-4-13-11(7)10/h1-6,13H
o-222	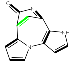 $C_{12}H_7N_3O$	2.4949	32.8575	-1.4083	131.8529	182.7319	InChI=1S/C12H7N3O/c16-12-7-6-8(14-12)11-10(3-4-13-11)15-5-1-2-9(7)15/h1-6,13H
H1-57	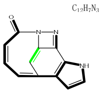 $C_{12}H_7N_3O$	4.2946	103.1244	-0.6537	61.08	183.0038	InChI=1S/C12H7N3O/c16-10-3-1-2-7-6-9-12(14-15(9)10)11-8(7)4-5-13-11/h1-6,13H/b3-1-,7-2+
D-24	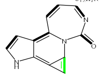 $C_{12}H_7N_3O$	16.2347	140.6010	-13.0611	28.5334	183.1472	InChI=1S/C12H7N3O/c16-12-14-4-1-2-9-7-3-5-13-11(7)8-6-10(8)15(9)12/h1-6,13H
H1-102	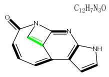 $C_{12}H_7N_3O$	4.5341	76.2478	-2.3341	92.3412	183.1818	InChI=1S/C12H7N3O/c16-10-3-1-2-7-8-4-5-13-11(8)14-12-9(7)6-15(10)12/h1-6,13H/b3-1-,7-2-
H-76	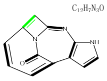 $C_{12}H_7N_3O$	4.9167	69.1839	-1.8255	106.5246	183.285	InChI=1S/C12H7N3O/c16-12-9-3-1-2-7-6-10(15(7)12)14-11-8(9)4-5-13-11/h1-6H,(H,13,14)
H-101	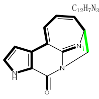 $C_{12}H_7N_3O$	5.8232	39.8649	-0.801	117.0571	183.3269	InChI=1S/C12H7N3O/c16-12-10-8(4-5-13-10)9-3-1-2-7-6-15(12)11(9)14-7/h1-6,13H
D-4	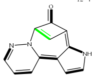 $C_{12}H_7N_3O$	3.5715	49.9432	-2.3423	110.6633	183.3468	InChI=1S/C12H7N3O/c16-12-8-6-10(12)15-9(2-1-4-14-15)7-3-5-13-11(7)8/h1-6,13H
s11-17	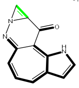 $C_{12}H_7N_3O$	9.6254	141.8565	-7.1825	15.1173	183.8615	InChI=1S/C12H7N3O/c16-12-9-6-15(9)14-8-3-1-2-7-4-5-13-11(7)10(8)12/h1-6,13H
B-87	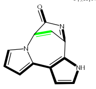 $C_{12}H_7N_3O$	2.4120	32.0922	-1.4678	133.3005	184.0518	InChI=1S/C12H7N3O/c16-12-10-6-8(14-12)11-7(3-4-13-11)9-2-1-5-15(9)10/h1-6,13H
B-39	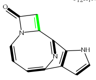 $C_{12}H_7N_3O$	1.9130	94.1783	-0.5022	88.0528	184.2918	InChI=1S/C12H7N3O/c16-10-6-9-12-11-7(3-4-13-11)8(14-12)2-1-5-15(9)10/h1-6,13H/b5-1-,8-2-
r-46	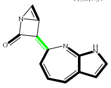 $C_{12}H_7N_3O$	21.4879	155.8057	-15.9717	16.6769	184.3004	InChI=1S/C12H7N3O/c16-12-10(9-6-15(9)12)8-3-1-2-7-4-5-13-11(7)14-8/h1-6H,(H,13,14)/b10-8+
B-64	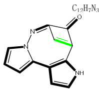 $C_{12}H_7N_3O$	2.7833	68.1035	-1.4902	101.9827	185.4752	InChI=1S/C12H7N3O/c16-12-8-6-9(12)14-15-5-1-2-10(15)7-3-4-13-11(7)8/h1-6,13H/b14-9+
r1-28	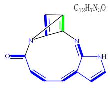 $C_{12}H_7N_3O$	8.1016	65.9566	-7.9122	98.8439	185.7817	InChI=1S/C12H7N3O/c16-10-3-1-2-7-4-5-13-12(7)14-11-8-6-9(11)15(8)10/h1-6H,(H,13,14)/b3-1-,7-2-

p1-23	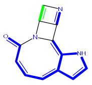 $C_{12}H_7N_3O$	7.0286	88.2227	-6.2578	92.9829	185.8994	InChI=1S/C12H7N3O/c16-9-3-1-2-7-4-5-13-10(7)12-11-8(6-14-11)15(9)12/h1-6,13H/b3-1-,7-2-,12-10+
r-51	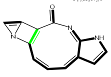 $C_{12}H_7N_3O$	18.071	152.1334	-14.4942	13.1802	185.9392	InChI=1S/C12H7N3O/c16-12-10-8(15-6-9(10)15)3-1-2-7-4-5-13-11(7)14-12/h1-6H,(H,13,14,16)
H11-81	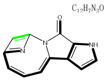 $C_{12}H_7N_3O$	2.3408	78.2946	-1.2903	100.4996	186.2417	InChI=1S/C12H7N3O/c16-12-11-8(4-5-13-11)9-3-1-2-7-6-10(14-7)15(9)12/h1-6,13H/b2-1-,9-3-
H1-55	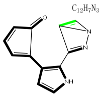 $C_{12}H_7N_3O$	18.4	149.9367	-14.4214	23.1417	186.7559	InChI=1S/C12H7N3O/c16-10-3-1-2-7(10)8-4-5-13-11(8)12-9-6-15(9)14-12/h1-6,13H
H11-8	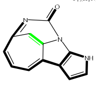 $C_{12}H_7N_3O$	4.6756	52.9127	-1.0674	108.569	186.8569	InChI=1S/C12H7N3O/c16-12-14-7-2-1-3-8-9-4-5-13-11(9)15(12)10(8)6-7/h1-6,13H
p-28	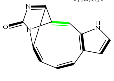 $C_{12}H_7N_3O$	4.7141	82.2546	-3.9506	100.0256	186.9254	InChI=1S/C12H7N3O/c16-12-14-11-9-3-1-2-7-4-5-13-8(7)6-10(11)15(9)12/h1-6,13H/b2-1-,9-3+,10-6-
q1-9	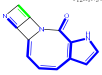 $C_{12}H_7N_3O$	7.1364	88.7328	-6.1786	91.9165	187.7356	InChI=1S/C12H7N3O/c16-12-10-7(4-5-13-10)2-1-3-8-11-9(6-14-11)15(8)12/h1-6,13H/b2-1-,8-3-
G1-87	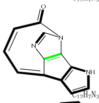 $C_{12}H_7N_3O$	5.2311	66.2549	-0.8107	104.058	187.8009	InChI=1S/C12H7N3O/c16-9-3-1-2-7-8-4-5-13-10(8)12-11(7)14-6-15(9)12/h1-6,13H/b3-1-,7-2-
H11-123	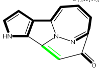 $C_{12}H_7N_3O$	6.3211	64.9993	0.3543	90.322	187.8322	InChI=1S/C12H7N3O/c16-11-6-10-12-7(4-5-13-12)9-3-1-2-8(11)14-15(9)10/h1-6,13H
G-42	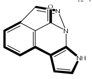 $C_{12}H_7N_3O$	2.5515	90.9628	-0.8953	79.2273	187.9293	InChI=1S/C12H7N3O/c16-12-10-7-2-1-3-8(10)9-4-5-13-11(9)15(12)14-6-7/h1-6,13H
q1-54	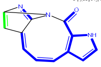 $C_{12}H_7N_3O$	5.2484	53.8557	-4.6202	126.9986	187.9864	InChI=1S/C12H7N3O/c16-12-10-7(4-5-13-10)2-1-3-8-6-9-14-11(8)15(9)12/h1-6,13H/b2-1-,8-3-
C-51	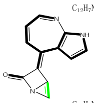 $C_{12}H_7N_3O$	21.5486	157.2139	-15.6416	16.7246	188.1383	InChI=1S/C12H7N3O/c16-12-10(9-6-15(9)12)7-2-1-4-13-11-8(7)3-5-14-11/h1-6,14H/b10-7+
X22-3	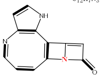 $C_{12}H_7N_3O$	4.0731	148.6576	-3.8749	39.8264	188.16	InChI=1S/C12H7N3O/c16-10-6-9-11-8(15(9)10)2-1-4-13-7-3-5-14-12(7)11/h1-6,14H/b2-1-,4-1-,8-2+,12-11-,13-4-,13-7-
s11-37	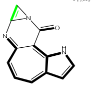 $C_{12}H_7N_3O$	29.1859	149.729	-18.1174	13.5391	188.2658	InChI=1S/C12H7N3O/c16-12-10-8(14-9-6-15(9)12)3-1-2-7-4-5-13-11(7)10/h1-6,13H
X22-2	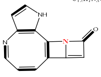 $C_{12}H_7N_3O$	4.0174	148.627	-3.8165	38.7633	188.3089	InChI=1S/C12H7N3O/c16-10-6-9-7-2-1-4-13-8-3-5-14-11(8)12(7)15(9)10/h1-6,14H/b2-1-,4-1-,7-2-,12-11-,13-4-,13-8-
o-75	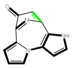 $C_{12}H_7N_3O$	2.1657	32.9716	-1.0209	139.214	188.867	InChI=1S/C12H7N3O/c16-10-6-7-11-9(3-4-13-11)15-5-1-2-8(15)12(10)14-7/h1-6,13H
r-4	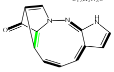 $C_{12}H_7N_3O$	5.0505	53.4778	-5.0751	117.6826	188.9603	InChI=1S/C12H7N3O/c16-11-9-6-15-10(11)8(9)3-1-2-7-4-5-13-12(7)14-15/h1-6H,(H,13,14)/b3-1-,7-2-
x4-40	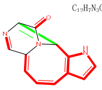 $C_{12}H_7N_3O$	5.3879	76.3699	-2.4702	94.9236	189.3458	InChI=1S/C12H7N3O/c16-12-10-11-9-7(4-5-13-9)2-1-3-8(6-14-10)15(11)12/h1-6,13H/b2-1-,3-1-,7-2?,8-3-,11-9+
X22-21	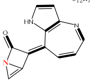 $C_{12}H_7N_3O$	21.7735	157.2358	-16.0054	19.3404	189.4612	InChI=1S/C12H7N3O/c16-12-10(9-6-15(9)12)7-2-1-4-13-8-3-5-14-11(7)8/h1-6,14H/b10-7-

q-49	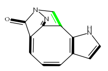	$C_{12}H_7N_3O$	3.167	56.3556	-2.3104	117.5566	189.7924	InChI=1S/C12H7N3O/c16-12-8-3-1-2-7-4-5-13-10(7)9-6-15(12)14-11(8)9/h1-6,13H/b2-1-,8-3+
o-82	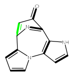	$C_{12}H_7N_3O$	2.1749	32.8485	-0.9936	139.812	189.7929	InChI=1S/C12H7N3O/c16-10-6-7-8-2-1-5-15(8)9-3-4-13-11(9)12(10)14-7/h1-6,13H
A-78	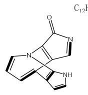	$C_{12}H_7N_3O$	3.4893	34.8422	-2.1022	124.3005	190.0713	InChI=1S/C12H7N3O/c16-12-10-9(6-14-12)7-2-1-5-15(10)11-8(7)3-4-13-11/h1-6,13H
q1-18	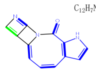	$C_{12}H_7N_3O$	6.364	90.4827	-5.7725	94.3089	190.1406	InChI=1S/C12H7N3O/c16-12-10-7(4-5-13-10)2-1-3-9-8-6-14-11(8)15(9)12/h1-6,13H/b2-1-,9-3-
o-86	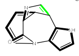	$C_{12}H_7N_3O$	5.7944	37.6499	-1.6408	130.1714	190.6841	InChI=1S/C12H7N3O/c16-12-11-7-2-1-5-15(12)9-3-4-13-10(9)8(7)6-14-11/h1-6,13H
A2-20	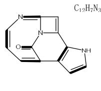	$C_{12}H_7N_3O$	3.2034	70.8554	-1.1244	115.3354	190.8621	InChI=1S/C12H7N3O/c16-12-8-2-1-4-13-10-6-9(15(10)12)11-7(8)3-5-14-11/h1-6,14H/b2-1-,4-1-,8-2-,13-4-,13-10-
x2-17	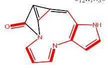	$C_{12}H_7N_3O$	19.3346	126.3221	-11.3978	47.475	191.0323	InChI=1S/C12H7N3O/c16-12-10-7-6-9-8(2-4-14-9)13-3-1-5-15(12)11(7)10/h1-6,14H/b5-1-,7-6-,13-3+
I-13	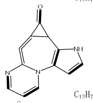	$C_{12}H_7N_3O$	11.9386	156.9465	-10.5928	11.4469	191.3651	InChI=1S/C12H7N3O/c16-12-7-6-9-13-3-1-5-15(9)8-2-4-14-11(8)10(7)12/h1-5,10,14H
o-51	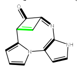	$C_{12}H_7N_3O$	3.109	69.592	-1.3498	105.2136	191.5327	InChI=1S/C12H7N3O/c16-11-7-6-8(11)14-12-10(3-4-13-12)15-5-1-2-9(7)15/h1-6,13H/b14-8+
o-180	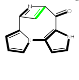	$C_{12}H_7N_3O$	3.4364	70.5752	-1.2293	102.2899	191.7114	InChI=1S/C12H7N3O/c16-12-8-6-7(14-8)9-2-1-5-15(9)10-3-4-13-11(10)12/h1-6,13H
H1-46	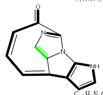	$C_{12}H_7N_3O$	4.7226	59.1239	-0.9962	113.0796	191.7272	InChI=1S/C12H7N3O/c16-10-3-1-2-7-8-4-5-13-11(8)15-9(7)6-14-12(10)15/h1-6,13H/b3-1-,7-2-
E-36	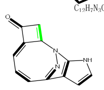	$C_{12}H_7N_3O$	8.3379	135.9304	0.1856	19.6237	191.7293	InChI=1S/C12H7N3O/c16-11-6-10-8(11)2-1-3-9-7-4-5-13-12(7)15(10)14-9/h1-6,13H/b3-1-,8-2+
A2-41	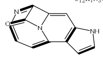	$C_{12}H_7N_3O$	3.6878	77.7517	-0.9857	103.5818	191.8679	InChI=1S/C12H7N3O/c16-12-11-10-6-8-7(3-5-13-8)9(15(10)12)2-1-4-14-11/h1-6,13H/b2-1-,4-1-,9-2-,14-4-,14-11-
x6-40	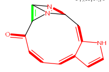	$C_{12}H_7N_3O$	15.7612	123.1308	-10.7783	37.906	191.9173	InChI=1S/C12H7N3O/c16-9-3-1-2-7-4-5-13-8(7)6-10-14-12-11(9)15(10)12/h1-6,13H/b3-1-,7-2-,8-6+
H11-67	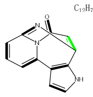	$C_{12}H_7N_3O$	4.3531	29.3777	-1.1756	145.7394	192.017	InChI=1S/C12H7N3O/c16-11-6-8-12-7(4-5-13-12)9-2-1-3-10(14-8)15(9)11/h1-6,13H
I-19	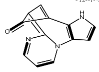	$C_{12}H_7N_3O$	4.471	49.9794	-2.7877	117.9557	192.5579	InChI=1S/C12H7N3O/c16-11-7-6-8(11)12-14-3-1-5-15(12)9-2-4-13-10(7)9/h1-6,13H
B-21	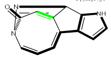	$C_{12}H_7N_3O$	6.3858	52.7413	-1.4743	97.3776	192.6615	InChI=1S/C12H7N3O/c16-10-6-9-7-2-1-5-15(10)14-12(9)11-8(7)3-4-13-11/h1-6,13H/b5-1-,7-2-
B-66	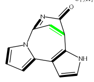	$C_{12}H_7N_3O$	2.3806	31.5196	-1.4024	143.2325	192.6658	InChI=1S/C12H7N3O/c16-12-8-6-10(14-12)15-5-1-2-9(15)7-3-4-13-11(7)8/h1-6,13H
x7-16	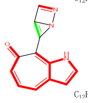	$C_{12}H_7N_3O$	15.9834	152.0666	-10.9336	16.8512	193.1598	InChI=1S/C12H7N3O/c16-9-3-1-2-7-4-5-13-11(7)10(9)12-8-6-14-15(8)12/h1-6,13H

H-106	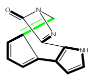 $C_{12}H_7N_3O$	7.9994	150.2604	-1.8262	17.9143	193.2735	InChI=1S/C12H7N3O/c16-12-11-10-9(4-5-13-10)8-3-1-2-7(8)6-15(12)14-11/h1-6,13H/b7-6+
B-117	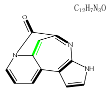 $C_{12}H_7N_3O$	5.1653	34.0296	-0.9789	133.5773	193.2756	InChI=1S/C12H7N3O/c16-12-9-6-10-7(2-1-5-15(10)12)8-3-4-13-11(8)14-9/h1-6,13H
q-22	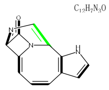 $C_{12}H_7N_3O$	5.6087	79.3971	-2.3508	96.5773	193.5204	InChI=1S/C12H7N3O/c16-12-11-8-3-1-2-7-4-5-13-10(7)9(6-14-11)15(8)12/h1-6,13H/b2-1-,3-1-,7-2-,8-3-,10-9+
A2-56	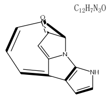 $C_{12}H_7N_3O$	4.5262	55.1997	-1.2475	117.7667	194.1001	InChI=1S/C12H7N3O/c16-10-6-9-7-2-1-4-13-12(10)15(9)11-8(7)3-5-14-11/h1-6,14H/b4-1-,7-2-,13-12+
r-15	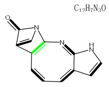 $C_{12}H_7N_3O$	4.1907	85.3634	-4.4024	106.0149	194.1454	InChI=1S/C12H7N3O/c16-12-9-6-15(12)11-8(9)3-1-2-7-4-5-13-10(7)14-11/h1-6H,(H,13,14)
r1-7	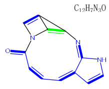 $C_{12}H_7N_3O$	13.2254	117.6991	-10.7014	61.2287	194.1483	InChI=1S/C12H7N3O/c16-9-3-1-2-7-4-5-13-12(7)14-10-8-6-15(9)11(8)10/h1-6H,(H,13,14)/b3-1-,7-2-
q11-65	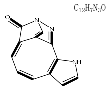 $C_{12}H_7N_3O$	3.5248	52.533	-2.7291	119.3861	194.2395	InChI=1S/C12H7N3O/c16-12-8-3-1-2-7-4-5-13-10(7)11-9(8)6-15(12)14-11/h1-6,13H/b2-1-,8-3+
o-36	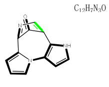 $C_{12}H_7N_3O$	2.4785	31.8613	-1.2981	144.4271	194.3986	InChI=1S/C12H7N3O/c16-12-7-6-14-11(12)8-2-1-5-15(8)9-3-4-13-10(7)9/h1-6,13H
o-126	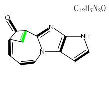 $C_{12}H_7N_3O$	2.3875	74.6673	-1.7146	108.4398	194.4732	InChI=1S/C12H7N3O/c16-10-7-2-1-5-15-9-3-4-13-11(9)14-12(15)8(10)6-7/h1-6,13H/b5-1-,7-2+
s11-39	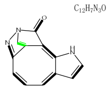 $C_{12}H_7N_3O$	3.5121	53.2085	-2.7216	119.4333	194.6104	InChI=1S/C12H7N3O/c16-12-10-8-6-15(12)14-9(8)3-1-2-7-4-5-13-11(7)10/h1-6,13H/b3-1-,7-2-,11-10-
G1-68	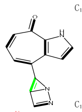 $C_{12}H_7N_3O$	15.3827	151.5405	-10.9756	18.1523	195.3412	InChI=1S/C12H7N3O/c16-10-3-1-2-8(7-4-5-13-11(7)10)12-9-6-14-15(9)12/h1-6,13H
x9-25	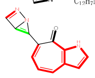 $C_{12}H_7N_3O$	15.9444	153.012	-10.9392	16.551	196.0665	InChI=1S/C12H7N3O/c16-12-8(11-9-6-14-15(9)11)3-1-2-7-4-5-13-10(7)12/h1-6,13H
H-23	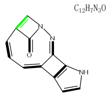 $C_{12}H_7N_3O$	4.6117	137.4506	-1.8718	55.6449	196.1947	InChI=1S/C12H7N3O/c16-12-7-2-1-3-8-9-4-5-13-10(9)11(8)14-15(12)6-7/h1-6,13H/b2-1-,8-3-,14-11+
B-52	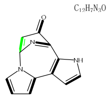 $C_{12}H_7N_3O$	2.0557	32.9466	-0.9800	147.5796	196.2259	InChI=1S/C12H7N3O/c16-9-6-10-14-12(9)11-7(3-4-13-11)8-2-1-5-15(8)10/h1-6,13H
G-63	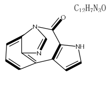 $C_{12}H_7N_3O$	4.759	39.4986	-1.1245	131.5732	196.3838	InChI=1S/C12H7N3O/c16-12-11-8(4-5-13-11)7-2-1-3-9-10(7)14-6-15(9)12/h1-6,13H
B-115	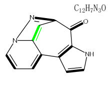 $C_{12}H_7N_3O$	5.3757	32.2214	-0.8095	132.4213	196.4807	InChI=1S/C12H7N3O/c16-12-9-6-10-7(2-1-5-15(10)14-9)8-3-4-13-11(8)12/h1-6,13H
s12-30	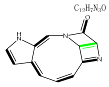 $C_{12}H_7N_3O$	7.4878	87.0626	-5.6032	102.1504	196.6345	InChI=1S/C12H7N3O/c16-12-10-11-8(14-10)3-1-2-7-4-5-13-9(7)6-15(11)12/h1-6,13H/b3-1-,7-2-,9-6+
H1-125	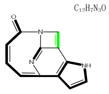 $C_{12}H_7N_3O$	4.1118	96.1394	-0.2098	90.2526	196.9734	InChI=1S/C12H7N3O/c16-10-3-1-2-9-7-4-5-13-11(7)8-6-15(10)12(8)14-9/h1-6,13H/b3-1-,9-2-
o-202	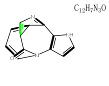 $C_{12}H_7N_3O$	5.3947	40.399	-1.4307	137.4653	197.1262	InChI=1S/C12H7N3O/c16-12-8-6-14-10-7(8)2-1-5-15(12)9-3-4-13-11(9)10/h1-6,13H

G-76	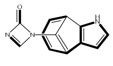	$C_{12}H_7N_3O$	3.8322	87.1896	-2.5874	105.2713	197.5654	InChI=1S/C12H7N3O/c16-12-14-6-15(12)11-8-2-1-3-9(11)10-7(8)4-5-13-10/h1-6,13H
A-80	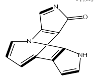	$C_{12}H_7N_3O$	3.5602	35.8233	-2.1671	132.2546	197.5835	InChI=1S/C12H7N3O/c16-12-10-7-2-1-5-15(9(10)6-14-12)11-8(7)3-4-13-11/h1-6,13H
r-32	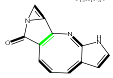	$C_{12}H_7N_3O$	22.0594	167.2708	-14.9091	16.3014	197.6605	InChI=1S/C12H7N3O/c16-12-8-3-1-2-7-4-5-13-11(7)14-10(8)9-6-15(9)12/h1-6H,(H,13,14)
B-75	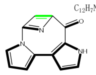	$C_{12}H_7N_3O$	2.6207	67.9332	-1.1617	113.2062	197.6812	InChI=1S/C12H7N3O/c16-12-8-6-10(14-8)15-5-1-2-9(15)7-3-4-13-11(7)12/h1-6,13H
X-20	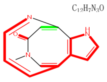	$C_{13}H_{11}N_3O$	3.6396	39.3416	0.3498	138.6864	198.0779	InChI=1S/C13H11N3O/c1-16-8-9-3-2-5-14-11(7-12(16)17)13-10(9)4-6-15-13/h2-8,15H,1H3/b3-2-,9-8-,11-7+,14-5-
B-251	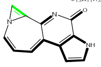	$C_{12}H_7N_3O$	19.5566	152.7600	-11.6943	13.4288	198.3912	InChI=1S/C12H7N3O/c16-12-11-8(3-4-13-11)7-2-1-5-15-6-9(15)10(7)14-12/h1-6,13H
x2-4	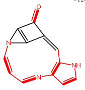	$C_{12}H_7N_3O$	20.5461	140.7699	-11.5038	28.2621	198.5072	InChI=1S/C12H7N3O/c16-12-7-6-9-8(2-4-14-9)13-3-1-5-15-10(7)11(12)15/h1-6,14H/b5-1-,7-6+,13-3+
X23-6	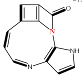	$C_{12}H_7N_3O$	6.7308	87.8405	-5.1731	110.7353	198.6276	InChI=1S/C12H7N3O/c16-12-8-6-7-2-1-4-13-9-3-5-14-11(9)15(12)10(7)8/h1-6,14H/b2-1-,13-4-
q-67	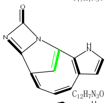	$C_{12}H_7N_3O$	5.5478	117.6032	-0.6777	71.8415	198.7185	InChI=1S/C12H7N3O/c16-12-14-11-8-3-1-2-7-4-5-13-10(7)9(6-8)15(11)12/h1-6,13H/b2-1-,3-1-,7-2-,8-3+,10-9-
G-68	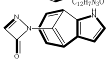	$C_{12}H_7N_3O$	3.6332	85.1245	-2.6736	105.2229	198.9734	InChI=1S/C12H7N3O/c16-10-6-14-15(10)12-8-2-1-3-9(12)11-7(8)4-5-13-11/h1-6,13H
H11-87	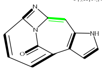	$C_{12}H_7N_3O$	5.2578	71.6006	-1.8279	122.284	199.4523	InChI=1S/C12H7N3O/c16-12-8-2-1-3-10-14-11(15(10)12)6-9-7(8)4-5-13-9/h1-6,13H
r-2	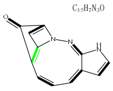	$C_{12}H_7N_3O$	5.3419	51.5049	-4.9031	131.0908	199.4916	InChI=1S/C12H7N3O/c16-11-8-3-1-2-7-4-5-13-12(7)14-15-9(8)6-10(11)15/h1-6H,(H,13,14)/b3-1-,7-2-
F-6	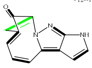	$C_{12}H_7N_3O$	6.0618	85.816	-2.0488	91.8315	199.955	InChI=1S/C12H7N3O/c16-11-7-2-1-3-9-8-4-5-13-12(8)14-15(9)10(11)6-7/h1-6H,(H,13,14)/b3-1-,7-2-
o-144	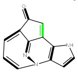	$C_{12}H_7N_3O$	8.3812	62.9362	0.3676	103.6842	200.059	InChI=1S/C12H7N3O/c16-10-6-8-11-7(10)2-1-5-15(14-11)9-3-4-13-12(8)9/h1-6,13H
G1-42	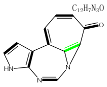	$C_{12}H_7N_3O$	12.7837	153.9825	-6.5026	20.2053	200.0878	InChI=1S/C12H7N3O/c16-9-3-1-2-7-8-4-5-13-12(8)14-6-15-10(7)11(9)15/h1-6,13H
B-62	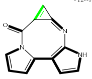	$C_{12}H_7N_3O$	24.8968	131.2636	-16.4417	40.6715	200.2822	InChI=1S/C12H7N3O/c16-12-8-6-9(8)14-11-7(3-4-13-11)10-2-1-5-15(10)12/h1-6,13H/b14-9-
s12-42	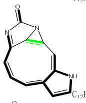	$C_{12}H_7N_3O$	13.8088	157.3361	-8.7276	16.0777	200.4169	InChI=1S/C12H7N3O/c16-12-14-8-3-1-2-7-4-5-13-9(7)6-10-11(8)15(10)12/h1-6,13H/b3-1-,7-2-,9-6+
A-165	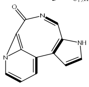	$C_{12}H_7N_3O$	11.1724	155.4483	-6.3101	16.6695	200.6597	InChI=1S/C12H7N3O/c16-12-11-10-8(2-1-5-15(10)11)7-3-4-13-9(7)6-14-12/h1-6,13H/b14-6-
G-45	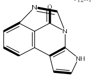	$C_{12}H_7N_3O$	2.975	85.2699	-0.8383	102.7676	201.1512	InChI=1S/C12H7N3O/c16-12-10-7-2-1-3-9(10)14-6-15(12)11-8(7)4-5-13-11/h1-6,13H

H1-28	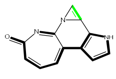 $C_{12}H_7N_3O$	26.0752	157.6419	-17.051	14.5391	201.2877	InChI=1S/C12H7N3O/c16-10-3-1-2-8-7-4-5-13-11(7)9-6-15(9)12(8)14-10/h1-6,13H
r1-31	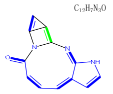 $C_{12}H_7N_3O$	13.9961	135.3994	-13.918	53.0801	201.6113	InChI=1S/C12H7N3O/c16-10-3-1-2-7-4-5-13-11(7)14-12-8-6-9(8)15(10)12/h1-6H,(H,13,14)/b3-1-,7-2-
H11-141	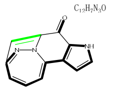 $C_{12}H_7N_3O$	4.9332	42.611	-0.7141	126.1791	201.8289	InChI=1S/C12H7N3O/c16-12-10-6-7-2-1-3-9(15(10)14-7)8-4-5-13-11(8)12/h1-6,13H
H-159	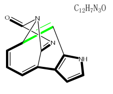 $C_{12}H_7N_3O$	2.8113	88.3997	-1.4149	106.5172	202.3606	InChI=1S/C12H7N3O/c16-12-14-11-9-3-1-2-7(15(11)12)6-10-8(9)4-5-13-10/h1-6,13H
H-153	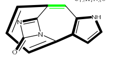 $C_{12}H_7N_3O$	2.7674	88.9402	-1.415	106.2531	202.5799	InChI=1S/C12H7N3O/c16-12-14-11-7-2-1-3-10(15(11)12)8-4-5-13-9(8)6-7/h1-6,13H
H1-2	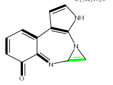 $C_{12}H_7N_3O$	32.5001	151.524	-18.9256	18.3715	202.7886	InChI=1S/C12H7N3O/c16-9-3-1-2-7-8-4-5-13-12(8)15-6-10(15)14-11(7)9/h1-6,13H
H1-1	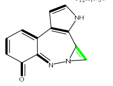 $C_{12}H_7N_3O$	10.1248	157.0471	-6.7654	15.6791	203.0717	InChI=1S/C12H7N3O/c16-10-3-1-2-7-8-4-5-13-11(8)9-6-15(9)14-12(7)10/h1-6,13H
A-200	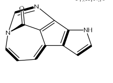 $C_{12}H_7N_3O$	4.3229	48.8463	-0.9987	122.7673	203.5869	InChI=1S/C12H7N3O/c16-12-9-7-2-1-5-15(12)6-14-11(9)10-8(7)3-4-13-10/h1-6,13H
p1-11	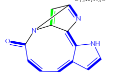 $C_{12}H_7N_3O$	5.2979	47.8191	-4.6813	145.9246	203.7847	InChI=1S/C12H7N3O/c16-10-3-1-2-7-4-5-13-11(7)12-8-6-9(14-12)15(8)10/h1-6,13H/b3-1-,7-2-,12-11-
H11-145	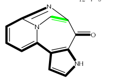 $C_{12}H_7N_3O$	5.8911	35.3454	-0.7056	136.0079	204.0675	InChI=1S/C12H7N3O/c16-12-8-6-15-9(2-1-3-10(15)14-8)7-4-5-13-11(7)12/h1-6,13H
q1-33	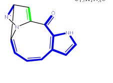 $C_{12}H_7N_3O$	5.254	56.2389	-4.8825	126.2473	204.1585	InChI=1S/C12H7N3O/c16-12-10-6-8-9(15(10)14-8)3-1-2-7-4-5-13-11(7)12/h1-6,13H/b2-1-,9-3-
B-236	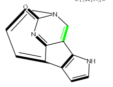 $C_{12}H_7N_3O$	5.9406	51.4285	-1.3711	114.7518	204.3059	InChI=1S/C12H7N3O/c16-12-14-11-7-2-1-5-15(12)6-9(11)10-8(7)3-4-13-10/h1-6,13H/b5-1-,7-2-
s11-42	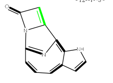 $C_{12}H_7N_3O$	9.918	158.9866	0.5146	15.8662	204.4707	InChI=1S/C12H7N3O/c16-10-6-8-12-11-7(4-5-13-11)2-1-3-9(14-12)15(8)10/h1-6,13H/b2-1-,3-1-,7-2-,9-3+,12-11+
A-60	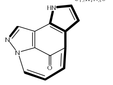 $C_{12}H_7N_3O$	5.1618	45.5747	-0.2571	131.3653	204.7129	InChI=1S/C12H7N3O/c16-12-8-2-1-5-15-11(12)9(6-14-15)10-7(8)3-4-13-10/h1-6,13H
x5-10	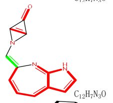 $C_{12}H_7N_3O$	51.1042	154.7884	-28.9274	11.0764	204.7688	InChI=1S/C12H7N3O/c16-11-9-10(11)15(9)6-8-3-1-2-7-4-5-13-12(7)14-8/h1-6H,(H,13,14)/b8-6-
C-62	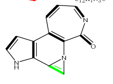 $C_{12}H_7N_3O$	27.5081	154.8625	-17.4980	14.8557	204.7839	InChI=1S/C12H7N3O/c16-12-11-8(2-1-4-14-12)7-3-5-13-10(7)9-6-15(9)11/h1-6,13H
C-15	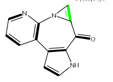 $C_{12}H_7N_3O$	22.9529	152.5482	-12.4385	20.3836	204.8985	InChI=1S/C12H7N3O/c16-11-9-6-15(9)12-8(2-1-4-14-12)7-3-5-13-10(7)11/h1-6,13H
p1-13	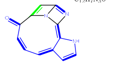 $C_{12}H_7N_3O$	4.2278	164.4442	-1.435	19.8872	205.0896	InChI=1S/C12H7N3O/c16-9-3-1-2-7-4-5-13-11(7)12-14-10-6-8(9)15(10)12/h1-6,13H/b3-1-,7-2-,12-11+
G-62	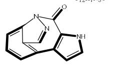 $C_{12}H_7N_3O$	5.1842	43.9755	-1.2443	129.1464	205.0977	InChI=1S/C12H7N3O/c16-12-11-8(4-5-13-11)7-2-1-3-10-9(7)6-14-15(10)12/h1-6,13H

r-58	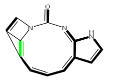 $C_{12}H_7N_3O$	17.095	150.7892	-11.6145	35.5085	205.1282	InChI=1S/C12H7N3O/c16-12-14-11-7(4-5-13-11)2-1-3-8-9-6-15(12)10(8)9/h1-6H,(H,13,14,16)/b3-1-,7-2-
B-226	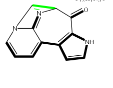 $C_{12}H_7N_3O$	5.1240	34.9950	-0.3474	142.0440	205.1912	InChI=1S/C12H7N3O/c16-11-9-6-15-5-1-2-8(12(15)14-9)7-3-4-13-10(7)11/h1-6,13H
H11-74	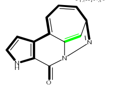 $C_{12}H_7N_3O$	4.2806	38.0105	-1.9891	138.6704	205.1943	InChI=1S/C12H7N3O/c16-12-11-9(4-5-13-11)8-3-1-2-7-6-10(8)15(12)14-7/h1-6,13H
B-203	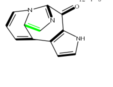 $C_{12}H_7N_3O$	6.9680	37.0691	-1.8861	131.5896	205.3937	InChI=1S/C12H7N3O/c16-11-10-8(3-4-13-10)7-2-1-5-15-9(7)6-14-12(11)15/h1-6,13H
C-67	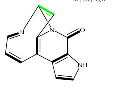 $C_{12}H_7N_3O$	5.0187	62.2587	-3.0194	131.6022	205.5432	InChI=1S/C12H7N3O/c16-12-11-8(3-5-14-11)7-2-1-4-13-10-6-9(7)15(10)12/h1-6,14H/b2-1-,4-1-,7-2-,13-4+,13-10+
A2-18	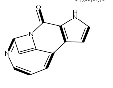 $C_{12}H_7N_3O$	5.0013	62.1845	-3.0195	131.7378	205.5477	InChI=1S/C12H7N3O/c16-12-11-8(3-5-14-11)7-2-1-4-13-10-6-9(7)15(10)12/h1-6,14H/b2-1-,4-1-,7-2-,13-4-,13-10+
x5-27	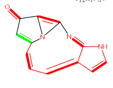 $C_{12}H_7N_3O$	12.4707	131.9353	-9.5242	46.568	205.8154	InChI=1S/C12H7N3O/c16-9-6-8-3-1-2-7-4-5-13-11(7)14-12-10(9)15(8)12/h1-6H,(H,13,14)/b3-1-,7-2-
H1-6	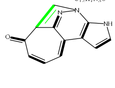 $C_{12}H_7N_3O$	2.9475	32.0025	-1.5479	151.0325	206.0314	InChI=1S/C12H7N3O/c16-10-3-1-2-7-8-4-5-13-12(8)15-6-9(10)11(7)14-15/h1-6,13H
q11-50	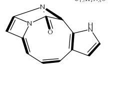 $C_{12}H_7N_3O$	2.7391	78.1386	-1.0227	122.7792	206.4645	InChI=1S/C12H7N3O/c16-12-11-10-7(4-5-13-10)2-1-3-8-6-9(14-11)15(8)12/h1-6,13H/b2-1-,3-1-,7-2-,8-3-,11-10-
A-168	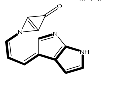 $C_{12}H_7N_3O$	50.6349	164.1033	-28.9094	10.0558	206.5689	InChI=1S/C12H7N3O/c16-11-9-10(11)15(9)5-1-2-7-6-14-12-8(7)3-4-13-12/h1-6,13H/b5-1-,7-2+
o-37	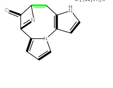 $C_{12}H_7N_3O$	2.9999	70.9155	-1.7767	116.6809	206.7321	InChI=1S/C12H7N3O/c16-12-8-6-7-9(3-4-13-7)15-5-1-2-10(15)11(12)14-8/h1-6,13H/b8-6-
B-133	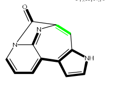 $C_{12}H_7N_3O$	4.1872	39.2540	-0.8166	146.7820	207.0045	InChI=1S/C12H7N3O/c16-12-10-6-9-7(3-4-13-9)8-2-1-5-15(12)11(8)14-10/h1-6,13H
x4-62	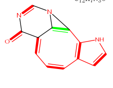 $C_{12}H_7N_3O$	12.7983	151.7478	-6.7907	19.3227	207.057	InChI=1S/C12H7N3O/c16-12-8-3-1-2-7-4-5-13-9(7)11-10(8)15(11)6-14-12/h1-6,13H/b2-1-,3-1-,7-2-,8-3+,11-9-
q1-8	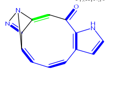 $C_{12}H_7N_3O$	7.6014	57.5418	-8.1189	116.5029	207.2088	InChI=1S/C12H7N3O/c16-10-6-9-12-8(15(9)14-12)3-1-2-7-4-5-13-11(7)10/h1-6,13H/b2-1-,8-3+,9-6+
p1-20	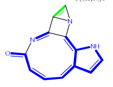 $C_{12}H_7N_3O$	17.4418	138.4803	-14.5626	41.6261	207.2405	InChI=1S/C12H7N3O/c16-9-3-1-2-7-4-5-13-10(7)12-11(14-9)8-6-15(8)12/h1-6,13H/b3-1-,7-2-,12-10-,14-11-
G-23	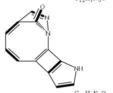 $C_{12}H_7N_3O$	7.2049	135.7461	0.0109	48.1927	207.3029	InChI=1S/C12H7N3O/c16-12-7-2-1-3-9-8-4-5-13-10(8)11(9)15(12)14-6-7/h1-6,13H/b3-1-,7-2-
B-223	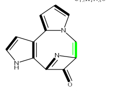 $C_{12}H_7N_3O$	2.9610	70.5162	-1.7344	116.8846	207.4899	InChI=1S/C12H7N3O/c16-12-8-6-15-5-1-2-9(15)7-3-4-13-10(7)11(12)14-8/h1-6,13H/b8-6-
o-204	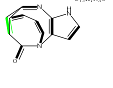 $C_{12}H_7N_3O$	4.3926	65.6973	-1.3025	129.7869	207.5686	InChI=1S/C12H7N3O/c16-12-8-6-9-7(8)2-1-5-15(12)10-3-4-13-11(10)14-9/h1-6,13H/b14-9-
A-197	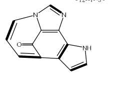 $C_{12}H_7N_3O$	5.021	47.1283	-0.1921	134.4048	208.1973	InChI=1S/C12H7N3O/c16-12-8-2-1-5-15-6-14-10(11(12)15)9-7(8)3-4-13-9/h1-6,13H

p-4	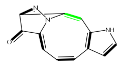 C12H7N3O	6.0387	54.2327	-5.065	129.744	208.3963	InChI=1S/C12H7N3O/c16-12-9-3-1-2-7-4-5-13-8(7)6-10-11(12)14-15(9)10/h1-6,13H/b2-1-,9-3-,10-6-
G1-65	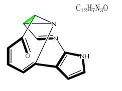 C12H7N3O	25.5558	157.1772	-13.2852	22.1485	208.4285	InChI=1S/C12H7N3O/c16-10-3-1-2-8-7-4-5-13-12(7)14-6-9-11(10)15(8)9/h1-6,13H
r-26	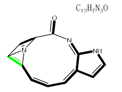 C12H7N3O	14.6717	155.5602	-10.2728	20.9095	208.4836	InChI=1S/C12H7N3O/c16-12-10-6-9-8(15(9)10)3-1-2-7-4-5-13-11(7)14-12/h1-6H,(H,13,14,16)
B-228	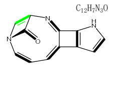 C12H7N3O	5.7565	134.8823	-2.0770	70.7178	208.5443	InChI=1S/C12H7N3O/c16-12-9-6-15(12)5-1-2-7-8-3-4-13-10(8)11(7)14-9/h1-6,13H/b5-1-,7-2-,14-11+
G-3	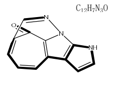 C12H7N3O	4.8578	46.7798	-0.1851	137.1473	208.5629	InChI=1S/C12H7N3O/c16-11-7-2-1-3-8-9-4-5-13-12(9)15(10(8)11)14-6-7/h1-6,13H
s12-24	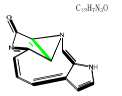 C12H7N3O	10.3107	141.2797	-6.6163	42.8587	208.8339	InChI=1S/C12H7N3O/c16-12-11-10-8(14-12)3-1-2-7-4-5-13-9(7)6-15(10)11/h1-6,13H/b3-1-,7-2-,9-6-
k1-15	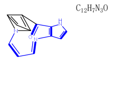 C12H7N3O	8.4273	71.8097	-7.7112	105.1349	208.9446	InChI=1S/C12H7N3O/c16-12-10-8-6-9(10)15(8)5-1-3-13-7-2-4-14-11(7)12/h1-6,14H/b5-1-,13-3+
H-49	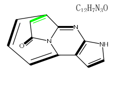 C12H7N3O	5.7122	35.2984	-2.2899	147.3529	208.9858	InChI=1S/C12H7N3O/c16-10-6-7-2-1-3-9-8-4-5-13-11(8)14-12(7)15(9)10/h1-6,13H
F-71	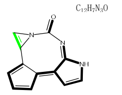 C12H7N3O	19.8158	165.3877	-13.9605	16.0914	209.0142	InChI=1S/C12H7N3O/c16-12-14-11-9(4-5-13-11)7-2-1-3-8(7)10-6-15(10)12/h1-6H,(H,13,14,16)/b9-7-
p1-26	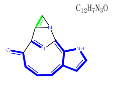 C12H7N3O	17.2208	144.6024	-11.5533	37.0872	209.3657	InChI=1S/C12H7N3O/c16-9-3-1-2-7-4-5-13-10(7)12-14-11(9)8-6-15(8)12/h1-6,13H/b3-1-,7-2-,12-10-
q1-21	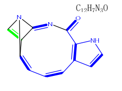 C12H7N3O	7.4753	57.8675	-8.14	117.4465	209.531	InChI=1S/C12H7N3O/c16-12-10-7(4-5-13-10)2-1-3-9-8-6-15(9)11(8)14-12/h1-6,13H/b2-1-,9-3+,14-11+
i-9	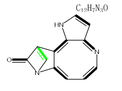 C12H7N3O	7.1186	90.4082	-3.9051	96.4049	209.5406	InChI=1S/C12H7N3O/c16-12-7-6-15(12)9-2-1-4-13-8-3-5-14-11(8)10(7)9/h1-6,14H/b2-1-,4-1-,9-2+,11-10+,13-4+,13-8+
r-49	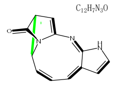 C12H7N3O	5.9773	87.5197	-4.645	112.9676	209.6268	InChI=1S/C12H7N3O/c16-12-8-6-10-14-11-7(4-5-13-11)2-1-3-9(8)15(10)12/h1-6H,(H,13,14)
H-104	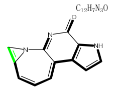 C12H7N3O	22.6259	155.2592	-12.3555	16.4426	209.6434	InChI=1S/C12H7N3O/c16-12-10-8(4-5-13-10)9-3-1-2-7-6-15(7)11(9)14-12/h1-6,13H
B-50	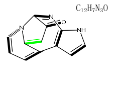 C12H7N3O	6.6254	35.7588	-1.9484	138.6494	209.8548	InChI=1S/C12H7N3O/c16-10-6-9-7-2-1-5-15(9)12(10)14-11-8(7)3-4-13-11/h1-6,13H
H1-85	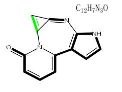 C12H7N3O	17.1261	134.0246	-12.5559	44.0856	209.8655	InChI=1S/C12H7N3O/c16-11-3-1-2-9-7-4-5-13-12(7)14-8-6-10(8)15(9)11/h1-6,13H
B-193	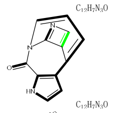 C12H7N3O	5.0650	110.0433	-2.0341	92.7341	210.7179	InChI=1S/C12H7N3O/c16-12-10-8(3-4-13-10)7-2-1-5-15(12)11-9(7)6-14-11/h1-6,13H
k-18	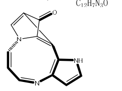 C12H7N3O	4.5711	59.3064	-4.9526	136.6344	211.0917	InChI=1S/C12H7N3O/c16-12-7-6-15-5-1-3-13-8-2-4-14-10(8)9(7)11(12)15/h1-6,14H/b5-1-,13-3-
B-191	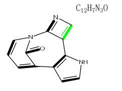 C12H7N3O	2.4485	143.5660	-1.2992	50.2164	211.2176	InChI=1S/C12H7N3O/c16-12-8-2-1-5-15(12)11-9(6-14-11)10-7(8)3-4-13-10/h1-6,13H

x6-4	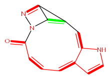 $C_{12}H_7N_3O$	16.4794	133.7688	-11.0112	50.3501	211.3448	InChI=1S/C12H7N3O/c16-10-3-1-2-7-4-5-13-9(7)6-8-11-12(8)15(10)14-11/h1-6,13H/b3-1-,7-2-,9-6+
r1-30	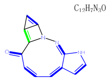 $C_{12}H_7N_3O$	13.6228	130.0755	-13.6869	59.2201	211.6549	InChI=1S/C12H7N3O/c16-10-3-1-2-7-4-5-13-12(7)14-15-9-6-8(9)11(10)15/h1-6H,(H,13,14)/b3-1-,7-2-
H1-26	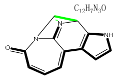 $C_{12}H_7N_3O$	3.0129	30.4782	-1.7338	163.8402	212.1507	InChI=1S/C12H7N3O/c16-10-3-1-2-8-7-4-5-13-11(7)9-6-15(10)12(8)14-9/h1-6,13H
r-50	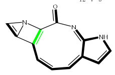 $C_{12}H_7N_3O$	18.2604	138.7853	-13.8047	50.6023	212.3206	InChI=1S/C12H7N3O/c16-12-10-8(9-6-15(9)10)3-1-2-7-4-5-13-11(7)14-12/h1-6H,(H,13,14,16)
I-60	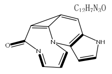 $C_{12}H_7N_3O$	11.1663	149.5885	26.2881	33.7741	212.3763	InChI=1S/C12H7N3O/c16-12-10-7-6-8-9(2-4-13-8)15(11(7)10)5-1-3-14-12/h1-6,13H/b5-1-,14-3+
s11-7	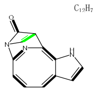 $C_{12}H_7N_3O$	7.2525	79.8439	-3.4351	102.8563	212.4776	InChI=1S/C12H7N3O/c16-12-8-6-15(12)9-3-1-2-7-4-5-13-10(7)11(8)14-9/h1-6,13H/b2-1-,3-1-,7-2-,9-3+,11-10+
A2-25	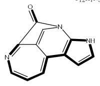 $C_{12}H_7N_3O$	2.6093	41.1356	-1.8464	152.18	212.5822	InChI=1S/C12H7N3O/c16-12-10-9-6-15(12)11-8(3-5-14-11)7(9)2-1-4-13-10/h1-6,14H
s11-12	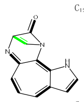 $C_{12}H_7N_3O$	5.0499	72.3172	-3.9054	120.8153	212.5874	InChI=1S/C12H7N3O/c16-12-9-6-15(12)11-8(14-9)3-1-2-7-4-5-13-10(7)11/h1-6,13H
X-37	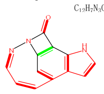 $C_{12}H_7N_3O$	5.2930	143.5597	-1.2149	48.0688	212.8026	InChI=1S/C12H7N3O/c16-12-10-9-6-7(2-1-4-14-15(9)12)8-3-5-13-11(8)10/h1-6,13H/b2-1-,14-4-
B-123	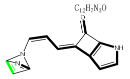 $C_{12}H_7N_3O$	7.5545	136.6673	-7.2378	63.1012	212.9245	InChI=1S/C12H7N3O/c16-12-8(7-3-4-13-11(7)12)2-1-5-15-9-6-10(15)14-9/h1-6,13H/b5-1-,8-2-
G1-83	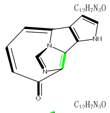 $C_{12}H_7N_3O$	4.8075	61.8324	-1.2756	127.5212	212.9374	InChI=1S/C12H7N3O/c16-9-3-1-2-8-7-4-5-13-10(7)12-11(9)14-6-15(8)12/h1-6,13H/b3-1-,8-2-
s11-28	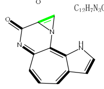 $C_{12}H_7N_3O$	17.2082	168.4311	-10.0486	11.566	213.0093	InChI=1S/C12H7N3O/c16-12-9-6-15(9)11-8(14-12)3-1-2-7-4-5-13-10(7)11/h1-6,13H
B-199	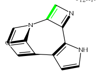 $C_{12}H_7N_3O$	2.3608	143.1694	-1.2461	51.5418	213.1124	InChI=1S/C12H7N3O/c16-12-8-2-1-5-15(12)9-6-14-11(9)10-7(8)3-4-13-10/h1-6,13H
I-108	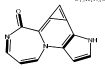 $C_{12}H_7N_3O$	15.8954	147.6461	-12.3373	36.1603	213.1998	InChI=1S/C12H7N3O/c16-12-11-8-6-7(8)10-9(2-4-13-10)15(11)5-1-3-14-12/h1-6,13H
X22-5	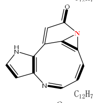 $C_{12}H_7N_3O$	13.5548	167.4098	-8.5998	24.3772	213.4219	InChI=1S/C12H7N3O/c16-10-6-7-11-8(3-5-14-11)13-4-1-2-9-12(7)15(9)10/h1-6,14H/b2-1-,13-4-
A-153	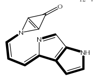 $C_{12}H_7N_3O$	50.9257	167.9063	-28.83	11.9843	213.4331	InChI=1S/C12H7N3O/c16-12-10-11(12)15(10)5-1-2-8-7-3-4-13-9(7)6-14-8/h1-6,13H/b5-1-,8-2-
G-30	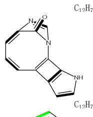 $C_{12}H_7N_3O$	7.3067	142.7552	0.102	48.7656	214.0817	InChI=1S/C12H7N3O/c16-12-9-3-1-2-8-7-4-5-13-10(7)11(8)15(12)6-14-9/h1-6,13H/b2-1-,9-3+
H-86	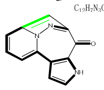 $C_{12}H_7N_3O$	3.6126	36.5293	-0.2964	149.7942	214.2306	InChI=1S/C12H7N3O/c16-12-9-6-7-2-1-3-10(15(7)14-9)8-4-5-13-11(8)12/h1-6,13H
B-204	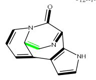 $C_{12}H_7N_3O$	3.3999	28.8555	-1.6264	167.8730	214.2956	InChI=1S/C12H7N3O/c16-12-11-10-8(3-4-13-10)7-2-1-5-15(12)9(7)6-14-11/h1-6,13H

p11-21	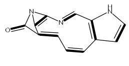 $C_{12}H_7N_3O$	15.864	175.8799	-10.8751	17.7359	214.5693	InChI=1S/C12H7N3O/c16-12-8-3-1-2-7-4-5-13-9(7)6-14-11-10(8)15(11)12/h1-6,13H/b2-1-,3-1+,7-2-,8-3-,9-6+,14-6+,14-11-
I1-3	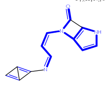 $C_{12}H_7N_3O$	19.2266	275.0135	-21.4631	41.071	214.8764	InChI=1S/C12H7N3O/c16-12-11-9(2-4-14-11)15(12)5-1-3-13-10-7-6-8(7)10/h1-6,14H/b5-1-,13-3-
G-9	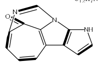 $C_{12}H_7N_3O$	5.3458	51.7549	0.0592	139.3191	215.0071	InChI=1S/C12H7N3O/c16-11-9-3-1-2-7-8-4-5-13-12(8)15(6-14-9)10(7)11/h1-6,13H
X-16	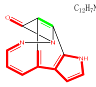 $C_{12}H_7N_3O$	5.2752	108.8523	-2.4107	86.7488	215.3045	InChI=1S/C12H7N3O/c16-12-9-6-7-2-1-4-14-15(12)11(9)10-8(7)3-5-13-10/h1-6,13H/b2-1-,14-4-
H1-20	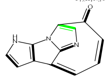 $C_{12}H_7N_3O$	4.3423	56.264	-0.8856	142.0287	215.4433	InChI=1S/C12H7N3O/c16-10-3-1-2-7-8-4-5-13-11(8)15-9(10)6-14-12(7)15/h1-6,13H/b3-1-,7-2-
B-184	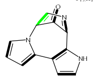 $C_{12}H_7N_3O$	2.1380	36.7230	-1.2350	160.1929	215.7348	InChI=1S/C12H7N3O/c16-12-9-6-14-11(12)10-7(3-4-13-10)8-2-1-5-15(8)9/h1-6,13H
B-237	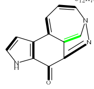 $C_{12}H_7N_3O$	4.9748	33.6539	-0.8207	147.7988	215.8487	InChI=1S/C12H7N3O/c16-12-10-8(3-4-13-10)7-2-1-5-15-6-9(7)11(12)14-15/h1-6,13H
x4-45	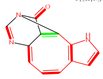 $C_{12}H_7N_3O$	4.311	81.5439	-3.1881	117.5249	215.8899	InChI=1S/C12H7N3O/c16-12-9-8-3-1-2-7-4-5-13-10(7)11(9)15(12)6-14-8/h1-6,13H/b2-1-,3-1-,7-2-,8-3+,11-10-
X-6	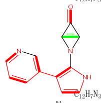 $C_{12}H_7N_3O$	53.4083	161.0656	-28.2317	12.0036	215.9809	InChI=1S/C12H7N3O/c16-11-9-10(11)15(9)12-8(3-5-14-12)7-2-1-4-13-6-7/h1-6,14H
H1-56	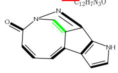 $C_{12}H_7N_3O$	4.6419	119.2208	-0.9384	74.9623	216.0661	InChI=1S/C12H7N3O/c16-10-3-1-2-7-8-4-5-13-11(8)12-9(7)6-15(10)14-12/h1-6,13H/b3-1-,7-2-
I-106	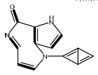 $C_{12}H_7N_3O$	19.9866	107.8697	-25.0051	86.6182	216.3031	InChI=1S/C12H7N3O/c16-12-10-9(2-4-13-10)15(5-1-3-14-12)11-7-6-8(7)11/h1-6,13H/b5-1-,14-3+
H-90	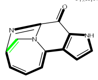 $C_{12}H_7N_3O$	5.2782	37.1637	-0.9237	144.2541	216.3191	InChI=1S/C12H7N3O/c16-11-10-8(4-5-13-10)9-3-1-2-7-6-15(9)12(11)14-7/h1-6,13H
B-215	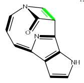 $C_{12}H_7N_3O$	5.9125	87.0923	-2.5727	112.9176	216.4565	InChI=1S/C12H7N3O/c16-12-8-6-15(12)5-1-2-9-7-3-4-13-10(7)11(8)14-9/h1-6,13H/b5-1-,9-2-
n-189	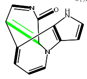 $C_{12}H_7N_3O$	3.8995	35.705	-2.3341	145.6413	216.4998	InChI=1S/C12H7N3O/c16-12-11-8(6-14-12)7-2-1-5-15(11)9-3-4-13-10(7)9/h1-6,13H
I-81	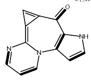 $C_{12}H_7N_3O$	22.6642	155.3525	-13.7035	32.7432	216.7913	InChI=1S/C12H7N3O/c16-11-7-6-8(7)12-14-3-1-5-15(12)9-2-4-13-10(9)11/h1-6,13H
r1-35	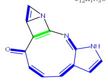 $C_{12}H_7N_3O$	18.2206	148.6619	-14.6596	41.2837	216.86	InChI=1S/C12H7N3O/c16-9-3-1-2-7-4-5-13-11(7)14-12-10(9)8-6-15(8)12/h1-6H,(H,13,14)/b3-1-,7-2-
x6-42	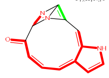 $C_{12}H_7N_3O$	18.022	129.0963	-10.3151	46.0087	216.9411	InChI=1S/C12H7N3O/c16-10-3-1-2-7-4-5-13-8(7)6-9-11-14-12(10)15(9)11/h1-6,13H/b3-1-,7-2-,8-6+
x7-22	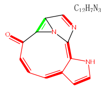 $C_{12}H_7N_3O$	19.494	127.594	-11.0706	54.7524	216.9785	InChI=1S/C12H7N3O/c16-9-3-1-2-7-4-5-13-10(7)12-14-6-8-11(9)15(8)12/h1-6,13H/b3-1-,7-2-,12-10+
p1-3	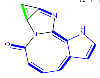 $C_{12}H_7N_3O$	13.2792	130.404	-11.8999	72.5279	217.1502	InChI=1S/C12H7N3O/c16-10-3-1-2-7-4-5-13-11(7)12-14-8-6-9(8)15(10)12/h1-6,13H/b3-1-,7-2-,12-11+

**q1-34**	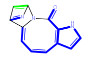	$C_{12}H_7N_3O$	5.414	52.1131	-5.9233	150.3894	217.2416	InChI=1S/C12H7N3O/c16-12-11-7(4-5-13-11)2-1-3-9-8-6-10(14-8)15(9)12/h1-6,13H/b2-1-,9-3-
**r-7**	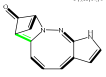	$C_{12}H_7N_3O$	4.8605	56.2857	-6.0286	148.5868	217.2528	InChI=1S/C12H7N3O/c16-11-8-6-10(11)15-9(8)3-1-2-7-4-5-13-12(7)14-15/h1-6H,(H,13,14)/b3-1-,7-2-
**x9-18**	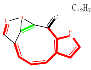	$C_{12}H_7N_3O$	7.8384	129.4834	-6.8365	62.6153	217.3212	InChI=1S/C12H7N3O/c16-12-9-7(4-5-13-9)2-1-3-8-6-14-15-10(8)11(12)15/h1-6,13H/b2-1-,8-3-
**q1-43**	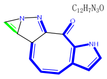	$C_{12}H_7N_3O$	16.0301	126.4601	-12.589	60.3424	217.3597	InChI=1S/C12H7N3O/c16-12-10-7(4-5-13-10)2-1-3-8-9-6-15(9)14-11(8)12/h1-6,13H/b2-1-,8-3-
**o-158**	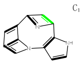	$C_{12}H_7N_3O$	3.8951	65.3956	-2.1932	143.8704	217.4127	InChI=1S/C12H7N3O/c16-12-7-2-1-5-15(12)10-3-4-13-11(10)9-6-8(7)14-9/h1-6,13H
**H-40**	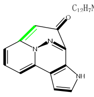	$C_{12}H_7N_3O$	2.947	25.0821	-0.6278	162.7278	217.5138	InChI=1S/C12H7N3O/c16-10-6-7-2-1-3-9-8-4-5-13-11(8)12(10)14-15(7)9/h1-6,13H
**I1-10**	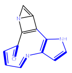	$C_{12}H_7N_3O$	18.6535	144.7436	-14.2666	35.8553	217.5173	InChI=1S/C12H7N3O/c16-9-2-1-4-13-7-3-5-14-11(7)10-8-6-15(8)12(9)10/h1-6,14H/b2-1-,13-4+
**A-65**	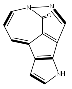	$C_{12}H_7N_3O$	4.4854	82.4943	-0.3995	99.4697	217.6143	InChI=1S/C12H7N3O/c16-12-10-7-2-1-5-15(12)14-6-9(10)11-8(7)3-4-13-11/h1-6,13H
**x4-53**	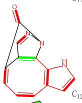	$C_{12}H_7N_3O$	4.4424	61.1269	-2.736	130.9901	217.6354	InChI=1S/C12H7N3O/c16-12-8-3-1-2-7-4-5-13-10(7)11-9(8)6-14-15(11)12/h1-6,13H/b2-1-,3-1-,7-2-,8-3+,11-10-
**H-98**	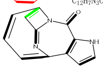	$C_{12}H_7N_3O$	7.242	72.2202	-2.6825	126.3838	217.8616	InChI=1S/C12H7N3O/c16-12-11-8(4-5-13-11)9-3-1-2-7-6-10(14-9)15(7)12/h1-6,13H/b2-1-,3-1-,7-2+,9-3-
**r1-25**	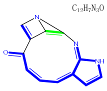	$C_{12}H_7N_3O$	14.2187	139.1267	-9.0182	46.7141	218.3283	InChI=1S/C12H7N3O/c16-9-3-1-2-7-4-5-13-11(7)14-12-10-8(9)6-15(10)12/h1-6H,(H,13,14)/b3-1-,7-2-
**q-57**	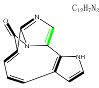	$C_{12}H_7N_3O$	11.895	121.8665	-1.3248	75.8599	218.4648	InChI=1S/C12H7N3O/c16-12-8-3-1-2-7-4-5-13-10(7)9-6-14-11(8)15(9)12/h1-6,13H/b2-1-,3-1-,7-2-,8-3+,10-9+
**q11-36**	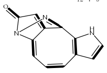	$C_{12}H_7N_3O$	4.6024	64.9014	-2.6906	127.0552	218.4982	InChI=1S/C12H7N3O/c16-10-6-8-9-3-1-2-7-4-5-13-11(7)12(8)14-15(9)10/h1-6,13H/b2-1-,9-3+
**r1-33**	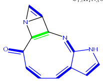	$C_{12}H_7N_3O$	17.6288	148.2085	-14.2203	39.9018	218.7731	InChI=1S/C12H7N3O/c16-9-3-1-2-7-4-5-13-12(7)14-10-8-6-15(8)11(9)10/h1-6H,(H,13,14)/b3-1-,7-2-
**p1-50**	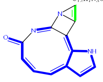	$C_{12}H_7N_3O$	16.8053	130.3852	-14.9644	69.4028	218.8639	InChI=1S/C12H7N3O/c16-9-3-1-2-7-4-5-13-11(7)10-8-6-15(8)12(10)14-9/h1-6,13H/b3-1-,7-2-,11-10+,14-12+
**A-201**	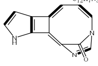	$C_{12}H_7N_3O$	4.5481	102.8645	-0.4156	101.6327	218.9168	InChI=1S/C12H7N3O/c16-12-11-9-7(8-3-4-13-10(8)9)2-1-5-15(12)6-14-11/h1-6,13H/b5-1-,7-2-,11-9-
**X22-58**	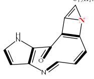	$C_{12}H_7N_3O$	18.8506	149.248	-14.0842	32.5856	218.9386	InChI=1S/C12H7N3O/c16-12-10-8(15-6-9(10)15)2-1-4-13-7-3-5-14-11(7)12/h1-6,14H/b2-1-,13-4+
**q1-1**	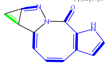	$C_{12}H_7N_3O$	13.307	175.3259	-12.9425	27.4648	218.9829	InChI=1S/C12H7N3O/c16-12-11-7(4-5-13-11)2-1-3-10-8-6-9(8)14-15(10)12/h1-6,13H/b2-1-,10-3-
**r-20**	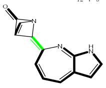	$C_{12}H_7N_3O$	30.3949	152.4808	-16.8526	36.3121	219.1508	InChI=1S/C12H7N3O/c16-12-8-6-15(12)10(8)9-3-1-2-7-4-5-13-11(7)14-9/h1-6H,(H,13,14)/b10-9-

H1-35	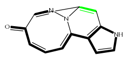	$C_{12}H_7N_3O$	8.6483	83.4488	-1.7835	99.7658	219.3646	InChI=1S/C12H7N3O/c16-11-3-1-2-9-7-4-5-13-8(7)6-10-12(11)14-15(9)10/h1-6,13H/b3-1-,9-2-
X22-52	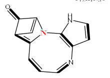	$C_{12}H_7N_3O$	4.543	51.7791	-5.6918	159.745	219.5177	InChI=1S/C12H7N3O/c16-11-7-6-10(11)15-9(7)2-1-4-13-8-3-5-14-12(8)15/h1-6,14H/b2-1-,13-4-
A-86	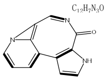	$C_{12}H_7N_3O$	13.3463	162.4563	-6.7739	20.1917	219.6635	InChI=1S/C12H7N3O/c16-12-10-7(3-4-13-10)8-2-1-5-15-9(6-14-12)11(8)15/h1-6,13H/b14-6-
A-143	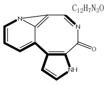	$C_{12}H_7N_3O$	13.3485	162.4604	-6.7754	20.1829	219.6635	InChI=1S/C12H7N3O/c16-12-10-7(3-4-13-10)8-2-1-5-15-9(6-14-12)11(8)15/h1-6,13H/b14-6-
F-9	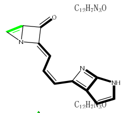	$C_{12}H_7N_3O$	18.8514	193.4215	-14.6965	15.6642	219.6932	InChI=1S/C12H7N3O/c16-11-9(15-6-10(11)15)3-1-2-8-7-4-5-13-12(7)14-8/h1-6H,(H,13,14)/b2-1-,9-3-
o-123	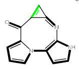	$C_{12}H_7N_3O$	15.6464	145.7608	-12.0142	38.0359	219.9552	InChI=1S/C12H7N3O/c16-11-7-6-8(7)14-12-10(3-4-13-12)15-5-1-2-9(11)15/h1-6,13H/b14-8-
x6-6	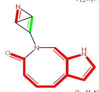	$C_{12}H_7N_3O$	29.0043	129.3372	-23.0957	72.4015	219.9775	InChI=1S/C12H7N3O/c16-9-3-1-2-7-4-5-13-8(7)6-15(9)12-10-11(12)14-10/h1-6,13H/b3-1-,7-2-,8-6+
C-37	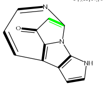	$C_{12}H_7N_3O$	4.6772	55.4682	-1.0003	144.9773	220.0923	InChI=1S/C12H7N3O/c16-9-6-10-13-4-1-2-7-8-3-5-14-12(8)15(10)11(7)9/h1-6,14H/b2-1-,13-4-
H1-33	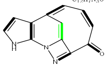	$C_{12}H_7N_3O$	3.9828	88.5684	-0.6983	108.5312	220.1362	InChI=1S/C12H7N3O/c16-10-3-1-2-7-6-9-11(10)14-15(9)12-8(7)4-5-13-12/h1-6,13H/b3-1-,7-2+
n-119	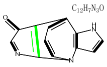	$C_{12}H_7N_3O$	3.8495	33.1956	-2.3513	155.4802	220.2087	InChI=1S/C12H7N3O/c16-9-6-14-12-10(9)7-2-1-5-15(12)8-3-4-13-11(7)8/h1-6,13H
x4-35	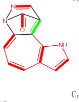	$C_{12}H_7N_3O$	3.8875	54.7172	-3.6095	146.2687	220.2441	InChI=1S/C12H7N3O/c16-12-8-6-14-15(12)9-3-1-2-7-4-5-13-11(7)10(8)9/h1-6,13H
q11-19	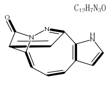	$C_{12}H_7N_3O$	5.1089	66.8269	-3.2904	129.3441	220.6268	InChI=1S/C12H7N3O/c16-12-8-6-9-11-7(4-5-13-11)2-1-3-10(8)15(12)14-9/h1-6,13H/b2-1-,10-3-
H1-65	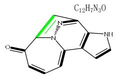	$C_{12}H_7N_3O$	3.2289	31.6704	-1.6311	163.644	220.7408	InChI=1S/C12H7N3O/c16-11-3-1-2-9-7-4-5-13-12(7)8-6-10(11)15(9)14-8/h1-6,13H
E-21	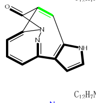	$C_{12}H_7N_3O$	3.6303	77.7746	-1.8124	129.7378	220.7776	InChI=1S/C12H7N3O/c16-12-8-6-10-7(4-5-13-10)9-2-1-3-11(8)15(12)14-9/h1-6,13H/b8-6+
p1-12	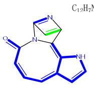	$C_{12}H_7N_3O$	5.0759	48.5427	-5.8702	161.2696	220.9824	InChI=1S/C12H7N3O/c16-10-3-1-2-7-4-5-13-11(7)12-8-6-9(14-8)15(10)12/h1-6,13H/b3-1-,7-2-,12-11+
q-43	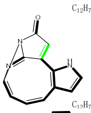	$C_{12}H_7N_3O$	2.5072	69.2206	-2.0999	148.8891	221.0597	InChI=1S/C12H7N3O/c16-10-6-8-11-7(4-5-13-11)2-1-3-9-14-12(8)15(9)10/h1-6,13H/b2-1-,9-3-
X22-6	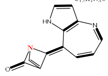	$C_{12}H_7N_3O$	30.3767	152.0426	-16.5553	36.4732	221.0696	InChI=1S/C12H7N3O/c16-12-8-6-15(12)11(8)7-2-1-4-13-9-3-5-14-10(7)9/h1-6,14H/b11-7-
C-19	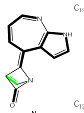	$C_{12}H_7N_3O$	31.0585	153.2240	-16.9772	34.5824	221.1188	InChI=1S/C12H7N3O/c16-12-9-6-15(12)10(9)7-2-1-4-13-11-8(7)3-5-14-11/h1-6,14H/b10-7-
n-117	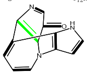	$C_{12}H_7N_3O$	3.84	33.0644	-2.3236	153.659	221.1803	InChI=1S/C12H7N3O/c16-9-6-14-11-7-2-1-5-15(12(9)11)8-3-4-13-10(7)8/h1-6,13H

k1-19	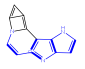 $C_{12}H_7N_3O$	15.8187	135.1096	-13.4446	49.3834	221.6505	InChI=1S/C12H7N3O/c16-12-10-8(2-4-14-10)13-3-1-5-15-9-6-7(9)11(12)15/h1-6,14H/b5-1-,13-3+
H-30	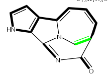 $C_{12}H_7N_3O$	5.1027	50.6598	-0.1735	140.1156	221.7946	InChI=1S/C12H7N3O/c16-12-7-2-1-3-9-8-4-5-13-10(8)11(14-12)15(9)6-7/h1-6,13H
B-165	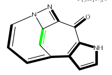 $C_{12}H_7N_3O$	4.9581	91.1158	-0.4099	101.5544	222.0292	InChI=1S/C12H7N3O/c16-12-10-8(3-4-13-10)7-2-1-5-15-9(6-7)11(12)14-15/h1-6,13H
i-16	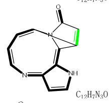 $C_{12}H_7N_3O$	4.9812	80.7523	-4.015	136.6466	222.2649	InChI=1S/C12H7N3O/c16-10-6-7-9-2-1-4-13-8-3-5-14-11(8)12(7)15(9)10/h1-6,14H/b4-1-,9-2+,12-11+,13-8-
s11-6	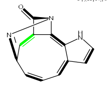 $C_{12}H_7N_3O$	4.3319	73.0847	-2.6883	123.3667	222.4647	InChI=1S/C12H7N3O/c16-12-9-6-8-3-1-2-7-4-5-13-10(7)11(9)15(12)14-8/h1-6,13H/b3-1-,7-2-,11-10-
r-36	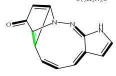 $C_{12}H_7N_3O$	5.3987	51.5376	-4.9086	153.9048	222.7868	InChI=1S/C12H7N3O/c16-10-6-9-8-3-1-2-7-4-5-13-12(7)14-15(9)11(8)10/h1-6H,(H,13,14)/b3-1-,7-2-
r1-38	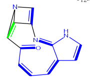 $C_{12}H_7N_3O$	11.4845	149.9043	-9.4824	53.3034	222.854	InChI=1S/C12H7N3O/c16-9-3-1-2-7-4-5-13-12(7)14-8-6-15-10(8)11(9)15/h1-6H,(H,13,14)/b3-1-,7-2-
r-52	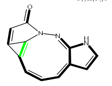 $C_{12}H_7N_3O$	13.2229	127.3957	-8.6257	69.7981	222.9527	InChI=1S/C12H7N3O/c16-10-6-9-8-3-1-2-7-4-5-13-12(7)14-15(10)11(8)9/h1-6H,(H,13,14)/b3-1-,7-2-
x5-43	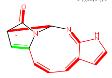 $C_{12}H_7N_3O$	5.4419	81.245	-5.0234	132.815	222.9734	InChI=1S/C12H7N3O/c16-12-9-6-8-3-1-2-7-4-5-13-10(7)14-11(9)15(8)12/h1-6H,(H,13,14)
H1-21	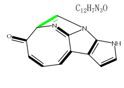 $C_{12}H_7N_3O$	16.2589	152.9878	1.3982	20.8719	223.1595	InChI=1S/C12H7N3O/c16-10-3-1-2-7-8-4-5-13-11(8)15-6-9(10)14-12(7)15/h1-6,13H/b3-1-,7-2-
H11-102	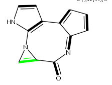 $C_{12}H_7N_3O$	15.9252	160.2903	-11.0574	35.8871	223.2278	InChI=1S/C12H7N3O/c16-12-10-6-15(10)11-8(4-5-13-11)7-2-1-3-9(7)14-12/h1-6,13H/b14-9-
X22-57	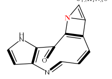 $C_{12}H_7N_3O$	19.7876	147.8836	-14.219	34.5736	223.2283	InChI=1S/C12H7N3O/c16-12-10-8(3-5-14-10)13-4-1-2-7-9-6-15(9)11(7)12/h1-6,14H/b2-1-,13-4+
q11-40	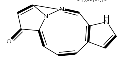 $C_{12}H_7N_3O$	10.0888	107.1905	-1.6303	82.1668	223.392	InChI=1S/C12H7N3O/c16-10-6-9-12-11-7(4-5-13-11)2-1-3-8(10)15(9)14-12/h1-6,13H/b2-1-,8-3-
D-18	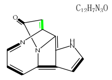 $C_{12}H_7N_3O$	4.2543	57.2443	-1.1156	148.2987	223.4708	InChI=1S/C12H7N3O/c16-10-6-8-12-11-7(3-5-14-11)9(15(10)12)2-1-4-13-8/h1-6,14H/b2-1-,13-4+
B-107	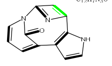 $C_{12}H_7N_3O$	4.0510	67.8195	-1.9917	144.7063	224.0918	InChI=1S/C12H7N3O/c16-12-8-2-1-5-15(12)10-6-9(14-10)11-7(8)3-4-13-11/h1-6,13H
n-191	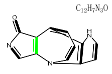 $C_{12}H_7N_3O$	3.9616	36.6296	-2.3945	153.6172	224.0984	InChI=1S/C12H7N3O/c16-12-10-7-2-1-5-15(9(10)6-14-12)8-3-4-13-11(7)8/h1-6,13H
X23-16	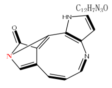 $C_{12}H_7N_3O$	4.1559	80.522	-3.8657	131.8964	224.1003	InChI=1S/C12H7N3O/c16-12-9-7-2-1-4-13-8-3-5-14-10(8)11(9)15(12)6-7/h1-6,14H/b2-1-,13-4-
l1-29	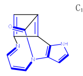 $C_{12}H_7N_3O$	5.5418	77.7291	-3.111	134.811	224.126	InChI=1S/C12H7N3O/c16-12-7-6-8-10(7)11-9(2-4-14-11)15(12)5-1-3-13-8/h1-6,14H/b5-1-,13-3+
p1-55	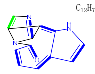 $C_{12}H_7N_3O$	5.3263	55.3768	-5.2303	158.2471	224.1305	InChI=1S/C12H7N3O/c16-9-3-1-2-7-4-5-13-11(7)10-8-6-14-12(10)15(8)9/h1-6,13H/b3-1-,7-2-,11-10+

G1-110	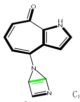	$C_{12}H_7N_3O$	18.9291	126.3952	-12.8274	66.4924	224.2216	InChI=1S/C12H7N30/c16-10-3-1-2-8(7-4-5-13-11(7)10)15-9-6-14-12(9)15/h1-6,13H
B-154	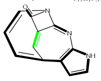	$C_{12}H_7N_3O$	4.3759	90.1342	-2.1721	118.1903	224.5032	InChI=1S/C12H7N30/c16-12-9-6-7-2-1-5-15(12)11(9)14-10-8(7)3-4-13-10/h1-6,13H/b5-1-,7-2+
q1-3	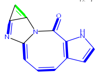	$C_{12}H_7N_3O$	13.1848	132.9094	-12.3746	73.9098	224.5108	InChI=1S/C12H7N30/c16-12-11-7(4-5-13-11)2-1-3-10-14-8-6-9(8)15(10)12/h1-6,13H/b2-1-,10-3-
r1-40	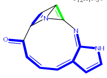	$C_{12}H_7N_3O$	17.5042	138.3289	-10.314	48.4042	224.6111	InChI=1S/C12H7N30/c16-10-3-1-2-7-4-5-13-11(7)14-12-9-6-8(10)15(9)12/h1-6H,(H,13,14)/b3-1-,7-2-
k1-14	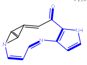	$C_{12}H_7N_3O$	36.318	157.2218	-24.7239	23.9104	224.628	InChI=1S/C12H7N30/c16-9-6-7-11-12(7)15(11)5-1-3-13-8-2-4-14-10(8)9/h1-6,14H/b5-1-,7-6-,13-3-
B-136	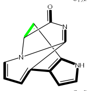	$C_{12}H_7N_3O$	6.0121	32.4184	-2.4450	157.0102	224.7193	InChI=1S/C12H7N30/c16-12-10-6-9-7(3-4-13-9)8-2-1-5-15(10)11(8)14-12/h1-6,13H
q11-66	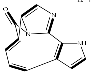	$C_{12}H_7N_3O$	13.1896	95.972	-2.4409	97.8268	224.7593	InChI=1S/C12H7N30/c16-12-8-3-1-2-7-4-5-13-10(7)11-14-6-9(8)15(11)12/h1-6,13H/b2-1-,3-1-,7-2-,8-3+,11-10+
s11-59	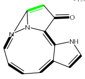	$C_{12}H_7N_3O$	9.7831	111.9354	-1.5056	78.554	224.9246	InChI=1S/C12H7N30/c16-10-6-9-8-3-1-2-7-4-5-13-11(7)12(10)15(9)14-8/h1-6,13H/b3-1-,7-2-,12-11+
x8-20	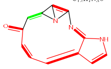	$C_{12}H_7N_3O$	17.0047	170.2675	-15.4379	21.4422	224.9869	InChI=1S/C12H7N30/c16-8-3-1-2-7-4-5-13-11(7)14-12-10-9(6-8)15(10)12/h1-6H,(H,13,14)/b3-1-,7-2-,9-6-
l1-9	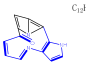	$C_{12}H_7N_3O$	17.3722	136.7354	-10.3875	48.4526	225.0473	InChI=1S/C12H7N30/c16-10-2-1-4-13-7-3-5-14-11(7)12-9-6-8(10)15(9)12/h1-6,14H/b2-1-,13-4+
G1-86	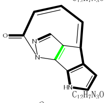	$C_{12}H_7N_3O$	7.0381	121.2254	-1.5155	77.9218	225.1296	InChI=1S/C12H7N30/c16-10-3-1-2-7-8-4-5-13-11(8)12-9(7)6-14-15(10)12/h1-6,13H/b3-1-,7-2-
p-16	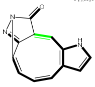	$C_{12}H_7N_3O$	6.2096	141.2172	-3.7805	68.0772	225.2802	InChI=1S/C12H7N30/c16-12-8-6-9-7(4-5-13-9)2-1-3-10-11(8)14-15(10)12/h1-6,13H/b2-1-,8-6+,10-3+
A-47	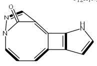	$C_{12}H_7N_3O$	4.1174	102.7829	-0.5002	108.199	225.3988	InChI=1S/C12H7N30/c16-12-9-6-14-15(12)5-1-2-7-8-3-4-13-11(8)10(7)9/h1-6,13H/b5-1-,7-2-,10-9+
x9-24	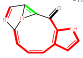	$C_{12}H_7N_3O$	16.0348	132.0522	-9.5805	66.3497	225.4263	InChI=1S/C12H7N3O/c16-12-10-7(4-5-13-10)2-1-3-9-14-6-8-11(12)15(8)9/h1-6,13H/b2-1-,9-3-
H11-161	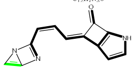	$C_{12}H_7N_3O$	18.6078	198.5772	-14.2783	14.7769	225.4585	InChI=1S/C12H7N30/c16-12-8(7-4-5-13-11(7)12)2-1-3-9-14-10-6-15(9)10/h1-6,13H/b3-1-,8-2-
X23-29	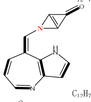	$C_{12}H_7N_3O$	53.4677	169.1962	-28.2102	12.3401	225.6032	InChI=1S/C12H7N30/c16-12-10-11(12)15(10)6-7-2-1-4-13-8-3-5-14-9(7)8/h1-6,14H/b7-6-
p-8	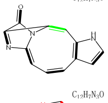	$C_{12}H_7N_3O$	4.0762	145.2442	-3.2705	73.6728	225.7477	InChI=1S/C12H7N30/c16-12-11-9-6-8-7(4-5-13-8)2-1-3-10(14-11)15(9)12/h1-6,13H/b2-1-,3-1-,7-2-,8-6+,9-6-,10-3-
x7-44	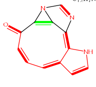	$C_{12}H_7N_3O$	12.5996	128.4863	-7.0014	66.2986	225.9575	InChI=1S/C12H7N30/c16-8-3-1-2-7-4-5-13-9(7)10-12-11(8)15(12)6-14-10/h1-6,13H/b3-1-,7-2-,10-9-
p1-1	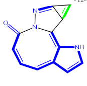	$C_{12}H_7N_3O$	14.0734	129.7645	-12.7344	81.2317	226.035	InChI=1S/C12H7N30/c16-10-3-1-2-7-4-5-13-11(7)12-8-6-9(8)14-15(10)12/h1-6,13H/b3-1-,7-2-,12-11+

x9-19	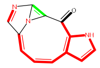 $C_{12}H_7N_3O$	16.4595	133.8775	-9.824	64.5147	226.0643	InChI=1S/C12H7N3O/c16-11-9-7(4-5-13-9)2-1-3-8-6-14-12-10(11)15(8)12/h1-6,13H/b2-1-,8-3-
G-59	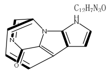 $C_{12}H_7N_3O$	6.3399	42.2367	-2.0519	147.6577	226.142	InChI=1S/C12H7N3O/c16-12-10-8-3-1-2-7(6-14-12)15(10)11-9(8)4-5-13-11/h1-6,13H
x7-27	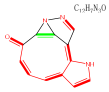 $C_{12}H_7N_3O$	7.7531	132.4495	-6.6921	69.5381	226.3537	InChI=1S/C12H7N3O/c16-9-3-1-2-7-4-5-13-10(7)8-6-14-15-11(8)12(9)15/h1-6,13H/b3-1-,7-2-,10-8+
r-3	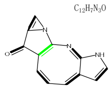 $C_{12}H_7N_3O$	19.4063	141.7956	-14.9835	66.8359	226.3763	InChI=1S/C12H7N3O/c16-10-8-3-1-2-7-4-5-13-11(7)14-12(8)15-6-9(10)15/h1-6H,(H,13,14)
A-55	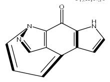 $C_{12}H_7N_3O$	5.9131	37.7618	-1.7722	149.4792	226.4694	InChI=1S/C12H7N3O/c16-12-10-8(3-4-13-10)7-2-1-5-15-11(12)9(7)6-14-15/h1-6,13H
H11-155	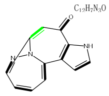 $C_{12}H_7N_3O$	9.9036	68.8427	-3.0007	116.9367	226.4853	InChI=1S/C12H7N3O/c16-11-6-10-8-2-1-3-9(15(10)14-8)7-4-5-13-12(7)11/h1-6,13H
G-34	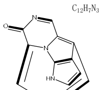 $C_{12}H_7N_3O$	6.1938	40.0236	-1.9462	151.0178	226.5027	InChI=1S/C12H7N3O/c16-12-9-3-1-2-7-8-4-5-13-11(8)15(9)10(7)6-14-12/h1-6,13H
s11-57	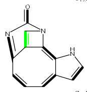 $C_{12}H_7N_3O$	5.574	70.1601	-3.4282	133.1485	226.597	InChI=1S/C12H7N3O/c16-12-14-9-3-1-2-7-4-5-13-10(7)11-8(9)6-15(11)12/h1-6,13H/b3-1-,7-2-,11-10-
q-8	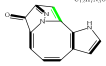 $C_{12}H_7N_3O$	4.1343	36.979	-3.0263	164.2574	226.7391	InChI=1S/C12H7N3O/c16-12-8-6-10-11-7(4-5-13-11)2-1-3-9(12)15(10)14-8/h1-6,13H/b2-1-,9-3-
r-25	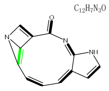 $C_{12}H_7N_3O$	13.8141	151.4293	-8.7211	49.9992	227.0231	InChI=1S/C12H7N3O/c16-12-8-6-15-9(10(8)15)3-1-2-7-4-5-13-11(7)14-12/h1-6H,(H,13,14,16)/b3-1-,7-2-
s11-54	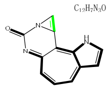 $C_{12}H_7N_3O$	17.3772	184.2837	-9.3866	11.943	227.1153	InChI=1S/C12H7N3O/c16-12-14-8-3-1-2-7-4-5-13-11(7)10(8)9-6-15(9)12/h1-6,13H
H1-123	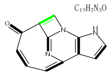 $C_{12}H_7N_3O$	6.8859	110.963	0.3284	91.466	227.1235	InChI=1S/C12H7N3O/c16-10-3-1-2-9-7-4-5-13-11(7)15-6-8(10)12(15)14-9/h1-6,13H/b3-1-,9-2-
x6-24	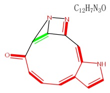 $C_{12}H_7N_3O$	9.7612	161.1797	-8.1956	35.6092	227.2352	InChI=1S/C12H7N3O/c16-10-3-1-2-7-4-5-13-8(7)6-9-11-12(10)15(11)14-9/h1-6,13H/b3-1-,7-2-,8-6+
s12-10	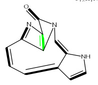 $C_{12}H_7N_3O$	7.7433	86.5087	-5.9751	132.5339	227.3681	InChI=1S/C12H7N3O/c16-12-10-11-8(14-10)3-1-2-7-4-5-13-9(7)6-15(11)12/h1-6,13H/b3-1-,7-2-,9-6-
n-166	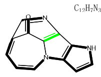 $C_{12}H_7N_3O$	5.5398	52.2455	-0.0668	143.9665	227.4273	InChI=1S/C12H7N3O/c16-12-7-2-1-5-15-8-3-4-13-9(8)10(11(12)15)14-6-7/h1-6,13H
k-31	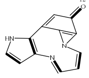 $C_{12}H_7N_3O$	15.2891	163.3559	-6.6676	29.9763	227.4451	InChI=1S/C12H7N3O/c16-9-6-7-10-8(2-4-14-10)13-3-1-5-15-11(7)12(9)15/h1-6,14H/b5-1-,13-3+
p11-12	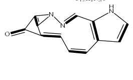 $C_{12}H_7N_3O$	13.1808	161.4249	-9.2895	39.4862	227.6249	InChI=1S/C12H7N3O/c16-12-8-3-1-2-7-4-5-13-9(7)6-14-15-10(8)11(12)15/h1-6,13H/b2-1-,8-3-,14-6+
x5-26	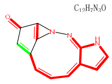 $C_{12}H_7N_3O$	7.038	155.9753	-5.1464	57.3656	227.6784	InChI=1S/C12H7N3O/c16-9-6-8-3-1-2-7-4-5-13-12(7)14-15-10(8)11(9)15/h1-6H,(H,13,14)/b3-1-,7-2-
p11-1	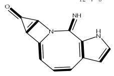 $C_{12}H_7N_3O$	42.9392	183.0573	-19.0256	12.6189	227.6851	InChI=1S/C12H7N3O/c13-12-9-6(4-5-14-9)2-1-3-7-8-10(11(8)16)15(7)12/h1-5,13-14H/b2-1-,7-3-,13-12?

q11-46	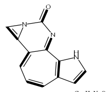 $C_{12}H_7N_3O$	17.5136	184.3489	-9.4517	11.9214	227.7688	InChI=1S/C12H7N3O/c16-12-14-11-8(9-6-15(9)12)3-1-2-7-4-5-13-10(7)11/h1-6,13H
G1-41	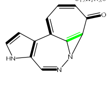 $C_{12}H_7N_3O$	5.4911	168.0252	-3.1772	28.2141	227.8499	InChI=1S/C12H7N3O/c16-10-3-1-2-8-7-4-5-13-9(7)6-14-15-11(8)12(10)15/h1-6,13H
I-94	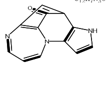 $C_{12}H_7N_3O$	3.8015	29.2239	-2.0357	168.6921	227.883	InChI=1S/C12H7N3O/c16-12-7-6-8-11(12)15(5-1-3-13-8)9-2-4-14-10(7)9/h1-6,14H
G-17	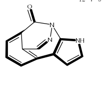 $C_{12}H_7N_3O$	5.5686	96.0939	-1.442	94.7596	228.0403	InChI=1S/C12H7N3O/c16-12-9-3-1-2-7-8-4-5-13-11(8)15(12)14-6-10(7)9/h1-6,13H
F-42	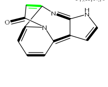 $C_{12}H_7N_3O$	5.5017	37.1308	-1.8323	163.7139	228.0867	InChI=1S/C12H7N3O/c16-11-6-8-10-3-1-2-9(15(10)11)7-4-5-13-12(7)14-8/h1-6H,(H,13,14)
X-7	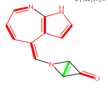 $C_{12}H_7N_3O$	53.8869	172.2992	-28.1117	10.3475	228.1907	InChI=1S/C12H7N3O/c16-11-9-10(11)15(9)6-7-2-1-4-13-12-8(7)3-5-14-12/h1-6,14H/b7-6-
r-37	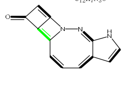 $C_{12}H_7N_3O$	6.0171	85.9405	-5.7194	128.7364	228.2057	InChI=1S/C12H7N3O/c16-10-6-9-11(10)8-3-1-2-7-4-5-13-12(7)14-15(8)9/h1-6H,(H,13,14)/b3-1-,7-2-
p1-43	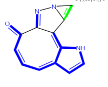 $C_{12}H_7N_3O$	16.6821	125.3602	-12.2721	73.126	228.2855	InChI=1S/C12H7N3O/c16-9-3-1-2-7-4-5-13-11(7)10-8-6-15(8)14-12(9)10/h1-6,13H/b3-1-,7-2-,11-10+
C-10	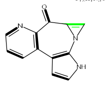 $C_{12}H_7N_3O$	22.8992	169.1708	-12.2867	23.9828	228.3335	InChI=1S/C12H7N3O/c16-11-9-6-15(9)12-8(3-5-14-12)7-2-1-4-13-10(7)11/h1-6,14H
r1-42	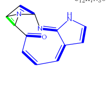 $C_{12}H_7N_3O$	13.3497	155.283	-10.2791	52.3339	228.3955	InChI=1S/C12H7N3O/c16-9-3-1-2-7-4-5-13-12(7)14-10-6-8-11(9)15(8)10/h1-6H,(H,13,14)/b3-1-,7-2-
H11-75	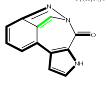 $C_{12}H_7N_3O$	4.9464	97.4025	-2.0096	99.4759	228.4168	InChI=1S/C12H7N3O/c16-12-11-8(4-5-13-11)7-2-1-3-10-9(7)6-15(12)14-10/h1-6,13H
C-63	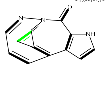 $C_{12}H_7N_3O$	26.5390	127.2790	-16.4157	65.3674	228.4815	InChI=1S/C12H7N3O/c16-12-11-8(3-5-13-11)7-2-1-4-14-15(12)10-6-9(7)10/h1-6,13H/b2-1-,14-4-
X22-41	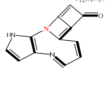 $C_{12}H_7N_3O$	6.0202	85.1302	-5.7045	132.7059	228.5261	InChI=1S/C12H7N3O/c16-10-6-9-11(10)8-2-1-4-13-7-3-5-14-12(7)15(8)9/h1-6,14H/b2-1-,13-4+
A-172	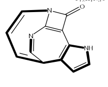 $C_{12}H_7N_3O$	5.695	97.3315	-2.2893	113.6657	228.9442	InChI=1S/C12H7N3O/c16-12-9-10-8(3-4-13-10)7-2-1-5-15(12)11(9)14-6-7/h1-6,13H/b5-1-,7-2+
n-150	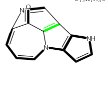 $C_{12}H_7N_3O$	7.8432	70.0965	0.5004	122.2938	229.1199	InChI=1S/C12H7N3O/c16-12-8-2-1-5-15-9-3-4-13-10(9)7(6-14-8)11(12)15/h1-6,13H
G1-82	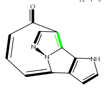 $C_{12}H_7N_3O$	4.5136	57.4885	-1.2218	148.9697	229.1829	InChI=1S/C12H7N3O/c16-10-3-1-2-9-7-4-5-13-11(7)12-8(10)6-14-15(9)12/h1-6,13H/b3-1-,9-2-
X23-15	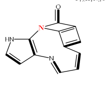 $C_{12}H_7N_3O$	5.4255	78.0872	-4.0815	140.1474	229.3894	InChI=1S/C12H7N3O/c16-12-8-6-7-2-1-4-13-9-3-5-14-11(9)15(12)10(7)8/h1-6,14H/b2-1-,13-4+
o-148	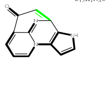 $C_{12}H_7N_3O$	4.1498	32.9299	-0.6266	164.9834	229.4777	InChI=1S/C12H7N3O/c16-10-6-8-11-9(3-4-13-11)15-5-1-2-7(10)12(15)14-8/h1-6,13H
r-33	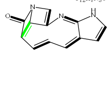 $C_{12}H_7N_3O$	5.2931	112.437	-4.7317	112.26	229.5214	InChI=1S/C12H7N3O/c16-12-8-3-1-2-7-4-5-13-11(7)14-9-6-15(12)10(8)9/h1-6H,(H,13,14)/b3-1-,7-2-

B-187	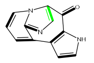 $C_{12}H_7N_3O$	6.3594	34.7582	-1.9179	160.8514	229.5353	InChI=1S/C12H7N3O/c16-11-9-6-14-12-8(2-1-5-15(9)12)7-3-4-13-10(7)11/h1-6,13H
X22-40	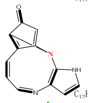 $C_{12}H_7N_3O$	4.8741	52.6574	-5.327	168.3175	229.5603	InChI=1S/C12H7N3O/c16-10-6-9-7-2-1-4-13-8-3-5-14-12(8)15(9)11(7)10/h1-6,14H/b2-1-,13-4-
B-58	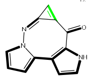 $C_{12}H_7N_3O$	14.4718	171.9581	-11.8850	33.5445	230.0207	InChI=1S/C12H7N3O/c16-12-8-6-9(8)14-15-5-1-2-10(15)7-3-4-13-11(7)12/h1-6,13H/b14-9-
o-176	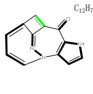 $C_{12}H_7N_3O$	9.3388	94.2249	0.3614	102.6403	230.0974	InChI=1S/C12H7N3O/c16-12-8-6-7-2-1-5-15(14-10(7)8)9-3-4-13-11(9)12/h1-6,13H
B-55	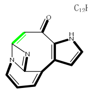 $C_{12}H_7N_3O$	10.0110	82.6531	-1.5205	115.5414	230.1907	InChI=1S/C12H7N3O/c16-9-6-10-14-12-8(2-1-5-15(10)12)7-3-4-13-11(7)9/h1-6,13H/b10-6+
s12-47	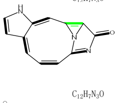 $C_{12}H_7N_3O$	19.3885	133.1544	-10.7308	59.3525	230.2168	InChI=1S/C12H7N3O/c16-12-11-9-6-8-7(4-5-13-8)2-1-3-10(14-12)15(9)11/h1-6,13H/b3-1-,7-2-,8-6+
o-127	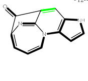 $C_{12}H_7N_3O$	5.4239	43.3885	-0.496	155.9307	230.6668	InChI=1S/C12H7N3O/c16-11-7-6-9-10(3-4-13-9)15-5-1-2-8(11)14-12(7)15/h1-6,13H
H11-36	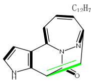 $C_{12}H_7N_3O$	6.2618	35.3051	-2.1832	162.7873	231.0737	InChI=1S/C12H7N3O/c16-12-9-6-7-2-1-3-10(15(12)14-7)8-4-5-13-11(8)9/h1-6,13H
G-40	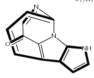 $C_{12}H_7N_3O$	6.1572	41.4521	-1.7434	156.7944	231.1564	InChI=1S/C12H7N3O/c16-9-6-14-10-3-1-2-7-8-4-5-13-12(8)15(10)11(7)9/h1-6,13H
x7-30	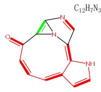 $C_{12}H_7N_3O$	20.3482	132.4381	-11.3554	66.4233	231.2409	InChI=1S/C12H7N3O/c16-9-3-1-2-7-4-5-13-10(7)8-6-14-12-11(9)15(8)12/h1-6,13H/b3-1-,7-2-,10-8+
o-118	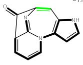 $C_{12}H_7N_3O$	4.7946	40.6438	-0.5251	162.3902	231.2451	InChI=1S/C12H7N3O/c16-11-7-2-1-5-15-10-3-4-13-8(10)6-9(11)14-12(7)15/h1-6,13H
x8-19	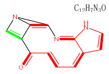 $C_{12}H_7N_3O$	12.6105	162.9416	-9.5	44.6892	231.2846	InChI=1S/C12H7N3O/c16-9-3-1-2-7-4-5-13-11(7)14-12-10-8(9)6-15(10)12/h1-6H,(H,13,14)/b3-1+,7-2-
G-15	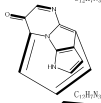 $C_{12}H_7N_3O$	5.8282	39.0806	-1.8029	160.7818	231.3071	InChI=1S/C12H7N3O/c16-10-6-14-12-7-2-1-3-9(10)15(12)11-8(7)4-5-13-11/h1-6,13H
H-95	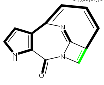 $C_{12}H_7N_3O$	5.3762	66.5584	-3.0319	147.1722	231.4534	InChI=1S/C12H7N3O/c16-12-10-8(4-5-13-10)9-3-1-2-7-6-15(12)11(7)14-9/h1-6,13H
B-167	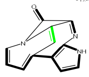 $C_{12}H_7N_3O$	4.0089	83.5312	-2.3711	131.8013	232.0434	InChI=1S/C12H7N3O/c16-12-10-9-6-7(2-1-5-15(9)12)8-3-4-13-11(8)14-10/h1-6,13H
F-16	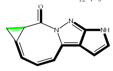 $C_{12}H_7N_3O$	25.4538	145.7628	-16.7352	58.2731	232.1061	InChI=1S/C12H7N3O/c16-12-9-6-7(9)2-1-3-10-8-4-5-13-11(8)14-15(10)12/h1-6H,(H,13,14)/b3-1-,7-2-
H1-13	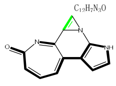 $C_{12}H_7N_3O$	11.2978	201.4482	-4.407	0.2928	232.1665	InChI=1S/C12H7N3O/c16-10-3-1-2-7-8-4-5-13-12(8)15-6-9(15)11(7)14-10/h1-6,13H
H1-128	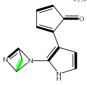 $C_{12}H_7N_3O$	26.8766	129.5682	-11.9062	72.6757	232.1763	InChI=1S/C12H7N3O/c16-9-3-1-2-7(9)8-4-5-13-12(8)15-10-6-11(15)14-10/h1-6,13H
H-97	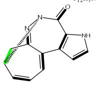 $C_{12}H_7N_3O$	3.7037	34.0406	-1.1905	171.5263	232.2515	InChI=1S/C12H7N3O/c16-12-11-9(4-5-13-11)8-3-1-2-7-6-10(8)14-15(7)12/h1-6,13H

o-129	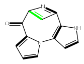 $C_{12}H_7N_3O$	5.4166	111.5226	-2.3068	90.8058	232.5158	InChI=1S/C12H7N3O/c16-12-8-6-7(14-8)11-9(3-4-13-11)15-5-1-2-10(12)15/h1-6,13H
E-45	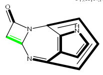 $C_{12}H_7N_3O$	2.6309	87.4731	-1.4917	137.3528	232.5638	InChI=1S/C12H7N3O/c16-11-6-10-14-8-2-1-3-9(15(10)11)12-7(8)4-5-13-12/h1-6,13H
r1-27	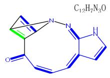 $C_{12}H_7N_3O$	9.5498	70.995	-7.6582	122.0955	232.633	InChI=1S/C12H7N3O/c16-10-3-1-2-7-4-5-13-12(7)14-15-8-6-9(15)11(8)10/h1-6H,(H,13,14)/b3-1-,7-2-
X23-44	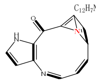 $C_{12}H_7N_3O$	18.3949	167.5106	-9.0148	39.9135	232.7164	InChI=1S/C12H7N3O/c16-12-10-8(3-5-14-10)13-4-1-2-7-6-9-11(12)15(7)9/h1-6,14H/b2-1-,13-4-
q1-32	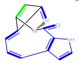 $C_{12}H_7N_3O$	5.2401	48.3767	-5.4399	165.6213	232.7223	InChI=1S/C12H7N3O/c16-12-11-7(4-5-13-11)2-1-3-8-9-6-10(8)15(12)14-9/h1-6,13H/b2-1-,8-3+
C-61	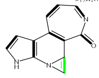 $C_{12}H_7N_3O$	11.5383	201.0088	-4.4427	0.1180	232.7302	InChI=1S/C12H7N3O/c16-12-10-7(2-1-4-14-12)8-3-5-13-11(8)15-6-9(10)15/h1-6,13H
x2-18	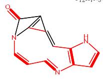 $C_{12}H_7N_3O$	19.4285	147.9367	-13.5681	66.9484	232.9439	InChI=1S/C12H7N3O/c16-12-10-7-6-9-8(2-4-14-9)13-3-1-5-15(12)11(7)10/h1-6,14H/b5-1-,7-6+,13-3+
q1-42	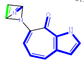 $C_{12}H_7N_3O$	30.1319	124.7522	-11.8872	59.0494	232.9637	InChI=1S/C12H7N3O/c16-12-8(15-9-6-10(15)14-9)3-1-2-7-4-5-13-11(7)12/h1-6,13H
X22-4	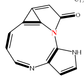 $C_{12}H_7N_3O$	7.6323	183.696	-8.0542	34.7744	233.1502	InChI=1S/C12H7N3O/c16-10-6-8-7-2-1-4-13-9-3-5-14-12(9)15(10)11(7)8/h1-6,14H/b2-1-,13-4-
F-69	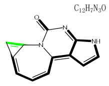 $C_{12}H_7N_3O$	15.9111	199.066	-7.0721	10.0136	233.1561	InChI=1S/C12H7N3O/c16-12-14-11-8(4-5-13-11)9-3-1-2-7-6-10(7)15(9)12/h1-6H,(H,13,14,16)
x7-17	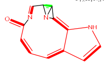 $C_{12}H_7N_3O$	18.2895	149.2043	-15.4691	52.1892	233.6744	InChI=1S/C12H7N3O/c16-9-3-1-2-7-4-5-13-10(7)12-11-8(6-14-9)15(11)12/h1-6,13H/b3-1-,7-2-,12-10+,14-6+
B-208	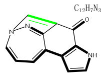 $C_{12}H_7N_3O$	11.8450	110.0315	0.2354	74.3510	233.6863	InChI=1S/C12H7N3O/c16-12-9-6-15-5-1-2-7(10(9)14-15)8-3-4-13-11(8)12/h1-6,13H
o-70	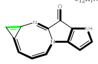 $C_{12}H_7N_3O$	15.2078	168.682	-12.9434	38.1839	233.7024	InChI=1S/C12H7N3O/c16-11-10-9(3-4-13-10)15-5-1-2-7-6-8(7)14-12(11)15/h1-6,13H/b5-1-,7-2-,14-12-
A-160	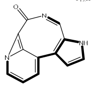 $C_{12}H_7N_3O$	13.6497	152.8499	-6.4192	46.7986	233.7059	InChI=1S/C12H7N3O/c16-12-11-10-8(2-1-5-15(10)11)7-3-4-13-9(7)6-14-12/h1-6,13H/b14-6-
H1-5	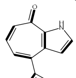 $C_{12}H_7N_3O$	18.882	147.977	-15.0609	62.804	233.7534	InChI=1S/C12H7N3O/c16-10-3-1-2-7(8-4-5-13-12(8)10)11-9-6-15(9)14-11/h1-6,13H
G1-90	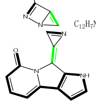 $C_{12}H_7N_3O$	18.0752	140.2962	-11.6047	72.2171	233.76	InChI=1S/C12H7N3O/c16-10-3-1-2-9-7-4-5-13-11(7)12(15(9)10)8-6-14-8/h1-6,13H/b12-8+
r-38	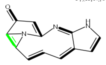 $C_{12}H_7N_3O$	12.6606	184.8085	-9.2839	26.6117	233.8792	InChI=1S/C12H7N3O/c16-9-6-10-14-12-7(4-5-13-12)2-1-3-8-11(9)15(8)10/h1-6H,(H,13,14)/b3-1-,7-2-
C-49	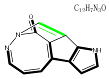 $C_{12}H_7N_3O$	10.1922	146.7333	0.6096	51.2103	234.0225	InChI=1S/C12H7N3O/c16-12-10-7-2-1-4-14-15(12)6-9(10)11-8(7)3-5-13-11/h1-6,13H/b2-1-,14-4-
B-92	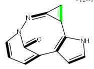 $C_{12}H_7N_3O$	20.6992	144.3666	-15.1999	69.6780	234.0546	InChI=1S/C12H7N3O/c16-12-8-2-1-5-15(12)14-10-6-9(10)11-7(8)3-4-13-11/h1-6,13H/b14-10-

q-34	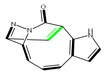 $C_{12}H_7N_3O$	5.5639	66.0165	-3.0827	146.0699	234.0986	InChI=1S/C12H7N3O/c16-12-8-6-9-10(15(12)14-9)3-1-2-7-4-5-13-11(7)8/h1-6,13H/b2-1-,10-3-
H-55	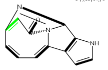 $C_{12}H_7N_3O$	6.5222	49.7558	-1.7669	155.2879	234.2652	InChI=1S/C12H7N3O/c16-10-6-7-2-1-3-9-8-4-5-13-11(8)12(14-7)15(9)10/h1-6,13H/b2-1-,9-3-
k-30	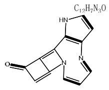 $C_{12}H_7N_3O$	6.0001	85.0581	-5.6872	135.7164	234.3206	InChI=1S/C12H7N3O/c16-9-6-8-10(9)12-11-7(2-4-14-11)13-3-1-5-15(8)12/h1-6,14H/b5-1-,13-3+
B-169	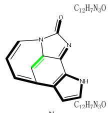 $C_{12}H_7N_3O$	3.8988	92.6715	-0.6888	118.0569	234.4115	InChI=1S/C12H7N3O/c16-12-14-11-9-6-7(2-1-5-15(9)12)8-3-4-13-10(8)11/h1-6,13H
A-54	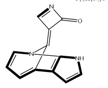 $C_{12}H_7N_3O$	4.4632	89.1429	-3.0975	124.7452	234.567	InChI=1S/C12H7N3O/c16-12-9(6-14-12)10-7-2-1-5-15(10)11-8(7)3-4-13-11/h1-6,13H/b10-9+
A-138	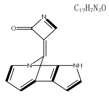 $C_{12}H_7N_3O$	4.4627	89.1406	-3.0973	124.7462	234.567	InChI=1S/C12H7N3O/c16-12-9(6-14-12)10-7-2-1-5-15(10)11-8(7)3-4-13-11/h1-6,13H/b10-9-
B-118	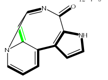 $C_{12}H_7N_3O$	5.4979	72.4402	-1.2768	131.5807	234.7420	InChI=1S/C12H7N3O/c16-12-11-8(3-4-13-11)7-2-1-5-15-9(7)6-10(15)14-12/h1-6,13H/b14-10-
x4-10	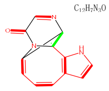 $C_{12}H_7N_3O$	5.237	57.8784	-4.2308	156.6333	234.863	InChI=1S/C12H7N3O/c16-9-6-14-11-8-3-1-2-7-4-5-13-10(7)12(11)15(8)9/h1-6,13H/b2-1-,3-1-,7-2-,8-3+,12-10+
x9-22	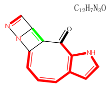 $C_{12}H_7N_3O$	5.7086	97.0237	-4.8935	118.8518	234.906	InChI=1S/C12H7N3O/c16-12-10-8(15-9(10)6-14-15)3-1-2-7-4-5-13-11(7)12/h1-6,13H/b2-1-,8-3+
H11-55	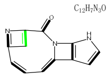 $C_{12}H_7N_3O$	6.3126	180.2328	-0.0963	43.0576	234.9871	InChI=1S/C12H7N3O/c16-12-9-6-7(14-9)2-1-3-10-8-4-5-13-11(8)15(10)12/h1-6,13H/b2-1-,10-3-
G1-107	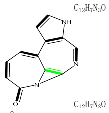 $C_{12}H_7N_3O$	19.4549	186.8631	-6.3525	20.8858	234.9956	InChI=1S/C12H7N3O/c16-10-3-1-2-8-7-4-5-13-9(7)6-14-12-11(8)15(10)12/h1-6,13H
A-3	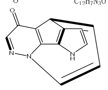 $C_{12}H_7N_3O$	6.8185	45.5305	-1.6038	150.2606	235.0039	InChI=1S/C12H7N3O/c16-9-6-14-15-5-1-2-7-8-3-4-13-11(8)12(15)10(7)9/h1-6,13H
n-183	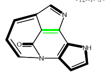 $C_{12}H_7N_3O$	4.473	84.6969	-0.4592	117.5213	235.1446	InChI=1S/C12H7N3O/c16-12-9-7-2-1-5-15(12)8-3-4-13-10(8)11(9)14-6-7/h1-6,13H
x7-37	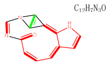 $C_{12}H_7N_3O$	42.4627	144.8357	-27.9588	46.3108	235.1549	InChI=1S/C12H7N3O/c16-8-3-1-2-7-4-5-13-10(7)9-11-12(9)15(11)6-14-8/h1-6,13H/b3-1-,7-2-,10-9-,14-6-
H-67	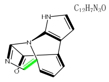 $C_{12}H_7N_3O$	6.4168	44.4882	-1.9093	157.6008	235.1678	InChI=1S/C12H7N3O/c16-10-8-3-1-2-7-6-14-12(10)15(7)11-9(8)4-5-13-11/h1-6,13H/b2-1-,8-3-
p1-59	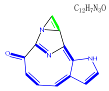 $C_{12}H_7N_3O$	19.5343	141.4102	-13.0237	60.1465	235.2569	InChI=1S/C12H7N3O/c16-9-3-1-2-7-4-5-13-10(7)11-8-6-15(8)12(9)14-11/h1-6,13H/b3-1-,7-2-,11-10+
A-114	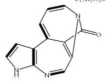 $C_{12}H_7N_3O$	3.4111	121.5172	-1.6734	98.4001	235.2985	InChI=1S/C12H7N3O/c16-12-10-7-2-1-5-15(12)9(10)6-14-11-8(7)3-4-13-11/h1-6,13H
H-157	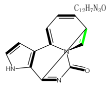 $C_{12}H_7N_3O$	5.3406	34.793	-1.6585	172.1692	235.308	InChI=1S/C12H7N3O/c16-12-14-10-7-2-1-3-9(15(12)6-7)8-4-5-13-11(8)10/h1-6,13H
I1-35	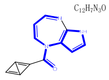 $C_{12}H_7N_3O$	16.4049	190.4723	-20.7734	46.8782	235.3859	InChI=1S/C12H7N3O/c16-12(10-7-6-8(7)10)15-5-1-3-13-11-9(15)2-4-14-11/h1-6,14H

B-32	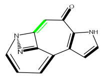 $C_{12}H_7N_3O$	4.0310	53.8065	-2.0160	153.6411	235.7093	InChI=1S/C12H7N3O/c16-10-6-9-11-7(2-1-5-15(9)14-11)8-3-4-13-12(8)10/h1-6,13H
q11-56	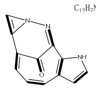 $C_{12}H_7N_3O$	14.7876	131.044	-11.0555	70.2386	235.9875	InChI=1S/C12H7N3O/c16-12-8-3-1-2-7-4-5-13-10(7)11(12)14-15-6-9(8)15/h1-6,13H/b2-1-,8-3-
C-64	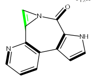 $C_{12}H_7N_3O$	35.3079	175.0093	-15.8221	18.2245	236.1323	InChI=1S/C12H7N3O/c16-12-11-8(3-5-14-11)7-2-1-4-13-10(7)9-6-15(9)12/h1-6,14H
A2-2	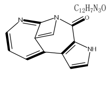 $C_{12}H_7N_3O$	4.846	103.887	-2.8188	107.8602	236.2458	InChI=1S/C12H7N3O/c16-12-10-8(3-5-13-10)7-2-1-4-14-11-9(7)6-15(11)12/h1-6,13H
F-38	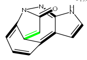 $C_{12}H_7N_3O$	7.1382	52.8548	-2.3348	151.3416	236.2717	InChI=1S/C12H7N3O/c16-11-6-9-7-2-1-3-10(9)15(11)14-12-8(7)4-5-13-12/h1-6H,(H,13,14)
r-40	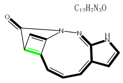 $C_{12}H_7N_3O$	5.212	53.1064	-5.5346	168.0242	236.3113	InChI=1S/C12H7N3O/c16-12-9-6-10-8(9)3-1-2-7-4-5-13-11(7)14-15(10)12/h1-6H,(H,13,14)/b3-1-,7-2-
r-42	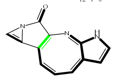 $C_{12}H_7N_3O$	21.662	156.4119	-15.0252	62.5204	236.5194	InChI=1S/C12H7N3O/c16-12-10-8(9-6-15(9)12)3-1-2-7-4-5-13-11(7)14-10/h1-6H,(H,13,14)
I-74	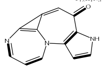 $C_{12}H_7N_3O$	16.8217	129.1038	-11.7042	74.4554	236.6476	InChI=1S/C12H7N3O/c16-9-6-7-10-12(7)15(5-1-3-13-10)8-2-4-14-11(8)9/h1-6,14H
A-206	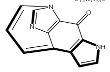 $C_{12}H_7N_3O$	5.6489	34.7901	-1.9616	163.9372	236.6877	InChI=1S/C12H7N3O/c16-12-10-8(3-4-13-10)7-2-1-5-15-6-14-9(7)11(12)15/h1-6,13H
s12-45	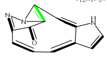 $C_{12}H_7N_3O$	11.9854	148.9434	-6.8837	49.096	236.7013	InChI=1S/C12H7N3O/c16-12-8-3-1-2-7-4-5-13-9(7)6-10-11(12)15(10)14-8/h1-6,13H/b3-1-,7-2-,9-6-
I-36	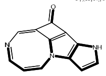 $C_{12}H_7N_3O$	13.3774	213.0623	-1.6772	0.74	236.7718	InChI=1S/C12H7N3O/c16-12-7-6-13-3-1-5-15-8-2-4-14-10(8)9(12)11(7)15/h1-6,14H/b5-1-,7-6+,13-3-
B-162	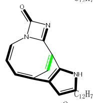 $C_{12}H_7N_3O$	3.6082	155.1536	-1.3118	75.5759	236.8136	InChI=1S/C12H7N3O/c16-12-14-11-9-6-7(2-1-5-15(11)12)8-3-4-13-10(8)9/h1-6,13H/b5-1-,7-2+
H-102	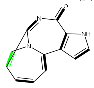 $C_{12}H_7N_3O$	8.3104	69.148	-2.949	127.886	236.8199	InChI=1S/C12H7N3O/c16-12-10-8(4-5-13-10)9-3-1-2-7-6-15(9)11(7)14-12/h1-6,13H
x9-23	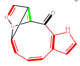 $C_{12}H_7N_3O$	5.5449	58.9511	-4.6161	151.7238	236.8921	InChI=1S/C12H7N3O/c16-12-10-7(4-5-13-10)2-1-3-9-8-6-14-15(9)11(8)12/h1-6,13H/b2-1-,9-3+
n-145	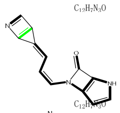 $C_{12}H_7N_3O$	15.8594	200.659	-11.8811	29.0824	236.9182	InChI=1S/C12H7N3O/c16-12-11-9(3-4-13-11)15(12)5-1-2-7-8-6-14-10(7)8/h1-6,13H/b5-1-,7-2-
A-157	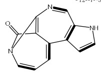 $C_{12}H_7N_3O$	3.3946	120.0503	-1.7466	100.9181	236.9842	InChI=1S/C12H7N3O/c16-12-10-8-2-1-5-15(12)11(10)14-6-9-7(8)3-4-13-9/h1-6,13H
n-176	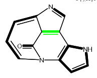 $C_{12}H_7N_3O$	4.5784	86.4905	-0.4668	117.6719	236.9969	InChI=1S/C12H7N3O/c16-12-10-7-6-14-8(10)2-1-5-15(12)9-3-4-13-11(7)9/h1-6,13H
p-3	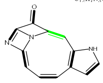 $C_{12}H_7N_3O$	5.3453	93.8672	-4.345	128.9922	237.0443	InChI=1S/C12H7N3O/c16-11-9-6-8-7(4-5-13-8)2-1-3-10-14-12(11)15(9)10/h1-6,13H/b2-1-,9-6-,10-3-
x4-47	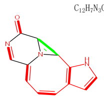 $C_{12}H_7N_3O$	13.4872	192.5869	-6.6971	10.1273	237.5042	InChI=1S/C12H7N3O/c16-12-11-10-9-7(4-5-13-9)2-1-3-8(6-14-12)15(10)11/h1-6,13H/b2-1-,8-3-

C-55	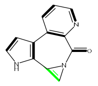	$C_{12}H_7N_3O$	33.9860	174.3142	-15.0482	17.3311	237.5734	InChI=1S/C12H7N3O/c16-12-11-7(2-1-4-13-11)8-3-5-14-10(8)9-6-15(9)12/h1-6,14H
o-149	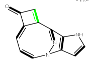	$C_{12}H_7N_3O$	4.6514	95.6722	-1.7943	117.1836	237.8869	InChI=1S/C12H7N3O/c16-10-6-8-7(10)2-1-5-15-9-3-4-13-12(9)11(8)14-15/h1-6,13H/b5-1-,7-2+
k1-16	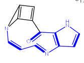	$C_{12}H_7N_3O$	22.535	148.0259	-12.0882	42.6975	237.9141	InChI=1S/C12H7N3O/c16-12-7-6-9-11(7)15(9)5-1-3-13-8-2-4-14-10(8)12/h1-6,14H/b5-1-,13-3+
o-253	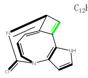	$C_{12}H_7N_3O$	5.9063	60.5419	-3.1271	153.8771	238.3723	InChI=1S/C12H7N3O/c16-12-14-9-6-8-7(9)2-1-5-15(12)10-3-4-13-11(8)10/h1-6,13H/b5-1-,7-2+,14-9-
B-130	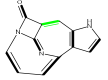	$C_{12}H_7N_3O$	4.1157	87.4054	-2.0463	138.5348	238.4131	InChI=1S/C12H7N3O/c16-12-8-6-10-7(3-4-13-10)9-2-1-5-15(12)11(8)14-9/h1-6,13H
p1-14	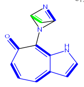	$C_{12}H_7N_3O$	28.9104	126.1455	-11.5132	66.7335	238.435	InChI=1S/C12H7N3O/c16-8-3-1-2-7-4-5-13-11(7)12(8)15-9-6-10(15)14-9/h1-6,13H
H1-134	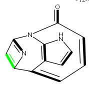	$C_{12}H_7N_3O$	5.0438	70.585	-2.743	149.1579	238.5059	InChI=1S/C12H7N3O/c16-11-3-1-2-7-8-4-5-13-12(8)15(11)10-6-9(7)14-10/h1-6,13H/b3-1-,7-2-
o-20	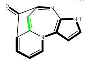	$C_{12}H_7N_3O$	5.6163	39.0538	-1.1884	166.3329	238.577	InChI=1S/C12H7N3O/c16-11-7-2-1-5-15-9-3-4-13-12(9)14-8(11)6-10(7)15/h1-6,13H
p11-14	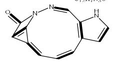	$C_{12}H_7N_3O$	18.0268	118.6225	-12.7712	99.8425	238.7343	InChI=1S/C12H7N3O/c16-12-10-8-3-1-2-7-4-5-13-9(7)6-14-15(12)11(8)10/h1-6,13H/b2-1-,8-3-,14-6-
x4-4	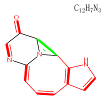	$C_{12}H_7N_3O$	13.6235	194.1125	-6.8663	13.0311	238.7979	InChI=1S/C12H7N3O/c16-8-6-14-9-3-1-2-7-4-5-13-10(7)12-11(8)15(9)12/h1-6,13H/b2-1-,9-3-
H-73	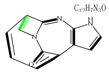	$C_{12}H_7N_3O$	6.2084	73.3471	-2.3343	153.5252	238.8004	InChI=1S/C12H7N3O/c16-12-9-3-1-2-7-6-15(12)11(7)14-10-8(9)4-5-13-10/h1-6,13H/b2-1-,9-3-
x4-38	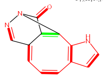	$C_{12}H_7N_3O$	7.7595	143.7756	-3.2322	67.2917	238.9582	InChI=1S/C12H7N3O/c16-12-9-8-3-1-2-7-4-5-13-10(7)11(9)15(12)14-6-8/h1-6,13H/b2-1-,3-1-,7-2-,8-3-,11-10-
x9-48	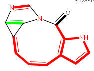	$C_{12}H_7N_3O$	12.3702	126.511	-8.4338	82.1383	239.1765	InChI=1S/C12H7N3O/c16-12-9-7(4-5-13-9)2-1-3-8-10-11(8)15(12)6-14-10/h1-6,13H/b2-1-,8-3-
x7-32	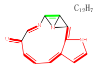	$C_{12}H_7N_3O$	17.7841	147.9991	-15.4866	56.7966	239.1875	InChI=1S/C12H7N3O/c16-8-3-1-2-7-4-5-13-9(7)10-11-12(14-6-8)15(10)11/h1-6,13H/b3-1-,7-2-,10-9+,14-6+
o-167	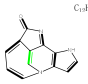	$C_{12}H_7N_3O$	5.7022	43.7935	-0.1122	164.9248	239.3381	InChI=1S/C12H7N3O/c16-12-7-2-1-5-15-6-8(7)10(14-12)11-9(15)3-4-13-11/h1-6,13H
X23-46	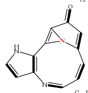	$C_{12}H_7N_3O$	22.3422	135.698	-11.2101	63.0155	239.3771	InChI=1S/C12H7N3O/c16-9-6-7-2-1-4-13-8-3-5-14-10(8)12-11(9)15(7)12/h1-6,14H/b2-1-,13-4-
x6-5	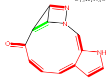	$C_{12}H_7N_3O$	22.89	150.1825	-12.3508	59.3132	239.4146	InChI=1S/C12H7N3O/c16-9-3-1-2-7-4-5-13-8(7)6-15-12-10(9)11(12)14-15/h1-6,13H/b3-1-,7-2-,8-6+
o-243	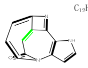	$C_{12}H_7N_3O$	2.4676	61.6351	-1.8065	171.6128	239.4483	InChI=1S/C12H7N3O/c16-10-6-7-8-2-1-5-15(10)9-3-4-13-12(9)11(7)14-8/h1-6,13H/b5-1-,8-2+
o-54	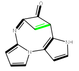	$C_{12}H_7N_3O$	6.4289	122.9127	-2.2845	85.882	239.5157	InChI=1S/C12H7N3O/c16-12-7-6-8(12)14-10-2-1-5-15(10)9-3-4-13-11(7)9/h1-6,13H/b14-8+

x6-26	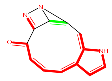 $C_{12}H_7N_3O$	13.4144	161.1687	-9.0753	41.6756	239.5438	InChI=1S/C12H7N3O/c16-10-3-1-2-7-4-5-13-8(7)6-9-12-11(10)14-15(9)12/h1-6,13H/b3-1-,7-2-,8-6+
x7-31	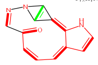 $C_{12}H_7N_3O$	19.522	139.2145	-18.4576	58.5042	239.7469	InChI=1S/C12H7N3O/c16-8-3-1-2-7-4-5-13-10(7)9-11-12(9)15(11)14-6-8/h1-6,13H/b3-1-,7-2-,10-9-,14-6-
o-172	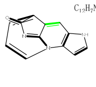 $C_{12}H_7N_3O$	6.407	43.7696	-2.0468	155.4234	239.7625	InChI=1S/C12H7N3O/c16-12-7-2-1-5-15-10-3-4-13-9(10)6-8(7)11(15)14-12/h1-6,13H
x9-41	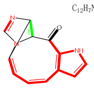 $C_{12}H_7N_3O$	5.3661	58.3066	-4.9588	160.8194	239.8689	InChI=1S/C12H7N3O/c16-12-9-7(4-5-13-9)2-1-3-8-10-11(12)15(8)6-14-10/h1-6,13H/b2-1-,8-3+
q-59	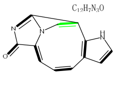 $C_{12}H_7N_3O$	10.526	109.9774	-1.7253	94.982	240.0184	InChI=1S/C12H7N3O/c16-12-9-3-1-2-7-4-5-13-10(7)8-6-15(9)11(8)14-12/h1-6,13H/b2-1-,9-3-
I1-4	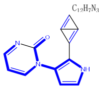 $C_{12}H_7N_3O$	19.8556	191.9848	-21.8644	45.7405	240.0277	InChI=1S/C12H7N3O/c16-12-14-3-1-5-15(12)9-2-4-13-11(9)10-7-6-8(7)10/h1-6,13H
B-81	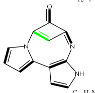 $C_{12}H_7N_3O$	6.2613	119.3281	-2.2587	87.2091	240.0513	InChI=1S/C12H7N3O/c16-11-8-6-10(11)15-5-1-2-9(15)7-3-4-13-12(7)14-8/h1-6,13H/b14-8+
q-4	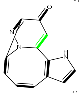 $C_{12}H_7N_3O$	6.3747	55.9861	-3.4749	151.8547	240.1564	InChI=1S/C12H7N3O/c16-10-6-9-11-7(4-5-13-11)2-1-3-8-12(10)14-15(8)9/h1-6,13H/b2-1-,8-3-
G-38	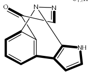 $C_{12}H_7N_3O$	4.9734	27.2877	-1.9936	175.6906	240.1849	InChI=1S/C12H7N3O/c16-10-6-14-15-9-3-1-2-7(11(9)10)8-4-5-13-12(8)15/h1-6,13H
H11-22	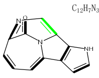 $C_{12}H_7N_3O$	5.4495	50.9886	-1.0178	164.796	240.3637	InChI=1S/C12H7N3O/c16-12-8-2-1-3-9-7-4-5-13-11(7)10(6-14-8)15(9)12/h1-6,13H
q-64	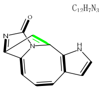 $C_{12}H_7N_3O$	4.2659	50.798	-2.6957	169.6562	240.4826	InChI=1S/C12H7N3O/c16-12-14-8-6-10-11-7(4-5-13-11)2-1-3-9(8)15(10)12/h1-6,13H/b2-1-,9-3-
o-199	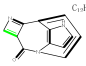 $C_{12}H_7N_3O$	3.5552	90.3155	-2.1622	139.1277	240.6488	InChI=1S/C12H7N3O/c16-12-8-6-14-10(8)7-2-1-5-15(12)9-3-4-13-11(7)9/h1-6,13H
r-8	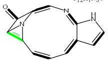 $C_{12}H_7N_3O$	19.0369	144.5411	-13.5798	66.397	240.8203	InChI=1S/C12H7N3O/c16-11-9-6-14-12-7(4-5-13-12)2-1-3-8-10(11)15(8)9/h1-6H,(H,13,14)/b3-1-,7-2-,9-6-
F-31	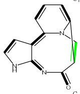 $C_{12}H_7N_3O$	6.4483	78.6891	-1.5998	132.0745	240.9216	InChI=1S/C12H7N3O/c16-12-8-6-15-9(2-1-3-10(8)15)7-4-5-13-11(7)14-12/h1-6H,(H,13,14,16)/b9-7-
q-53	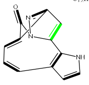 $C_{12}H_7N_3O$	3.9676	56.3652	-2.3623	163.5474	240.9891	InChI=1S/C12H7N3O/c16-12-8-3-1-2-7-4-5-13-11(7)10-6-9(8)14-15(10)12/h1-6,13H/b2-1-,8-3+
r1-5	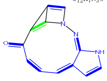 $C_{12}H_7N_3O$	23.063	146.3055	-12.5068	66.4165	241.1032	InChI=1S/C12H7N3O/c16-9-3-1-2-7-4-5-13-12(7)14-15-6-8-10(9)11(8)15/h1-6H,(H,13,14)/b3-1-,7-2-
x7-21	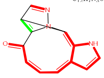 $C_{12}H_7N_3O$	5.0425	57.3162	-4.8088	160.4361	241.4273	InChI=1S/C12H7N3O/c16-9-3-1-2-7-4-5-13-10(7)12-8-6-14-15(12)11(8)9/h1-6,13H/b3-1-,7-2-,12-10-
H-50	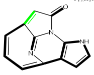 $C_{12}H_7N_3O$	3.935	103.5715	-0.8197	113.2415	241.7669	InChI=1S/C12H7N3O/c16-10-6-7-2-1-3-9-8-4-5-13-12(8)15(10)11(7)14-9/h1-6,13H
A-139	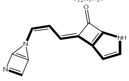 $C_{12}H_7N_3O$	19.5803	203.4857	-11.7371	16.8385	241.7915	InChI=1S/C12H7N3O/c16-11-8(7-3-4-13-10(7)11)2-1-5-15-9-6-14-12(9)15/h1-6,13H/b5-1-,8-2-

k-45	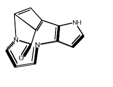	$C_{12}H_7N_3O$	4.2593	177.6282	-1.4932	64.1788	241.8893	InChI=1S/C12H7N3O/c16-12-10-7-6-9(10)15(12)5-1-3-13-8-2-4-14-11(7)8/h1-6,14H/b5-1-,13-3+
H11-13	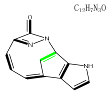	$C_{12}H_7N_3O$	4.1047	101.1007	-2.1306	127.2043	241.9395	InChI=1S/C12H7N3O/c16-12-9-3-1-2-7-6-10(15(12)14-9)11-8(7)4-5-13-11/h1-6,13H/b3-1-,7-2+
G-11	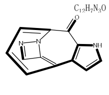	$C_{12}H_7N_3O$	3.1031	84.7821	-1.6985	133.0256	241.9454	InChI=1S/C12H7N3O/c16-12-9-3-1-2-7(10-6-14-15(9)10)8-4-5-13-11(8)12/h1-6,13H
p11-19	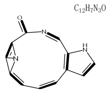	$C_{12}H_7N_3O$	18.266	151.9113	-14.6336	59.9277	242.1453	InChI=1S/C12H7N3O/c16-12-11-10-9(15(10)11)3-1-2-7-4-5-13-8(7)6-14-12/h1-6,13H/b2-1-,9-3-,14-6-
x5-40	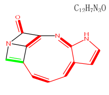	$C_{12}H_7N_3O$	4.4562	117.3397	-3.7726	118.3995	242.1633	InChI=1S/C12H7N3O/c16-12-9-10-8(6-15(10)12)3-1-2-7-4-5-13-11(7)14-9/h1-6H,(H,13,14)/b3-1-,7-2-
q11-67	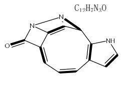	$C_{12}H_7N_3O$	4.2369	96.3689	-2.4449	128.9925	242.1651	InChI=1S/C12H7N3O/c16-12-8-3-1-2-7-4-5-13-11(7)9-6-10(8)15(12)14-9/h1-6,13H/b2-1-,8-3+
o-229	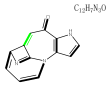	$C_{12}H_7N_3O$	13.8239	74.8653	-2.6136	118.8357	242.3582	InChI=1S/C12H7N3O/c16-10-6-7-8-2-1-5-15(12(7)14-8)9-3-4-13-11(9)10/h1-6,13H
x9-30	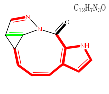	$C_{12}H_7N_3O$	13.269	134.2138	-8.222	78.6737	242.4382	InChI=1S/C12H7N3O/c16-12-10-7(4-5-13-10)2-1-3-8-9-6-14-15(12)11(8)9/h1-6,13H/b2-1-,8-3-
q11-64	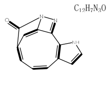	$C_{12}H_7N_3O$	6.8166	151.1206	-2.2285	68.4219	242.5161	InChI=1S/C12H7N3O/c16-12-8-3-1-2-7-4-5-13-10(7)11-9(6-8)15(12)14-11/h1-6,13H/b2-1-,8-3+
x7-33	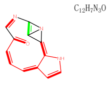	$C_{12}H_7N_3O$	18.0067	146.8751	-14.6132	64.2142	242.5671	InChI=1S/C12H7N3O/c16-8-3-1-2-7-4-5-13-9(7)10-11-12(14-6-8)15(10)11/h1-6,13H/b3-1-,7-2-,10-9-,14-6-
p1-51	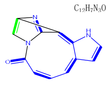	$C_{12}H_7N_3O$	7.3995	60.1603	-5.842	152.6547	242.7183	InChI=1S/C12H7N3O/c16-9-3-1-2-7-4-5-13-11(7)10-8-6-15(9)12(10)14-8/h1-6,13H/b3-1-,7-2-,11-10+
B-231	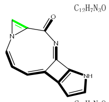	$C_{12}H_7N_3O$	15.8109	190.0125	-9.2424	18.4947	243.0287	InChI=1S/C12H7N3O/c16-12-9-6-15(9)5-1-2-7-8-3-4-13-10(8)11(7)14-12/h1-6,13H/b5-1-,7-2-,14-11+
A1-36	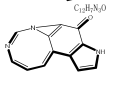	$C_{12}H_7N_3O$	7.6389	192.5339	-5.7235	25.9137	243.1508	InChI=1S/C12H7N3O/c16-12-9-7(3-5-14-9)8-2-1-4-13-6-15-10(8)11(12)15/h1-6,14H/b4-1-,8-2-,13-6-
i-25	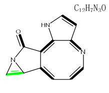	$C_{12}H_7N_3O$	24.1093	149.7976	-16.3959	74.1693	243.3247	InChI=1S/C12H7N3O/c16-12-10-7(9-6-15(9)12)2-1-4-13-8-3-5-14-11(8)10/h1-6,14H/b2-1-,4-1-,7-2-,11-10-,13-4-,13-8-
x4-44	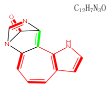	$C_{12}H_7N_3O$	4.0233	60.6437	-3.6465	164.3112	243.4323	InChI=1S/C12H7N3O/c16-12-11-9-8(15(12)6-14-11)3-1-2-7-4-5-13-10(7)9/h1-6,13H
H1-59	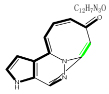	$C_{12}H_7N_3O$	5.6797	59.7057	-3.5645	153.2934	243.6019	InChI=1S/C12H7N3O/c16-7-2-1-3-9-8-4-5-13-11(8)12-10(6-7)15(9)14-12/h1-6,13H/b2-1-,9-3-,10-6-
B-132	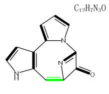	$C_{12}H_7N_3O$	5.7484	120.8495	-2.7801	91.6077	243.7016	InChI=1S/C12H7N3O/c16-11-9-6-8-7(3-4-13-8)10-2-1-5-15(10)12(11)14-9/h1-6,13H/b9-6-
C-33	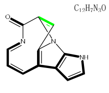	$C_{12}H_7N_3O$	8.8174	121.8181	-1.7139	91.4490	244.1836	InChI=1S/C12H7N3O/c16-12-10-6-9-7(2-1-4-14-12)8-3-5-13-11(8)15(9)10/h1-6,13H/b2-1-,14-4+
X23-39	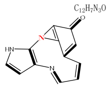	$C_{12}H_7N_3O$	12.6662	140.5264	-6.5939	74.6008	244.2204	InChI=1S/C12H7N3O/c16-9-6-7-2-1-4-13-8-3-5-14-12(8)15-10(7)11(9)15/h1-6,14H/b2-1-,13-4+

X23-45	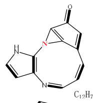 $C_{12}H_7N_3O$	12.6645	140.5257	-6.5917	74.597	244.2204	InChI=1S/C12H7N3O/c16-9-6-7-2-1-4-13-8-3-5-14-12(8)15-10(7)11(9)15/h1-6,14H/b2-1-,13-4-
G1-70	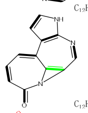 $C_{12}H_7N_3O$	22.7006	191.9953	-7.1368	22.3382	244.2428	InChI=1S/C12H7N3O/c16-10-3-1-2-7-8-4-5-13-12(8)14-6-9-11(7)15(9)10/h1-6,13H
x5-16	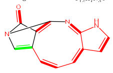 $C_{12}H_7N_3O$	9.6604	151.1014	-4.5219	81.3766	244.3857	InChI=1S/C12H7N3O/c16-12-9-8-3-1-2-7-4-5-13-10(7)14-11(9)15(12)6-8/h1-6H,(H,13,14)/b3-1+,7-2-
r1-3	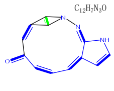 $C_{12}H_7N_3O$	23.4002	150.5176	-19.8241	49.1417	244.6427	InChI=1S/C12H7N3O/c16-8-3-1-2-7-4-5-13-12(7)14-15-10-9(6-8)11(10)15/h1-6H,(H,13,14)/b3-1-,7-2-,9-6-
o-228	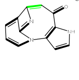 $C_{12}H_7N_3O$	10.6027	83.5608	-1.282	117.718	244.6467	InChI=1S/C12H7N3O/c16-10-6-8-7-2-1-5-15(12(7)14-8)9-3-4-13-11(9)10/h1-6,13H/b8-6-
X23-43	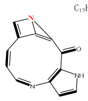 $C_{12}H_7N_3O$	9.931	199.4421	-8.3852	24.6845	244.8222	InChI=1S/C12H7N3O/c16-12-9-8(3-5-14-9)13-4-1-2-7-6-15-10(7)11(12)15/h1-6,14H/b2-1-,13-4-
n-164	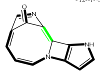 $C_{12}H_7N_3O$	5.4442	110.0688	-0.3498	112.1621	244.9565	InChI=1S/C12H7N3O/c16-12-7-2-1-5-15-8-3-4-13-9(8)11(15)10(12)14-6-7/h1-6,13H/b5-1-,7-2-,11-10+
q-62	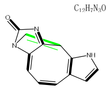 $C_{12}H_7N_3O$	3.4502	57.4982	-2.569	166.9192	245.0278	InChI=1S/C12H7N3O/c16-12-14-11-8-6-15(12)9(11)3-1-2-7-4-5-13-10(7)8/h1-6,13H/b2-1-,9-3+
C-18	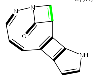 $C_{12}H_7N_3O$	4.7642	139.0906	-1.4021	98.2811	245.1282	InChI=1S/C12H7N3O/c16-12-9-6-15(12)14-4-1-2-7-8-3-5-13-11(8)10(7)9/h1-6,13H/b2-1-,14-4-
r1-8	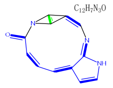 $C_{12}H_7N_3O$	42.296	163.4048	-27.7993	40.791	245.2317	InChI=1S/C12H7N3O/c16-9-3-1-2-7-4-5-13-12(7)14-6-8-10-11(8)15(9)10/h1-6H,(H,13,14)/b3-1-,7-2-,8-6-
o-109	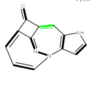 $C_{12}H_7N_3O$	8.0714	111.6225	0.0953	105.6517	245.7193	InChI=1S/C12H7N3O/c16-12-7-2-1-5-15-10-3-4-13-9(10)6-8(12)11(7)14-15/h1-6,13H
l-38	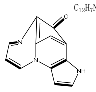 $C_{12}H_7N_3O$	3.9166	30.2351	-2.1878	185.3701	246.0198	InChI=1S/C12H7N3O/c16-12-7-6-9-11(12)13-3-1-5-15(9)8-2-4-14-10(7)8/h1-6,14H
k-34	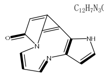 $C_{12}H_7N_3O$	13.524	131.016	-8.036	80.8169	246.2373	InChI=1S/C12H7N3O/c16-9-6-7-10-11-8(2-4-14-11)13-3-1-5-15(9)12(7)10/h1-6,14H/b5-1-,13-3+
G-57	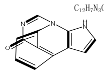 $C_{12}H_7N_3O$	5.6497	27.6794	-2.3909	179.2026	246.4004	InChI=1S/C12H7N3O/c16-12-10-7-2-1-3-9(10)15(6-14-12)11-8(7)4-5-13-11/h1-6,13H
p-22	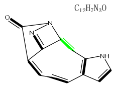 $C_{12}H_7N_3O$	7.1483	83.2387	-5.2395	146.5137	246.5805	InChI=1S/C12H7N3O/c16-12-8-3-1-2-7-4-5-13-9(7)6-10-11(8)14-15(10)12/h1-6,13H/b2-1+,8-3+,10-6-
x9-45	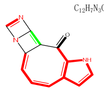 $C_{12}H_7N_3O$	5.332	98.9018	-4.6174	133.3593	246.7828	InChI=1S/C12H7N3O/c16-11-9-8(15-6-14-12(9)15)3-1-2-7-4-5-13-10(7)11/h1-6,13H/b2-1-,8-3+
x4-7	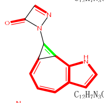 $C_{12}H_7N_3O$	13.3077	200.0333	-10.0222	29.9886	246.7987	InChI=1S/C12H7N3O/c16-9-6-14-15(9)12-8-3-1-2-7-4-5-13-11(7)10(8)12/h1-6,13H
x4-27	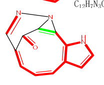 $C_{12}H_7N_3O$	9.5534	177.9296	-4.7002	41.2341	247.0075	InChI=1S/C12H7N3O/c16-12-8-3-1-2-7-4-5-13-9(7)10-11(12)15(10)14-6-8/h1-6,13H/b2-1-,8-3-
r1-36	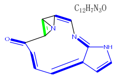 $C_{12}H_7N_3O$	17.9625	166.7592	-15.3786	49.6958	247.1399	InChI=1S/C12H7N3O/c16-9-3-1-2-7-4-5-13-12(7)14-6-8-10-11(9)15(8)10/h1-6H,(H,13,14)/b3-1+,7-2-,8-6-

r1-2	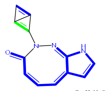 $C_{12}H_7N_3O$	16.8995	196.3954	-20.94	46.0335	247.1777	InChI=1S/C12H7N3O/c16-10-3-1-2-7-4-5-13-12(7)14-15(10)11-8-6-9(8)11/h1-6H,(H,13,14)/b3-1-,7-2-
x4-67	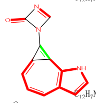 $C_{12}H_7N_3O$	13.1097	202.0803	-9.8911	30.0306	247.2342	InChI=1S/C12H7N3O/c16-12-14-6-15(12)11-8-3-1-2-7-4-5-13-10(7)9(8)11/h1-6,13H
C-7	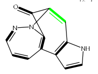 $C_{12}H_7N_3O$	5.1063	54.8837	-2.8419	160.9390	247.3043	InChI=1S/C12H7N3O/c16-12-10-6-9-7(3-5-13-9)8-2-1-4-14-15(10)11(8)12/h1-6,13H
A-71	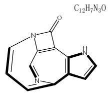 $C_{12}H_7N_3O$	7.2056	100.2634	-2.4286	125.8972	247.5054	InChI=1S/C12H7N3O/c16-12-10-9-6-14-8(2-1-5-15(9)12)7-3-4-13-11(7)10/h1-6,13H/b5-1-,8-2-
C-12	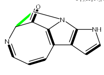 $C_{12}H_7N_3O$	9.7206	141.5892	0.6069	64.8715	247.5450	InChI=1S/C12H7N3O/c16-11-9-6-15-10(11)7(2-1-4-13-9)8-3-5-14-12(8)15/h1-6,14H/b2-1-,13-4-
G1-89	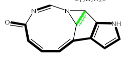 $C_{12}H_7N_3O$	14.5711	147.5792	-6.6587	64.6324	247.7116	InChI=1S/C12H7N3O/c16-9-3-1-2-8-7-4-5-13-10(7)12-11(8)15(12)6-14-9/h1-6,13H/b3-1-,8-2-,14-6-
r-56	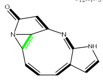 $C_{12}H_7N_3O$	14.1727	174.0853	-8.2223	47.5102	247.8864	InChI=1S/C12H7N3O/c16-10-6-8-11-9(15(10)11)3-1-2-7-4-5-13-12(7)14-8/h1-6H,(H,13,14)/b3-1-,7-2-
A2-47	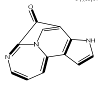 $C_{12}H_7N_3O$	3.4687	27.7277	-1.898	191.2126	247.8985	InChI=1S/C12H7N3O/c16-11-8-6-15-9(2-1-4-14-12(11)15)7-3-5-13-10(7)8/h1-6,13H
E-49	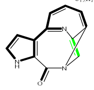 $C_{12}H_7N_3O$	6.8805	76.3303	-2.9643	149.0215	248.0216	InChI=1S/C12H7N3O/c16-12-11-7(4-5-13-11)8-2-1-3-10-9(14-8)6-15(10)12/h1-6,13H
p-23	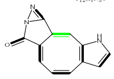 $C_{12}H_7N_3O$	18.3878	192.7632	-4.4081	34.9412	248.331	InChI=1S/C12H7N3O/c16-12-8-3-1-2-7-4-5-13-10(7)6-9(8)11-14-15(11)12/h1-6,13H/b2-1-,3-1-,7-2-,8-3+,9-6+,10-6+
x4-26	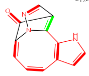 $C_{12}H_7N_3O$	5.4209	39.1241	-3.3105	173.9208	248.3489	InChI=1S/C12H7N3O/c16-12-8-6-14-15-9(12)3-1-2-7-4-5-13-10(7)11(8)15/h1-6,13H/b2-1-,9-3-
X22-19	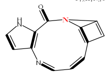 $C_{12}H_7N_3O$	21.8182	145.537	-12.1232	86.8778	248.4024	InChI=1S/C12H7N3O/c16-12-10-8(3-5-14-10)13-4-1-2-7-6-9-11(7)15(9)12/h1-6,14H/b2-1-,13-4+
p-18	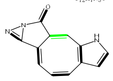 $C_{12}H_7N_3O$	18.3809	192.9279	-4.414	34.9543	248.5777	InChI=1S/C12H7N3O/c16-12-9-6-10-7(4-5-13-10)2-1-3-8(9)11-14-15(11)12/h1-6,13H/b2-1-,3-1-,7-2-,8-3+,9-6+,10-6+
x5-29	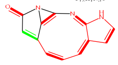 $C_{12}H_7N_3O$	13.1789	155.2125	-7.5702	71.0744	248.6186	InChI=1S/C12H7N3O/c16-9-6-8-3-1-2-7-4-5-13-11(7)14-12-10(8)15(9)12/h1-6H,(H,13,14)/b3-1-,7-2-
H1-76	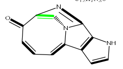 $C_{12}H_7N_3O$	4.9197	107.4836	-0.0935	111.886	248.9923	InChI=1S/C12H7N3O/c16-10-3-1-2-9-7-4-5-13-11(7)12-14-8(10)6-15(9)12/h1-6,13H/b3-1-,9-2-
X23-40	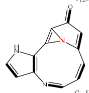 $C_{12}H_7N_3O$	24.7121	139.5452	-12.0807	67.0138	249.073	InChI=1S/C12H7N3O/c16-9-6-7-2-1-4-13-8-3-5-14-10(8)12-11(9)15(7)12/h1-6,14H/b2-1-,13-4+
p11-2	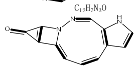 $C_{12}H_7N_3O$	42.8472	204.8605	-19.4144	12.5587	249.3673	InChI=1S/C12H7N3O/c16-12-10-9-3-1-2-7-4-5-13-8(7)6-14-15(9)11(10)12/h1-6,13H/b2-1-,9-3-,14-6-
F-50	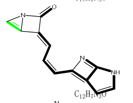 $C_{12}H_7N_3O$	23.0936	225.5882	-16.0852	15.5754	249.486	InChI=1S/C12H7N3O/c16-12-8(10-6-15(10)12)2-1-3-9-7-4-5-13-11(7)14-9/h1-6H,(H,13,14)/b3-1-,8-2+
p11-4	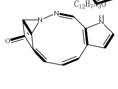 $C_{12}H_7N_3O$	12.1768	162.1413	-8.5657	60.3536	249.4895	InChI=1S/C12H7N3O/c16-12-8-3-1-2-7-4-5-13-9(7)6-14-15-10(8)11(12)15/h1-6,13H/b2-1-,8-3+,14-6-

H11-101	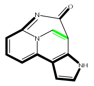 $C_{12}H_7N_3O$	4.0329	26.8049	-1.2942	189.3571	249.5407	InChI=1S/C12H7N3O/c16-12-8-6-15-9(2-1-3-10(15)14-12)7-4-5-13-11(7)8/h1-6,13H
x7-29	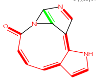 $C_{12}H_7N_3O$	23.8673	165.0777	-8.2491	48.499	249.5926	InChI=1S/C12H7N3O/c16-9-3-1-2-7-4-5-13-10(7)8-6-14-12-11(8)15(9)12/h1-6,13H/b3-1-,7-2-,10-8+
r-18	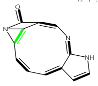 $C_{12}H_7N_3O$	22.2436	165.0768	-12.336	53.6117	249.7376	InChI=1S/C12H7N3O/c16-12-8-6-14-11-7(4-5-13-11)2-1-3-9-10(8)15(9)12/h1-6H,(H,13,14)
C-58	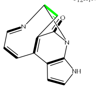 $C_{12}H_7N_3O$	5.3603	41.4412	-2.1247	178.0747	250.1303	InChI=1S/C12H7N3O/c16-12-9-6-10-13-4-1-2-7(9)8-3-5-14-11(8)15(10)12/h1-6,14H/b2-1-,4-1-,7-2-,13-4+,13-10+
o-235	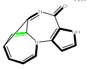 $C_{12}H_7N_3O$	5.21	51.484	-2.9099	166.0265	250.1985	InChI=1S/C12H7N3O/c16-12-11-8(3-4-13-11)15-5-1-2-7-6-9(15)10(7)14-12/h1-6,13H
x7-13	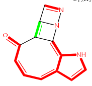 $C_{12}H_7N_3O$	15.8869	187.2353	-2.6093	37.7076	250.2268	InChI=1S/C12H7N3O/c16-9-3-1-2-7-4-5-13-11(7)12-10(9)8-6-14-15(8)12/h1-6,13H/b3-1-,7-2-,12-11-
i-17	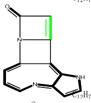 $C_{12}H_7N_3O$	21.8277	194.4602	-2.7117	41.8852	250.3106	InChI=1S/C12H7N3O/c16-10-6-9-11-8(15(9)10)2-1-4-13-7-3-5-14-12(7)11/h1-6,14H/b2-1-,4-1-,8-2+,12-11-,13-4+,13-7+
q11-34	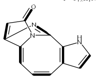 $C_{12}H_7N_3O$	5.9552	60.2068	-2.4695	165.1718	250.4044	InChI=1S/C12H7N3O/c16-10-6-8-9-3-1-2-7-4-5-13-11(7)12(14-8)15(9)10/h1-6,13H/b2-1-,9-3-
B-104	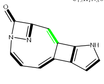 $C_{12}H_7N_3O$	5.6802	142.2327	-1.7178	96.5892	250.4420	InChI=1S/C12H7N3O/c16-12-10-6-9-7(2-1-5-15(12)14-10)8-3-4-13-11(8)9/h1-6,13H/b5-1-,7-2-,9-6-
H1-24	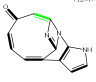 $C_{12}H_7N_3O$	9.9967	132.6683	-1.2407	92.4927	250.4728	InChI=1S/C12H7N3O/c16-7-2-1-3-8-9-4-5-13-11(9)15-10(6-7)14-12(8)15/h1-6,13H/b2-1-,8-3-,10-6+
q11-51	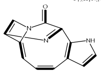 $C_{12}H_7N_3O$	5.9818	64.9528	-3.7205	157.5638	250.6523	InChI=1S/C12H7N3O/c16-12-11-10-7(4-5-13-10)2-1-3-9-8(14-11)6-15(9)12/h1-6,13H/b2-1-,9-3-
x7-45	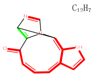 $C_{12}H_7N_3O$	5.5433	57.8411	-4.9641	171.6764	250.75	InChI=1S/C12H7N3O/c16-8-3-1-2-7-4-5-13-9(7)12-10-11(8)15(12)6-14-10/h1-6,13H/b3-1-,7-2-,12-9-
q-3	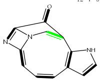 $C_{12}H_7N_3O$	4.8193	82.8594	-1.8844	145.8899	250.8262	InChI=1S/C12H7N3O/c16-11-8-6-15-9(14-12(11)15)3-1-2-7-4-5-13-10(7)8/h1-6,13H/b2-1-,9-3-
r-1	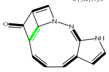 $C_{12}H_7N_3O$	7.0467	87.4517	-5.6003	147.4985	251.1746	InChI=1S/C12H7N3O/c16-11-8-3-1-2-7-4-5-13-12(7)14-15-6-9(11)10(8)15/h1-6H,(H,13,14)/b3-1-,7-2-
E-69	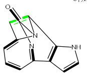 $C_{12}H_7N_3O$	3.3161	66.7527	-1.8883	171.0041	251.2007	InChI=1S/C12H7N3O/c16-12-14-9-3-1-2-7-6-10(15(7)12)11-8(9)4-5-13-11/h1-6,13H/b3-1-,7-2-
q-63	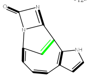 $C_{12}H_7N_3O$	3.8657	76.8125	-2.6842	167.1431	251.3735	InChI=1S/C12H7N3O/c16-12-14-11-9-6-8(15(11)12)3-1-2-7-4-5-13-10(7)9/h1-6,13H/b2-1-,3-1-,7-2-,8-3+,10-9-
l-97	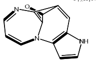 $C_{12}H_7N_3O$	4.4035	56.9392	-2.0406	164.5005	251.3736	InChI=1S/C12H7N3O/c16-12-7-6-8-9(2-4-13-8)15-5-1-3-14-10(7)11(12)15/h1-6,13H
n-181	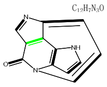 $C_{12}H_7N_3O$	7.2394	117.6548	-1.1854	113.5702	251.6484	InChI=1S/C12H7N3O/c16-12-7-6-14-8-2-1-5-15(12)9-3-4-13-11(9)10(7)8/h1-6,13H/b5-1-,8-2+
o-174	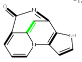 $C_{12}H_7N_3O$	3.7657	31.5544	-0.7364	186.2093	251.7181	InChI=1S/C12H7N3O/c16-12-7-2-1-5-15-9-3-4-13-11(9)8(14-12)6-10(7)15/h1-6,13H

x4-5	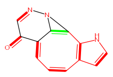	$C_{12}H_7N_3O$	8.0608	149.9157	-6.0502	69.3581	252.082	InChI=1S/C12H7N3O/c16-9-6-14-15-11-8(9)3-1-2-7-4-5-13-10(7)12(11)15/h1-6,13H/b2-1-,3-1-,7-2-,8-3-,12-10-
n-184	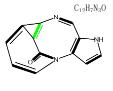	$C_{12}H_7N_3O$	7.0032	185.1097	-6.1428	52.9697	252.1672	InChI=1S/C12H7N3O/c16-12-10-7-2-1-5-15(12)9-3-4-13-8(9)6-14-11(7)10/h1-6,13H/b14-6-
X22-56	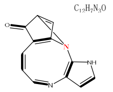	$C_{12}H_7N_3O$	8.5409	70.4234	-5.9156	153.3137	252.1868	InChI=1S/C12H7N3O/c16-11-7-2-1-4-13-8-3-5-14-12(8)15-9(7)6-10(11)15/h1-6,14H/b2-1-,13-4-
A1-14	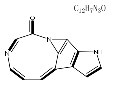	$C_{12}H_7N_3O$	23.8825	187.8476	-7.7691	28.1988	252.2367	InChI=1S/C12H7N3O/c16-9-6-13-4-1-2-8-7-3-5-14-10(7)12-11(8)15(9)12/h1-6,14H/b4-1-,8-2-,13-6-
q1-15	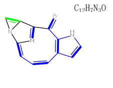	$C_{12}H_7N_3O$	19.1588	138.1681	-13.1283	83.3674	252.2892	InChI=1S/C12H7N3O/c16-12-10-7(4-5-13-10)2-1-3-9-14-11(12)8-6-15(8)9/h1-6,13H/b2-1-,9-3+
i-26	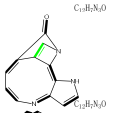	$C_{12}H_7N_3O$	5.7249	73.7027	-5.0045	171.5353	252.3243	InChI=1S/C12H7N3O/c16-12-7-2-1-4-13-9-3-5-14-10(9)11-8(7)6-15(11)12/h1-6,14H/b4-1-,7-2-,11-10-,13-9+
n-193	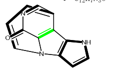	$C_{12}H_7N_3O$	6.8514	44.5674	-1.9978	165.742	252.4068	InChI=1S/C12H7N3O/c16-12-11-9-7(6-14-12)2-1-5-15(11)8-3-4-13-10(8)9/h1-6,13H
x4-21	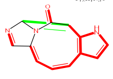	$C_{12}H_7N_3O$	9.9261	101.8665	-2.1327	132.8988	252.4378	InChI=1S/C12H7N3O/c16-12-9-10-7(4-5-13-10)2-1-3-8-6-14-11(9)15(8)12/h1-6,13H/b2-1-,3-1-,7-2?,8-3-,10-9-
X23-38	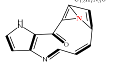	$C_{12}H_7N_3O$	14.2054	177.3543	-11.6833	45.1948	252.7335	InChI=1S/C12H7N3O/c16-12-10-8(3-5-14-10)13-4-1-2-7-6-9-11(12)15(7)9/h1-6,14H/b2-1-,13-4+
x6-25	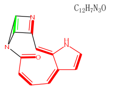	$C_{12}H_7N_3O$	26.4263	162.6429	-13.7198	64.5062	252.8078	InChI=1S/C12H7N3O/c16-10-3-1-2-7-4-5-13-8(7)6-9-11-12(14-9)15(10)11/h1-6,13H/b3-1-,7-2-,8-6-
B-17	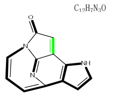	$C_{12}H_7N_3O$	4.2555	111.9752	-0.6511	112.7018	253.0623	InChI=1S/C12H7N3O/c16-10-6-8-11-7(3-4-13-11)9-2-1-5-15(10)12(8)14-9/h1-6,13H
p1-4	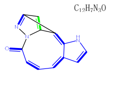	$C_{12}H_7N_3O$	6.925	49.039	-6.2115	177.8933	253.1348	InChI=1S/C12H7N3O/c16-10-3-1-2-7-4-5-13-12(7)11-8-6-9(11)15(10)14-8/h1-6,13H/b3-1-,7-2-,12-11+
k-35	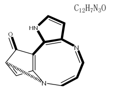	$C_{12}H_7N_3O$	5.2902	53.5092	-4.9584	182.6693	253.157	InChI=1S/C12H7N3O/c16-12-9-6-8-10(12)11-7(2-4-14-11)13-3-1-5-15(8)9/h1-6,14H/b5-1-,13-3-
C-31	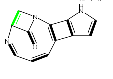	$C_{12}H_7N_3O$	4.5094	152.1671	-1.1217	91.7676	253.4230	InChI=1S/C12H7N3O/c16-12-9-6-15(12)11-8(2-1-4-13-9)7-3-5-14-10(7)11/h1-6,14H/b2-1-,13-4-
X22-26	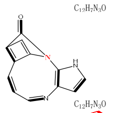	$C_{12}H_7N_3O$	5.8647	44.0112	-5.9157	193.9551	253.4457	InChI=1S/C12H7N3O/c16-12-8-6-10-7(8)2-1-4-13-9-3-5-14-11(9)15(10)12/h1-6,14H/b2-1-,13-4-
x3-1	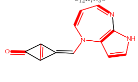	$C_{12}H_7N_3O$	48.7512	192.1269	-28.4528	17.1455	253.4581	InChI=1S/C12H7N3O/c16-11-9-7(10(9)11)6-15-5-1-3-13-12-8(15)2-4-14-12/h1-6,14H
H1-10	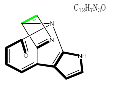	$C_{12}H_7N_3O$	4.6118	153.7749	-2.0879	92.8563	253.4924	InChI=1S/C12H7N3O/c16-10-3-1-2-7-8-4-5-13-12(8)15(10)9-6-14-11(7)9/h1-6,13H/b3-1-,7-2-
o-48	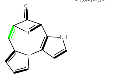	$C_{12}H_7N_3O$	6.0904	119.4549	-2.3993	100.366	253.5482	InChI=1S/C12H7N3O/c16-12-8-6-7-2-1-5-15(7)9-3-4-13-10(9)11(12)14-8/h1-6,13H/b8-6-
H1-114	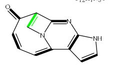	$C_{12}H_7N_3O$	8.0294	68.666	-2.9376	150.7624	253.7246	InChI=1S/C12H7N3O/c16-10-3-1-2-9-7-4-5-13-11(7)14-12-8(10)6-15(9)12/h1-6,13H/b3-1-,9-2-

n-45	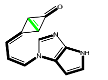 $C_{12}H_7N_3O$	48.0069	205.9024	-28.6904	14.2004	253.9365	InChI=1S/C12H7N3O/c16-11-9-7(10(9)11)2-1-5-15-6-14-12-8(15)3-4-13-12/h1-6,13H/b5-1-
B-240	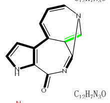 $C_{12}H_7N_3O$	4.6792	46.2645	-3.0613	175.6048	254.0438	InChI=1S/C12H7N3O/c16-12-10-8(3-4-13-10)7-2-1-5-15-6-9(7)11(15)14-12/h1-6,13H
x6-9	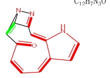 $C_{12}H_7N_3O$	11.5288	167.7379	-9.8669	60.3518	254.0903	InChI=1S/C12H7N3O/c16-10-3-1-2-7-4-5-13-8(7)6-9-11-12(10)15(11)14-9/h1-6,13H/b3-1-,7-2-,8-6-
H1-92	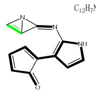 $C_{12}H_7N_3O$	20.5394	223.2765	-16.7461	19.5878	254.0922	InChI=1S/C12H7N3O/c16-10-3-1-2-7(10)8-4-5-13-11(8)14-12-9-6-15(9)12/h1-6,13H/b14-12+
s12-27	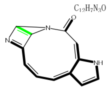 $C_{12}H_7N_3O$	29.0318	172.0983	-14.2302	54.3891	254.2569	InChI=1S/C12H7N3O/c16-10-6-9-7(4-5-13-9)2-1-3-8-11-12(14-8)15(10)11/h1-6,13H/b3-1-,7-2-,9-6+
E-68	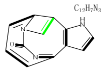 $C_{12}H_7N_3O$	4.947	72.2397	-2.0556	164.0325	254.6338	InChI=1S/C12H7N3O/c16-12-14-9-2-1-3-10-8(6-15(10)12)11-7(9)4-5-13-11/h1-6,13H/b2-1-,10-3-,14-9-
H-21	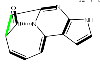 $C_{12}H_7N_3O$	6.9076	118.9248	-0.9475	107.5276	254.8234	InChI=1S/C12H7N3O/c16-12-7-2-1-3-9-8-4-5-13-11(8)14-10(6-7)15(9)12/h1-6H,(H,13,14)
H-156	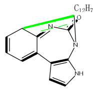 $C_{12}H_7N_3O$	5.9188	100.8961	-2.1218	108.01	254.8435	InChI=1S/C12H7N3O/c16-12-14-10-7-2-1-3-8(10)9-4-5-13-11(9)15(12)6-7/h1-6,13H
X22-25	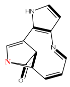 $C_{12}H_7N_3O$	13.0892	146.603	-8.229	88.2361	254.9007	InChI=1S/C12H7N3O/c16-12-10-7-6-15(12)9(10)2-1-4-13-8-3-5-14-11(7)8/h1-6,14H/b2-1-,13-4+
i-29	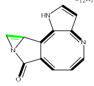 $C_{12}H_7N_3O$	24.6233	161.0985	-14.1982	57.5133	254.9335	InChI=1S/C12H7N3O/c16-12-7-2-1-4-13-8-3-5-14-11(8)10(7)9-6-15(9)12/h1-6,14H/b2-1-,4-1-,7-2+,11-10-,13-4-,13-8-
I1-43	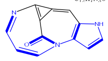 $C_{12}H_7N_3O$	6.5888	190.3462	-5.8819	52.3769	255.1843	InChI=1S/C12H7N3O/c16-12-10-7-6-8-9(2-4-13-8)15(12)5-1-3-14-11(7)10/h1-6,13H/b5-1-,14-3-
H-154	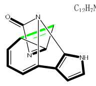 $C_{12}H_7N_3O$	16.2898	200.6342	-10.7414	33.6743	255.4128	InChI=1S/C12H7N3O/c16-12-14-9-6-7(9)2-1-3-10-8-4-5-13-11(8)15(10)12/h1-6,13H/b2-1-,10-3-,14-9-
x6-23	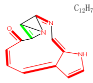 $C_{12}H_7N_3O$	17.8619	161.4558	-15.1498	57.4501	255.4637	InChI=1S/C12H7N3O/c16-9-3-1-2-7-4-5-13-8(7)6-14-12-11-10(9)15(11)12/h1-6,13H/b3-1-,7-2-,8-6-,14-12-
o-238	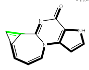 $C_{12}H_7N_3O$	12.9095	185.9725	-7.7885	33.5751	255.5117	InChI=1S/C12H7N3O/c16-12-10-9(3-4-13-10)15-5-1-2-7-6-8(7)11(15)14-12/h1-6,13H
I-92	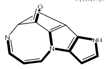 $C_{12}H_7N_3O$	9.9306	139.8422	0.4315	71.8231	256.0786	InChI=1S/C12H7N3O/c16-12-8-6-7-10-9(2-4-14-10)15(11(7)12)5-1-3-13-8/h1-6,14H/b5-1-,13-3-
o-161	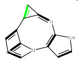 $C_{12}H_7N_3O$	20.458	147.6474	-14.1842	76.9119	256.3173	InChI=1S/C12H7N3O/c16-12-7-2-1-5-15(12)10-3-4-13-11(10)14-9-6-8(7)9/h1-6,13H/b14-9-
X23-35	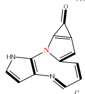 $C_{12}H_7N_3O$	30.1852	215.028	-12.0192	17.1317	256.5074	InChI=1S/C12H7N3O/c16-11-8-6-7-2-1-4-13-9-3-5-14-12(9)15(7)10(8)11/h1-6,14H/b2-1-,13-4+
q-51	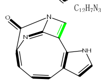 $C_{12}H_7N_3O$	4.5349	127.3422	-1.1165	116.5954	256.5454	InChI=1S/C12H7N3O/c16-12-9-3-1-2-7-4-5-13-10(7)8-6-15(12)11(8)14-9/h1-6,13H/b2-1-,9-3-
n-179	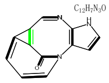 $C_{12}H_7N_3O$	7.5424	188.5377	-6.3193	52.1952	257.1546	InChI=1S/C12H7N3O/c16-12-10-7-2-1-5-15(12)9-3-4-13-11(9)14-6-8(7)10/h1-6,13H/b14-6-

k-44	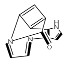 $C_{12}H_7N_3O$	8.3404	75.7174	-6.0544	153.939	257.2	InChI=1S/C12H7N3O/c16-12-7-6-9-10(7)11-8(2-4-14-11)13-3-1-5-15(9)12/h1-6,14H/b5-1-,13-3+
X22-36	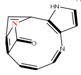 $C_{12}H_7N_3O$	5.7993	125.0833	-4.5824	117.772	257.2844	InChI=1S/C12H7N3O/c16-12-7-2-1-4-13-8-3-5-14-11(8)10-6-9(7)15(10)12/h1-6,14H/b2-1-,4-1-,7-2-,11-10+,13-4-,13-8-
H1-104	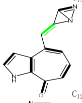 $C_{12}H_7N_3O$	25.5643	130.7463	-18.955	86.2928	257.2864	InChI=1S/C12H7N3O/c16-10-3-1-2-7(6-9-12-14-15(9)12)8-4-5-13-11(8)10/h1-6,13H/b9-6-
H1-91	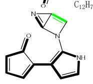 $C_{12}H_7N_3O$	20.656	140.3052	-10.9525	101.7782	257.3211	InChI=1S/C12H7N3O/c16-10-3-1-2-7(10)8-4-5-13-11(8)15-6-9-12(15)14-9/h1-6,13H
o-46	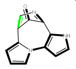 $C_{12}H_7N_3O$	5.0864	97.5237	-1.1765	133.7896	257.4603	InChI=1S/C12H7N3O/c16-12-7-6-14-11(12)10-9(3-4-13-10)15-5-1-2-8(7)15/h1-6,13H
o-236	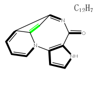 $C_{12}H_7N_3O$	12.759	83.607	-0.969	117.6476	257.5933	InChI=1S/C12H7N3O/c16-12-11-9(3-4-13-11)15-5-1-2-7-8(14-12)6-10(7)15/h1-6,13H/b14-8+
q1-41	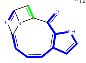 $C_{12}H_7N_3O$	5.6011	92.2448	-4.349	150.0649	257.6397	InChI=1S/C12H7N3O/c16-12-8-6-10-14-9(15(8)10)3-1-2-7-4-5-13-11(7)12/h1-6,13H/b2-1-,9-3-
q-44	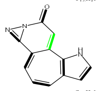 $C_{12}H_7N_3O$	17.6711	199.3149	-2.1887	34.9525	257.8894	InChI=1S/C12H7N3O/c16-10-6-9-8(12-14-15(10)12)3-1-2-7-4-5-13-11(7)9/h1-6,13H
l1-33	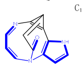 $C_{12}H_7N_3O$	27.4289	142.9561	-11.8093	61.8563	257.954	InChI=1S/C12H7N3O/c16-9-6-7-10-11(7)13-3-1-5-15(9)8-2-4-14-12(8)10/h1-6,14H/b5-1-,13-3-
q11-6	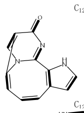 $C_{12}H_7N_3O$	6.7384	50.826	-4.5119	171.2158	258.241	InChI=1S/C12H7N3O/c16-12-8-6-15-9(8)3-1-2-7-4-5-13-10(7)11(15)14-12/h1-6,13H/b2-1-,9-3-
k-29	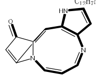 $C_{12}H_7N_3O$	5.2626	55.3263	-4.8876	188.4584	258.3039	InChI=1S/C12H7N3O/c16-9-6-8-10-11-7(2-4-14-11)13-3-1-5-15(8)12(9)10/h1-6,14H/b5-1-,13-3-
s11-49	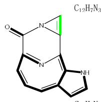 $C_{12}H_7N_3O$	27.0652	161.0275	-15.9562	64.5644	258.3346	InChI=1S/C12H7N3O/c16-12-8-3-1-2-7-4-5-13-10(7)11(14-8)9-6-15(9)12/h1-6,13H/b2-1-,3-1-,7-2-,8-3-,11-10+
o-182	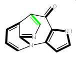 $C_{12}H_7N_3O$	6.9611	43.412	-1.7331	173.4356	258.5233	InChI=1S/C12H7N3O/c16-11-8-6-14-12-7(8)2-1-5-15(12)9-3-4-13-10(9)11/h1-6,13H
G1-105	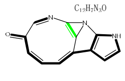 $C_{12}H_7N_3O$	12.5475	144.813	-7.4628	82.0408	258.6533	InChI=1S/C12H7N3O/c16-7-2-1-3-8-9-4-5-13-11(9)15-10(8)12(15)14-6-7/h1-6,13H/b2-1-,8-3-,14-6-
A2-72	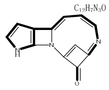 $C_{12}H_7N_3O$	6.6669	146.1773	-1.6266	92.9011	258.6606	InChI=1S/C12H7N3O/c16-11-8-6-10(11)15-9(2-1-4-13-8)7-3-5-14-12(7)15/h1-6,14H/b4-1-,9-2-,13-8+
F-17	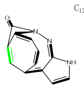 $C_{12}H_7N_3O$	6.9231	101.0194	-2.8697	134.1431	258.8913	InChI=1S/C12H7N3O/c16-12-9-6-7-2-1-3-10(9)15(12)14-11-8(7)4-5-13-11/h1-6H,(H,13,14)/b8-7+
X23-19	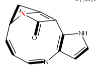 $C_{12}H_7N_3O$	5.3187	152.751	-2.6261	93.7299	259.0993	InChI=1S/C12H7N3O/c16-12-10-9-6-7(15(9)12)2-1-4-13-8-3-5-14-11(8)10/h1-6,14H/b2-1-,4-1-,7-2-,11-10+,13-4-,13-8-
n-34	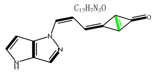 $C_{12}H_7N_3O$	48.4137	209.511	-28.6597	14.1045	259.3571	InChI=1S/C12H7N3O/c16-12-10-7(11(10)12)2-1-5-15-9-3-4-13-8(9)6-14-15/h1-6,13H/b5-1-
p1-15	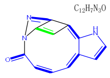 $C_{12}H_7N_3O$	10.0152	159.036	-3.4143	75.0497	259.4243	InChI=1S/C12H7N3O/c16-10-3-1-2-7-4-5-13-11(7)8-6-9-12(8)14-15(9)10/h1-6,13H/b3-1-,7-2-,11-8-

o-191	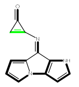 $C_{12}H_7N_3O$	38.1671	212.3142	-17.1727	16.2177	259.513	InChI=1S/C12H7N3O/c16-10-6-7(10)14-12-8-2-1-5-15(8)9-3-4-13-11(9)12/h1-6,13H/b14-12+
H11-79	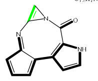 $C_{12}H_7N_3O$	34.8458	195.5731	-19.0718	29.2528	259.6126	InChI=1S/C12H7N3O/c16-12-11-8(4-5-13-11)7-2-1-3-9(7)14-10-6-15(10)12/h1-6,13H/b14-9-
H-158	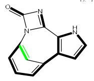 $C_{12}H_7N_3O$	8.1429	144.3713	-4.3734	90.116	259.8753	InChI=1S/C12H7N3O/c16-12-14-11-10-9(4-5-13-10)7-2-1-3-8(6-7)15(11)12/h1-6,13H
q1-10	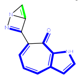 $C_{12}H_7N_3O$	13.2267	201.5409	-10.7817	38.7069	259.9083	InChI=1S/C12H7N3O/c16-12-8(11-9-6-15(9)14-11)3-1-2-7-4-5-13-10(7)12/h1-6,13H
n-197	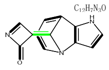 $C_{12}H_7N_3O$	4.5189	88.5521	-3.167	148.4849	259.931	InChI=1S/C12H7N3O/c16-12-8(6-14-12)11-7-2-1-5-15(11)9-3-4-13-10(7)9/h1-6,13H/b11-8-
I-24	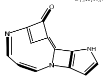 $C_{12}H_7N_3O$	6.1072	144.9669	-1.4745	93.8383	260.203	InChI=1S/C12H7N3O/c16-12-7-6-8(12)13-3-1-5-15-9-2-4-14-10(9)11(7)15/h1-6,14H/b5-1-,11-7+,13-3-
q11-47	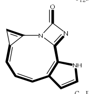 $C_{12}H_7N_3O$	24.1679	185.7488	-17.2976	54.5068	260.257	InChI=1S/C12H7N3O/c16-12-14-11-10-7(4-5-13-10)2-1-3-8-6-9(8)15(11)12/h1-6,13H/b2-1-,8-3-
H1-79	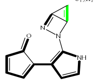 $C_{12}H_7N_3O$	13.901	230.7199	-13.1747	26.1209	260.404	InChI=1S/C12H7N3O/c16-11-3-1-2-7(11)8-4-5-13-12(8)15-10-6-9(10)14-15/h1-6,13H
A-25	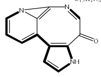 $C_{12}H_7N_3O$	5.902	196.5552	-3.4926	39.3143	260.5356	InChI=1S/C12H7N3O/c16-9-6-14-12-11-8(2-1-5-15(11)12)7-3-4-13-10(7)9/h1-6,13H/b14-6-
G1-92	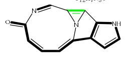 $C_{12}H_7N_3O$	12.9713	200.6004	-7.4304	28.9174	260.6451	InChI=1S/C12H7N3O/c16-10-3-1-2-8-7-4-5-13-11(7)12-9(6-14-10)15(8)12/h1-6,13H/b3-1-,8-2-,14-6-
p-26	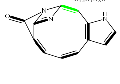 $C_{12}H_7N_3O$	8.8996	180.233	-1.4115	62.443	260.8044	InChI=1S/C12H7N3O/c16-12-8-3-1-2-7-4-5-13-9(7)6-10-14-11(8)15(10)12/h1-6,13H/b2-1-,3-1-,7-2-,8-3+,9-6+,10-6+
q11-8	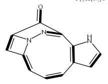 $C_{12}H_7N_3O$	7.3917	84.0495	-2.5999	151.7288	260.8485	InChI=1S/C12H7N3O/c16-12-9-6-8-3-1-2-7-4-5-13-10(7)11(12)14-15(8)9/h1-6,13H/b2-1-,8-3-
X22-1	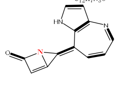 $C_{12}H_7N_3O$	19.9243	196.3831	-11.4111	56.1196	260.9643	InChI=1S/C12H7N3O/c16-10-6-9-12(15(9)10)7-2-1-4-13-8-3-5-14-11(7)8/h1-6,14H/b12-7-
I-95	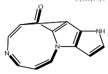 $C_{12}H_7N_3O$	10.2578	131.5551	-1.3562	93.7602	261.0058	InChI=1S/C12H7N3O/c16-12-7-6-13-3-1-5-15-8-2-4-14-10(8)9(7)11(12)15/h1-6,14H/b5-1-,7-6+,13-3-
A1-5	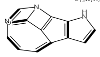 $C_{12}H_7N_3O$	8.253	134.7216	-0.6129	99.4584	261.0967	InChI=1S/C12H7N3O/c16-12-9-7-2-1-4-13-6-15(12)11(9)10-8(7)3-5-14-10/h1-6,14H/b4-1-,7-2-,13-6-
H-124	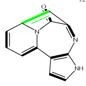 $C_{12}H_7N_3O$	6.4236	108.1481	-0.8204	119.6288	261.2754	InChI=1S/C12H7N3O/c16-12-9-6-7-2-1-3-10(15(7)12)8-4-5-13-11(8)14-9/h1-6H,(H,13,14)
D-7	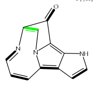 $C_{12}H_7N_3O$	4.6561	109.5842	-0.2227	125.2347	261.3295	InChI=1S/C12H7N3O/c16-12-8-6-15-9(2-1-4-13-8)7-3-5-14-10(7)11(12)15/h1-6,14H/b2-1-,13-4+
H1-47	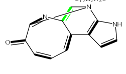 $C_{12}H_7N_3O$	4.7989	32.0724	-2.0761	195.4624	261.7746	InChI=1S/C12H7N3O/c16-10-3-1-2-7-8-4-5-13-11(8)15-6-9(7)14-12(10)15/h1-6,13H/b3-1-,7-2-
H1-41	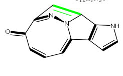 $C_{12}H_7N_3O$	10.7754	131.1185	0.5318	90.9029	262.0923	InChI=1S/C12H7N3O/c16-11-3-1-2-9-7-4-5-13-12(7)10-6-8(11)14-15(9)10/h1-6,13H/b3-1-,9-2-

I1-37	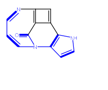 C12H7N3O	4.6245	156.2567	-1.1982	81.6515	262.1231	InChI=1S/C12H7N3O/c16-12-10-7-6-8(10)13-3-1-5-15(12)9-2-4-14-11(7)9/h1-6,14H/b5-1-,13-3-
p1-18	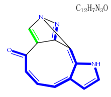 C12H7N3O	6.6031	57.268	-4.9208	176.3891	262.3674	InChI=1S/C12H7N3O/c16-9-3-1-2-7-4-5-13-10(7)12-11-8(9)6-15(12)14-11/h1-6,13H/b3-1-,7-2-,12-10-
E-10	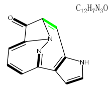 C12H7N3O	5.2814	92.9363	-1.317	139.6695	262.4445	InChI=1S/C12H7N3O/c16-12-10-3-1-2-8-7-4-5-13-9(7)6-11(12)15(10)14-8/h1-6,13H
D-17	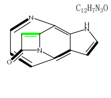 C12H7N3O	6.8018	135.1242	-1.3988	108.6573	262.5971	InChI=1S/C12H7N3O/c16-10-6-9-12-11-7(3-5-14-11)8(15(9)10)2-1-4-13-12/h1-6,14H/b2-1-,4-1-,8-2-,13-4-,13-12+
H1-89	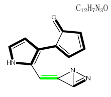 C12H7N3O	30.1418	164.2134	-20.1493	71.7347	262.687	InChI=1S/C12H7N3O/c16-11-3-1-2-8(11)7-4-5-13-9(7)6-10-12-14-15(10)12/h1-6,13H/b10-6-
B-51	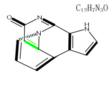 C12H7N3O	6.2016	30.9990	-2.3326	189.5883	262.7778	InChI=1S/C12H7N3O/c16-10-6-9-7-2-1-5-15(9)12(14-10)11-8(7)3-4-13-11/h1-6,13H
o-56	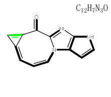 C12H7N3O	20.4834	198.8954	-14.0958	40.5978	262.8579	InChI=1S/C12H7N3O/c16-10-8-6-7(8)2-1-5-15-9-3-4-13-11(9)14-12(10)15/h1-6,13H/b5-1-,7-2-
H11-85	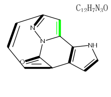 C12H7N3O	6.4286	35.297	-2.758	195.489	263.0725	InChI=1S/C12H7N3O/c16-12-9-3-1-2-7-6-10(15(12)14-7)11-8(9)4-5-13-11/h1-6,13H/b2-1-,9-3-
X22-59	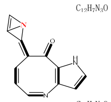 C12H7N3O	20.3671	143.2058	-19.6823	95.5263	263.0896	InChI=1S/C12H7N3O/c16-12-7(11-9-6-15(9)11)2-1-4-13-8-3-5-14-10(8)12/h1-6,14H/b2-1-,11-7-,13-4-
I1-49	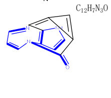 C12H7N3O	25.4235	132.0458	-16.6204	110.4888	263.0973	InChI=1S/C12H7N3O/c16-12-8-6-7(8)10-11-9(2-4-14-11)15(12)5-1-3-13-10/h1-6,14H/b5-1-,13-3+
p1-46	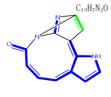 C12H7N3O	24.8224	130.8342	-11.4915	111.1514	263.143	InChI=1S/C12H7N3O/c16-10-3-1-2-7-4-5-13-11(7)8-6-9-14-12(8)15(9)10/h1-6,13H/b3-1-,7-2-,11-8+
s11-62	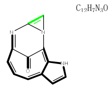 C12H7N3O	18.3945	149.4417	-12.2035	74.974	263.1573	InChI=1S/C12H7N3O/c16-12-8-3-1-2-7-4-5-13-10(7)11(12)15-6-9(15)14-8/h1-6,13H/b3-1-,7-2-,11-10-
j-5	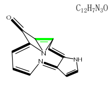 C12H7N3O	17.1725	209.2014	-10.0568	27.5804	263.1925	InChI=1S/C12H7N3O/c16-12-9-2-1-4-13-7-3-5-14-8(7)6-10-11(12)15(9)10/h1-6,14H/b4-1-,8-6-,9-2-,13-7+
x7-14	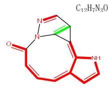 C12H7N3O	15.3351	182.0236	-7.5145	61.3871	263.2702	InChI=1S/C12H7N3O/c16-9-3-1-2-7-4-5-13-11(7)10-8-6-14-15(9)12(8)10/h1-6,13H/b3-1-,7-2-,11-10+
p1-39	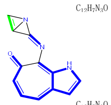 C12H7N3O	20.5694	225.0862	-16.4734	16.0036	263.3057	InChI=1S/C12H7N3O/c16-9-3-1-2-7-4-5-13-10(7)11(9)14-12-8-6-15(8)12/h1-6,13H/b14-12+
H11-61	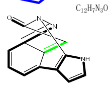 C12H7N3O	5.5278	108.7324	-1.5361	110.77	263.3921	InChI=1S/C12H7N3O/c16-11-6-9-7-2-1-3-10(9)14-15(11)12-8(7)4-5-13-12/h1-6,13H
X22-23	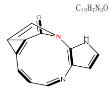 C12H7N3O	14.2153	171.8228	-7.4163	71.9234	263.5907	InChI=1S/C12H7N3O/c16-12-10-7-2-1-4-13-9-3-5-14-11(9)15(12)6-8(7)10/h1-6,14H/b2-1-,13-4-
q-58	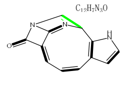 C12H7N3O	5.923	157.99	-2.4029	89.5247	263.7001	InChI=1S/C12H7N3O/c16-12-8-3-1-2-7-4-5-13-10(7)9-6-15(12)11(8)14-9/h1-6,13H/b2-1-,8-3+
i-6	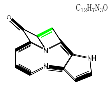 C12H7N3O	7.2029	175.8223	-3.197	54.6172	263.9338	InChI=1S/C12H7N3O/c16-12-8-2-1-4-13-7-3-5-14-11(7)9-6-10(12)15(8)9/h1-6,14H/b4-1-,8-2-,11-9+,13-7+

x6-31	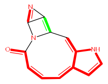	21.6554	148.8334	-10.9746	95.9225	264.1004	InChI=1S/C12H7N3O/c16-10-3-1-2-7-4-5-13-8(7)6-9-11-12(14-11)15(9)10/h1-6,13H/b3-1-,7-2-,8-6+	$C_{12}H_7N_3O$
n-51	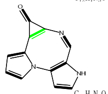	34.102	207.3871	-17.9195	21.67	264.3604	InChI=1S/C12H7N3O/c16-12-10-9-2-1-5-15(9)8-3-4-13-7(8)6-14-11(10)12/h1-6,13H/b14-6-	$C_{12}H_7N_3O$
x4-63	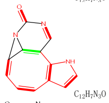	12.0486	156.3884	-7.6906	84.8926	264.4537	InChI=1S/C12H7N3O/c16-12-14-6-8-10-7(4-5-13-10)2-1-3-9-11(8)15(9)12/h1-6,13H/b2-1-,3-1-,7-2-,9-3+,10-8+	$C_{12}H_7N_3O$
H-114	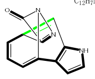	5.0618	78.2275	-2.1214	159.683	264.6516	InChI=1S/C12H7N3O/c16-12-11-7-2-1-3-10(15(12)14-11)8-4-5-13-9(8)6-7/h1-6,13H/b2-1-,3-1-,7-2-,7-6-,9-6+,10-3-,10-8-	$C_{12}H_7N_3O$
p1-5	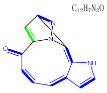	8.0875	63.9747	-5.5327	160.1358	264.8685	InChI=1S/C12H7N3O/c16-10-3-1-2-7-4-5-13-11(7)12-8-6-9(10)15(12)14-8/h1-6,13H/b3-1-,7-2-,12-11+	$C_{12}H_7N_3O$
X-26	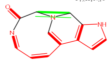	7.2363	194.8996	-3.5280	37.1332	265.0089	InChI=1S/C12H7N3O/c16-12-11-10-9-8(3-5-13-9)7(6-15(10)11)2-1-4-14-12/h1-6,13H/b2-1-,14-4-	$C_{12}H_7N_3O$
X-44	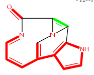	7.2359	194.8999	-3.5295	37.1377	265.0089	InChI=1S/C12H7N3O/c16-12-11-10-9-8(3-5-13-9)7(6-15(10)11)2-1-4-14-12/h1-6,13H/b2-1-,14-4+	$C_{12}H_7N_3O$
H11-132	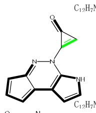	38.591	207.9669	-17.5451	21.3116	265.0898	InChI=1S/C12H7N3O/c16-11-6-10(11)15-12-8(4-5-13-12)7-2-1-3-9(7)14-15/h1-6,13H	$C_{12}H_7N_3O$
H-121	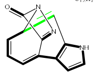	5.1059	77.9291	-2.1462	160.2427	265.1049	InChI=1S/C12H7N3O/c16-12-11-9-3-1-2-7(15(12)14-11)6-10-8(9)4-5-13-10/h1-6,13H/b2-1-,3-1-,7-2+,7-6+,9-3-,9-8-,10-6+	$C_{12}H_7N_3O$
q1-2	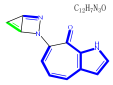	14.1784	226.9194	-13.1928	21.0411	265.1164	InChI=1S/C12H7N3O/c16-12-9(15-10-6-8(10)14-15)3-1-2-7-4-5-13-11(7)12/h1-6,13H	$C_{12}H_7N_3O$
o-164	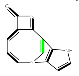	5.1972	100.7554	-2.1069	132.8425	265.1308	InChI=1S/C12H7N3O/c16-12-7-2-1-5-15-6-8(10(7)14-12)11-9(15)3-4-13-11/h1-6,13H/b5-1-,7-2+	$C_{12}H_7N_3O$
i-30	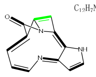	7.9178	164.4559	-2.6681	83.6337	265.1378	InChI=1S/C12H7N3O/c16-12-7-2-1-4-13-8-3-5-14-11(8)10-6-9(7)15(10)12/h1-6,14H/b2-1-,4-1-,7-2+,11-10+,13-4+,13-8+	$C_{12}H_7N_3O$
x9-31	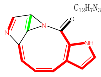	16.1107	174.6704	-7.08	67.0555	265.2259	InChI=1S/C12H7N3O/c16-12-10-7(4-5-13-10)2-1-3-8-11-9(6-14-8)15(11)12/h1-6,13H/b2-1-,8-3+	$C_{12}H_7N_3O$
A2-15	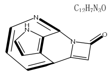	6.5305	132.3135	-1.8611	114.3836	265.3256	InChI=1S/C12H7N3O/c16-10-6-9-7-2-1-4-14-12(15(9)10)11-8(7)3-5-13-11/h1-6,13H/b2-1-,4-1-,7-2+,14-4-,14-12+	$C_{12}H_7N_3O$
H-15	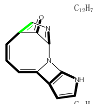	7.4784	115.4107	-1.2753	124.0781	265.4118	InChI=1S/C12H7N3O/c16-10-7-2-1-3-9-8-4-5-13-11(8)15(9)12(10)14-6-7/h1-6,13H/b2-1-,9-3-	$C_{12}H_7N_3O$
G1-103	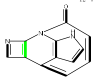	6.8029	173.9611	-2.8735	82.1857	265.6056	InChI=1S/C12H7N3O/c16-10-3-1-2-7-8-4-5-13-11(8)15(10)12-9(7)6-14-12/h1-6,13H/b3-1-,7-2+	$C_{12}H_7N_3O$
p1-2	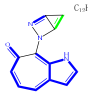	14.3271	226.7848	-13.1853	22.8881	265.733	InChI=1S/C12H7N3O/c16-10-3-1-2-7-4-5-13-11(7)12(10)15-9-6-8(9)14-15/h1-6,13H	$C_{12}H_7N_3O$
s12-23	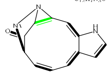	11.386	135.1393	-7.4922	95.171	265.8802	InChI=1S/C12H7N3O/c16-12-8-3-1-2-7-4-5-13-9(7)6-10-11(12)15(10)14-8/h1-6,13H/b3-1-,7-2-,9-6+	$C_{12}H_7N_3O$
x5-33	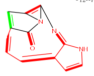	13.4776	190.8223	-6.1163	46.8948	266.0537	InChI=1S/C12H7N3O/c16-12-8-3-1-2-7-4-5-13-10(7)14-11-9(6-8)15(11)12/h1-6H,(H,13,14)	$C_{12}H_7N_3O$

F-2	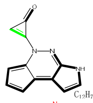 $C_{12}H_7N_3O$	38.8638	208.6444	-17.6774	21.4518	266.0698	InChI=1S/C12H7N3O/c16-11-6-10(11)15-9-3-1-2-7(9)8-4-5-13-12(8)14-15/h1-6H,(H,13,14)
x7-42	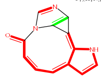 $C_{12}H_7N_3O$	15.8541	180.0457	-7.8204	62.7607	266.1268	InChI=1S/C12H7N3O/c16-8-3-1-2-7-4-5-13-10(7)9-11-12(9)15(8)6-14-11/h1-6,13H/b3-1-,7-2-,10-9+
o-91	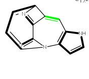 $C_{12}H_7N_3O$	3.5601	70.1685	-2.1492	181.7983	266.3691	InChI=1S/C12H7N3O/c16-12-11-7-6-9-10(3-4-13-9)15(12)5-1-2-8(7)14-11/h1-6,13H/b5-1-,8-2-
x9-21	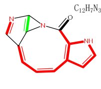 $C_{12}H_7N_3O$	15.6755	176.8735	-6.5579	66.5831	266.4055	InChI=1S/C12H7N3O/c16-12-9-7(4-5-13-9)2-1-3-8-6-14-11-10(8)15(11)12/h1-6,13H/b2-1-,8-3-
E-41	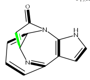 $C_{12}H_7N_3O$	7.4241	50.4435	-1.4671	181.8499	266.4793	InChI=1S/C12H7N3O/c16-11-6-9-10-3-1-2-8(14-9)7-4-5-13-12(7)15(10)11/h1-6,13H
H-117	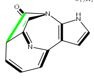 $C_{12}H_7N_3O$	4.7838	41.1789	-1.8213	197.2288	266.4958	InChI=1S/C12H7N3O/c16-12-10-7-2-1-3-9(14-10)8-4-5-13-11(8)15(12)6-7/h1-6,13H
I1-27	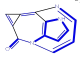 $C_{12}H_7N_3O$	19.4829	137.0406	-14.566	104.8203	266.6294	InChI=1S/C12H7N3O/c16-12-8-6-7(8)10-11-9(2-4-14-11)15(12)5-1-3-13-10/h1-6,14H/b5-1-,13-3-
E-23	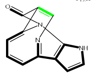 $C_{12}H_7N_3O$	7.7035	102.5525	-1.8888	132.5002	266.7656	InChI=1S/C12H7N3O/c16-12-8-6-14-9-2-1-3-10(8)15(12)11-7(9)4-5-13-11/h1-6,13H/b2-1-,3-1-,8-6+,9-2-,10-3-,14-6+,14-9-
j-18	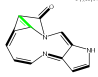 $C_{12}H_7N_3O$	17.826	194.6525	-9.7922	45.7128	266.8761	InChI=1S/C12H7N3O/c16-12-10-7-2-1-4-13-8-3-5-14-9(8)6-15(12)11(7)10/h1-6,14H/b4-1-,7-2-,9-6+,13-8+
p-1	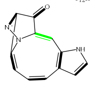 $C_{12}H_7N_3O$	6.4531	51.092	-5.6309	187.882	266.9787	InChI=1S/C12H7N3O/c16-12-10-6-8-7(4-5-13-8)2-1-3-9-11(12)14-15(9)10/h1-6,13H/b2-1-,9-3-,10-6-
H11-59	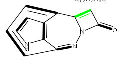 $C_{12}H_7N_3O$	4.9501	82.2704	-2.9986	163.4752	267.0869	InChI=1S/C12H7N3O/c16-11-6-10-7-2-1-3-9(14-15(10)11)12-8(7)4-5-13-12/h1-6,13H
o-65	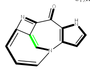 $C_{12}H_7N_3O$	7.0036	96.354	-0.2394	137.4813	267.1519	InChI=1S/C12H7N3O/c16-12-10-7-6-15(5-1-2-8(7)14-10)9-3-4-13-11(9)12/h1-6,13H
o-242	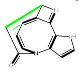 $C_{12}H_7N_3O$	6.8631	75.2938	-2.8969	169.9071	267.1796	InChI=1S/C12H7N3O/c16-10-6-8-7-2-1-5-15(10)9-3-4-13-12(9)11(7)14-8/h1-6,13H/b5-1-,7-2-,8-6+
p1-19	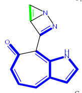 $C_{12}H_7N_3O$	14.4831	221.9069	-11.2218	21.439	267.2292	InChI=1S/C12H7N3O/c16-9-3-1-2-7-4-5-13-11(7)10(9)12-8-6-15(8)14-12/h1-6,13H
F-8	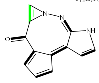	10.3947	163.1443	-8.58	83.6789	267.284	InChI=1S/C12H7N3O/c16-11-8-3-1-2-7(8)9-4-5-13-12(9)14-15-6-10(11)15/h1-6H,(H,13,14)/b9-7-
n-107	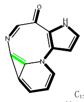 $C_{12}H_7N_3O$	5.5884	177.2905	-4.7305	63.0496	267.5498	InChI=1S/C12H7N3O/c16-9-6-14-10-7-2-1-5-15(12(7)10)8-3-4-13-11(8)9/h1-6,13H/b14-6-
G1-91	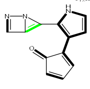 $C_{12}H_7N_3O$	11.8586	210.3654	-6.7359	40.2902	267.5749	InChI=1S/C12H7N3O/c16-10-3-1-2-7(10)8-4-5-13-11(8)12-9-6-14-15(9)12/h1-6,13H
X22-48	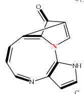 $C_{12}H_7N_3O$	13.8852	124.7053	-6.8774	116.1307	267.8009	InChI=1S/C12H7N3O/c16-11-8-6-15-10(11)7(8)2-1-4-13-9-3-5-14-12(9)15/h1-6,14H/b2-1-,13-4-
n-208	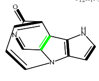 $C_{12}H_7N_3O$	7.2749	46.2621	-1.9566	180.8363	267.9216	InChI=1S/C12H7N3O/c16-12-7-2-1-5-15-8-3-4-13-11(8)10(7)9(15)6-14-12/h1-6,13H

p-5	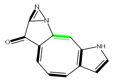 $C_{12}H_7N_3O$	18.3766	166.4133	-4.1907	70.7777	267.9266	InChI=1S/C12H7N3O/c16-11-8-3-1-2-7-4-5-13-9(7)6-10(8)15-12(11)14-15/h1-6,13H/b2-1-,3-1-,7-2-,8-3+,9-6+,10-6+
r-59	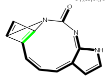 $C_{12}H_7N_3O$	32.0088	125.3754	-14.2495	111.3093	267.9708	InChI=1S/C12H7N3O/c16-12-14-11-7(4-5-13-11)2-1-3-8-9-6-10(8)15(9)12/h1-6H,(H,13,14,16)/b3-1-,7-2-
p-2	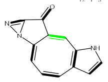 $C_{12}H_7N_3O$	18.3715	166.554	-4.2002	70.8092	268.0247	InChI=1S/C12H7N3O/c16-11-8-6-9-7(4-5-13-9)2-1-3-10(8)15-12(11)14-15/h1-6,13H/b2-1-,3-1-,7-2-,8-6+,9-6+,10-3+
F-67	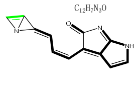 $C_{12}H_7N_3O$	20.9385	234.1669	-16.9853	19.2653	268.0811	InChI=1S/C12H7N3O/c16-12-8(7-4-5-13-11(7)14-12)2-1-3-9-10-6-15(9)10/h1-6H,(H,13,14,16)/b2-1-,9-3-
s11-21	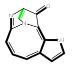 $C_{12}H_7N_3O$	5.1918	36.7051	-3.9748	196.3846	268.2974	InChI=1S/C12H7N3O/c16-12-8-6-15-9(14-8)3-1-2-7-4-5-13-10(7)11(12)15/h1-6,13H/b3-1-,7-2-,11-10+
E-48	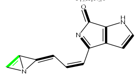 $C_{12}H_7N_3O$	21.1506	237.2349	-16.7622	15.7369	268.3125	InChI=1S/C12H7N3O/c16-12-11-7(4-5-13-11)8(14-12)2-1-3-9-10-6-15(9)10/h1-6,13H/b2-1-,9-3-
B-31	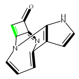 $C_{12}H_7N_3O$	3.0771	84.2254	-1.7887	160.4054	268.3231	InChI=1S/C12H7N3O/c16-10-6-9-11(10)14-8-2-1-5-15(9)12-7(8)3-4-13-12/h1-6,13H
H11-140	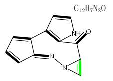 $C_{12}H_7N_3O$	13.7916	193.1917	-11.4221	45.3476	268.3574	InChI=1S/C12H7N3O/c16-12-10-6-15(10)14-9-3-1-2-7(9)8-4-5-13-11(8)12/h1-6,13H/b14-9+
H1-88	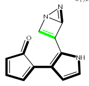 $C_{12}H_7N_3O$	23.7434	147.8239	-9.4002	100.1206	268.3744	InChI=1S/C12H7N3O/c16-10-3-1-2-7(10)8-4-5-13-11(8)9-6-15-12(9)14-15/h1-6,13H
E-63	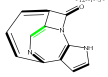 $C_{12}H_7N_3O$	5.1	88.8566	-2.7873	157.876	268.6071	InChI=1S/C12H7N3O/c16-12-8-2-1-3-9-7-4-5-13-11(7)15(12)10(8)6-14-9/h1-6,13H/b2-1-,3-1-,8-2+,9-3-
E-37	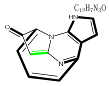 $C_{12}H_7N_3O$	5.7431	44.5414	-1.5221	193.104	268.6402	InChI=1S/C12H7N3O/c16-10-6-11-14-8-2-1-3-9(10)15(11)12-7(8)4-5-13-12/h1-6,13H/b2-1-,9-3-
H11-45	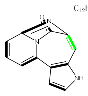 $C_{12}H_7N_3O$	5.4958	118.9425	-0.635	121.2882	268.8245	InChI=1S/C12H7N3O/c16-12-9-6-8-7(4-5-13-8)10-2-1-3-11(14-9)15(10)12/h1-6,13H
I-100	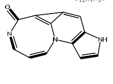 $C_{12}H_7N_3O$	12.751	155.6641	-8.5346	81.9144	268.8941	InChI=1S/C12H7N3O/c16-12-10-7-6-8-9(2-4-13-8)15(11(7)10)5-1-3-14-12/h1-6,13H/b5-1-,14-3-
X-25	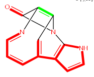 $C_{12}H_7N_3O$	8.4729	139.0849	-2.1481	110.2644	269.1313	InChI=1S/C12H7N3O/c16-12-10-9-6-7(2-1-4-13-10)8-3-5-14-11(8)15(9)12/h1-6,14H/b2-1-,4-1-,7-2+,13-4+,13-10-
o-87	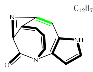 $C_{12}H_7N_3O$	8.8396	135.8842	-3.1814	102.6043	269.1346	InChI=1S/C12H7N3O/c16-12-11-7-2-1-5-15(12)10-3-4-13-9(10)6-8(7)14-11/h1-6,13H/b8-6+
H-87	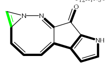 $C_{12}H_7N_3O$	13.3643	195.2826	-8.9015	43.2838	269.3	InChI=1S/C12H7N3O/c16-12-10-9(4-5-13-10)8-3-1-2-7-6-15(7)14-11(8)12/h1-6,13H/b2-1-,8-3-,14-11+
q1-56	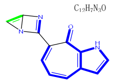 $C_{12}H_7N_3O$	15.5807	230.9341	-12.1394	16.9766	269.3425	InChI=1S/C12H7N3O/c16-11-8(12-14-9-6-15(9)12)3-1-2-7-4-5-13-10(7)11/h1-6,13H
q11-16	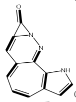 $C_{12}H_7N_3O$	13.7263	218.4223	-4.9944	30.089	269.3626	InChI=1S/C12H7N3O/c16-12-9-6-8-3-1-2-7-4-5-13-10(7)11(8)14-15(9)12/h1-6,13H
H1-127	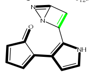 $C_{12}H_7N_3O$	23.7944	144.494	-9.8049	104.8398	269.4558	InChI=1S/C12H7N3O/c16-10-3-1-2-7(10)8-4-5-13-12(8)9-6-11-14-15(9)11/h1-6,13H

q1-38	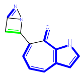	$C_{12}H_7N_3O$	23.9013	141.2334	-9.583	97.9737	269.5304	InChI=1S/C12H7N3O/c16-12-8(9-6-10-14-15(9)10)3-1-2-7-4-5-13-11(7)12/h1-6,13H
r-23	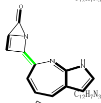	$C_{12}H_7N_3O$	16.2511	211.6004	-10.1616	50.9467	269.7676	InChI=1S/C12H7N3O/c16-12-10-6-9(15(10)12)8-3-1-2-7-4-5-13-11(7)14-8/h1-6H,(H,13,14)/b9-8-
l1-32	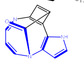	$C_{12}H_7N_3O$	6.3657	92.5972	-1.4865	143.5781	269.9233	InChI=1S/C12H7N3O/c16-12-7-6-8-10(7)11-9(2-4-14-11)15(12)5-1-3-13-8/h1-6,14H/b5-1-,13-3-
l-103	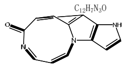	$C_{12}H_7N_3O$	10.4798	123.2725	-1.52	106.0255	269.9784	InChI=1S/C12H7N3O/c16-12-7-6-9-10(7)11-8(2-4-13-11)15(9)5-1-3-14-12/h1-6,13H/b5-1-,14-3-
X23-26	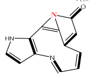	$C_{12}H_7N_3O$	18.3526	163.195	-7.9358	67.5321	270.0092	InChI=1S/C12H7N3O/c16-9-6-7-2-1-4-13-8-3-5-14-10(8)12-11(7)15(9)12/h1-6,14H/b2-1-,13-4+
X22-42	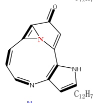	$C_{12}H_7N_3O$	15.3136	176.3069	-10.2613	69.2743	270.2289	InChI=1S/C12H7N3O/c16-10-6-9-11-7(3-5-14-11)13-4-1-2-8-12(10)15(8)9/h1-6,14H/b2-1-,13-4-
p1-35	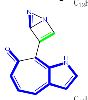	$C_{12}H_7N_3O$	23.9818	142.1045	-9.3106	99.5616	270.2334	InChI=1S/C12H7N3O/c16-9-3-1-2-7-4-5-13-11(7)10(9)8-6-15-12(8)14-15/h1-6,13H
p1-54	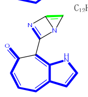	$C_{12}H_7N_3O$	16.074	230.9498	-12.1172	18.1087	270.2944	InChI=1S/C12H7N3O/c16-8-3-1-2-7-4-5-13-11(7)10(8)12-14-9-6-15(9)12/h1-6,13H
o-223	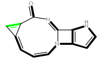	$C_{12}H_7N_3O$	15.9428	194.0952	-9.6289	39.08	270.3136	InChI=1S/C12H7N3O/c16-12-8-6-7(8)2-1-5-15-9-3-4-13-10(9)11(15)14-12/h1-6,13H/b5-1-,7-2-,14-11-
s11-23	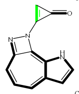	$C_{12}H_7N_3O$	42.5397	222.7128	-19.5289	10.2338	270.3293	InChI=1S/C12H7N3O/c16-10-6-9(10)15-12-8(14-15)3-1-2-7-4-5-13-11(7)12/h1-6,13H
q11-1	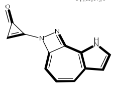	$C_{12}H_7N_3O$	41.3849	223.0005	-19.4366	9.1582	270.3331	InChI=1S/C12H7N3O/c16-10-6-9(10)15-8-3-1-2-7-4-5-13-11(7)12(8)14-15/h1-6,13H
k-25	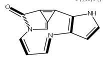	$C_{12}H_7N_3O$	14.0218	189.1175	-7.0613	63.1346	270.3507	InChI=1S/C12H7N3O/c16-12-10-7-6-15(12)5-1-3-13-8-2-4-14-11(8)9(7)10/h1-6,14H/b5-1-,13-3+
G1-67	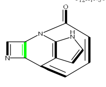	$C_{12}H_7N_3O$	6.8208	167.1528	-2.8989	92.9133	270.4957	InChI=1S/C12H7N3O/c16-10-3-1-2-7-8-4-5-13-12(8)15(10)9-6-14-11(7)9/h1-6,13H/b3-1-,7-2+
E-62	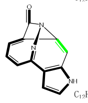	$C_{12}H_7N_3O$	4.1291	82.8702	-2.2261	167.5477	270.5926	InChI=1S/C12H7N3O/c16-12-8-2-1-3-9-7-4-5-13-10(7)6-11(8)15(12)14-9/h1-6,13H/b3-1-,8-2+
s12-36	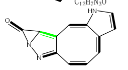	$C_{12}H_7N_3O$	12.2136	226.2923	-6.2463	28.6998	270.6062	InChI=1S/C12H7N3O/c16-12-11-8-6-10-7(4-5-13-10)2-1-3-9(8)14-15(11)12/h1-6,13H/b3-1-,7-2-,10-6+
C-25	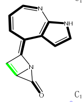	$C_{12}H_7N_3O$	16.2637	211.7792	-9.9640	49.3043	270.6401	InChI=1S/C12H7N3O/c16-12-10-6-9(15(10)12)7-2-1-4-13-11-8(7)3-5-14-11/h1-6,14H/b9-7-
q1-12	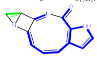	$C_{12}H_7N_3O$	17.5367	223.2863	-14.3443	23.8666	270.6582	InChI=1S/C12H7N3O/c16-12-10-7(4-5-13-10)2-1-3-8-11(14-12)9-6-15(8)9/h1-6,13H/b2-1-,8-3+,14-11+
A-208	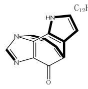	$C_{12}H_7N_3O$	7.6194	45.6854	-1.7747	184.6738	270.6881	InChI=1S/C12H7N3O/c16-12-8-2-1-5-15-6-14-10(12)11(15)9-7(8)3-4-13-9/h1-6,13H/b5-1?,8-2-
p1-9	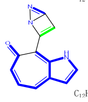	$C_{12}H_7N_3O$	23.5662	141.5112	-9.2103	99.4094	270.6925	InChI=1S/C12H7N3O/c16-9-3-1-2-7-4-5-13-12(7)11(9)8-6-10-14-15(8)10/h1-6,13H

x2-13	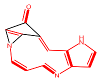	39.3498	168.5154	-17.2945	58.8043	270.6955	InChI=1S/C12H7N3O/c16-12-7-6-9-8(2-4-14-9)13-3-1-5-15-10(7)11(12)15/h1-6,14H/b5-1-,7-6-,13-3+	$C_{12}H_7N_3O$
i-3	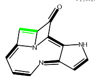	4.8713	96.4335	-4.2493	160.1313	270.9458	InChI=1S/C12H7N3O/c16-12-9-6-7-2-1-4-13-8-3-5-14-10(8)11(12)15(7)9/h1-6,14H/b4-1-,7-2-,11-10+,13-8+	$C_{12}H_7N_3O$
G1-88	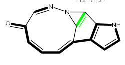	8.0354	169.9014	-4.4073	75.8332	271.0592	InChI=1S/C12H7N3O/c16-7-2-1-3-9-8-4-5-13-10(8)12-11(9)15(12)14-6-7/h1-6,13H/b2-1-,9-3-,14-6-	$C_{12}H_7N_3O$
I1-6	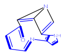	11.2898	198.8486	-8.1654	44.0154	271.099	InChI=1S/C12H7N3O/c16-9-2-1-4-13-8-3-5-14-10(8)7-6-15-11(7)12(9)15/h1-6,14H/b2-1-,13-4+	$C_{12}H_7N_3O$
I-79	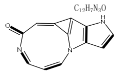	14.1355	135.5532	-8.2835	97.688	271.1617	InChI=1S/C12H7N3O/c16-9-6-7-10-11-8(2-4-14-11)15(12(7)10)5-1-3-13-9/h1-6,14H/b5-1-,7-6-,13-3-	$C_{12}H_7N_3O$
H11-42	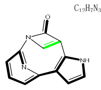	9.8659	155.4035	-2.2372	68.4777	271.4306	InChI=1S/C12H7N3O/c16-12-8-6-15(12)10-3-1-2-9(14-10)7-4-5-13-11(7)8/h1-6,13H	$C_{12}H_7N_3O$
x5-36	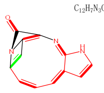	4.2497	126.3441	-3.3479	134.3575	271.4568	InChI=1S/C12H7N3O/c16-12-10-9-6-8(15(9)12)3-1-2-7-4-5-13-11(7)14-10/h1-6H,(H,13,14)	$C_{12}H_7N_3O$
H-151	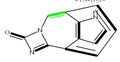	5.1668	83.207	-3.0573	169.3248	271.7613	InChI=1S/C12H7N3O/c16-12-14-11-9-3-1-2-7(6-15(11)12)10-8(9)4-5-13-10/h1-6,13H	$C_{12}H_7N_3O$
H11-84	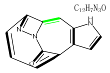	5.277	69.3427	-2.6884	178.0846	271.7748	InChI=1S/C12H7N3O/c16-12-8-2-1-3-9-11(15(12)14-9)6-10-7(8)4-5-13-10/h1-6,13H/b3-1-,8-2-	$C_{12}H_7N_3O$
C-35	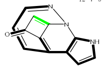	9.1177	126.0855	-2.0546	115.1748	271.9517	InChI=1S/C12H7N3O/c16-10-6-9-11(10)7-2-1-4-14-15(9)12-8(7)3-5-13-12/h1-6,13H/b2-1-,14-4-	$C_{12}H_7N_3O$
q1-7	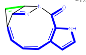	5.9748	145.3667	-2.5233	106.2465	271.9987	InChI=1S/C12H7N3O/c16-12-11-7(4-5-13-11)2-1-3-8-6-9-10(8)14-15(9)12/h1-6,13H/b2-1-,8-3-	$C_{12}H_7N_3O$
H11-21	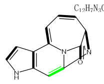	6.5446	124.1839	-0.6853	117.8515	272.0377	InChI=1S/C12H7N3O/c16-12-8-2-1-3-10-7-4-5-13-9(7)6-11(14-8)15(10)12/h1-6,13H	$C_{12}H_7N_3O$
o-221	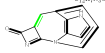	3.5493	89.1536	-2.2485	156.1442	272.0458	InChI=1S/C12H7N3O/c16-12-8-6-7-2-1-5-15(11(8)14-12)9-3-4-13-10(7)9/h1-6,13H	$C_{12}H_7N_3O$
x5-44	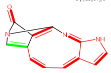	8.4426	137.4746	-4.848	119.036	272.0946	InChI=1S/C12H7N3O/c16-12-9-8-3-1-2-7-4-5-13-10(7)14-11(9)15(12)6-8/h1-6H,(H,13,14)/b3-1-,7-2-	$C_{12}H_7N_3O$
p1-29	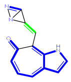	30.4155	162.9516	-19.9368	71.048	272.1779	InChI=1S/C12H7N3O/c16-10-3-1-2-7-4-5-13-11(7)8(10)6-9-12-14-15(9)12/h1-6,13H/b9-6-	$C_{12}H_7N_3O$
H11-32	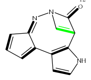	15.7146	197.7946	-5.4254	39.1802	272.2209	InChI=1S/C12H7N3O/c16-12-9-6-15(12)14-10-3-1-2-7(10)8-4-5-13-11(8)9/h1-6,13H/b14-10-	$C_{12}H_7N_3O$
A-45	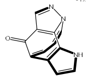	8.8134	51.1013	-1.6087	181.0185	272.306	InChI=1S/C12H7N3O/c16-12-8-2-1-5-15-11(9(12)6-14-15)10-7(8)3-4-13-10/h1-6,13H/b5-1?,8-2-	$C_{12}H_7N_3O$
q1-26	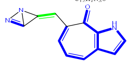	30.2024	162.9467	-19.9862	69.851	272.3173	InChI=1S/C12H7N3O/c16-11-8(6-9-12-14-15(9)12)3-1-2-7-4-5-13-10(7)11/h1-6,13H/b9-6+	$C_{12}H_7N_3O$
A1-28	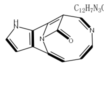	6.6847	164.3812	-4.5534	88.6674	272.9472	InChI=1S/C12H7N3O/c16-12-8-6-13-4-1-2-9-7-3-5-14-10(7)11(8)15(9)12/h1-6,14H/b2-1-,4-1-,8-6-,9-2-,13-4-,13-6-	$C_{12}H_7N_3O$

o-173	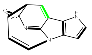	$C_{12}H_7N_3O$	6.8653	48.2711	-1.7671	185.6399	272.9663	InChI=1S/C12H7N3O/c16-12-7-2-1-5-15-9-3-4-13-10(9)8(6-7)11(15)14-12/h1-6,13H/b5-1-,7-2-
B-12	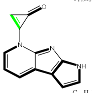	$C_{12}H_7N_3O$	38.8658	210.4918	-17.5920	26.6353	273.0177	InChI=1S/C12H7N3O/c16-10-6-9(10)15-5-1-2-8-7-3-4-13-11(7)14-12(8)15/h1-6,13H
o-6	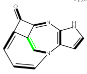	$C_{12}H_7N_3O$	6.569	97.3444	-0.4426	146.2064	273.058	InChI=1S/C12H7N3O/c16-11-7-2-1-5-15-6-8(7)10(11)14-12-9(15)3-4-13-12/h1-6,13H
B-230	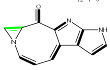	$C_{12}H_7N_3O$	12.6115	180.9988	-9.5379	62.0081	273.1227	InChI=1S/C12H7N3O/c16-11-9-6-15(9)5-1-2-7-8-3-4-13-12(8)14-10(7)11/h1-6,13H/b5-1-,7-2-
q1-31	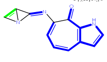	$C_{12}H_7N_3O$	17.1212	217.3519	-15.2517	30.3928	273.338	InChI=1S/C12H7N3O/c16-11-8(14-12-9-6-15(9)12)3-1-2-7-4-5-13-10(7)11/h1-6,13H/b14-12-
n-8	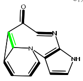	$C_{12}H_7N_3O$	14.9452	183.1758	-7.3706	52.1885	273.3404	InChI=1S/C12H7N3O/c16-9-6-14-12-8(3-4-13-12)15-5-1-2-7-10(9)11(7)15/h1-6,13H/b14-6+
F-59	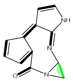	$C_{12}H_7N_3O$	40.1116	198.9502	-21.1729	23.627	273.491	InChI=1S/C12H7N3O/c16-12-9-3-1-2-7(9)8-4-5-13-11(8)14-10-6-15(10)12/h1-6H,(H,13,14)/b8-7-
q11-59	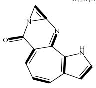	$C_{12}H_7N_3O$	20.2107	184.408	-10.8242	55.7734	273.5907	InChI=1S/C12H7N3O/c16-12-8-3-1-2-7-4-5-13-10(7)11(8)14-9-6-15(9)12/h1-6,13H
H-54	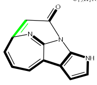	$C_{12}H_7N_3O$	6.8255	125.5488	-0.6988	114.7904	274.1684	InChI=1S/C12H7N3O/c16-10-6-7-2-1-3-8-9-4-5-13-11(9)15(10)12(8)14-7/h1-6,13H
x4-6	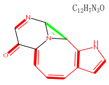	$C_{12}H_7N_3O$	16.2471	209.2332	-5.3887	28.3282	274.3784	InChI=1S/C12H7N3O/c16-9-6-14-12-11-10-7(4-5-13-10)2-1-3-8(9)15(11)12/h1-6,13H/b2-1-,8-3-
X-39	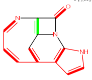	$C_{12}H_7N_3O$	11.1132	151.2987	-2.9123	96.6153	274.3875	InChI=1S/C12H7N3O/c16-12-10-9-6-7(2-1-4-13-10)8-3-5-14-11(8)15(9)12/h1-6,14H/b2-1-,4-1-,7-2+,13-4-,13-10+
I1-39	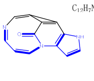	$C_{12}H_7N_3O$	7.4904	177.622	-5.6039	75.3332	274.7072	InChI=1S/C12H7N3O/c16-12-10-7-6-13-3-1-5-15(12)8-2-4-14-11(8)9(7)10/h1-6,14H/b5-1-,7-6-,13-3-
H-150	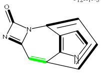	$C_{12}H_7N_3O$	7.3724	139.3043	-2.3279	120.6349	274.7694	InChI=1S/C12H7N3O/c16-12-14-10-6-7-2-1-3-9(15(10)12)8-4-5-13-11(7)8/h1-6,13H
H1-7	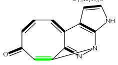	$C_{12}H_7N_3O$	6.6098	59.3041	-3.9741	180.9843	274.8727	InChI=1S/C12H7N3O/c16-7-2-1-3-8-9-4-5-13-12(9)15-10(6-7)11(8)14-15/h1-6,13H/b2-1-,8-3-,10-6+
H1-63	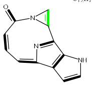	$C_{12}H_7N_3O$	25.04	191.7265	-15.6019	47.2029	274.8918	InChI=1S/C12H7N3O/c16-10-3-1-2-8-7-4-5-13-11(7)12(14-8)9-6-15(9)10/h1-6,13H/b3-1-,8-2-
H1-78	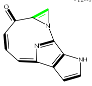	$C_{12}H_7N_3O$	12.7327	222.6855	-4.8014	14.209	275.0531	InChI=1S/C12H7N3O/c16-10-3-1-2-8-7-4-5-13-11(7)12(14-8)15-6-9(10)15/h1-6,13H/b3-1-,8-2-
I1-5	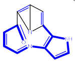	$C_{12}H_7N_3O$	15.7571	196.9395	-9.5163	46.4494	275.1376	InChI=1S/C12H7N3O/c16-10-2-1-4-13-7-3-5-14-11(7)8-6-9-12(10)15(8)9/h1-6,14H/b2-1-,13-4+
q11-26	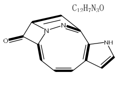	$C_{12}H_7N_3O$	7.2065	141.3603	-0.1822	106.5311	275.2076	InChI=1S/C12H7N3O/c16-12-9-3-1-2-7-4-5-13-11(7)8-6-10(12)15(9)14-8/h1-6,13H/b2-1-,9-3-
A2-43	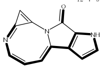	$C_{12}H_7N_3O$	18.2608	168.3353	-13.2196	69.5441	275.2699	InChI=1S/C12H7N3O/c16-12-11-7(3-5-14-11)9-2-1-4-13-8-6-10(8)15(9)12/h1-6,14H/b4-1-,9-2-,13-8-

H11-120	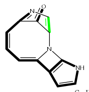	$C_{12}H_7N_3O$	7.9895	113.8385	-1.3477	132.1924	275.2775	InChI=1S/C12H7N3O/c16-11-8-2-1-3-9-7-4-5-13-12(7)15(9)10(11)6-14-8/h1-6,13H/b2-1-,9-3-
B-232	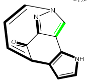	$C_{12}H_7N_3O$	7.6716	48.8210	-1.5878	190.3081	275.3252	InChI=1S/C12H7N3O/c16-12-8-2-1-5-15-6-9(11(12)14-15)10-7(8)3-4-13-10/h1-6,13H/b5-1-,8-2-
H11-76	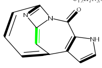	$C_{12}H_7N_3O$	10.0745	126.3189	-2.8752	129.7801	275.4223	InChI=1S/C12H7N3O/c16-12-11-8(4-5-13-11)7-2-1-3-9-14-10(6-7)15(9)12/h1-6,13H/b2-1-,3-1-,7-2+,9-3+
X22-55	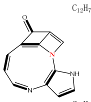	$C_{12}H_7N_3O$	26.2292	243.8188	0.3743	1.53	275.5194	InChI=1S/C12H7N3O/c16-11-7-2-1-4-13-9-3-5-14-12(9)15-6-8(11)10(7)15/h1-6,14H/b2-1-,13-4-
B-195	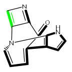	$C_{12}H_7N_3O$	2.9391	87.3509	-1.7340	164.1195	275.5687	InChI=1S/C12H7N3O/c16-11-7-2-1-5-15(9-6-14-10(9)11)12-8(7)3-4-13-12/h1-6,13H
x4-61	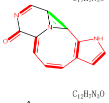	$C_{12}H_7N_3O$	16.8119	208.357	-5.7384	26.788	275.6575	InChI=1S/C12H7N3O/c16-12-8-3-1-2-7-4-5-13-10(7)11-9(6-14-12)15(8)11/h1-6,13H/b2-1-,8-3-
X22-34	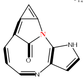	$C_{12}H_7N_3O$	16.3042	172.5104	-13.7678	90.2156	275.6871	InChI=1S/C12H7N3O/c16-12-7-2-1-4-13-9-3-5-14-11(9)15(12)10-6-8(7)10/h1-6,14H/b2-1-,13-4-
B-239	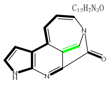	$C_{12}H_7N_3O$	6.0569	115.5031	-0.2536	130.0077	275.7503	InChI=1S/C12H7N3O/c16-12-10-9-6-15(12)5-1-2-7(9)8-3-4-13-11(8)14-10/h1-6,13H
X23-30	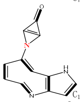	$C_{12}H_7N_3O$	51.6988	163.949	-29.8209	70.0776	275.802	InChI=1S/C12H7N3O/c16-12-10-11(12)15(10)7-2-1-4-13-8-3-5-14-9(8)6-7/h1-6,14H/b2-1-,4-1+,7-2+,7-6-,9-6-,13-4+,13-8-
I-86	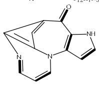	$C_{12}H_7N_3O$	4.4177	54.2127	-1.5254	189.8087	275.8522	InChI=1S/C12H7N3O/c16-12-7-6-9-10(7)13-3-1-5-15(9)8-2-4-14-11(8)12/h1-6,14H
o-232	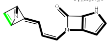	$C_{12}H_7N_3O$	26.2116	197.3671	-11.8989	54.3868	276.0241	InChI=1S/C12H7N3O/c16-12-11-10(3-4-13-11)15(12)5-1-2-7-8-6-9(7)14-8/h1-6,13H/b5-1-
x7-18	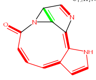	$C_{12}H_7N_3O$	17.0302	177.5692	-6.6691	72.4619	276.0585	InChI=1S/C12H7N3O/c16-9-3-1-2-7-4-5-13-10(7)11-12-8(6-14-11)15(9)12/h1-6,13H/b3-1-,7-2-,11-10-
q1-61	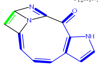	$C_{12}H_7N_3O$	5.0171	86.072	-4.0934	169.4279	276.1309	InChI=1S/C12H7N3O/c16-11-10-7(4-5-13-10)2-1-3-8-6-9-14-12(11)15(8)9/h1-6,13H/b2-1-,8-3-
q11-61	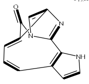	$C_{12}H_7N_3O$	4.6015	58.1414	-3.3169	190.4479	276.3832	InChI=1S/C12H7N3O/c16-12-8-3-1-2-7-4-5-13-10(7)11-14-9(8)6-15(11)12/h1-6,13H/b2-1-,8-3+
r-45	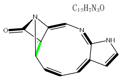	$C_{12}H_7N_3O$	21.7479	185.3757	-10.6078	64.7796	276.3953	InChI=1S/C12H7N3O/c16-12-10-8-3-1-2-7-4-5-13-11(7)14-6-9(10)15(8)12/h1-6H,(H,13,14)
I1-44	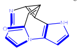	$C_{12}H_7N_3O$	11.8119	168.5367	-5.6766	77.8494	276.8031	InChI=1S/C12H7N3O/c16-9-6-7-10-11(7)13-3-1-5-15(9)8-2-4-14-12(8)10/h1-6,14H/b5-1-,13-3+
B-176	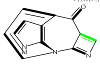	$C_{12}H_7N_3O$	3.0637	88.1284	-1.8602	167.7954	277.0013	InChI=1S/C12H7N3O/c16-10-7-2-1-5-15(12-9(10)6-14-12)11-8(7)3-4-13-11/h1-6,13H
q11-14	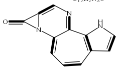	$C_{12}H_7N_3O$	16.1289	212.0865	-5.4804	44.5816	277.1225	InChI=1S/C12H7N3O/c16-12-9-6-14-11-8(15(9)12)3-1-2-7-4-5-13-10(7)11/h1-6,13H
s11-1	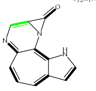	$C_{12}H_7N_3O$	16.139	211.9739	-5.4697	44.5989	277.1847	InChI=1S/C12H7N3O/c16-12-9-6-14-8-3-1-2-7-4-5-13-10(7)11(8)15(9)12/h1-6,13H

s11-32	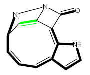 $C_{12}H_7N_3O$	7.4786	194.4991	-1.6047	61.4967	277.3735	InChI=1S/C12H7N3O/c16-12-10-9-6-8(14-15(9)12)3-1-2-7-4-5-13-11(7)10/h1-6,13H/b3-1-,7-2-,11-10-
x7-48	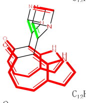 $C_{12}H_7N_3O$	13.2181	239.0131	-8.41	15.2593	277.3751	InChI=1S/C12H7N3O/c16-8-3-1-2-7-4-5-13-10(7)9(8)11-12-14-6-15(11)12/h1-6,13H
r-19	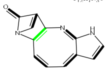 $C_{12}H_7N_3O$	17.6553	143.2303	-9.3937	111.7988	277.4144	InChI=1S/C12H7N3O/c16-12-8-6-15(12)9-3-1-2-7-4-5-13-11(7)14-10(8)9/h1-6H,(H,13,14)
p1-37	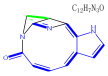 $C_{12}H_7N_3O$	7.2071	89.4664	-5.3907	166.3174	277.7072	InChI=1S/C12H7N3O/c16-9-3-1-2-7-4-5-13-10(7)11-8-6-15(9)12(8)14-11/h1-6,13H/b3-1-,7-2-,11-10+
H-109	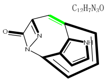 $C_{12}H_7N_3O$	5.7459	78.967	-2.7912	175.6216	277.8076	InChI=1S/C12H7N3O/c16-12-9-6-7-2-1-3-10(15(12)14-9)8-4-5-13-11(7)8/h1-6,13H/b7-6-
C-48	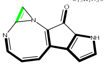 $C_{12}H_7N_3O$	12.0928	184.3321	-8.8533	56.4076	278.0940	InChI=1S/C12H7N3O/c16-12-10-7(3-5-14-10)8-2-1-4-13-9-6-15(9)11(8)12/h1-6,14H/b2-1-,13-4-
q-16	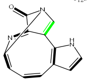 $C_{12}H_7N_3O$	4.0725	54.2207	-3.0311	198.4354	278.133	InChI=1S/C12H7N3O/c16-12-11-8-6-15(12)9(14-11)3-1-2-7-4-5-13-10(7)8/h1-6,13H/b2-1-,9-3-
k-38	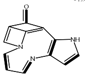 $C_{12}H_7N_3O$	5.9834	82.1597	-5.3412	179.2775	278.1828	InChI=1S/C12H7N3O/c16-12-7-6-15-5-1-3-13-8-2-4-14-10(8)9(12)11(7)15/h1-6,14H/b5-1-,13-3+
q11-11	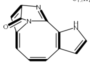 $C_{12}H_7N_3O$	4.0203	117.3499	-3.1354	139.5078	278.3459	InChI=1S/C12H7N3O/c16-12-9-6-8-3-1-2-7-4-5-13-10(7)11(14-9)15(8)12/h1-6,13H/b2-1-,3-1-,7-2-,8-3-,11-10+
E-24	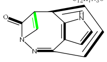 $C_{12}H_7N_3O$	6.9557	73.431	-3.75	169.2907	278.3973	InChI=1S/C12H7N3O/c16-12-9-6-15(12)14-10-3-1-2-7(9)11-8(10)4-5-13-11/h1-6,13H/b14-10-
E-4	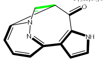 $C_{12}H_7N_3O$	7.475	74.7653	-2.0193	169.1013	278.5342	InChI=1S/C12H7N3O/c16-12-8-6-15-10(8)3-1-2-9(14-15)7-4-5-13-11(7)12/h1-6,13H
o-171	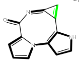 $C_{12}H_7N_3O$	14.0977	195.5762	-8.99	45.8534	278.6145	InChI=1S/C12H7N3O/c16-12-10-2-1-5-15(10)9-3-4-13-11(9)7-6-8(7)14-12/h1-6,13H/b14-8-
s11-55	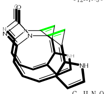 $C_{12}H_7N_3O$	21.8922	206.0038	-16.5636	46.0932	278.8171	InChI=1S/C12H7N3O/c16-12-14-10-3-1-2-7-4-5-13-11(7)8-6-9(8)15(10)12/h1-6,13H/b3-1-,7-2-,11-8+
p1-25	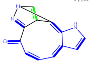 $C_{12}H_7N_3O$	8.1162	61.4223	-5.4189	173.079	278.8189	InChI=1S/C12H7N3O/c16-9-3-1-2-7-4-5-13-10(7)12-8-6-15(12)14-11(8)9/h1-6,13H/b3-1-,7-2-,12-10-
F-33	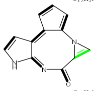 $C_{12}H_7N_3O$	21.934	223.0169	-15.085	21.5659	278.9946	InChI=1S/C12H7N3O/c16-12-10-6-15(10)9-3-1-2-7(9)8-4-5-13-11(8)14-12/h1-6H,(H,13,14,16)/b8-7-
q-61	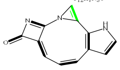 $C_{12}H_7N_3O$	18.463	188.2292	-11.0039	55.2189	279.568	InChI=1S/C12H7N3O/c16-12-8-3-1-2-7-4-5-13-10(7)9-6-15(9)11(8)14-12/h1-6,13H/b2-1-,8-3+
G1-69	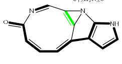 $C_{12}H_7N_3O$	10.2691	189.9755	-7.383	65.1971	279.5942	InChI=1S/C12H7N3O/c16-10-3-1-2-7-8-4-5-13-12(8)15-9(6-14-10)11(7)15/h1-6,13H/b3-1-,7-2-,14-6-
s11-61	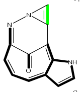 $C_{12}H_7N_3O$	24.3443	166.8828	-16.6638	65.5752	279.6296	InChI=1S/C12H7N3O/c16-12-8-3-1-2-7-4-5-13-11(7)10(12)9-6-15(9)14-8/h1-6,13H/b3-1-,7-2-,11-10+
s11-31	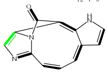 $C_{12}H_7N_3O$	5.307	115.589	-2.8561	149.8005	279.6963	InChI=1S/C12H7N3O/c16-12-10-8-6-14-9(15(8)12)3-1-2-7-4-5-13-11(7)10/h1-6,13H/b2-1-,3-1-,7-2-,9-3-,11-10-

s12-6	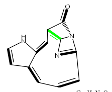 $C_{12}H_7N_3O$	6.347	104.381	-5.1005	161.8141	279.8449	InChI=1S/C12H7N3O/c16-12-8-6-9-7(4-5-13-9)2-1-3-10-14-11(8)15(10)12/h1-6,13H/b2-1-,3-1-,7-2-,8-6-,9-6-,10-3+
l1-42	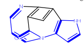 $C_{12}H_7N_3O$	**8.1298**	128.0553	-3.6113	107.0311	279.8549	InChI=1S/C12H7N3O/c16-12-8-6-7-10(8)13-3-1-5-15(12)9-2-4-14-11(7)9/h1-6,14H/b5-1-,13-3-
i-11	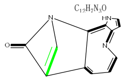 $C_{12}H_7N_3O$	6.5998	138.0683	-5.6709	128.6945	280.115	InChI=1S/C12H7N3O/c16-12-8-6-15(12)11-7(8)2-1-4-13-9-3-5-14-10(9)11/h1-6,14H/b2-1-,4-1-,7-2-,11-10-,13-4+,13-9+
F-35	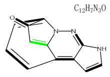 $C_{12}H_7N_3O$	7.3647	41.5509	-3.1719	201.8181	280.1486	InChI=1S/C12H7N3O/c16-11-6-10-7-2-1-3-9(11)15(10)14-12-8(7)4-5-13-12/h1-6H,(H,13,14)
I-82	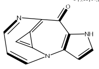 $C_{12}H_7N_3O$	18.975	137.0755	-12.4504	99.354	280.4558	InChI=1S/C12H7N3O/c16-12-10-7-6-9(7)15(5-1-3-13-10)8-2-4-14-11(8)12/h1-6,14H/b5-1-,13-3-
H11-41	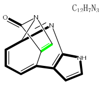 $C_{12}H_7N_3O$	5.112	76.1242	-2.0431	181.4048	280.7742	InChI=1S/C12H7N3O/c16-12-9-6-15(12)10-3-1-2-7(9)8-4-5-13-11(8)14-10/h1-6H,(H,13,14)
H11-158	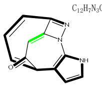 $C_{12}H_7N_3O$	8.247	76.1061	-2.777	171.8843	280.7794	InChI=1S/C12H7N3O/c16-11-6-10-9-3-1-2-7(11)8-4-5-13-12(8)15(10)14-9/h1-6,13H/b3-1-,7-2-
H-110	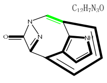 $C_{12}H_7N_3O$	5.564	69.4222	-3.0255	185.0469	280.8484	InChI=1S/C12H7N3O/c16-12-11-8-3-1-2-7(6-15(12)14-11)10-9(8)4-5-13-10/h1-6,13H/b7-6-
A2-7	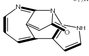 $C_{12}H_7N_3O$	5.2075	103.292	-1.869	152.6436	281.1129	InChI=1S/C12H7N3O/c16-10-6-9-7-2-1-4-13-12(9)15(10)11-8(7)3-5-14-11/h1-6,14H
A2-4	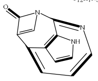 $C_{12}H_7N_3O$	4.2734	92.4946	-1.9569	164.8948	281.1491	InChI=1S/C12H7N3O/c16-12-9-6-15(12)11-10-8(3-5-13-10)7(9)2-1-4-14-11/h1-6,13H/b2-1-,4-1-,7-2+,14-4-,14-11+
A-154	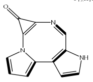 $C_{12}H_7N_3O$	25.7166	218.7105	-10.2464	33.8365	281.1715	InChI=1S/C12H7N3O/c16-12-10-11(12)15-5-1-2-9(15)7-3-4-13-8(7)6-14-10/h1-6,13H/b14-6-
B-178	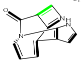 $C_{12}H_7N_3O$	5.5621	134.3960	-2.8733	125.8273	281.3962	InChI=1S/C12H7N3O/c16-12-9-6-14-10(9)7-2-1-5-15(12)11-8(7)3-4-13-11/h1-6,13H
B-45	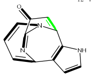 $C_{12}H_7N_3O$	7.0862	33.6130	-2.1727	205.4868	281.4349	InChI=1S/C12H7N3O/c16-10-6-9-11-7(3-4-13-11)8-2-1-5-15(9)12(10)14-8/h1-6,13H
H11-31	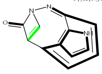 $C_{12}H_7N_3O$	6.3162	68.2397	-4.0796	183.1139	281.4543	InChI=1S/C12H7N3O/c16-12-9-6-15(12)14-10-3-1-2-7(9)8-4-5-13-11(8)10/h1-6,13H/b14-10-
H11-64	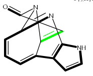 $C_{12}H_7N_3O$	5.2526	125.1335	-1.9385	140.5917	281.8557	InChI=1S/C12H7N3O/c16-11-6-9-7-2-1-3-10(15(9)11)14-12-8(7)4-5-13-12/h1-6H,(H,13,14)
D-10	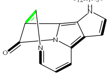 $C_{12}H_7N_3O$	7.4589	118.7892	-2.2445	146.3350	281.9242	InChI=1S/C12H7N3O/c16-12-8-6-13-4-1-2-9-7-3-5-14-10(7)11(8)15(9)12/h1-6,14H/b2-1-,4-1-,8-6+,9-2-,13-4+,13-6-
D-9	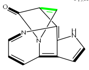 $C_{12}H_7N_3O$	6.6261	78.6423	-2.7199	173.8510	282.0449	InChI=1S/C12H7N3O/c16-12-8-6-15(12)9-2-1-4-13-11(8)10-7(9)3-5-14-10/h1-6,14H/b2-1-,4-1-,9-2-,13-4-,13-11-
q1-20	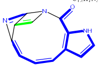 $C_{12}H_7N_3O$	7.1574	124.9421	-4.7741	139.6512	282.2053	InChI=1S/C12H7N3O/c16-12-10-7(4-5-13-10)2-1-3-9-8-6-15(12)11(8)14-9/h1-6,13H/b2-1-,9-3+
p1-42	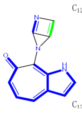 $C_{12}H_7N_3O$	18.5858	148.1898	-12.281	110.2526	282.3388	InChI=1S/C12H7N3O/c16-9-3-1-2-7-4-5-13-10(7)11(9)15-8-6-14-12(8)15/h1-6,13H

x4-9	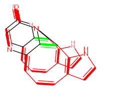	$C_{12}H_7N_3O$	19.8651	173.9418	-9.0431	72.3495	282.4306	InChI=1S/C12H7N3O/c16-9-6-14-8-3-1-2-7-4-5-13-10(7)12-11(8)15(9)12/h1-6,13H/b2-1-,3-1-,7-2-,8-3+,12-10-
H-43	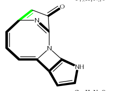	$C_{12}H_7N_3O$	9.7589	183.1233	-0.4698	62.8536	282.4359	InChI=1S/C12H7N3O/c16-10-6-7-2-1-3-9-8-4-5-13-11(8)15(9)12(10)14-7/h1-6,13H/b2-1-,9-3-
H1-42	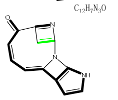	$C_{12}H_7N_3O$	6.1155	147.9819	-3.1386	121.5911	282.4394	InChI=1S/C12H7N3O/c16-10-3-1-2-9-7-4-5-13-12(7)15(9)11-6-8(10)14-11/h1-6,13H/b3-1-,9-2-
q11-54	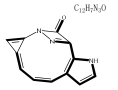	$C_{12}H_7N_3O$	24.1465	219.166	-17.3339	33.4006	282.5366	InChI=1S/C12H7N3O/c16-12-11-10-7(4-5-13-10)2-1-3-8-6-9(8)15(12)14-11/h1-6,13H/b2-1-,8-3-
x4-66	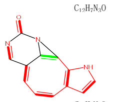	$C_{12}H_7N_3O$	20.1145	175.6835	-9.2798	69.8529	282.7059	InChI=1S/C12H7N3O/c16-12-14-6-8-3-1-2-7-4-5-13-9(7)11-10(8)15(11)12/h1-6,13H/b2-1-,3-1-,7-2-,8-3-,11-9-
F-19	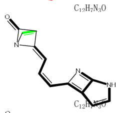	$C_{12}H_7N_3O$	31.7268	222.4865	-16.9974	32.8786	282.7382	InChI=1S/C12H7N3O/c16-12-8-6-15(12)10(8)3-1-2-9-7-4-5-13-11(7)14-9/h1-6H,(H,13,14)/b2-1-,10-3-
s12-41	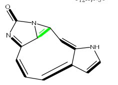	$C_{12}H_7N_3O$	18.8176	175.5205	-12.533	71.5772	282.8291	InChI=1S/C12H7N3O/c16-12-14-8-3-1-2-7-4-5-13-9(7)6-10-11(8)15(10)12/h1-6,13H/b3-1-,7-2-,9-6-
r1-37	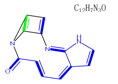	$C_{12}H_7N_3O$	31.155	161.9261	-15.8418	91.2495	283.181	InChI=1S/C12H7N3O/c16-10-3-1-2-7-4-5-13-12(7)14-8-6-9-11(8)15(9)10/h1-6H,(H,13,14)/b3-1+,7-2-
s11-56	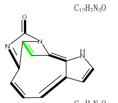	$C_{12}H_7N_3O$	20.2113	138.2119	-5.6082	86.9825	283.3825	InChI=1S/C12H7N3O/c16-12-14-8-3-1-2-7-4-5-13-11(7)10-6-9(8)15(10)12/h1-6,13H/b3-1-,7-2-,11-10-
X22-50	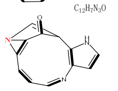	$C_{12}H_7N_3O$	11.9073	182.9028	-5.2772	75.4771	283.4417	InChI=1S/C12H7N3O/c16-12-7-6-15-9(11(12)15)2-1-4-13-8-3-5-14-10(7)8/h1-6,14H/b2-1-,13-4-
H-71	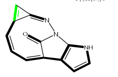	$C_{12}H_7N_3O$	26.1328	156.0144	-16.6645	101.6278	283.4926	InChI=1S/C12H7N3O/c16-12-9-3-1-2-7-6-10(7)14-15(12)11-8(9)4-5-13-11/h1-6,13H/b2-1-,9-3-,14-10-
H11-149	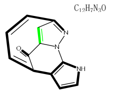	$C_{12}H_7N_3O$	6.2858	34.7141	-2.3357	215.1499	283.607	InChI=1S/C12H7N3O/c16-11-8-3-1-2-7-6-10(11)15(14-7)12-9(8)4-5-13-12/h1-6,13H/b2-1-,8-3-
q-5	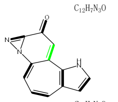	$C_{12}H_7N_3O$	19.9815	175.3068	-1.8891	71.9789	283.6541	InChI=1S/C12H7N3O/c16-10-6-8-9(15-12(10)14-15)3-1-2-7-4-5-13-11(7)8/h1-6,13H
x5-7	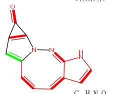	$C_{12}H_7N_3O$	36.2137	241.0198	-17.5869	16.5188	283.6602	InChI=1S/C12H7N3O/c16-11-9-6-8-3-1-2-7-4-5-13-12(7)14-15(8)10(9)11/h1-6H,(H,13,14)/b3-1-,7-2-
p-12	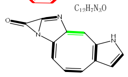	$C_{12}H_7N_3O$	11.3561	226.5495	-5.5322	46.7539	283.6822	InChI=1S/C12H7N3O/c16-12-11-14-9-6-8-7(4-5-13-8)2-1-3-10(9)15(11)12/h1-6,13H/b2-1-,3-1-,7-2-,8-6+,9-6+,10-3+
p-11	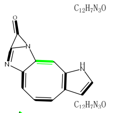	$C_{12}H_7N_3O$	11.3579	226.5228	-5.5296	46.7439	283.6852	InChI=1S/C12H7N3O/c16-12-11-14-8-3-1-2-7-4-5-13-9(7)6-10(8)15(11)12/h1-6,13H/b2-1-,3-1-,7-2-,8-3+,9-6+,10-6+
o-255	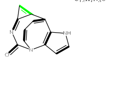	$C_{12}H_7N_3O$	32.1644	161.3824	-15.8338	96.5502	283.9396	InChI=1S/C12H7N3O/c16-12-14-9-6-8(9)7-2-1-5-15(12)10-3-4-13-11(7)10/h1-6,13H/b14-9-
l1-3	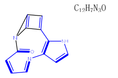	$C_{12}H_7N_3O$	32.1635	161.3787	-15.833	96.5467	283.9396	InChI=1S/C12H7N3O/c16-10-2-1-4-13-8-3-5-14-11(8)7-6-9-12(7)15(9)10/h1-6,14H/b2-1-,13-4+
r-22	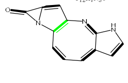	$C_{12}H_7N_3O$	13.8999	223.3773	-6.2927	50.4139	283.9833	InChI=1S/C12H7N3O/c16-12-10-6-8-9(15(10)12)3-1-2-7-4-5-13-11(7)14-8/h1-6H,(H,13,14)

q1-30	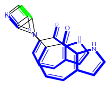 $C_{12}H_7N_3O$	18.6139	148.5902	-12.3521	109.1813	284.0614	InChI=1S/C12H7N3O/c16-11-8(15-9-6-14-12(9)15)3-1-2-7-4-5-13-10(7)11/h1-6,13H
o-71	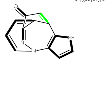 $C_{12}H_7N_3O$	5.7086	33.498	-1.789	212.1177	284.1755	InChI=1S/C12H7N3O/c16-10-6-8-7-2-1-5-15(14-12(7)10)9-3-4-13-11(8)9/h1-6,13H
H-127	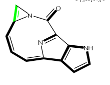 $C_{12}H_7N_3O$	29.3564	193.3415	-11.9977	49.7231	284.4018	InChI=1S/C12H7N3O/c16-12-11-10-8(4-5-13-10)9(14-11)3-1-2-7-6-15(7)12/h1-6,13H/b2-1-,9-3-
l1-50	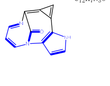 $C_{12}H_7N_3O$	26.3107	171.2122	-12.454	82.3967	284.5098	InChI=1S/C12H7N3O/c16-12-11-8-6-7(8)10-9(2-4-14-10)15(12)5-1-3-13-11/h1-6,14H
p1-44	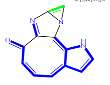 $C_{12}H_7N_3O$	17.0289	173.0059	-11.6986	83.094	284.6302	InChI=1S/C12H7N3O/c16-8-3-1-2-7-4-5-13-10(7)12-11(8)14-9-6-15(9)12/h1-6,13H/b3-1-,7-2-,12-10-
s12-35	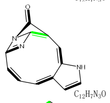 $C_{12}H_7N_3O$	10.6871	182.9374	-3.5507	86.6145	284.8534	InChI=1S/C12H7N3O/c16-12-8-6-9-7(4-5-13-9)2-1-3-10-14-11(8)15(10)12/h1-6,13H/b2-1-,3-1-,7-2-,8-6+,9-6+,10-3+
H-77	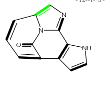 $C_{12}H_7N_3O$	4.5077	121.8012	-1.0678	133.9233	284.8888	InChI=1S/C12H7N3O/c16-12-9-3-1-2-7-6-14-11(15(7)12)10-8(9)4-5-13-10/h1-6,13H
I-32	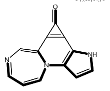 $C_{12}H_7N_3O$	24.9057	232.3668	-8.9476	19.8525	284.8904	InChI=1S/C12H7N3O/c16-12-9-8-6-13-3-1-5-15(8)7-2-4-14-11(7)10(9)12/h1-6,14H
G-29	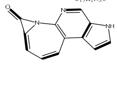 $C_{12}H_7N_3O$	24.1763	197.8558	-10.9561	60.9463	285.5162	InChI=1S/C12H7N3O/c16-12-10-3-1-2-8-7-4-5-13-9(7)6-14-11(8)15(10)12/h1-6,13H
H-91	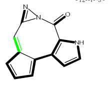 $C_{12}H_7N_3O$	24.6936	204.8164	-6.1077	40.7249	285.7901	InChI=1S/C12H7N3O/c16-12-11-9(4-5-13-11)8-3-1-2-7(8)6-10-14-15(10)12/h1-6,13H/b7-6-
H-68	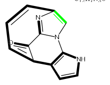 $C_{12}H_7N_3O$	7.5599	36.4479	-2.742	211.7965	285.8006	InChI=1S/C12H7N3O/c16-10-8-3-1-2-7-6-15(12(10)14-7)11-9(8)4-5-13-11/h1-6,13H/b2-1-,8-3-
H1-119	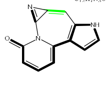 $C_{12}H_7N_3O$	19.4506	160.2801	-7.126	90.6457	285.8536	InChI=1S/C12H7N3O/c16-11-3-1-2-10-7-4-5-13-8(7)6-9-12(14-9)15(10)11/h1-6,13H
D-13	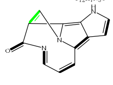 $C_{12}H_7N_3O$	7.7136	148.1636	-1.6579	104.7636	286.0760	InChI=1S/C12H7N3O/c16-12-8-6-15-9(2-1-4-14-12)7-3-5-13-10(7)11(8)15/h1-6,13H/b2-1-,14-4+
C-68	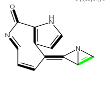 $C_{12}H_7N_3O$	21.1280	242.3229	-16.6603	16.0694	286.1138	InChI=1S/C12H7N3O/c16-12-10-7(3-5-13-10)8(2-1-4-14-12)11-9-6-15(9)11/h1-6,13H/b2-1-,11-8-,14-4+
l1-7	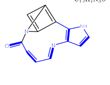 $C_{12}H_7N_3O$	33.7945	117.9504	-13.625	118.2906	286.2965	InChI=1S/C12H7N3O/c16-10-2-1-4-13-7-3-5-14-12(7)11-8-6-9(11)15(8)10/h1-6,14H/b2-1-,13-4+
k-17	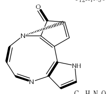 $C_{12}H_7N_3O$	6.0656	51.6611	-4.8573	212.9572	286.6337	InChI=1S/C12H7N3O/c16-12-9-6-7-10-8(2-4-14-10)13-3-1-5-15(9)11(7)12/h1-6,14H/b5-1-,13-3-
x4-30	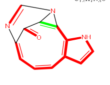 $C_{12}H_7N_3O$	11.2802	211.0477	-5.3628	46.4336	286.6464	InChI=1S/C12H7N3O/c16-12-8-3-1-2-7-4-5-13-9(7)10-11(12)15(10)6-14-8/h1-6,13H/b2-1-,8-3+
i-2	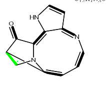 $C_{12}H_7N_3O$	6.0242	48.2635	-5.1651	209.3984	286.6912	InChI=1S/C12H7N3O/c16-12-7-6-15-9(7)2-1-4-13-8-3-5-14-10(8)11(12)15/h1-6,14H/b4-1-,9-2-,11-10+,13-8-
B-22	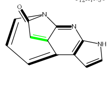 $C_{12}H_7N_3O$	5.4204	104.2680	-1.4774	157.9607	286.9300	InChI=1S/C12H7N3O/c16-10-6-9-7-2-1-5-15(10)12(9)14-11-8(7)3-4-13-11/h1-6,13H

B-221	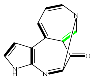	$C_{12}H_7N_3O$	6.5173	31.6942	-1.7927	212.9590	286.9998	InChI=1S/C12H7N3O/c16-10-9-6-15-5-1-2-7(9)8-3-4-13-11(8)14-12(10)15/h1-6,13H
H11-115	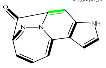	$C_{12}H_7N_3O$	7.6332	128.7719	-1.1949	120.0252	287.0608	InChI=1S/C12H7N3O/c16-12-8-2-1-3-10-7-4-5-13-9(7)6-11(12)15(10)14-8/h1-6,13H
j-22	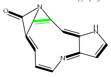	$C_{12}H_7N_3O$	19.7783	191.6137	-13.2327	68.5785	287.1839	InChI=1S/C12H7N3O/c16-12-7-2-1-4-13-8-3-5-14-9(8)6-10-11(7)15(10)12/h1-6,14H/b2-1+,4-1-,7-2-,9-6-,10-6-,13-4+,13-8+
A-161	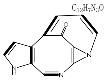	$C_{12}H_7N_3O$	13.2003	176.6799	-5.2391	62.1327	287.295	InChI=1S/C12H7N3O/c16-11-8-2-1-5-15-10(11)12(15)14-6-9-7(8)3-4-13-9/h1-6,13H/b14-6-
I-105	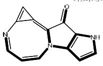	$C_{12}H_7N_3O$	16.8959	178.7622	-12.945	73.9829	287.5264	InChI=1S/C12H7N3O/c16-12-10-9(2-4-14-10)15-5-1-3-13-8-6-7(8)11(12)15/h1-6,14H/b5-1-,11-7-,13-3-
B-181	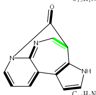	$C_{12}H_7N_3O$	4.6252	104.7459	-1.6566	157.4616	287.6029	InChI=1S/C12H7N3O/c16-12-9-6-14-11-8(2-1-5-15(11)12)7-3-4-13-10(7)9/h1-6,13H
A1-37	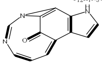	$C_{12}H_7N_3O$	8.0578	183.4658	-5.5308	68.5642	287.8516	InChI=1S/C12H7N3O/c16-12-8-2-1-4-13-6-15-10(11(12)15)9-7(8)3-5-14-9/h1-6,14H/b4-1-,8-2-,13-6-
H11-88	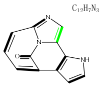	$C_{12}H_7N_3O$	4.6197	123.2683	-1.0363	135.6416	287.979	InChI=1S/C12H7N3O/c16-12-8-2-1-3-10-14-6-9(15(10)12)11-7(8)4-5-13-11/h1-6,13H
s11-64	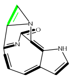	$C_{12}H_7N_3O$	18.5167	147.6865	-12.5738	96.2652	288.0306	InChI=1S/C12H7N3O/c16-12-11-10-7(4-5-13-10)2-1-3-8(14-12)9-6-15(9)11/h1-6,13H/b3-1-,7-2-,11-10-
A-110	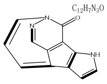	$C_{12}H_7N_3O$	13.6224	171.2839	-3.7172	76.226	288.1044	InChI=1S/C12H7N3O/c16-12-10-9-6-14-15(12)5-1-2-7(9)8-3-4-13-11(8)10/h1-6,13H/b5-1-,7-2-
C-27	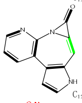	$C_{12}H_7N_3O$	22.5306	196.0763	-9.7045	67.1223	288.3716	InChI=1S/C12H7N3O/c16-12-10-6-9-7(3-5-13-9)8-2-1-4-14-11(8)15(10)12/h1-6,13H
x3-15	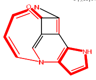	$C_{12}H_7N_3O$	7.8575	102.2105	-2.1166	146.8784	288.5054	InChI=1S/C12H7N3O/c16-12-7-6-15-5-1-3-13-11(12)9(7)10-8(15)2-4-14-10/h1-6,14H/b5-1-,13-3-
n-196	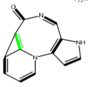	$C_{12}H_7N_3O$	8.5974	198.7193	-6.8282	68.6978	288.5113	InChI=1S/C12H7N3O/c16-12-10-7-2-1-5-15(11(7)10)9-3-4-13-8(9)6-14-12/h1-6,13H/b14-6-
F-49	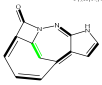	$C_{12}H_7N_3O$	10.1085	136.7281	-4.3294	119.4384	288.7137	InChI=1S/C12H7N3O/c16-12-9-3-1-2-7-6-10(9)15(12)14-11-8(7)4-5-13-11/h1-6H,(H,13,14)
B-210	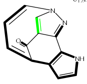	$C_{12}H_7N_3O$	9.5604	52.6997	-1.7961	194.9800	288.7293	InChI=1S/C12H7N3O/c16-12-8-2-1-5-15-6-9(12)11(14-15)10-7(8)3-4-13-10/h1-6,13H/b5-1-,8-2-
B-161	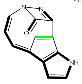	$C_{12}H_7N_3O$	7.0404	158.9899	-1.9899	103.8464	288.9494	InChI=1S/C12H7N3O/c16-12-11-9-6-7(2-1-5-15(12)14-11)8-3-4-13-10(8)9/h1-6,13H/b5-1-,7-2+
q-25	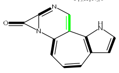	$C_{12}H_7N_3O$	12.3965	226.0416	-4.1159	47.6053	288.9612	InChI=1S/C12H7N3O/c16-12-11-14-6-8-9(15(11)12)3-1-2-7-4-5-13-10(7)8/h1-6,13H
H11-71	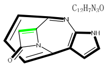	$C_{12}H_7N_3O$	5.9359	135.2562	-1.5825	138.6355	288.9812	InChI=1S/C12H7N3O/c16-11-6-10-8-2-1-3-9(15(10)11)7-4-5-13-12(7)14-8/h1-6H,(H,13,14)
G-47	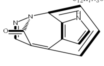	$C_{12}H_7N_3O$	7.0575	39.1919	-2.9015	209.7581	289.1149	InChI=1S/C12H7N3O/c16-12-9-6-14-15(12)10-3-1-2-7(9)8-4-5-13-11(8)10/h1-6,13H

B-229	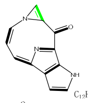 $C_{12}H_7N_3O$	17.6381	171.6536	-12.6392	86.4873	289.1327	InChI=1S/C12H7N3O/c16-12-9-6-15(9)5-1-2-8-7-3-4-13-10(7)11(12)14-8/h1-6,13H/b5-1-,8-2-
B-160	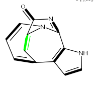 $C_{12}H_7N_3O$	7.7494	35.2983	-2.4813	207.1461	289.1777	InChI=1S/C12H7N3O/c16-12-9-6-7-2-1-5-15(9)11(14-12)10-8(7)3-4-13-10/h1-6,13H
H11-128	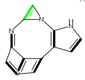 $C_{12}H_7N_3O$	21.8027	140.4274	-11.416	107.0879	289.2247	InChI=1S/C12H7N3O/c16-11-7-2-1-3-9(11)14-10-6-15(10)12-8(7)4-5-13-12/h1-6,13H/b14-9-
r-61	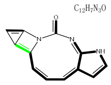 $C_{12}H_7N_3O$	15.0662	261.0521	-13.0769	20.8317	289.2957	InChI=1S/C12H7N3O/c16-12-14-11-7(4-5-13-11)2-1-3-9-8-6-10(8)15(9)12/h1-6H,(H,13,14,16)/b3-1-,7-2-
p1-58	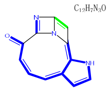 $C_{12}H_7N_3O$	6.0689	98.6288	-3.448	159.6167	289.4837	InChI=1S/C12H7N3O/c16-9-3-1-2-7-4-5-13-11(7)8-6-10-14-12(9)15(8)10/h1-6,13H/b3-1-,7-2-,11-8+
X23-18	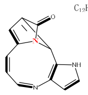 $C_{12}H_7N_3O$	12.588	150.9674	-6.6538	122.0965	289.6586	InChI=1S/C12H7N3O/c16-12-8-6-7-2-1-4-13-9-3-5-14-10(9)11(8)15(7)12/h1-6,14H/b2-1-,4-1-,7-2-,11-10+,13-4-,13-9-
o-247	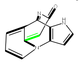 $C_{12}H_7N_3O$	5.3963	29.0694	-2.2083	218.5866	289.7053	InChI=1S/C12H7N3O/c16-10-6-9-7-2-1-5-15(9)8-3-4-13-12(8)11(7)14-10/h1-6,13H
H1-12	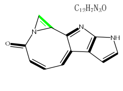 $C_{12}H_7N_3O$	13.6232	233.6199	-3.9258	33.2125	289.7779	InChI=1S/C12H7N3O/c16-10-3-1-2-7-8-4-5-13-12(8)14-11(7)9-6-15(9)10/h1-6,13H/b3-1-,7-2-
H-75	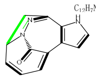 $C_{12}H_7N_3O$	7.3619	99.6933	-1.7569	149.2065	289.913	InChI=1S/C12H7N3O/c16-12-9-3-1-2-7-6-10(14-15(7)12)11-8(9)4-5-13-11/h1-6,13H
A2-28	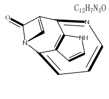 $C_{12}H_7N_3O$	7.0864	79.7175	-2.6946	178.7574	290.0521	InChI=1S/C12H7N3O/c16-12-8-6-15(12)9-2-1-4-13-11(8)10-7(9)3-5-14-10/h1-6,14H/b2-1-,4-1-,9-2+,13-4-,13-11-
H1-8	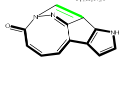 $C_{12}H_7N_3O$	5.3078	110.2044	-1.5256	154.5449	290.3109	InChI=1S/C12H7N3O/c16-10-3-1-2-7-8-4-5-13-11(8)9-6-15(10)14-12(7)9/h1-6,13H/b3-1-,7-2-
n-154	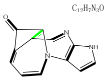 $C_{12}H_7N_3O$	8.1594	107.1358	-2.5207	139.1281	290.5949	InChI=1S/C12H7N3O/c16-11-7-2-1-5-15-9-3-4-13-12(9)14-6-8(7)10(11)15/h1-6,13H
A1-27	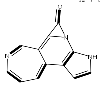 $C_{12}H_7N_3O$	14.3706	228.9544	-4.5298	46.8009	290.6989	InChI=1S/C12H7N3O/c16-12-10-9-6-13-4-1-2-7(9)8-3-5-14-11(8)15(10)12/h1-6,14H
H1-137	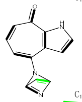 $C_{12}H_7N_3O$	28.0223	129.3267	-12.569	116.8018	290.7083	InChI=1S/C12H7N3O/c16-9-3-1-2-8(7-4-5-13-12(7)9)15-10-6-11(15)14-10/h1-6,13H
B-112	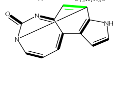 $C_{12}H_7N_3O$	6.1615	85.8902	-2.8633	173.4481	290.7209	InChI=1S/C12H7N3O/c16-12-14-9-6-10-11-8(3-4-13-11)7(9)2-1-5-15(10)12/h1-6,13H/b5-1-,7-2-
x6-38	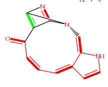 $C_{12}H_7N_3O$	32.714	141.7503	-11.8596	89.7757	290.8523	InChI=1S/C12H7N3O/c16-9-3-1-2-7-4-5-13-8(7)6-15-11-10(9)12(15)14-11/h1-6,13H/b3-1-,7-2-,8-6+
n-148	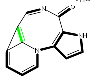 $C_{12}H_7N_3O$	10.9952	191.2585	-7.7524	66.347	290.8549	InChI=1S/C12H7N3O/c16-12-10-9(3-4-13-10)15-5-1-2-7-8(6-14-12)11(7)15/h1-6,13H/b14-6-
x5-8	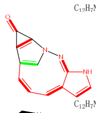 $C_{12}H_7N_3O$	32.9039	213.5949	-11.9245	41.9265	290.8942	InChI=1S/C12H7N3O/c16-11-9-8-3-1-2-7-4-5-13-12(7)14-15(6-8)10(9)11/h1-6H,(H,13,14)/b3-1-,7-2-
I-29	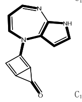 $C_{12}H_7N_3O$	25.5735	153.7118	-13.6396	104.3575	290.9358	InChI=1S/C12H7N3O/c16-11-7-6-9(10(7)11)15-5-1-3-13-12-8(15)2-4-14-12/h1-6,14H

q-24	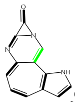 $C_{12}H_7N_3O$	10.4092	230.6242	-3.5182	46.8936	290.9757	InChI=1S/C12H7N3O/c16-12-11-14-9-3-1-2-7-4-5-13-10(7)8(9)6-15(11)12/h1-6,13H
B-141	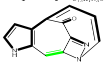 $C_{12}H_7N_3O$	9.2262	78.8221	-2.9975	171.0734	291.0696	InChI=1S/C12H7N3O/c16-12-8-2-1-5-15-10(11(12)14-15)6-9-7(8)3-4-13-9/h1-6,13H/b5-1-,8-2-
x2-14	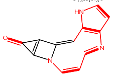 $C_{12}H_7N_3O$	44.4644	189.6656	-18.4986	61.9548	291.2005	InChI=1S/C12H7N3O/c16-12-10-9-6-8-7(2-4-14-8)13-3-1-5-15(9)11(10)12/h1-6,14H/b5-1-,9-6+,13-3+
q11-43	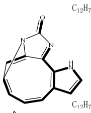 $C_{12}H_7N_3O$	4.5044	158.3045	-3.3019	117.2972	291.3085	InChI=1S/C12H7N3O/c16-12-14-11-9-6-8(15(9)12)3-1-2-7-4-5-13-10(7)11/h1-6,13H/b2-1-,8-3-
k1-17	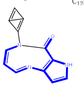 $C_{12}H_7N_3O$	17.9319	229.4727	-21.3013	50.107	291.3448	InChI=1S/C12H7N3O/c16-12-10-9(2-4-14-10)13-3-1-5-15(12)11-7-6-8(7)11/h1-6,14H/b5-1-,13-3+
o-141	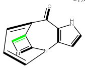 $C_{12}H_7N_3O$	3.5206	88.0862	-1.9453	174.5027	291.3469	InChI=1S/C12H7N3O/c16-11-7-2-1-5-15(12-8(7)6-14-12)9-3-4-13-10(9)11/h1-6,13H
E-71	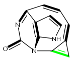 $C_{12}H_7N_3O$	20.6928	187.4377	-12.484	74.5703	291.4551	InChI=1S/C12H7N3O/c16-12-14-9-3-1-2-7-6-10(7)15(12)11-8(9)4-5-13-11/h1-6,13H/b3-1-,7-2-
C-8	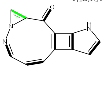 $C_{12}H_7N_3O$	12.3938	217.1513	-7.1334	47.6213	291.5055	InChI=1S/C12H7N3O/c16-12-9-6-15(9)14-4-1-2-7-8-3-5-13-11(8)10(7)12/h1-6,13H/b2-1-,14-4-
H-25	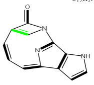 $C_{12}H_7N_3O$	9.9046	139.9782	-6.6209	126.5344	291.6181	InChI=1S/C12H7N3O/c16-12-7-2-1-3-9-8-4-5-13-10(8)11(14-9)15(12)6-7/h1-6,13H/b2-1-,9-3-
X-50	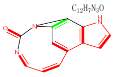 $C_{12}H_7N_3O$	12.2879	210.9911	-4.7381	48.8809	291.6769	InChI=1S/C12H7N3O/c16-12-14-4-1-2-7-6-9-11(15(9)12)10-8(7)3-5-13-10/h1-6,13H/b2-1-,14-4-
X22-20	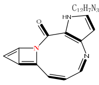 $C_{12}H_7N_3O$	13.7344	258.0083	-12.7329	24.775	291.9741	InChI=1S/C12H7N3O/c16-12-11-8(3-5-14-11)13-4-1-2-9-7-6-10(7)15(9)12/h1-6,14H/b2-1-,13-4-
X-45	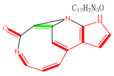 $C_{12}H_7N_3O$	6.3093	205.6155	-3.6019	61.8586	292.2587	InChI=1S/C12H7N3O/c16-12-10-9-6-7(2-1-4-14-12)8-3-5-13-11(8)15(9)10/h1-6,13H/b2-1-,14-4-
o-80	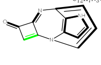 $C_{12}H_7N_3O$	3.4822	86.4737	-2.0468	177.6747	292.6788	InChI=1S/C12H7N3O/c16-10-6-9-12(10)14-7-2-1-5-15(9)8-3-4-13-11(7)8/h1-6,13H
H11-6	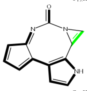 $C_{12}H_7N_3O$	7.683	268.4263	-4.4179	8.4085	292.7019	InChI=1S/C12H7N3O/c16-12-14-9-3-1-2-7(9)8-4-5-13-11(8)10-6-15(10)12/h1-6,13H/b14-9-
i-5	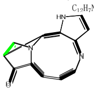 $C_{12}H_7N_3O$	8.7978	65.3071	-5.46	186.1112	292.7397	InChI=1S/C12H7N3O/c16-12-7-6-15-9(12)2-1-4-13-8-3-5-14-10(8)11(7)15/h1-6,14H/b4-1-,9-2-,11-10+,13-8-
I-69	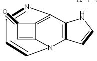 $C_{12}H_7N_3O$	3.2079	84.4988	-1.3605	180.5918	292.7656	InChI=1S/C12H7N3O/c16-9-6-8-10(9)12-11-7(2-4-14-11)15(8)5-1-3-13-12/h1-6,14H/b5-1-,13-3-
I-21	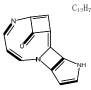 $C_{12}H_7N_3O$	8.6576	135.6167	-3.0317	122.9783	292.8707	InChI=1S/C12H7N3O/c16-12-7-6-8(12)13-3-1-5-15-9-2-4-14-10(9)11(7)15/h1-6,14H/b5-1-,11-7-,13-3-
x9-28	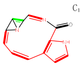 $C_{12}H_7N_3O$	21.1302	210.026	-17.2398	39.0098	292.9666	InChI=1S/C12H7N3O/c16-12-10-7(4-5-13-10)2-1-3-8-11-9(6-14-12)15(8)11/h1-6,13H/b2-1-,8-3-,14-6-
I-71	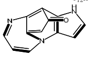 $C_{12}H_7N_3O$	5.6294	40.2371	-2.3325	217.4854	293.0702	InChI=1S/C12H7N3O/c16-9-6-8-12-10(9)11-7(2-4-14-11)15(8)5-1-3-13-12/h1-6,14H

H1-32	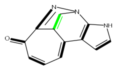 $C_{12}H_7N_3O$	5.1603	103.2288	-2.081	154.662	293.0743	InChI=1S/C12H7N30/c16-10-3-1-2-7-8-4-5-13-12(8)15-6-9(7)11(10)14-15/h1-6,13H
B-260	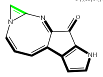 $C_{12}H_7N_3O$	8.5683	248.1879	-4.7627	9.9399	293.0793	InChI=1S/C12H7N30/c16-12-10-8(3-4-13-10)7-2-1-5-15-6-9(15)14-11(7)12/h1-6,13H/b5-1-,7-2-,14-11+
s12-21	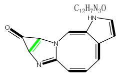 $C_{12}H_7N_3O$	31.2077	201.4535	-12.3343	61.8444	293.1498	InChI=1S/C12H7N30/c16-12-10-11(12)15-6-8-7(4-5-13-8)2-1-3-9(15)14-10/h1-6,13H/b3-1-,7-2-,8-6+
H-74	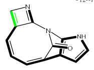 $C_{12}H_7N_3O$	4.5634	182.402	-1.5605	91.6426	293.5195	InChI=1S/C12H7N30/c16-12-9-3-1-2-7-6-14-10(7)15(12)11-8(9)4-5-13-11/h1-6,13H/b2-1-,9-3-
B-145	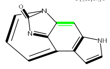 $C_{12}H_7N_3O$	5.7368	101.1529	-1.7681	166.8863	293.6345	InChI=1S/C12H7N30/c16-12-14-11-8-2-1-5-15(12)10(11)6-9-7(8)3-4-13-9/h1-6,13H
H11-159	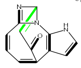 $C_{12}H_7N_3O$	7.9901	79.6953	-2.4983	187.9395	293.6772	InChI=1S/C12H7N30/c16-9-6-11-14-10-3-1-2-7(9)8-4-5-13-12(8)15(10)11/h1-6,13H/b3-1-,7-2-
B-93	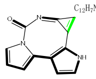 $C_{12}H_7N_3O$	11.8470	251.6173	-6.7214	21.8394	293.7612	InChI=1S/C12H7N30/c16-12-14-9-6-8(9)11-7(3-4-13-11)10-2-1-5-15(10)12/h1-6,13H/b14-9-
X23-34	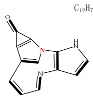 $C_{12}H_7N_3O$	32.0489	216.5674	-11.9538	47.1326	293.7919	InChI=1S/C12H7N30/c16-11-9-7-2-1-4-13-8-3-5-14-12(8)15(6-7)10(9)11/h1-6,14H/b2-1-,13-4+
s11-30	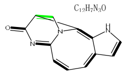 $C_{12}H_7N_3O$	6.4487	54.9346	-3.9252	200.778	293.8397	InChI=1S/C12H7N30/c16-12-8-6-15-9(14-12)3-1-2-7-4-5-13-10(7)11(8)15/h1-6,13H/b3-1-,7-2-,11-10+
o-121	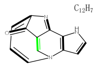 $C_{12}H_7N_3O$	10.0241	58.1264	-1.8532	187.3662	294.0756	InChI=1S/C12H7N30/c16-12-7-6-15-5-1-2-8(12)14-10(7)11-9(15)3-4-13-11/h1-6,13H/b5-1-,8-2+
x9-40	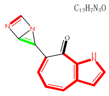 $C_{12}H_7N_3O$	11.7677	234.6095	-7.7937	34.9084	294.0771	InChI=1S/C12H7N3O/c16-11-8(10-12-14-6-15(10)12)3-1-2-7-4-5-13-9(7)11/h1-6,13H
H1-34	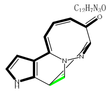 $C_{12}H_7N_3O$	5.3486	36.9389	-2.4445	219.761	294.0818	InChI=1S/C12H7N30/c16-10-3-1-2-9-7-4-5-13-11(7)8-6-15(9)14-12(8)10/h1-6,13H/b3-1-,9-2-
X-5	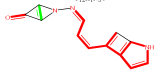 $C_{12}H_7N_3O$	44.5570	257.0095	-25.6236	4.6138	294.1319	InChI=1S/C12H7N30/c16-12-10-11(12)15(10)14-4-1-2-7-6-9-8(7)3-5-13-9/h1-6,13H/b2-1-,14-4-
s11-44	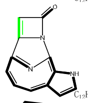 $C_{12}H_7N_3O$	8.3999	158.6513	-2.1565	119.9889	294.2659	InChI=1S/C12H7N30/c16-10-6-9-8-3-1-2-7-4-5-13-11(7)12(14-8)15(9)10/h1-6,13H/b2-1-,3-1-,7-2-,8-3-,12-11-
H11-10	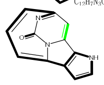 $C_{12}H_7N_3O$	8.1236	108.6759	-1.3066	148.7546	294.3151	InChI=1S/C12H7N30/c16-12-14-7-2-1-3-9-8-4-5-13-11(8)10(6-7)15(9)12/h1-6,13H/b2-1-,9-3-
A-4	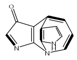 $C_{12}H_7N_3O$	8.3685	102.7647	-2.0791	161.61	294.4907	InChI=1S/C12H7N30/c16-9-6-14-12-10(9)7-2-1-5-15(12)11-8(7)3-4-13-11/h1-6,13H
H11-137	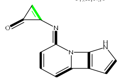 $C_{12}H_7N_3O$	44.0623	184.3229	-25.3071	79.0991	294.5293	InChI=1S/C12H7N30/c16-10-6-8(10)14-11-3-1-2-9-7-4-5-13-12(7)15(9)11/h1-6,13H/b14-11+
o-185	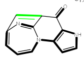 $C_{12}H_7N_3O$	6.644	92.8431	-2.3819	160.0428	294.5434	InChI=1S/C12H7N30/c16-11-8-6-7-2-1-5-15(12(8)14-7)9-3-4-13-10(9)11/h1-6,13H
o-117	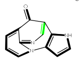 $C_{12}H_7N_3O$	5.372	37.4698	-1.98	221.4925	294.6236	InChI=1S/C12H7N30/c16-11-7-2-1-5-15-9-3-4-13-10(9)8(11)6-14-12(7)15/h1-6,13H

n-115	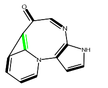	$C_{12}H_7N_3O$	11.8617	189.9637	-8.7527	79.57	294.6408	InChI=1S/C12H7N3O/c16-9-6-14-12-8(3-4-13-12)15-5-1-2-7-10(9)11(7)15/h1-6,13H/b14-6-
B-188	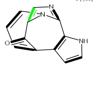	$C_{12}H_7N_3O$	9.3533	41.4057	-1.9806	210.8432	294.9903	InChI=1S/C12H7N3O/c16-11-8-2-1-5-15-9(11)6-14-12(15)10-7(8)3-4-13-10/h1-6,13H
H1-130	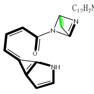	$C_{12}H_7N_3O$	20.2525	207.9265	-14.8336	78.2157	294.994	InChI=1S/C12H7N3O/c16-11(15-9-6-10(15)14-9)3-1-2-7-8-4-5-13-12(7)8/h1-6,13H/b3-1-,7-2-
q11-49	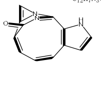	$C_{12}H_7N_3O$	21.6102	144.0373	-14.364	106.7932	295.1502	InChI=1S/C12H7N3O/c16-12-8-3-1-2-7-4-5-13-10(7)11(14-12)15-6-9(8)15/h1-6,13H/b2-1-,8-3-
x7-47	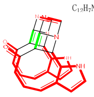	$C_{12}H_7N_3O$	4.9976	86.5004	-4.5001	192.9228	295.2358	InChI=1S/C12H7N3O/c16-8-3-1-2-7-4-5-13-10(7)11-9(8)12-14-6-15(11)12/h1-6,13H/b3-1-,7-2-,11-10-
o-29	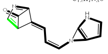	$C_{12}H_7N_3O$	14.6248	146.2098	-11.9461	122.7237	295.3225	InChI=1S/C12H7N3O/c16-11-8-6-14-10(11)7(8)2-1-5-15-9-3-4-13-12(9)15/h1-6,13H/b5-1-,7-2+
q1-46	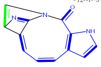	$C_{12}H_7N_3O$	11.3913	100.8202	-6.7176	174.4028	295.329	InChI=1S/C12H7N3O/c16-12-10-7(4-5-13-10)2-1-3-8-9-6-15(12)11(8)14-9/h1-6,13H/b2-1-,8-3-
G-73	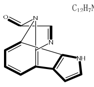	$C_{12}H_7N_3O$	8.8233	109.9891	-2.3756	157.3214	295.4664	InChI=1S/C12H7N3O/c16-10-6-14-12-8-2-1-3-9(15(10)12)11-7(8)4-5-13-11/h1-6,13H
B-113	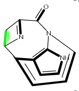	$C_{12}H_7N_3O$	7.4708	110.5517	-3.3727	155.6875	295.5028	InChI=1S/C12H7N3O/c16-12-10-6-9(14-10)7-2-1-5-15(12)11-8(7)3-4-13-11/h1-6,13H
i-19	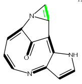	$C_{12}H_7N_3O$	19.8284	152.07	-13.223	95.1362	295.5332	InChI=1S/C12H7N3O/c16-12-8-2-1-4-13-7-3-5-14-11(7)10(12)9-6-15(8)9/h1-6,14H/b4-1-,8-2+,11-10+,13-7-
B-153	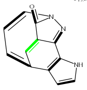	$C_{12}H_7N_3O$	6.8672	111.3130	-2.0928	147.8142	295.5493	InChI=1S/C12H7N3O/c16-12-9-6-7-2-1-5-15(12)14-11(9)10-8(7)3-4-13-10/h1-6,13H/b5-1-,7-2+
o-73	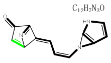	$C_{12}H_7N_3O$	14.7205	145.1396	-11.8205	125.4861	295.7119	InChI=1S/C12H7N3O/c16-10-6-8-7(11(10)14-8)2-1-5-15-9-3-4-13-12(9)15/h1-6,13H/b5-1-,7-2+
A-183	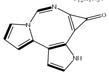	$C_{12}H_7N_3O$	31.9144	224.2051	-11.1096	25.9074	295.7537	InChI=1S/C12H7N3O/c16-12-9-10-7(3-4-13-10)8-2-1-5-15(8)6-14-11(9)12/h1-6,13H/b14-6-
G-48	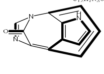	$C_{12}H_7N_3O$	6.6862	44.8034	-2.7304	211.8609	296.0592	InChI=1S/C12H7N3O/c16-12-11-7-2-1-3-9(15(12)6-14-11)10-8(7)4-5-13-10/h1-6,13H
A-156	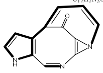	$C_{12}H_7N_3O$	24.4154	200.2425	-8.8865	50.1274	296.1775	InChI=1S/C12H7N3O/c16-11-8-2-1-5-15-10(11)12(15)14-6-9-7(8)3-4-13-9/h1-6,13H/b14-6-
A2-13	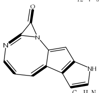	$C_{12}H_7N_3O$	20.824	220.0057	-8.6841	58.4345	296.2445	InChI=1S/C12H7N3O/c16-12-11-14-4-1-2-8-7-3-5-13-9(7)6-10(8)15(11)12/h1-6,13H/b4-1-,8-2-,14-11-
o-52	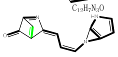	$C_{12}H_7N_3O$	14.4828	144.5772	-12.0062	125.3053	296.3553	InChI=1S/C12H7N3O/c16-11-7-6-9(11)14-8(7)2-1-5-15-10-3-4-13-12(10)15/h1-6,13H/b5-1-,8-2+
X22-14	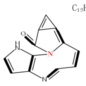	$C_{12}H_7N_3O$	15.6579	225.0955	-14.8935	53.1926	296.3706	InChI=1S/C12H7N3O/c16-12-8-6-7(8)10-2-1-4-13-9-3-5-14-11(9)15(10)12/h1-6,14H/b2-1-,13-4+
A2-48	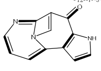	$C_{12}H_7N_3O$	7.0453	103.9001	-2.187	147.9216	296.5383	InChI=1S/C12H7N3O/c16-11-8-6-15-9(2-1-4-14-12(8)15)7-3-5-13-10(7)11/h1-6,13H

H-113	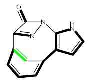	12.0377	127.6125	-4.5764	131.5908	296.6551	InChI=1S/C12H7N3O/c16-12-10-8-3-1-2-7(6-8)9-4-5-13-11(9)15(12)14-10/h1-6,13H
i-21	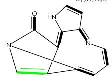	17.2459	136.032	-6.9574	135.4018	296.6672	InChI=1S/C12H7N3O/c16-12-10-7-6-15(12)9(7)2-1-4-13-8-3-5-14-11(8)10/h1-6,14H/b4-1-,9-2-,11-10-,13-8+
H11-110	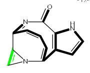	24.8109	142.9468	-15.4294	109.1981	296.8875	InChI=1S/C12H7N3O/c16-12-11-7(4-5-13-11)9-3-1-2-8(14-12)10-6-15(9)10/h1-6,13H
H1-99	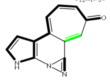	18.486	175.116	-1.9853	89.0687	297.0752	InChI=1S/C12H7N3O/c16-7-2-1-3-8-9-4-5-13-11(9)15-12(14-15)10(8)6-7/h1-6,13H
H11-54	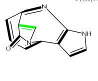	6.6152	75.7646	-3.6273	193.0825	297.1153	InChI=1S/C12H7N3O/c16-12-8-6-15(12)10-3-1-2-9(8)14-11-7(10)4-5-13-11/h1-6H,(H,13,14)
H1-64	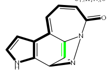	6.1788	108.2245	-2.7994	153.7978	297.1289	InChI=1S/C12H7N3O/c16-11-3-1-2-7-8-4-5-13-12(8)9-6-10(7)15(11)14-9/h1-6,13H
C-34	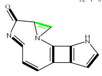	21.6078	203.4406	-10.1791	48.4315	297.1846	InChI=1S/C12H7N3O/c16-12-9-6-15(9)11-8(2-1-4-14-12)7-3-5-13-10(7)11/h1-6,13H/b2-1-,14-4+
A-105	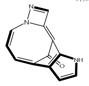	5.5083	162.3974	-1.7363	108.8302	297.2877	InChI=1S/C12H7N3O/c16-12-8-2-1-5-15-9(6-14-15)10(12)11-7(8)3-4-13-11/h1-6,13H/b5-1-,8-2-,10-9-
B-95	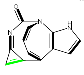	19.5405	191.1884	-14.4006	81.6326	297.2948	InChI=1S/C12H7N3O/c16-12-14-10-6-9(10)7-2-1-5-15(12)11-8(7)3-4-13-11/h1-6,13H/b14-10-
o-114	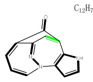	9.6353	39.2994	-1.9162	214.1047	297.428	InChI=1S/C12H7N3O/c16-12-7-2-1-5-15-10-3-4-13-11(10)8(12)6-9(7)14-15/h1-6,13H
F-53	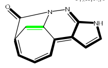	6.7207	125.6429	-1.9378	135.9501	297.434	InChI=1S/C12H7N3O/c16-12-7-2-1-3-8-9-4-5-13-11(9)14-15(12)10(8)6-7/h1-6H,(H,13,14)
H-138	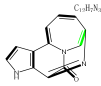	6.2768	44.9232	-1.9508	221.8401	297.4528	InChI=1S/C12H7N3O/c16-12-11-10-8(4-5-13-10)9-3-1-2-7(14-11)6-15(9)12/h1-6,13H
A1-45	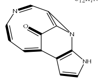	10.1659	229.8211	-4.8325	47.6967	297.5411	InChI=1S/C12H7N3O/c16-11-7-2-1-4-13-6-9-10(11)15(9)12-8(7)3-5-14-12/h1-6,14H/b4-1-,7-2-,13-6-
H11-121	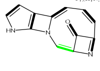	9.0685	155.9967	-2.2475	111.702	297.5727	InChI=1S/C12H7N3O/c16-11-8-2-1-3-10-7-4-5-13-12(7)15(10)6-9(11)14-8/h1-6,13H/b2-1-,9-6+,10-3-
B-258	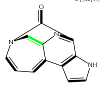	6.9446	99.3367	-1.8167	161.2175	297.5977	InChI=1S/C12H7N3O/c16-12-11-10-8(3-4-13-10)7-2-1-5-15(12)6-9(7)14-11/h1-6,13H
n-212	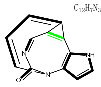	14.4712	204.7823	-5.8267	60.1729	297.607	InChI=1S/C12H7N3O/c16-12-14-6-8-7-2-1-5-15(12)9-3-4-13-11(9)10(7)8/h1-6,13H/b5-1-,7-2+,14-6-
B-94	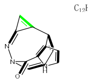	17.4231	186.9124	-11.2809	81.3505	297.7857	InChI=1S/C12H7N3O/c16-12-11-8(3-4-13-11)7-2-1-5-15(12)14-10-6-9(7)10/h1-6,13H/b5-1-,7-2+,14-10-
o-28	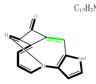	6.0495	58.9995	-3.1459	200.0194	297.9848	InChI=1S/C12H7N3O/c16-12-8-6-9-10(3-4-13-9)15-5-1-2-7(8)11(12)14-15/h1-6,13H/b8-6+
F-48	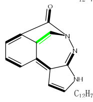	9.6974	99.1846	-3.2244	160.3447	297.9965	InChI=1S/C12H7N3O/c16-12-9-3-1-2-7-8-4-5-13-11(8)14-15(12)6-10(7)9/h1-6H,(H,13,14)

q11-55	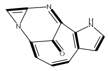 $C_{12}H_7N_3O$	12.9668	178.8999	-7.4623	77.3908	297.9996	InChI=1S/C12H7N3O/c16-12-8-3-1-2-7-4-5-13-10(7)11(12)14-9-6-15(8)9/h1-6,13H/b2-1-,8-3+
F-65	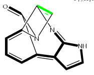 $C_{12}H_7N_3O$	6.4299	73.3604	-3.8282	207.4953	298.0671	InChI=1S/C12H7N3O/c16-12-8-2-1-3-10-9(6-15(10)12)14-11-7(8)4-5-13-11/h1-6H,(H,13,14)/b8-7-
o-116	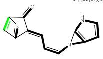 $C_{12}H_7N_3O$	14.6352	144.5564	-11.9185	127.9342	298.4505	InChI=1S/C12H7N3O/c16-11-7(8-6-9(11)14-8)2-1-5-15-10-3-4-13-12(10)15/h1-6,13H/b5-1-,7-2+
X22-28	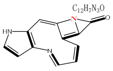 $C_{12}H_7N_3O$	16.5132	233.4715	-9.9173	51.8886	298.5332	InChI=1S/C12H7N3O/c16-12-11-7-2-1-4-13-8-3-5-14-9(8)6-10(7)15(11)12/h1-6,14H/b2-1-,10-6+,13-4+
A-85	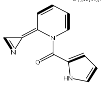 $C_{12}H_9N_3O$	2.4687	293.7689	-2.0948	5.3058	298.8915	InChI=1S/C12H9N3O/c16-12(9-4-3-6-13-9)15-7-2-1-5-11(15)10-8-14-10/h1-8,13H/b11-10-
H11-118	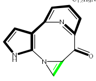 $C_{12}H_7N_3O$	11.7533	210.0306	-3.943	57.4536	298.9313	InChI=1S/C12H7N3O/c16-11-9-3-1-2-8(14-9)7-4-5-13-12(7)15-6-10(11)15/h1-6,13H
n-163	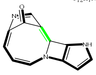 $C_{12}H_7N_3O$	5.9784	162.5785	-0.4543	107.9834	298.9669	InChI=1S/C12H7N3O/c16-12-7-6-14-8(12)2-1-5-15-9-3-4-13-10(9)11(7)15/h1-6,13H/b5-1-,8-2+,11-7-
q1-39	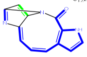 $C_{12}H_7N_3O$	13.4721	101.0377	-6.867	175.6205	299.0417	InChI=1S/C12H7N3O/c16-12-11-7(4-5-13-11)2-1-3-8-9-6-10(14-8)15(9)12/h1-6,13H/b2-1-,8-3+
n-52	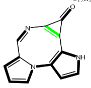 $C_{12}H_7N_3O$	32.3312	225.7906	-11.1171	25.967	299.3202	InChI=1S/C12H7N3O/c16-12-9-10-8(3-4-13-10)15-5-1-2-7(15)6-14-11(9)12/h1-6,13H/b14-6-
o-16	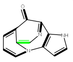 $C_{12}H_7N_3O$	5.3588	39.2434	-1.7954	221.4673	299.4435	InChI=1S/C12H7N3O/c16-12-7-2-1-5-15-8-3-4-13-10(8)11(12)14-6-9(7)15/h1-6,13H
q11-60	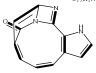 $C_{12}H_7N_3O$	6.0429	116.2057	-3.3222	166.5322	299.5979	InChI=1S/C12H7N3O/c16-12-8-3-1-2-7-4-5-13-10(7)11-14-9(6-8)15(11)12/h1-6,13H/b2-1-,3-1-,7-2-,8-3-,11-10+
H1-22	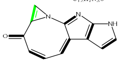 $C_{12}H_7N_3O$	14.6584	229.1632	-4.7219	38.2535	299.6162	InChI=1S/C12H7N3O/c16-10-3-1-2-8-7-4-5-13-11(7)14-12(8)15-6-9(10)15/h1-6,13H/b3-1-,8-2-
o-7	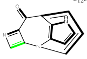 $C_{12}H_7N_3O$	3.3146	89.7637	-1.9998	181.1257	299.7106	InChI=1S/C12H7N3O/c16-12-7-2-1-5-15(9-6-14-11(9)12)8-3-4-13-10(7)8/h1-6,13H
H-126	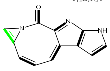 $C_{12}H_7N_3O$	22.7534	206.4993	-13.8746	63.7331	299.8536	InChI=1S/C12H7N3O/c16-12-10-8(3-1-2-7-6-15(7)12)9-4-5-13-11(9)14-10/h1-6,13H/b2-1-,8-3-
x6-32	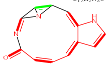 $C_{12}H_7N_3O$	27.7306	241.9086	-15.1481	23.528	299.9921	InChI=1S/C12H7N3O/c16-10-3-1-2-7-4-5-13-8(7)6-9-11-12(14-10)15(9)11/h1-6,13H/b3-1-,7-2-,8-6+,14-12-
B-209	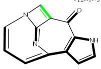 $C_{12}H_7N_3O$	7.6291	65.0075	-3.5339	200.4368	300.0352	InChI=1S/C12H7N3O/c16-11-8-6-15-5-1-2-9(14-12(8)15)7-3-4-13-10(7)11/h1-6,13H
B-124	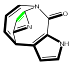 $C_{12}H_7N_3O$	7.2733	59.8454	-4.7812	211.4465	300.1014	InChI=1S/C12H7N3O/c16-12-11-8(3-4-13-11)7-2-1-5-15(12)10-6-9(7)14-10/h1-6,13H/b5-1-,7-2-
B-71	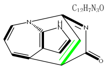 $C_{12}H_7N_3O$	7.8685	35.0593	-2.7857	215.0652	300.1431	InChI=1S/C12H7N3O/c16-12-9-6-10(14-12)15-5-1-2-7(9)8-3-4-13-11(8)15/h1-6,13H
B-142	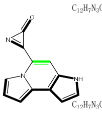 $C_{12}H_7N_3O$	20.5455	216.7062	-7.8283	46.5778	300.2691	InChI=1S/C12H7N3O/c16-12-11(14-12)10-6-8-7(3-4-13-8)9-2-1-5-15(9)10/h1-6,13H

r-44	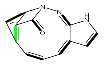	$C_{12}H_7N_3O$	9.1747	111.8845	-4.6926	152.6189	300.3489	InChI=1S/C12H7N3O/c16-12-10-8-3-1-2-7-4-5-13-11(7)14-15(12)9(10)6-8/h1-6H,(H,13,14)/b3-1-,7-2-
r-39	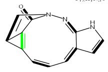	$C_{12}H_7N_3O$	15.2313	161.1512	-8.4927	115.6467	300.4079	InChI=1S/C12H7N3O/c16-12-10-8-3-1-2-7-4-5-13-11(7)14-15(12)6-9(8)10/h1-6H,(H,13,14)/b3-1-,7-2-
H1-38	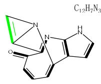	$C_{12}H_7N_3O$	20.842	169.4887	-11.5396	85.1789	300.9023	InChI=1S/C12H7N3O/c16-10-3-1-2-8-7-4-5-13-12(7)14-11(10)9-6-15(8)9/h1-6,13H/b3-1-,8-2-
B-143	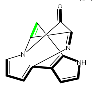	$C_{12}H_7N_3O$	7.9237	116.0139	-4.2187	153.4215	301.0019	InChI=1S/C12H7N3O/c16-12-11-10-6-9-7(3-4-13-9)8(14-11)2-1-5-15(10)12/h1-6,13H/b5-1-,8-2?
A-112	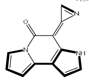	$C_{12}H_7N_3O$	2.5761	304.6855	-2.3943	-4.24	301.0355	InChI=1S/C12H7N3O/c16-12-10(8-6-14-8)11-7(3-4-13-11)9-2-1-5-15(9)12/h1-6,13H/b10-8+
o-125	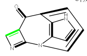	$C_{12}H_7N_3O$	3.4669	90.7347	-2.1484	184.6101	301.1951	InChI=1S/C12H7N3O/c16-11-7-2-1-5-15(12-8(11)6-14-12)9-3-4-13-10(7)9/h1-6,13H
x4-3	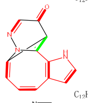	$C_{12}H_7N_3O$	6.4604	41.5972	-5.2892	223.7605	301.4445	InChI=1S/C12H7N3O/c16-9-6-14-15-8-3-1-2-7-4-5-13-11(7)12(15)10(8)9/h1-6,13H/b2-1-,3-1-,7-2-,8-3+,12-11+
H11-148	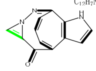	$C_{12}H_7N_3O$	13.8752	128.2455	-11.2374	135.8671	301.4773	InChI=1S/C12H7N3O/c16-12-8-2-1-3-9(14-15-6-10(12)15)11-7(8)4-5-13-11/h1-6,13H/b14-9+
C-2	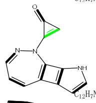	$C_{12}H_7N_3O$	40.2315	253.1360	-17.3688	11.6605	301.5344	InChI=1S/C12H7N3O/c16-10-6-9(10)15-12-8(2-1-4-14-15)7-3-5-13-11(7)12/h1-6,13H
r-34	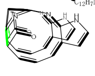	$C_{12}H_7N_3O$	8.8097	161.6291	-2.6475	124.8409	301.6949	InChI=1S/C12H7N3O/c16-12-8-3-1-2-7-4-5-13-11(7)14-10-6-9(8)15(10)12/h1-6H,(H,13,14)
X22-47	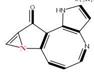	$C_{12}H_7N_3O$	19.6785	189.6369	-14.7218	89.7401	301.9421	InChI=1S/C12H7N3O/c16-12-9-6-15(9)8-2-1-4-13-7-3-5-14-11(7)10(8)12/h1-6,14H/b2-1-,4-1-,8-2+,11-10-,13-4-,13-7-
s11-35	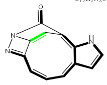	$C_{12}H_7N_3O$	6.8559	133.6553	-4.6747	135.9639	302.0117	InChI=1S/C12H7N3O/c16-12-8-6-10-9(14-15(10)12)3-1-2-7-4-5-13-11(7)8/h1-6,13H/b3-1-,7-2-,11-8-
H1-58	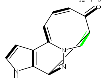	$C_{12}H_7N_3O$	4.9507	43.5491	-1.7905	221.3928	302.2293	InChI=1S/C12H7N3O/c16-10-3-1-2-9-7-4-5-13-11(7)12-8(10)6-15(9)14-12/h1-6,13H/b3-1-,9-2-
s12-17	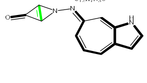	$C_{12}H_7N_3O$	40.5849	173.1501	-25.5486	87.8927	302.2481	InChI=1S/C12H7N3O/c16-12-10-11(12)15(10)14-8-3-1-2-7-4-5-13-9(7)6-8/h1-6,13H/b14-8+
x9-3	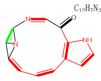	$C_{12}H_7N_3O$	20.3066	195.0032	-16.5436	60.362	302.4612	InChI=1S/C12H7N3O/c16-9-6-14-12-11-8(15(11)12)3-1-2-7-4-5-13-10(7)9/h1-6,13H/b2-1-,8-3-,14-6-
H1-40	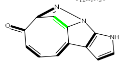	$C_{12}H_7N_3O$	7.4167	152.8202	-1.2914	121.3212	302.5034	InChI=1S/C12H7N3O/c16-11-3-1-2-7-8-4-5-13-12(8)15-10(7)6-9(11)14-15/h1-6,13H/b3-1-,7-2-
n-127	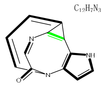	$C_{12}H_7N_3O$	11.1374	219.357	-3.7543	51.0967	302.5613	InChI=1S/C12H7N3O/c16-9-6-14-11-7-2-1-5-15(9)8-3-4-13-12(8)10(7)11/h1-6,13H/b5-1-,7-2-,14-6-
B-83	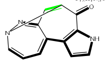	$C_{12}H_7N_3O$	7.5012	37.0390	-2.2485	218.6705	302.5676	InChI=1S/C12H7N3O/c16-12-10-6-9-7(2-1-5-15(10)14-9)8-3-4-13-11(8)12/h1-6,13H
X22-54	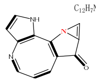	$C_{12}H_7N_3O$	19.2882	188.397	-14.5791	91.3053	302.646	InChI=1S/C12H7N3O/c16-12-7-2-1-4-13-8-3-5-14-10(8)11(7)15-6-9(12)15/h1-6,14H/b2-1-,4-1-,7-2+,11-10-,13-4-,13-8-

H-9	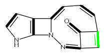	8.794	148.9752	-3.2451	128.2602	302.6714	InChI=1S/C12H7N3O/c16-11-7-2-1-3-10-8-4-5-13-12(8)15(10)14-9(11)6-7/h1-6,13H/b2-1-,10-3-,14-9-
H11-86	$C_{12}H_7N_3O$ 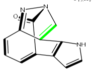	7.3353	41.4689	-3.0228	221.087	302.8328	InChI=1S/C12H7N3O/c16-12-8-2-1-3-10-9(6-15(12)14-10)11-7(8)4-5-13-11/h1-6,13H/b3-1-,8-2-
s11-63	$C_{12}H_7N_3O$ 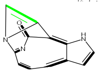	20.0209	138.1235	-13.7646	115.304	302.8552	InChI=1S/C12H7N3O/c16-12-10-8-6-15(8)9(14-12)3-1-2-7-4-5-13-11(7)10/h1-6,13H/b3-1-,7-2-,11-10+
I-80	$C_{12}H_7N_3O$ 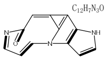	15.5783	194.0006	-7.7059	78.5793	302.9181	InChI=1S/C12H7N3O/c16-9-6-7-10-11-8(2-4-14-11)15(12(7)10)5-1-3-13-9/h1-6,14H/b5-1+,7-6-,13-3-
s12-46	$C_{12}H_7N_3O$ 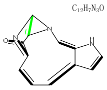	10.1718	178.9314	-6.6244	86.38	303.2843	InChI=1S/C12H7N3O/c16-11-8-3-1-2-7-4-5-13-9(7)6-15-10(11)12(15)14-8/h1-6,13H/b3-1-,7-2-,9-6-
G-22	$C_{12}H_7N_3O$ 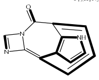	5.4373	135.5597	-2.0593	151.1189	303.5955	InChI=1S/C12H7N3O/c16-12-9-3-1-2-8(11-14-6-15(11)12)7-4-5-13-10(7)9/h1-6,13H
A2-11	$C_{12}H_7N_3O$ 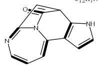	4.6757	95.9807	-2.2352	182.6854	303.6264	InChI=1S/C12H7N3O/c16-12-8-6-10-13-4-1-2-9(15(10)12)7-3-5-14-11(7)8/h1-6,14H
H11-43	$C_{12}H_7N_3O$ 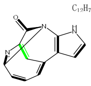	7.4931	43.2313	-2.9238	222.7176	303.6958	InChI=1S/C12H7N3O/c16-12-9-6-7-2-1-3-10(14-9)15(12)11-8(7)4-5-13-11/h1-6,13H/b2-1-,3-1-,7-2+,10-3-
X22-39	$C_{12}H_7N_3O$ 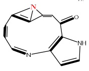	30.021	249.9632	-15.4549	20.7086	303.7422	InChI=1S/C12H7N3O/c16-10-6-9-12-8(15(9)12)2-1-4-13-7-3-5-14-11(7)10/h1-6,14H/b2-1-,9-6+,13-4-
s12-44	$C_{12}H_7N_3O$ 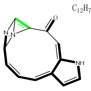	10.5576	220.9262	-7.8409	55.6651	304.045	InChI=1S/C12H7N3O/c16-9-6-8-7(4-5-13-8)2-1-3-10-14-12-11(9)15(10)12/h1-6,13H/b3-1-,7-2-,8-6+
H11-78	$C_{12}H_7N_3O$ 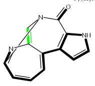	7.3305	44.4049	-3.2601	218.1927	304.2572	InChI=1S/C12H7N3O/c16-12-11-8(4-5-13-11)7-2-1-3-10-14-9(7)6-15(10)12/h1-6,13H
H11-93	$C_{12}H_7N_3O$ 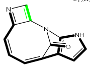	4.9465	185.128	-1.6694	98.7347	304.3009	InChI=1S/C12H7N3O/c16-12-8-2-1-3-9-10(6-14-9)15(12)11-7(8)4-5-13-11/h1-6,13H/b3-1-,8-2-
B-10	$C_{12}H_7N_3O$ 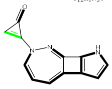	41.4955	254.0969	-17.8805	11.8608	304.4054	InChI=1S/C12H7N3O/c16-10-6-9(10)15-5-1-2-7-8-3-4-13-11(8)12(7)14-15/h1-6,13H
k-8	$C_{12}H_7N_3O$ 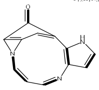	26.418	158.8026	-7.2203	80.5648	304.5097	InChI=1S/C12H7N3O/c16-12-7-6-9-11(12)15(9)5-1-3-13-8-2-4-14-10(7)8/h1-6,14H/b5-1-,13-3-
I-84	$C_{12}H_7N_3O$ 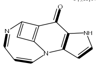	9.7814	72.3471	-3.7419	186.2711	304.7295	InChI=1S/C12H7N3O/c16-12-10-7-6-9(10)15(5-1-3-13-7)8-2-4-14-11(8)12/h1-6,14H/b5-1-,13-3-
o-162	$C_{12}H_7N_3O$ 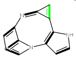	16.7362	207.4445	-10.2922	69.3679	304.862	InChI=1S/C12H7N3O/c16-12-8-2-1-5-15(12)10-3-4-13-11(10)7-6-9(7)14-8/h1-6,13H/b14-9-
n-190	$C_{12}H_7N_3O$ 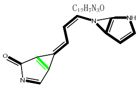	19.5651	220.5445	-14.3328	49.744	304.9913	InChI=1S/C12H7N3O/c16-12-10-7(8(10)6-14-12)2-1-5-15-9-3-4-13-11(9)15/h1-6,13H/b5-1-,7-2-
n-101	$C_{12}H_7N_3O$ 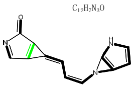	19.5658	220.5332	-14.3324	49.7448	304.9966	InChI=1S/C12H7N3O/c16-12-10-7(8(10)6-14-12)2-1-5-15-9-3-4-13-11(9)15/h1-6,13H/b5-1-,7-2+
B-99	$C_{12}H_7N_3O$ 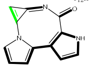	13.4720	250.1730	-7.7562	27.7937	305.1494	InChI=1S/C12H7N3O/c16-12-11-7(3-4-13-11)9-2-1-5-15(9)10-6-8(10)14-12/h1-6,13H/b14-8-
	$C_{12}H_7N_3O$						

E-55	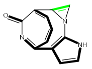 $C_{12}H_7N_3O$	29.0313	155.8931	-17.3916	104.0143	305.169	InChI=1S/C12H7N3O/c16-12-8-2-1-3-9(14-12)7-4-5-13-11(7)15-6-10(8)15/h1-6,13H
n-7	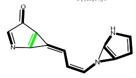 $C_{12}H_7N_3O$	18.9768	222.2016	-14.1298	49.7678	305.3636	InChI=1S/C12H7N3O/c16-9-6-14-11-7(10(9)11)2-1-5-15-8-3-4-13-12(8)15/h1-6,13H/b5-1-,7-2-
A1-50	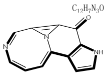 $C_{12}H_7N_3O$	26.9993	219.4469	-13.2157	37.4615	305.5073	InChI=1S/C12H7N3O/c16-12-10-7(3-5-14-10)8-2-1-4-13-6-9-11(12)15(8)9/h1-6,14H/b4-1-,8-2-,13-6-
H1-39	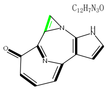 $C_{12}H_7N_3O$	19.6153	188.8091	-10.0866	79.9601	305.5401	InChI=1S/C12H7N3O/c16-10-3-1-2-8-7-4-5-13-12(7)15-6-9(15)11(10)14-8/h1-6,13H
G1-81	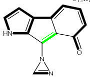 $C_{12}H_7N_3O$	1.8677	311.2616	-2.0541	-8.83	305.589	InChI=1S/C12H7N3O/c16-9-3-1-2-7-8-4-5-13-11(8)12(10(7)9)15-6-14-15/h1-6,13H
H11-98	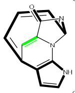 $C_{12}H_7N_3O$	8.3586	133.4658	-0.7802	134.8468	305.8243	InChI=1S/C12H7N3O/c16-12-9-6-7-2-1-3-10(14-12)15(9)11-8(7)4-5-13-11/h1-6,13H/b3-1-,7-2+
H11-124	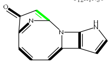 $C_{12}H_7N_3O$	10.2981	201.26	-0.3261	66.6895	306.2006	InChI=1S/C12H7N3O/c16-10-6-11-14-8(10)2-1-3-9-7-4-5-13-12(7)15(9)11/h1-6,13H/b2-1-,9-3-
G-35	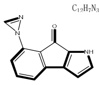 $C_{12}H_7N_3O$	1.9851	310.9121	-1.9498	-9.89	306.3266	InChI=1S/C12H7N3O/c16-12-10-7(8-4-5-13-11(8)12)2-1-3-9(10)15-6-14-15/h1-6,13H
x6-34	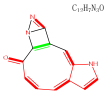 $C_{12}H_7N_3O$	23.7337	156.1097	-9.1143	107.2446	306.5943	InChI=1S/C12H7N3O/c16-10-3-1-2-7-4-5-13-9(7)6-8-11(10)15-12(8)14-15/h1-6,13H/b3-1-,7-2-,9-6+
r-57	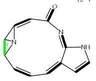 $C_{12}H_7N_3O$	31.2949	250.4438	-15.5206	19.774	306.6803	InChI=1S/C12H7N3O/c16-10-6-9-11-8(15(9)11)3-1-2-7-4-5-13-12(7)14-10/h1-6H,(H,13,14,16)
A1-51	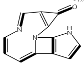 $C_{12}H_7N_3O$	34.5919	248.2518	-17.3154	23.0055	306.7655	InChI=1S/C12H7N3O/c16-11-8-6-13-4-1-2-9-7-3-5-14-12(7)15(9)10(8)11/h1-6,14H/b4-1-,9-2-,13-6+
o-10	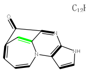 $C_{12}H_7N_3O$	6.7797	110.1949	-1.4068	152.7016	306.8749	InChI=1S/C12H7N3O/c16-11-7-2-1-5-15-8-3-4-13-12(8)14-10(11)9(15)6-7/h1-6,13H
C-16	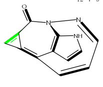 $C_{12}H_7N_3O$	18.9666	197.6738	-14.4028	79.0292	307.0189	InChI=1S/C12H7N3O/c16-12-10-6-9(10)7-2-1-4-14-15(12)11-8(7)3-5-13-11/h1-6,13H/b2-1-,14-4-
k-19	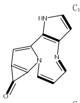 $C_{12}H_7N_3O$	30.4342	225.9135	-12.0752	55.6604	307.0989	InChI=1S/C12H7N3O/c16-12-7-6-9-10-8(2-4-14-10)13-3-1-5-15(9)11(7)12/h1-6,14H/b5-1-,13-3+
C-6	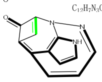 $C_{12}H_7N_3O$	8.0074	64.1834	-4.5520	202.2741	307.2047	InChI=1S/C12H7N3O/c16-11-9-6-10(11)15-12-8(3-5-13-12)7(9)2-1-4-14-15/h1-6,13H/b2-1-,14-4-
A2-42	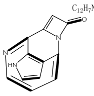 $C_{12}H_7N_3O$	12.4027	163.8404	-2.0406	114.4077	307.2167	InChI=1S/C12H7N3O/c16-10-6-9-12-11-7(3-5-14-11)8(15(9)10)2-1-4-13-12/h1-6,14H/b2-1-,4-1-,8-2+,13-4-,13-12-
A-37	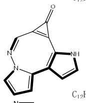 $C_{12}H_7N_3O$	29.2812	227.6797	-10.7759	35.6279	307.2906	InChI=1S/C12H7N3O/c16-12-8-6-14-15-5-1-2-9(15)7-3-4-13-11(7)10(8)12/h1-6,13H/b14-6-
n-177	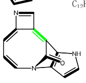 $C_{12}H_7N_3O$	5.8192	180.2048	-2.1477	103.3098	307.5775	InChI=1S/C12H7N3O/c16-12-10-7-6-14-8(7)2-1-5-15(12)9-3-4-13-11(9)10/h1-6,13H/b5-1-,8-2+,10-7-
H1-3	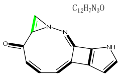 $C_{12}H_7N_3O$	10.6413	232.0947	-6.4387	49.5121	307.7018	InChI=1S/C12H7N3O/c16-10-3-1-2-7-8-4-5-13-11(8)12(7)14-15-6-9(10)15/h1-6,13H/b3-1-,7-2-,14-12+

A-107	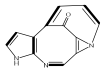 $C_{12}H_7N_3O$	24.0724	209.0615	-8.7431	54.245	307.7393	InChI=1S/C12H7N3O/c16-11-7-2-1-5-15-9(10(11)15)6-14-12-8(7)3-4-13-12/h1-6,13H/b14-6-
x6-39	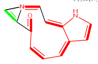 $C_{12}H_7N_3O$	20.5695	236.8543	-10.214	44.2202	307.8399	InChI=1S/C12H7N3O/c16-9-3-1-2-7-4-5-13-8(7)6-10-14-12-11(9)15(10)12/h1-6,13H/b3-1-,7-2-,8-6-
x4-33	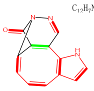 $C_{12}H_7N_3O$	10.4858	138.1653	-4.4392	134.0543	307.8787	InChI=1S/C12H7N3O/c16-12-10-8-6-14-15(12)9(10)3-1-2-7-4-5-13-11(7)8/h1-6,13H/b2-1-,3-1-,7-2-,9-3+,11-8+
H-108	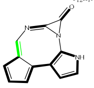 $C_{12}H_7N_3O$	18.1378	217.4184	-7.3148	61.0241	307.9327	InChI=1S/C12H7N3O/c16-12-11-14-6-7-2-1-3-8(7)9-4-5-13-10(9)15(11)12/h1-6,13H/b7-6-,14-11-
n-36	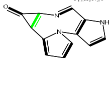 $C_{12}H_7N_3O$	26.6206	224.327	-10.1106	50.3647	308.1897	InChI=1S/C12H7N3O/c16-12-10-9-2-1-5-15(9)8-3-4-13-7(8)6-14-11(10)12/h1-6,13H/b14-6+
p11-13	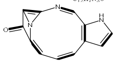 $C_{12}H_7N_3O$	13.4443	227.119	-8.2575	53.2706	308.2052	InChI=1S/C12H7N3O/c16-11-9-3-1-2-7-4-5-13-8(7)6-14-12-10(11)15(9)12/h1-6,13H/b2-1-,9-3-,14-6-
o-67	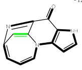 $C_{12}H_7N_3O$	6.9898	112.3869	-1.3514	148.2508	308.2365	InChI=1S/C12H7N3O/c16-12-10-8(3-4-13-10)15-5-1-2-7-6-9(15)11(12)14-7/h1-6,13H
B-18	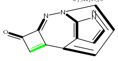 $C_{12}H_7N_3O$	6.5229	144.6333	-2.0717	127.7113	308.2606	InChI=1S/C12H7N3O/c16-10-6-9-7-2-1-5-15(14-11(9)10)12-8(7)3-4-13-12/h1-6,13H
B-63	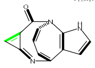 $C_{12}H_7N_3O$	16.1107	153.8981	-13.4576	130.0974	308.3420	InChI=1S/C12H7N3O/c16-12-8-6-10(8)14-9-2-1-5-15(12)11-7(9)3-4-13-11/h1-6,13H/b14-10-
q-54	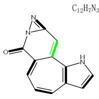 $C_{12}H_7N_3O$	18.2331	201.0827	-3.348	81.7647	308.3466	InChI=1S/C12H7N3O/c16-12-8-3-1-2-7-4-5-13-11(7)9(8)6-10-14-15(10)12/h1-6,13H
r-55	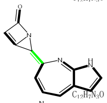 $C_{12}H_7N_3O$	10.6421	262.9812	-6.3266	35.5792	308.3575	InChI=1S/C12H7N3O/c16-10-6-9-11(15(9)10)8-3-1-2-7-4-5-13-12(7)14-8/h1-6H,(H,13,14)/b11-8-
H-72	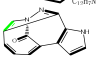 $C_{12}H_7N_3O$	7.0436	43.349	-2.2995	228.6278	308.4244	InChI=1S/C12H7N3O/c16-12-9-3-1-2-7-6-15(12)14-10(7)11-8(9)4-5-13-11/h1-6,13H/b2-1-,9-3-
H-48	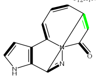 $C_{12}H_7N_3O$	5.5311	99.8454	-2.2335	176.073	308.4907	InChI=1S/C12H7N3O/c16-10-6-7-2-1-3-9-8-4-5-13-12(8)11(7)14-15(9)10/h1-6,13H/b2-1-,9-3-
G1-84	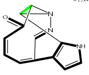 $C_{12}H_7N_3O$	5.3865	187.0784	-3.431	94.5286	308.6075	InChI=1S/C12H7N3O/c16-9-3-1-2-7-6-14-15-11(9)12(15)10-8(7)4-5-13-10/h1-6,13H/b3-1-,7-2+
x4-57	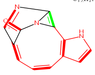 $C_{12}H_7N_3O$	18.4226	242.8169	-4.4932	36.7144	308.7869	InChI=1S/C12H7N3O/c16-12-8-3-1-2-7-4-5-13-9(7)10-11(14-6-8)15(10)12/h1-6,13H/b2-1-,3-1-,7-2-,8-3-,10-9+
B-4	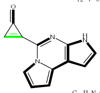 $C_{12}H_7N_3O$	10.3668	303.4465	-3.2479	-3.9087	308.7922	InChI=1S/C12H7N3O/c16-10-6-8(10)12-14-11-7(3-4-13-11)9-2-1-5-15(9)12/h1-6,13H
s12-39	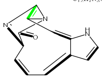 $C_{12}H_7N_3O$	14.7554	190.8557	-6.4966	81.1357	308.8113	InChI=1S/C12H7N3O/c16-12-8-3-1-2-7-4-5-13-9(7)6-10-11(14-8)15(10)12/h1-6,13H/b2-1-,3-1-,7-2-,8-3+,9-6-,10-6-
E-17	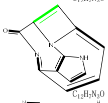 $C_{12}H_7N_3O$	5.5457	79.9682	-1.908	193.8849	308.8335	InChI=1S/C12H7N3O/c16-12-10-6-7-2-1-3-9(14-12)8-4-5-13-11(8)15(7)10/h1-6,13H/b3-1-,7-2-
o-215	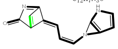 $C_{12}H_7N_3O$	14.7878	143.457	-12.4736	138.6018	308.8979	InChI=1S/C12H7N3O/c16-12-8-6-9(14-12)7(8)2-1-5-15-10-3-4-13-11(10)15/h1-6,13H/b5-1-,7-2+

n-132	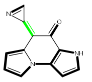 $C_{12}H_7N_3O$	2.4709	304.0531	-2.2557	-3.31	308.928	InChI=1S/C12H7N3O/c16-12-10(7-6-14-7)8-2-1-5-15(8)9-3-4-13-11(9)12/h1-6,13H/b10-7+
E-42	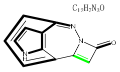 $C_{12}H_7N_3O$	8.2856	144.3312	-2.6235	117.6	308.9398	InChI=1S/C12H7N3O/c16-11-6-10-8-2-1-3-9(14-15(10)11)7-4-5-13-12(7)8/h1-6,13H
H1-69	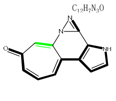 $C_{12}H_7N_3O$	17.7727	173.9	-2.2712	100.868	308.9584	InChI=1S/C12H7N3O/c16-7-2-1-3-8-9-4-5-13-11(9)12-14-15(12)10(8)6-7/h1-6,13H
p-27	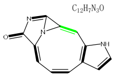 $C_{12}H_7N_3O$	10.671	137.7607	-7.1259	139.2835	309.412	InChI=1S/C12H7N3O/c16-12-9-3-1-2-7-4-5-13-8(7)6-10-11(14-12)15(9)10/h1-6,13H/b2-1-,9-3-,10-6-
H1-61	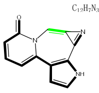 $C_{12}H_7N_3O$	18.4604	160.361	-4.5148	117.174	309.6399	InChI=1S/C12H7N3O/c16-10-3-1-2-9-7-4-5-13-11(7)12-8(14-12)6-15(9)10/h1-6,13H
H11-77	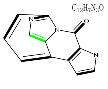 $C_{12}H_7N_3O$	5.384	111.0578	-1.6609	167.7345	309.7541	InChI=1S/C12H7N3O/c16-12-11-8(4-5-13-11)7-2-1-3-10-14-6-9(7)15(10)12/h1-6,13H
q-20	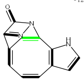 $C_{12}H_7N_3O$	7.7586	151.8445	-2.8499	105.595	309.8593	InChI=1S/C12H7N3O/c16-12-11-8-3-1-2-7-4-5-13-10(7)9(6-8)15(12)14-11/h1-6,13H/b2-1-,3-1-,7-2-,8-3+,10-9-
o-188	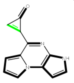 $C_{12}H_7N_3O$	10.3032	302.596	-3.2276	-3.9779	309.8834	InChI=1S/C12H7N3O/c16-10-6-7(10)11-8-2-1-5-15(8)9-3-4-13-12(9)14-11/h1-6,13H
F-61	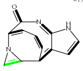 $C_{12}H_7N_3O$	20.0377	139.1646	-13.1334	120.613	310.295	InChI=1S/C12H7N3O/c16-12-9-3-1-2-7(10-6-15(9)10)8-4-5-13-11(8)14-12/h1-6H,(H,13,14,16)
I-63	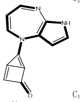 $C_{12}H_7N_3O$	12.0989	265.2713	-9.7472	26.4171	310.3671	InChI=1S/C12H7N3O/c16-9-6-7-10(9)11(7)15-5-1-3-13-12-8(15)2-4-14-12/h1-6,14H
A-59	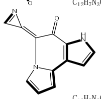 $C_{12}H_7N_3O$	2.7376	304.7561	-2.2573	-4.31	310.4219	InChI=1S/C12H7N3O/c16-12-10-7(3-4-13-10)9-2-1-5-15(9)11(12)8-6-14-8/h1-6,13H/b11-8+
A-103	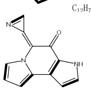 $C_{12}H_7N_3O$	2.737	304.7563	-2.2566	-4.31	310.4219	InChI=1S/C12H7N3O/c16-12-10-7(3-4-13-10)9-2-1-5-15(9)11(12)8-6-14-8/h1-6,13H/b11-8-
B-1	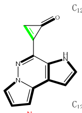 $C_{12}H_7N_3O$	10.3706	304.5912	-3.2159	-4.8400	310.4467	InChI=1S/C12H7N3O/c16-10-6-8(10)12-11-7(3-4-13-11)9-2-1-5-15(9)14-12/h1-6,13H
x6-35	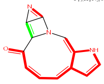 $C_{12}H_7N_3O$	23.0123	151.9938	-10.935	118.592	310.4601	InChI=1S/C12H7N3O/c16-9-3-1-2-7-4-5-13-8(7)6-15-11(9)10-12(15)14-10/h1-6,13H/b3-1-,7-2-,8-6+
C-5	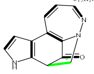 $C_{12}H_7N_3O$	4.7265	104.6521	-2.1768	172.1481	310.7083	InChI=1S/C12H7N3O/c16-12-9-6-15-11(12)8(2-1-4-14-15)7-3-5-13-10(7)9/h1-6,13H
H1-103	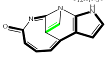 $C_{12}H_7N_3O$	6.8398	88.0435	-4.8906	189.5491	310.8708	InChI=1S/C12H7N3O/c16-10-3-1-2-7-8-4-5-13-11(8)15-6-9(7)12(15)14-10/h1-6,13H/b3-1-,7-2-,14-12+
A2-70	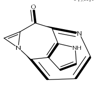 $C_{12}H_7N_3O$	16.7605	141.4378	-11.7505	119.1067	311.096	InChI=1S/C12H7N3O/c16-12-9-6-15(9)8-2-1-4-13-11(12)10-7(8)3-5-14-10/h1-6,14H/b2-1-,4-1-,8-2+,13-4-,13-11+
I-33	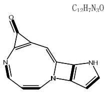 $C_{12}H_7N_3O$	31.0903	251.7363	-15.2745	22.6369	311.3173	InChI=1S/C12H7N3O/c16-12-7-6-9-11-8(2-4-14-11)15(9)5-1-3-13-10(7)12/h1-6,14H/b5-1-,9-6-,13-3-
n-186	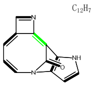 $C_{12}H_7N_3O$	5.2656	180.5498	-1.8871	106.1715	311.3351	InChI=1S/C12H7N3O/c16-12-9-10-7(6-14-10)2-1-5-15(12)8-3-4-13-11(8)9/h1-6,13H/b5-1-,7-2-,10-9+

o-196	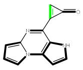	10.292	303.4448	-3.2463	-1.9195	311.3793	InChI=1S/C12H7N3O/c16-9-6-7(9)11-12-8(3-4-13-12)15-5-1-2-10(15)14-11/h1-6,13H
G1-113	$C_{12}H_7N_3O$ 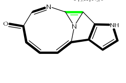	15.5678	192.9938	-7.8869	86.8553	311.4334	InChI=1S/C12H7N3O/c16-7-2-1-3-9-8-4-5-13-10(8)11-12(14-6-7)15(9)11/h1-6,13H/b2-1-,9-3-,14-6-
C-3	$C_{12}H_7N_3O$ 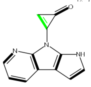	10.7671	308.7869	-3.1372	-4.7643	311.4427	InChI=1S/C12H7N3O/c16-10-6-9(10)15-11-7(2-1-4-13-11)8-3-5-14-12(8)15/h1-6,14H
H-99	$C_{12}H_7N_3O$ 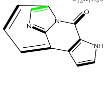	5.557	100.0915	-2.0407	182.0111	311.4489	InChI=1S/C12H7N3O/c16-12-10-8(4-5-13-10)9-3-1-2-7-6-14-11(9)15(7)12/h1-6,13H
n-64	$C_{12}H_7N_3O$ 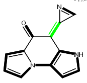	2.4733	304.3156	-2.2843	0.34	311.4764	InChI=1S/C12H7N3O/c16-12-9-2-1-5-15(9)8-3-4-13-11(8)10(12)7-6-14-7/h1-6,13H/b10-7-
n-158	$C_{12}H_7N_3O$ 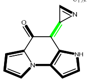	2.4747	304.314	-2.2851	0.34	311.4764	InChI=1S/C12H7N3O/c16-12-9-2-1-5-15(9)8-3-4-13-11(8)10(12)7-6-14-7/h1-6,13H/b10-7+
C-54	$C_{12}H_7N_3O$ 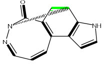	9.1065	44.1580	-3.0624	224.2046	311.6774	InChI=1S/C12H7N3O/c16-12-9-6-10-11-8(3-5-13-11)7(9)2-1-4-14-15(10)12/h1-6,13H/b2-1-,14-4-
H-129	$C_{12}H_7N_3O$ 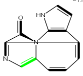	5.3118	98.435	-2.2713	184.8172	311.7376	InChI=1S/C12H7N3O/c16-12-10-8-3-1-2-7(6-14-10)15(12)11-9(8)4-5-13-11/h1-6,13H/b2-1-,3-1-,7-2-,8-3-
H1-68	$C_{12}H_7N_3O$ 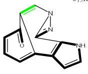	27.2378	156.5714	-6.4043	101.8232	311.7959	InChI=1S/C12H7N3O/c16-10-3-1-2-7-8-4-5-13-11(8)12-14-15(12)6-9(7)10/h1-6,13H
r1-17	$C_{12}H_7N_3O$ 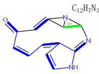	29.3397	248.9029	-15.3466	29.2529	311.8398	InChI=1S/C12H7N3O/c16-8-3-1-2-7-4-5-13-11(7)14-12-10-9(6-8)15(10)12/h1-6H,(H,13,14)/b3-1-,7-2+,9-6-
o-193	$C_{12}H_7N_3O$ 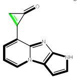	10.416	306.8439	-3.1975	-3.8472	311.8672	InChI=1S/C12H7N3O/c16-10-6-8(10)7-2-1-5-15-9-3-4-13-11(9)14-12(7)15/h1-6,13H
B-219	$C_{12}H_7N_3O$ 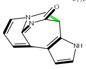	4.9835	33.8328	-2.1425	239.7607	311.8971	InChI=1S/C12H7N3O/c16-12-9-6-15-5-1-2-8(11(15)14-12)7-3-4-13-10(7)9/h1-6,13H
k-43	$C_{12}H_7N_3O$ 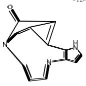	13.4211	178.1531	-7.9712	106.6194	311.9131	InChI=1S/C12H7N3O/c16-12-10-7-6-15(12)5-1-3-13-8-2-4-14-11(8)9(7)10/h1-6,14H/b5-1+,13-3+
F-1	$C_{12}H_7N_3O$ 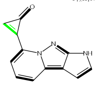	10.6378	306.5472	-3.1774	-4.8266	311.9631	InChI=1S/C12H7N3O/c16-11-6-8(11)10-3-1-2-9-7-4-5-13-12(7)14-15(9)10/h1-6H,(H,13,14)
H11-53	$C_{12}H_7N_3O$ 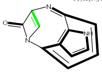	6.8963	74.9445	-3.8231	208.2216	312.0012	InChI=1S/C12H7N3O/c16-12-9-6-15(12)10-3-1-2-8(14-9)11-7(10)4-5-13-11/h1-6,13H/b14-8-
o-35	$C_{12}H_7N_3O$ 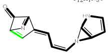	14.8701	148.4509	-12.0268	136.4392	312.2119	InChI=1S/C12H7N3O/c16-11-8-6-7(10(11)14-8)2-1-5-15-9-3-4-13-12(9)15/h1-6,13H/b5-1-,7-2+
o-104	$C_{12}H_7N_3O$ 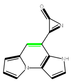	2.8789	309.1423	-2.3387	-5.3	312.489	InChI=1S/C12H7N3O/c16-12-11(14-12)8-6-7-2-1-5-15(7)9-3-4-13-10(8)9/h1-6,13H
o-102	$C_{12}H_7N_3O$ 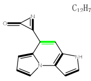	2.9152	309.1682	-2.3145	-6.23	312.5084	InChI=1S/C12H7N3O/c16-12-11(14-12)7-6-8-10(3-4-13-8)15-5-1-2-9(7)15/h1-6,13H
B-8	$C_{12}H_7N_3O$ 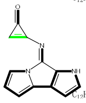 $C_{12}H_7N_3O$	42.9601	256.4993	-17.9518	13.2609	312.5399	InChI=1S/C12H7N3O/c16-10-6-8(10)14-12-11-7(3-4-13-11)9-2-1-5-15(9)12/h1-6,13H/b14-12-

B-49	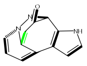 $C_{12}H_7N_3O$	4.6223	41.2763	-1.5848	234.1641	312.5505	InChI=1S/C12H7N3O/c16-10-6-9-7-2-1-5-15(9)14-12(10)11-8(7)3-4-13-11/h1-6,13H
I-59	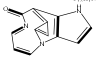 $C_{12}H_7N_3O$	19.3807	144.5724	-13.8023	127.6449	312.8513	InChI=1S/C12H7N3O/c16-12-10-7-6-9(7)15(5-1-3-14-12)8-2-4-13-11(8)10/h1-6,13H/b5-1-,14-3+
I-101	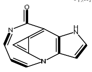 $C_{12}H_7N_3O$	19.359	144.5778	-13.7938	127.6361	312.8514	InChI=1S/C12H7N3O/c16-12-10-7-6-9(7)15(5-1-3-14-12)8-2-4-13-11(8)10/h1-6,13H/b5-1-,14-3-
H-65	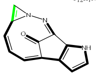 $C_{12}H_7N_3O$	2.5449	242.9961	-1.2733	49.2237	312.8624	InChI=1S/C12H7N3O/c16-12-9-3-1-2-7-6-15(7)14-11(12)10-8(9)4-5-13-10/h1-6,13H/b2-1-,9-3-,14-11-
o-34	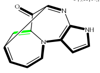 $C_{12}H_7N_3O$	5.9523	34.7442	-2.3034	234.0187	312.9849	InChI=1S/C12H7N3O/c16-11-9-6-7-2-1-5-15(9)8-3-4-13-12(8)14-10(7)11/h1-6,13H
H11-18	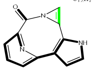 $C_{12}H_7N_3O$	23.2814	232.1553	-5.7974	42.6815	313.0631	InChI=1S/C12H7N3O/c16-12-9-3-1-2-8(14-9)7-4-5-13-11(7)10-6-15(10)12/h1-6,13H
B-233	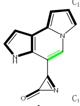 $C_{12}H_7N_3O$	2.9313	309.8885	-2.3299	-5.1700	313.1706	InChI=1S/C12H7N3O/c16-12-11(14-12)8-6-15-5-1-2-9(15)7-3-4-13-10(7)8/h1-6,13H
I1-40	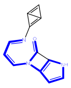 $C_{12}H_7N_3O$	19.1833	274.6317	-21.4888	40.8831	313.2456	InChI=1S/C12H7N3O/c16-12-11-9(2-4-14-11)15(12)5-1-3-13-10-7-6-8(7)10/h1-6,14H/b5-1-,13-3+
p1-60	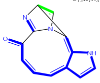 $C_{12}H_7N_3O$	6.9333	46.1842	-5.8893	238.397	313.2881	InChI=1S/C12H7N3O/c16-9-3-1-2-7-4-5-13-10(7)11-8-6-15(11)12(9)14-8/h1-6,13H/b3-1-,7-2-,11-10+
A-8	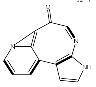 $C_{12}H_7N_3O$	21.772	237.3524	-9.6321	39.4809	313.3255	InChI=1S/C12H7N3O/c16-9-6-14-12-8(3-4-13-12)7-2-1-5-15-10(7)11(9)15/h1-6,13H/b14-6-
o-218	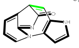 $C_{12}H_7N_3O$	8.1538	70.3117	-2.0678	195.134	313.509	InChI=1S/C12H7N3O/c16-12-8-6-9-10(3-4-13-9)15-5-1-2-7(8)11(15)14-12/h1-6,13H
o-17	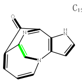 $C_{12}H_7N_3O$	7.2768	35.9741	-1.6428	234.7928	313.5715	InChI=1S/C12H7N3O/c16-12-7-2-1-5-15-6-8(7)14-11(12)10-9(15)3-4-13-10/h1-6,13H
H11-30	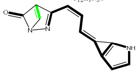 $C_{12}H_7N_3O$	19.3321	227.2583	-12.0315	71.803	313.6043	InChI=1S/C12H7N3O/c16-12-9-6-15(12)14-10(9)3-1-2-7-8-4-5-13-11(7)8/h1-6,13H/b3-1-,7-2-
s12-2	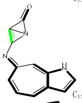 $C_{12}H_7N_3O$	25.8203	285.5071	-16.9956	10.0278	313.75	InChI=1S/C12H7N3O/c16-12-10-11(15(10)12)14-8-3-1-2-7-4-5-13-9(7)6-8/h1-6,13H/b14-8-
o-205	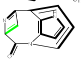 $C_{12}H_7N_3O$	6.9058	117.4832	-2.7311	166.9211	313.8136	InChI=1S/C12H7N3O/c16-12-9-6-8(14-9)7-2-1-5-15(12)10-3-4-13-11(7)10/h1-6,13H
D-23	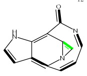 $C_{12}H_7N_3O$	17.0747	173.8824	-10.2052	102.8610	313.8785	InChI=1S/C12H7N3O/c16-12-10-9-6-15(9)8(2-1-4-14-12)7-3-5-13-11(7)10/h1-6,13H/b2-1-,14-4-
q11-44	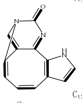 $C_{12}H_7N_3O$	9.1851	119.441	-5.7176	159.3183	313.9117	InChI=1S/C12H7N3O/c16-12-14-11-8-6-15(12)9(8)3-1-2-7-4-5-13-10(7)11/h1-6,13H/b2-1-,9-3+
r-6	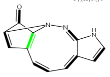 $C_{12}H_7N_3O$	6.4136	112.7865	-4.2017	172.3164	314.1385	InChI=1S/C12H7N3O/c16-11-9-6-8-3-1-2-7-4-5-13-12(7)14-15(9)10(8)11/h1-6H,(H,13,14)/b3-1-,7-2-
C-47	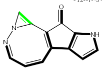 $C_{12}H_7N_3O$	4.0441	246.7152	-2.1891	50.2445	314.3742	InChI=1S/C12H7N3O/c16-12-10-7(8-3-5-13-11(8)12)2-1-4-14-15-6-9(10)15/h1-6,13H/b2-1-,14-4-

n-109	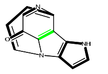 $C_{12}H_7N_3O$	6. 8879	94. 5107	-1. 7716	177. 9344	314. 4407	InChI=1S/C12H7N3O/c16-9-6-14-7-2-1-5-15-8-3-4-13-11(8)10(7)12(9)15/h1-6,13H
X22-17	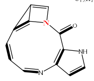 $C_{12}H_7N_3O$	12. 9427	258. 9273	-8. 3136	36. 5793	314. 558	InChI=1S/C12H7N3O/c16-12-10-9(3-5-14-10)13-4-1-2-7-8-6-15(12)11(7)8/h1-6,14H/b2-1-,13-4-
H11-9	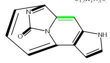 $C_{12}H_7N_3O$	7. 2846	120. 2866	-1. 2434	156. 5472	314. 8573	InChI=1S/C12H7N3O/c16-12-14-8-2-1-3-10-7-4-5-13-9(7)6-11(8)15(10)12/h1-6,13H
H-125	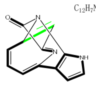 $C_{12}H_7N_3O$	7. 8197	48. 1039	-2. 5023	226. 1072	315. 0028	InChI=1S/C12H7N3O/c16-12-10-6-7-2-1-3-9(14-10)8-4-5-13-11(8)15(7)12/h1-6,13H/b2-1-,3-1-,7-2+,9-3-
H1-131	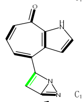 $C_{12}H_7N_3O$	24. 395	143. 2772	-10. 1126	141. 074	315. 0464	InChI=1S/C12H7N3O/c16-10-3-1-2-7(8-4-5-13-12(8)10)9-6-11-14-15(9)11/h1-6,13H
k-46	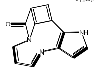 $C_{12}H_7N_3O$	27. 787	240. 7844	-1. 1068	46. 1786	315. 1659	InChI=1S/C12H7N3O/c16-12-8-6-7-10-9(2-4-14-10)13-3-1-5-15(12)11(7)8/h1-6,14H/b5-1-,13-3+
C-73	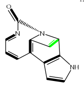 $C_{12}H_7N_3O$	9. 7606	178. 4313	-4. 4364	99. 6347	315. 2187	InChI=1S/C12H7N3O/c16-12-14-4-1-2-7-8-3-5-13-11(8)10-6-9(7)15(10)12/h1-6,13H/b2-1-,14-4+
s12-43	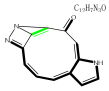 $C_{12}H_7N_3O$	10. 4399	245. 8797	-7. 155	43. 6944	315. 58	InChI=1S/C12H7N3O/c16-10-6-9-7(4-5-13-9)2-1-3-8-11-12(10)15(11)14-8/h1-6,13H/b3-1-,7-2-,9-6+
X-9	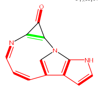 $C_{12}H_7N_3O$	26. 7636	219. 1431	-12. 4290	62. 8759	315. 6816	InChI=1S/C12H7N3O/c16-11-9-10(11)15-6-7(2-1-4-13-9)8-3-5-14-12(8)15/h1-6,14H/b2-1-,13-4-
H1-82	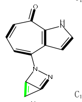 $C_{12}H_7N_3O$	15. 7637	214. 8021	-17. 2171	79. 2096	315. 7159	InChI=1S/C12H7N3O/c16-11-3-1-2-9(7-4-5-13-12(7)11)15-10-6-8(10)14-15/h1-6,13H
j-21	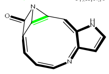 $C_{12}H_7N_3O$	11. 6271	215. 2692	-9. 5678	82. 5521	315. 8051	InChI=1S/C12H7N3O/c16-12-7-2-1-4-13-8-3-5-14-9(8)6-10-11(7)15(10)12/h1-6,14H/b2-1-,4-1-,7-2+,9-6+,10-6+,13-4-,13-8-
s11-16	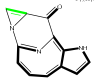 $C_{12}H_7N_3O$	15. 5125	173. 749	-10. 9072	110. 6101	315. 847	InChI=1S/C12H7N3O/c16-12-8-6-15(8)9-3-1-2-7-4-5-13-10(7)11(12)14-9/h1-6,13H/b2-1-,3-1-,7-2-,9-3+,11-10+
A2-34	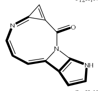 $C_{12}H_7N_3O$	24. 5557	237. 0201	-13. 3672	59. 2125	316. 1424	InChI=1S/C12H7N3O/c16-12-8-6-9(8)13-4-1-2-10-7-3-5-14-11(7)15(10)12/h1-6,14H/b4-1-,10-2-,13-9-
H-93	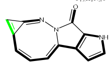 $C_{12}H_7N_3O$	14. 6426	209. 0033	-9. 6537	70. 3497	316. 2562	InChI=1S/C12H7N3O/c16-12-11-8(4-5-13-11)10-3-1-2-7-6-9(7)14-15(10)12/h1-6,13H/b2-1-,10-3-,14-9-
F-28	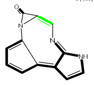 $C_{12}H_7N_3O$	12. 9435	269. 8555	-4. 3151	32. 165	316. 3337	InChI=1S/C12H7N3O/c16-12-10-6-14-11-8(4-5-13-11)7-2-1-3-9(7)15(10)12/h1-6H,(H,13,14)/b8-7-,10-6-
H-83	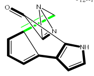 $C_{12}H_7N_3O$	16. 3631	177. 7765	-11. 4741	103. 5919	316. 3829	InChI=1S/C12H7N3O/c16-11-10-9(4-5-13-10)8-3-1-2-7(8)6-15-12(11)14-15/h1-6,13H/b7-6+
x4-31	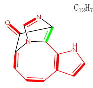 $C_{12}H_7N_3O$	9. 4491	98. 3008	-5. 0371	174. 7189	316. 59	InChI=1S/C12H7N3O/c16-12-8-3-1-2-7-4-5-13-9(7)11-10(12)14-6-15(8)11/h1-6,13H/b2-1-,8-3-
x6-30	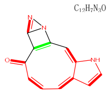 $C_{12}H_7N_3O$	24. 8518	158. 4784	-9. 4787	115. 8438	316. 7002	InChI=1S/C12H7N3O/c16-10-3-1-2-7-4-5-13-8(7)6-9-11(10)12-14-15(9)12/h1-6,13H/b3-1-,7-2-,8-6+
H11-114	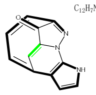 $C_{12}H_7N_3O$	9. 7836	115. 6472	-2. 2443	162. 4648	317. 2073	InChI=1S/C12H7N3O/c16-11-9-3-1-2-7-6-10(11)15(14-9)12-8(7)4-5-13-12/h1-6,13H/b3-1-,7-2+

H1-111	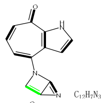 $C_{12}H_7N_3O$	21.8696	141.2111	-11.7677	147.5788	317.2772	InChI=1S/C12H7N3O/c16-10-3-1-2-9(7-4-5-13-11(7)10)15-6-8-12(15)14-8/h1-6,13H
I-67	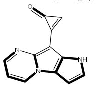 $C_{12}H_7N_3O$	10.6191	309.7027	-3.3023	-1.9184	317.3861	InChI=1S/C12H7N3O/c16-9-6-7(9)10-11-8(2-4-13-11)15-5-1-3-14-12(10)15/h1-6,13H
x7-7	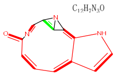 $C_{12}H_7N_3O$	29.981	247.4215	-15.76	41.6674	317.4129	InChI=1S/C12H7N3O/c16-9-3-1-2-7-4-5-13-10(7)12-11-8(6-14-9)15(11)12/h1-6,13H/b3-1-,7-2-,12-10-,14-6+
H1-98	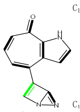 $C_{12}H_7N_3O$	24.69	144.6163	-10.2797	142.0617	317.4648	InChI=1S/C12H7N3O/c16-10-3-1-2-7(8-4-5-13-11(8)10)9-6-15-12(9)14-15/h1-6,13H
C-60	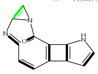 $C_{12}H_7N_3O$	27.3000	235.8692	-12.5884	40.8583	317.7645	InChI=1S/C12H7N3O/c16-12-10-7(8-3-5-14-11(8)10)2-1-4-13-9-6-15(9)12/h1-6,14H/b2-1-,13-4+
p1-56	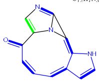 $C_{12}H_7N_3O$	10.5399	138.1545	-7.0148	149.9369	317.8991	InChI=1S/C12H7N3O/c16-9-3-1-2-7-4-5-13-10(7)11-12-14-6-8(9)15(11)12/h1-6,13H/b3-1-,7-2-,11-10+
j-15	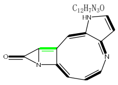 $C_{12}H_7N_3O$	17.3743	224.8466	-9.7016	67.7158	317.9955	InChI=1S/C12H7N3O/c16-12-11-7-6-9-8(3-5-14-9)13-4-1-2-10(7)15(11)12/h1-6,14H/b4-1-,9-6+,10-2+,13-8-
p11-16	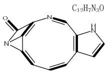 $C_{12}H_7N_3O$	15.5012	174.0018	-10.518	121.137	318.0092	InChI=1S/C12H7N3O/c16-12-10-11-9(15(11)12)3-1-2-7-4-5-13-8(7)6-14-10/h1-6,13H/b2-1-,3-1-,7-2-,8-6+,9-3+,14-6-,14-10+
q-47	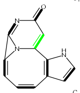 $C_{12}H_7N_3O$	10.4253	143.2734	-6.3676	139.8607	318.13	InChI=1S/C12H7N3O/c16-10-6-9-11-7(4-5-13-11)2-1-3-8-12(14-10)15(8)9/h1-6,13H/b2-1-,8-3-
o-57	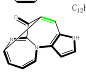 $C_{12}H_7N_3O$	5.0965	33.3123	-1.7411	245.7002	318.2781	InChI=1S/C12H7N3O/c16-11-7-6-9-10(3-4-13-9)15-5-1-2-8(7)14-12(11)15/h1-6,13H
o-30	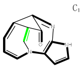 $C_{12}H_7N_3O$	6.403	72.8361	-2.0772	204.76	318.3322	InChI=1S/C12H7N3O/c16-11-8-6-15-5-1-2-7(8)10(11)14-12-9(15)3-4-13-12/h1-6,13H/b14-10-
G1-35	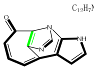 $C_{12}H_7N_3O$	15.2986	95.351	-4.1313	175.2603	318.5861	InChI=1S/C12H7N3O/c16-9-3-1-2-7-8-4-5-13-12(8)15-6-14-10(7)11(9)15/h1-6,13H
j-13	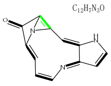 $C_{12}H_7N_3O$	16.9481	229.8972	-11.6096	61.8524	318.7452	InChI=1S/C12H7N3O/c16-12-9-2-1-4-13-7-3-5-14-8(7)6-10-11(12)15(9)10/h1-6,14H/b4-1-,8-6-,9-2+,13-7+
A1-39	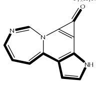 $C_{12}H_7N_3O$	24.7572	244.1837	-8.8446	47.1399	318.8529	InChI=1S/C12H7N3O/c16-12-9-10-7(3-5-14-10)8-2-1-4-13-6-15(8)11(9)12/h1-6,14H
H-44	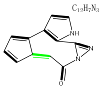 $C_{12}H_7N_3O$	20.8228	204.1442	-6.3144	79.5227	319.1696	InChI=1S/C12H7N3O/c16-10-6-7-2-1-3-8(7)9-4-5-13-11(9)12-14-15(10)12/h1-6,13H/b7-6+
H-12	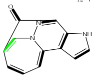 $C_{12}H_7N_3O$	5.7848	34.6729	-1.4201	238.8613	319.1793	InChI=1S/C12H7N3O/c16-12-7-2-1-3-9-8-4-5-13-10(8)11(12)14-15(9)6-7/h1-6,13H
B-111	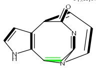 $C_{12}H_7N_3O$	9.4622	61.1771	-4.2654	212.0121	319.2151	InChI=1S/C12H7N3O/c16-12-8-2-1-5-15-9(6-10(15)14-12)11-7(8)3-4-13-11/h1-6,13H/b5-1-,8-2-,14-10+
j-14	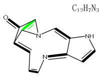 $C_{12}H_7N_3O$	22.411	199.5597	-13.7771	90.4627	319.3004	InChI=1S/C12H7N3O/c16-12-7-2-1-4-13-8-3-5-14-9(8)6-15-10(7)11(12)15/h1-6,14H/b4-1-,7-2-,9-6+,13-8+
I-87	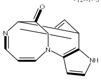 $C_{12}H_7N_3O$	6.7931	38.4503	-2.4454	234.2248	319.3318	InChI=1S/C12H7N3O/c16-12-9-6-7-10-8(2-4-14-10)15(9)5-1-3-13-11(7)12/h1-6,14H/b5-1-,13-3-

o-98	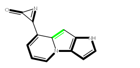	$C_{12}H_7N_3O$	3.1688	315.5445	-2.2232	-6.23	319.4161	InChI=1S/C12H7N3O/c16-12-11(14-12)7-2-1-5-15-9-3-4-13-8(9)6-10(7)15/h1-6,13H
r1-32	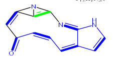	$C_{12}H_7N_3O$	18.4483	242.8025	-13.6118	47.2087	319.5661	InChI=1S/C12H7N3O/c16-8-3-1-2-7-4-5-13-11(7)14-12-10-9(6-8)15(10)12/h1-6H,(H,13,14)/b3-1-,7-2-,9-6+
E-46	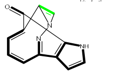	$C_{12}H_7N_3O$	14.6171	146.549	-10.8119	135.8597	319.7265	InChI=1S/C12H7N3O/c16-12-8-2-1-3-9(14-15-6-10(8)15)7-4-5-13-11(7)12/h1-6,13H/b3-1-,8-2+,14-9-
X-48	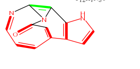	$C_{12}H_7N_3O$	20.4304	225.4575	-8.0750	55.8505	319.7574	InChI=1S/C12H7N3O/c16-9-6-7-2-1-4-14-12-11(15(9)12)10-8(7)3-5-13-10/h1-6,13H/b2-1-,14-4-
A-41	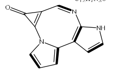	$C_{12}H_7N_3O$	27.4174	235.6397	-10.6258	55.6913	319.7926	InChI=1S/C12H7N3O/c16-11-8-6-14-12-7(3-4-13-12)9-2-1-5-15(9)10(8)11/h1-6,13H/b14-6-
H11-92	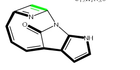	$C_{12}H_7N_3O$	6.8026	143.8225	-2.8103	151.6169	319.9262	InChI=1S/C12H7N3O/c16-12-9-3-1-2-7-6-10(14-7)15(12)11-8(9)4-5-13-11/h1-6,13H/b2-1-,9-3-
H11-15	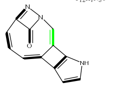	$C_{12}H_7N_3O$	11.5506	263.256	-0.8694	21.2869	320.1346	InChI=1S/C12H7N3O/c16-12-10-3-1-2-7-8-4-5-13-11(8)9(7)6-15(12)14-10/h1-6,13H/b3-1-,7-2-,9-6+
X-30	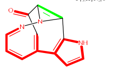	$C_{12}H_7N_3O$	5.7308	191.2725	-3.7435	100.8806	320.1388	InChI=1S/C12H7N3O/c16-9-6-7-2-1-4-14-15-11(9)12(15)10-8(7)3-5-13-10/h1-6,13H/b2-1-,14-4-
A-9	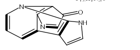	$C_{12}H_7N_3O$	10.1036	123.629	-2.5861	159.0908	320.1731	InChI=1S/C12H7N3O/c16-9-6-14-10-7-2-1-5-15(11(9)10)12-8(7)3-4-13-12/h1-6,13H
r-11	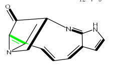	$C_{12}H_7N_3O$	9.7513	260.2814	-4.9889	29.6158	320.2472	InChI=1S/C12H7N3O/c16-11-8-6-15-9(10(11)15)3-1-2-7-4-5-13-12(7)14-8/h1-6H,(H,13,14)/b3-1+,7-2-
H-123	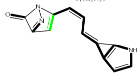	$C_{12}H_7N_3O$	19.257	228.1598	-12.0637	81.2697	321.2139	InChI=1S/C12H7N3O/c16-12-10-6-7(15(12)14-10)2-1-3-8-9-4-5-13-11(8)9/h1-6,13H/b2-1-,8-3-
H1-132	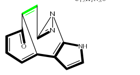	$C_{12}H_7N_3O$	24.8723	153.6644	-7.3158	127.5475	321.2518	InChI=1S/C12H7N3O/c16-10-3-1-2-7-8-4-5-13-12(8)15-11(14-15)6-9(7)10/h1-6,13H
F-60	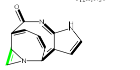	$C_{12}H_7N_3O$	19.4982	145.9334	-12.2227	125.9493	321.5251	InChI=1S/C12H7N3O/c16-12-8-2-1-3-9(15-6-10(8)15)7-4-5-13-11(7)14-12/h1-6H,(H,13,14,16)
x6-29	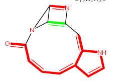	$C_{12}H_7N_3O$	23.9086	211.226	-14.5201	91.6976	321.615	InChI=1S/C12H7N3O/c16-10-3-1-2-7-4-5-13-8(7)6-9-11-12(14-9)15(10)11/h1-6,13H/b3-1-,7-2-,8-6+
E-28	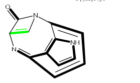	$C_{12}H_7N_3O$	7.3988	96.5711	-3.8492	188.6252	322.108	InChI=1S/C12H7N3O/c16-12-9-6-15(12)10-3-1-2-8(14-9)7-4-5-13-11(7)10/h1-6,13H/b14-8-
r-41	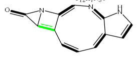	$C_{12}H_7N_3O$	16.1938	221.0346	-9.957	88.9855	322.146	InChI=1S/C12H7N3O/c16-12-10-8-3-1-2-7-4-5-13-11(7)14-6-9(8)15(10)12/h1-6H,(H,13,14)/b3-1-,7-2-,9-6+
o-94	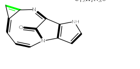	$C_{12}H_7N_3O$	21.8996	201.629	-12.7759	88.5456	322.1473	InChI=1S/C12H7N3O/c16-12-11-10-9(3-4-13-10)15(12)5-1-2-7-6-8(7)14-11/h1-6,13H/b5-1-,7-2-,14-11-
H-27	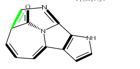	$C_{12}H_7N_3O$	5.9223	122.0041	-2.1837	164.9335	322.1898	InChI=1S/C12H7N3O/c16-12-7-2-1-3-9-8-4-5-13-10(8)11(14-6-7)15(9)12/h1-6,13H
H1-117	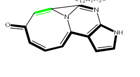	$C_{12}H_7N_3O$	3.4496	314.6995	-2.1369	-4.97	322.2114	InChI=1S/C12H7N3O/c16-7-2-1-3-9-8-4-5-13-11(8)14-12-10(6-7)15(9)12/h1-6,13H/b2-1-,9-3-,10-6-

x8-42	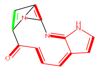 $C_{12}H_7N_3O$	11.6931	232.5301	-8.1839	59.9533	322.2381	InChI=1S/C12H7N3O/c16-10-3-1-2-7-4-5-13-11(7)14-12-9-6-8(10)15(9)12/h1-6H,(H,13,14)/b3-1+,7-2-
o-60	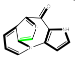 $C_{12}H_7N_3O$	10.2032	83.4459	-1.4717	186.9894	322.3706	InChI=1S/C12H7N3O/c16-12-10-7-2-1-5-15(9(7)6-14-10)8-3-4-13-11(8)12/h1-6,13H
H1-4	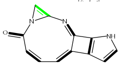 $C_{12}H_7N_3O$	23.0032	229.2796	-10.8701	60.4065	322.6853	InChI=1S/C12H7N3O/c16-10-3-1-2-7-8-4-5-13-11(8)12(7)14-9-6-15(9)10/h1-6,13H/b3-1-,7-2-,14-12+
q-23	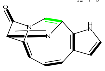 $C_{12}H_7N_3O$	9.0954	146.3492	-3.7305	128.4647	322.8845	InChI=1S/C12H7N3O/c16-12-11-9-3-1-2-7-4-5-13-10(7)8(14-11)6-15(9)12/h1-6,13H/b2-1-,9-3-
G-21	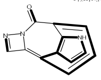 $C_{12}H_7N_3O$	7.2744	110.2844	-5.6755	184.311	322.8981	InChI=1S/C12H7N3O/c16-12-9-3-1-2-7(10-6-14-15(10)12)8-4-5-13-11(8)9/h1-6,13H
X-49	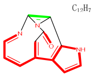 $C_{12}H_7N_3O$	18.3921	206.7738	-7.2790	74.8771	322.9435	InChI=1S/C12H7N3O/c16-9-6-7-2-1-4-14-12-11(15(9)12)10-8(7)3-5-13-10/h1-6,13H/b2-1-,14-4+
X22-51	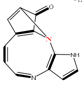 $C_{12}H_7N_3O$	5.2454	103.8589	-4.5579	198.9348	323.0094	InChI=1S/C12H7N3O/c16-11-9-6-7-2-1-4-13-8-3-5-14-12(8)15(9)10(7)11/h1-6,14H/b2-1-,13-4-
j-2	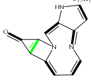 $C_{12}H_7N_3O$	43.849	183.4522	-19.6747	105.5004	323.0448	InChI=1S/C12H7N3O/c16-12-10-9-2-1-4-13-7-3-5-14-8(7)6-15(9)11(10)12/h1-6,14H/b4-1-,8-6+,9-2-,13-7+
G-7	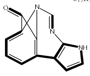 $C_{12}H_7N_3O$	7.1878	111.0751	-2.2408	179.4143	323.1684	InChI=1S/C12H7N3O/c16-11-9-3-1-2-7-8-4-5-13-12(8)14-6-15(9)10(7)11/h1-6,13H
j-16	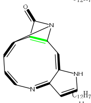 $C_{12}H_7N_3O$	34.9177	176.2139	-11.6661	102.2709	323.1887	InChI=1S/C12H7N3O/c16-12-11-9-2-1-4-13-7-3-5-14-8(7)6-10(11)15(9)12/h1-6,14H/b2-1-,4-1-,8-6+,9-2+,10-6+,13-4-,13-7-
H1-70	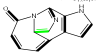 $C_{12}H_7N_3O$	5.8149	109.6856	-1.8814	179.8445	323.3168	InChI=1S/C12H7N3O/c16-10-3-1-2-7-8-4-5-13-11(8)12-14-6-9(7)15(10)12/h1-6,13H
x8-33	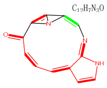 $C_{12}H_7N_3O$	30.6646	246.8901	-15.9943	44.9422	323.5791	InChI=1S/C12H7N3O/c16-9-3-1-2-7-4-5-13-12(7)14-6-8-10-11(9)15(8)10/h1-6H,(H,13,14)/b3-1-,7-2-,8-6-
r1-21	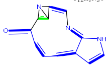 $C_{12}H_7N_3O$	17.042	248.406	-12.8016	49.6107	323.7516	InChI=1S/C12H7N3O/c16-9-3-1-2-7-4-5-13-12(7)14-6-8-10-11(9)15(8)10/h1-6H,(H,13,14)/b3-1-,7-2-,8-6+
H-115	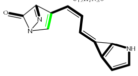 $C_{12}H_7N_3O$	19.4701	229.9522	-12.1241	80.9088	323.8598	InChI=1S/C12H7N3O/c16-12-10-7(6-15(12)14-10)2-1-3-8-9-4-5-13-11(8)9/h1-6,13H/b2-1-,8-3-
G-5	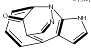 $C_{12}H_7N_3O$	6.7184	33.5261	-1.9776	238.8266	323.9794	InChI=1S/C12H7N3O/c16-11-9-6-14-15-10(11)3-1-2-7(9)8-4-5-13-12(8)15/h1-6,13H
x3-19	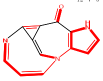 $C_{12}H_7N_3O$	17.7343	221.0329	-9.3046	51.7688	324.1335	InChI=1S/C12H7N3O/c16-12-9-7-6-15(5-1-3-13-10(7)9)8-2-4-14-11(8)12/h1-6,14H/b5-1-,13-3-
q1-13	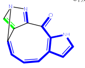 $C_{12}H_7N_3O$	13.6833	101.3802	-7.5023	186.2112	324.2038	InChI=1S/C12H7N3O/c16-12-10-7(4-5-13-10)2-1-3-9-8-6-15(9)14-11(8)12/h1-6,13H/b2-1-,9-3+
B-261	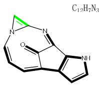 $C_{12}H_7N_3O$	11.5541	185.1853	-8.6438	103.4119	324.2391	InChI=1S/C12H7N3O/c16-12-8-2-1-5-15-6-9(15)14-11(12)10-7(8)3-4-13-10/h1-6,13H/b5-1-,8-2-,14-11-
C-52	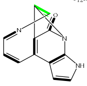 $C_{12}H_7N_3O$	11.9271	120.1919	-5.3248	175.5246	324.2574	InChI=1S/C12H7N3O/c16-12-10-7-2-1-4-13-6-9(10)15(12)11-8(7)3-5-14-11/h1-6,14H/b2-1-,4-1-,7-2-,9-6-,13-4?,13-6?

A-151	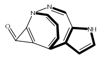	$C_{12}H_7N_3O$	28.1914	208.0815	-10.9522	72.0548	324.5239	InChI=1S/C12H7N3O/c16-12-10-8-2-1-5-15(11(10)12)14-6-9-7(8)3-4-13-9/h1-6,13H/b14-6-
I1-45	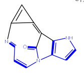	$C_{12}H_7N_3O$	16.5552	228.1068	-6.7528	59.958	324.9105	InChI=1S/C12H7N3O/c16-12-10-7-6-8(7)13-3-1-5-15(12)9-2-4-14-11(9)10/h1-6,14H/b5-1-,10-7-,13-3+
H-13	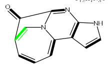	$C_{12}H_7N_3O$	7.5134	121.769	-1.6206	163.9985	324.9178	InChI=1S/C12H7N3O/c16-10-7-2-1-3-9-8-4-5-13-11(8)14-12(10)15(9)6-7/h1-6,13H
o-227	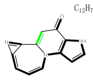	$C_{12}H_7N_3O$	24.5225	166.3196	-9.1128	115.0129	324.9256	InChI=1S/C12H7N3O/c16-10-6-9-11-7(14-11)2-1-5-15(9)8-3-4-13-12(8)10/h1-6,13H
x4-14	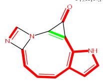	$C_{12}H_7N_3O$	28.8571	239.6461	-16.4459	55.8546	325.044	InChI=1S/C12H7N3O/c16-12-9-10-7(4-5-13-10)2-1-3-8-14-6-15(8)11(9)12/h1-6,13H/b2-1-,8-3-
A2-39	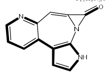	$C_{12}H_7N_3O$	20.6112	203.2816	-7.6498	90.225	325.1266	InChI=1S/C12H7N3O/c16-12-10-6-9-7(2-1-4-13-9)8-3-5-14-11(8)15(10)12/h1-6,14H
F-46	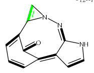	$C_{12}H_7N_3O$	12.1969	171.3321	-8.4357	118.9971	325.1852	InChI=1S/C12H7N3O/c16-11-7-2-1-3-9(11)10-6-15(10)14-12-8(7)4-5-13-12/h1-6H,(H,13,14)/b8-7-
j-12	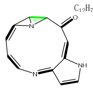	$C_{12}H_7N_3O$	16.1788	251.3693	-12.7943	48.9753	325.3577	InChI=1S/C12H7N3O/c16-10-6-8-7(3-5-14-8)13-4-1-2-9-11-12(10)15(9)11/h1-6,14H/b4-1-,8-6+,9-2-,13-7-
A-171	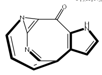	$C_{12}H_7N_3O$	8.1382	184.821	-5.6108	105.8705	325.3578	InChI=1S/C12H7N3O/c16-11-9-8(3-4-13-9)7-2-1-5-15-10(11)12(15)14-6-7/h1-6,13H/b5-1-,7-2+,14-6-
r-21	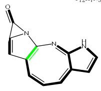	$C_{12}H_7N_3O$	14.6329	215.965	-6.6379	95.3195	325.4156	InChI=1S/C12H7N3O/c16-12-9-6-8-3-1-2-7-4-5-13-10(7)14-11(8)15(9)12/h1-6H,(H,13,14)
H11-134	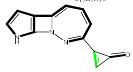	$C_{12}H_7N_3O$	42.2807	270.6401	-17.5813	16.0664	325.4455	InChI=1S/C12H7N3O/c16-11-6-8(11)9-2-1-3-10-7-4-5-13-12(7)15(10)14-9/h1-6,13H
H11-125	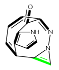	$C_{12}H_7N_3O$	16.4648	144.5879	-11.662	131.6228	325.4553	InChI=1S/C12H7N3O/c16-12-9-3-1-2-7(10-6-15(10)14-9)8-4-5-13-11(8)12/h1-6,13H/b3-1-,7-2+,14-9-
X23-14	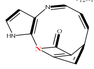	$C_{12}H_7N_3O$	35.1847	178.0543	-11.384	118.2383	325.4594	InChI=1S/C12H7N3O/c16-12-10-7-2-1-4-13-8-3-5-14-11(8)15(12)9(10)6-7/h1-6,14H/b2-1-,13-4-
o-207	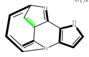	$C_{12}H_7N_3O$	8.1708	111.2585	-1.3792	170.7013	325.5857	InChI=1S/C12H7N3O/c16-12-8-6-7-2-1-5-15(12)9-3-4-13-11(9)10(8)14-7/h1-6,13H/b5-1-,7-2+
o-15	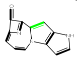	$C_{12}H_7N_3O$	11.3896	146.0053	-3.1998	138.6949	325.6652	InChI=1S/C12H7N3O/c16-12-7-2-1-5-15-9-3-4-13-8(9)6-10(15)11(12)14-7/h1-6,13H/b5-1-,7-2-
G-8	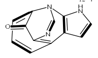	$C_{12}H_7N_3O$	6.1099	34.1798	-1.9431	242.3326	325.7278	InChI=1S/C12H7N3O/c16-11-9-3-1-2-7-8-4-5-13-12(8)15(9)6-14-10(7)11/h1-6,13H
o-61	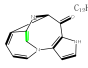	$C_{12}H_7N_3O$	5.3564	65.0881	-1.9593	219.5756	325.8005	InChI=1S/C12H7N3O/c16-12-10-7-2-1-5-15(6-8(7)14-10)9-3-4-13-11(9)12/h1-6,13H
H11-112	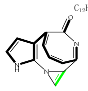	$C_{12}H_7N_3O$	19.3263	170.7741	-11.382	105.2929	325.8254	InChI=1S/C12H7N3O/c16-12-8-2-1-3-9(14-12)10-6-15(10)11-7(8)4-5-13-11/h1-6,13H
x2-2	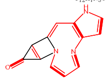	$C_{12}H_7N_3O$	44.6647	170.9174	-19.846	112.1233	325.8786	InChI=1S/C12H7N3O/c16-12-10-9-6-8-7(2-4-14-8)13-3-1-5-15(9)11(10)12/h1-6,14H/b5-1-,9-6-,13-3+

E-12	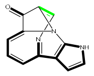 $C_{12}H_7N_3O$	9.2618	74.2055	-3.5044	206.8067	325.9341	InChI=1S/C12H7N3O/c16-11-9-3-1-2-8-7-4-5-13-12(7)15(9)10(11)6-14-8/h1-6,13H/b2-1-,9-3-
q-14	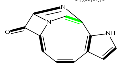 $C_{12}H_7N_3O$	8.1924	141.9647	-2.7498	150.3899	325.9495	InChI=1S/C12H7N3O/c16-11-9-3-1-2-7-4-5-13-10(7)8-6-15(9)12(11)14-8/h1-6,13H/b2-1-,9-3-
H-163	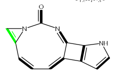 $C_{12}H_7N_3O$	23.503	237.7916	-10.716	50.8736	326.2605	InChI=1S/C12H7N3O/c16-12-14-11-8(9-4-5-13-10(9)11)3-1-2-7-6-15(7)12/h1-6,13H/b2-1-,8-3-,14-11+
X22-15	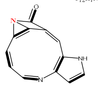 $C_{12}H_7N_3O$	11.2117	275.7946	-7.5539	31.8228	326.283	InChI=1S/C12H7N3O/c16-12-7-6-9-8(3-5-14-9)13-4-1-2-10-11(7)15(10)12/h1-6,14H/b2-1-,4-1-,7-6+,9-6+,10-2+,13-4-,13-8-
E-9	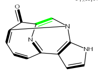 $C_{12}H_7N_3O$	5.6676	32.6603	-1.488	253.2698	326.3254	InChI=1S/C12H7N3O/c16-11-9-6-15-10(11)3-1-2-8(14-9)7-4-5-13-12(7)15/h1-6,13H/b2-1-,10-3-
x4-37	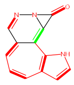 $C_{12}H_7N_3O$	13.419	231.0312	-5.0512	73.1905	326.4661	InChI=1S/C12H7N3O/c16-12-11-9-8(6-14-15(11)12)3-1-2-7-4-5-13-10(7)9/h1-6,13H
H11-34	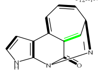 $C_{12}H_7N_3O$	6.402	104.3469	-2.3727	176.2397	326.5063	InChI=1S/C12H7N3O/c16-12-10-6-7-2-1-3-8(10)9-4-5-13-11(9)15(12)14-7/h1-6,13H
G1-85	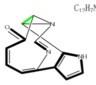 $C_{12}H_7N_3O$	20.3665	192.0836	-8.7673	69.7877	326.5209	InChI=1S/C12H7N3O/c16-9-3-1-2-8-7-4-5-13-10(7)12-11(9)15(12)6-14-8/h1-6,13H/b3-1-,8-2-
H-42	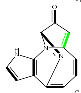 $C_{12}H_7N_3O$	8.1063	125.9041	-1.8084	164.0574	326.5657	InChI=1S/C12H7N3O/c16-10-6-7-2-1-3-9-8-4-5-13-11(8)15(7)12(10)14-9/h1-6,13H/b2-1-,9-3-
H11-26	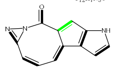 $C_{12}H_7N_3O$	30.7559	226.4123	-3.1898	60.7433	326.821	InChI=1S/C12H7N3O/c16-12-9-6-10-8(4-5-13-10)7(9)2-1-3-11-14-15(11)12/h1-6,13H/b3-1-,7-2-
n-172	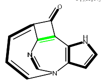 $C_{12}H_7N_3O$	9.5084	83.932	-3.139	205.0707	326.9305	InChI=1S/C12H7N3O/c16-12-7-2-1-5-15-6-14-10(7)9(12)11-8(15)3-4-13-11/h1-6,13H/b5-1-,7-2+
B-217	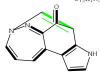 $C_{12}H_7N_3O$	5.9470	36.5117	-1.7935	246.7211	326.9660	InChI=1S/C12H7N3O/c16-12-9-6-15-5-1-2-7(11(12)14-15)8-3-4-13-10(8)9/h1-6,13H
H11-40	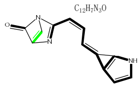 $C_{12}H_7N_3O$	19.0493	227.477	-11.769	89.5236	327.1579	InChI=1S/C12H7N3O/c16-12-9-6-15(12)10(14-9)3-1-2-7-8-4-5-13-11(7)8/h1-6,13H/b3-1-,7-2-
H1-45	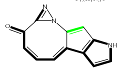 $C_{12}H_7N_3O$	12.4953	221.744	-6.8788	71.3658	327.1913	InChI=1S/C12H7N3O/c16-11-3-1-2-8-7-4-5-13-9(7)6-10(8)15-12(11)14-15/h1-6,13H/b3-1-,8-2-
G1-102	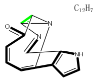 $C_{12}H_7N_3O$	17.2769	258.4896	-7.9662	25.5658	327.271	InChI=1S/C12H7N3O/c16-10-3-1-2-9-7-4-5-13-8(7)6-14-12-11(10)15(9)12/h1-6,13H
x5-46	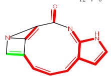 $C_{12}H_7N_3O$	21.2722	254.0267	-11.7759	37.9909	327.3277	InChI=1S/C12H7N3O/c16-12-10-9-8(6-15(9)10)3-1-2-7-4-5-13-11(7)14-12/h1-6H,(H,13,14,16)/b3-1-,7-2-
o-72	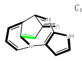 $C_{12}H_7N_3O$	9.6465	85.3861	-1.3028	190.7921	327.566	InChI=1S/C12H7N3O/c16-10-6-9-7-2-1-5-15(9)8-3-4-13-12(8)14-11(7)10/h1-6,13H
B-249	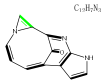 $C_{12}H_7N_3O$	12.1788	185.5793	-8.0506	105.6709	327.5795	InChI=1S/C12H7N3O/c16-11-7-2-1-5-15-6-9(15)10(11)14-12-8(7)3-4-13-12/h1-6,13H/b5-1-,7-2-
x4-12	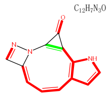 $C_{12}H_7N_3O$	28.843	239.7963	-16.5141	54.2626	327.6644	InChI=1S/C12H7N3O/c16-12-9-10-7(4-5-13-10)2-1-3-8-6-14-15(8)11(9)12/h1-6,13H/b2-1-,8-3-

C-70	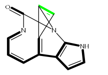 $C_{12}H_7N_3O$	15.4224	229.9853	-8.2708	69.6491	327.7104	InChI=1S/C12H7N3O/c16-12-14-4-1-2-7-8-3-5-13-11(8)15(12)10-6-9(7)10/h1-6,13H/b2-1-,14-4+
H1-74	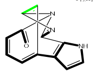 $C_{12}H_7N_3O$	10.2011	161.177	-4.0584	137.0456	327.9363	InChI=1S/C12H7N3O/c16-10-3-1-2-7-6-9-14-12(15(9)10)11-8(7)4-5-13-11/h1-6,13H/b3-1-,7-2+
j-6	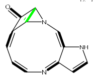 $C_{12}H_7N_3O$	19.2812	186.7255	-11.9631	105.5102	328.1127	InChI=1S/C12H7N3O/c16-12-7-2-1-4-13-8-3-5-14-9(8)6-15-10(7)11(12)15/h1-6,14H/b4-1-,7-2+,9-6+,13-8-
H11-160	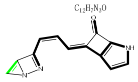 $C_{12}H_7N_3O$	14.8974	277.8875	-10.9147	37.22	328.2241	InChI=1S/C12H7N3O/c16-12-8(7-4-5-13-11(7)12)2-1-3-9-10-6-15(10)14-9/h1-6,13H/b3-1-,8-2-
x5-37	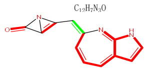 $C_{12}H_7N_3O$	27.1838	291.4628	-17.2036	15.7491	328.4594	InChI=1S/C12H7N3O/c16-12-10-9(15(10)12)6-8-3-1-2-7-4-5-13-11(7)14-8/h1-6H,(H,13,14)/b8-6+
H1-14	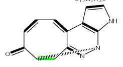 $C_{12}H_7N_3O$	5.1122	102.3543	-1.7656	192.1384	328.6005	InChI=1S/C12H7N3O/c16-11-3-1-2-7-8-4-5-13-12(8)15-10(11)6-9(7)14-15/h1-6,13H/b3-1-,7-2-
q1-24	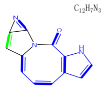 $C_{12}H_7N_3O$	15.515	162.4007	-5.1711	140.3574	328.7814	InChI=1S/C12H7N3O/c16-12-10-7(4-5-13-10)2-1-3-8-6-9-11(14-9)15(8)12/h1-6,13H/b2-1-,8-3-
r-43	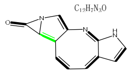 $C_{12}H_7N_3O$	14.0148	219.797	-6.4437	96.359	328.7999	InChI=1S/C12H7N3O/c16-12-10-8-3-1-2-7-4-5-13-11(7)14-9(8)6-15(10)12/h1-6H,(H,13,14)/b3-1-,7-2-
o-19	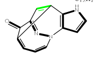 $C_{12}H_7N_3O$	5.3063	38.6774	-1.5446	251.8438	328.8518	InChI=1S/C12H7N3O/c16-12-7-2-1-5-15-10-3-4-13-11(10)8(7)6-9(12)14-15/h1-6,13H/b5-1-,7-2+
o-85	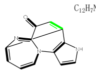 $C_{12}H_7N_3O$	6.0951	27.3608	-2.4223	256.3581	329.0251	InChI=1S/C12H7N3O/c16-10-6-7-8-2-1-5-15(12(10)14-8)9-3-4-13-11(7)9/h1-6,13H
G-27	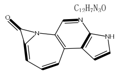 $C_{12}H_7N_3O$	24.4014	201.9059	-10.4277	95.783	329.2019	InChI=1S/C12H7N3O/c16-12-9-3-1-2-7-8-4-5-13-11(8)14-6-10(7)15(9)12/h1-6H,(H,13,14)
q1-47	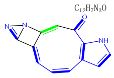 $C_{12}H_7N_3O$	25.8758	160.9622	-9.2859	126.9452	329.2413	InChI=1S/C12H7N3O/c16-10-6-9-8(12-14-15(9)12)3-1-2-7-4-5-13-11(7)10/h1-6,13H/b2-1-,8-3+,9-6+
x6-19	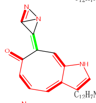 $C_{12}H_7N_3O$	25.1396	130.4511	-18.0599	162.6484	329.2563	InChI=1S/C12H7N3O/c16-10-3-1-2-7-4-5-13-9(7)6-8(10)11-12-14-15(11)12/h1-6,13H/b3-1-,7-2-,9-6+,11-8+
x6-28	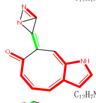 $C_{12}H_7N_3O$	25.1398	130.4462	-18.0624	162.6538	329.2563	InChI=1S/C12H7N3O/c16-10-3-1-2-7-4-5-13-9(7)6-8(10)11-12-14-15(11)12/h1-6,13H/b3-1-,7-2-,9-6+,11-8-
D-12	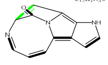 $C_{12}H_7N_3O$	6.3423	186.5784	-1.2905	115.3382	329.3851	InChI=1S/C12H7N3O/c16-12-8-6-10-11-7(3-5-14-11)9(15(10)12)2-1-4-13-8/h1-6,14H/b2-1-,4-1-,9-2-,13-4-,13-8-
B-149	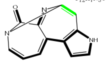 $C_{12}H_7N_3O$	7.9676	47.8663	-3.0484	244.2945	329.4064	InChI=1S/C12H7N3O/c16-12-11-8-2-1-5-15(12)10(14-11)6-9-7(8)3-4-13-9/h1-6,13H
A2-52	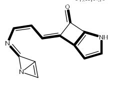 $C_{12}H_7N_3O$	22.2939	297.6044	-16.8012	18.3984	329.4496	InChI=1S/C12H7N3O/c16-11-8(7-3-5-13-10(7)11)2-1-4-14-12-9-6-15(9)12/h1-6,13H/b4-1-,8-2-,14-12+
n-39	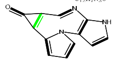 $C_{12}H_7N_3O$	32.1661	235.7711	-14.0655	58.758	329.5284	InChI=1S/C12H7N3O/c16-11-7-6-14-12-9(3-4-13-12)15-5-1-2-8(15)10(7)11/h1-6,13H/b14-6+
B-245	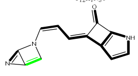 $C_{12}H_7N_3O$	22.7354	214.2626	-11.1336	96.2573	329.8089	InChI=1S/C12H7N3O/c16-11-8(7-3-4-13-10(7)11)2-1-5-15-6-9-12(15)14-9/h1-6,13H/b5-1-,8-2-

G-80	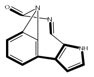		20.8215	184.3881	-10.7917	116.717	329.9656	InChI=1S/C12H7N3O/c16-12-14-6-9-7(4-5-13-9)8-2-1-3-10-11(8)15(10)12/h1-6,13H/b14-6+
X-38	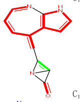	$C_{12}H_7N_3O$	27.2207	299.0374	-17.4719	15.2944	330.1413	InChI=1S/C12H7N3O/c16-12-10-9(15(10)12)6-7-2-1-4-13-11-8(7)3-5-14-11/h1-6,14H
q1-25	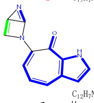	$C_{12}H_7N_3O$	3.8077	320.7917	-3.8746	-6.9	330.3065	InChI=1S/C12H7N3O/c16-11-9(15-6-8-12(15)14-8)3-1-2-7-4-5-13-10(7)11/h1-6,13H
B-222	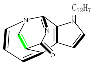	$C_{12}H_7N_3O$	6.9967	25.6906	-2.5947	252.3510	330.3711	InChI=1S/C12H7N3O/c16-12-9-6-15-5-1-2-7(9)8-3-4-13-10(8)11(15)14-12/h1-6,13H
H1-86	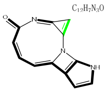	$C_{12}H_7N_3O$	29.2675	232.3062	-14.6918	54.3331	330.5339	InChI=1S/C12H7N3O/c16-11-3-1-2-9-7-4-5-13-12(7)15(9)10-6-8(10)14-11/h1-6,13H/b3-1-,9-2-,14-8-
H11-58	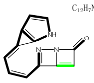	$C_{12}H_7N_3O$	19.5947	247.5686	-12.2697	67.4285	330.604	InChI=1S/C12H7N3O/c16-11-6-10-9(14-15(10)11)3-1-2-7-8-4-5-13-12(7)8/h1-6,13H/b3-1-,7-2-
H1-52	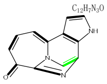	$C_{12}H_7N_3O$	6.097	110.2737	-2.5903	185.4815	330.8791	InChI=1S/C12H7N3O/c16-10-3-1-2-9-7-4-5-13-11(7)8-6-15(9)12(10)14-8/h1-6,13H
B-234	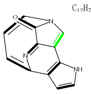	$C_{12}H_7N_3O$	9.1254	116.6030	-1.7667	178.0672	331.1962	InChI=1S/C12H7N3O/c16-12-11-8-6-15(12)5-1-2-9(14-11)7-3-4-13-10(7)8/h1-6,13H/b5-1-,9-2-
x5-42	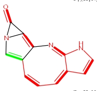	$C_{12}H_7N_3O$	14.0647	223.2202	-6.5934	94.1026	331.2831	InChI=1S/C12H7N3O/c16-12-10-9-8(6-15(10)12)3-1-2-7-4-5-13-11(7)14-9/h1-6H,(H,13,14)/b3-1-,7-2-
F-70	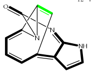	$C_{12}H_7N_3O$	10.4757	201.8327	-7.0701	100.2318	331.2863	InChI=1S/C12H7N3O/c16-12-14-11-8(4-5-13-11)7-2-1-3-9-10(6-7)15(9)12/h1-6H,(H,13,14,16)/b8-7-
p1-40	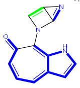	$C_{12}H_7N_3O$	4.254	321.4149	-3.7151	-5.8399	331.3219	InChI=1S/C12H7N3O/c16-9-3-1-2-7-4-5-13-10(7)11(9)15-6-8-12(15)14-8/h1-6,13H
A2-6	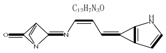	$C_{12}H_7N_3O$	22.6442	255.5083	-15.3912	59.6908	331.3838	InChI=1S/C12H7N3O/c16-12-9-6-15(12)11(9)14-4-1-2-7-8-3-5-13-10(7)8/h1-6,13H/b4-1-,7-2-,14-11+
r-31	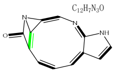	$C_{12}H_7N_3O$	19.0139	203.7369	-10.9016	116.8254	331.6135	InChI=1S/C12H7N3O/c16-12-8-3-1-2-7-4-5-13-11(7)14-6-9-10(8)15(9)12/h1-6H,(H,13,14)
B-109	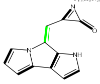	$C_{12}H_7N_3O$	3.0749	333.9557	-2.4695	-6.1700	331.6171	InChI=1S/C12H7N3O/c16-12-8(14-12)6-10-11-7(3-4-13-11)9-2-1-5-15(9)10/h1-6,13H/b10-6+
F-13	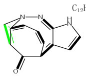	$C_{12}H_7N_3O$	8.1316	84.0056	-2.6531	209.865	331.625	InChI=1S/C12H7N3O/c16-11-7-2-1-3-10-9(11)6-15(10)14-12-8(7)4-5-13-12/h1-6H,(H,13,14)/b8-7+
o-79	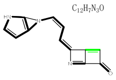	$C_{12}H_7N_3O$	16.1713	176.2141	-12.2469	126.979	331.6479	InChI=1S/C12H7N3O/c16-10-6-7-8(14-11(7)10)2-1-5-15-9-3-4-13-12(9)15/h1-6,13H/b5-1-,8-2+
o-145	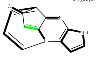	$C_{12}H_7N_3O$	9.7408	80.0743	-1.7626	202.6726	331.6808	InChI=1S/C12H7N3O/c16-10-6-9-11-7(10)2-1-5-15(9)8-3-4-13-12(8)14-11/h1-6,13H
C-53	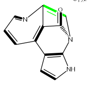	$C_{12}H_7N_3O$	6.4407	125.8465	-3.0804	172.2254	331.7248	InChI=1S/C12H7N3O/c16-12-10-7-2-1-4-13-9(10)6-15(12)11-8(7)3-5-14-11/h1-6,14H
k1-18	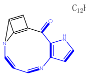	$C_{12}H_7N_3O$	16.8175	158.1135	-11.22	147.9749	331.7926	InChI=1S/C12H7N3O/c16-12-7-6-9-11(7)15(9)5-1-3-13-8-2-4-14-10(8)12/h1-6,14H/b5-1-,13-3-

$C_{12}H_7N_3O$

p1-24	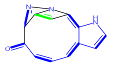	10.9482	147.4734	-3.4674	143.1155	331.9131	InChI=1S/C12H7N3O/c16-10-3-1-2-7-4-5-13-11(7)8-6-9-12(10)14-15(8)9/h1-6,13H/b3-1-,7-2-,11-8-
q11-48	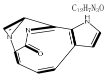	10.0202	190.8876	-5.1363	97.5052	332.0808	InChI=1S/C12H7N3O/c16-12-8-3-1-2-7-4-5-13-10(7)11(14-12)9-6-15(8)9/h1-6,13H/b2-1-,8-3+
H11-139	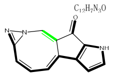	16.8839	190.0491	-3.7657	95.6086	332.1529	InChI=1S/C12H7N3O/c16-12-9-6-15-10(14-15)3-1-2-7(9)8-4-5-13-11(8)12/h1-6,13H/b3-1-,7-2-,9-6+
o-13	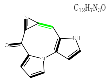	16.927	190.8585	-8.8846	93.8056	332.4291	InChI=1S/C12H7N3O/c16-12-10-2-1-5-15(10)9-3-4-13-7(9)6-8-11(12)14-8/h1-6,13H/b8-6+
x4-32	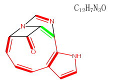	9.878	187.6349	-1.1636	107.9719	332.4739	InChI=1S/C12H7N3O/c16-12-8-3-1-2-7-4-5-13-9(7)10-11(12)15(8)6-14-10/h1-6,13H/b2-1-,8-3+
o-107	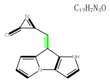	2.901	334.5617	-2.4463	-6.17	332.6181	InChI=1S/C12H7N3O/c16-12-8(14-12)6-7-9-2-1-5-15(9)10-3-4-13-11(7)10/h1-6,13H/b7-6+
p-24	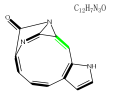	15.463	217.275	-6.2119	96.1616	332.8611	InChI=1S/C12H7N3O/c16-12-8-3-1-2-7-4-5-13-9(7)6-10-11(14-8)15(10)12/h1-6,13H/b2-1-,3-1-,7-2-,8-3-,9-6+,10-6+
o-3	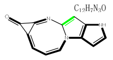	18.2406	222.3613	-9.4373	84.4834	332.9272	InChI=1S/C12H7N3O/c16-12-7-2-1-5-15-9-3-4-13-8(9)6-10(15)14-11(7)12/h1-6,13H/b5-1-,7-2+,14-11+
H1-25	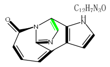	5.0164	102.2729	-2.2529	204.1877	332.9873	InChI=1S/C12H7N3O/c16-10-3-1-2-8-7-4-5-13-11(7)9-6-14-12(8)15(9)10/h1-6,13H
A-188	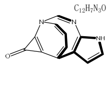	30.1092	206.1159	-11.8249	78.2583	333.0942	InChI=1S/C12H7N3O/c16-11-9-7-2-1-5-15(10(9)11)6-14-12-8(7)3-4-13-12/h1-6,13H/b14-6-
X22-44	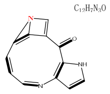	10.4703	243.6752	-7.9162	56.9717	333.2431	InChI=1S/C12H7N3O/c16-12-7-6-15-9(11(7)15)2-1-4-13-8-3-5-14-10(8)12/h1-6,14H/b2-1-,13-4-
x4-43	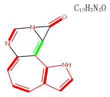	16.8683	220.0994	-5.7104	88.6999	333.322	InChI=1S/C12H7N3O/c16-12-11-9-8(14-6-15(11)12)3-1-2-7-4-5-13-10(7)9/h1-6,13H
q1-5	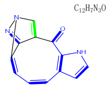	11.6463	109.3915	-7.3654	192.9686	333.4662	InChI=1S/C12H7N3O/c16-12-8-6-15-9(11(8)14-15)3-1-2-7-4-5-13-10(7)12/h1-6,13H/b2-1-,9-3+
q1-44	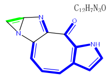	18.001	227.8925	-12.7477	77.8378	333.5158	InChI=1S/C12H7N3O/c16-12-10-7(4-5-13-10)2-1-3-8-11(12)14-9-6-15(8)9/h1-6,13H/b2-1-,8-3+
G-41	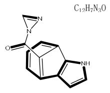	25.3092	164.5878	-11.5449	135.9675	333.6094	InChI=1S/C12H7N3O/c16-12(15-6-14-15)10-7-2-1-3-9(10)11-8(7)4-5-13-11/h1-6,13H
H11-113	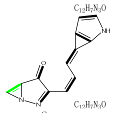	28.17	264.4491	-19.4634	41.5302	333.6596	InChI=1S/C12H7N3O/c16-12-9(14-15-6-10(12)15)3-1-2-7-8-4-5-13-11(7)8/h1-6,13H/b3-1-,7-2-
H11-97	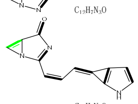	31.7296	272.4363	15.0725	36.2329	333.6722	InChI=1S/C12H7N3O/c16-12-9-6-15(9)10(14-12)3-1-2-7-8-4-5-13-11(7)8/h1-6,13H/b3-1-,7-2-
B-242	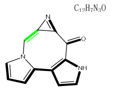	13.5278	196.6577	-6.6820	87.6995	333.8300	InChI=1S/C12H7N3O/c16-12-10-7(3-4-13-10)9-2-1-5-15(9)6-8-11(12)14-8/h1-6,13H/b8-6+
B-79	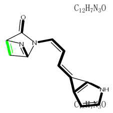	19.5665	194.5424	-13.1393	117.7632	334.0500	InChI=1S/C12H7N3O/c16-12-9-6-10(14-9)15(12)5-1-2-7-8-3-4-13-11(7)8/h1-6,13H/b5-1-,7-2-

F-25	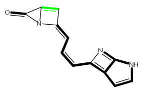 $C_{12}H_7N_3O$	18.0432	281.6491	-10.3816	47.6965	334.1887	InChI=1S/C12H7N3O/c16-12-10-6-7(15(10)12)2-1-3-9-8-4-5-13-11(8)14-9/h1-6H,(H,13,14)/b3-1-,7-2-
A2-38	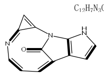 $C_{12}H_7N_3O$	13.7883	240.6196	-9.4275	64.3984	334.5143	InChI=1S/C12H7N3O/c16-12-8-2-1-4-13-9-6-10(9)15(12)11-7(8)3-5-14-11/h1-6,14H/b4-1-,8-2-,13-9-
n-12	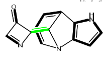 $C_{12}H_7N_3O$	6.4967	154.4565	-3.5067	148.8136	334.6785	InChI=1S/C12H7N3O/c16-9-6-14-11(9)12-7-2-1-5-15(12)8-3-4-13-10(7)8/h1-6,13H/b12-11-
X23-4	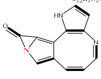 $C_{12}H_7N_3O$	7.2476	339.466	-5.0468	-5.7	335.0525	InChI=1S/C12H7N3O/c16-12-11-9-7(6-15(11)12)2-1-4-13-8-3-5-14-10(8)9/h1-6,14H/b2-1-,13-4-
X23-21	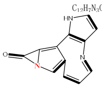 $C_{12}H_7N_3O$	7.2457	339.4678	-5.0468	-5.7	335.0525	InChI=1S/C12H7N3O/c16-12-11-9-7(6-15(11)12)2-1-4-13-8-3-5-14-10(8)9/h1-6,14H/b2-1-,13-4+
r1-29	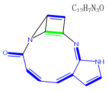 $C_{12}H_7N_3O$	18.8639	223.1337	-11.7074	97.9347	335.4465	InChI=1S/C12H7N3O/c16-10-3-1-2-7-4-5-13-12(7)14-8-6-9-11(8)15(9)10/h1-6H,(H,13,14)/b3-1-,7-2-
o-5	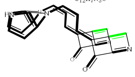 $C_{12}H_7N_3O$	16.4295	178.6009	-12.2921	127.8096	335.4469	InChI=1S/C12H7N3O/c16-11-7(8-6-14-10(8)11)2-1-5-15-9-3-4-13-12(9)15/h1-6,13H/b5-1-,7-2+
p11-20	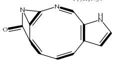 $C_{12}H_7N_3O$	22.7589	217.8445	-13.3048	82.3133	335.4713	InChI=1S/C12H7N3O/c16-12-8-3-1-2-7-4-5-13-9(7)6-14-11-10(8)15(11)12/h1-6,13H/b2-1-,3-1-,7-2-,8-3+,9-6+,14-6-,14-11+
H11-122	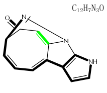 $C_{12}H_7N_3O$	11.1866	99.7375	-2.7149	175.0327	335.5051	InChI=1S/C12H7N3O/c16-11-6-10-7-2-1-3-9(11)14-15(10)12-8(7)4-5-13-12/h1-6,13H/b3-1-,7-2-
q-10	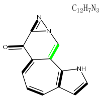 $C_{12}H_7N_3O$	19.4414	180.8494	-2.156	113.7445	335.5104	InChI=1S/C12H7N3O/c16-11-8-3-1-2-7-4-5-13-10(7)9(8)6-15-12(11)14-15/h1-6,13H
B-98	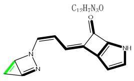 $C_{12}H_7N_3O$	15.8167	305.5684	-13.2833	22.6707	335.5370	InChI=1S/C12H7N3O/c16-12-8(7-3-4-13-11(7)12)2-1-5-15-10-6-9(10)14-15/h1-6,13H/b5-1-,8-2-
A1-42	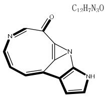 $C_{12}H_7N_3O$	17.6926	215.6149	-10.2434	77.8047	335.6108	InChI=1S/C12H7N3O/c16-9-6-13-4-1-2-7-8-3-5-14-12(8)15-10(7)11(9)15/h1-6,14H/b4-1-,7-2-,13-6-
H-66	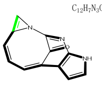 $C_{12}H_7N_3O$	18.1452	185.7179	-10.4126	102.6894	335.6184	InChI=1S/C12H7N3O/c16-10-8-3-1-2-7-6-15(7)12(10)14-11-9(8)4-5-13-11/h1-6,13H/b2-1-,8-3-
A2-50	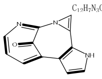 $C_{12}H_7N_3O$	22.0931	202.3427	-12.5906	81.7261	335.7968	InChI=1S/C12H7N3O/c16-11-8-2-1-4-14-12(11)15-6-9(15)10-7(8)3-5-13-10/h1-6,13H
B-257	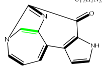 $C_{12}H_7N_3O$	8.4961	42.0850	-3.8879	239.5820	335.8619	InChI=1S/C12H7N3O/c16-11-10-8(3-4-13-10)7-2-1-5-15-6-9(7)14-12(11)15/h1-6,13H
H-57	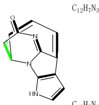 $C_{12}H_7N_3O$	7.3856	122.383	-2.2018	173.7937	335.9411	InChI=1S/C12H7N3O/c16-10-6-7-2-1-3-8-9-4-5-13-11(9)15(7)12(8)14-10/h1-6,13H
s11-40	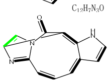 $C_{12}H_7N_3O$	4.2513	102.4648	-2.7962	210.8065	336.2329	InChI=1S/C12H7N3O/c16-12-10-8-6-15(12)9(14-8)3-1-2-7-4-5-13-11(7)10/h1-6,13H/b3-1-,7-2-,11-10-
o-233	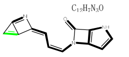 $C_{12}H_7N_3O$	15.8038	311.7954	-13.5174	27.6972	336.309	InChI=1S/C12H7N3O/c16-12-11-10(3-4-13-11)15(12)5-1-2-8-7-6-9(7)14-8/h1-6,13H/b5-1-,8-2+
o-248	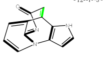 $C_{12}H_7N_3O$	6.0276	90.7349	-1.4137	196.8781	336.4234	InChI=1S/C12H7N3O/c16-10-6-8-7-2-1-5-15(12(7)14-10)9-3-4-13-11(8)9/h1-6,13H

p1-16	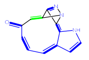 $C_{12}H_7N_3O$	27.9003	118.2154	-12.4422	165.3428	336.4681	InChI=1S/C12H7N3O/c16-8-3-1-2-7-4-5-13-10(7)12-11-9(6-8)15(12)14-11/h1-6,13H/b3-1-,7-2-,9-6-,12-10+
o-254	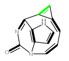 $C_{12}H_7N_3O$	2.0165	299.8684	-1.751	35.6676	336.5124	InChI=1S/C12H7N3O/c16-12-14-10-8-6-7(8)2-1-5-15(12)9-3-4-13-11(9)10/h1-6,13H/b5-1-,7-2-
q11-45	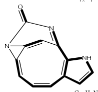 $C_{12}H_7N_3O$	12.7815	209.7689	-5.8231	103.0075	336.5238	InChI=1S/C12H7N3O/c16-12-14-8-6-10-9(15(10)12)3-1-2-7-4-5-13-11(7)8/h1-6,13H/b2-1-,9-3+
s11-53	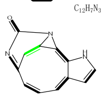 $C_{12}H_7N_3O$	11.1238	209.9077	-5.1073	105.4582	336.7171	InChI=1S/C12H7N3O/c16-12-14-8-3-1-2-7-4-5-13-10(7)11-9(6-8)15(11)12/h1-6,13H/b3-1-,7-2-,11-10-
s12-16	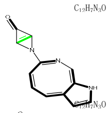 $C_{12}H_7N_3O$	54.8706	211.7163	-32.8787	71.295	336.8211	InChI=1S/C12H7N3O/c16-12-10-11(12)15(10)9-3-1-2-7-4-5-13-8(7)6-14-9/h1-6,13H/b2-1-,3-1-,7-2-,8-6+,9-3+,14-6-,14-9+
i-28	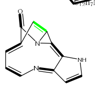 $C_{12}H_7N_3O$	14.7854	208.7932	-6.4856	114.8468	336.9067	InChI=1S/C12H7N3O/c16-12-7-2-1-4-13-8-3-5-14-10(8)11-9(6-7)15(11)12/h1-6,14H/b2-1-,4-1-,7-2+,11-10+,13-4+,13-8+
H-60	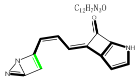 $C_{12}H_7N_3O$	26.0085	218.0912	-9.7625	96.1033	337.3351	InChI=1S/C12H7N3O/c16-12-9(8-4-5-13-11(8)12)3-1-2-7-6-10-14-15(7)10/h1-6,13H/b2-1-,9-3-
G-6	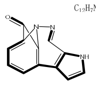 $C_{12}H_7N_3O$	10.0939	128.7254	-3.46	160.0812	337.4775	InChI=1S/C12H7N3O/c16-12-10-3-1-2-8-7-4-5-13-9(7)6-14-15(10)11(8)12/h1-6,13H
H11-106	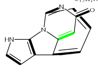 $C_{12}H_7N_3O$	10.1817	126.4028	-2.7997	167.3055	337.918	InChI=1S/C12H7N3O/c16-11-6-9-7-2-1-3-10(14-11)15(9)12-8(7)4-5-13-12/h1-6,13H
x9-1	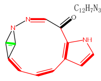 $C_{12}H_7N_3O$	35.3336	235.4143	-22.3718	71.0153	338.154	InChI=1S/C12H7N3O/c16-9-6-14-15-11-8(12(11)15)3-1-2-7-4-5-13-10(7)9/h1-6,13H/b2-1-,8-3-,14-6-
o-18	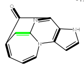 $C_{12}H_7N_3O$	7.7278	35.6726	-2.2317	249.6561	338.2417	InChI=1S/C12H7N3O/c16-12-7-2-1-5-15-8-3-4-13-10(8)11(12)14-9(15)6-7/h1-6,13H
o-179	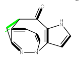 $C_{12}H_7N_3O$	7.858	70.9104	-2.4469	224.9587	338.3936	InChI=1S/C12H7N3O/c16-12-8-6-9-7(8)2-1-5-15(14-9)10-3-4-13-11(10)12/h1-6,13H
I-99	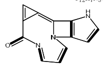 $C_{12}H_7N_3O$	25.8483	245.7115	-13.318	48.2028	338.3965	InChI=1S/C12H7N3O/c16-12-8-6-7(8)11-10-9(2-4-13-10)15(11)5-1-3-14-12/h1-6,13H/b5-1-,11-7-,14-3+
E-16	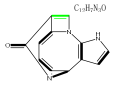 $C_{12}H_7N_3O$	8.1091	72.7688	-3.2688	224.3353	338.4704	InChI=1S/C12H7N3O/c16-12-8-6-15-10(8)3-1-2-9(14-12)7-4-5-13-11(7)15/h1-6,13H/b2-1-,10-3-,14-9+
B-68	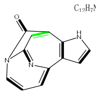 $C_{12}H_7N_3O$	11.8574	109.6420	-2.7712	186.5797	338.5070	InChI=1S/C12H7N3O/c16-12-8-6-10-14-9(2-1-5-15(10)12)7-3-4-13-11(7)8/h1-6,13H
C-36	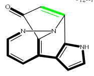 $C_{12}H_7N_3O$	14.4312	92.3847	-3.8004	196.1578	338.5475	InChI=1S/C12H7N3O/c16-10-6-9-11-7(3-5-13-11)8-2-1-4-14-15(9)12(8)10/h1-6,13H
H-84	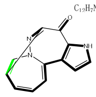 $C_{12}H_7N_3O$	7.3195	44.3855	-3.0549	243.7567	338.6053	InChI=1S/C12H7N3O/c16-12-10-7-2-1-3-9(15(6-7)14-10)8-4-5-13-11(8)12/h1-6,13H
B-102	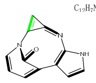 $C_{12}H_7N_3O$	13.1263	221.6240	-10.6649	88.0241	338.7482	InChI=1S/C12H7N3O/c16-12-8-2-1-5-15(12)10-6-9(10)14-11-7(8)3-4-13-11/h1-6,13H/b14-9-
G-12	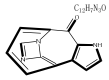 $C_{12}H_7N_3O$	6.7823	126.9748	-1.5787	176.1931	338.8253	InChI=1S/C12H7N3O/c16-11-9-3-1-2-8(12-14-6-15(9)12)7-4-5-13-10(7)11/h1-6,13H

A1-19	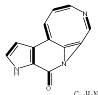	$C_{12}H_7N_3O$	17.5665	253.4454	-9.8606	32.0108	338.8317	InChI=1S/C12H7N3O/c16-12-10-7(3-5-14-10)8-2-1-4-13-6-9-11(8)15(9)12/h1-6,14H/b2-1-,4-1-,8-2-,9-6+,13-4-,13-6-
H11-109	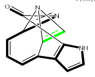	$C_{12}H_7N_3O$	10.3379	203.4154	-4.026	85.4265	338.8862	InChI=1S/C12H7N3O/c16-12-11-8(4-5-13-11)7-2-1-3-10(14-12)15-6-9(7)15/h1-6,13H/b14-10-
G-52	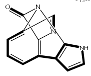	$C_{12}H_7N_3O$	5.7939	108.1391	-2.1437	193.9557	338.9486	InChI=1S/C12H7N3O/c16-12-10-8-3-1-2-7(6-14-10)15(12)11-9(8)4-5-13-11/h1-6,13H/b2-1-,3-1-,7-2+,8-3-
s11-4	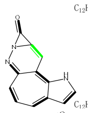	$C_{12}H_7N_3O$	22.0201	269.3135	-7.729	32.9072	338.9592	InChI=1S/C12H7N3O/c16-12-10-6-8-9(14-15(10)12)3-1-2-7-4-5-13-11(7)8/h1-6,13H
H11-104	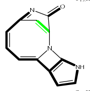	$C_{12}H_7N_3O$	10.0448	191.2616	-2.4097	106.2139	338.9683	InChI=1S/C12H7N3O/c16-12-10-6-7(14-12)2-1-3-9-8-4-5-13-11(8)15(9)10/h1-6,13H/b2-1-,9-3-
i-12	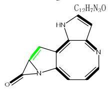	$C_{12}H_7N_3O$	16.1725	220.287	-5.893	87.2593	339.0965	InChI=1S/C12H7N3O/c16-12-10-6-7-9(15(10)12)2-1-4-13-8-3-5-14-11(7)8/h1-6,14H/b2-1-,4-1-,9-2+,11-7+,13-4-,13-8-
A1-49	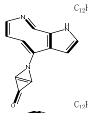	$C_{12}H_7N_3O$	55.5152	218.7234	-29.0852	52.3091	339.4702	InChI=1S/C12H7N3O/c16-12-10-11(12)15(10)9-2-1-4-13-6-8-7(9)3-5-14-8/h1-6,14H/b2-1+,4-1-,8-6-,9-2-,9-7+,13-4-,13-6+
o-9	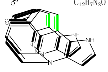	$C_{12}H_7N_3O$	9.4343	111.4506	-2.7987	181.5331	339.6004	InChI=1S/C12H7N3O/c16-12-7-2-1-5-15-9-3-4-13-10(9)8(6-7)11(12)14-15/h1-6,13H/b5-1-,7-2-
x9-42	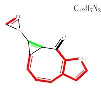	$C_{12}H_7N_3O$	30.3636	233.7751	-23.8712	59.1604	339.6402	InChI=1S/C12H7N3O/c16-12-9-8(11(9)15-6-14-15)3-1-2-7-4-5-13-10(7)12/h1-6,13H/b2-1-,8-3+
l-61	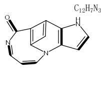	$C_{12}H_7N_3O$	13.4506	120.4575	-4.6611	166.5437	339.7146	InChI=1S/C12H7N3O/c16-12-10-7-6-9(10)15(5-1-3-14-12)8-2-4-13-11(7)8/h1-6,13H/b5-1-,14-3-
X-34	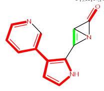	$C_{12}H_7N_3O$	27.8730	299.1631	-16.0762	16.9733	339.9600	InChI=1S/C12H7N3O/c16-12-11-10(15(11)12)9-8(3-5-14-9)7-2-1-4-13-6-7/h1-6,14H
p-14	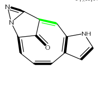	$C_{12}H_7N_3O$	20.2351	162.081	-3.9076	134.5841	339.9657	InChI=1S/C12H7N3O/c16-11-8-6-9-7(4-5-13-9)2-1-3-10(11)15-12(8)14-15/h1-6,13H/b2-1-,8-6+,10-3+
o-230	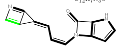	$C_{12}H_7N_3O$	25.6556	238.1183	-14.0606	91.9452	339.9695	InChI=1S/C12H7N3O/c16-12-11-9(3-4-13-11)15(12)5-1-2-7-8-6-14-10(7)8/h1-6,13H/b5-1-,7-2+
o-77	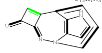	$C_{12}H_7N_3O$	6.7134	145.262	-2.141	153.9927	340.149	InChI=1S/C12H7N3O/c16-10-6-8-7-2-1-5-15(14-12(8)10)9-3-4-13-11(7)9/h1-6,13H
H1-122	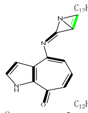	$C_{12}H_7N_3O$	21.1659	219.0202	-17.1838	99.1204	340.1576	InChI=1S/C12H7N3O/c16-10-3-1-2-8(7-4-5-13-11(7)10)14-12-9-6-15(9)12/h1-6,13H/b14-12+
H-160	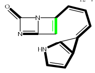	$C_{12}H_7N_3O$	18.8293	246.3922	-11.7981	85.2565	340.4883	InChI=1S/C12H7N3O/c16-12-14-10-6-7(15(10)12)2-1-3-8-9-4-5-13-11(8)9/h1-6,13H/b2-1-,8-3-
o-213	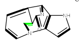	$C_{12}H_7N_3O$	6.89	28.3364	-2.491	257.0335	340.6312	InChI=1S/C12H7N3O/c16-12-8-6-15-5-1-2-7(8)10(14-12)11-9(15)3-4-13-11/h1-6,13H
o-214	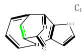	$C_{12}H_7N_3O$	6.8892	28.3366	-2.4911	257.0333	340.6312	InChI=1S/C12H7N3O/c16-12-9-6-7-2-1-5-15(9)8-3-4-13-11(8)10(7)14-12/h1-6,13H
p11-22	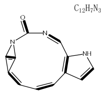	$C_{12}H_7N_3O$	47.5233	255.5096	-26.997	51.4866	340.6455	InChI=1S/C12H7N3O/c16-12-14-6-9-7(4-5-13-9)2-1-3-8-10-11(8)15(10)12/h1-6,13H/b2-1-,8-3-,14-6-

q1-4	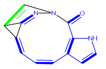	15.001	163.3035	-9.7754	142.7326	340.8909	InChI=1S/C12H7N3O/c16-12-10-7(4-5-13-10)2-1-3-8-9-6-15(12)14-11(8)9/h1-6,13H/b2-1-,8-3-
H1-43	$C_{12}H_7N_3O$ 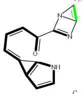	32.1367	270.6311	-22.0138	42.194	340.9394	InChI=1S/C12H7N3O/c16-9(12-14-10-6-15(10)12)3-1-2-7-8-4-5-13-11(7)8/h1-6,13H/b3-1-,7-2-
A-12	$C_{12}H_7N_3O$ 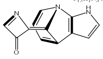	7.8283	163.8026	-3.318	145.9023	341.0126	InChI=1S/C12H7N3O/c16-9-6-14-10(9)11-7-2-1-5-15(11)12-8(7)3-4-13-12/h1-6,13H/b11-10-
H1-107	$C_{12}H_7N_3O$ 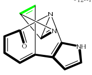	15.4403	166.0341	-3.1273	149.5285	341.2372	InChI=1S/C12H7N3O/c16-10-3-1-2-7-6-9-12(14-9)15(10)11-8(7)4-5-13-11/h1-6,13H/b3-1-,7-2+
o-239	$C_{12}H_7N_3O$ 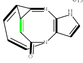	23.6941	212.579	-10.3956	83.8807	341.2591	InChI=1S/C12H7N3O/c16-10-6-8-7-2-1-5-15(10)9-3-4-13-12(9)14-11(7)8/h1-6,13H/b5-1-,7-2+,8-6-,14-11-
n-46	$C_{12}H_7N_3O$ 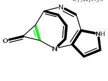	29.4146	212.8899	-11.5776	74.2928	341.2777	InChI=1S/C12H7N3O/c16-12-10-7-2-1-5-15(11(10)12)9-3-4-13-8(9)6-14-7/h1-6,13H/b14-6-
C-41	$C_{12}H_7N_3O$ 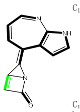	4.4506	351.5815	-4.3287	-5.7000	341.4528	InChI=1S/C12H7N3O/c16-10-6-9-11(15(9)10)7-2-1-4-13-12-8(7)3-5-14-12/h1-6,14H/b11-7-
H11-47	$C_{12}H_7N_3O$ 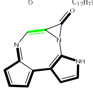	16.6399	257.5302	-6.462	65.7434	341.4878	InChI=1S/C12H7N3O/c16-12-10-6-14-9-3-1-2-7(9)8-4-5-13-11(8)15(10)12/h1-6,13H/b10-6-,14-9-
H11-154	$C_{12}H_7N_3O$ 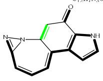	26.4572	163.5921	-4.3551	128.0526	341.5085	InChI=1S/C12H7N3O/c16-10-6-9-7(8-4-5-13-12(8)10)2-1-3-11-14-15(9)11/h1-6,13H
B-227	$C_{12}H_7N_3O$ 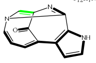	5.3579	34.9085	-1.6482	272.4896	341.5742	InChI=1S/C12H7N3O/c16-11-8-2-1-5-15-6-9(11)14-12(15)10-7(8)3-4-13-10/h1-6,13H/b5-1-,8-2-
H-155	$C_{12}H_7N_3O$ 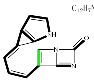	18.8088	247.4619	-11.7108	84.8688	341.6548	InChI=1S/C12H7N3O/c16-12-14-11-7(6-15(11)12)2-1-3-8-9-4-5-13-10(8)9/h1-6,13H/b2-1-,8-3-
q-15	$C_{12}H_7N_3O$ 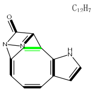	11.6599	174.3275	-4.9924	95.8393	341.8057	InChI=1S/C12H7N3O/c16-12-11-9-6-8(15(12)14-11)3-1-2-7-4-5-13-10(7)9/h1-6,13H/b2-1-,3-1-,7-2-,8-3+,10-9-
p-6	$C_{12}H_7N_3O$ 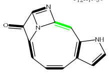	6.8366	153.3183	-5.0923	162.1241	342.1793	InChI=1S/C12H7N3O/c16-11-9-3-1-2-7-4-5-13-8(7)6-10-14-12(11)15(9)10/h1-6,13H/b2-1-,9-3-,10-6-
A-81	$C_{12}H_7N_3O$ 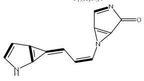	25.284	273.2003	-14.4318	39.9912	342.2298	InChI=1S/C12H7N3O/c16-12-11-9(6-14-12)15(11)5-1-2-7-8-3-4-13-10(7)8/h1-6,13H/b5-1-,7-2-
A-126	$C_{12}H_7N_3O$ 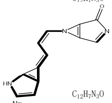	25.3688	273.0574	-14.4581	40.0425	342.3124	InChI=1S/C12H7N3O/c16-12-11-9(6-14-12)15(11)5-1-2-7-8-3-4-13-10(7)8/h1-6,13H/b5-1-,7-2-
A1-47	$C_{12}H_7N_3O$ 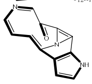	15.5637	210.0589	-7.7078	98.726	342.3528	InChI=1S/C12H7N3O/c16-9-6-13-4-1-2-8-7-3-5-14-10(7)12-11(9)15(8)12/h1-6,14H/b4-1-,8-2+,13-6-
G1-101	$C_{12}H_7N_3O$ 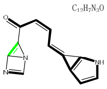	26.5141	272.8521	-17.2613	46.5652	342.5345	InChI=1S/C12H7N3O/c16-9(11-12-14-6-15(11)12)3-1-2-7-8-4-5-13-10(7)8/h1-6,13H/b3-1-,7-2-
q-29	$C_{12}H_7N_3O$ 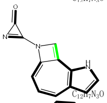	3.0595	347.1067	-2.7817	-11.68	342.5562	InChI=1S/C12H7N3O/c16-12-11(14-12)15-6-8-9(15)3-1-2-7-4-5-13-10(7)8/h1-6,13H
B-19	$C_{12}H_7N_3O$ 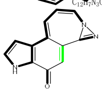	24.7065	166.7659	-6.6243	113.1278	342.6312	InChI=1S/C12H7N3O/c16-10-6-9-7(8-3-4-13-11(8)10)2-1-5-15-12(9)14-15/h1-6,13H

$C_{12}H_7N_3O$

A-24	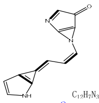 $C_{12}H_7N_3O$	24.8631	274.6012	-14.2849	39.9973	342.7898	InChI=1S/C12H7N3O/c16-9-6-14-12-11(9)15(12)5-1-2-7-8-3-4-13-10(7)8/h1-6,13H/b5-1-,7-2-
q1-22	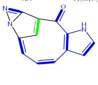 $C_{12}H_7N_3O$	20.386	163.2682	-4.2615	133.6545	342.8138	InChI=1S/C12H7N3O/c16-11-9-6-8(15-12(9)14-15)3-1-2-7-4-5-13-10(7)11/h1-6,13H/b2-1-,8-3+
A2-10	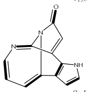 $C_{12}H_7N_3O$	14.9032	103.8318	-3.3891	199.3327	342.865	InChI=1S/C12H7N3O/c16-10-6-8-11-7(3-5-13-11)9-2-1-4-14-12(8)15(9)10/h1-6,13H
H1-87	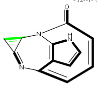 $C_{12}H_7N_3O$	23.2985	178.7529	-11.9692	132.0101	342.9071	InChI=1S/C12H7N3O/c16-11-3-1-2-8-7-4-5-13-12(7)15(11)10-6-9(10)14-8/h1-6,13H/b3-1-,8-2+
n-118	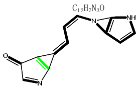 $C_{12}H_7N_3O$	25.2097	272.0734	-14.3146	39.8165	342.9219	InChI=1S/C12H7N3O/c16-9-6-14-11-7(10(9)11)2-1-5-15-8-3-4-13-12(8)15/h1-6,13H/b5-1-,7-2+
A-10	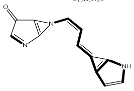 $C_{12}H_7N_3O$	24.9594	274.4688	-14.3194	40.0533	342.9464	InChI=1S/C12H7N3O/c16-9-6-14-12-11(9)15(12)5-1-2-7-8-3-4-13-10(7)8/h1-6,13H/b5-1-,7-2-
n-50	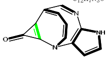 $C_{12}H_7N_3O$	29.8255	211.6253	-11.8679	77.7922	343.038	InChI=1S/C12H7N3O/c16-11-9-7-2-1-5-15(10(9)11)8-3-4-13-12(8)14-6-7/h1-6,13H/b14-6-
B-213	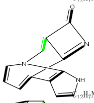 $C_{12}H_7N_3O$	7.0012	158.2152	-2.2356	147.8170	343.1748	InChI=1S/C12H7N3O/c16-12-9-6-15-5-1-2-7(10(9)14-12)8-3-4-13-11(8)15/h1-6,13H
o-25	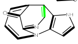 $C_{12}H_7N_3O$	6.4069	27.2816	-2.2409	269.506	343.3921	InChI=1S/C12H7N3O/c16-11-7-2-1-5-15-9-3-4-13-10(9)8(7)6-14-12(11)15/h1-6,13H
A-196	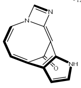 $C_{12}H_7N_3O$	6.6402	152.9018	-2.7797	163.7295	343.43	InChI=1S/C12H7N3O/c16-11-8-2-1-5-15-6-14-12(15)9(11)10-7(8)3-4-13-10/h1-6,13H/b5-1-,8-2-,12-9-
H11-117	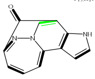 $C_{12}H_7N_3O$	8.2266	33.6961	-0.9124	254.2511	343.4601	InChI=1S/C12H7N3O/c16-12-8-6-15-10(3-1-2-9(12)14-15)7-4-5-13-11(7)8/h1-6,13H
p-20	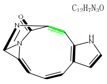 $C_{12}H_7N_3O$	11.4904	200.1261	-5.614	123.1249	343.687	InChI=1S/C12H7N3O/c16-12-9-6-8-7(4-5-13-8)2-1-3-10-11(14-9)15(10)12/h1-6,13H/b2-1-,3-1-,7-2-,8-6+,9-6+,10-3-
I-34	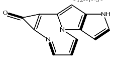 $C_{12}H_7N_3O$	27.8883	240.394	-11.2846	67.7402	343.6948	InChI=1S/C12H7N3O/c16-12-10-9-6-7-8(2-4-13-7)15(9)5-1-3-14-11(10)12/h1-6,13H/b5-1-,14-3+
E-22	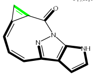 $C_{12}H_7N_3O$	2.4454	322.4384	-1.3686	11.1954	343.7677	InChI=1S/C12H7N3O/c16-12-9-6-7(9)2-1-3-10-8-4-5-13-11(8)15(12)14-10/h1-6,13H/b3-1-,7-2-
B-225	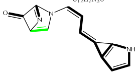 $C_{12}H_7N_3O$	19.5656	196.7825	-13.0672	124.1779	343.9435	InChI=1S/C12H7N3O/c16-11-9-6-15(12(11)14-9)5-1-2-7-8-3-4-13-10(7)8/h1-6,13H/b5-1-,7-2-
X22-16	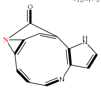 $C_{12}H_7N_3O$	23.3267	247.3793	-8.642	67.9826	343.9956	InChI=1S/C12H7N3O/c16-12-7-6-10-9(15(10)12)2-1-4-13-8-3-5-14-11(7)8/h1-6,14H/b2-1-,4-1-,9-2+,11-7-,13-4-,13-8-
k-40	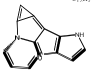 $C_{12}H_7N_3O$	28.9282	209.2907	-16.1624	105.9713	344.1004	InChI=1S/C12H7N3O/c16-12-10-7-6-9(7)15(12)5-1-3-13-8-2-4-14-11(8)10/h1-6,14H/b5-1-,13-3+
H-19	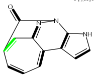 $C_{12}H_7N_3O$	10.5753	105.831	-4.2094	185.789	344.1949	InChI=1S/C12H7N3O/c16-12-7-2-1-3-8-9-4-5-13-11(9)15(12)14-10(8)6-7/h1-6,13H
I1-48	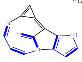 $C_{12}H_7N_3O$	15.6444	233.7757	-9.5521	76.382	344.296	InChI=1S/C12H7N3O/c16-12-10-7-6-8(7)13-3-1-5-15(12)9-2-4-14-11(9)10/h1-6,14H/b5-1-,10-7-,13-3-

n-29	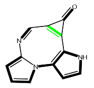 $C_{12}H_7N_3O$	29.5408	276.7119	-10.6153	28.9063	344.4339	InChI=1S/C12H7N3O/c16-12-7-6-14-9-2-1-5-15(9)8-3-4-13-11(8)10(7)12/h1-6,13H/b14-6-
I-90	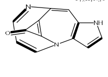 $C_{12}H_7N_3O$	30.9747	214.5762	-12.3833	75.3276	344.538	InChI=1S/C12H7N3O/c16-12-10-8-6-7-9(2-4-14-7)15(11(10)12)5-1-3-13-8/h1-6,14H/b5-1-,13-3-
B-80	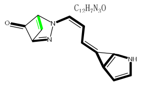 $C_{12}H_7N_3O$	19.6052	194.1136	-13.2924	133.7561	344.7994	InChI=1S/C12H7N3O/c16-12-9-6-10(12)15(14-9)5-1-2-7-8-3-4-13-11(7)8/h1-6,13H/b5-1-,7-2-
A2-27	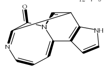 $C_{12}H_7N_3O$	7.8706	59.8038	-2.6803	244.8379	345.1971	InChI=1S/C12H7N3O/c16-12-11-8-6-15(12)9(2-1-4-13-11)7-3-5-14-10(7)8/h1-6,14H/b4-1-,9-2-,13-11+
X22-45	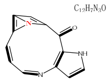 $C_{12}H_7N_3O$	13.0133	255.3054	-8.5552	50.7956	345.2312	InChI=1S/C12H7N3O/c16-12-10-6-9-8(15(9)10)2-1-4-13-7-3-5-14-11(7)12/h1-6,14H/b2-1-,13-4-
H1-120	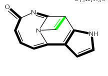 $C_{12}H_7N_3O$	11.8082	116.4633	-4.5121	178.3122	345.4397	InChI=1S/C12H7N3O/c16-10-3-1-2-9-7-4-5-13-11(7)8-6-15(9)12(8)14-10/h1-6,13H/b3-1-,9-2-,14-12-
o-150	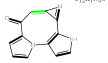 $C_{12}H_7N_3O$	19.2749	196.1112	-4.7853	106.7636	345.7449	InChI=1S/C12H7N3O/c16-10-6-7-11(14-7)12-9(3-4-13-12)15-5-1-2-8(10)15/h1-6,13H/b7-6+
o-58	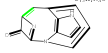 $C_{12}H_7N_3O$	6.7927	78.2316	-3.4083	224.6331	345.8865	InChI=1S/C12H7N3O/c16-11-8-6-7-2-1-5-15(12(11)14-8)9-3-4-13-10(7)9/h1-6,13H/b8-6-
A-215	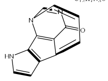 $C_{12}H_7N_3O$	27.2205	112.8724	-6.5043	147.4820	345.9276	InChI=1S/C12H7N3O/c16-12-9-7-2-1-5-15(6-14-12)11(9)10-8(7)3-4-13-10/h1-6,13H
X22-7	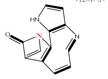 $C_{12}H_7N_3O$	8.6989	196.5303	-5.2672	118.8898	345.966	InChI=1S/C12H7N3O/c16-12-8-6-15(12)11-7(8)2-1-4-13-9-3-5-14-10(9)11/h1-6,14H/b2-1-,4-1+,7-2-,11-10-,13-4+,13-9-
B-148	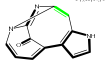 $C_{12}H_7N_3O$	10.4175	85.0375	-3.0521	215.5314	346.0132	InChI=1S/C12H7N3O/c16-11-8-2-1-5-15-10(14-12(11)15)6-9-7(8)3-4-13-9/h1-6,13H/b10-6-
k-28	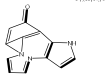 $C_{12}H_7N_3O$	5.4225	325.9488	-1.4559	0.87	346.0175	InChI=1S/C12H7N3O/c16-9-6-8-12-10(9)11-7(2-4-14-11)13-3-1-5-15(8)12/h1-6,14H/b5-1-,13-3+
H11-63	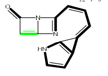 $C_{12}H_7N_3O$	19.1861	247.2316	-12.1016	91.9471	346.2341	InChI=1S/C12H7N3O/c16-11-6-10-14-9(15(10)11)3-1-2-7-8-4-5-13-12(7)8/h1-6,13H/b3-1-,7-2-
o-208	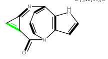 $C_{12}H_7N_3O$	16.8387	214.3139	-10.0793	101.8748	346.6231	InChI=1S/C12H7N3O/c16-12-7-6-9(7)14-8-2-1-5-15(12)10-3-4-13-11(8)10/h1-6,13H/b14-9-
n-114	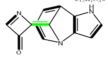 $C_{12}H_7N_3O$	12.9075	155.019	-4.5817	141.6958	346.8738	InChI=1S/C12H7N3O/c16-9-6-14-11(9)12-7-2-1-5-15(12)8-3-4-13-10(7)8/h1-6,13H/b12-11+
G-70	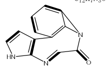 $C_{12}H_7N_3O$	13.9741	208.9391	-5.7467	124.9914	347.2313	InChI=1S/C12H7N3O/c16-10-6-14-12-8(4-5-13-12)7-2-1-3-9-11(7)15(9)10/h1-6,13H/b14-6+
x4-46	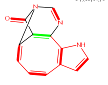 $C_{12}H_7N_3O$	11.0851	146.8777	-4.591	156.5231	347.3151	InChI=1S/C12H7N3O/c16-12-9-8-3-1-2-7-4-5-13-10(7)11(9)14-6-15(8)12/h1-6,13H/b2-1-,3-1-,7-2-,8-3+,11-10-
B-54	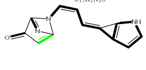 $C_{12}H_7N_3O$	19.4473	192.9077	-12.6764	138.0841	347.5067	InChI=1S/C12H7N3O/c16-9-6-10-14-12(9)15(10)5-1-2-7-8-3-4-13-11(7)8/h1-6,13H/b5-1-,7-2-
X-27	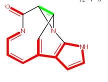 $C_{12}H_7N_3O$	32.8016	246.0185	-12.7483	37.7256	347.5645	InChI=1S/C12H7N3O/c16-12-10-9-6-7(2-1-4-14-12)8-3-5-13-11(8)15(9)10/h1-6,13H/b2-1-,14-4+

q1-45	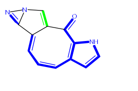 $C_{12}H_7N_3O$	18.2034	161.8705	-4.5004	152.0422	347.6037	InChI=1S/C12H7N3O/c16-11-9-6-15-12(14-15)8(9)3-1-2-7-4-5-13-10(7)11/h1-6,13H/b2-1-,8-3+
H1-141	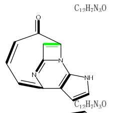 $C_{12}H_7N_3O$	6.6121	152.463	-1.516	164.5354	347.6637	InChI=1S/C12H7N3O/c16-10-3-1-2-8-7-4-5-13-12(7)15-9(10)6-11(15)14-8/h1-6,13H/b3-1-,8-2-
o-211	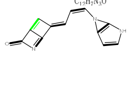 $C_{12}H_7N_3O$	15.8426	170.8558	-12.1393	146.6974	347.7367	InChI=1S/C12H7N3O/c16-12-8-6-7(10(8)14-12)2-1-5-15-9-3-4-13-11(9)15/h1-6,13H/b5-1-,7-2+
s11-10	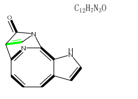 $C_{12}H_7N_3O$	9.0394	151.7148	-4.2375	150.8343	347.8085	InChI=1S/C12H7N3O/c16-12-8-6-15(12)11-10-7(4-5-13-10)2-1-3-9(8)14-11/h1-6,13H/b2-1-,3-1-,7-2-,9-3-,11-10-
H11-111	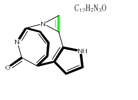 $C_{12}H_7N_3O$	12.8241	205.0944	-5.1018	94.6472	347.988	InChI=1S/C12H7N3O/c16-12-8-2-1-3-10(14-12)15-6-9(15)11-7(8)4-5-13-11/h1-6,13H
x6-7	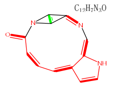 $C_{12}H_7N_3O$	46.3916	257.1949	-26.7209	60.8682	348.1159	InChI=1S/C12H7N3O/c16-9-3-1-2-7-4-5-13-8(7)6-14-10-11-12(10)15(9)11/h1-6,13H/b3-1-,7-2-,8-6+,14-10-
B-202	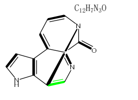 $C_{12}H_7N_3O$	12.5729	54.0028	-3.5848	222.9789	348.1640	InChI=1S/C12H7N3O/c16-12-11-7-2-1-5-15(12)9(6-14-11)10-8(7)3-4-13-10/h1-6,13H
D-19	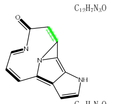 $C_{12}H_7N_3O$	12.9559	129.7493	-9.3496	190.5005	348.4503	InChI=1S/C12H7N3O/c16-10-6-9-12-11-7(3-5-14-11)8(15(9)12)2-1-4-13-10/h1-6,14H/b2-1-,9-6-,13-4+
H11-19	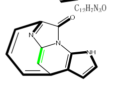 $C_{12}H_7N_3O$	10.435	114.626	-4.0662	189.6572	348.667	InChI=1S/C12H7N3O/c16-12-9-3-1-2-7-6-10(14-9)15(12)11-8(7)4-5-13-11/h1-6,13H/b2-1-,3-1-,7-2+,9-3-
B-86	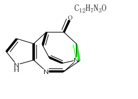 $C_{12}H_7N_3O$	8.5587	74.3810	-4.4111	233.5550	348.9412	InChI=1S/C12H7N3O/c16-11-7-2-1-5-15-9(11)6-10(15)14-12-8(7)3-4-13-12/h1-6,13H/b14-10-
o-203	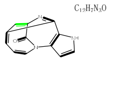 $C_{12}H_7N_3O$	8.8614	100.5732	-3.9438	208.915	348.9871	InChI=1S/C12H7N3O/c16-12-8-6-7-2-1-5-15(12)9-3-4-13-11(9)10(7)14-8/h1-6,13H/b5-1-,7-2-
n-125	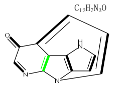 $C_{12}H_7N_3O$	7.1678	104.3791	-0.9996	205.2586	349.2052	InChI=1S/C12H7N3O/c16-9-6-14-12-10-7(9)2-1-5-15(12)8-3-4-13-11(8)10/h1-6,13H
X-33	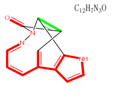 $C_{12}H_7N_3O$	14.5623	171.3439	-4.6292	132.4518	349.5789	InChI=1S/C12H7N3O/c16-12-9-6-7-2-1-4-14-15(12)11(9)10-8(7)3-5-13-10/h1-6,13H/b2-1-,14-4+
H1-54	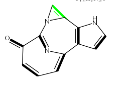 $C_{12}H_7N_3O$	7.1555	231.1202	-3.8667	79.642	349.6081	InChI=1S/C12H7N3O/c16-10-3-1-2-8-7-4-5-13-11(7)9-6-15(9)12(10)14-8/h1-6,13H
i-23	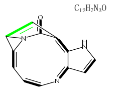 $C_{12}H_7N_3O$	13.1158	198.9243	-6.5594	127.9609	349.9043	InChI=1S/C12H7N3O/c16-12-7-6-10-9(15(10)12)2-1-4-13-8-3-5-14-11(7)8/h1-6,14H/b2-1-,4-1-,9-2-,11-7+,13-4-,13-8-
F-64	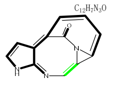 $C_{12}H_7N_3O$	22.864	218.0959	-8.2691	105.2427	350.0516	InChI=1S/C12H7N3O/c16-12-8-2-1-3-9-10(15(9)12)6-14-11-7(8)4-5-13-11/h1-6H,(H,13,14)
A-69	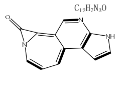 $C_{12}H_7N_3O$	14.8049	280.3973	-5.9719	36.0178	350.0609	InChI=1S/C12H7N3O/c16-12-10-9-6-14-11-8(3-4-13-11)7(9)2-1-5-15(10)12/h1-6,13H
X23-12	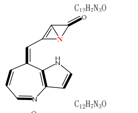 $C_{12}H_7N_3O$	31.7238	322.6931	-16.6811	5.9921	350.127	InChI=1S/C12H7N3O/c16-12-11-9(15(11)12)6-7-2-1-4-13-8-3-5-14-10(7)8/h1-6,14H/b7-6-
p-21	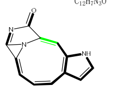 $C_{12}H_7N_3O$	13.1224	137.5348	-7.1533	176.3974	350.1749	InChI=1S/C12H7N3O/c16-12-10-6-8-7(4-5-13-8)2-1-3-9-11(14-12)15(9)10/h1-6,13H/b2-1-,9-3-,10-6-

G1-29	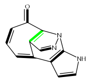	$C_{12}H_7N_3O$	33.9519	146.0801	-1.5755	101.8145	350.2226	InChI=1S/C12H7N3O/c16-10-3-1-2-7-8-4-5-13-12(8)15-11(10)9(7)6-14-15/h1-6,13H
X22-8	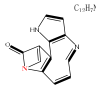	$C_{12}H_7N_3O$	8.3567	195.4239	-5.1717	127.0533	350.3507	InChI=1S/C12H7N3O/c16-12-7-6-15(12)9-2-1-4-13-8-3-5-14-11(8)10(7)9/h1-6,14H/b2-1-,4-1+,9-2+,11-10+,13-4+,13-8-
x3-5	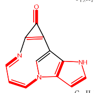	$C_{12}H_7N_3O$	30.6802	211.7078	-16.4982	92.7628	350.6896	InChI=1S/C12H7N3O/c16-12-9-7-6-15(5-1-3-13-11(9)12)8-2-4-14-10(7)8/h1-6,14H/b5-1-,13-3+
q-50	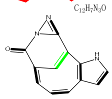	$C_{12}H_7N_3O$	12.8393	207.8316	-3.8111	114.6303	350.9989	InChI=1S/C12H7N3O/c16-12-8-3-1-2-7-4-5-13-10(7)9(6-8)11-14-15(11)12/h1-6,13H/b2-1-,3-1-,7-2-,8-3+,10-9-
B-140	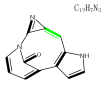	$C_{12}H_7N_3O$	18.9131	195.0910	-9.4755	130.2210	351.2580	InChI=1S/C12H7N3O/c16-12-8-2-1-5-15(12)11-10(14-11)6-9-7(8)3-4-13-9/h1-6,13H/b10-6+
B-186	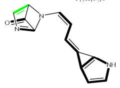	$C_{12}H_7N_3O$	19.9836	197.8071	-12.9876	132.7640	351.2701	InChI=1S/C12H7N3O/c16-11-9-6-14-12(11)15(9)5-1-2-7-8-3-4-13-10(7)8/h1-6,13H/b5-1-,7-2-
s12-4	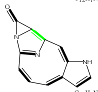	$C_{12}H_7N_3O$	21.5801	245.8718	-7.4915	54.1935	351.3019	InChI=1S/C12H7N3O/c16-12-11-9-6-8-7(4-5-13-8)2-1-3-10(14-9)15(11)12/h1-6,13H/b3-1-,7-2-,8-6+
F-12	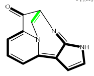	$C_{12}H_7N_3O$	9.9313	93.9073	-3.821	211.1584	351.7119	InChI=1S/C12H7N3O/c16-11-8-6-15-9(2-1-3-10(11)15)7-4-5-13-12(7)14-8/h1-6H,(H,13,14)
X-21	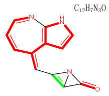	$C_{12}H_7N_3O$	32.0847	326.1695	-16.6754	3.1212	351.7200	InChI=1S/C12H7N3O/c16-12-10-9(15(10)12)6-7-2-1-4-13-11-8(7)3-5-14-11/h1-6,14H/b7-6-
C-71	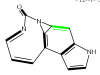	$C_{12}H_7N_3O$	3.2809	301.8370	-2.1021	33.6623	351.8446	InChI=1S/C12H7N3O/c16-12-14-4-1-2-8-7-3-5-13-9(7)6-10-11(8)15(10)12/h1-6,13H/b2-1-,14-4+
x6-8	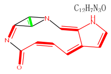	$C_{12}H_7N_3O$	36.2323	245.9439	-24.022	67.4751	352.0725	InChI=1S/C12H7N3O/c16-9-3-1-2-7-4-5-13-8(7)6-15-11-10(14-9)12(11)15/h1-6,13H/b3-1-,7-2-,8-6-,14-10-
o-47	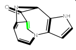	$C_{12}H_7N_3O$	8.0347	32.6852	-2.5691	271.1097	352.0924	InChI=1S/C12H7N3O/c16-12-7-6-15-5-1-2-8(7)14-11(12)10-9(15)3-4-13-10/h1-6,13H
C-72	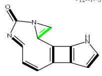	$C_{12}H_7N_3O$	25.4956	293.7610	-6.7510	19.6725	352.1818	InChI=1S/C12H7N3O/c16-12-14-4-1-2-7-8-3-5-13-11(8)10(7)9-6-15(9)12/h1-6,13H/b2-1-,14-4+
H11-57	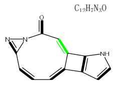	$C_{12}H_7N_3O$	24.7558	242.1763	-6.3139	75.4533	352.2242	InChI=1S/C12H7N3O/c16-11-6-9-7(8-4-5-13-12(8)9)2-1-3-10-14-15(10)11/h1-6,13H/b3-1-,7-2-,9-6+
p1-49	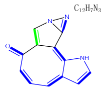	$C_{12}H_7N_3O$	18.3122	157.8525	-4.4159	162.9778	352.2746	InChI=1S/C12H7N3O/c16-9-3-1-2-7-4-5-13-11(7)10-8(9)6-15-12(10)14-15/h1-6,13H/b3-1-,7-2-,11-10-
n-175	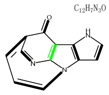	$C_{12}H_7N_3O$	9.249	43.0967	-3.3568	261.357	352.4087	InChI=1S/C12H7N3O/c16-11-7-2-1-5-15-8-3-4-13-10(8)9(11)12(15)14-6-7/h1-6,13H/b5-1-,7-2+
A2-29	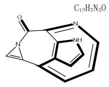	$C_{12}H_7N_3O$	26.3864	177.0612	-17.3042	5.0238352.47:	352.4726	InChI=1S/C12H7N3O/c16-12-11-10-8(3-5-14-10)7(2-1-4-13-11)9-6-15(9)12/h1-6,14H/b2-1-,4-1-,7-2+,13-4-,13-11+
I-83	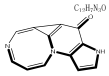	$C_{12}H_7N_3O$	7.1166	211.6778	-5.6091	112.3748	352.5915	InChI=1S/C12H7N3O/c16-12-9-7-6-13-3-1-5-15(11(7)9)8-2-4-14-10(8)12/h1-6,14H/b5-1-,7-6-,13-3-
B-44	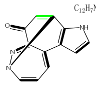	$C_{12}H_7N_3O$	8.4456	32.7371	-3.2322	265.2683	352.6131	InChI=1S/C12H7N3O/c16-10-6-9-11-8(3-4-13-11)7-2-1-5-15(9)14-12(7)10/h1-6,13H

B-179	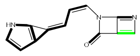 $C_{12}H_7N_3O$	22.1370	224.1673	-13.2651	116.7235	352.7252	InChI=1S/C12H7N3O/c16-12-9-6-14-11(9)15(12)5-1-2-7-8-3-4-13-10(7)8/h1-6,13H/b5-1-,7-2-
A1-6	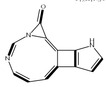 $C_{12}H_7N_3O$	21.8975	284.5503	-8.227	42.8425	352.8862	InChI=1S/C12H7N3O/c16-12-11-9-7(8-3-5-14-10(8)9)2-1-4-13-6-15(11)12/h1-6,14H/b4-1-,7-2-,11-9+,13-6-
H-29	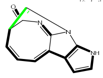 $C_{12}H_7N_3O$	10.9934	90.9764	-3.1155	206.659	352.8959	InChI=1S/C12H7N3O/c16-12-7-2-1-3-8-9-4-5-13-10(9)15(6-7)11(8)14-12/h1-6,13H/b2-1-,8-3-
H11-12	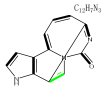 $C_{12}H_7N_3O$	9.2379	95.0732	-3.8468	218.0315	353.2159	InChI=1S/C12H7N3O/c16-12-14-9-2-1-3-10-7-4-5-13-11(7)8(9)6-15(10)12/h1-6,13H/b2-1-,10-3-
F-62	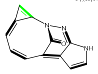 $C_{12}H_7N_3O$	15.0158	242.1577	-8.0848	78.1088	353.3531	InChI=1S/C12H7N3O/c16-12-9-3-1-2-7-6-10(7)15(12)14-11-8(9)4-5-13-11/h1-6H,(H,13,14)/b3-1-,7-2-
o-110	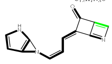 $C_{12}H_7N_3O$	17.0761	177.0623	-12.6082	146.7406	353.422	InChI=1S/C12H7N3O/c16-11-7(10-8(11)6-14-10)2-1-5-15-9-3-4-13-12(9)15/h1-6,13H/b5-1-,7-2+
A1-21	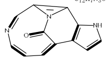 $C_{12}H_7N_3O$	11.6947	228.2749	-3.2068	96.4631	353.427	InChI=1S/C12H7N3O/c16-12-8-2-1-4-13-6-9-11(15(9)12)10-7(8)3-5-14-10/h1-6,14H/b2-1-,4-1-,8-2-,9-6-,13-4-,13-6-
H1-16	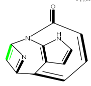 $C_{12}H_7N_3O$	11.0594	177.9608	-4.1784	140.3135	353.4631	InChI=1S/C12H7N3O/c16-11-3-1-2-7-8-4-5-13-12(8)15(11)10-6-9(7)14-10/h1-6,13H/b3-1-,7-2+
A-158	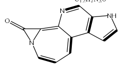 $C_{12}H_7N_3O$	15.3142	283.8904	-6.0637	35.6041	353.5351	InChI=1S/C12H7N3O/c16-12-11-10-8(2-1-5-15(11)12)7-3-4-13-9(7)6-14-10/h1-6,13H
x9-46	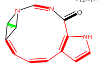 $C_{12}H_7N_3O$	38.0515	229.067	-23.8994	80.7424	353.7788	InChI=1S/C12H7N3O/c16-12-9-7(4-5-13-9)2-1-3-8-10-11(8)15(10)6-14-12/h1-6,13H/b2-1-,8-3-,14-6-
H11-90	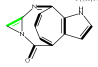 $C_{12}H_7N_3O$	24.3526	163.9546	-16.628	153.2787	354.178	InChI=1S/C12H7N3O/c16-12-8-2-1-3-9(14-10-6-15(10)12)11-7(8)4-5-13-11/h1-6,13H/b14-9+
H-35	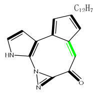 $C_{12}H_7N_3O$	15.9689	227.0712	-3.4489	94.6273	354.3727	InChI=1S/C12H7N3O/c16-10-6-7-2-1-3-8(7)9-4-5-13-11(9)15-12(10)14-15/h1-6,13H/b7-6-
q-46	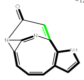 $C_{12}H_7N_3O$	9.9801	227.5151	-3.9076	110.2736	354.6103	InChI=1S/C12H7N3O/c16-10-6-8-11-7(4-5-13-11)2-1-3-9-12(14-8)15(9)10/h1-6,13H/b2-1-,9-3+
A-170	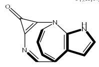 $C_{12}H_7N_3O$	27.9465	232.8078	-10.1632	86.4161	355.1117	InChI=1S/C12H7N3O/c16-11-9-10(11)15-5-1-2-7(6-14-9)8-3-4-13-12(8)15/h1-6,13H/b14-6-
q1-19	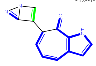 $C_{12}H_7N_3O$	19.7557	209.9681	-13.8179	116.8716	355.342	InChI=1S/C12H7N3O/c16-11-8(9-6-15-12(9)14-15)3-1-2-7-4-5-13-10(7)11/h1-6,13H
q1-23	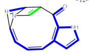 $C_{12}H_7N_3O$	7.2804	157.7975	-2.4385	168.4003	355.417	InChI=1S/C12H7N3O/c16-11-8-6-15-9(14-12(8)15)3-1-2-7-4-5-13-10(7)11/h1-6,13H/b2-1-,9-3-
s11-51	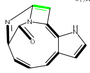 $C_{12}H_7N_3O$	13.2577	205.9148	-3.0692	125.4356	355.7266	InChI=1S/C12H7N3O/c16-12-8-3-1-2-7-4-5-13-11(7)9-6-10(14-8)15(9)12/h1-6,13H/b2-1-,3-1-,7-2-,8-3+,11-9+
A2-69	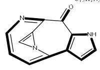 $C_{12}H_7N_3O$	24.0255	227.9868	-12.5083	74.7545	355.8438	InChI=1S/C12H7N3O/c16-12-10-7(3-5-14-10)8-2-1-4-13-11(12)9-6-15(8)9/h1-6,14H/b4-1-,8-2-,13-11+
B-91	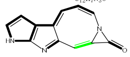 $C_{12}H_7N_3O$	8.5383	282.3750	-7.4345	68.4081	355.9379	InChI=1S/C12H7N3O/c16-12-10-6-9-7(2-1-5-15(10)12)8-3-4-13-11(8)14-9/h1-6,13H/b5-1-,7-2-,10-6-

G1-99	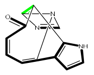	10.9977	174.2464	-1.8165	151.5534	356.0999	InChI=1S/C12H7N3O/c16-9-3-1-2-8-7-4-5-13-11(7)10(9)12-14-6-15(8)12/h1-6,13H/b3-1-,8-2-
o-251	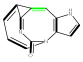	26.0913	203.7383	-9.3129	93.8813	356.12	InChI=1S/C12H7N3O/c16-12-14-11-7-2-1-5-15(12)10-3-4-13-9(10)6-8(7)11/h1-6,13H/b5-1-,7-2-,8-6-,14-11-
B-46	C12H7N3O 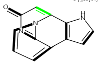	17.6995	89.6610	-3.6359	210.5486	356.2227	InChI=1S/C12H7N3O/c16-10-6-9-11-7(3-4-13-11)8-2-1-5-15(9)12(8)14-10/h1-6,13H
E-67	C12H7N3O 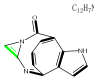	25.1885	166.5068	-16.5885	148.1195	356.429	InChI=1S/C12H7N3O/c16-12-8-2-1-3-9(14-10-6-15(10)12)7-4-5-13-11(7)8/h1-6,13H/b14-9+
q1-57	C12H7N3O 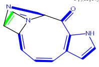	8.4494	180.4311	-5.574	143.4827	356.4295	InChI=1S/C12H7N3O/c16-11-10-7(4-5-13-10)2-1-3-9-8-6-15(9)12(11)14-8/h1-6,13H/b2-1-,9-3-
E-27	C12H7N3O 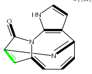	5.9458	96.7895	-2.2615	223.6454	356.468	InChI=1S/C12H7N3O/c16-12-10-6-7-2-1-3-9(14-10)8-4-5-13-11(8)15(7)12/h1-6,13H/b2-1-,3-1-,7-2-,9-3-
o-170	C12H7N3O 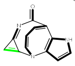	18.6294	173.4723	-13.674	139.3953	356.5295	InChI=1S/C12H7N3O/c16-12-7-2-1-5-15(10-6-8(10)14-12)9-3-4-13-11(7)9/h1-6,13H/b14-8-
G1-63	C12H7N3O 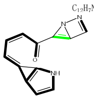	29.1437	289.4842	-18.4399	40.5821	357.019	InChI=1S/C12H7N3O/c16-10(12-9-6-14-15(9)12)3-1-2-7-8-4-5-13-11(7)8/h1-6,13H/b3-1-,7-2-
B-235	C12H7N3O 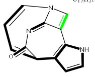	8.6235	70.3362	-3.1036	241.9506	357.1469	InChI=1S/C12H7N3O/c16-12-8-2-1-5-15-6-9(11(15)14-12)10-7(8)3-4-13-10/h1-6,13H/b5-1-,8-2-
C-46	C12H7N3O 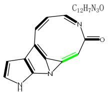	18.9583	193.5453	-6.9511	134.2526	357.1578	InChI=1S/C12H7N3O/c16-10-6-9-11-7(2-1-4-13-10)8-3-5-14-12(8)15(9)11/h1-6,14H/b2-1-,9-6+,13-4-
o-113	C12H7N3O 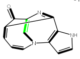	5.1407	38.9076	-1.4712	278.4769	357.1695	InChI=1S/C12H7N3O/c16-12-7-2-1-5-15-6-8(12)14-10(7)11-9(15)3-4-13-11/h1-6,13H/b5-1-,7-2+
C-14	C12H7N3O 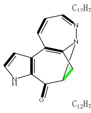	12.8227	146.6011	-2.9815	165.6015	357.1712	InChI=1S/C12H7N3O/c16-12-10-6-9-7(2-1-4-14-15(9)10)8-3-5-13-11(8)12/h1-6,13H
D-3	C12H7N3O 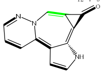	17.7607	201.6942	-8.8305	124.2492	357.3225	InChI=1S/C12H7N3O/c16-12-8-6-15-9(2-1-4-14-15)7-3-5-13-11(7)10(8)12/h1-6,13H
p11-18	C12H7N3O 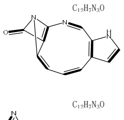	35.7288	217.797	-18.888	91.1	357.3365	InChI=1S/C12H7N3O/c16-12-10-9-3-1-2-7-4-5-13-8(7)6-14-11(10)15(9)12/h1-6,13H/b2-1-,3-1-,7-2-,8-6+,9-3+,14-6-,14-11+
G-16	C12H7N3O 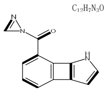	30.3066	317.8375	-11.7133	13.8911	357.337	InChI=1S/C12H7N3O/c16-12(15-6-14-15)9-3-1-2-7-8-4-5-13-11(8)10(7)9/h1-6,13H
X-3	C12H7N3O 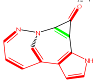	23.7712	254.4892	-7.4976	66.1850	357.3994	InChI=1S/C12H7N3O/c16-12-9-10-8(3-5-13-10)7-2-1-4-14-15(6-7)11(9)12/h1-6,13H
H1-81	C12H7N3O 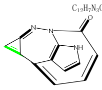	18.066	235.2784	-14.2733	87.0236	357.4388	InChI=1S/C12H7N3O/c16-11-3-1-2-7-8-4-5-13-12(8)15(11)14-10-6-9(7)10/h1-6,13H/b3-1-,7-2+
B-253	C12H7N3O 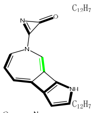	20.6503	286.7302	-7.6626	31.9001	357.4569	InChI=1S/C12H7N3O/c16-12-11(14-12)15-5-1-2-7-8-3-4-13-10(8)9(7)6-15/h1-6,13H
H-116	C12H7N3O 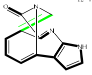	7.098	82.984	-2.6601	233.2515	357.6086	InChI=1S/C12H7N3O/c16-12-10-7-2-1-3-9(15(12)6-7)8-4-5-13-11(8)14-10/h1-6,13H

C12H7N3O

q1-40	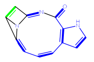 $C_{12}H_7N_3O$	19. 3674	129. 8165	-11. 7222	190. 3512	357. 6256	InChI=1S/C12H7N3O/c16-12-11-7(4-5-13-11)2-1-3-8-9-6-10(14-12)15(8)9/h1-6,13H/b2-1-,8-3-,14-10-
F-7	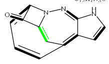 $C_{12}H_7N_3O$	11. 7877	145. 3081	-3. 3714	169. 1319	357. 6466	InChI=1S/C12H7N3O/c16-11-9-3-1-2-7-6-10(11)15(9)14-12-8(7)4-5-13-12/h1-6H,(H,13,14)/b2-1-,9-3+
B-152	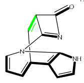 $C_{12}H_7N_3O$	6. 6045	138. 8015	-3. 0580	178. 6818	357. 6659	InChI=1S/C12H7N3O/c16-12-9-6-7-2-1-5-15(11(9)14-12)10-8(7)3-4-13-10/h1-6,13H
B-59	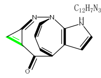 $C_{12}H_7N_3O$	18. 5091	148. 5981	-14. 3403	166. 2510	357. 7080	InChI=1S/C12H7N3O/c16-11-7-2-1-5-15(14-10-6-9(10)11)12-8(7)3-4-13-12/h1-6,13H/b14-10-
x3-12	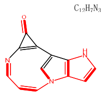 $C_{12}H_7N_3O$	27. 8299	243. 9356	-10. 6665	75. 718	357. 7978	InChI=1S/C12H7N3O/c16-12-9-7-6-15(5-1-3-13-11(9)12)8-2-4-14-10(7)8/h1-6,14H/b5-1-,13-3-
x4-15	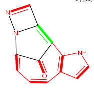 $C_{12}H_7N_3O$	6. 3976	182. 3631	-2. 4661	138. 787	358. 0612	InChI=1S/C12H7N3O/c16-12-8-3-1-2-7-4-5-13-11(7)10(12)9-6-14-15(8)9/h1-6,13H/b2-1-,8-3+
G-81	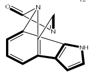 $C_{12}H_7N_3O$	7. 428	171. 7295	-2. 4283	153. 372	358. 076	InChI=1S/C12H7N3O/c16-12-14-6-10-7-2-1-3-9(15(10)12)11-8(7)4-5-13-11/h1-6,13H
E-72	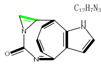 $C_{12}H_7N_3O$	19. 5873	214. 2455	-12. 3519	107. 7775	358. 4599	InChI=1S/C12H7N3O/c16-12-14-9-3-1-2-8(10-6-15(10)12)11-7(9)4-5-13-11/h1-6,13H/b14-9+
H-16	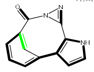 $C_{12}H_7N_3O$	26. 2718	253. 645	-0. 7124	65. 0697	358. 4805	InChI=1S/C12H7N3O/c16-12-8-3-1-2-7(6-8)9-4-5-13-10(9)11-14-15(11)12/h1-6,13H
q1-55	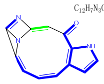 $C_{12}H_7N_3O$	17. 8728	131. 0019	-11. 355	197. 2071	358. 9308	InChI=1S/C12H7N3O/c16-9-6-10-14-12-8(15(10)12)3-1-2-7-4-5-13-11(7)9/h1-6,13H/b2-1-,8-3-,10-6-
H11-16	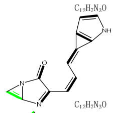 $C_{12}H_7N_3O$	36. 2382	296. 197	-22. 6558	40. 0081	359. 0486	InChI=1S/C12H7N3O/c16-12-9(14-10-6-15(10)12)3-1-2-7-8-4-5-13-11(7)8/h1-6,13H/b3-1-,7-2-
H-82	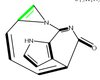 $C_{12}H_7N_3O$	13. 7007	213. 0682	-5. 7623	101. 7744	359. 1671	InChI=1S/C12H7N3O/c16-12-9-3-1-2-7-6-15(7)11(14-12)10-8(9)4-5-13-10/h1-6,13H/b2-1-,9-3+
C-22	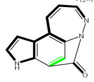 $C_{12}H_7N_3O$	7. 1790	129. 7094	-3. 3371	185. 8726	359. 3948	InChI=1S/C12H7N3O/c16-12-9-6-10-7(2-1-4-14-15(10)12)8-3-5-13-11(8)9/h1-6,13H
G1-114	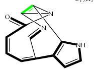 $C_{12}H_7N_3O$	16. 7974	216. 327	-6. 4338	107. 2498	359. 5419	InChI=1S/C12H7N3O/c16-9-3-1-2-7-6-14-12-11(15(9)12)10-8(7)4-5-13-10/h1-6,13H/b3-1-,7-2+
H11-65	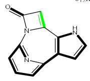 $C_{12}H_7N_3O$	13. 0264	194. 3278	-4. 4929	125. 5378	359. 6479	InChI=1S/C12H7N3O/c16-11-6-9-12-7(4-5-13-12)8-2-1-3-10(14-8)15(9)11/h1-6,13H
s11-34	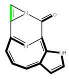 $C_{12}H_7N_3O$	19. 1708	201. 7029	-9. 1767	119. 7062	359. 8846	InChI=1S/C12H7N3O/c16-12-11-10-7(4-5-13-10)2-1-3-8(14-11)9-6-15(9)12/h1-6,13H/b2-1-,3-1-,7-2-,8-3-,11-10+
B-196	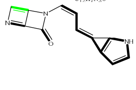 $C_{12}H_7N_3O$	22. 3495	224. 8795	-13. 4467	121. 4834	359. 9655	InChI=1S/C12H7N3O/c16-12-11-9(6-14-11)15(12)5-1-2-7-8-3-4-13-10(7)8/h1-6,13H/b5-1-,7-2-
q-38	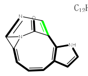 $C_{12}H_7N_3O$	7. 9099	203. 0362	-5. 1618	135. 7849	360. 1542	InChI=1S/C12H7N3O/c16-12-8-6-14-11-9(15(11)12)3-1-2-7-4-5-13-10(7)8/h1-6,13H/b2-1-,3-1-,7-2?,9-3-,10-8+
o-160	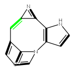 $C_{12}H_7N_3O$	18. 3373	192. 6342	-4. 6111	130. 7229	360. 1678	InChI=1S/C12H7N3O/c16-12-7-2-1-5-15(12)9-3-4-13-11(9)10-8(6-7)14-10/h1-6,13H/b8-6+

o-200	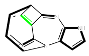 $C_{12}H_7N_3O$	37.3962	93.5428	-3.6922	142.5601	360.2204	InChI=1S/C12H7N3O/c16-12-8-6-7-2-1-5-15(12)9-3-4-13-11(9)14-10(7)8/h1-6,13H/b5-1-,7-2+
X-41	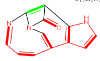 $C_{12}H_7N_3O$	12.4474	145.9632	-4.1576	177.3891	360.3239	InChI=1S/C12H7N3O/c16-12-9-10-8(3-5-13-10)7-2-1-4-14-11(9)15(12)6-7/h1-6,13H/b2-1-,14-4-
A2-35	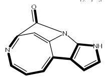 $C_{12}H_7N_3O$	10.4039	216.2386	-0.6621	98.691	360.75	InChI=1S/C12H7N3O/c16-12-9-6-10-7(2-1-4-13-9)8-3-5-14-11(8)15(10)12/h1-6,14H/b2-1-,4-1-,7-2-,13-4-,13-9+
H11-48	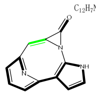 $C_{12}H_7N_3O$	6.0277	334.9954	-1.0624	-2.2999	360.8441	InChI=1S/C12H7N3O/c16-12-10-6-7-2-1-3-9(14-7)8-4-5-13-11(8)15(10)12/h1-6,13H/b10-6-
x4-1	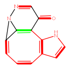 $C_{12}H_7N_3O$	9.3239	228.0712	-4.9477	107.2163	361.3278	InChI=1S/C12H7N3O/c16-9-6-14-15-8-3-1-2-7-4-5-13-11(7)10(9)12(8)15/h1-6,13H/b2-1-,3-1-,7-2-,8-3+,11-10+
F-14	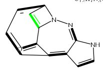 $C_{12}H_7N_3O$	13.9031	150.8384	-3.7384	166.2317	361.4487	InChI=1S/C12H7N3O/c16-11-8-3-1-2-7-6-10(11)15(7)14-12-9(8)4-5-13-12/h1-6H,(H,13,14)/b3-1-,7-2+
j-20	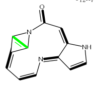 $C_{12}H_7N_3O$	52.0822	248.7604	-28.1838	78.4327	361.4616	InChI=1S/C12H7N3O/c16-10-6-9-8(3-5-14-9)13-4-1-2-7-11-12(7)15(10)11/h1-6,14H/b4-1-,7-2-,9-6+,13-8+
i-14	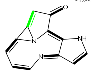 $C_{12}H_7N_3O$	16.2538	159.1262	-7.5876	160.0194	361.5319	InChI=1S/C12H7N3O/c16-10-6-9-8-2-1-4-13-7-3-5-14-11(7)12(10)15(8)9/h1-6,14H/b4-1-,8-2-,12-11+,13-7+
o-76	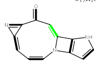 $C_{12}H_7N_3O$	16.3222	223.0386	-5.4969	90.0877	361.5466	InChI=1S/C12H7N3O/c16-10-6-9-12-8(3-4-13-12)15(9)5-1-2-7-11(10)14-7/h1-6,13H/b5-1-,7-2+,9-6-
x4-17	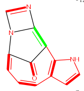 $C_{12}H_7N_3O$	5.7543	181.7657	-2.2622	147.1579	361.5525	InChI=1S/C12H7N3O/c16-11-8-3-1-2-7-4-5-13-10(7)9(11)12-14-6-15(8)12/h1-6,13H/b2-1-,8-3+
B-89	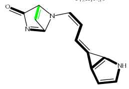 $C_{12}H_7N_3O$	19.8214	193.0310	-13.0852	149.5271	361.6426	InChI=1S/C12H7N3O/c16-12-9-6-10(14-12)15(9)5-1-2-7-8-3-4-13-11(7)8/h1-6,13H/b5-1-,7-2-
H11-35	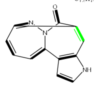 $C_{12}H_7N_3O$	8.4306	118.5863	-3.6794	200.3334	362.1659	InChI=1S/C12H7N3O/c16-12-8-6-10-7(4-5-13-10)11-3-1-2-9(8)14-15(11)12/h1-6,13H
n-95	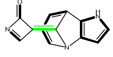 $C_{12}H_7N_3O$	7.908	171.4089	-3.2122	154.4457	362.2157	InChI=1S/C12H7N3O/c16-12-8(6-14-12)11-7-2-1-5-15(11)9-3-4-13-10(7)9/h1-6,13H/b11-8+
q11-52	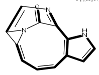 $C_{12}H_7N_3O$	7.9409	197.7129	-5.0617	140.5474	362.5136	InChI=1S/C12H7N3O/c16-12-11-10-7(4-5-13-10)2-1-3-8-9(6-14-11)15(8)12/h1-6,13H/b2-1-,3-1-,7-2?,8-3-,11-10-
q-12	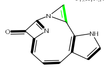 $C_{12}H_7N_3O$	21.827	257.3791	-12.4492	52.9452	362.5401	InChI=1S/C12H7N3O/c16-11-8-3-1-2-7-4-5-13-10(7)9-6-15(9)12(11)14-8/h1-6,13H/b2-1-,8-3-
p1-45	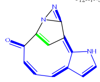 $C_{12}H_7N_3O$	20.6389	168.0479	-3.6582	149.8415	362.6076	InChI=1S/C12H7N3O/c16-10-3-1-2-7-4-5-13-11(7)8-6-9(10)15-12(8)14-15/h1-6,13H/b3-1-,7-2-,11-8-
o-241	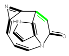 $C_{12}H_7N_3O$	25.6755	187.3657	-4.841	140.9863	363.208	InChI=1S/C12H7N3O/c16-10-6-7-11-8(14-11)2-1-5-15(10)9-3-4-13-12(7)9/h1-6,13H/b5-1-,8-2+
B-82	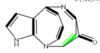 $C_{12}H_7N_3O$	9.4375	65.5313	-5.1913	248.0615	364.3133	InChI=1S/C12H7N3O/c16-11-9-6-10(11)15-5-1-2-8(14-9)7-3-4-13-12(7)15/h1-6,13H/b14-9+
l-35	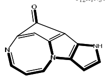 $C_{12}H_7N_3O$	11.7233	214.5309	1.3352	93.9193	364.3158	InChI=1S/C12H7N3O/c16-12-7-6-9-10(12)11-8(2-4-14-11)15(9)5-1-3-13-7/h1-6,14H/b5-1-,13-3-

q-36	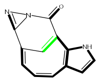 $C_{12}H_7N_3O$	15.4264	217.9696	-7.0823	118.0978	364.7496	InChI=1S/C12H7N3O/c16-12-9-6-8(11-14-15(11)12)3-1-2-7-4-5-13-10(7)9/h1-6,13H/b2-1-,3-1-,7-2?,8-3+,10-9-
H11-44	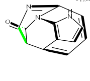 $C_{12}H_7N_3O$	9.568	108.0083	-3.784	209.3507	364.9065	InChI=1S/C12H7N3O/c16-12-9-6-14-10-3-1-2-7(9)8-4-5-13-11(8)15(10)12/h1-6,13H/b2-1-,3-1-,7-2+,10-3-
q11-41	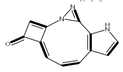 $C_{12}H_7N_3O$	16.049	216.3937	-5.6033	106.6339	365.0018	InChI=1S/C12H7N3O/c16-10-6-9-8(10)3-1-2-7-4-5-13-11(7)12-14-15(9)12/h1-6,13H/b2-1-,8-3+
q11-23	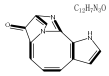 $C_{12}H_7N_3O$	7.6519	115.3346	-2.871	203.023	365.0165	InChI=1S/C12H7N3O/c16-11-8-6-15-9(11)3-1-2-7-4-5-13-10(7)12(15)14-8/h1-6,13H/b2-1-,9-3-
B-34	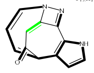 $C_{12}H_7N_3O$	12.0145	78.8127	-3.8106	234.7978	365.2996	InChI=1S/C12H7N3O/c16-10-6-9-12-11-8(3-4-13-11)7(10)2-1-5-15(9)14-12/h1-6,13H/b5-1-,7-2-
G-75	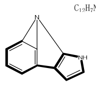 $C_{10}H_6N_2$	16.3341	217.3653	-6.8775	122.052	365.3625	InChI=1S/C10H6N2/c1-2-6-7-4-5-11-10(7)12-8(3-1)9(6)12/h1-5,11H
X22-29	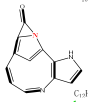 $C_{12}H_7N_3O$	19.6855	241.6703	-10.4099	103.9702	365.901	InChI=1S/C12H7N3O/c16-12-11-7-2-1-4-13-8-3-5-14-10(8)9(6-7)15(11)12/h1-6,14H/b2-1-,13-4-
H1-31	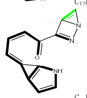 $C_{12}H_7N_3O$	33.0881	294.2946	-22.2101	45.0104	366.1205	InChI=1S/C12H7N3O/c16-10(12-9-6-15(9)14-12)3-1-2-7-8-4-5-13-11(7)8/h1-6,13H/b3-1-,7-2-
s12-20	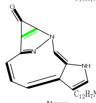 $C_{12}H_7N_3O$	31.8228	256.098	-14.339	66.1397	366.1429	InChI=1S/C12H7N3O/c16-12-10-8-3-1-2-7-4-5-13-9(7)6-15(14-8)11(10)12/h1-6,13H/b3-1-,7-2-,9-6-
G-67	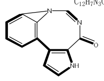 $C_{12}H_7N_3O$	9.6824	200.1228	-4.4344	140.1312	366.4059	InChI=1S/C12H7N3O/c16-12-10-7(4-5-13-10)8-2-1-3-9-11(8)15(9)6-14-12/h1-6,13H/b14-6-
x4-42	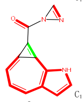 $C_{12}H_7N_3O$	35.1516	276.8718	-18.756	57.1519	366.4869	InChI=1S/C12H7N3O/c16-12(15-6-14-15)10-8-3-1-2-7-4-5-13-11(7)9(8)10/h1-6,13H
A-166	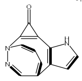 $C_{12}H_7N_3O$	27.4867	238.6362	-9.4751	89.6954	366.692	InChI=1S/C12H7N3O/c16-12-9-10-8(3-4-13-10)7-2-1-5-15(11(9)12)14-6-7/h1-6,13H
H1-106	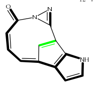 $C_{12}H_7N_3O$	37.9115	238.0814	-3.5103	80.7127	366.8203	InChI=1S/C12H7N3O/c16-10-3-1-2-7-6-9(12-14-15(10)12)11-8(7)4-5-13-11/h1-6,13H/b3-1-,7-2+
G1-32	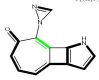 $C_{12}H_7N_3O$	3.6964	368.8892	-1.9363	-10.49	366.9746	InChI=1S/C12H7N3O/c16-9-3-1-2-7-8-4-5-13-11(8)10(7)12(9)15-6-14-15/h1-6,13H
G1-111	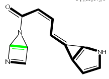 $C_{12}H_7N_3O$	39.1638	301.6379	-20.1433	38.0727	367.0445	InChI=1S/C12H7N3O/c16-10(15-9-6-14-12(9)15)3-1-2-7-8-4-5-13-11(7)8/h1-6,13H/b3-1-,7-2-
o-184	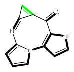 $C_{12}H_7N_3O$	21.9403	241.2134	-13.9223	77.8546	367.0457	InChI=1S/C12H7N3O/c16-12-7-6-8(7)14-10-2-1-5-15(10)9-3-4-13-11(9)12/h1-6,13H/b14-8-
I-96	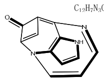 $C_{12}H_7N_3O$	8.7106	78.2311	-3.2675	234.5267	367.0629	InChI=1S/C12H7N3O/c16-12-7-6-9(12)15-5-1-3-13-10(7)11-8(15)2-4-14-11/h1-6,14H/b5-1-,13-3-
G-25	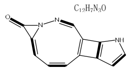 $C_{12}H_7N_3O$	8.3426	343.5923	-5.2559	14.4754	367.2447	InChI=1S/C12H7N3O/c16-12-10-3-1-2-7-8-4-5-13-11(8)9(7)6-14-15(10)12/h1-6,13H/b2-1-,10-3-,14-6-
H-119	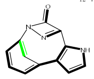 $C_{12}H_7N_3O$	8.9371	166.9977	-5.4604	161.7183	367.5905	InChI=1S/C12H7N3O/c16-12-11-10-9(4-5-13-10)7-2-1-3-8(6-7)15(12)14-11/h1-6,13H

o-90	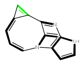	$C_{12}H_7N_3O$	24.7623	261.9209	-13.7079	51.5598	368.1457	InChI=1S/C12H7N3O/c16-12-10-8-6-7(8)2-1-5-15(12)9-3-4-13-11(9)14-10/h1-6,13H/b5-1-,7-2-
n-155	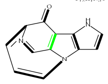	$C_{12}H_7N_3O$	9.6764	42.55	-3.2076	276.531	368.3403	InChI=1S/C12H7N3O/c16-12-7-2-1-5-15-8-3-4-13-11(8)10(12)9(15)6-14-7/h1-6,13H/b5-1-,7-2-
D-1	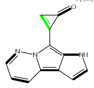	$C_{12}H_7N_3O$	11.8480	359.8022	-3.5368	-4.8238	368.3830	InChI=1S/C12H7N3O/c16-10-6-8(10)12-11-7(3-5-13-11)9-2-1-4-14-15(9)12/h1-6,13H
E-57	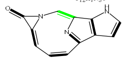	$C_{12}H_7N_3O$	6.7918	306.9802	-3.6346	44.7111	368.5699	InChI=1S/C12H7N3O/c16-12-10-3-1-2-8-7-4-5-13-11(7)9(14-8)6-15(10)12/h1-6,13H/b2-1-,9-6-,10-3-
I-88	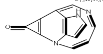	$C_{12}H_7N_3O$	33.2745	233.3379	-12.0615	90.5583	368.5794	InChI=1S/C12H7N3O/c16-12-9-7-6-13-3-1-5-15(11(9)12)8-2-4-14-10(7)8/h1-6,14H/b5-1-,7-6-,13-3-
o-53	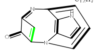	$C_{12}H_7N_3O$	6.8147	76.7257	-2.7153	242.2632	368.6395	InChI=1S/C12H7N3O/c16-12-8-6-10(12)15-5-1-2-7(14-8)11-9(15)3-4-13-11/h1-6,13H/b14-8+
H11-127	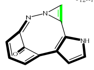	$C_{12}H_7N_3O$	14.1733	239.5553	-8.3514	82.1033	368.7102	InChI=1S/C12H7N3O/c16-12-8-2-1-3-9(12)14-15-6-10(15)11-7(8)4-5-13-11/h1-6,13H/b14-9-
q-52	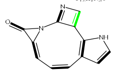	$C_{12}H_7N_3O$	2.4677	378.3159	-2.2351	-7.1	368.9684	InChI=1S/C12H7N3O/c16-12-9-3-1-2-7-4-5-13-10(7)8-6-14-11(8)15(9)12/h1-6,13H/b2-1-,9-3-
B-53	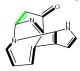	$C_{12}H_7N_3O$	6.6415	98.0687	-2.7796	231.2525	369.3214	InChI=1S/C12H7N3O/c16-9-6-10-14-11(9)7-2-1-5-15(10)12-8(7)3-4-13-12/h1-6,13H
o-26	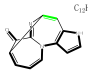	$C_{12}H_7N_3O$	29.9898	82.4332	-0.903	182.6085	369.5068	InChI=1S/C12H7N3O/c16-11-7-2-1-5-15-10-3-4-13-9(10)6-8(7)14-12(11)15/h1-6,13H
H-133	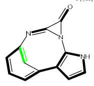	$C_{12}H_7N_3O$	2.3951	305.9491	-1.4391	48.3545	369.5692	InChI=1S/C12H7N3O/c16-12-11-14-8-3-1-2-7(6-8)9-4-5-13-10(9)15(11)12/h1-6,13H/b14-11-
H-22	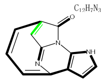	$C_{12}H_7N_3O$	10.7236	110.7754	-5.053	214.8156	369.728	InChI=1S/C12H7N3O/c16-12-7-2-1-3-9-8-4-5-13-11(8)15(12)10(6-7)14-9/h1-6,13H/b2-1-,3-1-,7-2+,9-3-
o-49	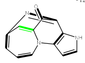	$C_{12}H_7N_3O$	6.0466	34.8679	-2.2957	285.5235	370.1044	InChI=1S/C12H7N3O/c16-12-9-6-7-2-1-5-15(9)8-3-4-13-10(8)11(12)14-7/h1-6,13H
B-11	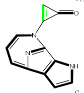	$C_{12}H_7N_3O$	41.2144	197.1890	-17.7926	125.0926	370.1200	InChI=1S/C12H7N3O/c16-10-6-9(10)15-5-1-2-8-7-3-4-13-11(7)12(15)14-8/h1-6,13H
E-54	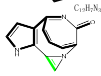	$C_{12}H_7N_3O$	13.4086	207.011	-4.9089	117.0614	370.3933	InChI=1S/C12H7N3O/c16-12-9-3-1-2-8(14-12)7-4-5-13-11(7)10-6-15(9)10/h1-6,13H
X22-24	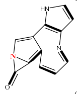	$C_{12}H_7N_3O$	18.059	222.3667	-10.5004	131.4791	370.4035	InChI=1S/C12H7N3O/c16-12-11-7-2-1-4-13-9-3-5-14-10(9)8(7)6-15(11)12/h1-6,14H/b2-1-,13-4+
s11-48	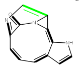	$C_{12}H_7N_3O$	13.7836	214.2104	-5.8742	134.0995	370.6094	InChI=1S/C12H7N3O/c16-12-8-3-1-2-7-4-5-13-10(7)11-9(6-14-8)15(11)12/h1-6,13H/b2-1-,3-1-,7-2-,8-3+,11-10+
n-53	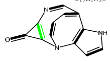	$C_{12}H_7N_3O$	28.0244	235.953	-10.2332	95.4831	370.8986	InChI=1S/C12H7N3O/c16-12-10-11(12)15-5-1-2-7(6-14-10)9-8(15)3-4-13-9/h1-6,13H/b14-6-
H11-136	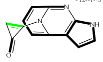	$C_{12}H_7N_3O$	40.3957	182.6014	-18.6435	141.8413	370.9652	InChI=1S/C12H7N3O/c16-10-6-9(10)15-8-2-1-3-11(15)14-12-7(8)4-5-13-12/h1-6H,(H,13,14)

X22-9	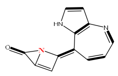 $C_{12}H_7N_3O$	14.7307	300.1629	-9.418	61.0408	370.9844	InChI=1S/C12H7N3O/c16-12-10-6-9(15(10)12)7-2-1-4-13-8-3-5-14-11(7)8/h1-6,14H/b9-7-
p-15	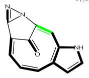 $C_{12}H_7N_3O$	21.1393	165.9135	-3.5988	160.4256	371.0786	InChI=1S/C12H7N3O/c16-11-8-3-1-2-7-4-5-13-9(7)6-10(11)15-12(8)14-15/h1-6,13H/b2-1-,8-3+,10-6+
A1-29	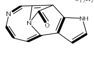 $C_{12}H_7N_3O$	28.7316	203.1437	-6.0521	107.4882	371.1511	InChI=1S/C12H7N3O/c16-12-10-9-6-13-4-1-2-8(15(9)12)7-3-5-14-11(7)10/h1-6,14H/b2-1-,4-1-,8-2-,9-6-,13-4-,13-6-
H-39	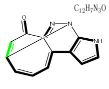 $C_{12}H_7N_3O$	9.2966	97.8013	-2.0925	213.9225	371.3012	InChI=1S/C12H7N3O/c16-10-6-7-2-1-3-8-9-4-5-13-12(9)15(7)14-11(8)10/h1-6,13H/b2-1-,8-3-
n-161	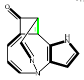 $C_{12}H_7N_3O$	14.4154	123.8091	-3.327	191.502	371.5831	InChI=1S/C12H7N3O/c16-12-7-2-1-5-15-9-3-4-13-11(9)10(7)8(12)6-14-15/h1-6,13H/b5-1-,7-2+
B-69	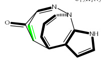 $C_{12}H_7N_3O$	11.4242	125.3384	-3.9610	190.8051	371.6340	InChI=1S/C12H7N3O/c16-11-9-6-10(11)14-15-5-1-2-7(9)8-3-4-13-12(8)15/h1-6,13H/b14-10+
H1-29	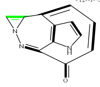 $C_{12}H_7N_3O$	17.5339	185.5619	-12.4778	128.6816	371.6371	InChI=1S/C12H7N3O/c16-10-3-1-2-7-8-4-5-13-11(8)12(10)14-15-6-9(7)15/h1-6,13H/b3-1-,7-2+
H1-48	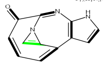 $C_{12}H_7N_3O$	32.5244	236.9127	-18.4822	78.5267	371.6996	InChI=1S/C12H7N3O/c16-10-3-1-2-7-8-4-5-13-11(8)14-12(10)15-6-9(7)15/h1-6,13H/b3-1-,7-2-
E-8	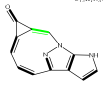 $C_{12}H_7N_3O$	4.1203	349.9685	-0.7266	-2.38	371.8685	InChI=1S/C12H7N3O/c16-11-7-2-1-3-10-8-4-5-13-12(8)15(14-10)6-9(7)11/h1-6,13H/b3-1-,7-2+,9-6+
p1-47	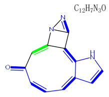 $C_{12}H_7N_3O$	24.5875	147.461	-10.1	193.7638	371.9059	InChI=1S/C12H7N3O/c16-8-3-1-2-7-4-5-13-11(7)10-9(6-8)15-12(10)14-15/h1-6,13H/b3-1-,7-2-,9-6+,11-10-
q1-17	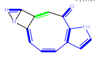 $C_{12}H_7N_3O$	24.0602	145.3116	-9.9427	196.7868	371.9232	InChI=1S/C12H7N3O/c16-10-6-8-9(15-12(8)14-15)3-1-2-7-4-5-13-11(7)10/h1-6,13H/b2-1-,8-6+,9-3+
A1-40	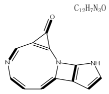 $C_{12}H_7N_3O$	32.0292	260.58	-14.8056	70.0872	372.1395	InChI=1S/C12H7N3O/c16-11-8-6-13-4-1-2-9-7-3-5-14-12(7)15(9)10(8)11/h1-6,14H/b4-1-,9-2-,13-6-
G1-100	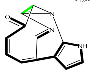 $C_{12}H_7N_3O$	16.6557	272.0284	-5.5065	41.4828	372.2318	InChI=1S/C12H7N3O/c16-9-3-1-2-7-6-14-12-10(9)15(12)11-8(7)4-5-13-11/h1-6,13H/b3-1-,7-2+
x5-31	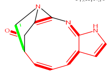 $C_{12}H_7N_3O$	12.8003	277.0201	-6.1549	54.5863	372.63	InChI=1S/C12H7N3O/c16-10-8-3-1-2-7-4-5-13-11(7)14-12-9(10)15(12)6-8/h1-6H,(H,13,14)/b3-1-,7-2-
p1-33	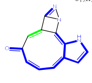 $C_{12}H_7N_3O$	16.0141	214.9551	-8.2407	128.1811	372.7541	InChI=1S/C12H7N3O/c16-8-3-1-2-7-4-5-13-10(7)11-9(6-8)12-14-15(11)12/h1-6,13H/b3-1-,7-2-,9-6+,11-10-
H11-25	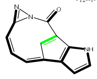 $C_{12}H_7N_3O$	4.1354	331.8086	-1.6928	28.7373	372.7771	InChI=1S/C12H7N3O/c16-12-9-6-7(8-4-5-13-11(8)9)2-1-3-10-14-15(10)12/h1-6,13H/b3-1-,7-2+
B-48	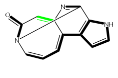 $C_{12}H_7N_3O$	7.9979	105.6074	-2.4001	227.4461	372.8104	InChI=1S/C12H7N3O/c16-10-6-9-7-2-1-5-15(10)12(14-9)11-8(7)3-4-13-11/h1-6,13H/b5-1-,7-2-
i-20	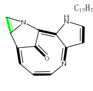 $C_{12}H_7N_3O$	15.5268	202.5069	-9.3695	116.0623	372.8175	InChI=1S/C12H7N3O/c16-12-7-2-1-4-13-8-3-5-14-10(8)11(12)15-6-9(7)15/h1-6,14H/b4-1-,7-2-,11-10-,13-8-
o-128	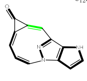 $C_{12}H_7N_3O$	3.9621	347.8911	-0.6529	-1.45	372.8191	InChI=1S/C12H7N3O/c16-12-7-2-1-5-15-10-3-4-13-11(10)9(14-15)6-8(7)12/h1-6,13H/b5-1-,7-2+,8-6+

H-11	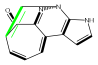	$C_{12}H_7N_3O$	7.7682	101.0018	-1.8472	217.5197	373.1574	InChI=1S/C12H7N3O/c16-11-7-2-1-3-8-9-4-5-13-12(9)15(6-7)14-10(8)11/h1-6,13H
H-92	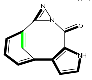	$C_{12}H_7N_3O$	18.759	215.631	-12.3191	123.0106	373.4137	InChI=1S/C12H7N3O/c16-12-10-9(4-5-13-10)7-2-1-3-8(6-7)11-14-15(11)12/h1-6,13H
o-151	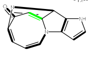	$C_{12}H_7N_3O$	10.2508	108.5215	-2.5487	205.4285	373.4321	InChI=1S/C12H7N3O/c16-10-6-9-12-11-8(3-4-13-11)15(9)5-1-2-7(10)14-12/h1-6,13H/b5-1-,7-2+
X-43	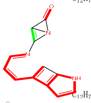	$C_{12}H_7N_3O$	26.8871	351.7298	-16.9523	7.7793	373.5181	InChI=1S/C12H7N3O/c16-12-10-11(15(10)12)14-4-1-2-7-6-9-8(7)3-5-13-9/h1-6,13H/b2-1-,14-4+
o-168	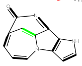	$C_{12}H_7N_3O$	10.7184	203.1599	-1.2724	108.7099	373.5567	InChI=1S/C12H7N3O/c16-12-7-2-1-5-15-8-3-4-13-10(8)11(14-12)9(15)6-7/h1-6,13H
A-89	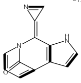	$C_{12}H_7N_3O$	3.2826	303.0629	-2.9036	67.9105	373.5658	InChI=1S/C12H7N3O/c16-12-8-2-1-5-15(12)11(9-6-14-9)10-7(8)3-4-13-10/h1-6,13H/b11-9-
A-144	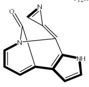	$C_{12}H_7N_3O$	3.2847	303.062	-2.9043	67.9121	373.5658	InChI=1S/C12H7N3O/c16-12-8-2-1-5-15(12)11(9-6-14-9)10-7(8)3-4-13-10/h1-6,13H/b11-9+
p1-32	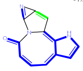	$C_{12}H_7N_3O$	12.7142	181.5779	-8.2325	176.9694	373.7968	InChI=1S/C12H7N3O/c16-10-3-1-2-7-4-5-13-11(7)9-6-8-12(14-8)15(9)10/h1-6,13H/b3-1-,7-2-,11-9+
p1-17	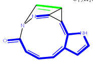	$C_{12}H_7N_3O$	15.7571	184.3735	-8.4936	163.1543	373.8377	InChI=1S/C12H7N3O/c16-9-3-1-2-7-4-5-13-11(7)10-8-6-15(9)14-12(8)10/h1-6,13H/b3-1-,7-2-,11-10+
i-22	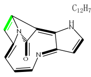	$C_{12}H_7N_3O$	12.3699	144.2187	-8.6058	192.0903	374.0981	InChI=1S/C12H7N3O/c16-12-10-9-6-7(15(9)12)2-1-4-13-8-3-5-14-11(8)10/h1-6,14H/b2-1-,4-1-,7-2-,11-10+,13-4+,13-8+
H1-105	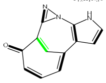	$C_{12}H_7N_3O$	21.1779	167.9882	-2.7615	159.696	374.2126	InChI=1S/C12H7N3O/c16-10-3-1-2-7-6-9(10)12-14-15(12)11-8(7)4-5-13-11/h1-6,13H
X22-43	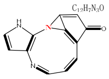	$C_{12}H_7N_3O$	8.7583	216.9879	-7.5247	149.3884	374.2401	InChI=1S/C12H7N3O/c16-10-6-9-11-7(10)2-1-4-13-8-3-5-14-12(8)15(9)11/h1-6,14H/b2-1-,13-4-
X22-49	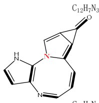	$C_{12}H_7N_3O$	9.8106	248.2682	-6.8284	116.1364	374.3756	InChI=1S/C12H7N3O/c16-11-7-6-15-9(10(7)11)2-1-4-13-8-3-5-14-12(8)15/h1-6,14H/b2-1-,13-4-
X23-28	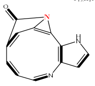	$C_{12}H_7N_3O$	24.2066	246.3291	-9.9084	96.0881	374.6283	InChI=1S/C12H7N3O/c16-12-7-2-1-4-13-8-3-5-14-10(8)11-9(6-7)15(11)12/h1-6,14H/b2-1-,4-1-,7-2+,11-10-,13-4-,13-8-
H11-33	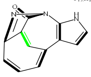	$C_{12}H_7N_3O$	7.4581	147.2728	-3.3255	185.5401	374.6385	InChI=1S/C12H7N3O/c16-12-9-6-7-2-1-3-10(9)14-15(12)11-8(7)4-5-13-11/h1-6,13H
E-70	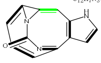	$C_{12}H_7N_3O$	15.0309	229.3004	-5.0131	117.0212	374.6747	InChI=1S/C12H7N3O/c16-12-14-8-2-1-3-10-11(15(10)12)6-9-7(8)4-5-13-9/h1-6,13H/b2-1-,10-3-,11-6-,14-8-
o-210	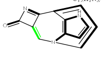	$C_{12}H_7N_3O$	6.9856	158.9022	-2.3393	173.9435	375.2987	InChI=1S/C12H7N3O/c16-12-8-6-15-5-1-2-7(10(8)14-12)11-9(15)3-4-13-11/h1-6,13H
o-157	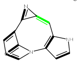	$C_{12}H_7N_3O$	11.0529	252.6149	-4.6084	92.0685	375.7524	InChI=1S/C12H7N3O/c16-12-7-2-1-5-15(12)10-3-4-13-8(10)6-9-11(7)14-9/h1-6,13H/b9-6+
X22-53	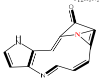	$C_{12}H_7N_3O$	19.4596	232.8245	-11.2555	102.4595	375.7914	InChI=1S/C12H7N3O/c16-12-10-6-8-7(3-5-14-8)13-4-1-2-9-11(12)15(9)10/h1-6,14H/b2-1-,10-6+,13-4+

X-35	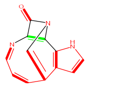	$C_{12}H_7N_3O$	8.0975	207.2664	-2.0586	139.7433	375.8136	InChI=1S/C12H7N3O/c16-12-10-11-9-8(3-5-14-9)7(6-15(11)12)2-1-4-13-10/h1-6,14H/b2-1-,13-4-
q11-13	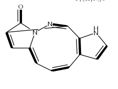	$C_{12}H_7N_3O$	32.3262	104.4232	-1.9383	169.2389	375.894	InChI=1S/C12H7N3O/c16-12-9-6-8-3-1-2-7-4-5-13-10(7)11(9)14-15(8)12/h1-6,13H/b2-1-,8-3-
B-20	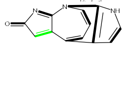	$C_{12}H_7N_3O$	10.9926	196.8951	-3.1754	128.9184	375.9011	InChI=1S/C12H7N3O/c16-10-6-9-7-2-1-5-15(12(9)14-10)11-8(7)3-4-13-11/h1-6,13H
G-4	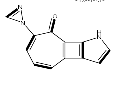	$C_{12}H_7N_3O$	34.3503	306.7886	-0.6493	25.9443	375.9638	InChI=1S/C12H7N3O/c16-12-9(15-6-14-15)3-1-2-7-8-4-5-13-11(8)10(7)12/h1-6,13H
C-24	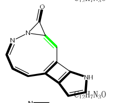	$C_{12}H_7N_3O$	9.9285	333.9921	-6.2759	26.6744	376.0325	InChI=1S/C12H7N3O/c16-12-10-6-9-7(2-1-4-14-15(10)12)8-3-5-13-11(8)9/h1-6,13H/b2-1-,10-6-,14-4-
n-202	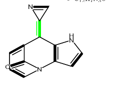	$C_{12}H_7N_3O$	3.3104	304.3819	-2.8024	69.9947	376.0421	InChI=1S/C12H7N3O/c16-12-7-2-1-5-15(12)9-3-4-13-11(9)10(7)8-6-14-8/h1-6,13H/b10-8+
o-42	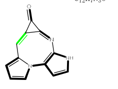	$C_{12}H_7N_3O$	4.2829	336.5798	-1.4156	27.9592	376.3951	InChI=1S/C12H7N3O/c16-11-8-6-7-2-1-5-15(7)9-3-4-13-12(9)14-10(8)11/h1-6,13H/b8-6+,14-10+
G1-54	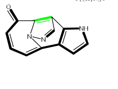	$C_{12}H_7N_3O$	9.0853	101.5378	-4.2033	234.7189	376.4993	InChI=1S/C12H7N3O/c16-10-3-1-2-9-7-4-5-13-11(7)8-6-14-15(9)12(8)10/h1-6,13H
X-51	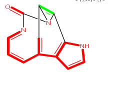	$C_{12}H_7N_3O$	40.7009	178.1793	-13.6208	127.4517	376.6416	InChI=1S/C12H7N3O/c16-12-14-4-1-2-7-6-9-11(15(9)12)10-8(7)3-5-13-10/h1-6,13H/b2-1-,14-4+
o-111	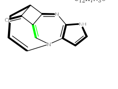	$C_{12}H_7N_3O$	6.8323	71.5989	-3.1054	264.3496	377.0699	InChI=1S/C12H7N3O/c16-11-7-2-1-5-15-6-8(11)10(7)14-12-9(15)3-4-13-12/h1-6,13H/b5-1-,7-2-
H11-142	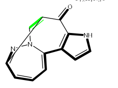	$C_{12}H_7N_3O$	29.2906	87.3477	-0.6664	194.6325	377.1724	InChI=1S/C12H7N3O/c16-12-8-6-15-10(3-1-2-9(8)14-15)7-4-5-13-11(7)12/h1-6,13H
I-78	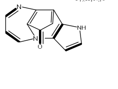	$C_{12}H_7N_3O$	30.8332	146.5095	-1.9266	131.4504	377.2	InChI=1S/C12H7N3O/c16-9-6-7-10-8(2-4-14-10)15-5-1-3-13-11(7)12(9)15/h1-6,14H
x4-25	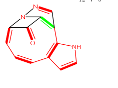	$C_{12}H_7N_3O$	10.3645	210.9421	-2.1629	126.8696	377.3435	InChI=1S/C12H7N3O/c16-12-9-3-1-2-7-4-5-13-10(7)8-6-14-15(9)11(8)12/h1-6,13H/b2-1-,9-3+
H-26	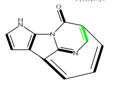	$C_{12}H_7N_3O$	13.0317	161.4802	-4.5056	174.7297	377.4772	InChI=1S/C12H7N3O/c16-12-7-2-1-3-8-9-4-5-13-10(9)15(12)11(8)14-6-7/h1-6,13H/b2-1-,3-1-,7-2+,8-3-
A-182	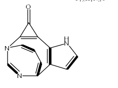	$C_{12}H_7N_3O$	27.2801	243.3722	-9.1879	95.3855	377.5027	InChI=1S/C12H7N3O/c16-12-9-10-7(3-4-13-10)8-2-1-5-15(6-14-8)11(9)12/h1-6,13H
H1-17	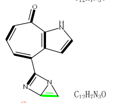	$C_{12}H_7N_3O$	24.1073	230.853	−18.0462	97.4676	377.6856	InChI=1S/C12H7N3O/c16-9-3-1-2-8(7-4-5-13-11(7)9)12-14-10-6-15(10)12/h1-6,13H
X-32	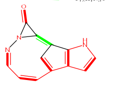	$C_{12}H_7N_3O$	9.5610	307.7570	-5.8589	52.9577	377.9733	InChI=1S/C12H7N3O/c16-12-11-9-6-7(2-1-4-14-15(11)12)8-3-5-13-10(8)9/h1-6,13H/b2-1-,11-9+,14-4-
x9-44	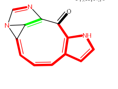	$C_{12}H_7N_3O$	6.0257	327.4753	-2.1886	26.0059	378.4639	InChI=1S/C12H7N3O/c16-12-9-7(4-5-13-9)2-1-3-8-11-10(12)14-6-15(8)11/h1-6,13H/b2-1-,8-3+
k-27	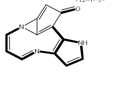	$C_{12}H_7N_3O$	9.0743	181.696	-6.6891	174.533	378.6925	InChI=1S/C12H7N3O/c16-9-6-8-12-10(9)11-7(2-4-14-11)13-3-1-5-15(8)12/h1-6,14H/b5-1-,13-3+

G-51	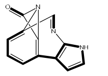	$C_{12}H_7N_3O$	23.8946	217.9301	-7.3242	126.2462	378.8251	InChI=1S/C12H7N3O/c16-12-10-8-3-1-2-7(15(10)12)6-14-11-9(8)4-5-13-11/h1-6,13H/b14-6+
x7-15	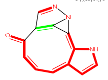	$C_{12}H_7N_3O$	18.2071	186.3008	-7.3792	158.2424	379.2397	InChI=1S/C12H7N3O/c16-9-3-1-2-7-4-5-13-10(7)12-11-8(9)6-14-15(11)12/h1-6,13H/b3-1-,7-2-,12-10-
q1-58	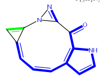	$C_{12}H_7N_3O$	38.9115	233.0783	-26.5945	92.9067	379.2409	InChI=1S/C12H7N3O/c16-11-10-7(4-5-13-10)2-1-3-8-6-9(8)15-12(11)14-15/h1-6,13H/b2-1-,8-3-
B-47	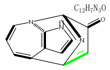	$C_{12}H_7N_3O$	9.3124	108.5350	-2.9316	219.3928	379.2905	InChI=1S/C12H7N3O/c16-10-6-9-7-2-1-5-15(12(10)14-9)11-8(7)3-4-13-11/h1-6,13H
G1-8	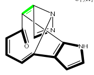	$C_{12}H_7N_3O$	10.2334	146.4419	-2.3512	195.8886	379.3802	InChI=1S/C12H7N3O/c16-10-3-1-2-8-7-4-5-13-12(7)11(10)9-6-14-15(8)9/h1-6,13H/b3-1-,8-2-
G1-61	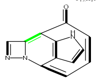	$C_{12}H_7N_3O$	10.2352	146.3976	-2.3533	195.9353	379.3802	InChI=1S/C12H7N3O/c16-10-3-1-2-8-7-4-5-13-12(7)11(10)9-6-14-15(8)9/h1-6,13H/b3-1-,8-2+
o-186	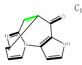	$C_{12}H_7N_3O$	4.7682	79.6515	-1.9862	261.9724	379.6236	InChI=1S/C12H7N3O/c16-12-7-6-10-14-8(7)2-1-5-15(10)9-3-4-13-11(9)12/h1-6,13H
q1-51	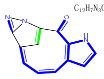	$C_{12}H_7N_3O$	19.8475	151.5249	-4.6765	179.909	379.6393	InChI=1S/C12H7N3O/c16-11-9-6-8(12-14-15(9)12)3-1-2-7-4-5-13-10(7)11/h1-6,13H/b2-1-,8-3+
n-41	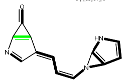	$C_{12}H_7N_3O$	41.5621	301.4429	-18.6991	37.8313	379.8706	InChI=1S/C12H7N3O/c16-11-9-7(6-14-10(9)11)2-1-5-15-8-3-4-13-12(8)15/h1-6,13H/b5-1-,7-2+
s11-13	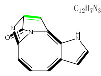	$C_{12}H_7N_3O$	6.4836	132.8394	-3.74	224.8715	379.8864	InChI=1S/C12H7N3O/c16-12-8-6-9-11-7(4-5-13-11)2-1-3-10(14-8)15(9)12/h1-6,13H/b2-1-,3-1-,7-2-,10-3-,11-9+
o-154	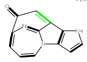	$C_{12}H_7N_3O$	10.7415	186.4911	-1.3167	139.0622	379.9356	InChI=1S/C12H7N3O/c16-10-6-7-11-9(3-4-13-11)15-5-1-2-8(10)14-12(7)15/h1-6,13H
q-32	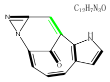	$C_{12}H_7N_3O$	20.5237	171.7874	-2.2849	159.7858	380.0552	InChI=1S/C12H7N3O/c16-12-8-6-10-14-15(10)9(12)3-1-2-7-4-5-13-11(7)8/h1-6,13H/b2-1-,9-3+
k-20	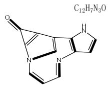	$C_{12}H_7N_3O$	31.7332	203.2749	-12.9333	136.3011	380.2208	InChI=1S/C12H7N3O/c16-12-9-7-6-15(11(9)12)5-1-3-13-8-2-4-14-10(7)8/h1-6,14H/b5-1-,13-3+
G-58	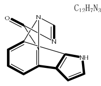	$C_{12}H_7N_3O$	7.7547	178.3329	-2.0206	160.4325	380.378	InChI=1S/C12H7N3O/c16-12-11-8-2-1-3-9(15(11)6-14-12)10-7(8)4-5-13-10/h1-6,13H
x4-18	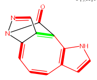	$C_{12}H_7N_3O$	13.8077	127.1981	-5.3276	208.9544	380.4401	InChI=1S/C12H7N3O/c16-12-10-8-6-14-15(12)9(8)3-1-2-7-4-5-13-11(7)10/h1-6,13H/b2-1-,3-1-,7-2-,9-3+,11-10-
q-11	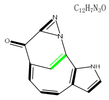	$C_{12}H_7N_3O$	21.8723	191.5779	-6.127	140.6933	380.5765	InChI=1S/C12H7N3O/c16-11-8-3-1-2-7-4-5-13-10(7)9(6-8)15-12(11)14-15/h1-6,13H/b2-1-,3-1-,7-2-,8-3+,10-9-
B-146	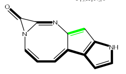	$C_{12}H_7N_3O$	5.8114	306.8303	-4.1638	63.8805	380.6165	InChI=1S/C12H7N3O/c16-12-11-14-10-6-9-8(3-4-13-9)7(10)2-1-5-15(11)12/h1-6,13H/b5-1-,7-2-,14-11-
n-54	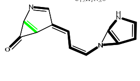	$C_{12}H_7N_3O$	41.5804	302.1261	-18.7673	37.9675	380.7042	InChI=1S/C12H7N3O/c16-11-9-7(6-14-10(9)11)2-1-5-15-8-3-4-13-12(8)15/h1-6,13H/b5-1-,7-2-
E-5	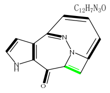	$C_{12}H_7N_3O$	17.7844	190.6341	-2.4951	138.0758	380.8977	InChI=1S/C12H7N3O/c16-12-10-6-7-2-1-3-9(14-15(7)10)8-4-5-13-11(8)12/h1-6,13H

x5-45	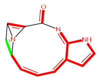	$C_{12}H_7N_3O$	19.1363	241.4263	-8.2395	67.4453	380.9713	InChI=1S/C12H7N3O/c16-12-10-9-6-8(15(9)10)3-1-2-7-4-5-13-11(7)14-12/h1-6H,(H,13,14,16)
B-134	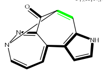	$C_{12}H_7N_3O$	8.7183	95.3234	-3.4991	232.5639	381.0257	InChI=1S/C12H7N3O/c16-12-10-6-9-7(3-4-13-9)8-2-1-5-15(10)14-11(8)12/h1-6,13H
o-44	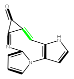	$C_{12}H_7N_3O$	3.8275	328.5753	-1.7103	40.1384	381.0944	InChI=1S/C12H7N3O/c16-12-7-6-8-9(3-4-13-8)15-5-1-2-10(15)14-11(7)12/h1-6,13H/b7-6-,14-11+
o-14	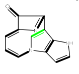	$C_{12}H_7N_3O$	12.6666	148.1222	-4.8182	184.2659	381.4021	InChI=1S/C12H7N3O/c16-12-8-2-1-5-15-6-7(11(12)14-8)10-9(15)3-4-13-10/h1-6,13H/b5-1-,8-2-
F-56	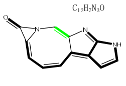	$C_{12}H_7N_3O$	6.8464	324.1257	-4.3256	48.4684	381.404	InChI=1S/C12H7N3O/c16-12-10-3-1-2-7-8-4-5-13-11(8)14-9(7)6-15(10)12/h1-6H,(H,13,14)/b2-1-,9-6+,10-3-
G1-64	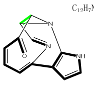	$C_{12}H_7N_3O$	12.9837	280.9963	-5.6158	44.8392	381.473	InChI=1S/C12H7N3O/c16-10-3-1-2-8-7-4-5-13-12(7)15-9(6-14-8)11(10)15/h1-6,13H/b3-1-,8-2-
E-56	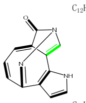	$C_{12}H_7N_3O$	5.746	109.6698	-2.6643	231.0018	381.6102	InChI=1S/C12H7N3O/c16-12-7-2-1-3-10-8-4-5-13-11(8)9(7)6-15(12)14-10/h1-6,13H/b3-1-,7-2+
A1-35	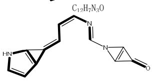	$C_{12}H_7N_3O$	65.4155	299.7358	-36.5455	36.1949	381.6798	InChI=1S/C12H7N3O/c16-12-10-11(12)15(10)6-13-4-1-2-7-8-3-5-14-9(7)8/h1-6,14H/b4-1-,7-2-,13-6+
A2-55	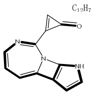	$C_{12}H_7N_3O$	12.6811	376.8157	-2.9406	-3.9061	381.7175	InChI=1S/C12H7N3O/c16-10-6-8(10)12-13-4-1-2-9-7-3-5-14-11(7)15(9)12/h1-6,14H
s12-7	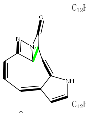	$C_{12}H_7N_3O$	33.5121	302.0714	-11.4204	33.4162	381.8104	InChI=1S/C12H7N3O/c16-12-8-6-10-7(4-5-13-10)2-1-3-9-11(8)15(12)14-9/h1-6,13H/b3-1-,7-2-,10-6-
n-38	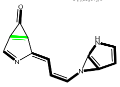	$C_{12}H_7N_3O$	41.6362	299.5563	-19.1408	40.9285	382.0145	InChI=1S/C12H7N3O/c16-11-7-6-14-8(10(7)11)2-1-5-15-9-3-4-13-12(9)15/h1-6,13H/b5-1-,8-2-
n-27	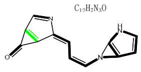	$C_{12}H_7N_3O$	41.6417	299.5758	-19.1411	40.9232	382.0531	InChI=1S/C12H7N3O/c16-11-7-6-14-8(10(7)11)2-1-5-15-9-3-4-13-12(9)15/h1-6,13H/b5-1-,8-2+
o-122	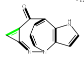	$C_{12}H_7N_3O$	18.906	149.5705	-14.5719	186.3231	382.4127	InChI=1S/C12H7N3O/c16-12-7-2-1-5-15(14-9-6-8(9)12)10-3-4-13-11(7)10/h1-6,13H/b14-9-
B-67	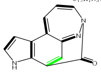	$C_{12}H_7N_3O$	8.2463	154.0109	-3.3952	183.2734	382.6586	InChI=1S/C12H7N3O/c16-12-9-6-10-7(2-1-5-15(12)14-10)8-3-4-13-11(8)9/h1-6,13H
G1-57	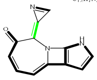	$C_{12}H_7N_3O$	5.1452	378.8144	-1.9874	-4.31	382.6774	InChI=1S/C12H7N3O/c16-10-3-1-2-9-7-4-5-13-12(7)15(9)11(10)8-6-14-8/h1-6,13H/b11-8+
p11-8	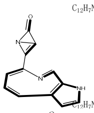	$C_{12}H_7N_3O$	24.0195	362.1523	-14.0581	2.3418	382.7947	InChI=1S/C12H7N3O/c16-12-11-10(15(11)12)8-3-1-2-7-4-5-13-9(7)6-14-8/h1-6,13H/b2-1-,3-1-,7-2?,8-3-,9-6+,14-6+,14-8-
q-26	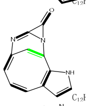	$C_{12}H_7N_3O$	19.1611	222.3701	-5.7734	125.1465	383.0988	InChI=1S/C12H7N3O/c16-12-11-14-8-3-1-2-7-4-5-13-10(7)9(6-8)15(11)12/h1-6,13H/b2-1-,3-1-,7-2-,8-3+,10-9-
A-40	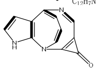	$C_{12}H_7N_3O$	28.9064	219.9518	-12.6774	112.3738	383.1125	InChI=1S/C12H7N3O/c16-11-8-6-14-9-2-1-5-15(10(8)11)12-7(9)3-4-13-12/h1-6,13H/b14-6-
A-97	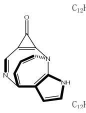	$C_{12}H_7N_3O$	28.9037	219.9567	-12.6751	112.3776	383.1125	InChI=1S/C12H7N3O/c16-12-9-6-14-15(11(9)12)5-1-2-7-8-3-4-13-10(7)8/h1-6,13H/b5-1-,7-2-

s11-22	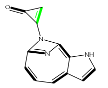 $C_{12}H_7N_3O$	45.6984	221.2654	-20.2391	109.0682	383.5982	InChI=1S/C12H7N3O/c16-9-6-8(9)15-10-3-1-2-7-4-5-13-11(7)12(15)14-10/h1-6,13H/b2-1-,3-1-,7-2-,10-3+,12-11-
H-7	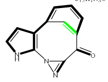 $C_{12}H_7N_3O$	31.4162	237.5892	-0.4189	91.6489	383.7032	InChI=1S/C12H7N3O/c16-10-8-3-1-2-7(6-8)9-4-5-13-11(9)15-12(10)14-15/h1-6,13H
F-45	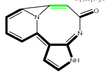 $C_{12}H_7N_3O$	16.0704	208.1672	-7.8066	142.9849	383.8432	InChI=1S/C12H7N3O/c16-11-6-10-9-3-1-2-8(15(9)10)7-4-5-13-12(7)14-11/h1-6H,(H,13,14,16)
H1-108	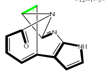 $C_{12}H_7N_3O$	22.9479	226.36	-12.6454	106.985	383.9893	InChI=1S/C12H7N3O/c16-10-3-1-2-7-6-9-12(15(9)10)14-11-8(7)4-5-13-11/h1-6,13H/b3-1-,7-2+
r-47	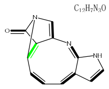 $C_{12}H_7N_3O$	36.2934	122.4188	-5.9502	154.0971	384.012	InChI=1S/C12H7N3O/c16-12-10-8-6-15(12)9(10)3-1-2-7-4-5-13-11(7)14-8/h1-6H,(H,13,14)/b3-1-,7-2-
p1-8	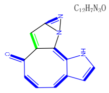 $C_{12}H_7N_3O$	27.4471	251.7443	0.1078	91.3611	384.0151	InChI=1S/C12H7N3O/c16-9-3-1-2-7-4-5-13-11(7)12-8(9)6-10-14-15(10)12/h1-6,13H/b3-1-,7-2-,12-11-
x9-27	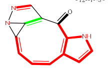 $C_{12}H_7N_3O$	6.7249	331.3418	-2.1824	27.9438	384.0773	InChI=1S/C12H7N3O/c16-12-8-6-14-15-9(11(8)15)3-1-2-7-4-5-13-10(7)12/h1-6,13H/b2-1-,9-3+
E-35	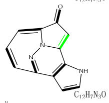 $C_{12}H_7N_3O$	17.1464	230.9232	0.4309	98.0555	384.2176	InChI=1S/C12H7N3O/c16-11-6-10-12-7(4-5-13-12)8-2-1-3-9(11)15(10)14-8/h1-6,13H
o-231	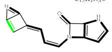 $C_{12}H_7N_3O$	5.2354	396.1676	-4.3733	-6.1106	384.4764	InChI=1S/C12H7N3O/c16-12-11-9(3-4-13-11)15(12)5-1-2-7-6-8-10(7)14-8/h1-6,13H/b5-1-,7-2+
H-139	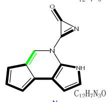 $C_{12}H_7N_3O$	2.8751	321.2462	-2.2854	54.0038	384.5051	InChI=1S/C12H7N3O/c16-12-11(14-12)15-6-7-2-1-3-8(7)9-4-5-13-10(9)15/h1-6,13H
p1-28	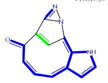 $C_{12}H_7N_3O$	21.5905	170.6936	-3.6864	167.9083	384.519	InChI=1S/C12H7N3O/c16-10-3-1-2-7-4-5-13-11(7)9-6-8(10)12-14-15(9)12/h1-6,13H/b3-1-,7-2-,11-9-
X-31	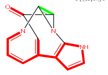 $C_{12}H_7N_3O$	24.0536	224.7255	-6.7368	86.1720	384.5838	InChI=1S/C12H7N3O/c16-9-6-7-2-1-4-13-12-10(9)15(12)11-8(7)3-5-14-11/h1-6,14H/b2-1-,13-4+
x4-11	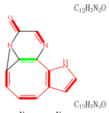 $C_{12}H_7N_3O$	22.193	267.0774	-10.6831	77.1749	384.6324	InChI=1S/C12H7N3O/c16-9-6-14-11-10-7(4-5-13-10)2-1-3-8-12(11)15(8)9/h1-6,13H/b2-1-,3-1-,7-2-,8-3+,11-10-
H11-116	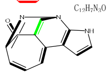 $C_{12}H_7N_3O$	6.9576	98.683	-0.3222	235.294	384.8603	InChI=1S/C12H7N3O/c16-11-9-6-15-12-8(4-5-13-12)7(9)2-1-3-10(11)14-15/h1-6,13H
B-182	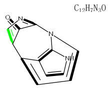 $C_{12}H_7N_3O$	12.1079	101.9582	-3.6023	220.0471	385.0658	InChI=1S/C12H7N3O/c16-10-9-6-14-12(10)15-5-1-2-7(9)8-3-4-13-11(8)15/h1-6,13H
B-197	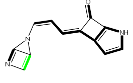 $C_{12}H_7N_3O$	12.1058	101.9626	-3.6014	220.0521	385.0658	InChI=1S/C12H7N3O/c16-11-8(7-3-4-13-10(7)11)2-1-5-15-9-6-14-12(9)15/h1-6,13H/b5-1-,8-2-
F-22	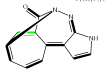 $C_{12}H_7N_3O$	32.3923	90.7082	-1.2189	175.0698	385.2188	InChI=1S/C12H7N3O/c16-12-10-6-7-2-1-3-8(10)9-4-5-13-11(9)14-15(7)12/h1-6H,(H,13,14)
G-39	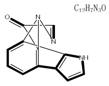 $C_{12}H_7N_3O$	8.0588	180.4439	-1.9583	163.7656	385.4359	InChI=1S/C12H7N3O/c16-10-6-14-15-9-3-1-2-8(12(10)15)7-4-5-13-11(7)9/h1-6,13H
H1-44	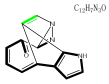 $C_{12}H_7N_3O$	34.7146	317.9273	-0.913	24.4868	385.5878	InChI=1S/C12H7N3O/c16-10-3-1-2-7-8-4-5-13-11(8)9(7)6-15-12(10)14-15/h1-6,13H/b3-1-,7-2-,9-6-

H11-151	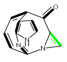 C12H7N3O	10.3889	246.9327	-5.0409	93.0635	385.6224	InChI=1S/C12H7N3O/c16-11-7-2-1-3-10(15-6-9(11)15)14-12-8(7)4-5-13-12/h1-6H,(H,13,14)
r-24	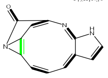 C12H7N3O	28.6283	260.1755	-15.9036	60.9602	385.7178	InChI=1S/C12H7N3O/c16-12-8-6-10-9(15(10)12)3-1-2-7-4-5-13-11(7)14-8/h1-6H,(H,13,14)
C-59	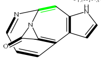 C12H7N3O	22.0180	224.8696	-8.4195	115.8410	385.7520	InChI=1S/C12H7N3O/c16-12-11-8-2-1-4-14-10(15(11)12)6-9-7(8)3-5-13-9/h1-6,13H/b2-1-,14-4-
s11-50	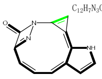 C12H7N3O	20.5703	231.4414	-15.2791	110.2615	386.1579	InChI=1S/C12H7N3O/c16-12-9-3-1-2-7-4-5-13-11(7)8-6-10(8)15(12)14-9/h1-6,13H/b3-1-,7-2-,11-8+
X23-22	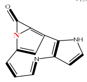 C12H7N3O	20.1554	243.1066	-12.0305	121.0635	386.1651	InChI=1S/C12H7N3O/c16-12-11-8-6-7(15(11)12)2-1-4-13-9-3-5-14-10(8)9/h1-6,14H/b2-1-,13-4+
B-29	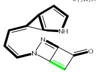 C12H7N3O	21.3203	221.5702	-13.4457	138.1159	386.1938	InChI=1S/C12H7N3O/c16-10-6-9-12(10)14-15(9)5-1-2-7-8-3-4-13-11(7)8/h1-6,13H/b5-1-,7-2-
H1-100	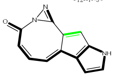 C12H7N3O	10.8615	316.3868	-3.3016	44.764	386.4151	InChI=1S/C12H7N3O/c16-11-3-1-2-7-8-4-5-13-10(8)6-9(7)12-14-15(11)12/h1-6,13H/b3-1-,7-2-
q-45	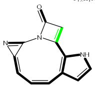 C12H7N3O	29.8538	302.6913	-1.3228	50.5487	386.4229	InChI=1S/C12H7N3O/c16-10-6-9-11-7(4-5-13-11)2-1-3-8-12(14-8)15(9)10/h1-6,13H/b2-1-,8-3+
E-11	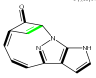 C12H7N3O	16.3085	190.5955	-5.7162	151.568	386.6361	InChI=1S/C12H7N3O/c16-11-7-2-1-3-9-8-4-5-13-12(8)15(14-9)10(11)6-7/h1-6,13H/b3-1-,7-2+
n-201	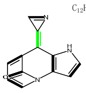 C12H7N3O	30.8459	249.3627	-1.7002	106.769	386.8984	InChI=1S/C12H7N3O/c16-12-7-2-1-5-15(12)9-3-4-13-11(9)10(7)8-6-14-8/h1-6,13H/b10-8-
x6-33	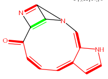 C12H7N3O	14.8256	210.6749	-11.1197	152.5941	386.9951	InChI=1S/C12H7N3O/c16-9-3-1-2-7-4-5-13-8(7)6-15-11-10(9)14-12(11)15/h1-6,13H/b3-1-,7-2-,8-6+
s11-33	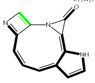 C12H7N3O	2.2266	363.2665	-2.1468	13.8607	387.0896	InChI=1S/C12H7N3O/c16-12-11-10-7(4-5-13-10)2-1-3-8-9(6-14-8)15(11)12/h1-6,13H/b3-1-,7-2-,11-10+
s11-66	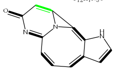 C12H7N3O	16.0458	204.1664	-5.5498	147.7744	387.1428	InChI=1S/C12H7N3O/c16-10-6-8-12-11-7(4-5-13-11)2-1-3-9(14-10)15(8)12/h1-6,13H/b3-1-,7-2-,12-11+
B-26	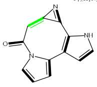 C12H7N3O	13.3250	239.7498	-7.9708	108.4249	387.3239	InChI=1S/C12H7N3O/c16-10-6-8-12(14-8)11-7(3-4-13-11)9-2-1-5-15(9)10/h1-6,13H/b8-6+
I-70	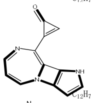 C12H7N3O	12.3622	374.8131	-2.963	-0.989	387.3557	InChI=1S/C12H7N3O/c16-9-6-7(9)10-12-11-8(2-4-14-11)15(12)5-1-3-13-10/h1-6,14H
H1-133	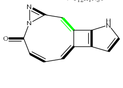 C12H7N3O	28.4088	323.5817	-0.7958	25.3718	387.7236	InChI=1S/C12H7N3O/c16-11-3-1-2-7-8-4-5-13-12(8)9(7)6-10-14-15(10)11/h1-6,13H/b3-1-,7-2-,9-6+
n-152	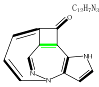 C12H7N3O	10.381	147.2249	-2.8163	178.2509	387.7757	InChI=1S/C12H7N3O/c16-12-7-2-1-5-15-9-3-4-13-11(9)10(12)8(7)6-14-15/h1-6,13H/b5-1-,7-2+
C-38	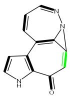 C12H7N3O	13.6934	182.8836	-7.9419	157.0088	388.0407	InChI=1S/C12H7N3O/c16-10-6-9-12-8(2-1-4-14-15(9)12)7-3-5-13-11(7)10/h1-6,13H
q1-16	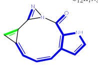 C12H7N3O	50.3245	260.6623	-31.441	85.7042	388.6626	InChI=1S/C12H7N3O/c16-12-10-7(4-5-13-10)2-1-3-8-6-9(8)11-14-15(11)12/h1-6,13H/b2-1-,8-3-

q-33	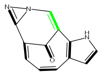	$C_{12}H_7N_3O$	20.5913	171.5996	-2.3227	170.0114	388.7535	InChI=1S/C12H7N3O/c16-11-8-3-1-2-7-4-5-13-10(7)9(11)6-15-12(8)14-15/h1-6,13H/b2-1-,8-3+
I-31	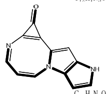	$C_{12}H_7N_3O$	30.0355	241.9833	-11.511	104.7319	389.0207	InChI=1S/C12H7N3O/c16-12-10-9-6-7-8(2-4-13-7)15(9)5-1-3-14-11(10)12/h1-6,13H/b5-1-,14-3-
r-48	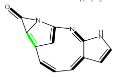	$C_{12}H_7N_3O$	16.6312	210.4221	-7.3584	150.5829	389.0624	InChI=1S/C12H7N3O/c16-12-10-8-3-1-2-7-4-5-13-11(7)14-9(6-8)15(10)12/h1-6H,(H,13,14)/b3-1-,7-2-
B-183	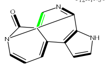	$C_{12}H_7N_3O$	9.4638	90.8963	-2.6880	233.3984	389.4499	InChI=1S/C12H7N3O/c16-12-9-6-14-11-10-8(3-4-13-10)7(9)2-1-5-15(11)12/h1-6,13H
o-45	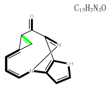	$C_{12}H_7N_3O$	29.1464	189.7053	-15.527	135.5273	389.4922	InChI=1S/C12H7N3O/c16-12-8-6-7(8)2-1-5-15-9-3-4-13-10(9)11(12)14-15/h1-6,13H/b5-1-,7-2+
x3-11	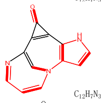	$C_{12}H_7N_3O$	29.5878	230.4044	-10.2537	111.7192	389.6294	InChI=1S/C12H7N3O/c16-12-9-7-6-15(5-1-3-13-7)8-2-4-14-11(8)10(9)12/h1-6,14H
X23-13	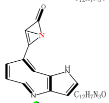	$C_{12}H_7N_3O$	26.8622	295.6441	-17.6454	71.3918	389.7792	InChI=1S/C12H7N3O/c16-12-11-10(15(11)12)7-2-1-4-13-8-3-5-14-9(8)6-7/h1-6,14H/b2-1-,4-1+,7-2+,7-6-,9-6-,13-4+,13-8-
H1-72	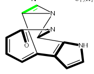	$C_{12}H_7N_3O$	9.1275	260.4201	-3.8942	92.8823	389.9076	InChI=1S/C12H7N3O/c16-10-3-1-2-7-8-4-5-13-11(8)12(14-10)15-6-9(7)15/h1-6,13H/b3-1-,7-2-,14-12-
B-220	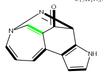	$C_{12}H_7N_3O$	7.0604	49.9000	-2.5844	293.1526	390.2323	InChI=1S/C12H7N3O/c16-12-9-6-15-5-1-2-7(9)8-3-4-13-10(8)11(12)14-15/h1-6,13H
H11-70	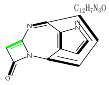	$C_{12}H_7N_3O$	11.0727	195.7445	-3.2136	163.4121	390.3056	InChI=1S/C12H7N3O/c16-11-6-10-14-8-2-1-3-9(15(10)11)7-4-5-13-12(7)8/h1-6,13H
r-60	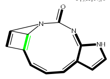	$C_{12}H_7N_3O$	26.9576	235.5927	-15.6562	143.7479	390.4587	InChI=1S/C12H7N3O/c16-12-14-11-7(4-5-13-11)2-1-3-8-6-9-10(8)15(9)12/h1-6H,(H,13,14,16)/b3-1-,7-2-
A2-58	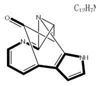	$C_{12}H_7N_3O$	24.6288	199.683	-8.8464	138.9161	390.4691	InChI=1S/C12H7N3O/c16-10-6-9-12-13-4-1-2-7(10)8-3-5-14-11(8)15(9)12/h1-6,14H/b4-1-,7-2-,13-12+
o-190	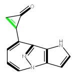	$C_{12}H_7N_3O$	43.2162	212.8658	-17.9671	122.5941	390.506	InChI=1S/C12H7N3O/c16-10-6-8(10)7-2-1-5-15-9-3-4-13-12(9)11(7)14-15/h1-6,13H
B-155	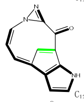	$C_{12}H_7N_3O$	4.3637	333.3225	-1.5723	33.7911	390.5621	InChI=1S/C12H7N3O/c16-11-9-6-7(8-3-4-13-10(8)9)2-1-5-15-12(11)14-15/h1-6,13H/b5-1-,7-2+
o-136	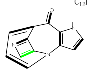	$C_{12}H_7N_3O$	8.9617	119.1315	-1.7455	217.4528	390.6212	InChI=1S/C12H7N3O/c16-12-7-2-1-5-15(9-6-14-10(7)9)8-3-4-13-11(8)12/h1-6,13H
A2-45	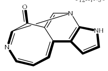	$C_{12}H_7N_3O$	9.761	90.2414	-3.6611	249.2791	391.02	InChI=1S/C12H7N3O/c16-10-9-6-15-11-8(3-5-14-11)7(9)2-1-4-13-12(10)15/h1-6,14H/b4-1-,7-2-,13-12-
q11-62	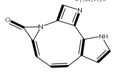	$C_{12}H_7N_3O$	2.3064	366.0954	-2.0956	13.5149	391.058	InChI=1S/C12H7N3O/c16-12-8-3-1-2-7-4-5-13-10(7)11-9(6-14-11)15(8)12/h1-6,13H/b2-1-,8-3-
q1-50	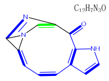	$C_{12}H_7N_3O$	17.4953	178.1749	-7.34	172.7485	391.3076	InChI=1S/C12H7N3O/c16-11-8-6-15-9(12(15)14-8)3-1-2-7-4-5-13-10(7)11/h1-6,13H/b2-1-,9-3-
H11-126	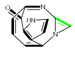	$C_{12}H_7N_3O$	18.3915	182.4848	-13.3984	151.815	391.8406	InChI=1S/C12H7N3O/c16-12-8-2-1-3-9(15-6-10(15)14-8)7-4-5-13-11(7)12/h1-6,13H/b2-1-,9-3+,14-8-

s12-40	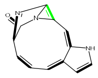	$C_{12}H_7N_3O$	32.2411	306.1649	-11.4919	32.4026	392.0367	InChI=1S/C12H7N3O/c16-12-8-3-1-2-7-4-5-13-9(7)6-10-11(14-8)15(10)12/h1-6,13H/b2-1-,3-1-,7-2-,8-3+,9-6+,10-6-
A-178	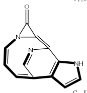	$C_{12}H_7N_3O$	16.6905	221.7856	-6.6724	134.3578	392.18	InChI=1S/C12H7N3O/c16-12-11-10-9-8(3-4-13-9)7(6-14-10)2-1-5-15(11)12/h1-6,13H/b5-1-,7-2+,11-10-
A-75	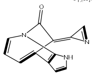	$C_{12}H_7N_3O$	3.8797	307.3977	-2.9374	80.4911	392.3123	InChI=1S/C12H7N3O/c16-12-10(9-6-14-9)7-2-1-5-15(12)11-8(7)3-4-13-11/h1-6,13H/b10-9+
A2-33	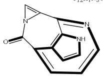	$C_{12}H_7N_3O$	16.0641	201.1743	-8.1536	158.8676	392.5144	InChI=1S/C12H7N3O/c16-12-8-2-1-4-13-11(9-6-15(9)12)10-7(8)3-5-14-10/h1-6,14H/b2-1-,4-1-,8-2+,13-4-,13-11-
o-201	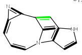	$C_{12}H_7N_3O$	11.0175	270.0908	-5.4091	98.6238	393.2011	InChI=1S/C12H7N3O/c16-12-7-6-9-10(3-4-13-9)15(12)5-1-2-8-11(7)14-8/h1-6,13H/b5-1-,8-2+
G-60	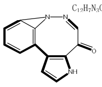	$C_{12}H_7N_3O$	27.0622	135.2239	-12.9603	202.5701	393.3255	InChI=1S/C12H7N3O/c16-10-6-14-15-9-3-1-2-8(12(9)15)7-4-5-13-11(7)10/h1-6,13H/b14-6-
E-64	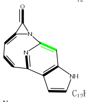	$C_{12}H_7N_3O$	6.1087	276.6665	-4.7719	98.5734	393.3497	InChI=1S/C12H7N3O/c16-12-10-3-1-2-8-7-4-5-13-9(7)6-11(14-8)15(10)12/h1-6,13H/b2-1-,10-3-
B-70	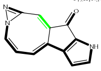	$C_{12}H_7N_3O$	15.9929	218.6936	-4.0322	129.8326	393.4414	InChI=1S/C12H7N3O/c16-12-9-6-10-14-15(10)5-1-2-7(9)8-3-4-13-11(8)12/h1-6,13H/b5-1-,7-2-,9-6+
o-206	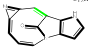	$C_{12}H_7N_3O$	3.9124	331.1109	-1.4793	44.3327	393.5433	InChI=1S/C12H7N3O/c16-12-7-6-9-8(14-9)2-1-5-15(12)10-3-4-13-11(7)10/h1-6,13H/b5-1-,7-6-,8-2+
o-134	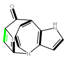	$C_{12}H_7N_3O$	7.2819	71.3418	-3.2078	277.0413	393.6694	InChI=1S/C12H7N3O/c16-12-7-2-1-5-15(10-6-8(12)14-10)9-3-4-13-11(7)9/h1-6,13H
A2-54	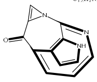	$C_{12}H_7N_3O$	30.4286	200.372	-16.4849	142.8395	393.6884	InChI=1S/C12H7N3O/c16-11-8-2-1-4-14-12(15-6-9(11)15)10-7(8)3-5-13-10/h1-6,13H/b2-1-,4-1-,8-2+,14-4-,14-12+
A2-57	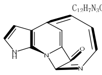	$C_{12}H_7N_3O$	9.6726	200.2529	-2.8935	153.8267	394.0419	InChI=1S/C12H7N3O/c16-10-9-6-7-2-1-4-13-12(10)15(9)11-8(7)3-5-14-11/h1-6,14H/b4-1-,7-2+,13-12+
B-224	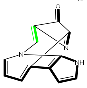	$C_{12}H_7N_3O$	11.1261	117.2096	-4.2309	220.9679	394.0717	InChI=1S/C12H7N3O/c16-11-9-6-15-5-1-2-7(10(11)14-9)8-3-4-13-12(8)15/h1-6,13H/b9-6+
o-133	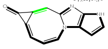	$C_{12}H_7N_3O$	4.1566	321.3279	-2.7564	59.2623	394.2847	InChI=1S/C12H7N3O/c16-11-7-2-1-5-15-9-3-4-13-12(9)14-10(15)6-8(7)11/h1-6,13H/b5-1-,7-2+,8-6+
B-65	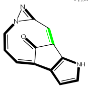	$C_{12}H_7N_3O$	2.8312	316.1776	-1.4743	61.6931	394.5034	InChI=1S/C12H7N3O/c16-12-8-2-1-5-15-10(14-15)6-9(12)11-7(8)3-4-13-11/h1-6,13H/b5-1-,8-2-,9-6-
H11-66	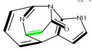	$C_{12}H_7N_3O$	12.5431	113.2213	-5.7391	227.4253	394.8312	InChI=1S/C12H7N3O/c16-11-6-9-7-2-1-3-10(14-9)15(11)12-8(7)4-5-13-12/h1-6,13H
q11-32	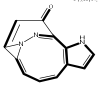	$C_{12}H_7N_3O$	23.6086	137.2225	-11.0093	201.3456	395.061	InChI=1S/C12H7N3O/c16-10-6-9-8-3-1-2-7-4-5-13-11(7)12(10)14-15(8)9/h1-6,13H/b2-1-,8-3-
H1-73	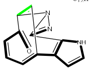	$C_{12}H_7N_3O$	16.7969	209.8785	-6.013	136.7235	395.0625	InChI=1S/C12H7N3O/c16-10-3-1-2-7-6-9(10)15-12(14-15)11-8(7)4-5-13-11/h1-6,13H
B-147	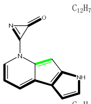	$C_{12}H_7N_3O$	3.1789	327.7203	-2.1795	55.2962	395.1918	InChI=1S/C12H7N3O/c16-12-11(14-12)15-5-1-2-8-7-3-4-13-9(7)6-10(8)15/h1-6,13H

B-200	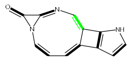 $C_{12}H_7N_3O$	8.7272	338.1042	-5.1547	45.1324	395.5303	InChI=1S/C12H7N3O/c16-12-11-14-6-9-7(2-1-5-15(11)12)8-3-4-13-10(8)9/h1-6,13H/b5-1-,7-2-,9-6+,14-11-
H11-91	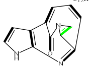 $C_{12}H_7N_3O$	16.9959	254.9599	-4.8991	101.1766	395.5609	InChI=1S/C12H7N3O/c16-12-8-2-1-3-9(10-6-15(10)12)14-11-7(8)4-5-13-11/h1-6H,(H,13,14)
A-102	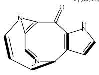 $C_{12}H_7N_3O$	5.6286	258.8255	-2.9818	100.1163	395.5672	InChI=1S/C12H7N3O/c16-12-10-7(3-4-13-10)8-2-1-5-15-9(6-14-8)11(12)15/h1-6,13H/b5-1-,8-2-,14-6-
B-252	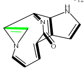 $C_{12}H_7N_3O$	39.2827	231.5208	-21.4907	110.9525	395.6011	InChI=1S/C12H7N3O/c16-12-8-2-1-5-15-6-9(15)11(14-12)10-7(8)3-4-13-10/h1-6,13H/b5-1-,8-2-
A-199	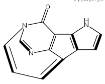 $C_{12}H_7N_3O$	11.7548	156.7419	-4.7505	191.6494	395.9290	InChI=1S/C12H7N3O/c16-12-9-10-8(3-4-13-10)7-2-1-5-15(12)6-14-11(7)9/h1-6,13H/b5-1-,7-2-
I-77	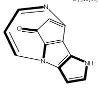 $C_{12}H_7N_3O$	17.696	160.2131	-4.4082	183.6305	396.0395	InChI=1S/C12H7N3O/c16-9-6-7-10-11-8(2-4-14-11)15(12(9)10)5-1-3-13-7/h1-6,14H/b5-1-,13-3-
p11-17	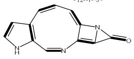 $C_{12}H_7N_3O$	17.774	288.1218	-10.1447	71.3682	396.4794	InChI=1S/C12H7N3O/c16-12-11-10-9(15(11)12)3-1-2-7-4-5-13-8(7)6-14-10/h1-6,13H/b2-1-,9-3+,14-6-
q1-14	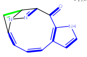 $C_{12}H_7N_3O$	22.5516	185.2736	-7.6494	163.2958	396.6427	InChI=1S/C12H7N3O/c16-12-10-7(4-5-13-10)2-1-3-8-6-9-11(12)14-15(8)9/h1-6,13H/b2-1-,8-3-
s11-58	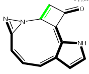 $C_{12}H_7N_3O$	32.6573	296.473	-0.7614	48.4964	396.6741	InChI=1S/C12H7N3O/c16-9-6-8-11(9)12-7(4-5-13-12)2-1-3-10-14-15(8)10/h1-6,13H/b3-1-,7-2-,12-11-
s12-34	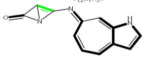 $C_{12}H_7N_3O$	26.1805	285.8108	-17.4528	90.2972	396.7302	InChI=1S/C12H7N3O/c16-12-10-11(15(10)12)14-8-3-1-2-7-4-5-13-9(7)6-8/h1-6,13H/b14-8+
H1-15	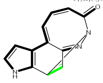 $C_{12}H_7N_3O$	27.2143	146.5195	-0.7758	155.923	396.9648	InChI=1S/C12H7N3O/c16-11-3-1-2-7-8-4-5-13-12(8)10-6-9(7)14-15(10)11/h1-6,13H/b3-1-,7-2-
E-13	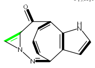 $C_{12}H_7N_3O$	5.5988	240.6331	-3.0768	126.0427	396.9994	InChI=1S/C12H7N3O/c16-12-8-2-1-3-9(14-15-6-10(12)15)7-4-5-13-11(7)8/h1-6,13H/b14-9+
X-24	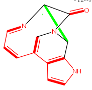 $C_{12}H_7N_3O$	35.1853	107.5470	-4.9518	167.0259	397.3322	InChI=1S/C12H7N3O/c16-12-10-11-9-8(3-5-14-9)7(6-15(11)12)2-1-4-13-10/h1-6,14H/b2-1-,13-4+
p1-22	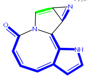 $C_{12}H_7N_3O$	10.2506	223.494	-3.7893	156.0392	397.3872	InChI=1S/C12H7N3O/c16-9-3-1-2-7-4-5-13-10(7)12-11-8(14-11)6-15(9)12/h1-6,13H/b3-1-,7-2-,12-10+
H11-143	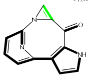 $C_{12}H_7N_3O$	21.4571	280.5736	-8.6731	62.0497	397.5504	InChI=1S/C12H7N3O/c16-12-9-6-15(9)10-3-1-2-8(14-10)7-4-5-13-11(7)12/h1-6,13H
o-175	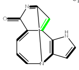 $C_{12}H_7N_3O$	8.7028	91.2252	-2.8035	251.6476	397.862	InChI=1S/C12H7N3O/c16-12-7-2-1-5-15-9-3-4-13-11(9)8(7)6-10(15)14-12/h1-6,13H/b5-1-,7-2+
A-38	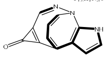 $C_{12}H_7N_3O$	43.1149	213.7075	-19.9262	125.5195	398.287	InChI=1S/C12H7N3O/c16-11-9-6-14-15-5-1-2-7(10(9)11)8-3-4-13-12(8)15/h1-6,13H/b14-6-
A-140	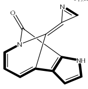 $C_{12}H_7N_3O$	31.4001	247.7778	-1.9229	117.2207	398.667	InChI=1S/C12H7N3O/c16-12-10-7(3-4-13-10)8-2-1-5-15(12)11(8)9-6-14-9/h1-6,13H/b11-9-
X-28	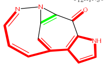 $C_{12}H_7N_3O$	16.8013	264.5596	-6.1221	68.0765	398.7473	InChI=1S/C12H7N3O/c16-12-10-8(3-5-13-10)7-2-1-4-14-15-9(6-7)11(12)15/h1-6,13H/b2-1-,14-4-

o-226	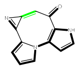 $C_{12}H_7N_3O$	16.9773	255.9126	-6.8639	103.0625	398.8468	InChI=1S/C12H7N3O/c16-10-6-7-11(14-7)8-2-1-5-15(8)9-3-4-13-12(9)10/h1-6,13H/b7-6+
o-55	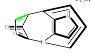 $C_{12}H_7N_3O$	9.7556	94.0681	-3.3881	248.7674	398.9458	InChI=1S/C12H7N3O/c16-11-8-6-14-12(11)15-5-1-2-7(8)10-9(15)3-4-13-10/h1-6,13H
A2-14	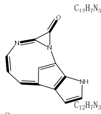 $C_{12}H_7N_3O$	6.362	316.5023	-4.4833	69.0862	399.0445	InChI=1S/C12H7N3O/c16-12-11-14-4-1-2-7-6-9(15(11)12)10-8(7)3-5-13-10/h1-6,13H/b4-1-,7-2+,14-11-
n-157	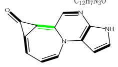 $C_{12}H_7N_3O$	10.4864	227.1501	-4.5453	140.1875	399.4082	InChI=1S/C12H7N3O/c16-11-7-2-1-5-15-8-3-4-13-12(8)14-6-9(15)10(7)11/h1-6,13H
B-173	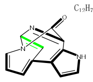 $C_{12}H_7N_3O$	10.6494	166.9557	-3.2741	182.3572	399.6050	InChI=1S/C12H7N3O/c16-12-11-10-8(3-4-13-10)7-2-1-5-15(12)9(6-7)14-11/h1-6,13H
B-2	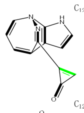 $C_{12}H_7N_3O$	40.7988	199.3532	-18.4767	145.0187	399.6152	InChI=1S/C12H7N3O/c16-10-6-9(10)11-7-2-1-5-15(14-11)12-8(7)3-4-13-12/h1-6,13H
A2-1	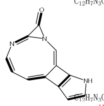 $C_{12}H_7N_3O$	4.7056	375.359	-1.2214	8.3403	399.7589	InChI=1S/C12H7N3O/c16-12-11-14-4-1-2-7-8-3-5-13-10(8)9(7)6-15(11)12/h1-6,13H/b4-1-,7-2-,9-6+,14-11-
x3-20	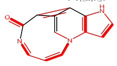 $C_{12}H_7N_3O$	22.6505	193.1365	-10.1245	120.0192	400.0334	InChI=1S/C12H7N3O/c16-12-10-7-6-15(5-1-3-14-12)8-2-4-13-11(8)9(7)10/h1-6,13H/b5-1-,14-3-
o-240	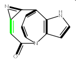 $C_{12}H_7N_3O$	13.869	213.3357	-5.2186	155.2466	400.0802	InChI=1S/C12H7N3O/c16-10-6-8-11(14-8)7-2-1-5-15(10)9-3-4-13-12(7)9/h1-6,13H/b8-6+
A-90	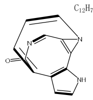 $C_{12}H_7N_3O$	5.8297	259.6163	-3.1021	101.7362	400.2021	InChI=1S/C12H7N3O/c16-12-8-2-1-5-15-9(6-14-12)11(15)10-7(8)3-4-13-10/h1-6,13H/b5-1-,8-2-,14-6-
A-147	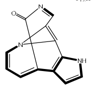 $C_{12}H_7N_3O$	5.8288	259.6198	-3.1013	101.7392	400.2021	InChI=1S/C12H7N3O/c16-12-8-2-1-5-15-9(6-14-12)11(15)10-7(8)3-4-13-10/h1-6,13H/b5-1-,8-2-,14-6-
q-37	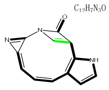 $C_{12}H_7N_3O$	21.0764	264.5947	-12.3504	104.5835	400.2749	InChI=1S/C12H7N3O/c16-12-8-6-15(12)11-9(14-11)3-1-2-7-4-5-13-10(7)8/h1-6,13H/b2-1-,9-3+
B-214	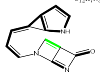 $C_{12}H_7N_3O$	20.9788	223.1406	-13.3699	150.8684	400.3782	InChI=1S/C12H7N3O/c16-12-9-6-15(11(9)14-12)5-1-2-7-8-3-4-13-10(7)8/h1-6,13H/b5-1-,7-2-
B-73	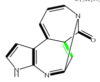 $C_{12}H_7N_3O$	8.5003	110.7119	-4.2030	240.9151	400.7151	InChI=1S/C12H7N3O/c16-12-9-6-10-14-11-8(3-4-13-11)7(9)2-1-5-15(10)12/h1-6,13H
o-143	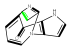 $C_{12}H_7N_3O$	13.1384	78.941	-4.0751	254.6704	401.012	InChI=1S/C12H7N3O/c16-12-7-2-1-5-15(10-6-8(7)14-10)9-3-4-13-11(9)12/h1-6,13H/b5-1-,7-2-
q11-31	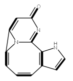 $C_{12}H_7N_3O$	15.7286	222.1347	-6.5177	146.3709	401.1255	InChI=1S/C12H7N3O/c16-10-6-9-8-3-1-2-7-4-5-13-11(7)12(14-10)15(8)9/h1-6,13H/b2-1-,8-3-
p1-34	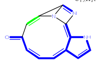 $C_{12}H_7N_3O$	14.7745	192.8863	-9.7113	179.2925	401.141	InChI=1S/C12H7N3O/c16-8-3-1-2-7-4-5-13-10(7)12-14-11-9(6-8)15(11)12/h1-6,13H/b3-1-,7-2-,9-6-,12-10+
q11-9	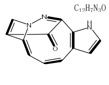 $C_{12}H_7N_3O$	10.6633	99.1836	-6.2713	247.1434	401.2129	InChI=1S/C12H7N3O/c16-12-8-6-15-9(8)3-1-2-7-4-5-13-10(7)11(12)14-15/h1-6,13H/b2-1-,9-3-
A-186	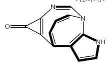 $C_{12}H_7N_3O$	32.7492	215.2297	-12.1799	137.6089	401.7052	InChI=1S/C12H7N3O/c16-11-9-7-2-1-5-15(6-14-10(9)11)12-8(7)3-4-13-12/h1-6,13H/b14-6-

q1-35	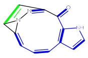	$C_{12}H_7N_3O$	17.7246	182.1614	-8.0541	186.4422	401.7966	InChI=1S/C12H7N3O/c16-12-8-6-10-9(15(10)14-8)3-1-2-7-4-5-13-11(7)12/h1-6,13H/b2-1-,9-3-
X22-31	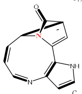	$C_{12}H_7N_3O$	15.8218	187.3031	-9.6674	181.7438	401.7984	InChI=1S/C12H7N3O/c16-12-7-6-10-11-8(3-5-14-11)13-4-1-2-9(7)15(10)12/h1-6,14H/b2-1-,4-1-,9-2-,11-10+,13-4-,13-8-
s11-11	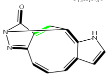	$C_{12}H_7N_3O$	30.6095	88.8487	-2.4147	212.229	401.9704	InChI=1S/C12H7N3O/c16-12-8-6-10-11-7(4-5-13-11)2-1-3-9(8)14-15(10)12/h1-6,13H/b3-1-,7-2-,11-10+
H-69	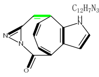	$C_{12}H_7N_3O$	24.304	236.4696	-8.4314	122.4646	402.1769	InChI=1S/C12H7N3O/c16-12-9-3-1-2-7(6-10-14-15(10)12)11-8(9)4-5-13-11/h1-6,13H/b7-6+
H-14	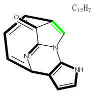	$C_{12}H_7N_3O$	9.689	163.971	-2.3686	186.9409	402.3238	InChI=1S/C12H7N3O/c16-10-7-2-1-3-9-8-4-5-13-11(8)15(6-7)12(10)14-9/h1-6,13H/b2-1-,9-3-
p1-38	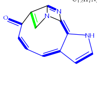	$C_{12}H_7N_3O$	9.0938	87.4765	-4.5203	274.5111	402.3338	InChI=1S/C12H7N3O/c16-9-3-1-2-7-4-5-13-10(7)12-14-11-8(9)6-15(11)12/h1-6,13H/b3-1-,7-2-,12-10+
j-11	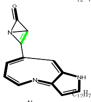	$C_{12}H_7N_3O$	29.1422	296.5759	-16.9884	70.4396	402.6073	InChI=1S/C12H7N3O/c16-12-11-10(15(11)12)7-2-1-4-13-8-3-5-14-9(8)6-7/h1-6,14H/b2-1-,4-1-,7-2+,7-6+,9-6+,13-4+,13-8+
H-144	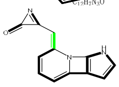	$C_{12}H_7N_3O$	4.2011	335.5062	-3.6905	56.6177	402.9528	InChI=1S/C12H7N3O/c16-12-9(14-12)6-7-2-1-3-10-8-4-5-13-11(8)15(7)10/h1-6,13H/b7-6+
B-72	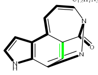	$C_{12}H_7N_3O$	9.5468	92.1662	-3.2993	248.6108	403.0349	InChI=1S/C12H7N3O/c16-12-9-6-10-11-8(3-4-13-11)7(9)2-1-5-15(12)14-10/h1-6,13H
H11-146	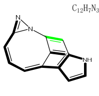	$C_{12}H_7N_3O$	26.5225	181.7576	-2.6243	175.1671	403.4782	InChI=1S/C12H7N3O/c16-12-8-2-1-3-11-14-15(11)10(12)6-9-7(8)4-5-13-9/h1-6,13H/b3-1-,8-2-
n-180	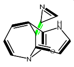	$C_{12}H_7N_3O$	3.943	308.6081	-3.0076	90.1258	404.1097	InChI=1S/C12H7N3O/c16-12-10(8-6-14-8)7-2-1-5-15(12)9-3-4-13-11(7)9/h1-6,13H/b10-8+
H1-124	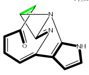	$C_{12}H_7N_3O$	14.404	217.317	-4.5402	157.8046	404.278	InChI=1S/C12H7N3O/c16-7-2-1-3-9-8-4-5-13-11(8)15-10(6-7)12(15)14-9/h1-6,13H/b2-1-,9-3-,10-6-
r-30	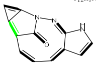	$C_{12}H_7N_3O$	28.0444	257.366	-16.1202	103.6407	404.6738	InChI=1S/C12H7N3O/c16-12-8-3-1-2-7-4-5-13-11(7)14-15(12)10-6-9(8)10/h1-6H,(H,13,14)/b3-1-,7-2-
X-47	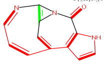	$C_{12}H_7N_3O$	26.8267	301.7881	-10.1582	38.2390	404.7158	InChI=1S/C12H7N3O/c16-12-10-8(3-5-13-10)7-2-1-4-14-11-9(6-7)15(11)12/h1-6,13H/b2-1-,4-1-,7-2+,14-4-,14-11+
E-18	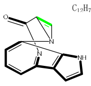	$C_{12}H_7N_3O$	17.1108	188.7346	-11.7536	171.9912	405.0279	InChI=1S/C12H7N3O/c16-12-10-6-15(10)9-3-1-2-8(14-12)7-4-5-13-11(7)9/h1-6,13H/b14-8+
r1-26	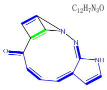	$C_{12}H_7N_3O$	14.4322	206.558	-10.0021	172.1751	405.0906	InChI=1S/C12H7N3O/c16-10-3-1-2-7-4-5-13-12(7)14-15-9-6-8(10)11(9)15/h1-6H,(H,13,14)/b3-1-,7-2-
B-90	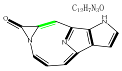	$C_{12}H_7N_3O$	8.6524	383.4929	-1.3744	-1.2400	405.1161	InChI=1S/C12H7N3O/c16-12-10-6-9-11-7(3-4-13-11)8(14-9)2-1-5-15(10)12/h1-6,13H/b5-1-,8-2-,10-6-
H1-62	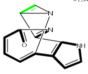	$C_{12}H_7N_3O$	37.4178	242.0817	-19.5418	101.3663	405.1623	InChI=1S/C12H7N3O/c16-10-3-1-2-8-7-4-5-13-11(7)12(14-10)9-6-15(8)9/h1-6,13H/b3-1-,8-2-,14-12+
s11-27	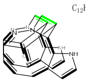	$C_{12}H_7N_3O$	13.0446	125.5835	-4.4157	209.2359	405.399	InChI=1S/C12H7N3O/c16-12-8-6-15-11(8)10-7(4-5-13-10)2-1-3-9(12)14-15/h1-6,13H/b3-1-,7-2-,11-10+

p1-57	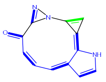 $C_{12}H_7N_3O$	45.0512	265.1361	-24.3066	74.1772	405.5603	InChI=1S/C12H7N3O/c16-10-3-1-2-7-4-5-13-11(7)8-6-9(8)15-12(10)14-15/h1-6,13H/b3-1-,7-2-,11-8+
B-156	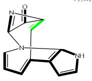 $C_{12}H_7N_3O$	11.1099	134.2884	-4.3342	217.2857	405.5885	InChI=1S/C12H7N3O/c16-10-9-6-7-2-1-5-15(12(10)14-9)11-8(7)3-4-13-11/h1-6,13H/b9-6-
n-173	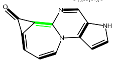 $C_{12}H_7N_3O$	10.528	233.6527	-4.4348	142.5101	405.6015	InChI=1S/C12H7N3O/c16-11-7-2-1-5-15-9-3-4-13-8(9)6-14-12(15)10(7)11/h1-6,13H
p1-7	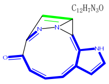 $C_{12}H_7N_3O$	18.6708	182.993	-8.3884	187.611	406.065	InChI=1S/C12H7N3O/c16-10-3-1-2-7-4-5-13-11(7)12-9-6-8(10)14-15(9)12/h1-6,13H/b3-1-,7-2-,12-11+
G1-93	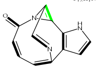 $C_{12}H_7N_3O$	25.0414	260.1715	-10.2187	86.8008	406.0666	InChI=1S/C12H7N3O/c16-10-3-1-2-8-7-4-5-13-11(7)12-9(6-14-8)15(10)12/h1-6,13H/b3-1-,8-2-
B-101	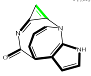 $C_{12}H_7N_3O$	14.5919	224.5256	-10.4700	126.4957	406.2290	InChI=1S/C12H7N3O/c16-12-8-2-1-5-15(10-6-9(10)14-12)11-7(8)3-4-13-11/h1-6,13H/b14-9-
H11-72	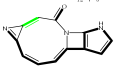 $C_{12}H_7N_3O$	19.1255	297.9108	-7.2137	95.8822	406.347	InChI=1S/C12H7N3O/c16-11-6-9-8(14-9)2-1-3-10-7-4-5-13-12(7)15(10)11/h1-6,13H/b2-1-,9-6+,10-3-
E-47	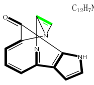 $C_{12}H_7N_3O$	17.9316	205.7924	-11.9457	142.5112	406.6189	InChI=1S/C12H7N3O/c16-12-9-3-1-2-8(14-10-6-15(9)10)7-4-5-13-11(7)12/h1-6,13H/b2-1-,9-3-,14-8?
o-2	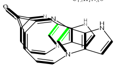 $C_{12}H_7N_3O$	3.91	332.5461	-0.8276	40.7799	406.7397	InChI=1S/C12H7N3O/c16-12-7-2-1-5-15-6-8(14-10(7)12)11-9(15)3-4-13-11/h1-6,13H/b5-1-,7-2+,14-10+
k-37	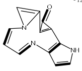 $C_{12}H_7N_3O$	75.7039	298.6089	-44.7342	46.0788	406.7574	InChI=1S/C12H7N3O/c16-12-9-8-6-15(8)5-1-3-13-7-2-4-14-11(7)10(9)12/h1-6,14H/b5-1-,13-3+
F-40	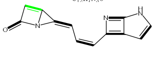 $C_{12}H_7N_3O$	6.0562	422.5676	-4.548	-7.09	407.7498	InChI=1S/C12H7N3O/c16-11-6-10-9(15(10)11)3-1-2-8-7-4-5-13-12(7)14-8/h1-6H,(H,13,14)/b2-1-,9-3-
o-24	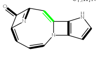 $C_{12}H_7N_3O$	16.3298	189.4401	-4.477	165.9804	407.9987	InChI=1S/C12H7N3O/c16-12-7-2-1-5-15-9-3-4-13-11(9)10(15)6-8(12)14-7/h1-6,13H/b5-1-,7-2-,10-6-
X-36	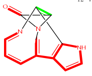 $C_{12}H_7N_3O$	22.4627	218.3116	-6.8948	154.3305	408.0401	InChI=1S/C12H7N3O/c16-12-11-10-9-8(3-5-14-9)7(6-15(11)12)2-1-4-13-10/h1-6,14H/b2-1-,13-4+
p1-61	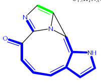 $C_{12}H_7N_3O$	11.7442	205.621	-5.6811	181.2857	408.2126	InChI=1S/C12H7N3O/c16-9-3-1-2-7-4-5-13-10(7)11-8-6-14-12(9)15(8)11/h1-6,13H/b3-1-,7-2-,11-10+
A2-17	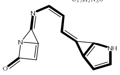 $C_{12}H_7N_3O$	28.1088	300.1096	-18.1858	90.5473	408.3433	InChI=1S/C12H7N3O/c16-10-6-9-12(15(9)10)14-4-1-2-7-8-3-5-13-11(7)8/h1-6,13H/b4-1-,7-2-,14-12+
A2-9	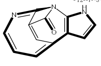 $C_{12}H_7N_3O$	38.5085	148.005	-2.2015	132.8147	408.6141	InChI=1S/C12H7N3O/c16-12-9-6-7-2-1-4-13-11(9)15(12)10-8(7)3-5-14-10/h1-6,14H/b2-1-,4-1-,7-2+,13-4-,13-11+
x4-23	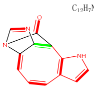 $C_{12}H_7N_3O$	14.3688	131.3423	-5.2323	235.0337	408.8757	InChI=1S/C12H7N3O/c16-12-9-10-7(4-5-13-10)2-1-3-8-11(9)14-6-15(8)12/h1-6,13H/b2-1-,3-1-,7-2-,8-3+,10-9-
q11-58	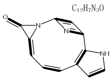 $C_{12}H_7N_3O$	10.8047	321.1137	-8.1516	71.1154	409.0259	InChI=1S/C12H7N3O/c16-12-9-3-1-2-7-4-5-13-11(7)8-6-10(14-8)15(9)12/h1-6,13H/b2-1-,9-3-
C-29	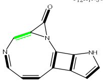 $C_{12}H_7N_3O$	4.6772	409.1886	-1.3878	-6.9000	409.0847	InChI=1S/C12H7N3O/c16-12-9-6-13-4-1-2-8-7-3-5-14-10(7)11(8)15(9)12/h1-6,14H/b2-1-,9-6-,13-4-

H11-29	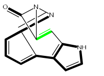	15.509	360.778	-6.23	13.5573	409.2413	InChI=1S/C12H7N3O/c16-12-11-6-10-8(4-5-13-10)7-2-1-3-9(7)14-15(11)12/h1-6,13H/b11-6+,14-9+
H1-96	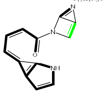	35.5576	285.8959	-18.4848	107.6659	409.6079	InChI=1S/C12H7N3O/c16-10(15-6-9-12(15)14-9)3-1-2-7-8-4-5-13-11(7)8/h1-6,13H/b3-1-,7-2-
X22-10	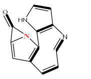	14.3582	268.9758	-6.302	121.8439	409.9068	InChI=1S/C12H7N3O/c16-12-9-6-7-2-1-4-13-8-3-5-14-10(8)11(7)15(9)12/h1-6,14H/b2-1-,4-1+,7-2-,11-10-,13-4+,13-8-
B-168	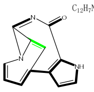	22.2631	202.4280	-10.5421	145.2049	409.9229	InChI=1S/C12H7N3O/c16-12-10-8(3-4-13-10)7-2-1-5-15-9(6-7)11(15)14-12/h1-6,13H/b14-11+
B-127	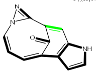	18.5362	245.7404	-0.7296	120.2535	409.9749	InChI=1S/C12H7N3O/c16-11-8-2-1-5-15-12(14-15)9(11)6-10-7(8)3-4-13-10/h1-6,13H/b5-1-,8-2-
B-27	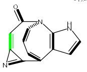	10.3319	290.0698	-3.9610	102.4724	410.0598	InChI=1S/C12H7N3O/c16-10-6-9-11(14-9)7-2-1-5-15(10)12-8(7)3-4-13-12/h1-6,13H/b9-6+
n-47	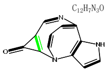	29.9418	236.7207	-11.1637	128.1542	410.266	InChI=1S/C12H7N3O/c16-12-7-6-14-8-2-1-5-15(11(7)12)9-3-4-13-10(8)9/h1-6,13H/b14-6-
X22-11	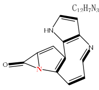	15.0521	267.8199	-6.6042	125.823	410.6209	InChI=1S/C12H7N3O/c16-12-10-6-7-9(15(10)12)2-1-4-13-8-3-5-14-11(7)8/h1-6,14H/b2-1-,4-1+,9-2+,11-7+,13-4+,13-8-
F-24	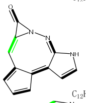	14.402	289.6439	-6.0627	83.2499	411.245	InChI=1S/C12H7N3O/c16-12-10-6-7-2-1-3-8(7)9-4-5-13-11(9)14-15(10)12/h1-6H,(H,13,14)/b9-8-,10-6-
B-189	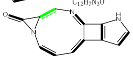	9.8923	343.2398	-5.6494	54.1731	411.4031	InChI=1S/C12H7N3O/c16-12-9-6-14-11-7(2-1-5-15(9)12)8-3-4-13-10(8)11/h1-6,13H/b5-1-,7-2-,9-6-,14-11+
q1-28	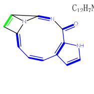	25.7905	237.6206	-13.3513	142.7206	411.4976	InChI=1S/C12H7N3O/c16-12-10-7(4-5-13-10)2-1-3-8-6-9-11(14-12)15(8)9/h1-6,13H/b2-1-,8-3-,14-11-
A1-46	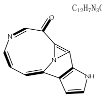	15.0399	317.2185	-6.9849	53.8231	411.5688	InChI=1S/C12H7N3O/c16-9-6-13-4-1-2-8-7-3-5-14-10(7)12-11(9)15(8)12/h1-6,14H/b4-1-,8-2-,13-6-
H1-83	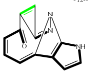	5.4295	378.3863	-1.4205	15.99	411.7236	InChI=1S/C12H7N3O/c16-11-3-1-2-10-7-4-5-13-12(7)15(10)14-9-6-8(9)11/h1-6,13H/b3-1-,10-2-,14-9+
A2-49	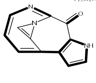	34.0664	259.5813	-18.142	72.4809	411.9961	InChI=1S/C12H7N3O/c16-11-10-8(3-5-13-10)7-2-1-4-14-12(11)15-6-9(7)15/h1-6,13H/b4-1-,7-2-,14-12-
s11-36	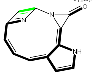	23.1218	261.8095	-7.5335	119.7223	412.2107	InChI=1S/C12H7N3O/c16-12-11-10-7(4-5-13-10)2-1-3-8-6-9(14-8)15(11)12/h1-6,13H/b3-1-,7-2-,11-10+
G-26	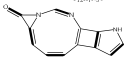	9.0995	343.0371	-5.665	55.9812	412.3139	InChI=1S/C12H7N3O/c16-12-9-3-1-2-7-8-4-5-13-10(8)11(7)14-6-15(9)12/h1-6,13H/b2-1-,9-3-,14-6-
s11-65	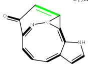	21.6676	206.8672	-8.0977	161.038	412.5423	InChI=1S/C12H7N3O/c16-10-6-9-12-11-7(4-5-13-11)2-1-3-8(10)14-15(9)12/h1-6,13H/b3-1-,7-2-,12-11+
q1-11	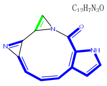	56.3338	297.5131	-35.4473	66.0221	412.5729	InChI=1S/C12H7N3O/c16-12-10-7(4-5-13-10)2-1-3-8-11(14-8)9-6-15(9)12/h1-6,13H/b2-1-,8-3+
X23-48	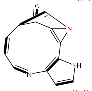	21.9523	201.5274	-11.6095	152.3051	412.8202	InChI=1S/C12H7N3O/c16-12-7-2-1-4-13-8-3-5-14-9(8)10-11(12)15(10)6-7/h1-6,14H/b2-1-,13-4-

o-38	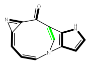 $C_{12}H_7N_3O$	5.2712	339.6806	-1.204	39.009	413.3015	InChI=1S/C12H7N3O/c16-12-7-6-15(9-3-4-13-10(7)9)5-1-2-8-11(12)14-8/h1-6,13H/b5-1-,8-2+
H11-103	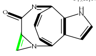 $C_{12}H_7N_3O$	11.6128	261.8223	-6.5209	96.4561	413.3017	InChI=1S/C12H7N3O/c16-12-10-6-15(10)9-3-1-2-8(14-12)11-7(9)4-5-13-11/h1-6,13H/b14-8+
E-34	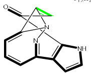 $C_{12}H_7N_3O$	9.7071	238.6297	-3.8805	147.2322	415.108	InChI=1S/C12H7N3O/c16-11-6-10-9-3-1-2-8(14-15(9)10)7-4-5-13-12(7)11/h1-6,13H/b10-6-
o-132	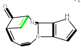 $C_{12}H_7N_3O$	44.9042	274.6467	-13.125	81.7624	415.1714	InChI=1S/C12H7N3O/c16-11-7-2-1-5-15-9-3-4-13-10(9)12(15)14-8(11)6-7/h1-6,13H/b5-1-,7-2+,14-12+
G-56	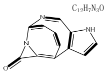 $C_{12}H_7N_3O$	10.8898	265.8503	-7.3089	122.2273	415.3505	InChI=1S/C12H7N3O/c16-12-11-8-2-1-3-10(15(11)12)14-6-9-7(8)4-5-13-9/h1-6,13H/b14-6-
q-2	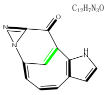 $C_{12}H_7N_3O$	34.9799	161.3317	-6.651	183.5588	415.8205	InChI=1S/C12H7N3O/c16-11-9-6-8(15-12(11)14-15)3-1-2-7-4-5-13-10(7)9/h1-6,13H/b2-1-,3-1-,7-2-,8-3+,10-9-
I-26	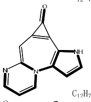 $C_{12}H_7N_3O$	39.4171	282.4875	-16.0584	77.2491	415.8239	InChI=1S/C12H7N3O/c16-12-7-6-9-13-3-1-5-15(9)8-2-4-14-11(8)10(7)12/h1-6,14H
A2-44	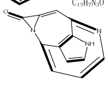 $C_{12}H_7N_3O$	26.6274	205.4175	-12.4222	174.5697	416.1063	InChI=1S/C12H7N3O/c16-12-10-6-8-11-7(3-5-14-11)9(15(10)12)2-1-4-13-8/h1-6,14H/b2-1-,4-1-,9-2+,13-4-,13-8-
r-27	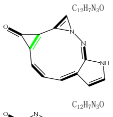 $C_{12}H_7N_3O$	47.3423	293.3871	-28.3765	69.6384	416.2701	InChI=1S/C12H7N3O/c16-11-8-3-1-2-7-4-5-13-12(7)14-15-6-9(15)10(8)11/h1-6H,(H,13,14)/b3-1-,7-2-
H-107	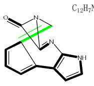 $C_{12}H_7N_3O$	11.3733	351.1073	-5.9723	40.0656	416.6119	InChI=1S/C12H7N3O/c16-12-11-14-10-9(4-5-13-10)8-3-1-2-7(8)6-15(11)12/h1-6,13H/b7-6+,14-11+
B-41	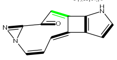 $C_{12}H_7N_3O$	3.5876	367.6267	-1.8386	37.5476	416.8128	InChI=1S/C12H7N3O/c16-10-6-9-7(8-3-4-13-11(8)9)2-1-5-15-12(10)14-15/h1-6,13H/b5-1-,7-2+,9-6+
B-121	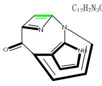 $C_{12}H_7N_3O$	10.1904	119.9854	-3.9390	245.2591	416.8571	InChI=1S/C12H7N3O/c16-11-7-2-1-5-15(10-6-9(11)14-10)12-8(7)3-4-13-12/h1-6,13H
D-21	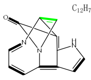 $C_{12}H_7N_3O$	37.6310	101.7245	-0.8324	204.4411	417.4198	InChI=1S/C12H7N3O/c16-12-10-8-6-15(12)9(2-1-4-13-8)7-3-5-14-11(7)10/h1-6,14H/b2-1-,13-4+
q1-53	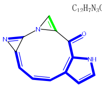 $C_{12}H_7N_3O$	43.142	272.9663	-29.0933	95.8204	417.4465	InChI=1S/C12H7N3O/c16-11-9-6-15(9)12-8(14-12)3-1-2-7-4-5-13-10(7)11/h1-6,13H/b2-1-,8-3+
n-108	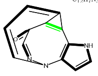 $C_{12}H_7N_3O$	13.797	225.0084	-5.7352	143.9402	417.8372	InChI=1S/C12H7N3O/c16-9-6-14-15-5-1-2-7-10(9)11(7)12-8(15)3-4-13-12/h1-6,13H/b5-1-,7-2+,14-6-
k-16	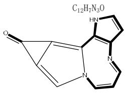 $C_{12}H_7N_3O$	14.2652	238.3662	-10.899	163.0535	417.8653	InChI=1S/C12H7N3O/c16-12-7-6-15-5-1-3-13-8-2-4-14-10(8)11(15)9(7)12/h1-6,14H/b5-1-,13-3+
H-28	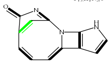 $C_{12}H_7N_3O$	10.6616	270.0842	-2.5097	107.9448	418.0164	InChI=1S/C12H7N3O/c16-12-7-2-1-3-9-8-4-5-13-11(8)15(9)10(6-7)14-12/h1-6,13H/b2-1-,9-3-
H1-116	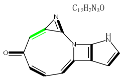 $C_{12}H_7N_3O$	6.8317	376.3778	-1.6488	18.8174	418.2076	InChI=1S/C12H7N3O/c16-7-2-1-3-10-8-4-5-13-11(8)15(10)12-9(6-7)14-12/h1-6,13H/b2-1-,9-6+,10-3-
p1-31	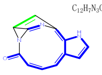 $C_{12}H_7N_3O$	13.186	256.0042	-6.3984	135.211	418.2742	InChI=1S/C12H7N3O/c16-10-3-1-2-7-4-5-13-11(7)8-6-9-12(14-8)15(9)10/h1-6,13H/b3-1-,7-2-,11-8+

H-18	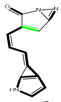 $C_{12}H_7N_3O$	33.1114	329.6061	-12.0708	62.797	418.4548	InChI=1S/C12H7N3O/c16-12-7(6-10-14-15(10)12)2-1-3-8-9-4-5-13-11(8)9/h1-6,13H/b2-1-,8-3-
r-29	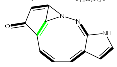 $C_{12}H_7N_3O$	8.1998	274.4753	-3.9283	118.0544	418.8747	InChI=1S/C12H7N3O/c16-10-6-9-11-8(10)3-1-2-7-4-5-13-12(7)14-15(9)11/h1-6H,(H,13,14)/b3-1-,7-2-
H1-109	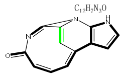 $C_{12}H_7N_3O$	13.707	256.0018	-4.1224	114.2555	419.1308	InChI=1S/C12H7N3O/c16-10-3-1-2-7-6-9-12(14-10)15(9)11-8(7)4-5-13-11/h1-6,13H/b3-1-,7-2+,14-12+
E-51	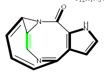 $C_{12}H_7N_3O$	12.9063	253.3421	-5.2919	139.6867	419.1834	InChI=1S/C12H7N3O/c16-12-11-7(4-5-13-11)8-2-1-3-9-10(6-14-8)15(9)12/h1-6,13H/b2-1-,3-1-,8-2-,9-3+,10-6-,14-6-,14-8-
A-192	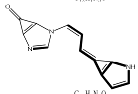 $C_{12}H_7N_3O$	46.4349	346.2874	-19.6216	38.4811	419.4754	InChI=1S/C12H7N3O/c16-12-10-11(12)15(6-14-10)5-1-2-7-8-3-4-13-9(7)8/h1-6,13H/b5-1-,7-2-
r-28	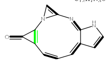 $C_{12}H_7N_3O$	39.6345	299.5055	-22.6694	69.1183	419.5641	InChI=1S/C12H7N3O/c16-11-8-3-1-2-7-4-5-13-12(7)14-9-6-15(9)10(8)11/h1-6H,(H,13,14)/b3-1-,7-2-
A2-67	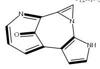 $C_{12}H_7N_3O$	12.8826	287.9336	-4.6364	71.4206	419.8102	InChI=1S/C12H7N3O/c16-11-7-2-1-4-13-10(11)9-6-15(9)12-8(7)3-5-14-12/h1-6,14H
B-131	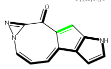 $C_{12}H_7N_3O$	9.4541	288.9039	-4.9602	106.8930	420.6262	InChI=1S/C12H7N3O/c16-11-9-6-10-8(3-4-13-10)7(9)2-1-5-15-12(11)14-15/h1-6,13H/b5-1-,7-2-
o-74	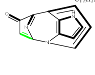 $C_{12}H_7N_3O$	10.9704	113.7608	-3.1505	247.3011	421.4933	InChI=1S/C12H7N3O/c16-9-6-10-14-12(9)7-2-1-5-15(10)8-3-4-13-11(7)8/h1-6,13H
p1-48	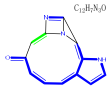 $C_{12}H_7N_3O$	14.6902	191.0045	-9.9452	210.7976	421.4943	InChI=1S/C12H7N3O/c16-8-3-1-2-7-4-5-13-10(7)11-12-14-9(6-8)15(11)12/h1-6,13H/b3-1-,7-2-,9-6-,11-10+
x5-9	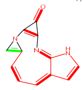 $C_{12}H_7N_3O$	42.5105	330.1977	-19.7335	38.7267	421.7303	InChI=1S/C12H7N3O/c16-11-9-10(11)15-6-8(15)3-1-2-7-4-5-13-12(7)14-9/h1-6H,(H,13,14)/b3-1-,7-2-
I-93	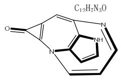 $C_{12}H_7N_3O$	28.5182	244.978	-10.9357	138.0366	421.8263	InChI=1S/C12H7N3O/c16-12-7-6-8-10-9(2-4-14-10)15(11(7)12)5-1-3-13-8/h1-6,14H/b5-1-,13-3-
H-61	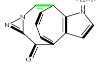 $C_{12}H_7N_3O$	28.2133	156.5812	-6.5905	207.4458	422.0654	InChI=1S/C12H7N3O/c16-11-9-3-1-2-7(6-15-12(11)14-15)10-8(9)4-5-13-10/h1-6,13H/b7-6+
q-27	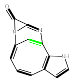 $C_{12}H_7N_3O$	9.8748	334.6597	-4.9325	65.7759	422.1236	InChI=1S/C12H7N3O/c16-12-11-14-9-6-8(15(11)12)3-1-2-7-4-5-13-10(7)9/h1-6,13H/b2-1-,3-1-,7-2-,8-3+,10-9-
n-128	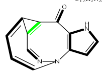 $C_{12}H_7N_3O$	14.8398	208.2431	-6.2049	159.3172	422.4126	InChI=1S/C12H7N3O/c16-12-10-7-2-1-5-15(14-6-8(7)10)9-3-4-13-11(9)12/h1-6,13H/b5-1-,7-2+,14-6-
B-170	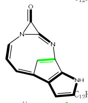 $C_{12}H_7N_3O$	7.0239	368.7743	-1.7311	34.8647	422.4965	InChI=1S/C12H7N3O/c16-12-11-14-9-6-7(2-1-5-15(11)12)8-3-4-13-10(8)9/h1-6,13H/b5-1-,7-2+,14-11-
p1-27	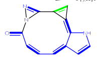 $C_{12}H_7N_3O$	47.4611	272.479	-23.0086	95.8691	422.608	InChI=1S/C12H7N3O/c16-10-3-1-2-7-4-5-13-11(7)8-6-9(8)12-14-15(10)12/h1-6,13H/b3-1-,7-2-,11-8+
o-216	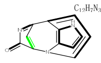 $C_{12}H_7N_3O$	11.1202	113.3175	-3.2751	245.6974	422.8723	InChI=1S/C12H7N3O/c16-12-10-6-8(14-12)7-2-1-5-15(10)9-3-4-13-11(7)9/h1-6,13H
H1-49	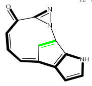 $C_{12}H_7N_3O$	6.2419	364.8261	-0.7995	27.2967	422.9695	InChI=1S/C12H7N3O/c16-10-3-1-2-7-6-9(15-12(10)14-15)11-8(7)4-5-13-11/h1-6,13H/b3-1-,7-2+

B-126	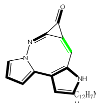	17.1357	353.5146	-5.4672	24.8415	423.0857	InChI=1S/C12H7N3O/c16-12-8-6-9-7(3-4-13-9)10-2-1-5-15(10)14-11(8)12/h1-6,13H/b8-6+,14-11+
A1-26	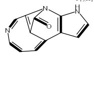	14.2232	227.3943	-5.835	147.4151	423.1151	InChI=1S/C12H7N3O/c16-12-10-7-2-1-4-13-6-9(10)15(12)11-8(7)3-5-14-11/h1-6,14H/b2-1-,4-1-,7-2-,9-6+,13-4-,13-6-
I-72	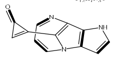	41.0449	200.2737	-18.5419	173.8625	423.2859	InChI=1S/C12H7N3O/c16-9-6-7(9)12-11-10-8(2-4-14-10)15(12)5-1-3-13-11/h1-6,14H
H11-17	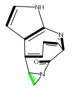	20.0378	223.1456	-12.7431	145.1855	423.4554	InChI=1S/C12H7N3O/c16-12-9-3-1-2-7(10-6-15(10)12)8-4-5-13-11(8)14-9/h1-6H,(H,13,14)
x5-12	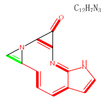	41.6476	327.6544	-19.36	43.1568	423.6578	InChI=1S/C12H7N3O/c16-11-9-10(11)15-6-8(15)3-1-2-7-4-5-13-12(7)14-9/h1-6H,(H,13,14)/b3-1+,7-2-
H-59	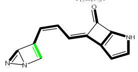	17.6614	301.1117	-10.083	107.3286	423.7168	InChI=1S/C12H7N3O/c16-11-9(8-4-5-13-10(8)11)3-1-2-7-6-15-12(7)14-15/h1-6,13H/b2-1-,9-3-
H1-121	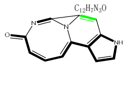	24.7106	186.2007	-15.7712	189.3037	423.8207	InChI=1S/C12H7N3O/c16-11-3-1-2-9-7-4-5-13-8(7)6-10-12(14-11)15(9)10/h1-6,13H/b3-1-,9-2-,14-12-
H11-100	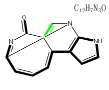	11.3107	89.7304	-3.9303	268.9902	424.0463	InChI=1S/C12H7N3O/c16-12-9-6-15-10(14-12)3-1-2-7(9)8-4-5-13-11(8)15/h1-6,13H/b3-1-,7-2-
p1-52	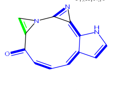	46.3614	261.1107	-26.5075	112.242	424.0732	InChI=1S/C12H7N3O/c16-9-3-1-2-7-4-5-13-10(7)11-12(14-11)15-6-8(9)15/h1-6,13H/b3-1-,7-2-,11-10-
H-33	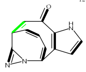	42.4539	167.6417	-5.5452	179.3323	424.5622	InChI=1S/C12H7N3O/c16-10-6-7-2-1-3-9(15-12(7)14-15)8-4-5-13-11(8)10/h1-6,13H/b7-6+
H-70	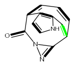	24.6988	242.0923	-8.7705	140.7063	425.0937	InChI=1S/C12H7N3O/c16-12-9-3-1-2-7(11-14-15(11)12)6-10-8(9)4-5-13-10/h1-6,13H/b2-1-,3-1-,7-2+,7-6-,9-3+,9-8+,10-6-
o-181	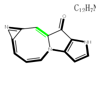	17.895	296.2275	-0.7618	95.5488	425.3649	InChI=1S/C12H7N3O/c16-12-10-6-8-7(14-8)2-1-5-15(10)9-3-4-13-11(9)12/h1-6,13H/b5-1-,7-2+,10-6-
H1-136	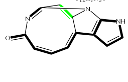	13.386	237.6454	-3.4279	130.4353	425.7003	InChI=1S/C12H7N3O/c16-11-3-1-2-7-8-4-5-13-12(8)15-9(7)6-10(15)14-11/h1-6,13H/b3-1-,7-2-,14-10-
H-62	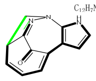	18.7494	113.1154	0.4264	223.0127	425.7237	InChI=1S/C12H7N3O/c16-11-8-3-1-2-7-6-15(14-10(7)11)12-9(8)4-5-13-12/h1-6,13H
n-138	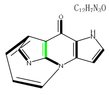	11.3296	154.2074	-3.516	211.4942	425.7329	InChI=1S/C12H7N3O/c16-11-9-7-2-1-5-15(12(9)14-6-7)8-3-4-13-10(8)11/h1-6,13H
H-45	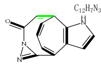	24.7334	210.4218	-5.4986	175.0062	425.7541	InChI=1S/C12H7N3O/c16-10-6-7-2-1-3-9(12-14-15(10)12)8-4-5-13-11(7)8/h1-6,13H/b7-6+
E-59	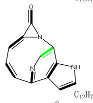	6.048	288.3555	-4.033	117.2931	426.4338	InChI=1S/C12H7N3O/c16-12-9-3-1-2-8-7-4-5-13-11(7)10(6-14-8)15(9)12/h1-6,13H/b2-1-,9-3-
q-39	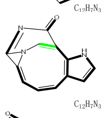	9.7403	157.7733	-4.9035	220.9339	426.5751	InChI=1S/C12H7N3O/c16-12-8-6-15-9(11(15)14-12)3-1-2-7-4-5-13-10(7)8/h1-6,13H/b2-1-,9-3-
s12-37	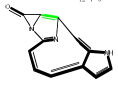	19.6614	261.1851	-7.5654	122.4141	426.9298	InChI=1S/C12H7N3O/c16-12-11-9-6-8-7(4-5-13-8)2-1-3-10(14-9)15(11)12/h1-6,13H/b3-1-,7-2-,8-6-

x5-11	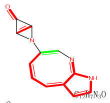	58.9177	267.1308	-33.5843	88.5578	427.1074	InChI=1S/C12H7N3O/c16-11-9-10(11)15(9)8-3-1-2-7-4-5-13-12(7)14-6-8/h1-6H,(H,13,14)
n-37	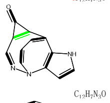	43.1635	216.9249	-19.7439	148.2142	427.1236	InChI=1S/C12H7N3O/c16-12-8-6-14-15-5-1-2-7(10(8)12)11-9(15)3-4-13-11/h1-6,13H/b14-6-
o-93	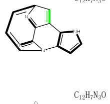	8.655	171.2507	-2.4238	200.6392	427.4907	InChI=1S/C12H7N3O/c16-12-11-8-6-7(14-11)2-1-5-15(12)9-3-4-13-10(8)9/h1-6,13H/b5-1-,7-2-
o-39	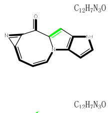	16.8175	284.8075	-1.4015	107.8789	427.9361	InChI=1S/C12H7N3O/c16-12-10-6-8-9(3-4-13-8)15(10)5-1-2-7-11(12)14-7/h1-6,13H/b5-1-,7-2+
H1-115	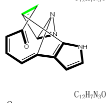	23.7915	208.9441	-8.9835	169.2083	428.5144	InChI=1S/C12H7N3O/c16-7-2-1-3-10-8-4-5-13-11(8)9(6-7)12-14-15(10)12/h1-6,13H/b2-1-,9-6+,10-3-
n-55	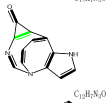	35.6201	233.9994	-15.4841	139.215	428.8988	InChI=1S/C12H7N3O/c16-12-9-7-2-1-5-15(6-14-11(9)12)8-3-4-13-10(7)8/h1-6,13H/b14-6-
E-39	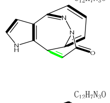	18.7952	181.6983	-4.7446	176.4615	429.4183	InChI=1S/C12H7N3O/c16-11-6-8-10-3-1-2-9(14-15(10)11)7-4-5-13-12(7)8/h1-6,13H
B-144	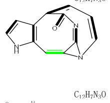	17.5118	239.9575	-6.1261	139.6164	429.5412	InChI=1S/C12H7N3O/c16-12-8-2-1-5-15-10(11(15)14-12)6-9-7(8)3-4-13-9/h1-6,13H/b5-1-,8-2-,10-6+,14-11+
o-244	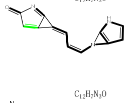	24.0463	285.6107	-13.8808	109.8294	429.6235	InChI=1S/C12H7N3O/c16-10-6-8-7(11(8)14-10)2-1-5-15-9-3-4-13-12(9)15/h1-6,13H/b5-1-,7-2+
q-41	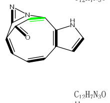	28.2041	170.6939	-5.7242	204.1974	430.1464	InChI=1S/C12H7N3O/c16-10-6-9-11-7(4-5-13-11)2-1-3-8(10)12-14-15(9)12/h1-6,13H/b2-1-,8-3+
B-14	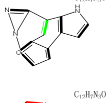	26.2597	177.0629	-3.7344	202.3775	430.1767	InChI=1S/C12H7N3O/c16-10-6-9-11-8(3-4-13-11)7(10)2-1-5-15-12(9)14-15/h1-6,13H/b5-1-,7-2-
x4-24	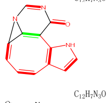	10.8816	276.6831	-7.1206	119.1811	430.4757	InChI=1S/C12H7N3O/c16-12-9-10-7(4-5-13-10)2-1-3-8-11(9)15(8)6-14-12/h1-6,13H/b2-1-,3-1-,7-2-,8-3+,10-9-
H-34	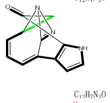	41.4222	173.5108	-4.9031	182.7742	430.4758	InChI=1S/C12H7N3O/c16-10-6-7-2-1-3-9(12-14-15(7)12)8-4-5-13-11(8)10/h1-6,13H/b7-6-
X-40	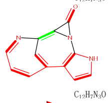	28.7128	271.8828	-9.1544	107.7547	430.5360	InChI=1S/C12H7N3O/c16-12-10-9-6-7(2-1-4-13-9)8-3-5-14-11(8)15(10)12/h1-6,14H
x7-36	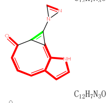	28.041	266.7163	-22.1944	118.642	430.5736	InChI=1S/C12H7N3O/c16-8-3-1-2-7-4-5-13-11(7)10-9(8)12(10)15-6-14-15/h1-6,13H/b3-1-,7-2-,11-10-
o-147	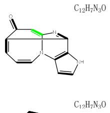	6.3847	94.4552	-2.0196	287.7538	430.7088	InChI=1S/C12H7N3O/c16-9-6-10-14-11-7(9)2-1-5-15(10)8-3-4-13-12(8)11/h1-6,13H/b5-1-,7-2-
n-194	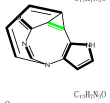	13.6155	225.8832	-5.8859	157.0082	430.9128	InChI=1S/C12H7N3O/c16-12-10-7-2-1-5-15(6-14-12)8-3-4-13-11(8)9(7)10/h1-6,13H/b5-1-,7-2-,14-6-
C-1	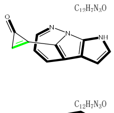	12.3328	307.7674	-3.9600	103.8312	431.3015	InChI=1S/C12H7N3O/c16-10-6-9(10)11-7-2-1-4-14-15(11)12-8(7)3-5-13-12/h1-6,13H
A2-66	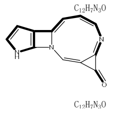	5.7022	385.9717	-1.1302	20.7343	431.3694	InChI=1S/C12H7N3O/c16-11-8-6-15-9(2-1-4-13-10(8)11)7-3-5-14-12(7)15/h1-6,14H/b4-1-,8-6+,9-2-,13-10+

B-151	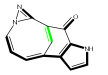 $C_{12}H_7N_3O$	20.3961	196.5206	-1.6404	184.1428	432.0725	InChI=1S/C12H7N3O/c16-11-9-6-7(8-3-4-13-10(8)11)2-1-5-15-12(9)14-15/h1-6,13H/b5-1-,7-2+
s11-3	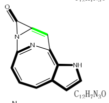 $C_{12}H_7N_3O$	11.3015	329.8128	-6.2257	79.8201	432.211	InChI=1S/C12H7N3O/c16-12-9-6-8-11-7(4-5-13-11)2-1-3-10(14-8)15(9)12/h1-6,13H/b2-1-,3-1-,7-2-,10-3+,11-8+
q-1	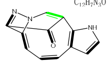 $C_{12}H_7N_3O$	14.1359	128.427	-4.5503	239.5969	432.8718	InChI=1S/C12H7N3O/c16-12-8-6-15-9(11(12)14-15)3-1-2-7-4-5-13-10(7)8/h1-6,13H/b2-1-,9-3-
H1-97	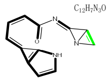 $C_{12}H_7N_3O$	35.261	367.5303	-24.3244	39.895	433.0871	InChI=1S/C12H7N3O/c16-10(14-12-9-6-15(9)12)3-1-2-7-8-4-5-13-11(7)8/h1-6,13H/b3-1-,7-2-,14-12-
A-180	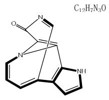 $C_{12}H_7N_3O$	13.396	259.5742	-7.0592	124.9311	433.4148	InChI=1S/C12H7N3O/c16-12-11-10-9-8(3-4-13-9)7(6-14-12)2-1-5-15(10)11/h1-6,13H/b5-1-,7-2+,14-6-
o-112	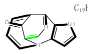 $C_{12}H_7N_3O$	10.6532	89.2497	-3.7913	276.59	433.4179	InChI=1S/C12H7N3O/c16-12-7-2-1-5-15-8-3-4-13-11(8)10(7)14-6-9(12)15/h1-6,13H
o-225	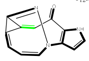 $C_{12}H_7N_3O$	10.5882	106.1123	-4.3093	268.2282	433.5861	InChI=1S/C12H7N3O/c16-10-6-8-7-2-1-5-15(14-11(7)8)9-3-4-13-12(9)10/h1-6,13H/b8-6-
o-224	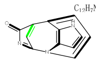 $C_{12}H_7N_3O$	10.5873	106.1127	-4.3087	268.2355	433.5862	InChI=1S/C12H7N3O/c16-12-8-6-10(14-12)15-5-1-2-7(8)11-9(15)3-4-13-11/h1-6,13H
q1-29	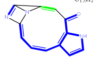 $C_{12}H_7N_3O$	27.4315	217.1627	-14.669	185.868	434.0516	InChI=1S/C12H7N3O/c16-9-6-8-12-14-10(15(8)12)3-1-2-7-4-5-13-11(7)9/h1-6,13H/b2-1-,8-6-,10-3-
q1-36	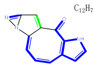 $C_{12}H_7N_3O$	21.3166	217.4147	-4.6911	165.5887	434.2783	InChI=1S/C12H7N3O/c16-12-8-6-10-14-15(10)9(8)3-1-2-7-4-5-13-11(7)12/h1-6,13H/b2-1-,9-3+
X23-27	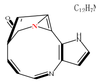 $C_{12}H_7N_3O$	37.6366	265.2255	-16.4262	112.4022	434.3538	InChI=1S/C12H7N3O/c16-12-7-2-1-4-13-8-3-5-14-10(8)11-9(6-7)15(11)12/h1-6,14H/b2-1-,4-1-,7-2-,11-10+,13-4-,13-8-
o-50	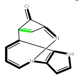 $C_{12}H_7N_3O$	12.5262	142.8663	-4.0064	233.1761	435.358	InChI=1S/C12H7N3O/c16-12-8-6-9(12)14-15-5-1-2-7(8)11-10(15)3-4-13-11/h1-6,13H/b14-9+
o-63	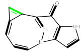 $C_{12}H_7N_3O$	25.8307	247.5647	-14.7347	127.9842	435.3591	InChI=1S/C12H7N3O/c16-12-10-8-6-7(8)2-1-5-15(14-10)9-3-4-13-11(9)12/h1-6,13H/b5-1-,7-2-
j-3	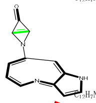 $C_{12}H_7N_3O$	58.8966	272.8244	-31.9211	86.3458	435.6512	InChI=1S/C12H7N3O/c16-12-10-11(12)15(10)7-2-1-4-13-8-3-5-14-9(8)6-7/h1-6,14H/b2-1-,4-1-,7-2+,7-6+,9-6+,13-4+,13-8+
x7-19	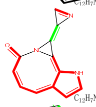 $C_{12}H_7N_3O$	54.8848	268.6388	-28.8255	112.5158	435.9409	InChI=1S/C12H7N3O/c16-9-3-1-2-7-4-5-13-10(7)12-11(15(9)12)8-6-14-8/h1-6,13H/b3-1-,7-2-,11-8+,12-10+
B-254	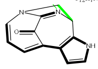 $C_{12}H_7N_3O$	19.3978	119.4070	0.2696	223.1006	436.2248	InChI=1S/C12H7N3O/c16-11-8-2-1-5-15-6-9(14-12(11)15)10-7(8)3-4-13-10/h1-6,13H
H-80	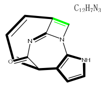 $C_{12}H_7N_3O$	15.3772	210.2573	-4.4337	163.2048	436.311	InChI=1S/C12H7N3O/c16-12-9-3-1-2-7-6-15(10(7)14-12)11-8(9)4-5-13-11/h1-6,13H/b2-1-,9-3-
H11-80	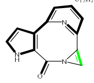 $C_{12}H_7N_3O$	24.1841	286.613	-13.3339	87.6126	436.3662	InChI=1S/C12H7N3O/c16-12-11-7(4-5-13-11)8-2-1-3-9(14-8)10-6-15(10)12/h1-6,13H
B-164	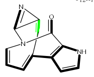 $C_{12}H_7N_3O$	4.1753	326.4802	-3.5774	96.0822	436.8150	InChI=1S/C12H7N3O/c16-12-10-8(3-4-13-10)7-2-1-5-15(12)11-9(6-7)14-11/h1-6,13H/b5-1-,7-2+,9-6-

H-38	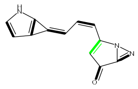 $C_{12}H_7N_3O$	33.1034	303.5849	-11.4176	95.9237	437.1871	InChI=1S/C12H7N3O/c16-10-6-7(15-12(10)14-15)2-1-3-8-9-4-5-13-11(8)9/h1-6,13H/b2-1-,8-3-
A2-3	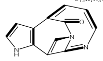 $C_{12}H_7N_3O$	40.1941	204.6816	-1.9358	116.1892	437.4215	InChI=1S/C12H7N3O/c16-12-8-2-1-4-14-11-9(6-15(11)12)10-7(8)3-5-13-10/h1-6,13H/b4-1-,8-2-,14-11-
s11-18	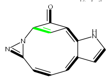 $C_{12}H_7N_3O$	33.0605	213.4451	-5.5896	168.068	437.6464	InChI=1S/C12H7N3O/c16-12-8-6-9(12)15-10(14-15)3-1-2-7-4-5-13-11(7)8/h1-6,13H/b3-1-,7-2-,11-8-
A2-63	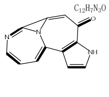 $C_{12}H_7N_3O$	26.6227	272.1981	-11.0367	113.3979	437.7401	InChI=1S/C12H7N3O/c16-10-6-9-12-14-4-1-2-8(15(9)12)7-3-5-13-11(7)10/h1-6,13H
I-16	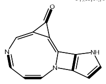 $C_{12}H_7N_3O$	5.3445	390.1145	-1.2643	20.0589	437.7894	InChI=1S/C12H7N3O/c16-12-7-6-13-3-1-5-15-8-2-4-14-10(8)11(15)9(7)12/h1-6,14H/b5-1-,7-6+,11-9+,13-3-
H11-94	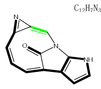 $C_{12}H_7N_3O$	6.1053	371.0272	-1.0262	50.7809	438.0128	InChI=1S/C12H7N3O/c16-12-8-2-1-3-9-10(14-9)6-15(12)11-7(8)4-5-13-11/h1-6,13H/b3-1-,8-2-,10-6+
I-98	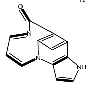 $C_{12}H_7N_3O$	29.6416	240.3748	-2.2587	93.0731	438.3743	InChI=1S/C12H7N3O/c16-12-10-7-6-9(10)15(5-1-3-14-12)8-2-4-13-11(7)8/h1-6,13H/b5-1-,14-3+
B-24	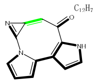 $C_{12}H_7N_3O$	13.5272	289.0356	-2.3639	110.6306	438.5470	InChI=1S/C12H7N3O/c16-10-6-8-12(14-8)15-5-1-2-9(15)7-3-4-13-11(7)10/h1-6,13H/b8-6+
o-4	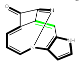 $C_{12}H_7N_3O$	11.2385	191.6347	-4.238	189.6789	438.6667	InChI=1S/C12H7N3O/c16-12-7-2-1-5-15-10-3-4-13-9(10)6-8(7)11(12)14-15/h1-6,13H/b5-1-,7-2+
n-40	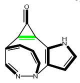 $C_{12}H_7N_3O$	29.388	228.7723	-10.501	152.1026	438.9068	InChI=1S/C12H7N3O/c16-12-9-7-2-1-5-15(14-6-7)8-3-4-13-11(8)10(9)12/h1-6,13H
r-35	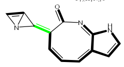 $C_{12}H_7N_3O$	31.0665	281.8318	-23.8393	88.9774	438.9889	InChI=1S/C12H7N3O/c16-12-8(10-9-6-15(9)10)3-1-2-7-4-5-13-11(7)14-12/h1-6H,(H,13,14,16)/b3-1-,7-2-,10-8+
o-27	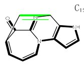 $C_{12}H_7N_3O$	9.2155	119.5684	-1.9931	257.9555	439.9163	InChI=1S/C12H7N3O/c16-11-7-2-1-5-15-9-3-4-13-10(9)8(6-7)14-12(11)15/h1-6,13H
F-37	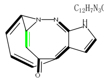 $C_{12}H_7N_3O$	17.2243	237.6246	-6.8365	154.3888	440.0133	InChI=1S/C12H7N3O/c16-11-6-10-9-3-1-2-7(11)8-4-5-13-12(8)14-15(9)10/h1-6H,(H,13,14)/b2-1-,8-7-,9-3+,10-6-
H-141	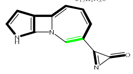 $C_{12}H_7N_3O$	6.0338	390.3097	-2.5098	37.7923	440.3037	InChI=1S/C12H7N3O/c16-12-10(14-12)7-2-1-3-9-8-4-5-13-11(8)15(9)6-7/h1-6,13H
H-10	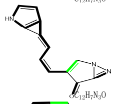 $C_{12}H_7N_3O$	33.1265	305.2755	-11.626	97.7105	440.3121	InChI=1S/C12H7N3O/c16-11-7(6-15-12(11)14-15)2-1-3-8-9-4-5-13-10(8)9/h1-6,13H/b2-1-,8-3-
H-143	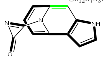 $C_{12}H_7N_3O$	22.0742	186.8198	-12.9146	212.547	440.5718	InChI=1S/C12H7N3O/c16-12-11(14-12)15-7-2-1-3-10(15)8-4-5-13-9(8)6-7/h1-6,13H
I-30	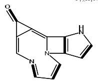 $C_{12}H_7N_3O$	5.4594	420.9913	-1.3202	2.4	440.6082	InChI=1S/C12H7N3O/c16-12-7-6-13-3-1-5-15-8-2-4-14-10(8)11(15)9(7)12/h1-6,14H/b5-1-,7-6+,11-9+,13-3+
n-162	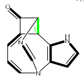 $C_{12}H_7N_3O$	11.0617	142.8319	-2.4058	242.0201	440.8303	InChI=1S/C12H7N3O/c16-12-7-2-1-5-15-6-14-11(12)9(7)10-8(15)3-4-13-10/h1-6,13H/b5-1-,7-2+
q-40	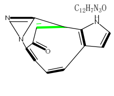 $C_{12}H_7N_3O$	25.3855	168.3784	-6.4861	220.3222	440.8509	InChI=1S/C12H7N3O/c16-10-6-8-11-7(4-5-13-11)2-1-3-9(10)15-12(8)14-15/h1-6,13H/b2-1-,9-3+

H1-71	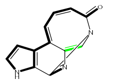 $C_{12}H_7N_3O$	27.7442	166.2168	-0.691	183.6354	440.907	InChI=1S/C12H7N3O/c16-10-3-1-2-7-8-4-5-13-11(8)12-14-9(7)6-15(10)12/h1-6,13H/b3-1-,7-2-
H11-38	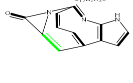 $C_{12}H_7N_3O$	18.3831	261.2861	-5.7578	143.3085	440.9325	InChI=1S/C12H7N3O/c16-12-9-6-7-2-1-3-10(15(9)12)14-11-8(7)4-5-13-11/h1-6H,(H,13,14)/b9-6-
s11-25	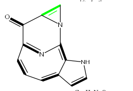 $C_{12}H_7N_3O$	20.4563	280.446	-7.4178	94.2085	441.7953	InChI=1S/C12H7N3O/c16-11-8-3-1-2-7-4-5-13-10(7)12(14-8)15-6-9(11)15/h1-6,13H/b2-1-,3-1-,7-2-,8-3-,12-10-
H11-83	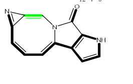 $C_{12}H_7N_3O$	13.1215	308.3287	-3.0582	94.3841	441.9333	InChI=1S/C12H7N3O/c16-12-11-7(4-5-13-11)10-3-1-2-8-9(14-8)6-15(10)12/h1-6,13H/b2-1-,9-6+,10-3-
G-49	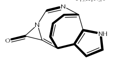 $C_{12}H_7N_3O$	19.3638	243.3415	-8.5373	164.2615	442.3374	InChI=1S/C12H7N3O/c16-12-11-8-2-1-3-9(14-6-15(11)12)10-7(8)4-5-13-10/h1-6,13H/b11-8+,14-6-
n-131	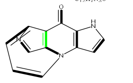 $C_{12}H_7N_3O$	13.6246	164.3127	-3.5853	224.3902	442.3669	InChI=1S/C12H7N3O/c16-12-10-7-2-1-5-15(9(10)6-14-7)8-3-4-13-11(8)12/h1-6,13H
H1-37	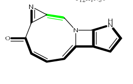 $C_{12}H_7N_3O$	32.0051	339.0933	-0.5383	49.937	442.7323	InChI=1S/C12H7N3O/c16-10-3-1-2-9-7-4-5-13-12(7)15(9)6-8-11(10)14-8/h1-6,13H/b3-1-,8-6+,9-2-
B-255	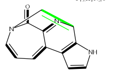 $C_{12}H_7N_3O$	9.9845	121.8987	-3.1551	268.2289	444.2806	InChI=1S/C12H7N3O/c16-12-11-7-2-1-5-15(12)6-9(14-11)10-8(7)3-4-13-10/h1-6,13H
H1-80	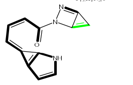 $C_{12}H_7N_3O$	28.9638	387.1369	-20.9407	44.7785	444.9692	InChI=1S/C12H7N3O/c16-11(15-10-6-9(10)14-15)3-1-2-7-8-4-5-13-12(7)8/h1-6,13H/b3-1-,7-2-
H1-11	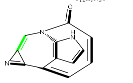 $C_{12}H_7N_3O$	17.29	239.3982	-4.2537	168.1118	445.0442	InChI=1S/C12H7N3O/c16-10-3-1-2-7-8-4-5-13-12(8)15(10)6-9-11(7)14-9/h1-6,13H/b3-1-,7-2+
E-50	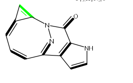 $C_{12}H_7N_3O$	16.0984	303.4664	-10.9586	79.9601	445.2336	InChI=1S/C12H7N3O/c16-12-11-8(4-5-13-11)9-3-1-2-7-6-10(7)15(12)14-9/h1-6,13H/b3-1-,7-2-
H11-89	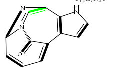 $C_{12}H_7N_3O$	20.1185	125.5402	0.3321	232.4967	445.44	InChI=1S/C12H7N3O/c16-12-8-2-1-3-10-14-9(6-15(10)12)11-7(8)4-5-13-11/h1-6,13H
x5-30	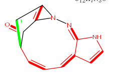 $C_{12}H_7N_3O$	13.678	317.1055	-7.8027	67.6148	445.5954	InChI=1S/C12H7N3O/c16-11-8-3-1-2-7-4-5-13-12(7)14-15-9(6-8)10(11)15/h1-6H,(H,13,14)/b3-1-,7-2-
H11-147	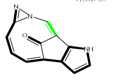 $C_{12}H_7N_3O$	4.2429	344.843	-1.6071	84.913	445.9834	InChI=1S/C12H7N3O/c16-12-8-2-1-3-10-14-15(10)6-9(12)11-7(8)4-5-13-11/h1-6,13H/b3-1-,8-2-,9-6-
A2-51	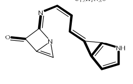 $C_{12}H_7N_3O$	30.7967	381.8482	-20.3529	41.3775	445.9847	InChI=1S/C12H7N3O/c16-11-9-6-15(9)12(11)14-4-1-2-7-8-3-5-13-10(7)8/h1-6,13H/b4-1-,7-2-,14-12-
o-198	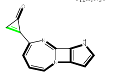 $C_{12}H_7N_3O$	13.2262	384.8515	-3.4294	46.4006	446.5643	InChI=1S/C12H7N3O/c16-10-6-7(10)8-2-1-5-15-9-3-4-13-11(9)12(15)14-8/h1-6,13H
H11-50	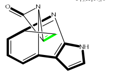 $C_{12}H_7N_3O$	20.3525	262.5275	-6.2512	139.5763	446.7743	InChI=1S/C12H7N3O/c16-12-10-6-7-2-1-3-9(15(10)12)8-4-5-13-11(8)14-7/h1-6H,(H,13,14)/b10-6-
o-32	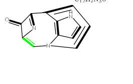 $C_{12}H_7N_3O$	11.441	144.8331	-3.6187	239.6874	447.6102	InChI=1S/C12H7N3O/c16-12-8-6-15-5-1-2-7(11(12)14-8)10-9(15)3-4-13-10/h1-6,13H/b8-6-
A2-31	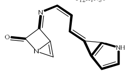 $C_{12}H_7N_3O$	30.9743	388.4952	-20.1535	41.3593	447.996	InChI=1S/C12H7N3O/c16-12-11(9-6-15(9)12)13-4-1-2-7-8-3-5-14-10(7)8/h1-6,14H/b4-1-,7-2-,13-11+

o-131	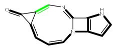		5.2523	401.8744	-1.3802	17.24	448.1023	InChI=1S/C12H7N3O/c16-11-7-2-1-5-15-9-3-4-13-10(9)12(15)14-6-8(7)11/h1-6,13H/b5-1-,7-2+,8-6+,14-12-
H11-150	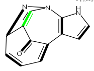	$C_{12}H_7N_3O$	21.2587	120.6305	0.1132	231.3328	448.1873	InChI=1S/C12H7N3O/c16-11-7-2-1-3-10-9(11)6-15(14-10)12-8(7)4-5-13-12/h1-6,13H
s11-52	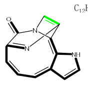	$C_{12}H_7N_3O$	23.3324	226.3885	-2.0801	123.1009	448.3755	InChI=1S/C12H7N3O/c16-12-8-3-1-2-7-4-5-13-10(7)11-9(14-8)6-15(11)12/h1-6,13H/b3-1-,7-2-,11-10+
q11-29	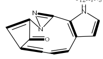	$C_{12}H_7N_3O$	23.852	179.2223	-13.0797	215.8213	448.5412	InChI=1S/C12H7N3O/c16-11-8-3-1-2-7-4-5-13-10(7)12-14-15(12)9(11)6-8/h1-6,13H/b2-1-,8-3?
n-142	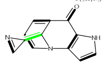	$C_{12}H_7N_3O$	27.9944	168.3973	-11.9776	237.6052	448.6427	InChI=1S/C12H7N3O/c16-12-7-2-1-5-15(11(7)8-6-14-8)9-3-4-13-10(9)12/h1-6,13H/b11-8+
H11-95	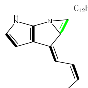	$C_{12}H_7N_3O$	18.4133	423.5314	-14.2717	12.7394	448.7571	InChI=1S/C12H7N3O/c16-12-9(14-12)3-1-2-7-8-4-5-13-11(8)15-6-10(7)15/h1-6,13H/b3-1-,7-2-
H1-94	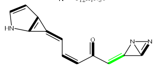	$C_{12}H_7N_3O$	44.6497	310.494	-27.7063	96.2767	448.9616	InChI=1S/C12H7N3O/c16-7(6-10-12-14-15(10)12)2-1-3-8-9-4-5-13-11(8)9/h1-6,13H/b2-1-,8-3-,10-6-
B-244	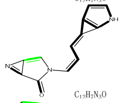	$C_{12}H_7N_3O$	29.8333	307.8213	-12.4353	112.7730	449.4524	InChI=1S/C12H7N3O/c16-12-11-9(14-11)6-15(12)5-1-2-7-8-3-4-13-10(7)8/h1-6,13H/b5-1-,7-2-
H-122	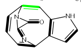	$C_{12}H_7N_3O$	7.9072	294.471	-5.2645	129.4008	450.218	InChI=1S/C12H7N3O/c16-12-11-14-9-3-1-2-7(15(11)12)6-10-8(9)4-5-13-10/h1-6,13H/b2-1-,3-1-,7-2-,7-6-,9-3-,9-8-,10-6+,14-11+
B-110	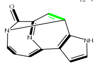	$C_{12}H_7N_3O$	8.4499	119.8740	-3.9032	278.1428	450.4195	InChI=1S/C12H7N3O/c16-12-9-6-10-11-7(3-4-13-11)8(14-9)2-1-5-15(10)12/h1-6,13H/b5-1-,8-2-
i-15	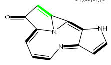	$C_{12}H_7N_3O$	21.9471	242.6314	-8.4386	155.6394	450.7177	InChI=1S/C12H7N3O/c16-10-6-9-12-11-7(3-5-14-11)13-4-1-2-8(10)15(9)12/h1-6,14H/b4-1-,8-2-,12-11+,13-7+
H1-129	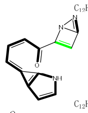	$C_{12}H_7N_3O$	37.9257	291.8006	-17.0545	122.6116	450.8782	InChI=1S/C12H7N3O/c16-10(9-6-11-14-15(9)11)3-1-2-7-8-4-5-13-12(7)8/h1-6,13H/b3-1-,7-2-
X23-36	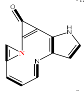	$C_{12}H_7N_3O$	35.5755	361.6302	-16.7489	48.5656	452.1322	InChI=1S/C12H7N3O/c16-12-9-10-8(3-5-14-10)13-4-1-2-7-6-15(7)11(9)12/h1-6,14H/b2-1-,13-4+
n-137	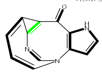	$C_{12}H_7N_3O$	14.0185	253.4141	-6.434	142.7367	452.7306	InChI=1S/C12H7N3O/c16-12-9-7-2-1-5-15(6-14-10(7)9)8-3-4-13-11(8)12/h1-6,13H/b5-1-,7-2-,14-6-
o-1	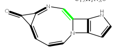	$C_{12}H_7N_3O$	4.3324	386.2022	-1.4882	54.5744	453.659	InChI=1S/C12H7N3O/c16-12-7-2-1-5-15-8-3-4-13-11(8)9(15)6-14-10(7)12/h1-6,13H/b5-1-,7-2+,9-6-,14-10+
i-27	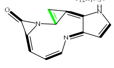	$C_{12}H_7N_3O$	26.1576	396.5689	-18.8152	31.8228	453.808	InChI=1S/C12H7N3O/c16-12-9-2-1-4-13-8-3-5-14-11(8)7-6-10(7)15(9)12/h1-6,14H/b4-1-,9-2-,11-7+,13-8+
X22-38	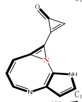	$C_{12}H_7N_3O$	49.2765	343.3314	-24.8616	51.2894	454.0522	InChI=1S/C12H7N3O/c16-10-6-7(10)11-9-2-1-4-13-8-3-5-14-12(8)15(9)11/h1-6,14H/b2-1-,13-4-
s12-33	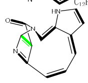	$C_{12}H_7N_3O$	21.5032	174.6845	-9.1315	212.826	454.0639	InChI=1S/C12H7N3O/c16-12-10-8-3-1-2-7-4-5-13-9(7)6-15(12)11(10)14-8/h1-6,13H/b3-1-,7-2-,9-6-
q-7	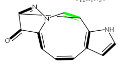	$C_{12}H_7N_3O$	14.7354	131.0188	-3.3814	240.7674	454.7878	InChI=1S/C12H7N3O/c16-12-9-3-1-2-7-4-5-13-10(7)8-6-15(9)14-11(8)12/h1-6,13H/b2-1-,9-3-

$C_{12}H_7N_3O$

H11-20	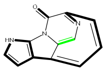	$C_{12}H_7N_3O$	18.5804	193.3616	-5.2157	200.3001	455.6606	InChI=1S/C12H7N3O/c16-12-9-3-1-2-7-8-4-5-13-11(8)15(12)10(7)6-14-9/h1-6,13H/b2-1-,3-1-,7-2-,9-3-
F-52	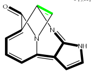	$C_{12}H_7N_3O$	30.4118	116.3083	-1.5718	228.2096	455.9466	InChI=1S/C12H7N3O/c16-12-7-2-1-3-10-8-4-5-13-11(8)14-9(7)6-15(10)12/h1-6H,(H,13,14)
X23-47	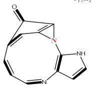	$C_{12}H_7N_3O$	16.2578	311.213	-10.2721	109.6841	456.3918	InChI=1S/C12H7N3O/c16-11-7-2-1-4-13-8-3-5-14-12(8)15-9(6-7)10(11)15/h1-6,14H/b2-1-,13-4-
B-85	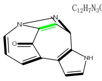	$C_{12}H_7N_3O$	20.6389	129.7882	0.3234	227.3057	456.5246	InChI=1S/C12H7N3O/c16-12-8-2-1-5-15-10(12)6-9(14-15)11-7(8)3-4-13-11/h1-6,13H
B-28	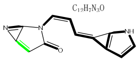	$C_{12}H_7N_3O$	30.1443	313.3940	-12.3300	115.9743	456.9533	InChI=1S/C12H7N3O/c16-10-6-9-12(14-9)15(10)5-1-2-7-8-3-4-13-11(7)8/h1-6,13H/b5-1-,7-2-
B-248	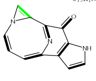	$C_{12}H_7N_3O$	11.7109	302.0143	-5.5247	96.9935	457.0196	InChI=1S/C12H7N3O/c16-12-10-7(3-4-13-10)8-2-1-5-15-6-9(15)11(12)14-8/h1-6,13H/b5-1-,8-2-
E-1	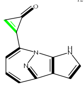	$C_{12}H_7N_3O$	12.8008	304.3642	-4.5826	123.9164	457.1528	InChI=1S/C12H7N3O/c16-11-6-8(11)10-3-1-2-9-7-4-5-13-12(7)15(10)14-9/h1-6,13H
E-2	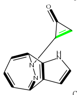	$C_{12}H_7N_3O$	12.8031	304.3628	-4.5825	123.9200	457.1533	InChI=1S/C12H7N3O/c16-11-6-10(11)15-9-3-1-2-8(14-15)7-4-5-13-12(7)9/h1-6,13H
B-135	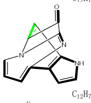	$C_{12}H_7N_3O$	17.5216	232.6777	-6.0462	151.3991	457.1800	InChI=1S/C12H7N3O/c16-11-10-6-9-7(3-4-13-9)8-2-1-5-15(10)12(11)14-8/h1-6,13H/b10-6+
o-99	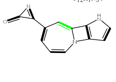	$C_{12}H_7N_3O$	8.9979	374.1875	-2.3464	66.9474	457.508	InChI=1S/C12H7N3O/c16-12-10(14-12)7-2-1-5-15-8-3-4-13-11(8)9(15)6-7/h1-6,13H
E-33	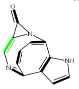	$C_{12}H_7N_3O$	10.0664	291.0211	-6.3202	134.5538	457.6312	InChI=1S/C12H7N3O/c16-12-10-6-14-8-2-1-3-9(15(10)12)11-7(8)4-5-13-11/h1-6,13H/b10-6-,14-8+
H1-142	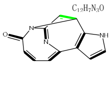	$C_{12}H_7N_3O$	40.9193	222.495	-9.5011	150.6661	457.6818	InChI=1S/C12H7N3O/c16-11-3-1-2-8-7-4-5-13-12(7)9-6-10(14-8)15(9)11/h1-6,13H/b3-1-,8-2-
H11-119	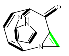	$C_{12}H_7N_3O$	18.6861	243.6485	-9.0381	153.2403	458.0606	InChI=1S/C12H7N3O/c16-11-8-2-1-3-9(15-6-10(11)15)7-4-5-13-12(7)14-8/h1-6H,(H,13,14)
B-5	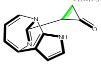	$C_{12}H_7N_3O$	45.4174	203.8970	-19.2428	193.2612	458.2123	InChI=1S/C12H7N3O/c16-10-6-8(10)12-14-9-2-1-5-15(12)11-7(9)3-4-13-11/h1-6,13H
H-88	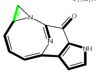	$C_{12}H_7N_3O$	36.7731	260.6384	-19.5216	129.1949	458.2484	InChI=1S/C12H7N3O/c16-11-10-8(4-5-13-10)9-3-1-2-7-6-15(7)12(11)14-9/h1-6,13H/b2-1-,9-3-
q-30	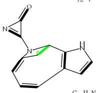	$C_{12}H_7N_3O$	7.3484	339.3393	-4.091	94.3142	458.2538	InChI=1S/C12H7N3O/c16-12-11(14-12)15-8-3-1-2-7-4-5-13-10(7)9(15)6-8/h1-6,13H/b2-1-,3-1-,7-2-,8-3+,10-9-
x3-10	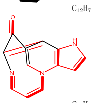	$C_{12}H_7N_3O$	35.7824	124.9855	-4.4295	190.5335	458.5641	InChI=1S/C12H7N3O/c16-12-7-6-15-5-1-3-13-11(12)9(7)10-8(15)2-4-14-10/h1-6,14H/b5-1-,13-3+
H1-93	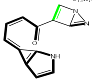	$C_{12}H_7N_3O$	38.7888	297.1687	-17.1392	125.8827	458.8206	InChI=1S/C12H7N3O/c16-10(9-6-15-12(9)14-15)3-1-2-7-8-4-5-13-11(7)8/h1-6,13H/b3-1-,7-2-
o-139	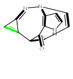	$C_{12}H_7N_3O$	13.2107	255.7504	-6.1328	154.8829	459.8502	InChI=1S/C12H7N3O/c16-12-7-2-1-5-15(14-9-6-8(7)9)10-3-4-13-11(10)12/h1-6,13H/b5-1-,7-2-,14-9-

r-13	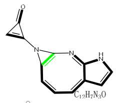 $C_{12}H_7N_3O$	48.9877	327.9018	-25.9436	77.0131	460.3606	InChI=1S/C12H7N3O/c16-10-6-9(10)15-8-3-1-2-7-4-5-13-11(7)14-12(8)15/h1-6H,(H,13,14)
o-59	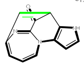 $C_{12}H_7N_3O$	9.8089	106.7539	-4.07	296.5981	460.6573	InChI=1S/C12H7N3O/c16-11-8-6-7-2-1-5-15(12(11)14-7)9-3-4-13-10(8)9/h1-6,13H
G1-78	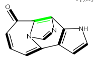 $C_{12}H_7N_3O$	9.3374	158.6259	-3.8307	254.9371	460.81	InChI=1S/C12H7N3O/c16-9-3-1-2-8-7-4-5-13-10(7)11-12(9)15(8)6-14-11/h1-6,13H
G-10	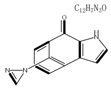 $C_{12}H_7N_3O$	34.2046	236.4184	-1.7806	171.963	461.0401	InChI=1S/C12H7N3O/c16-12-9-3-1-2-8(11(9)15-6-14-15)7-4-5-13-10(7)12/h1-6,13H
o-146	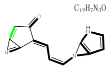 $C_{12}H_7N_3O$	23.3257	285.1249	-15.2076	147.2464	461.6591	InChI=1S/C12H7N3O/c16-10-6-8-11(14-8)7(10)2-1-5-15-9-3-4-13-12(9)15/h1-6,13H/b5-1-,7-2-
B-185	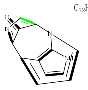 $C_{12}H_7N_3O$	11.4243	162.0162	-3.6609	233.7016	462.4641	InChI=1S/C12H7N3O/c16-11-9-6-14-10(11)7-2-1-5-15(9)12-8(7)3-4-13-12/h1-6,13H
H1-19	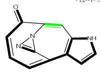 $C_{12}H_7N_3O$	27.3842	246.8254	-5.8965	137.6607	462.5736	InChI=1S/C12H7N3O/c16-11-3-1-2-8-7-4-5-13-9(7)6-10(11)15-12(8)14-15/h1-6,13H/b3-1-,8-2-
H-31	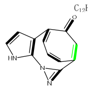 $C_{12}H_7N_3O$	24.1309	171.9646	-3.773	231.0461	462.6938	InChI=1S/C12H7N3O/c16-10-6-7-2-1-3-8(10)9-4-5-13-12(9)15-11(7)14-15/h1-6,13H
H11-62	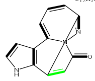 $C_{12}H_7N_3O$	31.3014	175.841	-7.5325	198.9007	462.9271	InChI=1S/C12H7N3O/c16-11-6-8-9-2-1-3-10(15(11)14-9)7-4-5-13-12(7)8/h1-6,13H
i-24	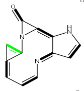 $C_{12}H_7N_3O$	21.0285	366.8348	-16.418	63.3099	463.1092	InChI=1S/C12H7N3O/c16-12-11-10-8(3-5-14-10)13-4-1-2-7-6-9(7)15(11)12/h1-6,14H/b4-1-,7-2-,11-10+,13-8+
k-32	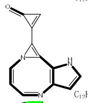 $C_{12}H_7N_3O$	58.7003	327.7866	-30.6852	72.9821	464.4104	InChI=1S/C12H7N3O/c16-9-6-7(9)11-12-10-8(2-4-14-10)13-3-1-5-15(11)12/h1-6,14H/b5-1-,13-3-
q1-6	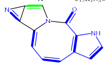	13.1394	253.5441	-8.0067	187.0666	464.5178	InChI=1S/C12H7N3O/c16-12-10-7(4-5-13-10)2-1-3-9-11-8(14-11)6-15(9)12/h1-6,13H/b2-1-,9-3-
G-2	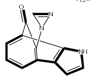 $C_{12}H_7N_3O$	4.2059	305.752	-3.4806	135.3752	464.5826	InChI=1S/C12H7N3O/c16-12-9-3-1-2-8(11(12)15-6-14-15)7-4-5-13-10(7)9/h1-6,13H
r-12	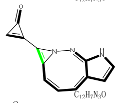 $C_{12}H_7N_3O$	50.36	331.8359	-24.3013	74.823	464.7951	InChI=1S/C12H7N3O/c16-10-6-8(10)11-9-3-1-2-7-4-5-13-12(7)14-15(9)11/h1-6H,(H,13,14)/b3-1-,7-2-
B-238	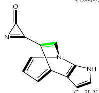 $C_{12}H_7N_3O$	5.6111	308.6107	-3.8788	128.2634	465.0462	InChI=1S/C12H7N3O/c16-12-10(14-12)9-6-15-5-1-2-7(9)8-3-4-13-11(8)15/h1-6,13H
H11-37	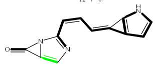 $C_{12}H_7N_3O$	33.8031	362.4331	-19.9895	84.2452	465.297	InChI=1S/C12H7N3O/c16-12-9-6-14-10(15(9)12)3-1-2-7-8-4-5-13-11(7)8/h1-6,13H/b3-1-,7-2-
o-234	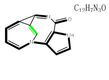 $C_{12}H_7N_3O$	13.8556	241.4753	-5.761	171.6303	466.3149	InChI=1S/C12H7N3O/c16-12-11-9(3-4-13-11)15-5-1-2-7-8(6-15)10(7)14-12/h1-6,13H/b14-10-
X-4	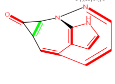 $C_{12}H_7N_3O$	34.7281	276.2723	-12.8916	128.3278	466.5022	InChI=1S/C12H7N3O/c16-11-9-6-7-2-1-4-14-15(10(9)11)12-8(7)3-5-13-12/h1-6,13H/b2-1-,14-4-
A2-8	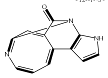 $C_{12}H_7N_3O$	29.6333	185.3333	0.0617	177.2056	466.628	InChI=1S/C12H7N3O/c16-12-9-6-10-13-4-1-2-7(9)8-3-5-14-11(8)15(10)12/h1-6,14H/b2-1-,4-1-,7-2-,13-4-,13-10-

H1-30	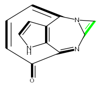 $C_{12}H_7N_3O$	14.5001	281.2593	-6.6309	128.4592	467.514	InChI=1S/C12H7N3O/c16-9-3-1-2-8-7-4-5-13-11(7)12(9)14-10-6-15(8)10/h1-6,13H/b3-1-,8-2+
n-182	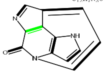 $C_{12}H_7N_3O$	70.4081	106.0874	-2.6801	164.1376	467.78	InChI=1S/C12H7N3O/c16-12-11-9-7(6-14-11)2-1-5-15(12)8-3-4-13-10(8)9/h1-6,13H/b5-1-,7-2-
s12-18	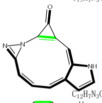 $C_{12}H_7N_3O$	65.2568	321.9806	-23.7271	73.2959	468.0456	InChI=1S/C12H7N3O/c16-12-8-6-9-7(4-5-13-9)2-1-3-10-14-15(10)11(8)12/h1-6,13H/b3-1-,7-2-,9-6+
H-36	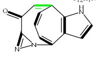 $C_{12}H_7N_3O$	20.1355	201.1598	-5.9547	204.0948	468.0947	InChI=1S/C12H7N3O/c16-10-6-7-2-1-3-9(15-12(10)14-15)8-4-5-13-11(7)8/h1-6,13H/b7-6+
H11-152	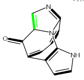 $C_{12}H_7N_3O$	15.997	208.7885	-3.9339	199..7616	469.2695	InChI=1S/C12H7N3O/c16-11-7-2-1-3-10-14-6-9(11)15(10)12-8(7)4-5-13-12/h1-6,13H/b3-1-,7-2-
H11-28	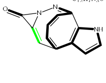 $C_{12}H_7N_3O$	16.9922	285.1541	-8.841	140.0194	469.7302	InChI=1S/C12H7N3O/c16-12-10-6-7-2-1-3-9(14-15(10)12)11-8(7)4-5-13-11/h1-6,13H/b10-6-,14-9?
B-37	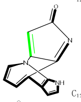 $C_{12}H_7N_3O$	7.7809	244.2875	-2.9872	170.6874	470.3701	InChI=1S/C12H7N3O/c16-10-6-9-11(14-10)7-2-1-5-15(9)12-8(7)3-4-13-12/h1-6,13H
o-130	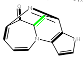 $C_{12}H_7N_3O$	10.3113	115.9426	-2.6044	291.9782	470.4342	InChI=1S/C12H7N3O/c16-12-7-2-1-5-15-9-3-4-13-11(9)8(14-7)6-10(12)15/h1-6,13H
q1-27	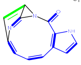 $C_{12}H_7N_3O$	34.9416	226.7454	-11.6374	187.3512	471.2955	InChI=1S/C12H7N3O/c16-12-10-7(4-5-13-10)2-1-3-8-6-9-11(14-8)15(9)12/h1-6,13H/b2-1-,8-3-
o-140	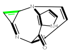 $C_{12}H_7N_3O$	15.12	263.9429	-11.2623	148.57	471.4065	InChI=1S/C12H7N3O/c16-12-7-2-1-5-15(10-6-8(10)14-7)9-3-4-13-11(9)12/h1-6,13H/b5-1-,7-2+,14-8-
B-61	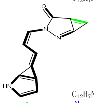 $C_{12}H_7N_3O$	40.5091	366.9093	-24.6072	58.9434	471.4340	InChI=1S/C12H7N3O/c16-12-9-6-10(9)14-15(12)5-1-2-7-8-3-4-13-11(7)8/h1-6,13H/b5-1-,7-2-
p1-21	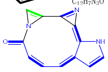 $C_{12}H_7N_3O$	49.5003	337.5004	-27.4487	78.5804	471.5873	InChI=1S/C12H7N3O/c16-9-3-1-2-7-4-5-13-10(7)12-11(14-12)8-6-15(8)9/h1-6,13H/b3-1-,7-2-,12-10-
s11-9	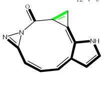 $C_{12}H_7N_3O$	49.0535	361.086	-16.5826	68.6329	471.6874	InChI=1S/C12H7N3O/c16-12-9-6-8(9)11-7(4-5-13-11)2-1-3-10-14-15(10)12/h1-6,13H/b3-1-,7-2-,11-8+
A-64	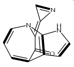 $C_{12}H_7N_3O$	23.6511	209.1926	-10.162	210.4172	473.2291	InChI=1S/C12H7N3O/c16-11-7-2-1-5-15(10(11)9-6-14-9)12-8(7)3-4-13-12/h1-6,13H/b10-9+
B-163	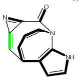 $C_{12}H_7N_3O$	4.9405	371.1543	-3.7825	91.1762	473.5028	InChI=1S/C12H7N3O/c16-12-10-9(14-10)6-7-2-1-5-15(12)11-8(7)3-4-13-11/h1-6,13H/b9-6+
n-43	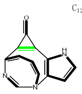 $C_{12}H_7N_3O$	26.5761	288.6748	-8.8186	140.1245	474.8718	InChI=1S/C12H7N3O/c16-12-9-7-2-1-5-15(6-14-7)8-3-4-13-11(8)10(9)12/h1-6,13H
o-97	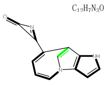 $C_{12}H_7N_3O$	7.4287	326.9653	-2.9058	118.3599	475.0624	InChI=1S/C12H7N3O/c16-12-11(14-12)7-2-1-5-15-6-8(7)10-9(15)3-4-13-10/h1-6,13H
p1-30	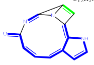 $C_{12}H_7N_3O$	18.4584	280.8982	-11.3441	164.1557	475.4006	InChI=1S/C12H7N3O/c16-10-3-1-2-7-4-5-13-11(7)8-6-9-12(14-10)15(8)9/h1-6,13H/b3-1-,7-2-,11-8+,14-12-
G-46	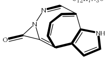 $C_{12}H_7N_3O$	10.9404	298.5454	-7.0494	147.9523	475.5558	InChI=1S/C12H7N3O/c16-12-11-9-3-1-2-7(6-14-15(11)12)10-8(9)4-5-13-10/h1-6,13H/b11-9+,14-6-

E-38	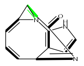 $C_{12}H_7N_3O$	13.639	239.1646	-6.0659	198.0443	475.8388	InChI=1S/C12H7N3O/c16-11-6-10-9-3-1-2-8(14-11)7-4-5-13-12(7)15(9)10/h1-6,13H/b2-1-,9-3-,10-6+,14-8-
H1-126	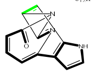 $C_{12}H_7N_3O$	19.6108	251.7064	-11.3912	165.5354	476.2105	InChI=1S/C12H7N3O/c16-11-3-1-2-8-7-4-5-13-9(7)6-10-12(14-8)15(10)11/h1-6,13H/b3-1-,8-2-
I-91	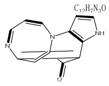 $C_{12}H_7N_3O$	14.6465	169.7737	-4.5927	245.7693	476.2608	InChI=1S/C12H7N3O/c16-12-9-6-7-10(12)11-8(2-4-14-11)15(9)5-1-3-13-7/h1-6,14H/b5-1-,13-3-
p1-53	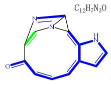 $C_{12}H_7N_3O$	19.3993	209.3147	-11.038	234.1386	476.3942	InChI=1S/C12H7N3O/c16-9-3-1-2-7-4-5-13-10(7)11-12-14-8(9)6-15(11)12/h1-6,13H/b3-1-,7-2-,11-10+
H-147	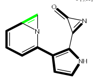 $C_{12}H_7N_3O$	25.0168	439.3958	-17.3921	14.729	476.3971	InChI=1S/C12H7N3O/c16-12-11(14-12)10-8(4-5-13-10)9-3-1-2-7-6-15(7)9/h1-6,13H
s12-31	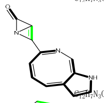 $C_{12}H_7N_3O$	27.1816	363.1546	-16.5929	78.3867	476.74	InChI=1S/C12H7N3O/c16-12-11-10(15(11)12)8-3-1-2-7-4-5-13-9(7)6-14-8/h1-6,13H/b2-1-,3-1-,7-2-,8-3-,9-6+,14-6-,14-8+
o-96	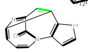 $C_{12}H_7N_3O$	70.2255	129.1134	-0.3704	173.36	477.0705	InChI=1S/C12H7N3O/c16-12-9-6-7-8(14-9)2-1-5-15(12)10-3-4-13-11(7)10/h1-6,13H/b5-1-,8-2+
H-6	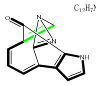 $C_{12}H_7N_3O$	23.0429	189.5113	-6.0834	231.6986	477.2086	InChI=1S/C12H7N3O/c16-11-7-2-1-3-9(12-14-15(12)6-7)8-4-5-13-10(8)11/h1-6,13H/b2-1-,7-6-,9-3-
H-3	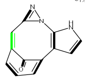 $C_{12}H_7N_3O$	22.9858	232.2597	-3.4305	188.7217	477.3862	InChI=1S/C12H7N3O/c16-11-7-2-1-3-8(11)9-4-5-13-12(9)15-10(6-7)14-15/h1-6,13H/b7-6-
B-33	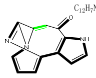 $C_{12}H_7N_3O$	11.4094	237.6571	-6.0927	188.2746	477.4251	InChI=1S/C12H7N3O/c16-10-6-9-12-14-8(2-1-5-15(9)12)7-3-4-13-11(7)10/h1-6,13H/b9-6-
B-88	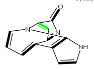 $C_{12}H_7N_3O$	14.1308	178.1172	-5.6147	226.8957	477.4608	InChI=1S/C12H7N3O/c16-12-10-6-9(14-12)7-2-1-5-15(10)11-8(7)3-4-13-11/h1-6,13H
H11-133	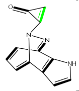 $C_{12}H_7N_3O$	44.6084	264.607	-19.612	151.7783	477.5938	InChI=1S/C12H7N3O/c16-11-6-10(11)15-9-3-1-2-8(14-15)12-7(9)4-5-13-12/h1-6,13H
B-206	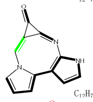 $C_{12}H_7N_3O$	20.4377	296.6558	-9.7353	127.1744	478.1293	InChI=1S/C12H7N3O/c16-11-8-6-15-5-1-2-9(15)7-3-4-13-12(7)14-10(8)11/h1-6,13H/b8-6+,14-10+
x5-34	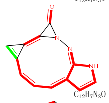 $C_{12}H_7N_3O$	32.1173	416.6908	-22.01	30.7674	478.2888	InChI=1S/C12H7N3O/c16-12-10-9-6-8(9)3-1-2-7-4-5-13-11(7)14-15(10)12/h1-6H,(H,13,14)/b3-1-,7-2-,10-9-
x7-46	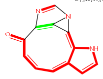 $C_{12}H_7N_3O$	16.0951	263.5092	-11.2466	187.3522	478.7525	InChI=1S/C12H7N3O/c16-8-3-1-2-7-4-5-13-9(7)11-12-10(8)14-6-15(11)12/h1-6,13H/b3-1-,7-2-,11-9-
H11-23	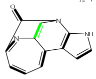 $C_{12}H_7N_3O$	26.5115	197.4967	-9.2987	196.9212	479.0314	InChI=1S/C12H7N3O/c16-12-9-3-1-2-7-8-4-5-13-11(8)15(12)6-10(7)14-9/h1-6,13H
A1-30	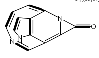 $C_{12}H_7N_3O$	32.3166	264.6705	-12.9051	156.5332	479.9345	InChI=1S/C12H7N3O/c16-12-11-8-6-13-4-1-2-9(15(11)12)7-3-5-14-10(7)8/h1-6,14H/b4-1-,9-2+,13-6-
B-38	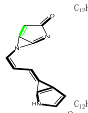 $C_{12}H_7N_3O$	26.3658	323.4106	-14.1996	131.4817	480.8151	InChI=1S/C12H7N3O/c16-10-6-9-12(14-10)15(9)5-1-2-7-8-3-4-13-11(7)8/h1-6,13H/b5-1-,7-2-
H1-138	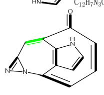 $C_{12}H_7N_3O$	36.5188	170.9108	-4.1722	245.6742	481.7835	InChI=1S/C12H7N3O/c16-10-3-1-2-9-7-4-5-13-12(7)8(10)6-11-14-15(9)11/h1-6,13H/b3-1-,9-2+

H11-39	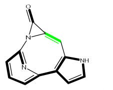 $C_{12}H_7N_3O$	14.995	335.5305	-8.837	105.4118	482.2364	InChI=1S/C12H7N3O/c16-12-10-6-9-7(4-5-13-9)8-2-1-3-11(14-8)15(10)12/h1-6,13H/b10-6-
s11-2	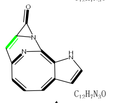 $C_{12}H_7N_3O$	6.4251	360.3669	-3.9391	95.202	482.5814	InChI=1S/C12H7N3O/c16-12-9-6-8-3-1-2-7-4-5-13-10(7)11(14-8)15(9)12/h1-6,13H/b2-1-,3-1-,7-2-,8-3-,11-10-
A-108	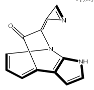 $C_{12}H_7N_3O$	5.3993	305.6151	-3.8571	149.2747	482.8402	InChI=1S/C12H7N3O/c16-11-7-2-1-5-15(10(11)9-6-14-9)12-8(7)3-4-13-12/h1-6,13H/b10-9-
o-124	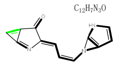 $C_{12}H_7N_3O$	38.2443	359.5606	-23.7052	77.9954	483.0718	InChI=1S/C12H7N3O/c16-11-7-6-9(7)14-8(11)2-1-5-15-10-3-4-13-12(10)15/h1-6,13H/b5-1-,8-2-
o-197	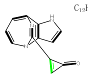 $C_{12}H_7N_3O$	43.6927	202.0178	-19.8559	215.3135	483.3586	InChI=1S/C12H7N3O/c16-10-6-7(10)12-14-8-2-1-5-15(12)9-3-4-13-11(8)9/h1-6,13H
x4-41	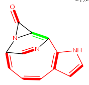 $C_{12}H_7N_3O$	27.4234	262.5196	-8.2632	148.5392	484.3867	InChI=1S/C12H7N3O/c16-12-11-10-9-7(4-5-13-9)2-1-3-8(6-14-10)15(11)12/h1-6,13H/b2-1-,8-3+
o-183	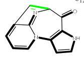 $C_{12}H_7N_3O$	11.2003	177.7562	-2.6419	240.0897	484.6597	InChI=1S/C12H7N3O/c16-11-8-6-7-2-1-5-15(12(7)14-8)9-3-4-13-10(9)11/h1-6,13H
X23-20	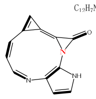 $C_{12}H_7N_3O$	32.7579	404.5339	-22.1232	57.4884	484.9333	InChI=1S/C12H7N3O/c16-12-10-8-6-7(8)2-1-4-13-9-3-5-14-11(9)15(10)12/h1-6,14H/b2-1-,10-8-,13-4-
X22-60	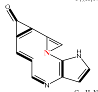 $C_{12}H_7N_3O$	50.9748	312.0827	-23.2406	103.4997	484.9758	InChI=1S/C12H7N3O/c16-11-7-2-1-4-13-8-3-5-14-12(8)15-6-9(15)10(7)11/h1-6,14H/b2-1+,13-4-
o-40	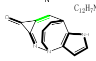 $C_{12}H_7N_3O$	6.8449	315.8798	-2.7449	137.7514	485.0558	InChI=1S/C12H7N3O/c16-12-8-6-7-2-1-5-15(14-11(8)12)9-3-4-13-10(7)9/h1-6,13H/b8-6+,14-11+
H11-49	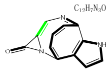 $C_{12}H_7N_3O$	9.7741	296.0713	-4.2852	161.6086	485.8526	InChI=1S/C12H7N3O/c16-12-10-6-14-8-2-1-3-9(15(10)12)7-4-5-13-11(7)8/h1-6,13H/b10-6-,14-8?
x4-34	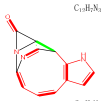 $C_{12}H_7N_3O$	18.8643	265.9221	-6.7289	179.5035	486.3198	InChI=1S/C12H7N3O/c16-12-11-8-6-14-9(15(11)12)3-1-2-7-4-5-13-10(7)8/h1-6,13H/b2-1-,9-3+
H-8	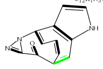 $C_{12}H_7N_3O$	15.5544	262.4972	-6.0425	169.8835	486.3522	InChI=1S/C12H7N3O/c16-11-7-2-1-3-10(15-12(11)14-15)8-4-5-13-9(8)6-7/h1-6,13H/b2-1-,3-1+,7-2-,7-6+,9-6+,10-3+,10-8+
o-108	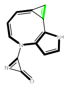 $C_{12}H_7N_3O$	25.2097	413.0662	-15.6831	35.0456	486.6476	InChI=1S/C12H7N3O/c16-12-11(14-12)15-5-1-2-7-6-8(7)10-9(15)3-4-13-10/h1-6,13H/b5-1-,7-2-
H-111	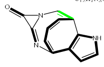 $C_{12}H_7N_3O$	13.7764	286.5892	-7.3514	163.5519	487.8977	InChI=1S/C12H7N3O/c16-12-11-14-9-3-1-2-7(6-15(11)12)10-8(9)4-5-13-10/h1-6,13H/b7-6?,14-11-
o-250	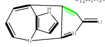 $C_{12}H_7N_3O$	7.7451	243.8332	-2.8344	191.2963	488.064	InChI=1S/C12H7N3O/c16-10-6-8-7-2-1-5-15(12(8)14-10)9-3-4-13-11(7)9/h1-6,13H
H-85	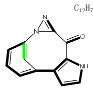 $C_{12}H_7N_3O$	7.0181	408.587	-1.5677	31.0612	488.6959	InChI=1S/C12H7N3O/c16-11-10-9(4-5-13-10)7-2-1-3-8(6-7)15-12(11)14-15/h1-6,13H
p1-10	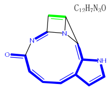 $C_{12}H_7N_3O$	19.971	276.2716	-11.9098	174.1788	489.6852	InChI=1S/C12H7N3O/c16-10-3-1-2-7-4-5-13-11(7)12-8-6-9(14-10)15(8)12/h1-6,13H/b3-1-,7-2-,12-11+,14-9-
B-35	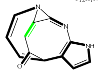 $C_{12}H_7N_3O$	29.4666	261.6122	-12.4034	153.3560	490.6897	InChI=1S/C12H7N3O/c16-10-6-9-12-14-11-8(3-4-13-11)7(10)2-1-5-15(9)12/h1-6,13H/b5-1-,7-2-,9-6+,14-12+

B-84	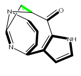 $C_{12}H_7N_3O$	19.8068	199.8298	-5.9387	218.6794	490.7781	InChI=1S/C12H7N3O/c16-12-9-6-10-14-8(2-1-5-15(9)10)7-3-4-13-11(7)12/h1-6,13H
B-211	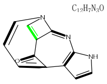 $C_{12}H_7N_3O$	20.9676	208.0068	-4.3459	198.6477	491.8809	InChI=1S/C12H7N3O/c16-10-7-2-1-5-15-6-9(10)12(15)14-11-8(7)3-4-13-11/h1-6,13H/b5-1-,7-2-
i-13	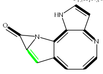 $C_{12}H_7N_3O$	20.6709	321.7143	-9.5137	125.8399	492.3697	InChI=1S/C12H7N3O/c16-12-9-6-7-2-1-4-13-8-3-5-14-10(8)11(7)15(9)12/h1-6,14H/b2-1-,4-1-,7-2-,11-10-,13-4-,13-8-
H-20	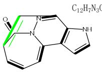 $C_{12}H_7N_3O$	29.6734	169.5819	-1.364	186.8487	492.8094	InChI=1S/C12H7N3O/c16-12-7-2-1-3-10-8-4-5-13-11(8)9(6-7)14-15(10)12/h1-6,13H
B-150	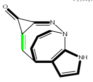 $C_{12}H_7N_3O$	3.8110	316.3044	-2.4339	160.6584	492.9003	InChI=1S/C12H7N3O/c16-11-9-6-7-2-1-5-15(14-10(9)11)12-8(7)3-4-13-12/h1-6,13H/b9-6+,14-10+
E-61	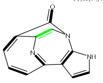 $C_{12}H_7N_3O$	32.8075	190.4475	-4.7519	171.8887	493.1829	InChI=1S/C12H7N3O/c16-12-8-2-1-3-9-7-4-5-13-11(7)15(12)6-10(8)14-9/h1-6,13H
k-41	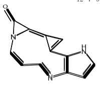 $C_{12}H_7N_3O$	33.5864	401.2117	-22.4158	64.0164	493.3655	InChI=1S/C12H7N3O/c16-12-11-8-6-7(8)10-9(2-4-14-10)13-3-1-5-15(11)12/h1-6,14H/b5-1-,11-8-,13-3+
n-149	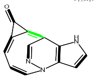 $C_{12}H_7N_3O$	2.7566	323.3734	-2.1323	152.3304	493.6298	InChI=1S/C12H7N3O/c16-12-7-2-1-5-15-9-3-4-13-11(9)8(6-14-15)10(7)12/h1-6,13H/b5-1-,7-2+,10-8-
H11-5	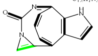 $C_{12}H_7N_3O$	15.0025	331.0532	-8.8023	103.4357	493.6742	InChI=1S/C12H7N3O/c16-12-14-9-3-1-2-7(10-6-15(10)12)8-4-5-13-11(8)9/h1-6,13H/b14-9+
B-103	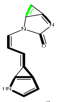 $C_{12}H_7N_3O$	44.3322	409.5198	-24.5352	46.3141	493.8548	InChI=1S/C12H7N3O/c16-12-14-9-6-10(9)15(12)5-1-2-7-8-3-4-13-11(7)8/h1-6,13H/b5-1-,7-2-
r-17	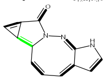 $C_{12}H_7N_3O$	41.5179	384.9292	-21.7348	55.8266	493.9054	InChI=1S/C12H7N3O/c16-12-9-6-8(9)10-3-1-2-7-4-5-13-11(7)14-15(10)12/h1-6H,(H,13,14)/b3-1-,7-2-
F-4	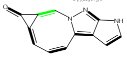 $C_{12}H_7N_3O$	6.103	337.8632	-4.9206	140.2904	494.3954	InChI=1S/C12H7N3O/c16-11-7-2-1-3-10-8-4-5-13-12(8)14-15(10)6-9(7)11/h1-6H,(H,13,14)/b3-1-,7-2+,9-6+
G1-58	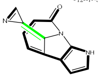 $C_{12}H_7N_3O$	8.9592	381.9419	-4.0671	93.0752	494.5243	InChI=1S/C12H7N3O/c16-10-3-1-2-7-8-4-5-13-12(8)15(10)11(7)9-6-14-9/h1-6,13H/b11-9+
H11-96	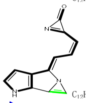 $C_{12}H_7N_3O$	13.8205	457.5465	-9.0367	14.7483	495.6627	InChI=1S/C12H7N3O/c16-12-8(14-12)2-1-3-9-7-4-5-13-11(7)10-6-15(9)10/h1-6,13H/b2-1-,9-3-
r1-19	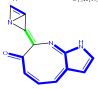 $C_{12}H_7N_3O$	30.2093	346.7206	-20.16	84.0866	495.7694	InChI=1S/C12H7N3O/c16-9-3-1-2-7-4-5-13-12(7)14-10(9)11-8-6-15(8)11/h1-6H,(H,13,14)/b3-1-,7-2-,11-10+
r1-34	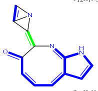 $C_{12}H_7N_3O$	30.2095	346.7198	-20.1599	84.0802	495.7694	InChI=1S/C12H7N3O/c16-9-3-1-2-7-4-5-13-12(7)14-10(9)11-8-6-15(8)11/h1-6H,(H,13,14)/b3-1-,7-2-,11-10-
H-64	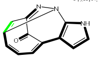 $C_{12}H_7N_3O$	15.3087	176.3736	-4.2285	258.9622	497.6912	InChI=1S/C12H7N3O/c16-11-8-3-1-2-7-6-10(11)14-15(7)12-9(8)4-5-13-12/h1-6,13H/b2-1-,8-3-
r-5	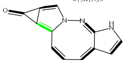 $C_{12}H_7N_3O$	13.101	294.0564	-10.3844	183.9353	497.7215	InChI=1S/C12H7N3O/c16-11-8-6-15-9(10(8)11)3-1-2-7-4-5-13-12(7)14-15/h1-6H,(H,13,14)/b3-1-,7-2-
H-128	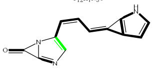 $C_{12}H_7N_3O$	32.7791	365.9397	-19.5399	110.5883	498.8001	InChI=1S/C12H7N3O/c16-12-11-14-6-7(15(11)12)2-1-3-8-9-4-5-13-10(8)9/h1-6,13H/b2-1-,8-3-

H-161	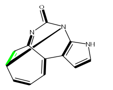 $C_{12}H_7N_3O$	27.464	216.3679	-14.0635	211.2627	499.5639	InChI=1S/C12H7N3O/c16-12-14-10-6-7-2-1-3-8(10)9-4-5-13-11(9)15(7)12/h1-6,13H
s11-38	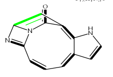 $C_{12}H_7N_3O$	44.5491	170.8566	-8.8544	209.7928	500.351	InChI=1S/C12H7N3O/c16-12-8-6-10-14-9(15(10)12)3-1-2-7-4-5-13-11(7)8/h1-6,13H/b2-1-,3-1-,7-2-,9-3-,11-8+
H1-23	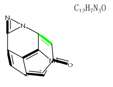 $C_{12}H_7N_3O$	11.6836	291.1883	-4.1	168.9386	500.6245	InChI=1S/C12H7N3O/c16-7-2-1-3-9-8-4-5-13-11(8)10(6-7)15-12(9)14-15/h1-6,13H/b2-1-,9-3+,10-6+
o-11	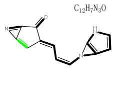 $C_{12}H_7N_3O$	30.069	327.9502	-12.1056	124.1393	500.7148	InChI=1S/C12H7N3O/c16-11-7(6-8-10(11)14-8)2-1-5-15-9-3-4-13-12(9)15/h1-6,13H/b5-1-,7-2+
q-21	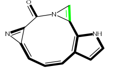 $C_{12}H_7N_3O$	52.026	385.49	-15.1559	56.5832	500.7518	InChI=1S/C12H7N3O/c16-12-11-8(14-11)3-1-2-7-4-5-13-10(7)9-6-15(9)12/h1-6,13H/b2-1-,8-3+
C-57	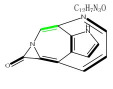 $C_{12}H_7N_3O$	18.3781	282.3149	-6.4475	172.8341	502.1458	InChI=1S/C12H7N3O/c16-12-11-8-2-1-4-13-9(6-15(11)12)10-7(8)3-5-14-10/h1-6,14H/b2-1-,13-4-
E-7	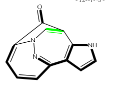 $C_{12}H_7N_3O$	27.2909	194.6153	-3.7697	188.6216	502.7499	InChI=1S/C12H7N3O/c16-12-8-6-15-10(12)3-1-2-9(14-15)7-4-5-13-11(7)8/h1-6,13H
s12-19	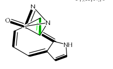 $C_{12}H_7N_3O$	62.4404	341.5027	-20.1788	98.5112	502.7801	InChI=1S/C12H7N3O/c16-12-8-6-9-7(4-5-13-9)2-1-3-10-14-15(10)11(8)12/h1-6,13H/b3-1-,7-2-,9-6-
n-141	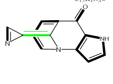 $C_{12}H_7N_3O$	4.4875	301.913	-3.3071	176.2196	503.378	InChI=1S/C12H7N3O/c16-12-7-2-1-5-15(11(7)8-6-14-8)9-3-4-13-10(9)12/h1-6,13H/b11-8-
A2-22	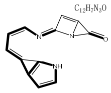 $C_{12}H_7N_3O$	20.1301	476.7627	-12.9076	19.0512	503.5611	InChI=1S/C12H7N3O/c16-12-9-6-10(15(9)12)13-4-1-2-7-8-3-5-14-11(7)8/h1-6,14H/b4-1-,7-2-,13-10+
o-101	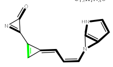 $C_{12}H_7N_3O$	64.4286	385.3272	-42.6067	60.359	503.7176	InChI=1S/C12H7N3O/c16-12-10(14-12)8-6-7(8)2-1-5-15-9-3-4-13-11(9)15/h1-6,13H/b5-1-,7-2+
A1-22	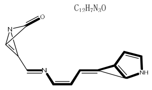 $C_{12}H_7N_3O$	41.1515	436.9778	-24.6839	39.6379	503.8292	InChI=1S/C12H7N3O/c16-12-11-9(15(11)12)6-13-4-1-2-7-8-3-5-14-10(7)8/h1-6,14H/b4-1-,7-2-,13-6+
A2-23	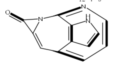 $C_{12}H_7N_3O$	23.1784	269.3333	-11.935	188.8555	504.3166	InChI=1S/C12H7N3O/c16-12-9-6-7-2-1-4-14-11(15(9)12)10-8(7)3-5-13-10/h1-6,13H/b2-1-,4-1-,7-2-,14-4-,14-11+
B-139	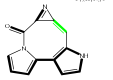 $C_{12}H_7N_3O$	22.1829	377.2852	-2.7719	77.4439	504.5369	InChI=1S/C12H7N3O/c16-12-11-9(14-11)6-8-7(3-4-13-8)10-2-1-5-15(10)12/h1-6,13H/b9-6+
B-256	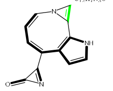 $C_{12}H_7N_3O$	11.6134	449.5147	-9.6907	24.7796	506.5129	InChI=1S/C12H7N3O/c16-12-11(14-12)7-2-1-5-15-6-9(15)10-8(7)3-4-13-10/h1-6,13H/b5-1-,7-2+
q-28	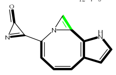 $C_{12}H_7N_3O$	11.9572	450.3436	-9.8034	23.2338	506.9304	InChI=1S/C12H7N3O/c16-12-11(14-12)8-3-1-2-7-4-5-13-10(7)9-6-15(8)9/h1-6,13H/b2-1-,8-3-
A2-46	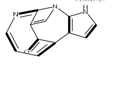 $C_{12}H_7N_3O$	41.078	189.4408	-1.688	175.4447	507.3965	InChI=1S/C12H7N3O/c16-10-7-2-1-4-13-12-9(10)6-15(12)11-8(7)3-5-14-11/h1-6,14H/b4-1-,7-2-,13-12+
F-47	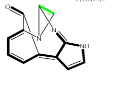 $C_{12}H_7N_3O$	30.4995	346.4942	-7.6898	63.4116	507.5261	InChI=1S/C12H7N3O/c16-11-7-2-1-3-9(11)15-6-10(15)14-12-8(7)4-5-13-12/h1-6H,(H,13,14)/b8-7-
A-26	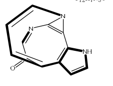 $C_{12}H_7N_3O$	17.2088	313.7283	-9.8066	129.7395	508.5933	InChI=1S/C12H7N3O/c16-9-6-14-12-11-10-8(3-4-13-10)7(9)2-1-5-15(11)12/h1-6,13H/b5-1-,7-2-,14-6-

B-171	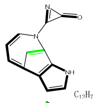	$C_{12}H_7N_3O$	7.6313	370.6679	-3.3594	107.5132	508.6998	InChI=1S/C12H7N3O/c16-12-11(14-12)15-5-1-2-7-6-9(15)10-8(7)3-4-13-10/h1-6,13H
o-187	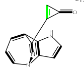	$C_{12}H_7N_3O$	44.7844	274.2274	-19.4033	168.2264	509.6677	InChI=1S/C12H7N3O/c16-10-6-8(10)11-7-2-1-5-15(14-11)9-3-4-13-12(7)9/h1-6,13H
B-157	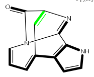	$C_{12}H_7N_3O$	64.6153	131.3070	-2.9553	207.3351	510.2658	InChI=1S/C12H7N3O/c16-12-9-6-7-2-1-5-15(12)11(14-9)10-8(7)3-4-13-10/h1-6,13H/b5-1-,7-2+
q-48	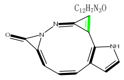	$C_{12}H_7N_3O$	28.9304	422.7677	-18.5593	59.0598	510.6603	InChI=1S/C12H7N3O/c16-12-10-3-1-2-7-4-5-13-11(7)8-6-9(8)14-15(10)12/h1-6,13H/b2-1-,10-3-,14-9-
C-56	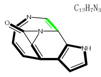	$C_{12}H_7N_3O$	24.1269	291.8084	-9.1694	156.8045	511.7807	InChI=1S/C12H7N3O/c16-12-11-8-2-1-4-13-6-9(15(11)12)10-7(8)3-5-14-10/h1-6,14H/b2-1-,9-6-,13-4-
H-32	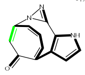	$C_{12}H_7N_3O$	17.3203	250.6551	-1.6068	221.3161	511.793	InChI=1S/C12H7N3O/c16-10-6-7-2-1-3-8(10)9-4-5-13-11(9)12-14-15(7)12/h1-6,13H
B-207	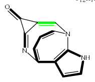	$C_{12}H_7N_3O$	3.3676	314.4998	-2.0115	172.9804	513.2982	InChI=1S/C12H7N3O/c16-11-8-6-15-5-1-2-9(14-10(8)11)7-3-4-13-12(7)15/h1-6,13H/b8-6+,14-10+
o-66	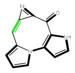	$C_{12}H_7N_3O$	26.9378	322.9294	-7.3883	117.0577	514.0534	InChI=1S/C12H7N3O/c16-12-10-8(14-10)6-7-2-1-5-15(7)9-3-4-13-11(9)12/h1-6,13H/b8-6+
B-243	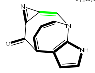	$C_{12}H_7N_3O$	5.3702	309.6318	-2.5067	172.2856	514.3673	InChI=1S/C12H7N3O/c16-11-7-2-1-5-15(6-9-10(11)14-9)12-8(7)3-4-13-12/h1-6,13H/b9-6+
E-30	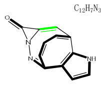	$C_{12}H_7N_3O$	14.3732	348.9453	-7.2207	108.6946	514.3833	InChI=1S/C12H7N3O/c16-12-10-6-7-2-1-3-9(14-15(10)12)8-4-5-13-11(7)8/h1-6,13H/b10-6-,14-9+
H-137	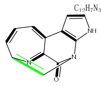	$C_{12}H_7N_3O$	23.3864	228.3339	-7.2017	183.9421	514.6981	InChI=1S/C12H7N3O/c16-12-10-8-3-1-2-7(14-10)6-15(12)11-9(8)4-5-13-11/h1-6,13H
A-6	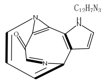	$C_{12}H_7N_3O$	17.5678	320.6604	-7.3681	132.9228	517.1849	InChI=1S/C12H7N3O/c16-9-6-15-8-2-1-5-14-11(9)12(14,15)10-7(8)3-4-13-10/h1-6,13H
A-15	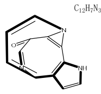	$C_{12}H_7N_3O$	17.5698	320.6424	-7.3694	132.9357	517.1849	InChI=1S/C12H7N3O/c16-9-6-14-8-2-1-5-15-11(9)12(15)10-7(8)3-4-13-10/h1-6,13H/b5-1-,8-2-,14-6-
H-136	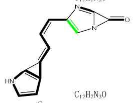	$C_{12}H_7N_3O$	32.2381	410.5806	-18.6836	85.3755	517.428	InChI=1S/C12H7N3O/c16-12-11-14-7(6-15(11)12)2-1-3-8-9-4-5-13-10(8)9/h1-6,13H/b2-1-,8-3-
o-169	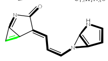	$C_{12}H_7N_3O$	44.0592	390.8025	-26.8	72.7839	517.4701	InChI=1S/C12H7N3O/c16-12-7(8-6-9(8)14-12)2-1-5-15-10-3-4-13-11(10)15/h1-6,13H/b5-1-,7-2+
H-5	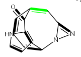	$C_{12}H_7N_3O$	19.8938	224.9747	-10.5674	239.9182	518.1268	InChI=1S/C12H7N3O/c16-12-7-2-1-3-9(15-10(6-7)14-15)8-4-5-13-11(8)12/h1-6,13H/b2-1-,7-6-,9-3+
C-4	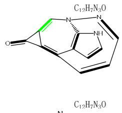	$C_{12}H_7N_3O$	21.1163	289.4894	-9.0025	174.9886	518.8057	InChI=1S/C12H7N3O/c16-11-9-6-15-12-8(3-5-13-12)7(10(9)11)2-1-4-14-15/h1-6,13H/b2-1-,14-4-
B-247	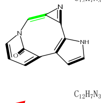	$C_{12}H_7N_3O$	7.1070	401.5440	-5.4404	86.0725	519.4280	InChI=1S/C12H7N3O/c16-12-8-2-1-5-15(12)6-9-11(14-9)10-7(8)3-4-13-10/h1-6,13H/b9-6+
x4-16	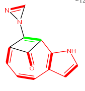	$C_{12}H_7N_3O$	34.7057	279.4893	-2.4058	195.3734	519.4702	InChI=1S/C12H7N3O/c16-12-8-3-1-2-7-4-5-13-10(7)9(12)11(8)15-6-14-15/h1-6,13H/b2-1-,3-1-,7-2-,8-3-,10-9+

H-112	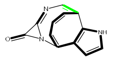 $C_{12}H_7N_3O$	10.7647	336.0059	-5.4077	140.1532	519.9452	InChI=1S/C12H7N3O/c16-12-11-14-6-7-2-1-3-9(15(11)12)8-4-5-13-10(7)8/h1-6,13H/b7-6?,14-11-
n-48	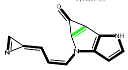 $C_{12}H_7N_3O$	33.9968	481.4187	-14.0268	14.1138	519.964	InChI=1S/C12H7N3O/c16-12-9-10-8(3-4-13-10)15(11(9)12)5-1-2-7-6-14-7/h1-6,13H/b5-1-,7-2-
A1-23	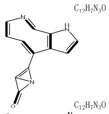 $C_{12}H_7N_3O$	31.5605	402.3908	-18.4958	66.3832	520.0129	InChI=1S/C12H7N3O/c16-12-11-10(15(11)12)8-2-1-4-13-6-9-7(8)3-5-14-9/h1-6,14H/b2-1+,4-1-,8-2-,8-7+,9-6-,13-4-,13-6+
n-170	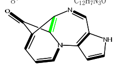 $C_{12}H_7N_3O$	60.464	209.6654	-15.6087	176.7022	520.2091	InChI=1S/C12H7N3O/c16-12-7-2-1-5-15-9-3-4-13-8(9)6-14-10(7)11(12)15/h1-6,13H
B-6	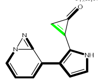 $C_{12}H_7N_3O$	56.7353	335.7516	-19.2223	121.9497	520.2093	InChI=1S/C12H7N3O/c16-10-6-9(10)11-7(3-4-13-11)8-2-1-5-15-12(8)14-15/h1-6,13H
H-2	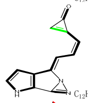 $C_{12}H_7N_3O$	59.78	337.2705	-22.8093	115.4289	520.6033	InChI=1S/C12H7N3O/c16-10-6-7(10)2-1-3-9-8-4-5-13-11(8)12-14-15(9)12/h1-6,13H/b2-1-,9-3-
x4-28	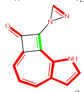 $C_{12}H_7N_3O$	42.0517	430.0337	-3.6306	26.2637	520.7946	InChI=1S/C12H7N3O/c16-12-8-3-1-2-7-4-5-13-10(7)9(8)11(12)15-6-14-15/h1-6,13H
I-68	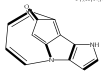 $C_{12}H_7N_3O$	24.167	227.172	-7.7117	204.3085	520.902	InChI=1S/C12H7N3O/c16-9-6-8-10-11-7(2-4-14-11)15(8)5-1-3-13-12(9)10/h1-6,14H/b5-1-,13-3-
q11-3	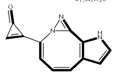 $C_{12}H_7N_3O$	40.534	470.4479	-19.0189	14.1099	521.1795	InChI=1S/C12H7N3O/c16-10-6-8(10)9-3-1-2-7-4-5-13-11(7)12-14-15(9)12/h1-6,13H/b2-1-,9-3-
G1-39	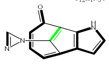 $C_{12}H_7N_3O$	7.6569	380.1387	-3.6516	114.9553	522.74	InChI=1S/C12H7N3O/c16-9-3-1-2-8-7-4-5-13-11(7)10(9)12(8)15-6-14-15/h1-6,13H
o-105	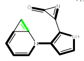 $C_{12}H_7N_3O$	12.382	474.8578	-7.5714	25.4591	522.8668	InChI=1S/C12H7N3O/c16-12-11(14-12)10-8(3-4-13-10)15-5-1-2-7-6-9(7)15/h1-6,13H
A-116	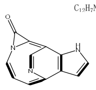 $C_{12}H_7N_3O$	7.5023	357.8306	-4.3312	145.4798	523.2895	InChI=1S/C12H7N3O/c16-12-11-8-6-14-9(2-1-5-15(11)12)7-3-4-13-10(7)8/h1-6,13H/b5-1-,9-2-,11-8+
F-58	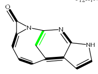 $C_{12}H_7N_3O$	14.4809	368.1225	-8.5212	123.0395	524.4219	InChI=1S/C12H7N3O/c16-12-9-3-1-2-7-6-10(15(9)12)14-11-8(7)4-5-13-11/h1-6H,(H,13,14)/b2-1-,9-3-
x5-38	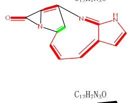 $C_{12}H_7N_3O$	20.5419	358.1564	-9.9801	131.5315	525.2484	InChI=1S/C12H7N3O/c16-12-10-9-6-8(15(10)12)3-1-2-7-4-5-13-11(7)14-9/h1-6H,(H,13,14)/b3-1-,7-2-
H11-27	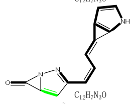 $C_{12}H_7N_3O$	34.3787	417.4561	-20.222	82.4347	526.6918	InChI=1S/C12H7N3O/c16-12-10-6-7(14-15(10)12)2-1-3-8-9-4-5-13-11(8)9/h1-6,13H/b2-1-,8-3-
o-245	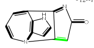 $C_{12}H_7N_3O$	23.8471	243.6354	-9.2964	199.7692	526.8428	InChI=1S/C12H7N3O/c16-10-6-9-12(14-10)7-2-1-5-15(9)8-3-4-13-11(7)8/h1-6,13H
x4-13	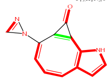 $C_{12}H_7N_3O$	56.7995	407.2539	-13.8339	53.8484	528.177	InChI=1S/C12H7N3O/c16-12-9-8(15-6-14-15)3-1-2-7-4-5-13-11(7)10(9)12/h1-6,13H/b2-1-,3-1-,7-2?,8-3+,9-8-,11-10-
E-19	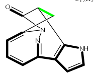 $C_{12}H_7N_3O$	22.5443	252.6839	-2.945	158.5938	528.2917	InChI=1S/C12H7N3O/c16-12-9-6-7-2-1-3-10(14-15(7)12)8-4-5-13-11(8)9/h1-6,13H
x9-32	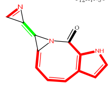 $C_{12}H_7N_3O$	49.3841	442.7775	-20.0128	33.2467	529.217	InChI=1S/C12H7N3O/c16-12-10-7(4-5-13-10)2-1-3-9-11(15(9)12)8-6-14-8/h1-6,13H/b2-1-,9-3-,11-8-

A-181	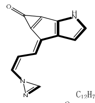 $C_{12}H_7N_3O$	64.5008	422.1344	-12.8372	50.0416	529.5293	InChI=1S/C12H7N3O/c16-12-9-7(2-1-5-15-6-14-15)8-3-4-13-11(8)10(9)12/h1-6,13H/b5-1-,7-2-
A-184	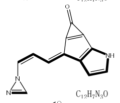 $C_{12}H_7N_3O$	64.4956	422.1389	-12.8387	50.0417	529.5293	InChI=1S/C12H7N3O/c16-12-9-7(2-1-5-15-6-14-15)8-3-4-13-11(8)10(9)12/h1-6,13H/b5-1-,7-2-
s11-15	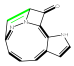 $C_{12}H_7N_3O$	27.8887	218.0268	-3.4304	186.8062	530.7711	InChI=1S/C12H7N3O/c16-12-9-6-8-3-1-2-7-4-5-13-10(7)11(12)15(9)14-8/h1-6,13H/b3-1-,7-2-,11-10+
x3-18	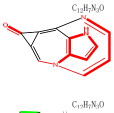 $C_{12}H_7N_3O$	17.1198	237.5195	-6.6959	252.472	530.8629	InChI=1S/C12H7N3O/c16-12-7-6-15-5-1-3-13-11(9(7)12)10-8(15)2-4-14-10/h1-6,14H/b5-1-,13-3-
o-88	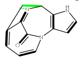 $C_{12}H_7N_3O$	74.5028	174.0144	-0.8599	173.7279	531.1528	InChI=1S/C12H7N3O/c16-12-10-7-2-1-5-15(12)9-3-4-13-11(9)8(6-7)14-10/h1-6,13H
X22-61	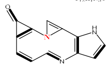 $C_{12}H_7N_3O$	15.0096	504.5486	-5.7686	6.5529	531.4198	InChI=1S/C12H7N3O/c16-12-7-2-1-4-13-8-3-5-14-10(8)9-6-15(9)11(7)12/h1-6,14H/b2-1-,13-4+
o-189	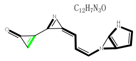 $C_{12}H_7N_3O$	85.2448	407.7816	-50.4001	59.1498	531.5637	InChI=1S/C12H7N3O/c16-10-6-7(10)11-8(14-11)2-1-5-15-9-3-4-13-12(9)15/h1-6,13H/b5-1-,8-2+
q-31	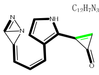 $C_{12}H_7N_3O$	39.5672	431.4769	-23.3095	69.0953	532.4619	InChI=1S/C12H7N3O/c16-10-6-8(10)11-7(4-5-13-11)2-1-3-9-12-14-15(9)12/h1-6,13H/b2-1-,9-3-
i-1	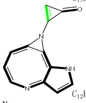 $C_{12}H_7N_3O$	55.7208	336.1653	-27.4489	115.9848	533.5404	InChI=1S/C12H7N3O/c16-10-6-9(10)15-8-2-1-4-13-7-3-5-14-11(7)12(8)15/h1-6,14H/b2-1-,4-1-,8-2+,12-11-,13-4-,13-7-
B-25	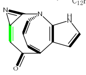 $C_{12}H_7N_3O$	14.0538	283.2203	-1.0748	210.5397	533.8624	InChI=1S/C12H7N3O/c16-10-6-9-12(14-9)15-5-1-2-7(10)8-3-4-13-11(8)15/h1-6,13H/b9-6+
o-43	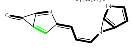 $C_{12}H_7N_3O$	29.1812	362.5097	-21.6032	134.2685	534.3421	InChI=1S/C12H7N3O/c16-11-8-6-7(14-10(8)11)2-1-5-15-9-3-4-13-12(9)15/h1-6,13H/b5-1-,7-2+
H11-52	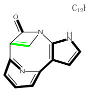 $C_{12}H_7N_3O$	35.4676	314.0187	-9.3026	121.1356	535.6238	InChI=1S/C12H7N3O/c16-12-8-6-15(12)11-7(4-5-13-11)9-2-1-3-10(8)14-9/h1-6,13H
o-142	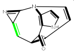 $C_{12}H_7N_3O$	13.9255	266.7158	-5.9232	212.1313	536.8329	InChI=1S/C12H7N3O/c16-11-7-2-1-5-15(12-8(6-7)14-12)9-3-4-13-10(9)11/h1-6,13H/b5-1-,7-2-,8-6+
H-120	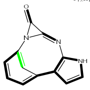 $C_{12}H_7N_3O$	19.0317	379.7294	-10.7438	106.8113	537.0635	InChI=1S/C12H7N3O/c16-12-11-14-10-9(4-5-13-10)7-2-1-3-8(6-7)15(11)12/h1-6,13H/b14-11-
B-56	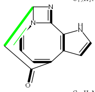 $C_{12}H_7N_3O$	68.3263	106.8393	-6.4000	241.3061	537.4364	InChI=1S/C12H7N3O/c16-9-6-10-14-12-11-8(3-4-13-11)7(9)2-1-5-15(10)12/h1-6,13H/b5-1-,7-2+,10-6-
H11-157	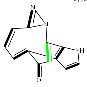 $C_{12}H_7N_3O$	6.2378	358.4387	-3.436	152.0061	537.6199	InChI=1S/C12H7N3O/c16-10-6-9-12-8(4-5-13-12)7(10)2-1-3-11-14-15(9)11/h1-6,13H/b3-1-,7-2+
o-92	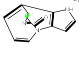 $C_{12}H_7N_3O$	6.1719	420.607	-4.4464	95.1141	537.8444	InChI=1S/C12H7N3O/c16-12-11-8(14-11)6-7-2-1-5-15(12)9-3-4-13-10(7)9/h1-6,13H/b8-6-
A1-38	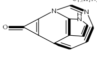 $C_{12}H_7N_3O$	33.4126	330.8115	-13.968	152.4089	538.5182	InChI=1S/C12H7N3O/c16-11-9-7-2-1-4-13-6-15(10(9)11)12-8(7)3-5-14-12/h1-6,14H/b4-1-,7-2+,13-6-
H-142	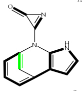 $C_{12}H_7N_3O$	11.7763	376.0464	-4.6876	121.8824	539.0595	InChI=1S/C12H7N3O/c16-12-11(14-12)15-8-3-1-2-7(6-8)9-4-5-13-10(9)15/h1-6,13H

q11-22	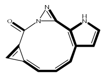 $C_{12}H_7N_3O$	25.5728	443.8257	-8.0346	57.4771	539.152	InChI=1S/C12H7N3O/c16-12-9-6-8(9)3-1-2-7-4-5-13-10(7)11-14-15(11)12/h1-6,13H/b2-1-,8-3-
X22-37	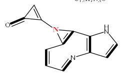 $C_{12}H_7N_3O$	49.0223	385.9611	-22.7423	84.8135	539.7301	InChI=1S/C12H7N3O/c16-10-6-9(10)15-8-2-1-4-13-7-3-5-14-11(7)12(8)15/h1-6,14H/b2-1-,4-1-,8-2+,12-11-,13-4+,13-7+
o-153	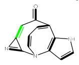 $C_{12}H_7N_3O$	11.8654	286.9106	-4.4862	202.8484	539.9066	InChI=1S/C12H7N3O/c16-10-6-8-12(14-8)15-5-1-2-7(10)11-9(15)3-4-13-11/h1-6,13H/b8-6+
o-8	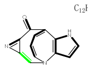 $C_{12}H_7N_3O$	5.9099	313.5441	-2.8623	192.6171	540.2982	InChI=1S/C12H7N3O/c16-12-7-2-1-5-15(6-8-11(12)14-8)9-3-4-13-10(7)9/h1-6,13H/b8-6+
B-246	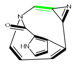 $C_{12}H_7N_3O$	20.4189	277.7493	-7.3434	212.1976	543.0819	InChI=1S/C12H7N3O/c16-12-11-8(3-4-13-11)7-2-1-5-15(12)6-9-10(7)14-9/h1-6,13H/b5-1-,7-2+,9-6+
H11-4	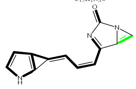 $C_{12}H_7N_3O$	42.272	449.3267	-27.8614	49.5383	544.3496	InChI=1S/C12H7N3O/c16-12-14-9(10-6-15(10)12)3-1-2-7-8-4-5-13-11(7)8/h1-6,13H/b3-1-,7-2-
q11-42	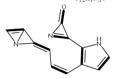 $C_{12}H_7N_3O$	21.6786	461.2642	-18.89	67.5798	544.5193	InChI=1S/C12H7N3O/c16-12-11(14-12)10-7(4-5-13-10)2-1-3-8-9-6-15(8)9/h1-6,13H/b2-1-,8-3-
H-17	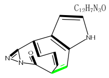 $C_{12}H_7N_3O$	27.5576	284.4669	-9.3889	181.4634	545.1328	InChI=1S/C12H7N3O/c16-12-7-2-1-3-9(11-14-15(11)12)8-4-5-13-10(8)6-7/h1-6,13H/b2-1-,3-1+,7-2-,7-6+,9-3+,9-8+,10-6+
H11-24	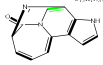 $C_{12}H_7N_3O$	35.4996	184.506	0.2029	215.4248	546.5195	InChI=1S/C12H7N3O/c16-12-8-2-1-3-10-7-4-5-13-11(7)9(14-8)6-15(10)12/h1-6,13H
n-167	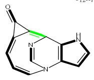 $C_{12}H_7N_3O$	21.7644	312.0941	-8.1315	183.8997	546.5702	InChI=1S/C12H7N3O/c16-12-7-2-1-5-15-6-14-11(9(7)12)10-8(15)3-4-13-10/h1-6,13H/b5-1-,7-2+,11-9+
x5-41	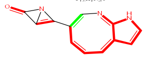 $C_{12}H_7N_3O$	36.1623	408.9338	-22.5447	82.6439	546.6424	InChI=1S/C12H7N3O/c16-12-10-9(15(10)12)8-3-1-2-7-4-5-13-11(7)14-6-8/h1-6H,(H,13,14)
B-250	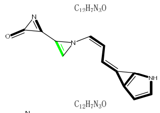 $C_{12}H_7N_3O$	55.8257	427.8550	-32.4853	58.7815	546.7424	InChI=1S/C12H7N3O/c16-12-11(14-12)9-6-15(9)5-1-2-7-8-3-4-13-10(7)8/h1-6,13H/b5-1-,7-2-
B-7	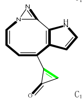 $C_{12}H_7N_3O$	53.1996	384.3254	-24.9124	100.6858	547.7187	InChI=1S/C12H7N3O/c16-10-6-9(10)7-2-1-5-15-12(14-15)11-8(7)3-4-13-11/h1-6,13H/b5-1-,7-2+
H-46	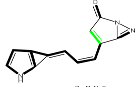 $C_{12}H_7N_3O$	29.6639	423.367	-18.102	100.504	547.8796	InChI=1S/C12H7N3O/c16-10-6-7(12-14-15(10)12)2-1-3-8-9-4-5-13-11(8)9/h1-6,13H/b2-1-,8-3-
H-1	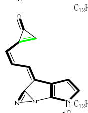 $C_{12}H_7N_3O$	28.2799	432.948	-7.1062	89.735	548.8865	InChI=1S/C12H7N3O/c16-10-6-7(10)2-1-3-8-9-4-5-13-11(9)15-12(8)14-15/h1-6,13H/b2-1-,8-3-
B-205	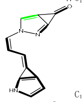 $C_{12}H_7N_3O$	28.3325	416.2083	-14.2854	101.2601	549.5034	InChI=1S/C12H7N3O/c16-12-9-6-15(14-11(9)12)5-1-2-7-8-3-4-13-10(7)8/h1-6,13H/b5-1-,7-2-
H11-131	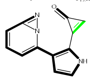 $C_{12}H_7N_3O$	63.209	328.2761	-21.4668	153.2239	550.2088	InChI=1S/C12H7N3O/c16-10-6-8(10)12-7(4-5-13-12)9-2-1-3-11-14-15(9)11/h1-6,13H
H-63	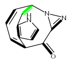 $C_{12}H_7N_3O$	7.6693	312.2742	-3.6849	200.6819	551.8636	InChI=1S/C12H7N3O/c16-11-9-3-1-2-7(15-12(11)14-15)6-10-8(9)4-5-13-10/h1-6,13H/b2-1-,3-1-,7-2+,7-6-,9-3+,9-8+,10-6-
H-140	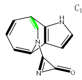 $C_{12}H_7N_3O$	8.8495	381.2359	-4.4102	132.551	552.0011	InChI=1S/C12H7N3O/c16-12-11(14-12)15-6-7-2-1-3-9(15)8-4-5-13-10(7)8/h1-6,13H

j-19	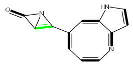	$C_{12}H_7N_3O$	33.8998	405.9618	-20.0515	90.9701	553.6468	InChI=1S/C12H7N3O/c16-12-11-10(15(11)12)7-2-1-4-13-8-3-5-14-9(8)6-7/h1-6,14H/b2-1-,4-1-,7-2+,7-6+,9-6+,13-4-,13-8-
B-9	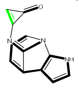	$C_{12}H_7N_3O$	47.0158	313.7818	-20.3052	177.3003	553.7674	InChI=1S/C12H7N3O/c16-10-6-9(10)14-12-7-2-1-5-15(12)11-8(7)3-4-13-11/h1-6,13H/b14-12-
H-145	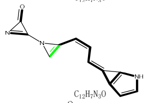	$C_{12}H_7N_3O$	61.9606	436.9493	-39.0581	62.4658	554.1098	InChI=1S/C12H7N3O/c16-12-11(14-12)15-6-7(15)2-1-3-8-9-4-5-13-10(8)9/h1-6,13H/b2-1-,8-3-
H1-18	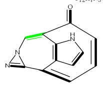	$C_{12}H_7N_3O$	23.0563	255.0699	-3.52	234.5017	554.4645	InChI=1S/C12H7N3O/c16-10-3-1-2-8-7-4-5-13-11(7)9(10)6-15-12(8)14-15/h1-6,13H/b3-1-,8-2+
H11-129	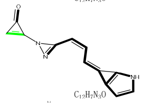	$C_{12}H_7N_3O$	92.9968	423.2239	-41.5388	67.8374	556.566	InChI=1S/C12H7N3O/c16-10-6-9(10)15-11(14-15)3-1-2-7-8-4-5-13-12(7)8/h1-6,13H/b3-1-,7-2-
o-41	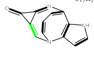	$C_{12}H_7N_3O$	7.6221	327.8079	-2.2259	197.6619	562.4258	InChI=1S/C12H7N3O/c16-12-7-6-15-5-1-2-8(14-10(7)12)11-9(15)3-4-13-11/h1-6,13H/b7-6+,14-10+
q1-59	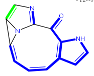	$C_{12}H_7N_3O$	35.2977	250.999	-7.1099	184.1047	562.4889	InChI=1S/C12H7N3O/c16-11-10-7(4-5-13-10)2-1-3-8-9-6-14-12(11)15(8)9/h1-6,13H/b2-1-,8-3-
q1-60	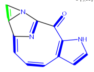	$C_{12}H_7N_3O$	35.2955	251.0004	-7.1093	184.1049	562.4889	InChI=1S/C12H7N3O/c16-11-10-7(4-5-13-10)2-1-3-8-9-6-15(9)12(11)14-8/h1-6,13H/b2-1-,8-3-
q-9	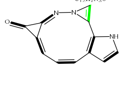	$C_{12}H_7N_3O$	5.3794	510.7308	-4.4858	39.0562	565.5103	InChI=1S/C12H7N3O/c16-12-8-3-1-2-7-4-5-13-10(7)9-6-15(9)14-11(8)12/h1-6,13H/b2-1-,8-3+,14-11+
x4-65	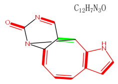	$C_{12}H_7N_3O$	118.5101	117.6217	0.7239	121.6109	567.3897	InChI=1S/C12H7N3O/c16-12-14-6-8-9-3-1-2-7-4-5-13-10(7)11(8)15(9)12/h1-6,13H/b2-1-,3-1-,7-2-,9-3+,11-10+
H11-153	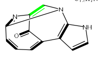	$C_{12}H_7N_3O$	14.8429	212.3338	-5.0959	286.9912	567.9757	InChI=1S/C12H7N3O/c16-11-7-2-1-3-10-14-9(11)6-15(10)12-8(7)4-5-13-12/h1-6,13H/b3-1-,7-2-
H11-138	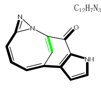	$C_{12}H_7N_3O$	31.0733	314.8555	-2.351	180.7329	569.5849	InChI=1S/C12H7N3O/c16-12-9-6-7(8-4-5-13-11(8)12)2-1-3-10-14-15(9)10/h1-6,13H/b3-1-,7-2+
B-100	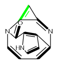	$C_{12}H_7N_3O$	25.3840	366.0297	-12.3389	120.5242	570.2147	InChI=1S/C12H7N3O/c16-12-11-7(3-4-13-11)8-2-1-5-15(12)10-6-9(10)14-8/h1-6,13H/b5-1-,8-2+,14-9-
H-79	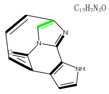	$C_{12}H_7N_3O$	27.2404	204.4452	-0.9662	255.4377	574.6805	InChI=1S/C12H7N3O/c16-12-9-3-1-2-7-6-15(12)11(14-7)10-8(9)4-5-13-10/h1-6,13H/b2-1-,9-3-
o-195	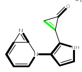	$C_{12}H_7N_3O$	57.2798	363.0401	-24.4922	148.6	575.2101	InChI=1S/C12H7N3O/c16-10-6-7(10)11-9(3-4-13-11)15-5-1-2-8-12(15)14-8/h1-6,13H
H-146	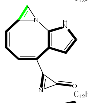	$C_{12}H_7N_3O$	37.6185	391.4367	-18.3924	119.1527	576.3995	InChI=1S/C12H7N3O/c16-12-10(14-12)8-3-1-2-7-6-15(7)11-9(8)4-5-13-11/h1-6,13H/b2-1-,8-3+
o-84	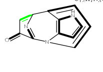	$C_{12}H_7N_3O$	35.6083	234.1044	-7.3865	229.8095	578.4437	InChI=1S/C12H7N3O/c16-10-6-8-7-2-1-5-15(12(10)14-8)9-3-4-13-11(7)9/h1-6,13H
q1-52	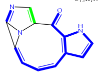	$C_{12}H_7N_3O$	52.0835	230.2145	-9.4445	203.4246	578.555	InChI=1S/C12H7N3O/c16-11-9-6-14-12-8(15(9)12)3-1-2-7-4-5-13-10(7)11/h1-6,13H/b2-1-,8-3-
B-166	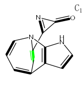	$C_{12}H_7N_3O$	8.3668	361.1655	-5.0258	180.9595	579.4220	InChI=1S/C12H7N3O/c16-12-10(14-12)9-6-7-2-1-5-15(9)11-8(7)3-4-13-11/h1-6,13H

o-103	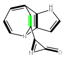	$C_{12}H_7N_3O$	9.2134	389.6344	-4.4371	151.8766	580.9726	InChI=1S/C12H7N3O/c16-12-11(14-12)9-6-7-2-1-5-15(9)8-3-4-13-10(7)8/h1-6,13H
o-100	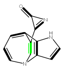	$C_{12}H_7N_3O$	9.2659	389.4437	-4.4386	152.8028	581.6421	InChI=1S/C12H7N3O/c16-12-11(14-12)8-6-15-5-1-2-7(8)10-9(15)3-4-13-10/h1-6,13H
H11-135	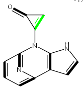	$C_{12}H_7N_3O$	56.9281	338.4564	-21.3536	151.2871	581.8178	InChI=1S/C12H7N3O/c16-10-6-9(10)15-11-3-1-2-8(14-11)7-4-5-13-12(7)15/h1-6,13H
G-28	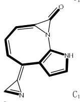	$C_{12}H_7N_3O$	7.2136	559.1289	-6.823	19.4606	582.526	InChI=1S/C12H7N3O/c16-12-10-3-1-2-7(9-6-14-9)8-4-5-13-11(8)15(10)12/h1-6,13H/b2-1-,9-7-,10-3-
o-33	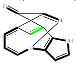	$C_{12}H_7N_3O$	29.128	205.1976	-1.6662	258.6443	582.7638	InChI=1S/C12H7N3O/c16-12-8-6-7-2-1-5-15(14-10(7)12)9-3-4-13-11(8)9/h1-6,13H
o-194	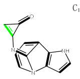	$C_{12}H_7N_3O$	47.5457	318.1315	-20.0676	201.4043	584.923	InChI=1S/C12H7N3O/c16-10-6-8(10)14-12-7-2-1-5-15(12)9-3-4-13-11(7)9/h1-6,13H/b14-12+
G-1	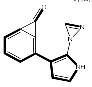	$C_{12}H_7N_3O$	4.8466	585.3746	-4.055	-8.8242	584.9703	InChI=1S/C12H7N3O/c16-11-9-3-1-2-7(10(9)11)8-4-5-13-12(8)15-6-14-15/h1-6,13H
X-29	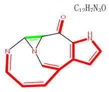	$C_{12}H_7N_3O$	46.7691	357.4902	-13.7856	109.0789	586.5769	InChI=1S/C12H7N3O/c16-11-9-8(3-5-13-9)7-2-1-4-14-12-10(11)15(12)6-7/h1-6,13H/b2-1-,14-4-
H-134	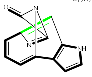	$C_{12}H_7N_3O$	8.2966	351.9734	-4.8319	197.0684	589.1494	InChI=1S/C12H7N3O/c16-12-11-14-7-2-1-3-10(15(11)12)8-4-5-13-9(8)6-7/h1-6,13H/b2-1-,3-1-,7-2-,7-6-,9-6+,10-3-,10-8-,14-11+
q11-30	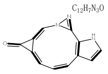	$C_{12}H_7N_3O$	23.6355	453.4107	-5.9429	102.6509	589.34	InChI=1S/C12H7N3O/c16-11-8-3-1-2-7-4-5-13-10(7)12-14-15(12)6-9(8)11/h1-6,13H/b2-1-,8-3+,9-6+
s11-24	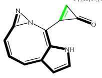	$C_{12}H_7N_3O$	25.8052	474.9788	-9.4184	72.1017	592.0316	InChI=1S/C12H7N3O/c16-9-6-8(9)12-11-7(4-5-13-11)2-1-3-10-14-15(10)12/h1-6,13H/b3-1-,7-2-,12-11+
B-158	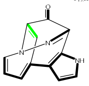	$C_{12}H_7N_3O$	112.3698	138.3066	-6.2273	214.5581	593.3360	InChI=1S/C12H7N3O/c16-12-9-6-7-2-1-5-15(9)14-11(12)10-8(7)3-4-13-10/h1-6,13H
q11-24	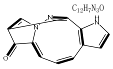	$C_{12}H_7N_3O$	119.0408	164.2401	-16.2932	174.6628	594.1339	InChI=1S/C12H7N3O/c16-12-8-6-15-9(12)3-1-2-7-4-5-13-10(7)11(8)14-15/h1-6,13H/b2-1-,9-3-
X23-5	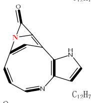	$C_{12}H_7N_3O$	17.3383	395.5576	-8.105	151.0733	594.5359	InChI=1S/C12H7N3O/c16-12-11-8-6-7(15(11)12)2-1-4-13-9-3-5-14-10(8)9/h1-6,14H/b2-1-,13-4-
A-48	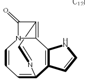	$C_{12}H_7N_3O$	110.8024	191.1679	-11.5963	141.2124	595.2185	InChI=1S/C12H7N3O/c16-12-8-6-14-9-2-1-5-15(12)11(8)10-7(9)3-4-13-10/h1-6,13H/b5-1-,9-2?
x4-39	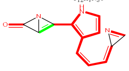	$C_{12}H_7N_3O$	28.4676	567.4099	-18.9209	11.6416	596.5025	InChI=1S/C12H7N3O/c16-12-11-10(15(11)12)9-7(4-5-13-9)2-1-3-8-6-14-8/h1-6,13H/b2-1-,8-3-
E-58	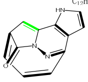	$C_{12}H_7N_3O$	56.366	304.5087	-1.6749	151.5082	596.8912	InChI=1S/C12H7N3O/c16-12-7-2-1-3-9-8-4-5-13-11(8)10(6-7)15(12)14-9/h1-6,13H/b3-1-,7-2-
F-5	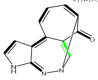	$C_{12}H_7N_3O$	116.5159	182.4432	-7.0139	169.0299	597.4067	InChI=1S/C12H7N3O/c16-11-9-6-15-10(11)3-1-2-7(9)8-4-5-13-12(8)14-15/h1-6H,(H,13,14)
s11-26	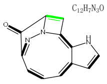	$C_{12}H_7N_3O$	41.1956	221.3895	-3.2479	221.1417	597.7032	InChI=1S/C12H7N3O/c16-12-8-3-1-2-7-4-5-13-11(7)9-6-10(12)15(9)14-8/h1-6,13H/b3-1-,7-2-,11-9+

s11-19	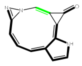 $C_{12}H_7N_3O$	32.5894	520.617	-1.6299	32.073	599.5328	InChI=1S/C12H7N3O/c16-12-8-6-15-9(14-15)3-1-2-7-4-5-13-11(7)10(8)12/h1-6,13H/b3-1-,7-2-,8-6+,11-10-
B-159	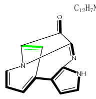 $C_{12}H_7N_3O$	106.2673	168.7315	-6.9524	173.8143	599.8938	InChI=1S/C12H7N3O/c16-10-9-6-7-2-1-5-15(9)12(10)14-11-8(7)3-4-13-11/h1-6,13H/b14-12-
A-174	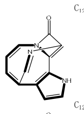 $C_{12}H_7N_3O$	110.4153	188.1457	-10.6784	140.6899	600.1554	InChI=1S/C12H7N3O/c16-12-10-11-9-8(3-4-13-9)7(6-14-10)2-1-5-15(11)12/h1-6,13H/b5-1-,7-2+
s12-1	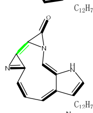 $C_{12}H_7N_3O$	59.2945	444.2272	-34.8579	86.1374	600.5944	InChI=1S/C12H7N3O/c16-12-11-10-8(14-10)3-1-2-7-4-5-13-9(7)6-15(11)12/h1-6,13H/b3-1-,7-2-,9-6-,11-10+
p-25	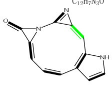 $C_{12}H_7N_3O$	31.3362	535.9802	-2.3182	31.4898	603.5707	InChI=1S/C12H7N3O/c16-12-10-3-1-2-7-4-5-13-8(7)6-9-11(14-9)15(10)12/h1-6,13H/b2-1-,9-6+,10-3-
B-3	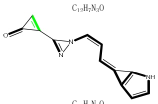 $C_{12}H_7N_3O$	80.2990	484.5911	-39.7794	56.9957	604.8136	InChI=1S/C12H7N3O/c16-10-6-9(10)12-14-15(12)5-1-2-7-8-3-4-13-11(7)8/h1-6,13H/b5-1-,7-2-
s11-41	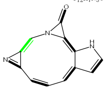 $C_{12}H_7N_3O$	32.8314	494.664	-5.1573	77.7814	605.1568	InChI=1S/C12H7N3O/c16-12-11-10-7(4-5-13-10)2-1-3-8-9(14-8)6-15(11)12/h1-6,13H/b3-1-,7-2-,9-6+,11-10+
s12-38	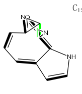 $C_{12}H_7N_3O$	63.5595	452.6179	-37.5274	77.6939	605.233	InChI=1S/C12H7N3O/c16-12-11-10-8(14-10)3-1-2-7-4-5-13-9(7)6-15(11)12/h1-6,13H/b3-1-,7-2-,9-6-,11-10-
x3-16	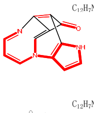 $C_{12}H_7N_3O$	81.1163	131.415	-6.0587	252.8722	606.6167	InChI=1S/C12H7N3O/c16-12-7-6-15-5-1-3-13-10(7)9(12)11-8(15)2-4-14-11/h1-6,14H/b5-1-,13-3+
o-137	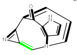 $C_{12}H_7N_3O$	10.4623	280.5907	-5.6046	266.679	607.4944	InChI=1S/C12H7N3O/c16-12-7-2-1-5-15(6-8-10(7)14-8)9-3-4-13-11(9)12/h1-6,13H/b5-1-,7-2-,8-6+
o-138	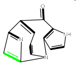 $C_{12}H_7N_3O$	10.4623	280.5907	-5.6046	266.679	607.4944	InChI=1S/C12H7N3O/c16-12-7-2-1-5-15(10-6-8(7)14-10)9-3-4-13-11(9)12/h1-6,13H/b5-1-,7-2+
H-81	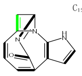 $C_{12}H_7N_3O$	83.8146	132.7448	-2.0764	218.0375	607.5426	InChI=1S/C12H7N3O/c16-12-9-3-1-2-7-6-10(14-12)15(7)11-8(9)4-5-13-11/h1-6,13H/b2-1-,9-3-
x4-49	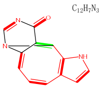 $C_{12}H_7N_3O$	100.7238	78.9635	-1.278	208.6361	609.2112	InChI=1S/C12H7N3O/c16-12-9-8-3-1-2-7-4-5-13-10(7)11(9)15(8)6-14-12/h1-6,13H/b2-1-,3-1-,7-2-,8-3+,11-10+
o-83	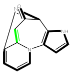 $C_{12}H_7N_3O$	83.1147	155.1226	-2.4533	209.5822	610.4506	InChI=1S/C12H7N3O/c16-10-6-9-7-2-1-5-15(9)8-3-4-13-11(8)12(10)14-7/h1-6,13H
q11-57	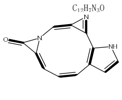 $C_{12}H_7N_3O$	19.3409	484.0376	-7.1388	100.4018	611.173	InChI=1S/C12H7N3O/c16-12-9-3-1-2-7-4-5-13-10(7)11-8(14-11)6-15(9)12/h1-6,13H/b2-1-,8-6+,9-3-
o-115	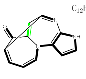 $C_{12}H_7N_3O$	121.1788	137.1193	-12.1887	211.5004	613.1668	InChI=1S/C12H7N3O/c16-11-7-2-1-5-15-9-3-4-13-12(9)14-8(7)6-10(11)15/h1-6,13H
p-19	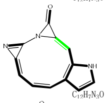 $C_{12}H_7N_3O$	36.3524	542.6912	-1.6868	30.967	614.5603	InChI=1S/C12H7N3O/c16-12-10-6-9-7(4-5-13-9)2-1-3-8-11(14-8)15(10)12/h1-6,13H/b2-1-,8-3+,10-6-
n-160	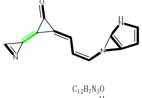 $C_{12}H_7N_3O$	37.4044	527.7858	-21.214	47.597	616.1988	InChI=1S/C12H7N3O/c16-11-7(10(11)8-6-14-8)2-1-5-15-9-3-4-13-12(9)15/h1-6,13H/b5-1-,7-2+,10-8+
A2-64	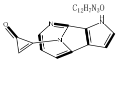 $C_{12}H_7N_3O$	18.8474	458.7429	-4.8857	120.3507	617.7947	InChI=1S/C12H7N3O/c16-10-6-9(10)15-8-2-1-4-14-12(15)11-7(8)3-5-13-11/h1-6,13H

n-159	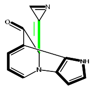	$C_{12}H_7N_3O$	9.8923	373.3664	-4.988	200.8884	618.5545	InChI=1S/C12H7N3O/c16-12-7-2-1-5-15(11(12)8-6-14-8)9-3-4-13-10(7)9/h1-6,13H/b11-8-
G-53	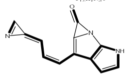	$C_{12}H_7N_3O$	21.7228	540.4176	-14.1698	72.5792	622.4466	InChI=1S/C12H7N3O/c16-12-10-8(3-1-2-7-6-14-7)9-4-5-13-11(9)15(10)12/h1-6,13H/b3-1-,7-2-
x4-55	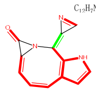	$C_{12}H_7N_3O$	35.9644	497.5163	-6.2288	88.2876	625.1611	InChI=1S/C12H7N3O/c16-12-9-3-1-2-7-4-5-13-10(7)11(15(9)12)8-6-14-8/h1-6,13H/b2-1-,9-3-,11-8-
I-28	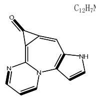	$C_{12}H_7N_3O$	20.499	413.0557	-10.8252	154.8829	626.8357	InChI=1S/C12H7N3O/c16-11-7-6-8-9(2-4-13-8)15-5-1-3-14-12(15)10(7)11/h1-6,13H
A2-60	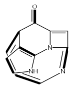	$C_{12}H_7N_3O$	181.5459	141.6323	-18.4344	149.7095	629.3483	InChI=1S/C12H7N3O/c16-11-7-2-1-4-13-10-6-9(11)15(10)12-8(7)3-5-14-12/h1-6,14H/b4-1-,7-2+,13-10-
o-135	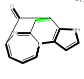	$C_{12}H_7N_3O$	51.3558	51.3558	-1.9351	263.1238	629.4345	InChI=1S/C12H7N3O/c16-12-7-6-10-14-8(12)2-1-5-15(10)9-3-4-13-11(7)9/h1-6,13H
H1-112	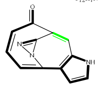	$C_{12}H_7N_3O$	51.3419	285.6661	-5.2579	152.7529	631.9066	InChI=1S/C12H7N3O/c16-11-3-1-2-10-7-4-5-13-9(7)6-8(11)12-14-15(10)12/h1-6,13H/b3-1-,10-2-
o-178	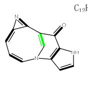	$C_{12}H_7N_3O$	7.9808	385.6085	-4.3072	200.308	632.9609	InChI=1S/C12H7N3O/c16-12-7-6-15(9-3-4-13-11(9)12)5-1-2-8-10(7)14-8/h1-6,13H/b5-1-,8-2+
H1-50	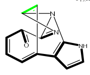	$C_{12}H_7N_3O$	161.1305	188.3019	-16.5316	137.0661	634.7372	InChI=1S/C12H7N3O/c16-9-3-1-2-7-6-10-14-12(9)15(10)11-8(7)4-5-13-11/h1-6,13H/b3-1-,7-2+
B-116	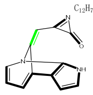	$C_{12}H_7N_3O$	11.4350	446.7503	-5.1116	152.5187	636.2027	InChI=1S/C12H7N3O/c16-12-9(14-12)6-10-7-2-1-5-15(10)11-8(7)3-4-13-11/h1-6,13H/b10-6+
H-4	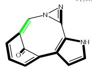	$C_{12}H_7N_3O$	50.8879	330.9189	-14.6343	209.4347	638.2767	InChI=1S/C12H7N3O/c16-11-7-2-1-3-9(11)8-4-5-13-10(8)12-14-15(12)6-7/h1-6,13H/b7-6-
A-76	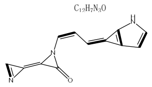	$C_{12}H_7N_3O$	46.8207	549.5131	-24.4362	53.3533	638.8785	InChI=1S/C12H7N3O/c16-12-11(9-6-14-9)15(12)5-1-2-7-8-3-4-13-10(7)8/h1-6,13H/b5-1-,7-2-,11-9+
x4-29	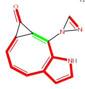	$C_{12}H_7N_3O$	33.8434	521.6337	-2.2975	79.9158	641.2281	InChI=1S/C12H7N3O/c16-12-8-3-1-2-7-4-5-13-10(7)11(9(8)12)15-6-14-15/h1-6,13H/b2-1-,3-1-,7-2?,8-3+,11-9-,11-10-
H11-130	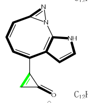	$C_{12}H_7N_3O$	30.7082	530.2823	-3.6247	81.121	646.3184	InChI=1S/C12H7N3O/c16-10-6-9(10)7-2-1-3-11-14-15(11)12-8(7)4-5-13-12/h1-6,13H/b3-1-,7-2+
o-69	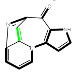	$C_{12}H_7N_3O$	88.2494	183.3953	-5.1615	258.8157	655.8837	InChI=1S/C12H7N3O/c16-12-8-6-10-7(14-8)2-1-5-15(10)9-3-4-13-11(9)12/h1-6,13H
o-192	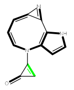	$C_{12}H_7N_3O$	45.3109	477.3198	-20.603	124.4462	658.8215	InChI=1S/C12H7N3O/c16-10-6-9(10)15-5-1-2-7-11(14-7)12-8(15)3-4-13-12/h1-6,13H/b5-1-,7-2+
o-106	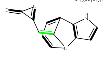	$C_{12}H_7N_3O$	12.5848	449.9503	-5.1905	169.0786	661.2269	InChI=1S/C12H7N3O/c16-12-8(14-12)6-10-7-2-1-5-15(10)9-3-4-13-11(7)9/h1-6,13H/b10-6+
B-172	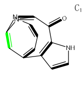	$C_{12}H_7N_3O$	113.7871	209.4162	-11.0960	187.9011	665.9597	InChI=1S/C12H7N3O/c16-11-10-8(3-4-13-10)7-2-1-5-15-9(6-7)14-12(11)15/h1-6,13H
o-209	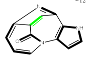	$C_{12}H_7N_3O$	156.8627	172.7787	-10.4908	195.8172	680.1633	InChI=1S/C12H7N3O/c16-12-7-6-9-11-10(3-4-13-11)15(12)5-1-2-8(7)14-9/h1-6,13H

x3-9	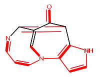 $C_{12}H_7N_3O$	81.1163	131.415	-6.0587	285.4218	689.2473	InChI=1S/C12H7N3O/c16-12-7-6-15-5-1-3-13-10(7)9(12)11-8(15)2-4-14-11/h1-6,14H/b5-1-,13-3-
x4-59	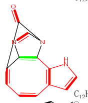 $C_{12}H_7N_3O$	147.6248	257.6234	-13.1834	138.7773	691.3369	InChI=1S/C12H7N3O/c16-12-8-3-1-2-7-4-5-13-9(7)11-10(8)14-6-15(11)12/h1-6,13H/b2-1-,3-1-,7-2-,8-3+,11-9-
o-62	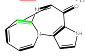 $C_{12}H_7N_3O$	175.5508	214.8686	-13.4074	185.8839	692.3203	InChI=1S/C12H7N3O/c16-12-10-7-2-1-5-15(9(6-7)14-10)8-3-4-13-11(8)12/h1-6,13H
s11-20	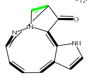 $C_{12}H_7N_3O$	171.9744	198.0843	-10.8864	174.1847	693.4302	InChI=1S/C12H7N3O/c16-12-8-6-15-11(12)10-7(4-5-13-10)2-1-3-9(8)14-15/h1-6,13H/b3-1-,7-2-,11-10+
B-108	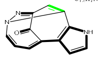 $C_{12}H_7N_3O$	71.7100	257.8296	-7.7718	236.8467	694.7424	InChI=1S/C12H7N3O/c16-12-8-2-1-5-15-10(6-9(12)14-15)11-7(8)3-4-13-11/h1-6,13H/b5-1-,8-2-
n-74	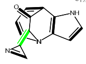 $C_{12}H_7N_3O$	11.7073	429.6718	-5.3127	208.2608	695.1251	InChI=1S/C12H7N3O/c16-12-7-2-1-5-15(11(12)8-6-14-8)9-3-4-13-10(7)9/h1-6,13H/b11-8+
p-13	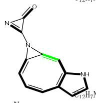 $C_{12}H_7N_3O$	12.9049	684.8786	-5.6729	-10.75	709.4234	InChI=1S/C12H7N3O/c16-12-11(14-12)15-9-3-1-2-7-4-5-13-8(7)6-10(9)15/h1-6,13H/b2-1-,3-1-,7-2?,8-6+,9-3+,10-6+
A-118	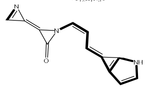 $C_{12}H_7N_3O$	58.4707	619.5777	-27.1136	52.0104	711.5618	InChI=1S/C12H7N3O/c16-12-11(9-6-14-9)15(12)5-1-2-7-8-3-4-13-10(7)8/h1-6,13H/b5-1-,7-2-,11-9-
o-219	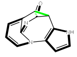 $C_{12}H_7N_3O$	87.7034	197.5438	-5.045	265.9833	722.5407	InChI=1S/C12H7N3O/c16-12-8-6-7-2-1-5-15(11(7)14-12)9-3-4-13-10(8)9/h1-6,13H
B-218	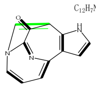 $C_{12}H_7N_3O$	76.0661	314.2668	-3.1857	248.8399	765.7155	InChI=1S/C12H7N3O/c16-11-8-6-15-5-1-2-9(14-12(11)15)7-3-4-13-10(7)8/h1-6,13H
s11-47	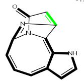 $C_{12}H_7N_3O$	195.679	281.7681	-15.7946	152.2319	776.5139	InChI=1S/C12H7N3O/c16-10-6-8-12-11-7(4-5-13-11)2-1-3-9(14-8)15(10)12/h1-6,13H/b3-1-,7-2-,12-11+
B-201	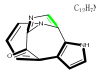 $C_{12}H_7N_3O$	170.3337	121.7425	-5.7893	263.9435	804.0994	InChI=1S/C12H7N3O/c16-11-8-2-1-5-15-9(6-14-12(11)15)10-7(8)3-4-13-10/h1-6,13H
o-31	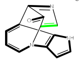 $C_{12}H_7N_3O$	155.0843	274.2628	-17.4045	306.8765	863.3481	InChI=1S/C12H7N3O/c16-12-9-6-14-11(12)7-2-1-5-15(9)8-3-4-13-10(7)8/h1-6,13H
C-11	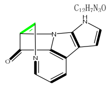 $C_{12}H_7N_3O$	162.7668	259.6384	-33.8478	369.1963	960.8567	InChI=1S/C12H7N3O/c16-11-9-6-13-4-1-2-7-8-3-5-14-12(8)15(9)10(7)11/h1-6,14H/b2-1-,9-6-,13-4?
B-259	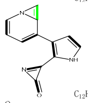 $C_{12}H_7N_3O$	194.8672	704.8841	-10.6092	76.6735	972.0031	InChI=1S/C12H7N3O/c16-12-11(14-12)10-8(3-4-13-10)7-2-1-5-15-6-9(7)15/h1-6,13H
A-92	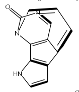 $C_{12}H_7N_3O$	296.6812	157.9234	-17.0657	157.6324	1007.5428	InChI=1S/C12H7N3O/c16-12-14-6-9-7-2-1-5-15(12)11(9)10-8(7)3-4-13-10/h1-6,13H
s11-46	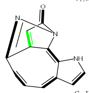 $C_{12}H_7N_3O$	484.2379	525.8648	-12.8248	230.6381	1058.2742	InChI=1S/C12H7N3O/c16-10-6-8-9-3-1-2-7-4-5-13-11(7)12(8)15(10)14-9/h1-6,13H/b3-1-,7-2-,12-11-
A2-21	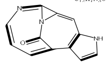 $C_{12}H_7N_3O$	732.8354	338.8582	-21.9599	304.307	1356.0904	InChI=1S/C12H7N3O/c16-12-8-2-1-4-14-11-10(15(11)12)6-9-7(8)3-5-13-9/h1-6,13H/b2-1-,4-1-,8-2-,14-4-,14-11-
o-237	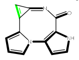 $C_{12}H_7N_3O$	238.8518	840.113	-21.3841	119.8159	1440.7624	InChI=1S/C12H7N3O/c16-12-11-10(3-4-13-11)15-5-1-2-9(15)7-6-8(7)14-12/h1-6,13H/b14-8-
