# Supplementary material for: Structural Investigation of Aaptourinamine by a Novel Module-Assembly-Based Calculation
Source: Mar Drugs. 2022 Oct 20;20(10):649. doi: 10.3390/md20100649 (PMC9604825; doi:10.3390/md20100649)
Supplement: Supplementary file 1 [file marinedrugs-20-00649-s001.zip › Supporting Information.pdf]

Supporting Information

Structural Investigation of Aaptourinamine by a Novel Module-Assembly-Based Calculation

Xing Shi [1,2,†], Zhihui Wu [1,2,†], Tianyun Jin [1,2], Cili Wang [1,2] and Pinglin Li [1,2,*]

[1]Key Laboratory of Marine Drugs, Chinese Ministry of Education, School of Medicine and Pharmacy Department, Ocean University of China, Qingdao 266003, China
[2]Laboratory of Marine Drugs and Biological Products, Pilot National Laboratory for Marine Science and Technology (Qingdao), Qingdao 266003, China

[†]These authors have contributed equally to this work and share first authorship

*Correspondence: lipinglin@ouc.edu.cn

Table of Contents

1. Methods for calculation in Dooerafa........3

2. Mata-groups and meta-structures of aaptourinamine........3

2.1 Core structures according to experimental data of aaptourinamine........3

2.2 The connection site counting principle for aaptourinamine takes A-a as an example........3

2.3 Meta-groups and meta-structures of aaptourinamine........3

3. The structural data groups for aaptourinamine........5

4. The structures converted from the data groups........21

5. Calculated detail for DFT-GIAO........23

6. Experimental NMR data of aaptourinamine........33

7. Experimental spectra of aaptourinamine........34

Reference........37

1. Methods for calculation in Dooerafa

The meta-groups were deduced on the basis of experimental data, and the meta-structures were assembled by the grafting method to make the connection points less than 14 for saving time. Then the original procedure code which was written by Python is expected to assemble the meta-groups based on meta-structures to shape up all the structures which were converted from the group data and were drawn manually. The InChI key of each structure was obtained in Excel embedded Chemoffice 14. The duplicates were removed according to the InChI code. The molecular energy calculation in the mechanic force field was performed by Python controlling Microsoft Excel containing all the structures and Chem 3D, which was provided as Supplementary Data 1. The conformational search was performed on Maestro 11.9 of the Schrödinger software package [32]. DFT-GIAO calculation of ^{1}H and ^{13}C NMR data were performed on Gaussian 16 at PCM/mPW1PW91/6-31+G** level [33]. The total times consumed for both structure determinations were about 3-4 days. The original procedure code and DFT calculation were performed in Supercomputing Center in Pilot National Laboratory for Marine Science and Technology (Qingdao). Details of the software codes used are available from the corresponding author on reasonable request. Codes used to calculate and analyze data about Dooerafa are provided as Supplementary Data 2 (review only).

2. Mata-groups and meta-structures of aaptourinamine

2.1 Core structures according to experimental data of aaptourinamine

Four core structures were deduced from the experimental data of aaptourinamine including of ^{1}H and ^{13}C NMR, and especially ^{1}H-^{1}H COSY, HMQC, and HMBC spectral data. The core structure D is not reasonable because of the high deshielded chemical shift of the carbonyl group at more than δ_C180, much bigger than the practical 168.8.

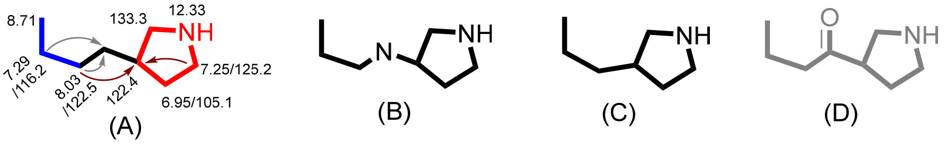

Figure S1. Core structures A-D for aaptourinamine

2.2 The connection site counting principle for aaptourinamine takes A-a as an example

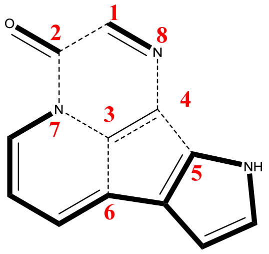

A-a

Connection points of A-a = $1_{C1} + 2_{C2} + 3_{C3} + 3_{C4} + 1_{C5} + 1_{C6} + 2_{C7} + 1_{C8} = 14$

Figure S2. The counting principle of the connection site

2.3 Meta-groups and meta-structures of aaptourinamine

The meta-groups include one carbonyl group, one imine group, and several olefinic carbons including of CH and C. And the meta-groups depend on each core structure. Thus, there are 14 meta-structures for the core structure A, 14 meta-structures for the core structure B, and 16 meta-structures for the core structure C. To decrease the connection points less than 14, some meta-structures were shaped up by connecting two additional bonds as shown in A-c, A-c1, A-e, and so on. Because the meta-structures are different from each other, each has different connection point serial numbers.

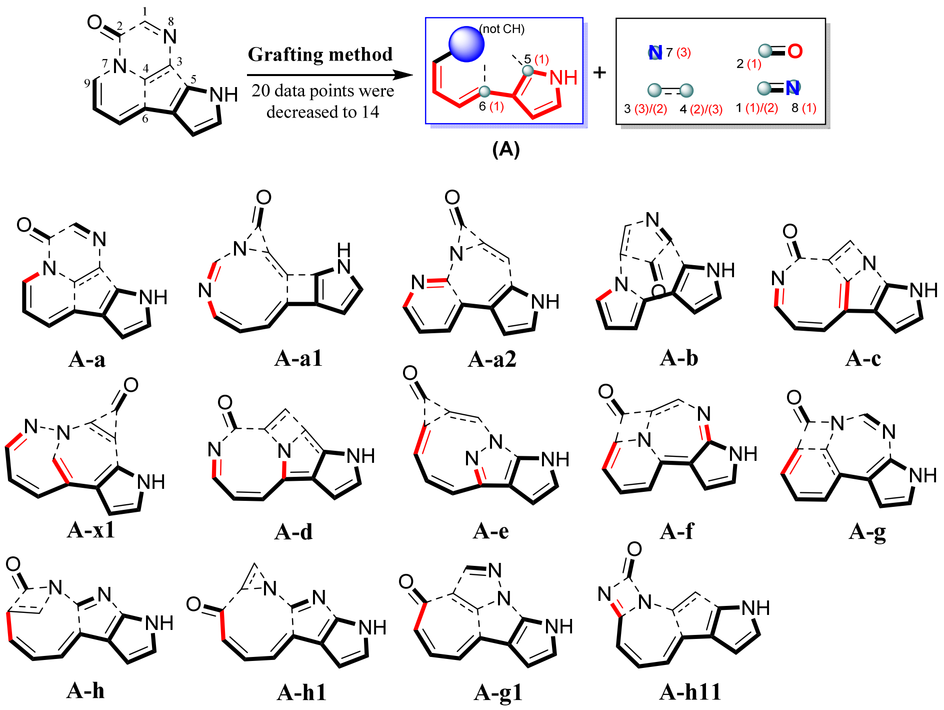

Figure S3. 14 meta-structures for the core structure A

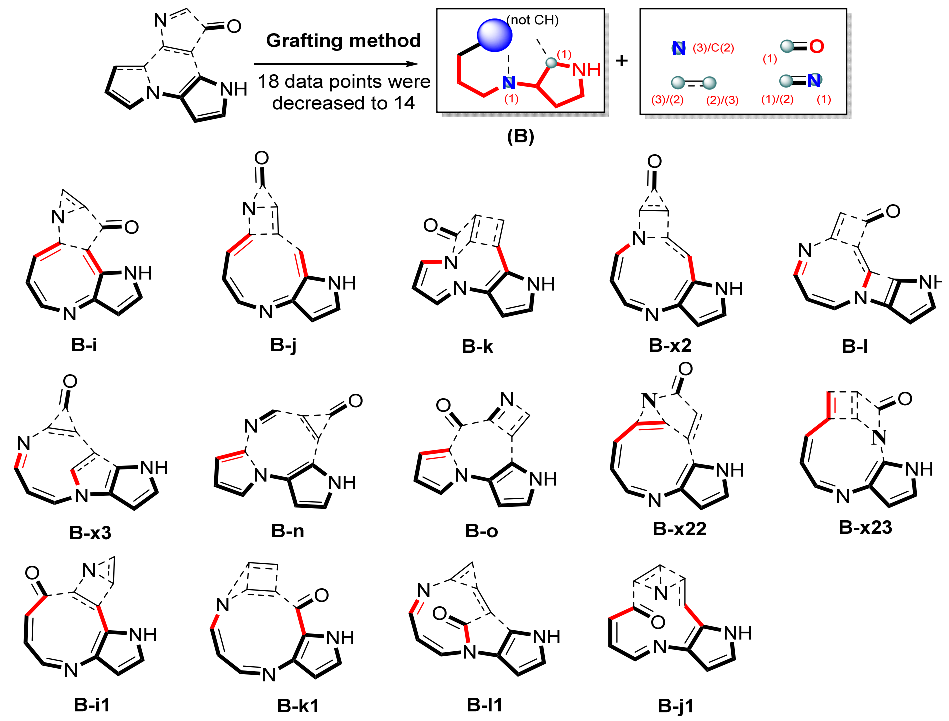

Figure S4. 14 meta-structures for the core structure B

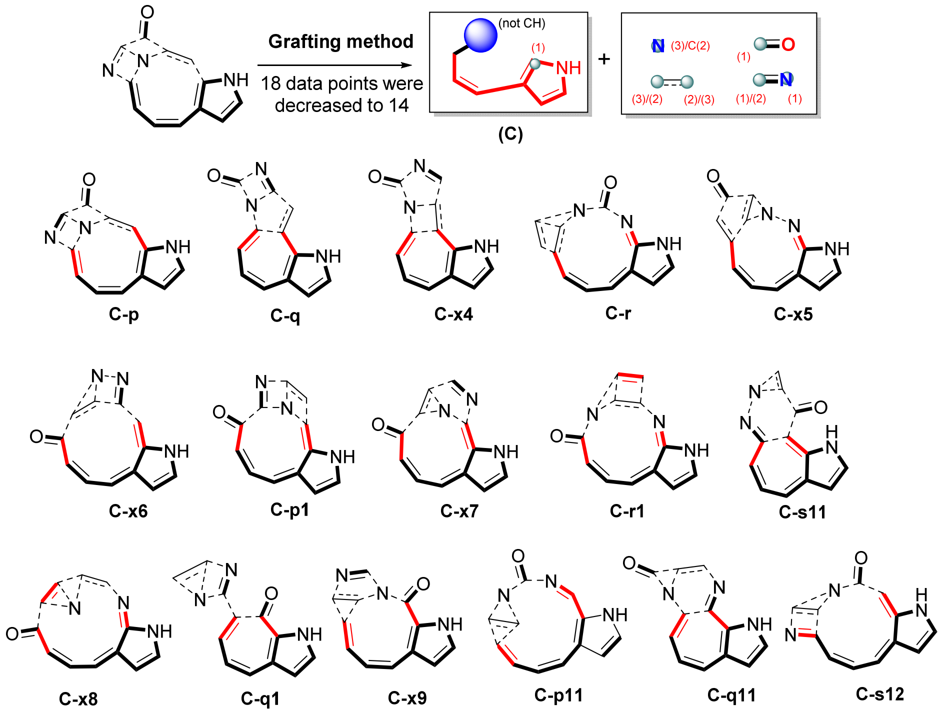

Figure S5. 16 meta-structures for the core structure C

3. The structural data groups for aaptourinamine

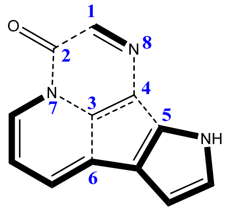

A-a

[[1, 2], [2, 3], [3, 4], [3, 5], [4, 6], [4, 7], [7, 8
[[1, 2], [2, 3], [3, 4], [3, 5], [4, 7], [4, 8], [6, 7
[[1, 2], [2, 3], [3, 4], [3, 6], [4, 5], [4, 7], [7, 8
[[1, 2], [2, 3], [3, 4], [3, 6], [4, 7], [4, 8], [5, 7
[[1, 2], [2, 3], [3, 4], [3, 7], [4, 5], [4, 6], [7, 8
[[1, 2], [2, 3], [3, 4], [3, 7], [4, 5], [4, 7], [6, 8
[[1, 2], [2, 3], [3, 4], [3, 7], [4, 5], [4, 8], [6, 7
[[1, 2], [2, 3], [3, 4], [3, 7], [4, 6], [4, 7], [5, 8
[[1, 2], [2, 3], [3, 4], [3, 7], [4, 6], [4, 8], [5, 7
[[1, 2], [2, 3], [3, 4], [3, 7], [4, 7], [4, 8], [5, 6
[[1, 2], [2, 3], [3, 4], [3, 8], [4, 5], [4, 7], [6, 7
[[1, 2], [2, 3], [3, 4], [3, 8], [4, 6], [4, 7], [5, 7
[[1, 2], [2, 4], [3, 4], [3, 5], [3, 6], [4, 7], [7, 8
[[1, 2], [2, 4], [3, 4], [3, 5], [3, 7], [4, 6], [7, 8
[[1, 2], [2, 4], [3, 4], [3, 5], [3, 7], [4, 7], [6, 8
[[1, 2], [2, 4], [3, 4], [3, 5], [3, 7], [4, 8], [6, 7
[[1, 2], [2, 4], [3, 4], [3, 5], [3, 8], [4, 7], [6, 7
[[1, 2], [2, 4], [3, 4], [3, 6], [3, 7], [4, 5], [7, 8
[[1, 2], [2, 4], [3, 4], [3, 6], [3, 7], [4, 7], [5, 8
[[1, 2], [2, 4], [3, 4], [3, 6], [3, 7], [4, 8], [5, 7
[[1, 2], [2, 4], [3, 4], [3, 6], [3, 8], [4, 7], [5, 7
[[1, 2], [2, 4], [3, 4], [3, 7], [3, 8], [4, 5], [6, 7
[[1, 2], [2, 4], [3, 4], [3, 7], [3, 8], [4, 6], [5, 7
[[1, 2], [2, 4], [3, 4], [3, 7], [3, 8], [4, 7], [5, 6
[[1, 2], [2, 5], [3, 4], [3, 6], [3, 7], [4, 7], [4, 8
[[1, 2], [2, 5], [3, 4], [3, 7], [3, 8], [4, 6], [4, 7
[[1, 2], [2, 6], [3, 4], [3, 5], [3, 7], [4, 7], [4, 8
[[1, 2], [2, 6], [3, 4], [3, 7], [3, 8], [4, 5], [4, 7
[[1, 2], [2, 7], [3, 4], [3, 5], [3, 6], [4, 7], [4, 8
[[1, 2], [2, 7], [3, 4], [3, 5], [3, 7], [4, 6], [4, 8
[[1, 2], [2, 7], [3, 4], [3, 5], [3, 8], [4, 6], [4, 7
[[1, 2], [2, 7], [3, 4], [3, 6], [3, 7], [4, 5], [4, 8
[[1, 2], [2, 7], [3, 4], [3, 6], [3, 8], [4, 5], [4, 7
[[1, 2], [2, 7], [3, 4], [3, 7], [3, 8], [4, 5], [4, 6
[[1, 2], [2, 8], [3, 4], [3, 5], [3, 7], [4, 6], [4, 7
[[1, 2], [2, 8], [3, 4], [3, 6], [3, 7], [4, 5], [4, 7
[[1, 3], [2, 3], [2, 4], [3, 4], [4, 5], [6, 7], [7, 8
[[1, 3], [2, 3], [2, 4], [3, 4], [4, 6], [5, 7], [7, 8
[[1, 3], [2, 3], [2, 4], [3, 4], [4, 7], [5, 6], [7, 8
[[1, 3], [2, 3], [2, 4], [3, 4], [4, 7], [5, 7], [6, 8
[[1, 3], [2, 3], [2, 4], [3, 4], [4, 7], [5, 8], [6, 7
[[1, 3], [2, 3], [2, 4], [3, 4], [4, 8], [5, 7], [6, 7
[[1, 3], [2, 3], [2, 5], [3, 4], [4, 6], [4, 7], [7, 8
[[1, 3], [2, 3], [2, 5], [3, 4], [4, 7], [4, 8], [6, 7
[[1, 3], [2, 3], [2, 6], [3, 4], [4, 5], [4, 7], [7, 8
[[1, 3], [2, 3], [2, 6], [3, 4], [4, 7], [4, 8], [5, 7
[[1, 3], [2, 3], [2, 7], [3, 4], [4, 5], [4, 6], [7, 8
[[1, 3], [2, 3], [2, 7], [3, 4], [4, 5], [4, 7], [6, 8
[[1, 3], [2, 3], [2, 7], [3, 4], [4, 5], [4, 8], [6, 7
[[1, 3], [2, 3], [2, 7], [3, 4], [4, 6], [4, 7], [5, 8
[[1, 3], [2, 3], [2, 7], [3, 4], [4, 6], [4, 8], [5, 7
[[1, 3], [2, 3], [2, 7], [3, 4], [4, 7], [4, 8], [5, 6
[[1, 3], [2, 3], [2, 8], [3, 4], [4, 5], [4, 7], [6, 7
[[1, 3], [2, 3], [2, 8], [3, 4], [4, 6], [4, 7], [5, 7
[[1, 3], [2, 4], [2, 5], [3, 4], [3, 6], [4, 7], [7, 8
[[1, 3], [2, 4], [2, 5], [3, 4], [3, 7], [4, 6], [7, 8
[[1, 3], [2, 4], [2, 5], [3, 4], [3, 7], [4, 7], [6, 8
[[1, 3], [2, 4], [2, 5], [3, 4], [3, 7], [4, 8], [6, 7
[[1, 3], [2, 4], [2, 5], [3, 4], [3, 8], [4, 7], [6, 7
[[1, 3], [2, 4], [2, 6], [3, 4], [3, 5], [4, 7], [7, 8
[[1, 3], [2, 4], [2, 6], [3, 4], [3, 7], [4, 5], [7, 8
[[1, 3], [2, 4], [2, 6], [3, 4], [3, 7], [4, 7], [5, 8
[[1, 3], [2, 4], [2, 6], [3, 4], [3, 7], [4, 8], [5, 7
[[1, 3], [2, 4], [2, 6], [3, 4], [3, 8], [4, 7], [5, 7
[[1, 3], [2, 4], [2, 7], [3, 4], [3, 5], [4, 6], [7, 8
[[1, 3], [2, 4], [2, 7], [3, 4], [3, 5], [4, 7], [6, 8
[[1, 3], [2, 4], [2, 7], [3, 4], [3, 5], [4, 8], [6, 7
[[1, 3], [2, 4], [2, 7], [3, 4], [3, 6], [4, 5], [7, 8
[[1, 3], [2, 4], [2, 7], [3, 4], [3, 6], [4, 7], [5, 8
[[1, 3], [2, 4], [2, 7], [3, 4], [3, 6], [4, 8], [5, 7
[[1, 3], [2, 4], [2, 7], [3, 4], [3, 7], [4, 5], [6, 8
[[1, 3], [2, 4], [2, 7], [3, 4], [3, 7], [4, 6], [5, 8
[[1, 3], [2, 4], [2, 7], [3, 4], [3, 7], [4, 8], [5, 6
[[1, 3], [2, 4], [2, 7], [3, 4], [3, 8], [4, 5], [6, 7
[[1, 3], [2, 4], [2, 7], [3, 4], [3, 8], [4, 6], [5, 7
[[1, 3], [2, 4], [2, 7], [3, 4], [3, 8], [4, 7], [5, 6
[[1, 3], [2, 4], [2, 8], [3, 4], [3, 5], [4, 7], [6, 7
[[1, 3], [2, 4], [2, 8], [3, 4], [3, 6], [4, 7], [5, 7
[[1, 3], [2, 4], [2, 8], [3, 4], [3, 7], [4, 5], [6, 7
[[1, 3], [2, 4], [2, 8], [3, 4], [3, 7], [4, 6], [5, 7
[[1, 3], [2, 4], [2, 8], [3, 4], [3, 7], [4, 7], [5, 6
[[1, 3], [2, 5], [2, 6], [3, 4], [3, 7], [4, 7], [4, 8
[[1, 3], [2, 5], [2, 7], [3, 4], [3, 6], [4, 7], [4, 8
[[1, 3], [2, 5], [2, 7], [3, 4], [3, 7], [4, 6], [4, 8
[[1, 3], [2, 5], [2, 7], [3, 4], [3, 8], [4, 6], [4, 7
[[1, 3], [2, 5], [2, 8], [3, 4], [3, 7], [4, 6], [4, 7
[[1, 3], [2, 6], [2, 7], [3, 4], [3, 5], [4, 7], [4, 8
[[1, 3], [2, 6], [2, 7], [3, 4], [3, 7], [4, 5], [4, 8
[[1, 3], [2, 6], [2, 7], [3, 4], [3, 8], [4, 5], [4, 7
[[1, 3], [2, 6], [2, 8], [3, 4], [3, 7], [4, 5], [4, 7
[[1, 3], [2, 7], [2, 8], [3, 4], [3, 5], [4, 6], [4, 7
[[1, 3], [2, 7], [2, 8], [3, 4], [3, 6], [4, 5], [4, 7
[[1, 3], [2, 7], [2, 8], [3, 4], [3, 7], [4, 5], [4, 6
[[1, 4], [2, 3], [2, 4], [3, 4], [3, 5], [6, 7], [7, 8
[[1, 4], [2, 3], [2, 4], [3, 4], [3, 6], [5, 7], [7, 8
[[1, 4], [2, 3], [2, 4], [3, 4], [3, 7], [5, 6], [7, 8
[[1, 4], [2, 3], [2, 4], [3, 4], [3, 7], [5, 7], [6, 8
[[1, 4], [2, 3], [2, 4], [3, 4], [3, 7], [5, 8], [6, 7
[[1, 4], [2, 3], [2, 4], [3, 4], [3, 8], [5, 7], [6, 7
[[1, 4], [2, 3], [2, 5], [3, 4], [3, 6], [4, 7], [7, 8
[[1, 4], [2, 3], [2, 5], [3, 4], [3, 7], [4, 6], [7, 8
[[1, 4], [2, 3], [2, 5], [3, 4], [3, 7], [4, 7], [6, 8
[[1, 4], [2, 3], [2, 5], [3, 4], [3, 7], [4, 8], [6, 7
[[1, 4], [2, 3], [2, 5], [3, 4], [3, 8], [4, 7], [6, 7
[[1, 4], [2, 3], [2, 6], [3, 4], [3, 5], [4, 7], [7, 8
[[1, 4], [2, 3], [2, 6], [3, 4], [3, 7], [4, 5], [7, 8
[[1, 4], [2, 3], [2, 6], [3, 4], [3, 7], [4, 7], [5, 8
[[1, 4], [2, 3], [2, 6], [3, 4], [3, 7], [4, 8], [5, 7
[[1, 4], [2, 3], [2, 6], [3, 4], [3, 8], [4, 7], [5, 7
[[1, 4], [2, 3], [2, 7], [3, 4], [3, 5], [4, 6], [7, 8
[[1, 4], [2, 3], [2, 7], [3, 4], [3, 5], [4, 7], [6, 8
[[1, 4], [2, 3], [2, 7], [3, 4], [3, 5], [4, 8], [6, 7
[[1, 4], [2, 3], [2, 7], [3, 4], [3, 6], [4, 5], [7, 8

[[1, 4], [2, 3], [2, 7], [3, 4], [3, 6], [4, 7], [5, 8
[[1, 4], [2, 3], [2, 7], [3, 4], [3, 6], [4, 8], [5, 7
[[1, 4], [2, 3], [2, 7], [3, 4], [3, 7], [4, 5], [6, 8
[[1, 4], [2, 3], [2, 7], [3, 4], [3, 7], [4, 6], [5, 8
[[1, 4], [2, 3], [2, 7], [3, 4], [3, 7], [4, 8], [5, 6
[[1, 4], [2, 3], [2, 7], [3, 4], [3, 8], [4, 5], [6, 7
[[1, 4], [2, 3], [2, 7], [3, 4], [3, 8], [4, 6], [5, 7
[[1, 4], [2, 3], [2, 7], [3, 4], [3, 8], [4, 7], [5, 6
[[1, 4], [2, 3], [2, 8], [3, 4], [3, 5], [4, 7], [6, 7
[[1, 4], [2, 3], [2, 8], [3, 4], [3, 6], [4, 7], [5, 7
[[1, 4], [2, 3], [2, 8], [3, 4], [3, 7], [4, 5], [6, 7
[[1, 4], [2, 3], [2, 8], [3, 4], [3, 7], [4, 6], [5, 7
[[1, 4], [2, 3], [2, 8], [3, 4], [3, 7], [4, 7], [5, 6
[[1, 4], [2, 4], [2, 5], [3, 4], [3, 6], [3, 7], [7, 8
[[1, 4], [2, 4], [2, 5], [3, 4], [3, 7], [3, 8], [6, 7
[[1, 4], [2, 4], [2, 6], [3, 4], [3, 5], [3, 7], [7, 8
[[1, 4], [2, 4], [2, 6], [3, 4], [3, 7], [3, 8], [5, 7
[[1, 4], [2, 4], [2, 7], [3, 4], [3, 5], [3, 6], [7, 8
[[1, 4], [2, 4], [2, 7], [3, 4], [3, 5], [3, 7], [6, 8
[[1, 4], [2, 4], [2, 7], [3, 4], [3, 5], [3, 8], [6, 7
[[1, 4], [2, 4], [2, 7], [3, 4], [3, 6], [3, 7], [5, 8
[[1, 4], [2, 4], [2, 7], [3, 4], [3, 6], [3, 8], [5, 7
[[1, 4], [2, 4], [2, 7], [3, 4], [3, 7], [3, 8], [5, 6
[[1, 4], [2, 4], [2, 8], [3, 4], [3, 5], [3, 7], [6, 7
[[1, 4], [2, 4], [2, 8], [3, 4], [3, 6], [3, 7], [5, 7
[[1, 4], [2, 5], [2, 6], [3, 4], [3, 7], [3, 8], [4, 7
[[1, 4], [2, 5], [2, 7], [3, 4], [3, 6], [3, 7], [4, 8
[[1, 4], [2, 5], [2, 7], [3, 4], [3, 6], [3, 8], [4, 7
[[1, 4], [2, 5], [2, 7], [3, 4], [3, 7], [3, 8], [4, 6
[[1, 4], [2, 5], [2, 8], [3, 4], [3, 6], [3, 7], [4, 7
[[1, 4], [2, 6], [2, 7], [3, 4], [3, 5], [3, 7], [4, 8
[[1, 4], [2, 6], [2, 7], [3, 4], [3, 5], [3, 8], [4, 7
[[1, 4], [2, 6], [2, 7], [3, 4], [3, 7], [3, 8], [4, 5
[[1, 4], [2, 6], [2, 8], [3, 4], [3, 5], [3, 7], [4, 7
[[1, 4], [2, 7], [2, 8], [3, 4], [3, 5], [3, 6], [4, 7
[[1, 4], [2, 7], [2, 8], [3, 4], [3, 5], [3, 7], [4, 6
[[1, 4], [2, 7], [2, 8], [3, 4], [3, 6], [3, 7], [4, 5
[[1, 5], [2, 3], [2, 4], [3, 4], [3, 6], [4, 7], [7, 8
[[1, 5], [2, 3], [2, 4], [3, 4], [3, 7], [4, 6], [7, 8
[[1, 5], [2, 3], [2, 4], [3, 4], [3, 7], [4, 7], [6, 8
[[1, 5], [2, 3], [2, 4], [3, 4], [3, 7], [4, 8], [6, 7
[[1, 5], [2, 3], [2, 4], [3, 4], [3, 8], [4, 7], [6, 7
[[1, 5], [2, 3], [2, 6], [3, 4], [3, 7], [4, 7], [4, 8
[[1, 5], [2, 3], [2, 7], [3, 4], [3, 6], [4, 7], [4, 8
[[1, 5], [2, 3], [2, 7], [3, 4], [3, 7], [4, 6], [4, 8
[[1, 5], [2, 3], [2, 7], [3, 4], [3, 8], [4, 6], [4, 7
[[1, 5], [2, 3], [2, 8], [3, 4], [3, 7], [4, 6], [4, 7
[[1, 5], [2, 4], [2, 6], [3, 4], [3, 7], [3, 8], [4, 7
[[1, 5], [2, 4], [2, 7], [3, 4], [3, 6], [3, 7], [4, 8
[[1, 5], [2, 4], [2, 7], [3, 4], [3, 6], [3, 8], [4, 7
[[1, 5], [2, 4], [2, 7], [3, 4], [3, 7], [3, 8], [4, 6
[[1, 5], [2, 4], [2, 8], [3, 4], [3, 6], [3, 7], [4, 7
[[1, 6], [2, 3], [2, 4], [3, 4], [3, 5], [4, 7], [7, 8
[[1, 6], [2, 3], [2, 4], [3, 4], [3, 7], [4, 5], [7, 8
[[1, 6], [2, 3], [2, 4], [3, 4], [3, 7], [4, 7], [5, 8
[[1, 6], [2, 3], [2, 4], [3, 4], [3, 7], [4, 8], [5, 7
[[1, 6], [2, 3], [2, 4], [3, 4], [3, 8], [4, 7], [5, 7
[[1, 6], [2, 3], [2, 5], [3, 4], [3, 7], [4, 7], [4, 8
[[1, 6], [2, 3], [2, 7], [3, 4], [3, 5], [4, 7], [4, 8
[[1, 6], [2, 3], [2, 7], [3, 4], [3, 7], [4, 5], [4, 8
[[1, 6], [2, 3], [2, 7], [3, 4], [3, 8], [4, 5], [4, 7
[[1, 6], [2, 3], [2, 8], [3, 4], [3, 7], [4, 5], [4, 7
[[1, 6], [2, 4], [2, 5], [3, 4], [3, 7], [3, 8], [4, 7
[[1, 6], [2, 4], [2, 7], [3, 4], [3, 5], [3, 7], [4, 8
[[1, 6], [2, 4], [2, 7], [3, 4], [3, 5], [3, 8], [4, 7
[[1, 6], [2, 4], [2, 7], [3, 4], [3, 7], [3, 8], [4, 5
[[1, 6], [2, 4], [2, 8], [3, 4], [3, 5], [3, 7], [4, 7
[[1, 7], [2, 3], [2, 4], [3, 4], [3, 5], [4, 6], [7, 8
[[1, 7], [2, 3], [2, 4], [3, 4], [3, 5], [4, 7], [6, 8
[[1, 7], [2, 3], [2, 4], [3, 4], [3, 5], [4, 8], [6, 7
[[1, 7], [2, 3], [2, 4], [3, 4], [3, 6], [4, 5], [7, 8
[[1, 7], [2, 3], [2, 4], [3, 4], [3, 6], [4, 7], [5, 8
[[1, 7], [2, 3], [2, 4], [3, 4], [3, 6], [4, 8], [5, 7
[[1, 7], [2, 3], [2, 4], [3, 4], [3, 7], [4, 5], [6, 8
[[1, 7], [2, 3], [2, 4], [3, 4], [3, 7], [4, 6], [5, 8
[[1, 7], [2, 3], [2, 4], [3, 4], [3, 7], [4, 8], [5, 6
[[1, 7], [2, 3], [2, 4], [3, 4], [3, 8], [4, 5], [6, 7
[[1, 7], [2, 3], [2, 4], [3, 4], [3, 8], [4, 6], [5, 7
[[1, 7], [2, 3], [2, 4], [3, 4], [3, 8], [4, 7], [5, 6
[[1, 7], [2, 3], [2, 5], [3, 4], [3, 6], [4, 7], [4, 8
[[1, 7], [2, 3], [2, 5], [3, 4], [3, 7], [4, 6], [4, 8
[[1, 7], [2, 3], [2, 5], [3, 4], [3, 8], [4, 6], [4, 7
[[1, 7], [2, 3], [2, 6], [3, 4], [3, 5], [4, 7], [4, 8
[[1, 7], [2, 3], [2, 6], [3, 4], [3, 7], [4, 5], [4, 8
[[1, 7], [2, 3], [2, 6], [3, 4], [3, 8], [4, 5], [4, 7
[[1, 7], [2, 3], [2, 7], [3, 4], [3, 5], [4, 6], [4, 8
[[1, 7], [2, 3], [2, 7], [3, 4], [3, 6], [4, 5], [4, 8
[[1, 7], [2, 3], [2, 7], [3, 4], [3, 8], [4, 5], [4, 6
[[1, 7], [2, 3], [2, 8], [3, 4], [3, 5], [4, 6], [4, 7
[[1, 7], [2, 3], [2, 8], [3, 4], [3, 6], [4, 5], [4, 7
[[1, 7], [2, 3], [2, 8], [3, 4], [3, 7], [4, 5], [4, 6
[[1, 7], [2, 4], [2, 5], [3, 4], [3, 6], [3, 7], [4, 8
[[1, 7], [2, 4], [2, 5], [3, 4], [3, 6], [3, 8], [4, 7
[[1, 7], [2, 4], [2, 5], [3, 4], [3, 7], [3, 8], [4, 6
[[1, 7], [2, 4], [2, 6], [3, 4], [3, 5], [3, 7], [4, 8
[[1, 7], [2, 4], [2, 6], [3, 4], [3, 5], [3, 8], [4, 7
[[1, 7], [2, 4], [2, 6], [3, 4], [3, 7], [3, 8], [4, 5
[[1, 7], [2, 4], [2, 7], [3, 4], [3, 5], [3, 6], [4, 8
[[1, 7], [2, 4], [2, 7], [3, 4], [3, 5], [3, 8], [4, 6
[[1, 7], [2, 4], [2, 7], [3, 4], [3, 6], [3, 8], [4, 5
[[1, 7], [2, 4], [2, 8], [3, 4], [3, 5], [3, 6], [4, 7
[[1, 7], [2, 4], [2, 8], [3, 4], [3, 5], [3, 7], [4, 6
[[1, 7], [2, 4], [2, 8], [3, 4], [3, 6], [3, 7], [4, 5

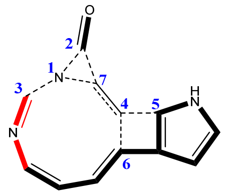

A-a1

[[1, 2], [1, 3], [1, 4], [2, 4], [4, 7], [5, 7], [6, 7
[[1, 2], [1, 3], [1, 4], [2, 7], [4, 5], [4, 7], [6, 7
[[1, 2], [1, 3], [1, 4], [2, 7], [4, 6], [4, 7], [5, 7
[[1, 2], [1, 3], [1, 7], [2, 4], [4, 5], [4, 7], [6, 7
[[1, 2], [1, 3], [1, 7], [2, 4], [4, 6], [4, 7], [5, 7
[[1, 2], [1, 3], [1, 7], [2, 7], [4, 5], [4, 6], [4, 7
[[1, 2], [1, 4], [1, 5], [2, 4], [3, 7], [4, 7], [6, 7
[[1, 2], [1, 4], [1, 5], [2, 7], [3, 4], [4, 7], [6, 7
[[1, 2], [1, 4], [1, 5], [2, 7], [3, 7], [4, 6], [4, 7
[[1, 2], [1, 4], [1, 6], [2, 4], [3, 7], [4, 7], [5, 7
[[1, 2], [1, 4], [1, 6], [2, 7], [3, 4], [4, 7], [5, 7
[[1, 2], [1, 4], [1, 6], [2, 7], [3, 7], [4, 5], [4, 7
[[1, 2], [1, 4], [1, 7], [2, 3], [4, 5], [4, 7], [6, 7
[[1, 2], [1, 4], [1, 7], [2, 3], [4, 6], [4, 7], [5, 7
[[1, 2], [1, 4], [1, 7], [2, 4], [3, 5], [4, 7], [6, 7
[[1, 2], [1, 4], [1, 7], [2, 4], [3, 6], [4, 7], [5, 7
[[1, 2], [1, 4], [1, 7], [2, 4], [3, 7], [4, 7], [5, 6
[[1, 2], [1, 4], [1, 7], [2, 5], [3, 4], [4, 7], [6, 7
[[1, 2], [1, 4], [1, 7], [2, 5], [3, 7], [4, 6], [4, 7
[[1, 2], [1, 4], [1, 7], [2, 6], [3, 4], [4, 7], [5, 7
[[1, 2], [1, 4], [1, 7], [2, 6], [3, 7], [4, 5], [4, 7
[[1, 2], [1, 4], [1, 7], [2, 7], [3, 4], [4, 7], [5, 6
[[1, 2], [1, 4], [1, 7], [2, 7], [3, 5], [4, 6], [4, 7
[[1, 2], [1, 4], [1, 7], [2, 7], [3, 6], [4, 5], [4, 7
[[1, 2], [1, 5], [1, 7], [2, 4], [3, 4], [4, 7], [6, 7
[[1, 2], [1, 5], [1, 7], [2, 4], [3, 7], [4, 6], [4, 7
[[1, 2], [1, 5], [1, 7], [2, 7], [3, 4], [4, 6], [4, 7
[[1, 2], [1, 6], [1, 7], [2, 4], [3, 4], [4, 7], [5, 7
[[1, 2], [1, 6], [1, 7], [2, 4], [3, 7], [4, 5], [4, 7
[[1, 2], [1, 6], [1, 7], [2, 7], [3, 4], [4, 5], [4, 7
[[1, 3], [1, 4], [1, 5], [2, 4], [2, 7], [4, 7], [6, 7
[[1, 3], [1, 4], [1, 6], [2, 4], [2, 7], [4, 7], [5, 7
[[1, 3], [1, 4], [1, 7], [2, 4], [2, 5], [4, 7], [6, 7
[[1, 3], [1, 4], [1, 7], [2, 4], [2, 6], [4, 7], [5, 7
[[1, 3], [1, 4], [1, 7], [2, 4], [2, 7], [4, 7], [5, 6
[[1, 3], [1, 4], [1, 7], [2, 5], [2, 7], [4, 6], [4, 7
[[1, 3], [1, 4], [1, 7], [2, 6], [2, 7], [4, 5], [4, 7
[[1, 3], [1, 5], [1, 7], [2, 4], [2, 7], [4, 6], [4, 7
[[1, 3], [1, 6], [1, 7], [2, 4], [2, 7], [4, 5], [4, 7
[[1, 4], [1, 5], [1, 6], [2, 4], [2, 7], [3, 7], [4, 7
[[1, 4], [1, 5], [1, 7], [2, 3], [2, 4], [4, 7], [6, 7
[[1, 4], [1, 5], [1, 7], [2, 3], [2, 7], [4, 6], [4, 7
[[1, 4], [1, 5], [1, 7], [2, 4], [2, 6], [3, 7], [4, 7
[[1, 4], [1, 5], [1, 7], [2, 4], [2, 7], [3, 6], [4, 7
[[1, 4], [1, 5], [1, 7], [2, 6], [2, 7], [3, 4], [4, 7
[[1, 4], [1, 6], [1, 7], [2, 3], [2, 4], [4, 7], [5, 7
[[1, 4], [1, 6], [1, 7], [2, 3], [2, 7], [4, 5], [4, 7
[[1, 4], [1, 6], [1, 7], [2, 4], [2, 5], [3, 7], [4, 7
[[1, 4], [1, 6], [1, 7], [2, 4], [2, 7], [3, 5], [4, 7
[[1, 4], [1, 6], [1, 7], [2, 5], [2, 7], [3, 4], [4, 7
[[1, 5], [1, 6], [1, 7], [2, 4], [2, 7], [3, 4], [4, 7]]]

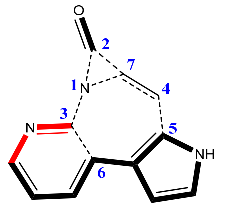

A-a2

[[1, 2], [1, 3], [1, 4], [2, 3], [4, 7], [5, 7], [6, 7
[[1, 2], [1, 3], [1, 4], [2, 5], [3, 7], [4, 7], [6, 7
[[1, 2], [1, 3], [1, 4], [2, 6], [3, 7], [4, 7], [5, 7
[[1, 2], [1, 3], [1, 4], [2, 7], [3, 5], [4, 7], [6, 7
[[1, 2], [1, 3], [1, 4], [2, 7], [3, 6], [4, 7], [5, 7
[[1, 2], [1, 3], [1, 4], [2, 7], [3, 7], [4, 7], [5, 6
[[1, 2], [1, 3], [1, 5], [2, 4], [3, 7], [4, 7], [6, 7
[[1, 2], [1, 3], [1, 5], [2, 7], [3, 4], [4, 7], [6, 7
[[1, 2], [1, 3], [1, 5], [2, 7], [3, 7], [4, 6], [4, 7
[[1, 2], [1, 3], [1, 6], [2, 4], [3, 7], [4, 7], [5, 7
[[1, 2], [1, 3], [1, 6], [2, 7], [3, 4], [4, 7], [5, 7
[[1, 2], [1, 3], [1, 6], [2, 7], [3, 7], [4, 5], [4, 7
[[1, 2], [1, 3], [1, 7], [2, 3], [4, 5], [4, 7], [6, 7
[[1, 2], [1, 3], [1, 7], [2, 3], [4, 6], [4, 7], [5, 7
[[1, 2], [1, 3], [1, 7], [2, 4], [3, 5], [4, 7], [6, 7
[[1, 2], [1, 3], [1, 7], [2, 4], [3, 6], [4, 7], [5, 7
[[1, 2], [1, 3], [1, 7], [2, 4], [3, 7], [4, 7], [5, 6
[[1, 2], [1, 3], [1, 7], [2, 5], [3, 4], [4, 7], [6, 7
[[1, 2], [1, 3], [1, 7], [2, 5], [3, 7], [4, 6], [4, 7
[[1, 2], [1, 3], [1, 7], [2, 6], [3, 4], [4, 7], [5, 7
[[1, 2], [1, 3], [1, 7], [2, 6], [3, 7], [4, 5], [4, 7
[[1, 2], [1, 3], [1, 7], [2, 7], [3, 4], [4, 7], [5, 6
[[1, 2], [1, 3], [1, 7], [2, 7], [3, 5], [4, 6], [4, 7
[[1, 2], [1, 3], [1, 7], [2, 7], [3, 6], [4, 5], [4, 7
[[1, 2], [1, 4], [1, 5], [2, 3], [3, 7], [4, 7], [6, 7
[[1, 2], [1, 4], [1, 5], [2, 7], [3, 6], [3, 7], [4, 7
[[1, 2], [1, 4], [1, 6], [2, 3], [3, 7], [4, 7], [5, 7
[[1, 2], [1, 4], [1, 6], [2, 7], [3, 5], [3, 7], [4, 7
[[1, 2], [1, 4], [1, 7], [2, 3], [3, 5], [4, 7], [6, 7
[[1, 2], [1, 4], [1, 7], [2, 3], [3, 6], [4, 7], [5, 7
[[1, 2], [1, 4], [1, 7], [2, 3], [3, 7], [4, 7], [5, 6
[[1, 2], [1, 4], [1, 7], [2, 5], [3, 6], [3, 7], [4, 7
[[1, 2], [1, 4], [1, 7], [2, 6], [3, 5], [3, 7], [4, 7
[[1, 2], [1, 5], [1, 6], [2, 7], [3, 4], [3, 7], [4, 7
[[1, 2], [1, 5], [1, 7], [2, 3], [3, 4], [4, 7], [6, 7
[[1, 2], [1, 5], [1, 7], [2, 3], [3, 7], [4, 6], [4, 7
[[1, 2], [1, 5], [1, 7], [2, 4], [3, 6], [3, 7], [4, 7
[[1, 2], [1, 5], [1, 7], [2, 6], [3, 4], [3, 7], [4, 7
[[1, 2], [1, 5], [1, 7], [2, 7], [3, 4], [3, 6], [4, 7
[[1, 2], [1, 6], [1, 7], [2, 3], [3, 4], [4, 7], [5, 7
[[1, 2], [1, 6], [1, 7], [2, 3], [3, 7], [4, 5], [4, 7
[[1, 2], [1, 6], [1, 7], [2, 4], [3, 5], [3, 7], [4, 7
[[1, 2], [1, 6], [1, 7], [2, 5], [3, 4], [3, 7], [4, 7
[[1, 2], [1, 6], [1, 7], [2, 7], [3, 4], [3, 5], [4, 7
[[1, 3], [1, 4], [1, 5], [2, 3], [2, 7], [4, 7], [6, 7
[[1, 3], [1, 4], [1, 5], [2, 6], [2, 7], [3, 7], [4, 7
[[1, 3], [1, 4], [1, 6], [2, 3], [2, 7], [4, 7], [5, 7
[[1, 3], [1, 4], [1, 6], [2, 5], [2, 7], [3, 7], [4, 7
[[1, 3], [1, 4], [1, 7], [2, 3], [2, 5], [4, 7], [6, 7
[[1, 3], [1, 4], [1, 7], [2, 3], [2, 6], [4, 7], [5, 7
[[1, 3], [1, 4], [1, 7], [2, 3], [2, 7], [4, 7], [5, 6
[[1, 3], [1, 4], [1, 7], [2, 5], [2, 6], [3, 7], [4, 7
[[1, 3], [1, 4], [1, 7], [2, 5], [2, 7], [3, 6], [4, 7
[[1, 3], [1, 4], [1, 7], [2, 6], [2, 7], [3, 5], [4, 7
[[1, 3], [1, 5], [1, 6], [2, 4], [2, 7], [3, 7], [4, 7
[[1, 3], [1, 5], [1, 7], [2, 3], [2, 4], [4, 7], [6, 7

[[1, 3], [1, 5], [1, 7], [2, 3], [2, 7], [4, 6], [4, 7
[[1, 3], [1, 5], [1, 7], [2, 4], [2, 6], [3, 7], [4, 7
[[1, 3], [1, 5], [1, 7], [2, 4], [2, 7], [3, 6], [4, 7
[[1, 3], [1, 5], [1, 7], [2, 6], [2, 7], [3, 4], [4, 7
[[1, 3], [1, 6], [1, 7], [2, 3], [2, 4], [4, 7], [5, 7
[[1, 3], [1, 6], [1, 7], [2, 3], [2, 7], [4, 5], [4, 7
[[1, 3], [1, 6], [1, 7], [2, 4], [2, 5], [3, 7], [4, 7
[[1, 3], [1, 6], [1, 7], [2, 4], [2, 7], [3, 5], [4, 7
[[1, 3], [1, 6], [1, 7], [2, 5], [2, 7], [3, 4], [4, 7
[[1, 4], [1, 5], [1, 6], [2, 3], [2, 7], [3, 7], [4, 7
[[1, 4], [1, 5], [1, 7], [2, 3], [2, 6], [3, 7], [4, 7
[[1, 4], [1, 5], [1, 7], [2, 3], [2, 7], [3, 6], [4, 7
[[1, 4], [1, 6], [1, 7], [2, 3], [2, 5], [3, 7], [4, 7
[[1, 4], [1, 6], [1, 7], [2, 3], [2, 7], [3, 5], [4, 7
[[1, 5], [1, 6], [1, 7], [2, 3], [2, 4], [3, 7], [4, 7
[[1, 5], [1, 6], [1, 7], [2, 3], [2, 7], [3, 4], [4, 7

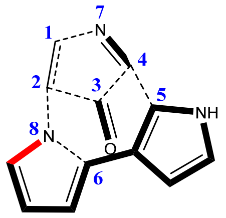

A-b

[[[1, 2], [1, 3], [2, 3], [2, 4], [4, 5], [6, 8], [7, 8
[[1, 2], [1, 3], [2, 3], [2, 6], [4, 5], [4, 8], [7, 8
[[1, 2], [1, 3], [2, 3], [2, 7], [4, 5], [4, 8], [6, 8
[[1, 2], [1, 3], [2, 3], [2, 8], [4, 5], [4, 6], [7, 8
[[1, 2], [1, 3], [2, 3], [2, 8], [4, 5], [4, 8], [6, 7
[[1, 2], [1, 3], [2, 4], [2, 6], [3, 8], [4, 5], [7, 8
[[1, 2], [1, 3], [2, 4], [2, 7], [3, 8], [4, 5], [6, 8
[[1, 2], [1, 3], [2, 4], [2, 8], [3, 6], [4, 5], [7, 8
[[1, 2], [1, 3], [2, 4], [2, 8], [3, 7], [4, 5], [6, 8
[[1, 2], [1, 3], [2, 4], [2, 8], [3, 8], [4, 5], [6, 7
[[1, 2], [1, 3], [2, 6], [2, 7], [3, 8], [4, 5], [4, 8
[[1, 2], [1, 3], [2, 6], [2, 8], [3, 4], [4, 5], [7, 8
[[1, 2], [1, 3], [2, 6], [2, 8], [3, 7], [4, 5], [4, 8
[[1, 2], [1, 3], [2, 7], [2, 8], [3, 4], [4, 5], [6, 8
[[1, 2], [1, 3], [2, 7], [2, 8], [3, 6], [4, 5], [4, 8
[[1, 2], [1, 3], [2, 7], [2, 8], [3, 8], [4, 5], [4, 6
[[1, 2], [1, 4], [2, 3], [2, 6], [3, 8], [4, 5], [7, 8
[[1, 2], [1, 4], [2, 3], [2, 7], [3, 8], [4, 5], [6, 8
[[1, 2], [1, 4], [2, 3], [2, 8], [3, 6], [4, 5], [7, 8
[[1, 2], [1, 4], [2, 3], [2, 8], [3, 7], [4, 5], [6, 8
[[1, 2], [1, 4], [2, 3], [2, 8], [3, 8], [4, 5], [6, 7
[[1, 2], [1, 4], [2, 6], [2, 8], [3, 7], [3, 8], [4, 5
[[1, 2], [1, 4], [2, 7], [2, 8], [3, 6], [3, 8], [4, 5
[[1, 2], [1, 6], [2, 3], [2, 4], [3, 8], [4, 5], [7, 8
[[1, 2], [1, 6], [2, 3], [2, 7], [3, 8], [4, 5], [4, 8
[[1, 2], [1, 6], [2, 3], [2, 8], [3, 4], [4, 5], [7, 8
[[1, 2], [1, 6], [2, 3], [2, 8], [3, 7], [4, 5], [4, 8
[[1, 2], [1, 6], [2, 4], [2, 8], [3, 7], [3, 8], [4, 5
[[1, 2], [1, 6], [2, 7], [2, 8], [3, 4], [3, 8], [4, 5
[[1, 2], [1, 7], [2, 3], [2, 4], [3, 8], [4, 5], [6, 8
[[1, 2], [1, 7], [2, 3], [2, 6], [3, 8], [4, 5], [4, 8
[[1, 2], [1, 7], [2, 3], [2, 8], [3, 4], [4, 5], [6, 8
[[1, 2], [1, 7], [2, 3], [2, 8], [3, 6], [4, 5], [4, 8
[[1, 2], [1, 7], [2, 3], [2, 8], [3, 8], [4, 5], [4, 6
[[1, 2], [1, 7], [2, 4], [2, 8], [3, 6], [3, 8], [4, 5
[[1, 2], [1, 7], [2, 6], [2, 8], [3, 4], [3, 8], [4, 5
[[1, 2], [1, 8], [2, 3], [2, 4], [3, 6], [4, 5], [7, 8
[[1, 2], [1, 8], [2, 3], [2, 4], [3, 7], [4, 5], [6, 8
[[1, 2], [1, 8], [2, 3], [2, 4], [3, 8], [4, 5], [6, 7
[[1, 2], [1, 8], [2, 3], [2, 6], [3, 4], [4, 5], [7, 8
[[1, 2], [1, 8], [2, 3], [2, 6], [3, 7], [4, 5], [4, 8
[[1, 2], [1, 8], [2, 3], [2, 7], [3, 4], [4, 5], [6, 8
[[1, 2], [1, 8], [2, 3], [2, 7], [3, 6], [4, 5], [4, 8
[[1, 2], [1, 8], [2, 3], [2, 7], [3, 8], [4, 5], [4, 6
[[1, 2], [1, 8], [2, 3], [2, 8], [3, 4], [4, 5], [6, 7
[[1, 2], [1, 8], [2, 3], [2, 8], [3, 7], [4, 5], [4, 6
[[1, 2], [1, 8], [2, 4], [2, 6], [3, 7], [3, 8], [4, 5
[[1, 2], [1, 8], [2, 4], [2, 7], [3, 6], [3, 8], [4, 5
[[1, 2], [1, 8], [2, 4], [2, 8], [3, 6], [3, 7], [4, 5
[[1, 2], [1, 8], [2, 6], [2, 7], [3, 4], [3, 8], [4, 5
[[1, 2], [1, 8], [2, 6], [2, 8], [3, 4], [3, 7], [4, 5
[[1, 2], [1, 8], [2, 7], [2, 8], [3, 4], [3, 6], [4, 5

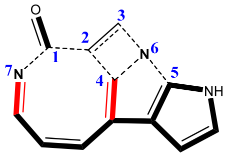

A-c

[[[1, 2], [1, 3], [2, 3], [2, 4], [4, 6], [5, 6], [6, 7
[[1, 2], [1, 3], [2, 3], [2, 6], [4, 5], [4, 6], [6, 7
[[1, 2], [1, 3], [2, 3], [2, 6], [4, 6], [4, 7], [5, 6
[[1, 2], [1, 4], [2, 3], [2, 4], [3, 6], [5, 6], [6, 7
[[1, 2], [1, 4], [2, 3], [2, 5], [3, 6], [4, 6], [6, 7
[[1, 2], [1, 4], [2, 3], [2, 6], [3, 4], [5, 6], [6, 7
[[1, 2], [1, 4], [2, 3], [2, 6], [3, 5], [4, 6], [6, 7
[[1, 2], [1, 4], [2, 3], [2, 6], [3, 6], [4, 5], [6, 7
[[1, 2], [1, 4], [2, 3], [2, 6], [3, 6], [4, 6], [5, 7
[[1, 2], [1, 4], [2, 3], [2, 6], [3, 6], [4, 7], [5, 6
[[1, 2], [1, 4], [2, 3], [2, 6], [3, 7], [4, 6], [5, 6
[[1, 2], [1, 4], [2, 3], [2, 7], [3, 6], [4, 6], [5, 6
[[1, 2], [1, 5], [2, 3], [2, 4], [3, 6], [4, 6], [6, 7
[[1, 2], [1, 5], [2, 3], [2, 6], [3, 4], [4, 6], [6, 7
[[1, 2], [1, 5], [2, 3], [2, 6], [3, 6], [4, 6], [4, 7
[[1, 2], [1, 6], [2, 3], [2, 4], [3, 4], [5, 6], [6, 7
[[1, 2], [1, 6], [2, 3], [2, 4], [3, 5], [4, 6], [6, 7
[[1, 2], [1, 6], [2, 3], [2, 4], [3, 6], [4, 5], [6, 7
[[1, 2], [1, 6], [2, 3], [2, 4], [3, 6], [4, 6], [5, 7
[[1, 2], [1, 6], [2, 3], [2, 4], [3, 6], [4, 7], [5, 6
[[1, 2], [1, 6], [2, 3], [2, 4], [3, 7], [4, 6], [5, 6
[[1, 2], [1, 6], [2, 3], [2, 5], [3, 4], [4, 6], [6, 7
[[1, 2], [1, 6], [2, 3], [2, 5], [3, 6], [4, 6], [4, 7
[[1, 2], [1, 6], [2, 3], [2, 6], [3, 4], [4, 5], [6, 7
[[1, 2], [1, 6], [2, 3], [2, 6], [3, 4], [4, 6], [5, 7
[[1, 2], [1, 6], [2, 3], [2, 6], [3, 4], [4, 7], [5, 6
[[1, 2], [1, 6], [2, 3], [2, 6], [3, 5], [4, 6], [4, 7
[[1, 2], [1, 6], [2, 3], [2, 6], [3, 6], [4, 5], [4, 7
[[1, 2], [1, 6], [2, 3], [2, 6], [3, 7], [4, 5], [4, 6
[[1, 2], [1, 6], [2, 3], [2, 7], [3, 4], [4, 6], [5, 6
[[1, 2], [1, 6], [2, 3], [2, 7], [3, 6], [4, 5], [4, 6
[[1, 2], [1, 7], [2, 3], [2, 4], [3, 6], [4, 6], [5, 6
[[1, 2], [1, 7], [2, 3], [2, 6], [3, 4], [4, 6], [5, 6
[[1, 2], [1, 7], [2, 3], [2, 6], [3, 6], [4, 5], [4, 6
[[1, 3], [1, 4], [2, 3], [2, 4], [2, 6], [5, 6], [6, 7
[[1, 3], [1, 4], [2, 3], [2, 5], [2, 6], [4, 6], [6, 7
[[1, 3], [1, 4], [2, 3], [2, 6], [2, 7], [4, 6], [5, 6
[[1, 3], [1, 5], [2, 3], [2, 4], [2, 6], [4, 6], [6, 7
[[1, 3], [1, 6], [2, 3], [2, 4], [2, 5], [4, 6], [6, 7
[[1, 3], [1, 6], [2, 3], [2, 4], [2, 6], [4, 5], [6, 7
[[1, 3], [1, 6], [2, 3], [2, 4], [2, 6], [4, 6], [5, 7
[[1, 3], [1, 6], [2, 3], [2, 4], [2, 6], [4, 7], [5, 6
[[1, 3], [1, 6], [2, 3], [2, 4], [2, 7], [4, 6], [5, 6
[[1, 3], [1, 6], [2, 3], [2, 5], [2, 6], [4, 6], [4, 7
[[1, 3], [1, 6], [2, 3], [2, 6], [2, 7], [4, 5], [4, 6
[[1, 3], [1, 7], [2, 3], [2, 4], [2, 6], [4, 6], [5, 6
[[1, 4], [1, 5], [2, 3], [2, 4], [2, 6], [3, 6], [6, 7
[[1, 4], [1, 5], [2, 3], [2, 6], [2, 7], [3, 6], [4, 6
[[1, 4], [1, 6], [2, 3], [2, 4], [2, 5], [3, 6], [6, 7
[[1, 4], [1, 6], [2, 3], [2, 4], [2, 6], [3, 5], [6, 7
[[1, 4], [1, 6], [2, 3], [2, 4], [2, 6], [3, 6], [5, 7
[[1, 4], [1, 6], [2, 3], [2, 4], [2, 6], [3, 7], [5, 6
[[1, 4], [1, 6], [2, 3], [2, 4], [2, 7], [3, 6], [5, 6
[[1, 4], [1, 6], [2, 3], [2, 5], [2, 6], [3, 4], [6, 7
[[1, 4], [1, 6], [2, 3], [2, 5], [2, 6], [3, 6], [4, 7
[[1, 4], [1, 6], [2, 3], [2, 5], [2, 6], [3, 7], [4, 6
[[1, 4], [1, 6], [2, 3], [2, 5], [2, 7], [3, 6], [4, 6
[[1, 4], [1, 6], [2, 3], [2, 6], [2, 7], [3, 4], [5, 6
[[1, 4], [1, 6], [2, 3], [2, 6], [2, 7], [3, 5], [4, 6
[[1, 4], [1, 6], [2, 3], [2, 6], [2, 7], [3, 6], [4, 5
[[1, 4], [1, 7], [2, 3], [2, 4], [2, 6], [3, 6], [5, 6
[[1, 4], [1, 7], [2, 3], [2, 5], [2, 6], [3, 6], [4, 6
[[1, 5], [1, 6], [2, 3], [2, 4], [2, 6], [3, 4], [6, 7
[[1, 5], [1, 6], [2, 3], [2, 4], [2, 6], [3, 6], [4, 7
[[1, 5], [1, 6], [2, 3], [2, 4], [2, 6], [3, 7], [4, 6

[[1, 5], [1, 6], [2, 3], [2, 4], [2, 7], [3, 6], [4, 6
[[1, 5], [1, 6], [2, 3], [2, 6], [2, 7], [3, 4], [4, 6
[[1, 5], [1, 7], [2, 3], [2, 4], [2, 6], [3, 6], [4, 6
[[1, 6], [1, 7], [2, 3], [2, 4], [2, 5], [3, 6], [4, 6
[[1, 6], [1, 7], [2, 3], [2, 4], [2, 6], [3, 4], [5, 6
[[1, 6], [1, 7], [2, 3], [2, 4], [2, 6], [3, 5], [4, 6
[[1, 6], [1, 7], [2, 3], [2, 4], [2, 6], [3, 6], [4, 5
[[1, 6], [1, 7], [2, 3], [2, 5], [2, 6], [3, 4], [4, 6

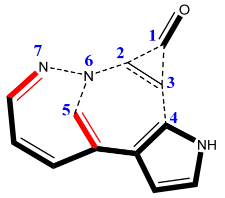

A-x1

[[1, 2], [1, 3], [2, 3], [2, 4], [3, 6], [5, 6], [6, 7
[[1, 2], [1, 3], [2, 3], [2, 5], [3, 6], [4, 6], [6, 7
[[1, 2], [1, 3], [2, 3], [2, 6], [3, 4], [5, 6], [6, 7
[[1, 2], [1, 3], [2, 3], [2, 6], [3, 5], [4, 6], [6, 7
[[1, 2], [1, 3], [2, 3], [2, 6], [3, 6], [4, 5], [6, 7
[[1, 2], [1, 3], [2, 3], [2, 6], [3, 6], [4, 6], [5, 7
[[1, 2], [1, 3], [2, 3], [2, 6], [3, 6], [4, 7], [5, 6
[[1, 2], [1, 3], [2, 3], [2, 6], [3, 7], [4, 6], [5, 6
[[1, 2], [1, 3], [2, 3], [2, 7], [3, 6], [4, 6], [5, 6
[[1, 2], [1, 4], [2, 3], [2, 6], [3, 5], [3, 6], [6, 7
[[1, 2], [1, 4], [2, 3], [2, 6], [3, 6], [3, 7], [5, 6
[[1, 2], [1, 5], [2, 3], [2, 6], [3, 4], [3, 6], [6, 7
[[1, 2], [1, 5], [2, 3], [2, 6], [3, 6], [3, 7], [4, 6
[[1, 2], [1, 6], [2, 3], [2, 4], [3, 5], [3, 6], [6, 7
[[1, 2], [1, 6], [2, 3], [2, 4], [3, 6], [3, 7], [5, 6
[[1, 2], [1, 6], [2, 3], [2, 5], [3, 4], [3, 6], [6, 7
[[1, 2], [1, 6], [2, 3], [2, 5], [3, 6], [3, 7], [4, 6
[[1, 2], [1, 6], [2, 3], [2, 6], [3, 4], [3, 5], [6, 7
[[1, 2], [1, 6], [2, 3], [2, 6], [3, 4], [3, 6], [5, 7
[[1, 2], [1, 6], [2, 3], [2, 6], [3, 4], [3, 7], [5, 6
[[1, 2], [1, 6], [2, 3], [2, 6], [3, 5], [3, 6], [4, 7
[[1, 2], [1, 6], [2, 3], [2, 6], [3, 5], [3, 7], [4, 6
[[1, 2], [1, 6], [2, 3], [2, 6], [3, 6], [3, 7], [4, 5
[[1, 2], [1, 6], [2, 3], [2, 7], [3, 4], [3, 6], [5, 6
[[1, 2], [1, 6], [2, 3], [2, 7], [3, 5], [3, 6], [4, 6
[[1, 2], [1, 7], [2, 3], [2, 6], [3, 4], [3, 6], [5, 6
[[1, 2], [1, 7], [2, 3], [2, 6], [3, 5], [3, 6], [4, 6
[[1, 3], [1, 4], [2, 3], [2, 5], [2, 6], [3, 6], [6, 7
[[1, 3], [1, 4], [2, 3], [2, 6], [2, 7], [3, 6], [5, 6
[[1, 3], [1, 5], [2, 3], [2, 4], [2, 6], [3, 6], [6, 7
[[1, 3], [1, 5], [2, 3], [2, 6], [2, 7], [3, 6], [4, 6
[[1, 3], [1, 6], [2, 3], [2, 4], [2, 5], [3, 6], [6, 7
[[1, 3], [1, 6], [2, 3], [2, 4], [2, 6], [3, 5], [6, 7
[[1, 3], [1, 6], [2, 3], [2, 4], [2, 6], [3, 6], [5, 7
[[1, 3], [1, 6], [2, 3], [2, 4], [2, 6], [3, 7], [5, 6
[[1, 3], [1, 6], [2, 3], [2, 4], [2, 7], [3, 6], [5, 6
[[1, 3], [1, 6], [2, 3], [2, 5], [2, 6], [3, 4], [6, 7
[[1, 3], [1, 6], [2, 3], [2, 5], [2, 6], [3, 6], [4, 7
[[1, 3], [1, 6], [2, 3], [2, 5], [2, 6], [3, 7], [4, 6
[[1, 3], [1, 6], [2, 3], [2, 5], [2, 7], [3, 6], [4, 6
[[1, 3], [1, 6], [2, 3], [2, 6], [2, 7], [3, 4], [5, 6
[[1, 3], [1, 6], [2, 3], [2, 6], [2, 7], [3, 5], [4, 6
[[1, 3], [1, 6], [2, 3], [2, 6], [2, 7], [3, 6], [4, 5
[[1, 3], [1, 7], [2, 3], [2, 4], [2, 6], [3, 6], [5, 6
[[1, 3], [1, 7], [2, 3], [2, 5], [2, 6], [3, 6], [4, 6
[[1, 4], [1, 6], [2, 3], [2, 5], [2, 6], [3, 6], [3, 7
[[1, 4], [1, 6], [2, 3], [2, 6], [2, 7], [3, 5], [3, 6
[[1, 5], [1, 6], [2, 3], [2, 4], [2, 6], [3, 6], [3, 7
[[1, 5], [1, 6], [2, 3], [2, 6], [2, 7], [3, 4], [3, 6
[[1, 6], [1, 7], [2, 3], [2, 4], [2, 6], [3, 5], [3, 6
[[1, 6], [1, 7], [2, 3], [2, 5], [2, 6], [3, 4], [3, 6]]]

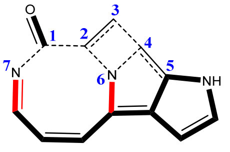

A-d

[[[1, 2], [1, 3], [2, 3], [2, 4], [4, 5], [4, 6], [6, 7
[[1, 2], [1, 3], [2, 3], [2, 6], [4, 5], [4, 6], [4, 7
[[1, 2], [1, 4], [2, 3], [2, 4], [3, 6], [4, 5], [6, 7
[[1, 2], [1, 4], [2, 3], [2, 6], [3, 4], [4, 5], [6, 7
[[1, 2], [1, 4], [2, 3], [2, 6], [3, 6], [4, 5], [4, 7
[[1, 2], [1, 4], [2, 3], [2, 6], [3, 7], [4, 5], [4, 6
[[1, 2], [1, 4], [2, 3], [2, 7], [3, 6], [4, 5], [4, 6
[[1, 2], [1, 6], [2, 3], [2, 4], [3, 4], [4, 5], [6, 7
[[1, 2], [1, 6], [2, 3], [2, 4], [3, 6], [4, 5], [4, 7
[[1, 2], [1, 6], [2, 3], [2, 4], [3, 7], [4, 5], [4, 6
[[1, 2], [1, 6], [2, 3], [2, 6], [3, 4], [4, 5], [4, 7
[[1, 2], [1, 6], [2, 3], [2, 7], [3, 4], [4, 5], [4, 6
[[1, 2], [1, 7], [2, 3], [2, 4], [3, 6], [4, 5], [4, 6
[[1, 2], [1, 7], [2, 3], [2, 6], [3, 4], [4, 5], [4, 6
[[1, 3], [1, 4], [2, 3], [2, 4], [2, 6], [4, 5], [6, 7
[[1, 3], [1, 4], [2, 3], [2, 6], [2, 7], [4, 5], [4, 6
[[1, 3], [1, 6], [2, 3], [2, 4], [2, 6], [4, 5], [4, 7
[[1, 3], [1, 6], [2, 3], [2, 4], [2, 7], [4, 5], [4, 6
[[1, 3], [1, 7], [2, 3], [2, 4], [2, 6], [4, 5], [4, 6
[[1, 4], [1, 6], [2, 3], [2, 4], [2, 6], [3, 7], [4, 5
[[1, 4], [1, 6], [2, 3], [2, 4], [2, 7], [3, 6], [4, 5
[[1, 4], [1, 6], [2, 3], [2, 6], [2, 7], [3, 4], [4, 5
[[1, 4], [1, 7], [2, 3], [2, 4], [2, 6], [3, 6], [4, 5
[[1, 6], [1, 7], [2, 3], [2, 4], [2, 6], [3, 4], [4, 5]]]

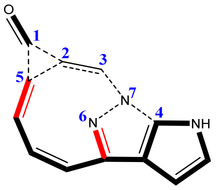

A-e

[[[1, 2], [1, 3], [2, 3], [2, 5], [4, 7], [5, 7], [6, 7
[[1, 2], [1, 3], [2, 3], [2, 7], [4, 5], [5, 7], [6, 7
[[1, 2], [1, 3], [2, 3], [2, 7], [4, 7], [5, 6], [5, 7
[[1, 2], [1, 4], [2, 3], [2, 5], [3, 7], [5, 7], [6, 7
[[1, 2], [1, 4], [2, 3], [2, 7], [3, 5], [5, 7], [6, 7
[[1, 2], [1, 4], [2, 3], [2, 7], [3, 7], [5, 6], [5, 7
[[1, 2], [1, 5], [2, 3], [2, 4], [3, 7], [5, 7], [6, 7
[[1, 2], [1, 5], [2, 3], [2, 5], [3, 7], [4, 7], [6, 7
[[1, 2], [1, 5], [2, 3], [2, 6], [3, 7], [4, 7], [5, 7
[[1, 2], [1, 5], [2, 3], [2, 7], [3, 4], [5, 7], [6, 7
[[1, 2], [1, 5], [2, 3], [2, 7], [3, 5], [4, 7], [6, 7
[[1, 2], [1, 5], [2, 3], [2, 7], [3, 6], [4, 7], [5, 7
[[1, 2], [1, 5], [2, 3], [2, 7], [3, 7], [4, 5], [6, 7
[[1, 2], [1, 5], [2, 3], [2, 7], [3, 7], [4, 6], [5, 7
[[1, 2], [1, 5], [2, 3], [2, 7], [3, 7], [4, 7], [5, 6
[[1, 2], [1, 6], [2, 3], [2, 5], [3, 7], [4, 7], [5, 7
[[1, 2], [1, 6], [2, 3], [2, 7], [3, 5], [4, 7], [5, 7
[[1, 2], [1, 6], [2, 3], [2, 7], [3, 7], [4, 5], [5, 7
[[1, 2], [1, 7], [2, 3], [2, 4], [3, 5], [5, 7], [6, 7
[[1, 2], [1, 7], [2, 3], [2, 4], [3, 7], [5, 6], [5, 7
[[1, 2], [1, 7], [2, 3], [2, 5], [3, 4], [5, 7], [6, 7
[[1, 2], [1, 7], [2, 3], [2, 5], [3, 5], [4, 7], [6, 7
[[1, 2], [1, 7], [2, 3], [2, 5], [3, 6], [4, 7], [5, 7
[[1, 2], [1, 7], [2, 3], [2, 5], [3, 7], [4, 5], [6, 7
[[1, 2], [1, 7], [2, 3], [2, 5], [3, 7], [4, 6], [5, 7
[[1, 2], [1, 7], [2, 3], [2, 5], [3, 7], [4, 7], [5, 6
[[1, 2], [1, 7], [2, 3], [2, 6], [3, 5], [4, 7], [5, 7
[[1, 2], [1, 7], [2, 3], [2, 6], [3, 7], [4, 5], [5, 7
[[1, 2], [1, 7], [2, 3], [2, 7], [3, 4], [5, 6], [5, 7
[[1, 2], [1, 7], [2, 3], [2, 7], [3, 5], [4, 5], [6, 7
[[1, 2], [1, 7], [2, 3], [2, 7], [3, 5], [4, 6], [5, 7
[[1, 2], [1, 7], [2, 3], [2, 7], [3, 5], [4, 7], [5, 6
[[1, 2], [1, 7], [2, 3], [2, 7], [3, 6], [4, 5], [5, 7
[[1, 2], [1, 7], [2, 3], [2, 7], [3, 7], [4, 5], [5, 6
[[1, 3], [1, 4], [2, 3], [2, 5], [2, 7], [5, 7], [6, 7
[[1, 3], [1, 5], [2, 3], [2, 4], [2, 7], [5, 7], [6, 7
[[1, 3], [1, 5], [2, 3], [2, 5], [2, 7], [4, 7], [6, 7
[[1, 3], [1, 5], [2, 3], [2, 6], [2, 7], [4, 7], [5, 7
[[1, 3], [1, 6], [2, 3], [2, 5], [2, 7], [4, 7], [5, 7
[[1, 3], [1, 7], [2, 3], [2, 4], [2, 5], [5, 7], [6, 7
[[1, 3], [1, 7], [2, 3], [2, 4], [2, 7], [5, 6], [5, 7
[[1, 3], [1, 7], [2, 3], [2, 5], [2, 6], [4, 7], [5, 7
[[1, 3], [1, 7], [2, 3], [2, 5], [2, 7], [4, 5], [6, 7
[[1, 3], [1, 7], [2, 3], [2, 5], [2, 7], [4, 6], [5, 7
[[1, 3], [1, 7], [2, 3], [2, 5], [2, 7], [4, 7], [5, 6
[[1, 3], [1, 7], [2, 3], [2, 6], [2, 7], [4, 5], [5, 7
[[1, 4], [1, 5], [2, 3], [2, 5], [2, 7], [3, 7], [6, 7
[[1, 4], [1, 5], [2, 3], [2, 6], [2, 7], [3, 7], [5, 7
[[1, 4], [1, 6], [2, 3], [2, 5], [2, 7], [3, 7], [5, 7
[[1, 4], [1, 7], [2, 3], [2, 5], [2, 6], [3, 7], [5, 7
[[1, 4], [1, 7], [2, 3], [2, 5], [2, 7], [3, 5], [6, 7
[[1, 4], [1, 7], [2, 3], [2, 5], [2, 7], [3, 6], [5, 7
[[1, 4], [1, 7], [2, 3], [2, 5], [2, 7], [3, 7], [5, 6
[[1, 4], [1, 7], [2, 3], [2, 6], [2, 7], [3, 5], [5, 7
[[1, 5], [1, 6], [2, 3], [2, 4], [2, 7], [3, 7], [5, 7
[[1, 5], [1, 6], [2, 3], [2, 5], [2, 7], [3, 7], [4, 7
[[1, 5], [1, 7], [2, 3], [2, 4], [2, 5], [3, 7], [6, 7
[[1, 5], [1, 7], [2, 3], [2, 4], [2, 6], [3, 7], [5, 7
[[1, 5], [1, 7], [2, 3], [2, 4], [2, 7], [3, 5], [6, 7
[[1, 5], [1, 7], [2, 3], [2, 4], [2, 7], [3, 6], [5, 7
[[1, 5], [1, 7], [2, 3], [2, 4], [2, 7], [3, 7], [5, 6
[[1, 5], [1, 7], [2, 3], [2, 5], [2, 6], [3, 7], [4, 7
[[1, 5], [1, 7], [2, 3], [2, 5], [2, 7], [3, 4], [6, 7
[[1, 5], [1, 7], [2, 3], [2, 5], [2, 7], [3, 6], [4, 7
[[1, 5], [1, 7], [2, 3], [2, 5], [2, 7], [3, 7], [4, 6
[[1, 5], [1, 7], [2, 3], [2, 6], [2, 7], [3, 4], [5, 7
[[1, 5], [1, 7], [2, 3], [2, 6], [2, 7], [3, 5], [4, 7
[[1, 5], [1, 7], [2, 3], [2, 6], [2, 7], [3, 7], [4, 5
[[1, 6], [1, 7], [2, 3], [2, 4], [2, 5], [3, 7], [5, 7
[[1, 6], [1, 7], [2, 3], [2, 4], [2, 7], [3, 5], [5, 7
[[1, 6], [1, 7], [2, 3], [2, 5], [2, 7], [3, 4], [5, 7
[[1, 6], [1, 7], [2, 3], [2, 5], [2, 7], [3, 5], [4, 7
[[1, 6], [1, 7], [2, 3], [2, 5], [2, 7], [3, 7], [4, 5

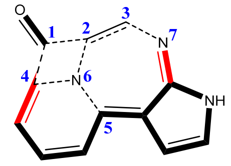

A-f

[[[1, 2], [1, 3], [2, 3], [2, 4], [4, 6], [5, 6], [6, 7
[[1, 2], [1, 3], [2, 3], [2, 6], [4, 5], [4, 6], [6, 7
[[1, 2], [1, 3], [2, 3], [2, 6], [4, 6], [4, 7], [5, 6
[[1, 2], [1, 4], [2, 3], [2, 4], [3, 6], [5, 6], [6, 7
[[1, 2], [1, 4], [2, 3], [2, 5], [3, 6], [4, 6], [6, 7
[[1, 2], [1, 4], [2, 3], [2, 6], [3, 4], [5, 6], [6, 7
[[1, 2], [1, 4], [2, 3], [2, 6], [3, 5], [4, 6], [6, 7
[[1, 2], [1, 4], [2, 3], [2, 6], [3, 6], [4, 5], [6, 7
[[1, 2], [1, 4], [2, 3], [2, 6], [3, 6], [4, 6], [5, 7
[[1, 2], [1, 4], [2, 3], [2, 6], [3, 6], [4, 7], [5, 6
[[1, 2], [1, 4], [2, 3], [2, 6], [3, 7], [4, 6], [5, 6
[[1, 2], [1, 4], [2, 3], [2, 7], [3, 6], [4, 6], [5, 6
[[1, 2], [1, 5], [2, 3], [2, 4], [3, 6], [4, 6], [6, 7
[[1, 2], [1, 5], [2, 3], [2, 6], [3, 4], [4, 6], [6, 7
[[1, 2], [1, 5], [2, 3], [2, 6], [3, 6], [4, 6], [4, 7
[[1, 2], [1, 6], [2, 3], [2, 4], [3, 4], [5, 6], [6, 7
[[1, 2], [1, 6], [2, 3], [2, 4], [3, 5], [4, 6], [6, 7
[[1, 2], [1, 6], [2, 3], [2, 4], [3, 6], [4, 5], [6, 7
[[1, 2], [1, 6], [2, 3], [2, 4], [3, 6], [4, 6], [5, 7
[[1, 2], [1, 6], [2, 3], [2, 4], [3, 6], [4, 7], [5, 6
[[1, 2], [1, 6], [2, 3], [2, 4], [3, 7], [4, 6], [5, 6
[[1, 2], [1, 6], [2, 3], [2, 5], [3, 4], [4, 6], [6, 7
[[1, 2], [1, 6], [2, 3], [2, 5], [3, 6], [4, 6], [4, 7
[[1, 2], [1, 6], [2, 3], [2, 6], [3, 4], [4, 5], [6, 7
[[1, 2], [1, 6], [2, 3], [2, 6], [3, 4], [4, 6], [5, 7
[[1, 2], [1, 6], [2, 3], [2, 6], [3, 4], [4, 7], [5, 6
[[1, 2], [1, 6], [2, 3], [2, 6], [3, 5], [4, 6], [4, 7
[[1, 2], [1, 6], [2, 3], [2, 6], [3, 6], [4, 5], [4, 7
[[1, 2], [1, 6], [2, 3], [2, 6], [3, 7], [4, 5], [4, 6
[[1, 2], [1, 6], [2, 3], [2, 7], [3, 4], [4, 6], [5, 6
[[1, 2], [1, 6], [2, 3], [2, 7], [3, 6], [4, 5], [4, 6
[[1, 2], [1, 7], [2, 3], [2, 4], [3, 6], [4, 6], [5, 6
[[1, 2], [1, 7], [2, 3], [2, 6], [3, 4], [4, 6], [5, 6
[[1, 2], [1, 7], [2, 3], [2, 6], [3, 6], [4, 5], [4, 6
[[1, 3], [1, 4], [2, 3], [2, 4], [2, 6], [5, 6], [6, 7
[[1, 3], [1, 4], [2, 3], [2, 5], [2, 6], [4, 6], [6, 7
[[1, 3], [1, 4], [2, 3], [2, 6], [2, 7], [4, 6], [5, 6
[[1, 3], [1, 5], [2, 3], [2, 4], [2, 6], [4, 6], [6, 7
[[1, 3], [1, 6], [2, 3], [2, 4], [2, 5], [4, 6], [6, 7
[[1, 3], [1, 6], [2, 3], [2, 4], [2, 6], [4, 5], [6, 7
[[1, 3], [1, 6], [2, 3], [2, 4], [2, 6], [4, 6], [5, 7
[[1, 3], [1, 6], [2, 3], [2, 4], [2, 6], [4, 7], [5, 6
[[1, 3], [1, 6], [2, 3], [2, 4], [2, 7], [4, 6], [5, 6
[[1, 3], [1, 6], [2, 3], [2, 5], [2, 6], [4, 6], [4, 7
[[1, 3], [1, 6], [2, 3], [2, 6], [2, 7], [4, 5], [4, 6
[[1, 3], [1, 7], [2, 3], [2, 4], [2, 6], [4, 6], [5, 6
[[1, 4], [1, 5], [2, 3], [2, 4], [2, 6], [3, 6], [6, 7
[[1, 4], [1, 5], [2, 3], [2, 6], [2, 7], [3, 6], [4, 6
[[1, 4], [1, 6], [2, 3], [2, 4], [2, 5], [3, 6], [6, 7
[[1, 4], [1, 6], [2, 3], [2, 4], [2, 6], [3, 5], [6, 7
[[1, 4], [1, 6], [2, 3], [2, 4], [2, 6], [3, 6], [5, 7
[[1, 4], [1, 6], [2, 3], [2, 4], [2, 6], [3, 7], [5, 6
[[1, 4], [1, 6], [2, 3], [2, 4], [2, 7], [3, 6], [5, 6
[[1, 4], [1, 6], [2, 3], [2, 5], [2, 6], [3, 4], [6, 7
[[1, 4], [1, 6], [2, 3], [2, 5], [2, 6], [3, 6], [4, 7
[[1, 4], [1, 6], [2, 3], [2, 5], [2, 6], [3, 7], [4, 6
[[1, 4], [1, 6], [2, 3], [2, 5], [2, 7], [3, 6], [4, 6
[[1, 4], [1, 6], [2, 3], [2, 6], [2, 7], [3, 4], [5, 6
[[1, 4], [1, 6], [2, 3], [2, 6], [2, 7], [3, 5], [4, 6
[[1, 4], [1, 6], [2, 3], [2, 6], [2, 7], [3, 6], [4, 5
[[1, 4], [1, 7], [2, 3], [2, 4], [2, 6], [3, 6], [5, 6
[[1, 4], [1, 7], [2, 3], [2, 5], [2, 6], [3, 6], [4, 6
[[1, 5], [1, 6], [2, 3], [2, 4], [2, 6], [3, 4], [6, 7
[[1, 5], [1, 6], [2, 3], [2, 4], [2, 6], [3, 6], [4, 7
[[1, 5], [1, 6], [2, 3], [2, 4], [2, 6], [3, 7], [4, 6
[[1, 5], [1, 6], [2, 3], [2, 4], [2, 7], [3, 6], [4, 6
[[1, 5], [1, 6], [2, 3], [2, 6], [2, 7], [3, 4], [4, 6
[[1, 5], [1, 7], [2, 3], [2, 4], [2, 6], [3, 6], [4, 6
[[1, 6], [1, 7], [2, 3], [2, 4], [2, 5], [3, 6], [4, 6
[[1, 6], [1, 7], [2, 3], [2, 4], [2, 6], [3, 4], [5, 6
[[1, 6], [1, 7], [2, 3], [2, 4], [2, 6], [3, 5], [4, 6
[[1, 6], [1, 7], [2, 3], [2, 4], [2, 6], [3, 6], [4, 5
[[1, 6], [1, 7], [2, 3], [2, 5], [2, 6], [3, 4], [4, 6

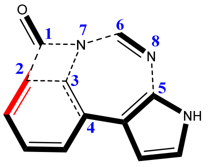

A-g

[[[1, 2], [1, 3], [2, 3], [3, 4], [5, 7], [6, 7], [7, 8
[[1, 2], [1, 3], [2, 5], [3, 4], [3, 7], [6, 7], [7, 8
[[1, 2], [1, 3], [2, 6], [3, 4], [3, 7], [5, 7], [7, 8
[[1, 2], [1, 3], [2, 7], [3, 4], [3, 5], [6, 7], [7, 8
[[1, 2], [1, 3], [2, 7], [3, 4], [3, 6], [5, 7], [7, 8
[[1, 2], [1, 3], [2, 7], [3, 4], [3, 7], [5, 6], [7, 8
[[1, 2], [1, 3], [2, 7], [3, 4], [3, 7], [5, 8], [6, 7
[[1, 2], [1, 3], [2, 7], [3, 4], [3, 8], [5, 7], [6, 7
[[1, 2], [1, 3], [2, 8], [3, 4], [3, 7], [5, 7], [6, 7
[[1, 2], [1, 5], [2, 3], [3, 4], [3, 7], [6, 7], [7, 8
[[1, 2], [1, 5], [2, 7], [3, 4], [3, 6], [3, 7], [7, 8
[[1, 2], [1, 5], [2, 7], [3, 4], [3, 7], [3, 8], [6, 7
[[1, 2], [1, 6], [2, 3], [3, 4], [3, 7], [5, 7], [7, 8
[[1, 2], [1, 6], [2, 7], [3, 4], [3, 5], [3, 7], [7, 8
[[1, 2], [1, 6], [2, 7], [3, 4], [3, 7], [3, 8], [5, 7
[[1, 2], [1, 7], [2, 3], [3, 4], [3, 5], [6, 7], [7, 8
[[1, 2], [1, 7], [2, 3], [3, 4], [3, 6], [5, 7], [7, 8
[[1, 2], [1, 7], [2, 3], [3, 4], [3, 7], [5, 6], [7, 8
[[1, 2], [1, 7], [2, 3], [3, 4], [3, 7], [5, 8], [6, 7
[[1, 2], [1, 7], [2, 3], [3, 4], [3, 8], [5, 7], [6, 7

[[1, 2], [1, 7], [2, 5], [3, 4], [3, 6], [3, 7], [7, 8
[[1, 2], [1, 7], [2, 5], [3, 4], [3, 7], [3, 8], [6, 7
[[1, 2], [1, 7], [2, 6], [3, 4], [3, 5], [3, 7], [7, 8
[[1, 2], [1, 7], [2, 6], [3, 4], [3, 7], [3, 8], [5, 7
[[1, 2], [1, 7], [2, 7], [3, 4], [3, 5], [3, 6], [7, 8
[[1, 2], [1, 7], [2, 7], [3, 4], [3, 5], [3, 8], [6, 7
[[1, 2], [1, 7], [2, 7], [3, 4], [3, 6], [3, 7], [5, 8
[[1, 2], [1, 7], [2, 7], [3, 4], [3, 6], [3, 8], [5, 7
[[1, 2], [1, 7], [2, 7], [3, 4], [3, 7], [3, 8], [5, 6
[[1, 2], [1, 7], [2, 8], [3, 4], [3, 5], [3, 7], [6, 7
[[1, 2], [1, 7], [2, 8], [3, 4], [3, 6], [3, 7], [5, 7
[[1, 2], [1, 8], [2, 3], [3, 4], [3, 7], [5, 7], [6, 7
[[1, 2], [1, 8], [2, 7], [3, 4], [3, 5], [3, 7], [6, 7
[[1, 2], [1, 8], [2, 7], [3, 4], [3, 6], [3, 7], [5, 7
[[1, 3], [1, 5], [2, 3], [2, 7], [3, 4], [6, 7], [7, 8
[[1, 3], [1, 5], [2, 6], [2, 7], [3, 4], [3, 7], [7, 8
[[1, 3], [1, 5], [2, 7], [2, 8], [3, 4], [3, 7], [6, 7
[[1, 3], [1, 6], [2, 3], [2, 7], [3, 4], [5, 7], [7, 8
[[1, 3], [1, 6], [2, 5], [2, 7], [3, 4], [3, 7], [7, 8
[[1, 3], [1, 6], [2, 7], [2, 8], [3, 4], [3, 7], [5, 7
[[1, 3], [1, 7], [2, 3], [2, 5], [3, 4], [6, 7], [7, 8
[[1, 3], [1, 7], [2, 3], [2, 6], [3, 4], [5, 7], [7, 8
[[1, 3], [1, 7], [2, 3], [2, 7], [3, 4], [5, 6], [7, 8
[[1, 3], [1, 7], [2, 3], [2, 7], [3, 4], [5, 8], [6, 7
[[1, 3], [1, 7], [2, 3], [2, 8], [3, 4], [5, 7], [6, 7
[[1, 3], [1, 7], [2, 5], [2, 6], [3, 4], [3, 7], [7, 8
[[1, 3], [1, 7], [2, 5], [2, 7], [3, 4], [3, 6], [7, 8
[[1, 3], [1, 7], [2, 5], [2, 7], [3, 4], [3, 8], [6, 7
[[1, 3], [1, 7], [2, 5], [2, 8], [3, 4], [3, 7], [6, 7
[[1, 3], [1, 7], [2, 6], [2, 7], [3, 4], [3, 5], [7, 8
[[1, 3], [1, 7], [2, 6], [2, 7], [3, 4], [3, 7], [5, 8
[[1, 3], [1, 7], [2, 6], [2, 7], [3, 4], [3, 8], [5, 7
[[1, 3], [1, 7], [2, 6], [2, 8], [3, 4], [3, 7], [5, 7
[[1, 3], [1, 7], [2, 7], [2, 8], [3, 4], [3, 5], [6, 7
[[1, 3], [1, 7], [2, 7], [2, 8], [3, 4], [3, 6], [5, 7
[[1, 3], [1, 7], [2, 7], [2, 8], [3, 4], [3, 7], [5, 6
[[1, 3], [1, 8], [2, 3], [2, 7], [3, 4], [5, 7], [6, 7
[[1, 3], [1, 8], [2, 5], [2, 7], [3, 4], [3, 7], [6, 7
[[1, 3], [1, 8], [2, 6], [2, 7], [3, 4], [3, 7], [5, 7
[[1, 5], [1, 6], [2, 3], [2, 7], [3, 4], [3, 7], [7, 8
[[1, 5], [1, 7], [2, 3], [2, 6], [3, 4], [3, 7], [7, 8
[[1, 5], [1, 7], [2, 3], [2, 7], [3, 4], [3, 6], [7, 8
[[1, 5], [1, 7], [2, 3], [2, 7], [3, 4], [3, 8], [6, 7
[[1, 5], [1, 7], [2, 3], [2, 8], [3, 4], [3, 7], [6, 7
[[1, 5], [1, 7], [2, 6], [2, 7], [3, 4], [3, 7], [3, 8
[[1, 5], [1, 7], [2, 7], [2, 8], [3, 4], [3, 6], [3, 7
[[1, 5], [1, 8], [2, 3], [2, 7], [3, 4], [3, 7], [6, 7
[[1, 6], [1, 7], [2, 3], [2, 5], [3, 4], [3, 7], [7, 8
[[1, 6], [1, 7], [2, 3], [2, 7], [3, 4], [3, 5], [7, 8
[[1, 6], [1, 7], [2, 3], [2, 7], [3, 4], [3, 7], [5, 8
[[1, 6], [1, 7], [2, 3], [2, 7], [3, 4], [3, 8], [5, 7
[[1, 6], [1, 7], [2, 3], [2, 8], [3, 4], [3, 7], [5, 7
[[1, 6], [1, 7], [2, 5], [2, 7], [3, 4], [3, 7], [3, 8
[[1, 6], [1, 7], [2, 7], [2, 8], [3, 4], [3, 5], [3, 7
[[1, 6], [1, 8], [2, 3], [2, 7], [3, 4], [3, 7], [5, 7
[[1, 7], [1, 8], [2, 3], [2, 5], [3, 4], [3, 7], [6, 7
[[1, 7], [1, 8], [2, 3], [2, 6], [3, 4], [3, 7], [5, 7
[[1, 7], [1, 8], [2, 3], [2, 7], [3, 4], [3, 5], [6, 7
[[1, 7], [1, 8], [2, 3], [2, 7], [3, 4], [3, 6], [5, 7
[[1, 7], [1, 8], [2, 3], [2, 7], [3, 4], [3, 7], [5, 6
[[1, 7], [1, 8], [2, 5], [2, 7], [3, 4], [3, 6], [3, 7
[[1, 7], [1, 8], [2, 6], [2, 7], [3, 4], [3, 5], [3, 7]]]

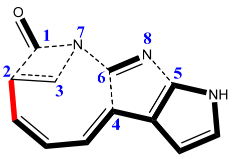

A-h

[[[1, 2], [1, 3], [2, 3], [4, 6], [5, 7], [6, 7], [7, 8
[[1, 2], [1, 3], [2, 3], [4, 7], [5, 6], [6, 7], [7, 8
[[1, 2], [1, 4], [2, 3], [3, 6], [5, 7], [6, 7], [7, 8
[[1, 2], [1, 4], [2, 3], [3, 7], [5, 6], [6, 7], [7, 8
[[1, 2], [1, 5], [2, 3], [3, 6], [4, 7], [6, 7], [7, 8
[[1, 2], [1, 5], [2, 3], [3, 7], [4, 6], [6, 7], [7, 8
[[1, 2], [1, 6], [2, 3], [3, 4], [5, 7], [6, 7], [7, 8
[[1, 2], [1, 6], [2, 3], [3, 5], [4, 7], [6, 7], [7, 8
[[1, 2], [1, 6], [2, 3], [3, 6], [4, 7], [5, 7], [7, 8
[[1, 2], [1, 6], [2, 3], [3, 7], [4, 5], [6, 7], [7, 8
[[1, 2], [1, 6], [2, 3], [3, 7], [4, 6], [5, 7], [7, 8
[[1, 2], [1, 6], [2, 3], [3, 7], [4, 7], [5, 6], [7, 8
[[1, 2], [1, 6], [2, 3], [3, 7], [4, 7], [5, 8], [6, 7
[[1, 2], [1, 6], [2, 3], [3, 7], [4, 8], [5, 7], [6, 7
[[1, 2], [1, 6], [2, 3], [3, 8], [4, 7], [5, 7], [6, 7
[[1, 2], [1, 7], [2, 3], [3, 4], [5, 6], [6, 7], [7, 8
[[1, 2], [1, 7], [2, 3], [3, 5], [4, 6], [6, 7], [7, 8
[[1, 2], [1, 7], [2, 3], [3, 6], [4, 5], [6, 7], [7, 8
[[1, 2], [1, 7], [2, 3], [3, 6], [4, 6], [5, 7], [7, 8
[[1, 2], [1, 7], [2, 3], [3, 6], [4, 7], [5, 6], [7, 8
[[1, 2], [1, 7], [2, 3], [3, 6], [4, 7], [5, 8], [6, 7
[[1, 2], [1, 7], [2, 3], [3, 6], [4, 8], [5, 7], [6, 7
[[1, 2], [1, 7], [2, 3], [3, 7], [4, 6], [5, 6], [7, 8
[[1, 2], [1, 7], [2, 3], [3, 7], [4, 6], [5, 8], [6, 7
[[1, 2], [1, 7], [2, 3], [3, 7], [4, 8], [5, 6], [6, 7
[[1, 2], [1, 7], [2, 3], [3, 8], [4, 6], [5, 7], [6, 7
[[1, 2], [1, 7], [2, 3], [3, 8], [4, 7], [5, 6], [6, 7
[[1, 2], [1, 8], [2, 3], [3, 6], [4, 7], [5, 7], [6, 7
[[1, 2], [1, 8], [2, 3], [3, 7], [4, 6], [5, 7], [6, 7
[[1, 2], [1, 8], [2, 3], [3, 7], [4, 7], [5, 6], [6, 7
[[1, 3], [1, 4], [2, 3], [2, 6], [5, 7], [6, 7], [7, 8
[[1, 3], [1, 4], [2, 3], [2, 7], [5, 6], [6, 7], [7, 8
[[1, 3], [1, 5], [2, 3], [2, 6], [4, 7], [6, 7], [7, 8
[[1, 3], [1, 5], [2, 3], [2, 7], [4, 6], [6, 7], [7, 8
[[1, 3], [1, 6], [2, 3], [2, 4], [5, 7], [6, 7], [7, 8
[[1, 3], [1, 6], [2, 3], [2, 5], [4, 7], [6, 7], [7, 8
[[1, 3], [1, 6], [2, 3], [2, 6], [4, 7], [5, 7], [7, 8
[[1, 3], [1, 6], [2, 3], [2, 7], [4, 5], [6, 7], [7, 8
[[1, 3], [1, 6], [2, 3], [2, 7], [4, 6], [5, 7], [7, 8
[[1, 3], [1, 6], [2, 3], [2, 7], [4, 7], [5, 6], [7, 8
[[1, 3], [1, 6], [2, 3], [2, 7], [4, 7], [5, 8], [6, 7
[[1, 3], [1, 6], [2, 3], [2, 7], [4, 8], [5, 7], [6, 7
[[1, 3], [1, 6], [2, 3], [2, 8], [4, 7], [5, 7], [6, 7
[[1, 3], [1, 7], [2, 3], [2, 4], [5, 6], [6, 7], [7, 8
[[1, 3], [1, 7], [2, 3], [2, 5], [4, 6], [6, 7], [7, 8
[[1, 3], [1, 7], [2, 3], [2, 6], [4, 5], [6, 7], [7, 8
[[1, 3], [1, 7], [2, 3], [2, 6], [4, 6], [5, 7], [7, 8
[[1, 3], [1, 7], [2, 3], [2, 6], [4, 7], [5, 6], [7, 8
[[1, 3], [1, 7], [2, 3], [2, 6], [4, 7], [5, 8], [6, 7
[[1, 3], [1, 7], [2, 3], [2, 6], [4, 8], [5, 7], [6, 7
[[1, 3], [1, 7], [2, 3], [2, 7], [4, 6], [5, 6], [7, 8
[[1, 3], [1, 7], [2, 3], [2, 7], [4, 6], [5, 8], [6, 7
[[1, 3], [1, 7], [2, 3], [2, 7], [4, 8], [5, 6], [6, 7
[[1, 3], [1, 7], [2, 3], [2, 8], [4, 6], [5, 7], [6, 7
[[1, 3], [1, 7], [2, 3], [2, 8], [4, 7], [5, 6], [6, 7
[[1, 3], [1, 8], [2, 3], [2, 6], [4, 7], [5, 7], [6, 7
[[1, 3], [1, 8], [2, 3], [2, 7], [4, 6], [5, 7], [6, 7
[[1, 3], [1, 8], [2, 3], [2, 7], [4, 7], [5, 6], [6, 7
[[1, 4], [1, 5], [2, 3], [2, 6], [3, 7], [6, 7], [7, 8
[[1, 4], [1, 5], [2, 3], [2, 7], [3, 6], [6, 7], [7, 8
[[1, 4], [1, 6], [2, 3], [2, 5], [3, 7], [6, 7], [7, 8
[[1, 4], [1, 6], [2, 3], [2, 6], [3, 7], [5, 7], [7, 8
[[1, 4], [1, 6], [2, 3], [2, 7], [3, 5], [6, 7], [7, 8
[[1, 4], [1, 6], [2, 3], [2, 7], [3, 6], [5, 7], [7, 8
[[1, 4], [1, 6], [2, 3], [2, 7], [3, 7], [5, 6], [7, 8
[[1, 4], [1, 6], [2, 3], [2, 7], [3, 7], [5, 8], [6, 7
[[1, 4], [1, 6], [2, 3], [2, 7], [3, 8], [5, 7], [6, 7
[[1, 4], [1, 6], [2, 3], [2, 8], [3, 7], [5, 7], [6, 7
[[1, 4], [1, 7], [2, 3], [2, 5], [3, 6], [6, 7], [7, 8
[[1, 4], [1, 7], [2, 3], [2, 6], [3, 5], [6, 7], [7, 8
[[1, 4], [1, 7], [2, 3], [2, 6], [3, 6], [5, 7], [7, 8
[[1, 4], [1, 7], [2, 3], [2, 6], [3, 7], [5, 6], [7, 8
[[1, 4], [1, 7], [2, 3], [2, 6], [3, 7], [5, 8], [6, 7
[[1, 4], [1, 7], [2, 3], [2, 6], [3, 8], [5, 7], [6, 7
[[1, 4], [1, 7], [2, 3], [2, 7], [3, 6], [5, 6], [7, 8
[[1, 4], [1, 7], [2, 3], [2, 7], [3, 6], [5, 8], [6, 7
[[1, 4], [1, 7], [2, 3], [2, 7], [3, 8], [5, 6], [6, 7
[[1, 4], [1, 7], [2, 3], [2, 8], [3, 6], [5, 7], [6, 7
[[1, 4], [1, 7], [2, 3], [2, 8], [3, 7], [5, 6], [6, 7
[[1, 4], [1, 8], [2, 3], [2, 6], [3, 7], [5, 7], [6, 7
[[1, 4], [1, 8], [2, 3], [2, 7], [3, 6], [5, 7], [6, 7
[[1, 4], [1, 8], [2, 3], [2, 7], [3, 7], [5, 6], [6, 7
[[1, 5], [1, 6], [2, 3], [2, 4], [3, 7], [6, 7], [7, 8
[[1, 5], [1, 6], [2, 3], [2, 6], [3, 7], [4, 7], [7, 8
[[1, 5], [1, 6], [2, 3], [2, 7], [3, 4], [6, 7], [7, 8
[[1, 5], [1, 6], [2, 3], [2, 7], [3, 6], [4, 7], [7, 8
[[1, 5], [1, 6], [2, 3], [2, 7], [3, 7], [4, 6], [7, 8
[[1, 5], [1, 6], [2, 3], [2, 7], [3, 7], [4, 8], [6, 7
[[1, 5], [1, 6], [2, 3], [2, 7], [3, 8], [4, 7], [6, 7
[[1, 5], [1, 6], [2, 3], [2, 8], [3, 7], [4, 7], [6, 7
[[1, 5], [1, 7], [2, 3], [2, 4], [3, 6], [6, 7], [7, 8
[[1, 5], [1, 7], [2, 3], [2, 6], [3, 4], [6, 7], [7, 8
[[1, 5], [1, 7], [2, 3], [2, 6], [3, 6], [4, 7], [7, 8
[[1, 5], [1, 7], [2, 3], [2, 6], [3, 7], [4, 6], [7, 8
[[1, 5], [1, 7], [2, 3], [2, 6], [3, 7], [4, 8], [6, 7
[[1, 5], [1, 7], [2, 3], [2, 6], [3, 8], [4, 7], [6, 7
[[1, 5], [1, 7], [2, 3], [2, 7], [3, 6], [4, 6], [7, 8
[[1, 5], [1, 7], [2, 3], [2, 7], [3, 6], [4, 8], [6, 7
[[1, 5], [1, 7], [2, 3], [2, 7], [3, 8], [4, 6], [6, 7
[[1, 5], [1, 7], [2, 3], [2, 8], [3, 6], [4, 7], [6, 7
[[1, 5], [1, 7], [2, 3], [2, 8], [3, 7], [4, 6], [6, 7
[[1, 5], [1, 8], [2, 3], [2, 6], [3, 7], [4, 7], [6, 7
[[1, 5], [1, 8], [2, 3], [2, 7], [3, 6], [4, 7], [6, 7
[[1, 5], [1, 8], [2, 3], [2, 7], [3, 7], [4, 6], [6, 7
[[1, 6], [1, 7], [2, 3], [2, 4], [3, 5], [6, 7], [7, 8
[[1, 6], [1, 7], [2, 3], [2, 4], [3, 6], [5, 7], [7, 8
[[1, 6], [1, 7], [2, 3], [2, 4], [3, 7], [5, 6], [7, 8
[[1, 6], [1, 7], [2, 3], [2, 4], [3, 7], [5, 8], [6, 7
[[1, 6], [1, 7], [2, 3], [2, 4], [3, 8], [5, 7], [6, 7
[[1, 6], [1, 7], [2, 3], [2, 5], [3, 4], [6, 7], [7, 8
[[1, 6], [1, 7], [2, 3], [2, 5], [3, 6], [4, 7], [7, 8
[[1, 6], [1, 7], [2, 3], [2, 5], [3, 7], [4, 6], [7, 8
[[1, 6], [1, 7], [2, 3], [2, 5], [3, 7], [4, 8], [6, 7
[[1, 6], [1, 7], [2, 3], [2, 5], [3, 8], [4, 7], [6, 7
[[1, 6], [1, 7], [2, 3], [2, 6], [3, 4], [5, 7], [7, 8
[[1, 6], [1, 7], [2, 3], [2, 6], [3, 5], [4, 7], [7, 8
[[1, 6], [1, 7], [2, 3], [2, 6], [3, 7], [4, 5], [7, 8
[[1, 6], [1, 7], [2, 3], [2, 6], [3, 7], [4, 7], [5, 8
[[1, 6], [1, 7], [2, 3], [2, 6], [3, 7], [4, 8], [5, 7
[[1, 6], [1, 7], [2, 3], [2, 6], [3, 8], [4, 7], [5, 7
[[1, 6], [1, 7], [2, 3], [2, 7], [3, 4], [5, 6], [7, 8
[[1, 6], [1, 7], [2, 3], [2, 7], [3, 4], [5, 8], [6, 7
[[1, 6], [1, 7], [2, 3], [2, 7], [3, 5], [4, 6], [7, 8
[[1, 6], [1, 7], [2, 3], [2, 7], [3, 5], [4, 8], [6, 7
[[1, 6], [1, 7], [2, 3], [2, 7], [3, 6], [4, 5], [7, 8
[[1, 6], [1, 7], [2, 3], [2, 7], [3, 6], [4, 7], [5, 8
[[1, 6], [1, 7], [2, 3], [2, 7], [3, 6], [4, 8], [5, 7
[[1, 6], [1, 7], [2, 3], [2, 7], [3, 7], [4, 6], [5, 8
[[1, 6], [1, 7], [2, 3], [2, 7], [3, 7], [4, 8], [5, 6
[[1, 6], [1, 7], [2, 3], [2, 7], [3, 8], [4, 5], [6, 7
[[1, 6], [1, 7], [2, 3], [2, 7], [3, 8], [4, 6], [5, 7
[[1, 6], [1, 7], [2, 3], [2, 7], [3, 8], [4, 7], [5, 6
[[1, 6], [1, 7], [2, 3], [2, 8], [3, 4], [5, 7], [6, 7
[[1, 6], [1, 7], [2, 3], [2, 8], [3, 5], [4, 7], [6, 7
[[1, 6], [1, 7], [2, 3], [2, 8], [3, 6], [4, 7], [5, 7
[[1, 6], [1, 7], [2, 3], [2, 8], [3, 7], [4, 5], [6, 7
[[1, 6], [1, 7], [2, 3], [2, 8], [3, 7], [4, 6], [5, 7
[[1, 6], [1, 7], [2, 3], [2, 8], [3, 7], [4, 7], [5, 6
[[1, 6], [1, 8], [2, 3], [2, 4], [3, 7], [5, 7], [6, 7
[[1, 6], [1, 8], [2, 3], [2, 5], [3, 7], [4, 7], [6, 7
[[1, 6], [1, 8], [2, 3], [2, 6], [3, 7], [4, 7], [5, 7
[[1, 6], [1, 8], [2, 3], [2, 7], [3, 4], [5, 7], [6, 7
[[1, 6], [1, 8], [2, 3], [2, 7], [3, 5], [4, 7], [6, 7
[[1, 6], [1, 8], [2, 3], [2, 7], [3, 6], [4, 7], [5, 7
[[1, 6], [1, 8], [2, 3], [2, 7], [3, 7], [4, 5], [6, 7
[[1, 6], [1, 8], [2, 3], [2, 7], [3, 7], [4, 6], [5, 7
[[1, 6], [1, 8], [2, 3], [2, 7], [3, 7], [4, 7], [5, 6
[[1, 7], [1, 8], [2, 3], [2, 4], [3, 6], [5, 7], [6, 7
[[1, 7], [1, 8], [2, 3], [2, 4], [3, 7], [5, 6], [6, 7
[[1, 7], [1, 8], [2, 3], [2, 5], [3, 6], [4, 7], [6, 7
[[1, 7], [1, 8], [2, 3], [2, 5], [3, 7], [4, 6], [6, 7
[[1, 7], [1, 8], [2, 3], [2, 6], [3, 4], [5, 7], [6, 7
[[1, 7], [1, 8], [2, 3], [2, 6], [3, 5], [4, 7], [6, 7
[[1, 7], [1, 8], [2, 3], [2, 6], [3, 6], [4, 7], [5, 7
[[1, 7], [1, 8], [2, 3], [2, 6], [3, 7], [4, 5], [6, 7
[[1, 7], [1, 8], [2, 3], [2, 6], [3, 7], [4, 6], [5, 7
[[1, 7], [1, 8], [2, 3], [2, 6], [3, 7], [4, 7], [5, 6
[[1, 7], [1, 8], [2, 3], [2, 7], [3, 4], [5, 6], [6, 7
[[1, 7], [1, 8], [2, 3], [2, 7], [3, 5], [4, 6], [6, 7

[[1, 7], [1, 8], [2, 3], [2, 7], [3, 6], [4, 5], [6, 7
[[1, 7], [1, 8], [2, 3], [2, 7], [3, 6], [4, 6], [5, 7
[[1, 7], [1, 8], [2, 3], [2, 7], [3, 6], [4, 7], [5, 6
[[1, 7], [1, 8], [2, 3], [2, 7], [3, 7], [4, 6], [5, 6

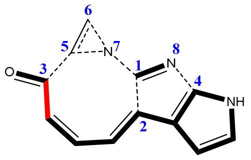

A-h1

[[[1, 2], [1, 3], [4, 5], [5, 6], [5, 7], [6, 7], [7, 8
[[1, 2], [1, 3], [4, 7], [5, 6], [5, 7], [5, 8], [6, 7
[[1, 2], [1, 4], [3, 5], [5, 6], [5, 7], [6, 7], [7, 8
[[1, 2], [1, 4], [3, 7], [5, 6], [5, 7], [5, 8], [6, 7
[[1, 2], [1, 5], [3, 4], [5, 6], [5, 7], [6, 7], [7, 8
[[1, 2], [1, 5], [3, 5], [4, 7], [5, 6], [6, 7], [7, 8
[[1, 2], [1, 5], [3, 6], [4, 7], [5, 6], [5, 7], [7, 8
[[1, 2], [1, 5], [3, 7], [4, 5], [5, 6], [6, 7], [7, 8
[[1, 2], [1, 5], [3, 7], [4, 6], [5, 6], [5, 7], [7, 8
[[1, 2], [1, 5], [3, 7], [4, 7], [5, 6], [5, 7], [6, 8
[[1, 2], [1, 5], [3, 7], [4, 7], [5, 6], [5, 8], [6, 7
[[1, 2], [1, 5], [3, 7], [4, 8], [5, 6], [5, 7], [6, 7
[[1, 2], [1, 5], [3, 8], [4, 7], [5, 6], [5, 7], [6, 7
[[1, 2], [1, 6], [3, 5], [4, 7], [5, 6], [5, 7], [7, 8
[[1, 2], [1, 6], [3, 7], [4, 5], [5, 6], [5, 7], [7, 8
[[1, 2], [1, 6], [3, 7], [4, 7], [5, 6], [5, 7], [5, 8
[[1, 2], [1, 7], [3, 4], [5, 6], [5, 7], [5, 8], [6, 7
[[1, 2], [1, 7], [3, 5], [4, 5], [5, 6], [6, 7], [7, 8
[[1, 2], [1, 7], [3, 5], [4, 6], [5, 6], [5, 7], [7, 8
[[1, 2], [1, 7], [3, 5], [4, 7], [5, 6], [5, 7], [6, 8
[[1, 2], [1, 7], [3, 5], [4, 7], [5, 6], [5, 8], [6, 7
[[1, 2], [1, 7], [3, 5], [4, 8], [5, 6], [5, 7], [6, 7
[[1, 2], [1, 7], [3, 6], [4, 5], [5, 6], [5, 7], [7, 8
[[1, 2], [1, 7], [3, 6], [4, 7], [5, 6], [5, 7], [5, 8
[[1, 2], [1, 7], [3, 7], [4, 5], [5, 6], [5, 7], [6, 8
[[1, 2], [1, 7], [3, 7], [4, 5], [5, 6], [5, 8], [6, 7
[[1, 2], [1, 7], [3, 7], [4, 6], [5, 6], [5, 7], [5, 8
[[1, 2], [1, 7], [3, 8], [4, 5], [5, 6], [5, 7], [6, 7
[[1, 3], [1, 4], [2, 5], [5, 6], [5, 7], [6, 7], [7, 8
[[1, 3], [1, 4], [2, 7], [5, 6], [5, 7], [5, 8], [6, 7
[[1, 3], [1, 5], [2, 4], [5, 6], [5, 7], [6, 7], [7, 8
[[1, 3], [1, 5], [2, 5], [4, 7], [5, 6], [6, 7], [7, 8
[[1, 3], [1, 5], [2, 6], [4, 7], [5, 6], [5, 7], [7, 8
[[1, 3], [1, 5], [2, 7], [4, 5], [5, 6], [6, 7], [7, 8
[[1, 3], [1, 5], [2, 7], [4, 6], [5, 6], [5, 7], [7, 8
[[1, 3], [1, 5], [2, 7], [4, 7], [5, 6], [5, 7], [6, 8
[[1, 3], [1, 5], [2, 7], [4, 7], [5, 6], [5, 8], [6, 7
[[1, 3], [1, 5], [2, 7], [4, 8], [5, 6], [5, 7], [6, 7
[[1, 3], [1, 5], [2, 8], [4, 7], [5, 6], [5, 7], [6, 7
[[1, 3], [1, 6], [2, 5], [4, 7], [5, 6], [5, 7], [7, 8
[[1, 3], [1, 6], [2, 7], [4, 5], [5, 6], [5, 7], [7, 8
[[1, 3], [1, 6], [2, 7], [4, 7], [5, 6], [5, 7], [5, 8
[[1, 3], [1, 7], [2, 4], [5, 6], [5, 7], [5, 8], [6, 7
[[1, 3], [1, 7], [2, 5], [4, 5], [5, 6], [6, 7], [7, 8
[[1, 3], [1, 7], [2, 5], [4, 6], [5, 6], [5, 7], [7, 8
[[1, 3], [1, 7], [2, 5], [4, 7], [5, 6], [5, 7], [6, 8
[[1, 3], [1, 7], [2, 5], [4, 7], [5, 6], [5, 8], [6, 7
[[1, 3], [1, 7], [2, 5], [4, 8], [5, 6], [5, 7], [6, 7
[[1, 3], [1, 7], [2, 6], [4, 5], [5, 6], [5, 7], [7, 8
[[1, 3], [1, 7], [2, 6], [4, 7], [5, 6], [5, 7], [5, 8
[[1, 3], [1, 7], [2, 7], [4, 5], [5, 6], [5, 7], [6, 8
[[1, 3], [1, 7], [2, 7], [4, 5], [5, 6], [5, 8], [6, 7
[[1, 3], [1, 7], [2, 7], [4, 6], [5, 6], [5, 7], [5, 8
[[1, 3], [1, 7], [2, 8], [4, 5], [5, 6], [5, 7], [6, 7
[[1, 4], [1, 5], [2, 3], [5, 6], [5, 7], [6, 7], [7, 8
[[1, 4], [1, 5], [2, 5], [3, 7], [5, 6], [6, 7], [7, 8
[[1, 4], [1, 5], [2, 6], [3, 7], [5, 6], [5, 7], [7, 8
[[1, 4], [1, 5], [2, 7], [3, 5], [5, 6], [6, 7], [7, 8
[[1, 4], [1, 5], [2, 7], [3, 6], [5, 6], [5, 7], [7, 8
[[1, 4], [1, 5], [2, 7], [3, 7], [5, 6], [5, 7], [6, 8
[[1, 4], [1, 5], [2, 7], [3, 7], [5, 6], [5, 8], [6, 7
[[1, 4], [1, 5], [2, 7], [3, 8], [5, 6], [5, 7], [6, 7
[[1, 4], [1, 5], [2, 8], [3, 7], [5, 6], [5, 7], [6, 7

[[1, 4], [1, 6], [2, 5], [3, 7], [5, 6], [5, 7], [7, 8
[[1, 4], [1, 6], [2, 7], [3, 5], [5, 6], [5, 7], [7, 8
[[1, 4], [1, 6], [2, 7], [3, 7], [5, 6], [5, 7], [5, 8
[[1, 4], [1, 7], [2, 3], [5, 6], [5, 7], [5, 8], [6, 7
[[1, 4], [1, 7], [2, 5], [3, 5], [5, 6], [6, 7], [7, 8
[[1, 4], [1, 7], [2, 5], [3, 6], [5, 6], [5, 7], [7, 8
[[1, 4], [1, 7], [2, 5], [3, 7], [5, 6], [5, 7], [6, 8
[[1, 4], [1, 7], [2, 5], [3, 7], [5, 6], [5, 8], [6, 7
[[1, 4], [1, 7], [2, 5], [3, 8], [5, 6], [5, 7], [6, 7
[[1, 4], [1, 7], [2, 6], [3, 5], [5, 6], [5, 7], [7, 8
[[1, 4], [1, 7], [2, 6], [3, 7], [5, 6], [5, 7], [5, 8
[[1, 4], [1, 7], [2, 7], [3, 5], [5, 6], [5, 7], [6, 8
[[1, 4], [1, 7], [2, 7], [3, 5], [5, 6], [5, 8], [6, 7
[[1, 4], [1, 7], [2, 7], [3, 6], [5, 6], [5, 7], [5, 8
[[1, 4], [1, 7], [2, 8], [3, 5], [5, 6], [5, 7], [6, 7
[[1, 5], [1, 6], [2, 3], [4, 7], [5, 6], [5, 7], [7, 8
[[1, 5], [1, 6], [2, 4], [3, 7], [5, 6], [5, 7], [7, 8
[[1, 5], [1, 6], [2, 5], [3, 7], [4, 7], [5, 6], [7, 8
[[1, 5], [1, 6], [2, 7], [3, 4], [5, 6], [5, 7], [7, 8
[[1, 5], [1, 6], [2, 7], [3, 5], [4, 7], [5, 6], [7, 8
[[1, 5], [1, 6], [2, 7], [3, 7], [4, 5], [5, 6], [7, 8
[[1, 5], [1, 6], [2, 7], [3, 7], [4, 7], [5, 6], [5, 8
[[1, 5], [1, 6], [2, 7], [3, 7], [4, 8], [5, 6], [5, 7
[[1, 5], [1, 6], [2, 7], [3, 8], [4, 7], [5, 6], [5, 7
[[1, 5], [1, 6], [2, 8], [3, 7], [4, 7], [5, 6], [5, 7
[[1, 5], [1, 7], [2, 3], [4, 5], [5, 6], [6, 7], [7, 8
[[1, 5], [1, 7], [2, 3], [4, 6], [5, 6], [5, 7], [7, 8
[[1, 5], [1, 7], [2, 3], [4, 7], [5, 6], [5, 7], [6, 8
[[1, 5], [1, 7], [2, 3], [4, 7], [5, 6], [5, 8], [6, 7
[[1, 5], [1, 7], [2, 3], [4, 8], [5, 6], [5, 7], [6, 7
[[1, 5], [1, 7], [2, 4], [3, 5], [5, 6], [6, 7], [7, 8
[[1, 5], [1, 7], [2, 4], [3, 6], [5, 6], [5, 7], [7, 8
[[1, 5], [1, 7], [2, 4], [3, 7], [5, 6], [5, 7], [6, 8
[[1, 5], [1, 7], [2, 4], [3, 7], [5, 6], [5, 8], [6, 7
[[1, 5], [1, 7], [2, 4], [3, 8], [5, 6], [5, 7], [6, 7
[[1, 5], [1, 7], [2, 5], [3, 4], [5, 6], [6, 7], [7, 8
[[1, 5], [1, 7], [2, 5], [3, 6], [4, 7], [5, 6], [7, 8
[[1, 5], [1, 7], [2, 5], [3, 7], [4, 6], [5, 6], [7, 8
[[1, 5], [1, 7], [2, 5], [3, 7], [4, 7], [5, 6], [6, 8
[[1, 5], [1, 7], [2, 5], [3, 7], [4, 8], [5, 6], [6, 7
[[1, 5], [1, 7], [2, 5], [3, 8], [4, 7], [5, 6], [6, 7
[[1, 5], [1, 7], [2, 6], [3, 4], [5, 6], [5, 7], [7, 8
[[1, 5], [1, 7], [2, 6], [3, 5], [4, 7], [5, 6], [7, 8
[[1, 5], [1, 7], [2, 6], [3, 7], [4, 5], [5, 6], [7, 8
[[1, 5], [1, 7], [2, 6], [3, 7], [4, 7], [5, 6], [5, 8
[[1, 5], [1, 7], [2, 6], [3, 7], [4, 8], [5, 6], [5, 7
[[1, 5], [1, 7], [2, 6], [3, 8], [4, 7], [5, 6], [5, 7
[[1, 5], [1, 7], [2, 7], [3, 4], [5, 6], [5, 7], [6, 8
[[1, 5], [1, 7], [2, 7], [3, 4], [5, 6], [5, 8], [6, 7
[[1, 5], [1, 7], [2, 7], [3, 5], [4, 6], [5, 6], [7, 8
[[1, 5], [1, 7], [2, 7], [3, 5], [4, 7], [5, 6], [6, 8
[[1, 5], [1, 7], [2, 7], [3, 5], [4, 8], [5, 6], [6, 7
[[1, 5], [1, 7], [2, 7], [3, 6], [4, 5], [5, 6], [7, 8
[[1, 5], [1, 7], [2, 7], [3, 6], [4, 7], [5, 6], [5, 8
[[1, 5], [1, 7], [2, 7], [3, 6], [4, 8], [5, 6], [5, 7
[[1, 5], [1, 7], [2, 7], [3, 7], [4, 5], [5, 6], [6, 8
[[1, 5], [1, 7], [2, 7], [3, 7], [4, 6], [5, 6], [5, 8
[[1, 5], [1, 7], [2, 7], [3, 8], [4, 5], [5, 6], [6, 7
[[1, 5], [1, 7], [2, 7], [3, 8], [4, 6], [5, 6], [5, 7
[[1, 5], [1, 7], [2, 8], [3, 4], [5, 6], [5, 7], [6, 7
[[1, 5], [1, 7], [2, 8], [3, 5], [4, 7], [5, 6], [6, 7
[[1, 5], [1, 7], [2, 8], [3, 6], [4, 7], [5, 6], [5, 7
[[1, 5], [1, 7], [2, 8], [3, 7], [4, 5], [5, 6], [6, 7
[[1, 5], [1, 7], [2, 8], [3, 7], [4, 6], [5, 6], [5, 7
[[1, 6], [1, 7], [2, 3], [4, 5], [5, 6], [5, 7], [7, 8
[[1, 6], [1, 7], [2, 3], [4, 7], [5, 6], [5, 7], [5, 8
[[1, 6], [1, 7], [2, 4], [3, 5], [5, 6], [5, 7], [7, 8
[[1, 6], [1, 7], [2, 4], [3, 7], [5, 6], [5, 7], [5, 8
[[1, 6], [1, 7], [2, 5], [3, 4], [5, 6], [5, 7], [7, 8
[[1, 6], [1, 7], [2, 5], [3, 5], [4, 7], [5, 6], [7, 8
[[1, 6], [1, 7], [2, 5], [3, 7], [4, 5], [5, 6], [7, 8
[[1, 6], [1, 7], [2, 5], [3, 7], [4, 7], [5, 6], [5, 8
[[1, 6], [1, 7], [2, 5], [3, 7], [4, 8], [5, 6], [5, 7
[[1, 6], [1, 7], [2, 5], [3, 8], [4, 7], [5, 6], [5, 7
[[1, 6], [1, 7], [2, 7], [3, 4], [5, 6], [5, 7], [5, 8
[[1, 6], [1, 7], [2, 7], [3, 5], [4, 5], [5, 6], [7, 8
[[1, 6], [1, 7], [2, 7], [3, 5], [4, 7], [5, 6], [5, 8

[[1, 6], [1, 7], [2, 7], [3, 5], [4, 8], [5, 6], [5, 7
[[1, 6], [1, 7], [2, 7], [3, 7], [4, 5], [5, 6], [5, 8
[[1, 6], [1, 7], [2, 7], [3, 8], [4, 5], [5, 6], [5, 7
[[1, 6], [1, 7], [2, 8], [3, 5], [4, 7], [5, 6], [5, 7
[[1, 6], [1, 7], [2, 8], [3, 7], [4, 5], [5, 6], [5, 7

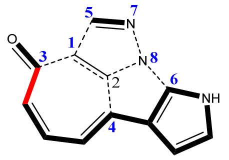

A-g1

[[[1, 2], [1, 3], [1, 4], [2, 5], [2, 8], [6, 8], [7, 8
[[1, 2], [1, 3], [1, 4], [2, 6], [2, 8], [5, 8], [7, 8
[[1, 2], [1, 3], [1, 4], [2, 7], [2, 8], [5, 8], [6, 8
[[1, 2], [1, 3], [1, 5], [2, 4], [2, 8], [6, 8], [7, 8
[[1, 2], [1, 3], [1, 5], [2, 6], [2, 8], [4, 8], [7, 8
[[1, 2], [1, 3], [1, 5], [2, 7], [2, 8], [4, 8], [6, 8
[[1, 2], [1, 3], [1, 6], [2, 4], [2, 8], [5, 8], [7, 8
[[1, 2], [1, 3], [1, 6], [2, 5], [2, 8], [4, 8], [7, 8
[[1, 2], [1, 3], [1, 6], [2, 7], [2, 8], [4, 8], [5, 8
[[1, 2], [1, 3], [1, 7], [2, 4], [2, 8], [5, 8], [6, 8
[[1, 2], [1, 3], [1, 7], [2, 5], [2, 8], [4, 8], [6, 8
[[1, 2], [1, 3], [1, 7], [2, 6], [2, 8], [4, 8], [5, 8
[[1, 2], [1, 3], [1, 8], [2, 4], [2, 5], [6, 8], [7, 8
[[1, 2], [1, 3], [1, 8], [2, 4], [2, 6], [5, 8], [7, 8
[[1, 2], [1, 3], [1, 8], [2, 4], [2, 7], [5, 8], [6, 8
[[1, 2], [1, 3], [1, 8], [2, 4], [2, 8], [5, 6], [7, 8
[[1, 2], [1, 3], [1, 8], [2, 4], [2, 8], [5, 8], [6, 7
[[1, 2], [1, 3], [1, 8], [2, 5], [2, 6], [4, 8], [7, 8
[[1, 2], [1, 3], [1, 8], [2, 5], [2, 7], [4, 8], [6, 8
[[1, 2], [1, 3], [1, 8], [2, 5], [2, 8], [4, 6], [7, 8
[[1, 2], [1, 3], [1, 8], [2, 5], [2, 8], [4, 7], [6, 8
[[1, 2], [1, 3], [1, 8], [2, 5], [2, 8], [4, 8], [6, 7
[[1, 2], [1, 3], [1, 8], [2, 6], [2, 7], [4, 8], [5, 8
[[1, 2], [1, 3], [1, 8], [2, 6], [2, 8], [4, 5], [7, 8
[[1, 2], [1, 3], [1, 8], [2, 6], [2, 8], [4, 7], [5, 8
[[1, 2], [1, 3], [1, 8], [2, 7], [2, 8], [4, 5], [6, 8
[[1, 2], [1, 3], [1, 8], [2, 7], [2, 8], [4, 6], [5, 8
[[1, 2], [1, 3], [1, 8], [2, 7], [2, 8], [4, 8], [5, 6
[[1, 2], [1, 4], [1, 5], [2, 3], [2, 8], [6, 8], [7, 8
[[1, 2], [1, 4], [1, 5], [2, 6], [2, 8], [3, 8], [7, 8
[[1, 2], [1, 4], [1, 5], [2, 7], [2, 8], [3, 8], [6, 8
[[1, 2], [1, 4], [1, 6], [2, 3], [2, 8], [5, 8], [7, 8
[[1, 2], [1, 4], [1, 6], [2, 5], [2, 8], [3, 8], [7, 8
[[1, 2], [1, 4], [1, 6], [2, 7], [2, 8], [3, 8], [5, 8
[[1, 2], [1, 4], [1, 7], [2, 3], [2, 8], [5, 8], [6, 8
[[1, 2], [1, 4], [1, 7], [2, 5], [2, 8], [3, 8], [6, 8
[[1, 2], [1, 4], [1, 7], [2, 6], [2, 8], [3, 8], [5, 8
[[1, 2], [1, 4], [1, 8], [2, 3], [2, 5], [6, 8], [7, 8
[[1, 2], [1, 4], [1, 8], [2, 3], [2, 6], [5, 8], [7, 8
[[1, 2], [1, 4], [1, 8], [2, 3], [2, 7], [5, 8], [6, 8
[[1, 2], [1, 4], [1, 8], [2, 3], [2, 8], [5, 6], [7, 8
[[1, 2], [1, 4], [1, 8], [2, 3], [2, 8], [5, 8], [6, 7
[[1, 2], [1, 4], [1, 8], [2, 5], [2, 6], [3, 8], [7, 8
[[1, 2], [1, 4], [1, 8], [2, 5], [2, 7], [3, 8], [6, 8
[[1, 2], [1, 4], [1, 8], [2, 5], [2, 8], [3, 6], [7, 8
[[1, 2], [1, 4], [1, 8], [2, 5], [2, 8], [3, 7], [6, 8
[[1, 2], [1, 4], [1, 8], [2, 5], [2, 8], [3, 8], [6, 7
[[1, 2], [1, 4], [1, 8], [2, 6], [2, 7], [3, 8], [5, 8
[[1, 2], [1, 4], [1, 8], [2, 6], [2, 8], [3, 5], [7, 8
[[1, 2], [1, 4], [1, 8], [2, 6], [2, 8], [3, 7], [5, 8
[[1, 2], [1, 4], [1, 8], [2, 7], [2, 8], [3, 5], [6, 8
[[1, 2], [1, 4], [1, 8], [2, 7], [2, 8], [3, 6], [5, 8
[[1, 2], [1, 4], [1, 8], [2, 7], [2, 8], [3, 8], [5, 6
[[1, 2], [1, 5], [1, 6], [2, 3], [2, 8], [4, 8], [7, 8
[[1, 2], [1, 5], [1, 6], [2, 4], [2, 8], [3, 8], [7, 8
[[1, 2], [1, 5], [1, 6], [2, 7], [2, 8], [3, 8], [4, 8
[[1, 2], [1, 5], [1, 7], [2, 3], [2, 8], [4, 8], [6, 8
[[1, 2], [1, 5], [1, 7], [2, 4], [2, 8], [3, 8], [6, 8
[[1, 2], [1, 5], [1, 7], [2, 6], [2, 8], [3, 8], [4, 8
[[1, 2], [1, 5], [1, 8], [2, 3], [2, 4], [6, 8], [7, 8
[[1, 2], [1, 5], [1, 8], [2, 3], [2, 6], [4, 8], [7, 8
[[1, 2], [1, 5], [1, 8], [2, 3], [2, 7], [4, 8], [6, 8

[[1, 2], [1, 5], [1, 8], [2, 3], [2, 8], [4, 6], [7, 8
[[1, 2], [1, 5], [1, 8], [2, 3], [2, 8], [4, 7], [6, 8
[[1, 2], [1, 5], [1, 8], [2, 3], [2, 8], [4, 8], [6, 7
[[1, 2], [1, 5], [1, 8], [2, 4], [2, 6], [3, 8], [7, 8
[[1, 2], [1, 5], [1, 8], [2, 4], [2, 7], [3, 8], [6, 8
[[1, 2], [1, 5], [1, 8], [2, 4], [2, 8], [3, 6], [7, 8
[[1, 2], [1, 5], [1, 8], [2, 4], [2, 8], [3, 7], [6, 8
[[1, 2], [1, 5], [1, 8], [2, 4], [2, 8], [3, 8], [6, 7
[[1, 2], [1, 5], [1, 8], [2, 6], [2, 7], [3, 8], [4, 8
[[1, 2], [1, 5], [1, 8], [2, 6], [2, 8], [3, 4], [7, 8
[[1, 2], [1, 5], [1, 8], [2, 6], [2, 8], [3, 7], [4, 8
[[1, 2], [1, 5], [1, 8], [2, 6], [2, 8], [3, 8], [4, 7
[[1, 2], [1, 5], [1, 8], [2, 7], [2, 8], [3, 4], [6, 8
[[1, 2], [1, 5], [1, 8], [2, 7], [2, 8], [3, 6], [4, 8
[[1, 2], [1, 5], [1, 8], [2, 7], [2, 8], [3, 8], [4, 6
[[1, 2], [1, 6], [1, 7], [2, 3], [2, 8], [4, 8], [5, 8
[[1, 2], [1, 6], [1, 7], [2, 4], [2, 8], [3, 8], [5, 8
[[1, 2], [1, 6], [1, 7], [2, 5], [2, 8], [3, 8], [4, 8
[[1, 2], [1, 6], [1, 8], [2, 3], [2, 4], [5, 8], [7, 8
[[1, 2], [1, 6], [1, 8], [2, 3], [2, 5], [4, 8], [7, 8
[[1, 2], [1, 6], [1, 8], [2, 3], [2, 7], [4, 8], [5, 8
[[1, 2], [1, 6], [1, 8], [2, 3], [2, 8], [4, 5], [7, 8
[[1, 2], [1, 6], [1, 8], [2, 3], [2, 8], [4, 7], [5, 8
[[1, 2], [1, 6], [1, 8], [2, 4], [2, 5], [3, 8], [7, 8
[[1, 2], [1, 6], [1, 8], [2, 4], [2, 7], [3, 8], [5, 8
[[1, 2], [1, 6], [1, 8], [2, 4], [2, 8], [3, 5], [7, 8
[[1, 2], [1, 6], [1, 8], [2, 4], [2, 8], [3, 7], [5, 8
[[1, 2], [1, 6], [1, 8], [2, 5], [2, 7], [3, 8], [4, 8
[[1, 2], [1, 6], [1, 8], [2, 5], [2, 8], [3, 4], [7, 8
[[1, 2], [1, 6], [1, 8], [2, 5], [2, 8], [3, 7], [4, 8
[[1, 2], [1, 6], [1, 8], [2, 5], [2, 8], [3, 8], [4, 7
[[1, 2], [1, 6], [1, 8], [2, 7], [2, 8], [3, 4], [5, 8
[[1, 2], [1, 6], [1, 8], [2, 7], [2, 8], [3, 5], [4, 8
[[1, 2], [1, 6], [1, 8], [2, 7], [2, 8], [3, 8], [4, 5
[[1, 2], [1, 7], [1, 8], [2, 3], [2, 4], [5, 8], [6, 8
[[1, 2], [1, 7], [1, 8], [2, 3], [2, 5], [4, 8], [6, 8
[[1, 2], [1, 7], [1, 8], [2, 3], [2, 6], [4, 8], [5, 8
[[1, 2], [1, 7], [1, 8], [2, 3], [2, 8], [4, 5], [6, 8
[[1, 2], [1, 7], [1, 8], [2, 3], [2, 8], [4, 6], [5, 8
[[1, 2], [1, 7], [1, 8], [2, 3], [2, 8], [4, 8], [5, 6
[[1, 2], [1, 7], [1, 8], [2, 4], [2, 5], [3, 8], [6, 8
[[1, 2], [1, 7], [1, 8], [2, 4], [2, 6], [3, 8], [5, 8
[[1, 2], [1, 7], [1, 8], [2, 4], [2, 8], [3, 5], [6, 8
[[1, 2], [1, 7], [1, 8], [2, 4], [2, 8], [3, 6], [5, 8
[[1, 2], [1, 7], [1, 8], [2, 4], [2, 8], [3, 8], [5, 6
[[1, 2], [1, 7], [1, 8], [2, 5], [2, 6], [3, 8], [4, 8
[[1, 2], [1, 7], [1, 8], [2, 5], [2, 8], [3, 4], [6, 8
[[1, 2], [1, 7], [1, 8], [2, 5], [2, 8], [3, 6], [4, 8
[[1, 2], [1, 7], [1, 8], [2, 5], [2, 8], [3, 8], [4, 6
[[1, 2], [1, 7], [1, 8], [2, 6], [2, 8], [3, 4], [5, 8
[[1, 2], [1, 7], [1, 8], [2, 6], [2, 8], [3, 5], [4, 8
[[1, 2], [1, 7], [1, 8], [2, 6], [2, 8], [3, 8], [4, 5

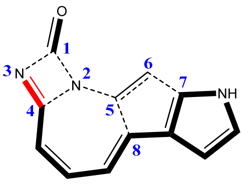

A-h11

[[[1, 2], [1, 3], [2, 4], [2, 5], [5, 6], [5, 7], [6, 8
[[1, 2], [1, 3], [2, 4], [2, 5], [5, 6], [5, 8], [6, 7
[[1, 2], [1, 3], [2, 4], [2, 6], [5, 6], [5, 7], [5, 8
[[1, 2], [1, 3], [2, 5], [2, 6], [4, 5], [5, 6], [7, 8
[[1, 2], [1, 3], [2, 5], [2, 6], [4, 7], [5, 6], [5, 8
[[1, 2], [1, 3], [2, 5], [2, 6], [4, 8], [5, 6], [5, 7
[[1, 2], [1, 3], [2, 5], [2, 7], [4, 5], [5, 6], [6, 8
[[1, 2], [1, 3], [2, 5], [2, 7], [4, 6], [5, 6], [5, 8
[[1, 2], [1, 3], [2, 5], [2, 8], [4, 5], [5, 6], [6, 7
[[1, 2], [1, 3], [2, 5], [2, 8], [4, 6], [5, 6], [5, 7
[[1, 2], [1, 3], [2, 6], [2, 7], [4, 5], [5, 6], [5, 8
[[1, 2], [1, 3], [2, 6], [2, 8], [4, 5], [5, 6], [5, 7
[[1, 2], [1, 4], [2, 3], [2, 5], [5, 6], [5, 7], [6, 8
[[1, 2], [1, 4], [2, 3], [2, 5], [5, 6], [5, 8], [6, 7
[[1, 2], [1, 4], [2, 3], [2, 6], [5, 6], [5, 7], [5, 8
[[1, 2], [1, 4], [2, 5], [2, 6], [3, 5], [5, 6], [7, 8
[[1, 2], [1, 4], [2, 5], [2, 6], [3, 7], [5, 6], [5, 8
[[1, 2], [1, 4], [2, 5], [2, 6], [3, 8], [5, 6], [5, 7
[[1, 2], [1, 4], [2, 5], [2, 7], [3, 5], [5, 6], [6, 8
[[1, 2], [1, 4], [2, 5], [2, 7], [3, 6], [5, 6], [5, 8
[[1, 2], [1, 4], [2, 5], [2, 8], [3, 5], [5, 6], [6, 7
[[1, 2], [1, 4], [2, 5], [2, 8], [3, 6], [5, 6], [5, 7
[[1, 2], [1, 4], [2, 6], [2, 7], [3, 5], [5, 6], [5, 8
[[1, 2], [1, 4], [2, 6], [2, 8], [3, 5], [5, 6], [5, 7
[[1, 2], [1, 5], [2, 3], [2, 4], [5, 6], [5, 7], [6, 8
[[1, 2], [1, 5], [2, 3], [2, 4], [5, 6], [5, 8], [6, 7
[[1, 2], [1, 5], [2, 3], [2, 5], [4, 6], [5, 6], [7, 8
[[1, 2], [1, 5], [2, 3], [2, 5], [4, 7], [5, 6], [6, 8
[[1, 2], [1, 5], [2, 3], [2, 5], [4, 8], [5, 6], [6, 7
[[1, 2], [1, 5], [2, 3], [2, 6], [4, 5], [5, 6], [7, 8
[[1, 2], [1, 5], [2, 3], [2, 6], [4, 7], [5, 6], [5, 8
[[1, 2], [1, 5], [2, 3], [2, 6], [4, 8], [5, 6], [5, 7
[[1, 2], [1, 5], [2, 3], [2, 7], [4, 5], [5, 6], [6, 8
[[1, 2], [1, 5], [2, 3], [2, 7], [4, 6], [5, 6], [5, 8
[[1, 2], [1, 5], [2, 3], [2, 8], [4, 5], [5, 6], [6, 7
[[1, 2], [1, 5], [2, 3], [2, 8], [4, 6], [5, 6], [5, 7
[[1, 2], [1, 5], [2, 4], [2, 5], [3, 6], [5, 6], [7, 8
[[1, 2], [1, 5], [2, 4], [2, 5], [3, 7], [5, 6], [6, 8
[[1, 2], [1, 5], [2, 4], [2, 5], [3, 8], [5, 6], [6, 7
[[1, 2], [1, 5], [2, 4], [2, 6], [3, 5], [5, 6], [7, 8
[[1, 2], [1, 5], [2, 4], [2, 6], [3, 7], [5, 6], [5, 8
[[1, 2], [1, 5], [2, 4], [2, 6], [3, 8], [5, 6], [5, 7
[[1, 2], [1, 5], [2, 4], [2, 7], [3, 5], [5, 6], [6, 8
[[1, 2], [1, 5], [2, 4], [2, 7], [3, 6], [5, 6], [5, 8
[[1, 2], [1, 5], [2, 4], [2, 8], [3, 5], [5, 6], [6, 7
[[1, 2], [1, 5], [2, 4], [2, 8], [3, 6], [5, 6], [5, 7
[[1, 2], [1, 5], [2, 5], [2, 6], [3, 7], [4, 8], [5, 6
[[1, 2], [1, 5], [2, 5], [2, 6], [3, 8], [4, 7], [5, 6
[[1, 2], [1, 5], [2, 5], [2, 7], [3, 6], [4, 8], [5, 6
[[1, 2], [1, 5], [2, 5], [2, 7], [3, 8], [4, 6], [5, 6
[[1, 2], [1, 5], [2, 5], [2, 8], [3, 6], [4, 7], [5, 6
[[1, 2], [1, 5], [2, 5], [2, 8], [3, 7], [4, 6], [5, 6
[[1, 2], [1, 5], [2, 6], [2, 7], [3, 5], [4, 8], [5, 6
[[1, 2], [1, 5], [2, 6], [2, 7], [3, 8], [4, 5], [5, 6
[[1, 2], [1, 5], [2, 6], [2, 8], [3, 5], [4, 7], [5, 6
[[1, 2], [1, 5], [2, 6], [2, 8], [3, 7], [4, 5], [5, 6
[[1, 2], [1, 5], [2, 7], [2, 8], [3, 5], [4, 6], [5, 6
[[1, 2], [1, 5], [2, 7], [2, 8], [3, 6], [4, 5], [5, 6
[[1, 2], [1, 6], [2, 3], [2, 4], [5, 6], [5, 7], [5, 8
[[1, 2], [1, 6], [2, 3], [2, 5], [4, 5], [5, 6], [7, 8
[[1, 2], [1, 6], [2, 3], [2, 5], [4, 7], [5, 6], [5, 8
[[1, 2], [1, 6], [2, 3], [2, 5], [4, 8], [5, 6], [5, 7
[[1, 2], [1, 6], [2, 3], [2, 7], [4, 5], [5, 6], [5, 8
[[1, 2], [1, 6], [2, 3], [2, 8], [4, 5], [5, 6], [5, 7
[[1, 2], [1, 6], [2, 4], [2, 5], [3, 5], [5, 6], [7, 8
[[1, 2], [1, 6], [2, 4], [2, 5], [3, 7], [5, 6], [5, 8
[[1, 2], [1, 6], [2, 4], [2, 5], [3, 8], [5, 6], [5, 7
[[1, 2], [1, 6], [2, 4], [2, 7], [3, 5], [5, 6], [5, 8
[[1, 2], [1, 6], [2, 4], [2, 8], [3, 5], [5, 6], [5, 7
[[1, 2], [1, 6], [2, 5], [2, 7], [3, 5], [4, 8], [5, 6
[[1, 2], [1, 6], [2, 5], [2, 7], [3, 8], [4, 5], [5, 6
[[1, 2], [1, 6], [2, 5], [2, 8], [3, 5], [4, 7], [5, 6
[[1, 2], [1, 6], [2, 5], [2, 8], [3, 7], [4, 5], [5, 6
[[1, 2], [1, 6], [2, 7], [2, 8], [3, 5], [4, 5], [5, 6
[[1, 2], [1, 7], [2, 3], [2, 5], [4, 5], [5, 6], [6, 8
[[1, 2], [1, 7], [2, 3], [2, 5], [4, 6], [5, 6], [5, 8
[[1, 2], [1, 7], [2, 3], [2, 6], [4, 5], [5, 6], [5, 8
[[1, 2], [1, 7], [2, 4], [2, 5], [3, 5], [5, 6], [6, 8
[[1, 2], [1, 7], [2, 4], [2, 5], [3, 6], [5, 6], [5, 8
[[1, 2], [1, 7], [2, 4], [2, 6], [3, 5], [5, 6], [5, 8
[[1, 2], [1, 7], [2, 5], [2, 6], [3, 5], [4, 8], [5, 6
[[1, 2], [1, 7], [2, 5], [2, 6], [3, 8], [4, 5], [5, 6
[[1, 2], [1, 7], [2, 5], [2, 8], [3, 5], [4, 6], [5, 6
[[1, 2], [1, 7], [2, 5], [2, 8], [3, 6], [4, 5], [5, 6
[[1, 2], [1, 7], [2, 6], [2, 8], [3, 5], [4, 5], [5, 6
[[1, 2], [1, 8], [2, 3], [2, 5], [4, 5], [5, 6], [6, 7
[[1, 2], [1, 8], [2, 3], [2, 5], [4, 6], [5, 6], [5, 7
[[1, 2], [1, 8], [2, 3], [2, 6], [4, 5], [5, 6], [5, 7
[[1, 2], [1, 8], [2, 4], [2, 5], [3, 5], [5, 6], [6, 7
[[1, 2], [1, 8], [2, 4], [2, 5], [3, 6], [5, 6], [5, 7
[[1, 2], [1, 8], [2, 4], [2, 6], [3, 5], [5, 6], [5, 7
[[1, 2], [1, 8], [2, 5], [2, 6], [3, 5], [4, 7], [5, 6
[[1, 2], [1, 8], [2, 5], [2, 6], [3, 7], [4, 5], [5, 6
[[1, 2], [1, 8], [2, 5], [2, 7], [3, 5], [4, 6], [5, 6
[[1, 2], [1, 8], [2, 5], [2, 7], [3, 6], [4, 5], [5, 6
[[1, 2], [1, 8], [2, 6], [2, 7], [3, 5], [4, 5], [5, 6
[[1, 3], [1, 4], [2, 5], [2, 6], [2, 7], [5, 6], [5, 8
[[1, 3], [1, 4], [2, 5], [2, 6], [2, 8], [5, 6], [5, 7
[[1, 3], [1, 5], [2, 4], [2, 5], [2, 6], [5, 6], [7, 8
[[1, 3], [1, 5], [2, 4], [2, 5], [2, 7], [5, 6], [6, 8
[[1, 3], [1, 5], [2, 4], [2, 5], [2, 8], [5, 6], [6, 7
[[1, 3], [1, 5], [2, 4], [2, 6], [2, 7], [5, 6], [5, 8
[[1, 3], [1, 5], [2, 4], [2, 6], [2, 8], [5, 6], [5, 7
[[1, 3], [1, 5], [2, 5], [2, 6], [2, 7], [4, 8], [5, 6
[[1, 3], [1, 5], [2, 5], [2, 6], [2, 8], [4, 7], [5, 6
[[1, 3], [1, 5], [2, 5], [2, 7], [2, 8], [4, 6], [5, 6
[[1, 3], [1, 5], [2, 6], [2, 7], [2, 8], [4, 5], [5, 6
[[1, 3], [1, 6], [2, 4], [2, 5], [2, 7], [5, 6], [5, 8
[[1, 3], [1, 6], [2, 4], [2, 5], [2, 8], [5, 6], [5, 7
[[1, 3], [1, 6], [2, 5], [2, 7], [2, 8], [4, 5], [5, 6
[[1, 3], [1, 7], [2, 4], [2, 5], [2, 6], [5, 6], [5, 8
[[1, 3], [1, 7], [2, 5], [2, 6], [2, 8], [4, 5], [5, 6
[[1, 3], [1, 8], [2, 4], [2, 5], [2, 6], [5, 6], [5, 7
[[1, 3], [1, 8], [2, 5], [2, 6], [2, 7], [4, 5], [5, 6
[[1, 4], [1, 5], [2, 3], [2, 5], [2, 6], [5, 6], [7, 8
[[1, 4], [1, 5], [2, 3], [2, 5], [2, 7], [5, 6], [6, 8
[[1, 4], [1, 5], [2, 3], [2, 5], [2, 8], [5, 6], [6, 7
[[1, 4], [1, 5], [2, 3], [2, 6], [2, 7], [5, 6], [5, 8
[[1, 4], [1, 5], [2, 3], [2, 6], [2, 8], [5, 6], [5, 7
[[1, 4], [1, 5], [2, 5], [2, 6], [2, 7], [3, 8], [5, 6
[[1, 4], [1, 5], [2, 5], [2, 6], [2, 8], [3, 7], [5, 6
[[1, 4], [1, 5], [2, 5], [2, 7], [2, 8], [3, 6], [5, 6
[[1, 4], [1, 5], [2, 6], [2, 7], [2, 8], [3, 5], [5, 6
[[1, 4], [1, 6], [2, 3], [2, 5], [2, 7], [5, 6], [5, 8
[[1, 4], [1, 6], [2, 3], [2, 5], [2, 8], [5, 6], [5, 7
[[1, 4], [1, 6], [2, 5], [2, 7], [2, 8], [3, 5], [5, 6
[[1, 4], [1, 7], [2, 3], [2, 5], [2, 6], [5, 6], [5, 8
[[1, 4], [1, 7], [2, 5], [2, 6], [2, 8], [3, 5], [5, 6
[[1, 4], [1, 8], [2, 3], [2, 5], [2, 6], [5, 6], [5, 7
[[1, 4], [1, 8], [2, 5], [2, 6], [2, 7], [3, 5], [5, 6
[[1, 5], [1, 6], [2, 3], [2, 4], [2, 5], [5, 6], [7, 8
[[1, 5], [1, 6], [2, 3], [2, 4], [2, 7], [5, 6], [5, 8
[[1, 5], [1, 6], [2, 3], [2, 4], [2, 8], [5, 6], [5, 7
[[1, 5], [1, 6], [2, 3], [2, 5], [2, 7], [4, 8], [5, 6
[[1, 5], [1, 6], [2, 3], [2, 5], [2, 8], [4, 7], [5, 6
[[1, 5], [1, 6], [2, 3], [2, 7], [2, 8], [4, 5], [5, 6
[[1, 5], [1, 6], [2, 4], [2, 5], [2, 7], [3, 8], [5, 6
[[1, 5], [1, 6], [2, 4], [2, 5], [2, 8], [3, 7], [5, 6
[[1, 5], [1, 6], [2, 4], [2, 7], [2, 8], [3, 5], [5, 6
[[1, 5], [1, 7], [2, 3], [2, 4], [2, 5], [5, 6], [6, 8
[[1, 5], [1, 7], [2, 3], [2, 4], [2, 6], [5, 6], [5, 8
[[1, 5], [1, 7], [2, 3], [2, 5], [2, 6], [4, 8], [5, 6
[[1, 5], [1, 7], [2, 3], [2, 5], [2, 8], [4, 6], [5, 6
[[1, 5], [1, 7], [2, 3], [2, 6], [2, 8], [4, 5], [5, 6
[[1, 5], [1, 7], [2, 4], [2, 5], [2, 6], [3, 8], [5, 6
[[1, 5], [1, 7], [2, 4], [2, 5], [2, 8], [3, 6], [5, 6
[[1, 5], [1, 7], [2, 4], [2, 6], [2, 8], [3, 5], [5, 6
[[1, 5], [1, 8], [2, 3], [2, 4], [2, 5], [5, 6], [6, 7
[[1, 5], [1, 8], [2, 3], [2, 4], [2, 6], [5, 6], [5, 7
[[1, 5], [1, 8], [2, 3], [2, 5], [2, 6], [4, 7], [5, 6
[[1, 5], [1, 8], [2, 3], [2, 5], [2, 7], [4, 6], [5, 6
[[1, 5], [1, 8], [2, 3], [2, 6], [2, 7], [4, 5], [5, 6
[[1, 5], [1, 8], [2, 4], [2, 5], [2, 6], [3, 7], [5, 6
[[1, 5], [1, 8], [2, 4], [2, 5], [2, 7], [3, 6], [5, 6
[[1, 5], [1, 8], [2, 4], [2, 6], [2, 7], [3, 5], [5, 6
[[1, 6], [1, 7], [2, 3], [2, 4], [2, 5], [5, 6], [5, 8
[[1, 6], [1, 7], [2, 3], [2, 5], [2, 8], [4, 5], [5, 6
[[1, 6], [1, 7], [2, 4], [2, 5], [2, 8], [3, 5], [5, 6
[[1, 6], [1, 8], [2, 3], [2, 4], [2, 5], [5, 6], [5, 7
[[1, 6], [1, 8], [2, 3], [2, 5], [2, 7], [4, 5], [5, 6
[[1, 6], [1, 8], [2, 4], [2, 5], [2, 7], [3, 5], [5, 6
[[1, 7], [1, 8], [2, 3], [2, 5], [2, 6], [4, 5], [5, 6
[[1, 7], [1, 8], [2, 4], [2, 5], [2, 6], [3, 5], [5, 6]]]

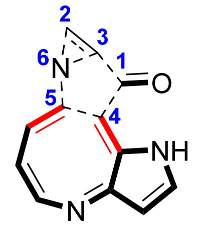

B-i

[[1, 2], [1, 3], [2, 3], [2, 6], [4, 5], [4, 6], [5, 6
[[1, 2], [1, 4], [2, 3], [2, 5], [3, 6], [4, 6], [5, 6
[[1, 2], [1, 4], [2, 3], [2, 6], [3, 5], [4, 6], [5, 6
[[1, 2], [1, 4], [2, 3], [2, 6], [3, 6], [4, 5], [5, 6
[[1, 2], [1, 5], [2, 3], [2, 4], [3, 6], [4, 6], [5, 6
[[1, 2], [1, 5], [2, 3], [2, 6], [3, 4], [4, 6], [5, 6
[[1, 2], [1, 5], [2, 3], [2, 6], [3, 6], [4, 5], [4, 6
[[1, 2], [1, 6], [2, 3], [2, 4], [3, 5], [4, 6], [5, 6
[[1, 2], [1, 6], [2, 3], [2, 4], [3, 6], [4, 5], [5, 6
[[1, 2], [1, 6], [2, 3], [2, 5], [3, 4], [4, 6], [5, 6
[[1, 2], [1, 6], [2, 3], [2, 5], [3, 6], [4, 5], [4, 6
[[1, 2], [1, 6], [2, 3], [2, 6], [3, 4], [4, 5], [5, 6
[[1, 2], [1, 6], [2, 3], [2, 6], [3, 5], [4, 5], [4, 6
[[1, 3], [1, 4], [2, 3], [2, 5], [2, 6], [4, 6], [5, 6
[[1, 3], [1, 5], [2, 3], [2, 4], [2, 6], [4, 6], [5, 6
[[1, 3], [1, 6], [2, 3], [2, 4], [2, 5], [4, 6], [5, 6
[[1, 3], [1, 6], [2, 3], [2, 4], [2, 6], [4, 5], [5, 6
[[1, 3], [1, 6], [2, 3], [2, 5], [2, 6], [4, 5], [4, 6
[[1, 4], [1, 5], [2, 3], [2, 4], [2, 6], [3, 6], [5, 6
[[1, 4], [1, 5], [2, 3], [2, 5], [2, 6], [3, 6], [4, 6
[[1, 4], [1, 6], [2, 3], [2, 4], [2, 5], [3, 6], [5, 6
[[1, 4], [1, 6], [2, 3], [2, 4], [2, 6], [3, 5], [5, 6
[[1, 4], [1, 6], [2, 3], [2, 5], [2, 6], [3, 4], [5, 6
[[1, 4], [1, 6], [2, 3], [2, 5], [2, 6], [3, 5], [4, 6
[[1, 4], [1, 6], [2, 3], [2, 5], [2, 6], [3, 6], [4, 5
[[1, 5], [1, 6], [2, 3], [2, 4], [2, 5], [3, 6], [4, 6
[[1, 5], [1, 6], [2, 3], [2, 4], [2, 6], [3, 4], [5, 6
[[1, 5], [1, 6], [2, 3], [2, 4], [2, 6], [3, 5], [4, 6
[[1, 5], [1, 6], [2, 3], [2, 4], [2, 6], [3, 6], [4, 5
[[1, 5], [1, 6], [2, 3], [2, 5], [2, 6], [3, 4], [4, 6

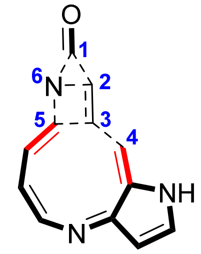

B-j

[[1, 2], [1, 3], [2, 3], [2, 5], [3, 6], [4, 6], [5, 6
[[1, 2], [1, 3], [2, 3], [2, 6], [3, 5], [4, 6], [5, 6
[[1, 2], [1, 3], [2, 3], [2, 6], [3, 6], [4, 5], [5, 6
[[1, 2], [1, 4], [2, 3], [2, 6], [3, 5], [3, 6], [5, 6
[[1, 2], [1, 5], [2, 3], [2, 6], [3, 4], [3, 6], [5, 6
[[1, 2], [1, 5], [2, 3], [2, 6], [3, 5], [3, 6], [4, 6
[[1, 2], [1, 6], [2, 3], [2, 4], [3, 5], [3, 6], [5, 6
[[1, 2], [1, 6], [2, 3], [2, 5], [3, 4], [3, 6], [5, 6
[[1, 2], [1, 6], [2, 3], [2, 5], [3, 5], [3, 6], [4, 6
[[1, 2], [1, 6], [2, 3], [2, 6], [3, 4], [3, 5], [5, 6
[[1, 2], [1, 6], [2, 3], [2, 6], [3, 5], [3, 6], [4, 5
[[1, 3], [1, 4], [2, 3], [2, 5], [2, 6], [3, 6], [5, 6
[[1, 3], [1, 5], [2, 3], [2, 4], [2, 6], [3, 6], [5, 6
[[1, 3], [1, 5], [2, 3], [2, 5], [2, 6], [3, 6], [4, 6
[[1, 3], [1, 6], [2, 3], [2, 4], [2, 5], [3, 6], [5, 6
[[1, 3], [1, 6], [2, 3], [2, 4], [2, 6], [3, 5], [5, 6
[[1, 3], [1, 6], [2, 3], [2, 5], [2, 6], [3, 4], [5, 6
[[1, 3], [1, 6], [2, 3], [2, 5], [2, 6], [3, 5], [4, 6
[[1, 3], [1, 6], [2, 3], [2, 5], [2, 6], [3, 6], [4, 5
[[1, 4], [1, 6], [2, 3], [2, 5], [2, 6], [3, 5], [3, 6

[[1, 5], [1, 6], [2, 3], [2, 4], [2, 6], [3, 5], [3, 6
[[1, 5], [1, 6], [2, 3], [2, 5], [2, 6], [3, 4], [3, 6]]]

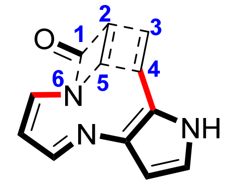

B-k

[[1, 2], [1, 3], [2, 3], [2, 5], [4, 5], [4, 6], [5, 6
[[1, 2], [1, 3], [2, 4], [2, 5], [3, 5], [4, 6], [5, 6
[[1, 2], [1, 3], [2, 4], [2, 6], [3, 5], [4, 5], [5, 6
[[1, 2], [1, 3], [2, 5], [2, 6], [3, 4], [4, 5], [5, 6
[[1, 2], [1, 4], [2, 3], [2, 5], [3, 6], [4, 5], [5, 6
[[1, 2], [1, 4], [2, 3], [2, 6], [3, 5], [4, 5], [5, 6
[[1, 2], [1, 4], [2, 4], [2, 5], [3, 5], [3, 6], [5, 6
[[1, 2], [1, 4], [2, 5], [2, 6], [3, 4], [3, 5], [5, 6
[[1, 2], [1, 5], [2, 3], [2, 4], [3, 5], [4, 6], [5, 6
[[1, 2], [1, 5], [2, 3], [2, 4], [3, 6], [4, 5], [5, 6
[[1, 2], [1, 5], [2, 3], [2, 5], [3, 4], [4, 6], [5, 6
[[1, 2], [1, 5], [2, 3], [2, 5], [3, 6], [4, 5], [4, 6
[[1, 2], [1, 5], [2, 3], [2, 6], [3, 4], [4, 5], [5, 6
[[1, 2], [1, 5], [2, 3], [2, 6], [3, 5], [4, 5], [4, 6
[[1, 2], [1, 5], [2, 4], [2, 5], [3, 4], [3, 6], [5, 6
[[1, 2], [1, 5], [2, 4], [2, 5], [3, 5], [3, 6], [4, 6
[[1, 2], [1, 5], [2, 4], [2, 6], [3, 4], [3, 5], [5, 6
[[1, 2], [1, 5], [2, 4], [2, 6], [3, 5], [3, 6], [4, 5
[[1, 2], [1, 5], [2, 5], [2, 6], [3, 4], [3, 5], [4, 6
[[1, 2], [1, 5], [2, 5], [2, 6], [3, 4], [3, 6], [4, 5
[[1, 2], [1, 6], [2, 3], [2, 4], [3, 5], [4, 5], [5, 6
[[1, 2], [1, 6], [2, 3], [2, 5], [3, 4], [4, 5], [5, 6
[[1, 2], [1, 6], [2, 3], [2, 5], [3, 5], [4, 5], [4, 6
[[1, 2], [1, 6], [2, 4], [2, 5], [3, 4], [3, 5], [5, 6
[[1, 2], [1, 6], [2, 4], [2, 5], [3, 5], [3, 6], [4, 5
[[1, 2], [1, 6], [2, 5], [2, 6], [3, 4], [3, 5], [4, 5
[[1, 3], [1, 4], [2, 3], [2, 5], [2, 6], [4, 5], [5, 6
[[1, 3], [1, 4], [2, 4], [2, 5], [2, 6], [3, 5], [5, 6
[[1, 3], [1, 5], [2, 3], [2, 4], [2, 6], [4, 5], [5, 6
[[1, 3], [1, 5], [2, 3], [2, 5], [2, 6], [4, 5], [4, 6
[[1, 3], [1, 5], [2, 4], [2, 5], [2, 6], [3, 4], [5, 6
[[1, 3], [1, 5], [2, 4], [2, 5], [2, 6], [3, 5], [4, 6
[[1, 3], [1, 6], [2, 3], [2, 4], [2, 5], [4, 5], [5, 6
[[1, 3], [1, 6], [2, 4], [2, 5], [2, 6], [3, 5], [4, 5
[[1, 4], [1, 5], [2, 3], [2, 4], [2, 6], [3, 5], [5, 6
[[1, 4], [1, 5], [2, 3], [2, 5], [2, 6], [3, 4], [5, 6
[[1, 4], [1, 5], [2, 3], [2, 5], [2, 6], [3, 6], [4, 5
[[1, 4], [1, 5], [2, 4], [2, 5], [2, 6], [3, 5], [3, 6
[[1, 4], [1, 6], [2, 3], [2, 4], [2, 5], [3, 5], [5, 6
[[1, 4], [1, 6], [2, 3], [2, 5], [2, 6], [3, 5], [4, 5
[[1, 5], [1, 6], [2, 3], [2, 4], [2, 5], [3, 4], [5, 6
[[1, 5], [1, 6], [2, 3], [2, 4], [2, 5], [3, 5], [4, 6
[[1, 5], [1, 6], [2, 3], [2, 4], [2, 5], [3, 6], [4, 5
[[1, 5], [1, 6], [2, 3], [2, 4], [2, 6], [3, 5], [4, 5
[[1, 5], [1, 6], [2, 3], [2, 5], [2, 6], [3, 4], [4, 5
[[1, 5], [1, 6], [2, 4], [2, 5], [2, 6], [3, 4], [3, 5]]]

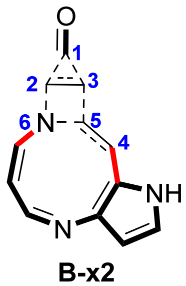

B-x2

[[1, 2], [1, 3], [2, 3], [2, 5], [3, 6], [4, 5], [5, 6
[[1, 2], [1, 3], [2, 3], [2, 6], [3, 5], [4, 5], [5, 6
[[1, 2], [1, 3], [2, 4], [2, 5], [3, 5], [3, 6], [5, 6

[[1, 2], [1, 3], [2, 5], [2, 6], [3, 4], [3, 5], [5, 6
[[1, 2], [1, 5], [2, 3], [2, 4], [3, 5], [3, 6], [5, 6
[[1, 2], [1, 5], [2, 3], [2, 5], [3, 4], [3, 6], [5, 6
[[1, 2], [1, 5], [2, 3], [2, 6], [3, 5], [3, 6], [4, 5
[[1, 2], [1, 5], [2, 5], [2, 6], [3, 4], [3, 5], [3, 6
[[1, 2], [1, 6], [2, 3], [2, 5], [3, 4], [3, 5], [5, 6
[[1, 2], [1, 6], [2, 3], [2, 5], [3, 5], [3, 6], [4, 5
[[1, 3], [1, 5], [2, 3], [2, 4], [2, 6], [3, 5], [5, 6
[[1, 3], [1, 5], [2, 3], [2, 5], [2, 6], [3, 4], [5, 6
[[1, 3], [1, 5], [2, 3], [2, 5], [2, 6], [3, 6], [4, 5
[[1, 3], [1, 5], [2, 4], [2, 5], [2, 6], [3, 5], [3, 6
[[1, 3], [1, 6], [2, 3], [2, 4], [2, 5], [3, 5], [5, 6
[[1, 3], [1, 6], [2, 3], [2, 5], [2, 6], [3, 5], [4, 5
[[1, 5], [1, 6], [2, 3], [2, 4], [2, 5], [3, 5], [3, 6
[[1, 5], [1, 6], [2, 3], [2, 5], [2, 6], [3, 4], [3, 5]]]

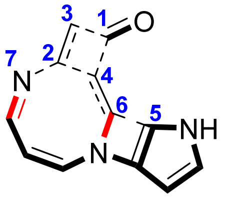

B-l

[[1, 2], [1, 3], [2, 3], [2, 4], [4, 5], [4, 6], [6, 7
[[1, 2], [1, 3], [2, 3], [2, 4], [4, 6], [4, 7], [5, 6
[[1, 2], [1, 3], [2, 3], [2, 6], [4, 5], [4, 6], [4, 7
[[1, 2], [1, 3], [2, 4], [2, 5], [3, 6], [4, 6], [4, 7
[[1, 2], [1, 3], [2, 4], [2, 6], [3, 4], [4, 5], [6, 7
[[1, 2], [1, 3], [2, 4], [2, 6], [3, 4], [4, 6], [5, 7
[[1, 2], [1, 3], [2, 4], [2, 6], [3, 4], [4, 7], [5, 6
[[1, 2], [1, 3], [2, 4], [2, 6], [3, 6], [4, 5], [4, 7
[[1, 2], [1, 3], [2, 4], [2, 7], [3, 6], [4, 5], [4, 6
[[1, 2], [1, 3], [2, 5], [2, 6], [3, 4], [4, 6], [4, 7
[[1, 2], [1, 3], [2, 6], [2, 7], [3, 4], [4, 5], [4, 6
[[1, 2], [1, 4], [2, 3], [2, 4], [3, 5], [4, 6], [6, 7
[[1, 2], [1, 4], [2, 3], [2, 4], [3, 6], [4, 5], [6, 7
[[1, 2], [1, 4], [2, 3], [2, 4], [3, 6], [4, 6], [5, 7
[[1, 2], [1, 4], [2, 3], [2, 4], [3, 6], [4, 7], [5, 6
[[1, 2], [1, 4], [2, 3], [2, 4], [3, 7], [4, 6], [5, 6
[[1, 2], [1, 4], [2, 3], [2, 5], [3, 4], [4, 6], [6, 7
[[1, 2], [1, 4], [2, 3], [2, 5], [3, 6], [4, 6], [4, 7
[[1, 2], [1, 4], [2, 3], [2, 6], [3, 4], [4, 5], [6, 7
[[1, 2], [1, 4], [2, 3], [2, 6], [3, 4], [4, 6], [5, 7
[[1, 2], [1, 4], [2, 3], [2, 6], [3, 4], [4, 7], [5, 6
[[1, 2], [1, 4], [2, 3], [2, 6], [3, 5], [4, 6], [4, 7
[[1, 2], [1, 4], [2, 3], [2, 6], [3, 7], [4, 5], [4, 6
[[1, 2], [1, 4], [2, 3], [2, 7], [3, 4], [4, 6], [5, 6
[[1, 2], [1, 4], [2, 3], [2, 7], [3, 6], [4, 5], [4, 6
[[1, 2], [1, 4], [2, 4], [2, 5], [3, 4], [3, 6], [6, 7
[[1, 2], [1, 4], [2, 4], [2, 5], [3, 6], [3, 7], [4, 6
[[1, 2], [1, 4], [2, 4], [2, 6], [3, 4], [3, 5], [6, 7
[[1, 2], [1, 4], [2, 4], [2, 6], [3, 4], [3, 6], [5, 7
[[1, 2], [1, 4], [2, 4], [2, 6], [3, 4], [3, 7], [5, 6
[[1, 2], [1, 4], [2, 4], [2, 6], [3, 5], [3, 6], [4, 7
[[1, 2], [1, 4], [2, 4], [2, 6], [3, 6], [3, 7], [4, 5
[[1, 2], [1, 4], [2, 4], [2, 7], [3, 4], [3, 6], [5, 6
[[1, 2], [1, 4], [2, 4], [2, 7], [3, 5], [3, 6], [4, 6
[[1, 2], [1, 4], [2, 5], [2, 6], [3, 4], [3, 6], [4, 7
[[1, 2], [1, 4], [2, 5], [2, 6], [3, 4], [3, 7], [4, 6
[[1, 2], [1, 4], [2, 6], [2, 7], [3, 4], [3, 5], [4, 6
[[1, 2], [1, 4], [2, 6], [2, 7], [3, 4], [3, 6], [4, 5
[[1, 2], [1, 5], [2, 3], [2, 4], [3, 4], [4, 6], [6, 7
[[1, 2], [1, 5], [2, 3], [2, 4], [3, 6], [4, 6], [4, 7
[[1, 2], [1, 5], [2, 3], [2, 6], [3, 4], [4, 6], [4, 7
[[1, 2], [1, 5], [2, 4], [2, 6], [3, 4], [3, 6], [4, 7
[[1, 2], [1, 5], [2, 4], [2, 6], [3, 4], [3, 7], [4, 6
[[1, 2], [1, 5], [2, 4], [2, 7], [3, 4], [3, 6], [4, 6
[[1, 2], [1, 6], [2, 3], [2, 4], [3, 4], [4, 6], [5, 7
[[1, 2], [1, 6], [2, 3], [2, 4], [3, 5], [4, 6], [4, 7
[[1, 2], [1, 6], [2, 3], [2, 4], [3, 6], [4, 5], [4, 7
[[1, 2], [1, 6], [2, 3], [2, 4], [3, 7], [4, 5], [4, 6
[[1, 2], [1, 6], [2, 3], [2, 5], [3, 4], [4, 6], [4, 7
[[1, 2], [1, 6], [2, 3], [2, 6], [3, 4], [4, 5], [4, 7

[[1, 2], [1, 6], [2, 3], [2, 7], [3, 4], [4, 5], [4, 6
[[1, 2], [1, 6], [2, 4], [2, 5], [3, 4], [3, 6], [4, 7
[[1, 2], [1, 6], [2, 4], [2, 5], [3, 4], [3, 7], [4, 6
[[1, 2], [1, 6], [2, 4], [2, 6], [3, 4], [3, 5], [4, 7
[[1, 2], [1, 6], [2, 4], [2, 6], [3, 4], [3, 7], [4, 5
[[1, 2], [1, 6], [2, 4], [2, 7], [3, 4], [3, 6], [4, 5
[[1, 2], [1, 7], [2, 3], [2, 4], [3, 4], [4, 6], [5, 6
[[1, 2], [1, 7], [2, 3], [2, 4], [3, 6], [4, 5], [4, 6
[[1, 2], [1, 7], [2, 3], [2, 6], [3, 4], [4, 5], [4, 6
[[1, 2], [1, 7], [2, 4], [2, 5], [3, 4], [3, 6], [4, 6
[[1, 2], [1, 7], [2, 4], [2, 6], [3, 4], [3, 5], [4, 6
[[1, 2], [1, 7], [2, 4], [2, 6], [3, 4], [3, 6], [4, 5
[[1, 3], [1, 4], [2, 3], [2, 4], [2, 5], [4, 6], [6, 7
[[1, 3], [1, 4], [2, 3], [2, 4], [2, 6], [4, 6], [5, 7
[[1, 3], [1, 4], [2, 3], [2, 4], [2, 7], [4, 6], [5, 6
[[1, 3], [1, 4], [2, 3], [2, 6], [2, 7], [4, 5], [4, 6
[[1, 3], [1, 4], [2, 4], [2, 5], [2, 6], [3, 4], [6, 7
[[1, 3], [1, 4], [2, 4], [2, 5], [2, 6], [3, 6], [4, 7
[[1, 3], [1, 4], [2, 4], [2, 5], [2, 7], [3, 6], [4, 6
[[1, 3], [1, 4], [2, 4], [2, 6], [2, 7], [3, 4], [5, 6
[[1, 3], [1, 4], [2, 4], [2, 6], [2, 7], [3, 5], [4, 6
[[1, 3], [1, 4], [2, 4], [2, 6], [2, 7], [3, 6], [4, 5
[[1, 3], [1, 4], [2, 5], [2, 6], [2, 7], [3, 4], [4, 6
[[1, 3], [1, 5], [2, 3], [2, 4], [2, 6], [4, 6], [4, 7
[[1, 3], [1, 5], [2, 4], [2, 6], [2, 7], [3, 4], [4, 6
[[1, 3], [1, 6], [2, 3], [2, 4], [2, 5], [4, 6], [4, 7
[[1, 3], [1, 6], [2, 3], [2, 4], [2, 7], [4, 5], [4, 6
[[1, 3], [1, 6], [2, 4], [2, 5], [2, 6], [3, 4], [4, 7
[[1, 3], [1, 6], [2, 4], [2, 6], [2, 7], [3, 4], [4, 5
[[1, 3], [1, 7], [2, 3], [2, 4], [2, 6], [4, 5], [4, 6
[[1, 3], [1, 7], [2, 4], [2, 5], [2, 6], [3, 4], [4, 6
[[1, 4], [1, 5], [2, 3], [2, 4], [2, 6], [3, 4], [6, 7
[[1, 4], [1, 5], [2, 3], [2, 4], [2, 6], [3, 6], [4, 7
[[1, 4], [1, 5], [2, 3], [2, 4], [2, 6], [3, 7], [4, 6
[[1, 4], [1, 5], [2, 3], [2, 4], [2, 7], [3, 6], [4, 6
[[1, 4], [1, 5], [2, 3], [2, 6], [2, 7], [3, 4], [4, 6
[[1, 4], [1, 5], [2, 4], [2, 6], [2, 7], [3, 4], [3, 6
[[1, 4], [1, 6], [2, 3], [2, 4], [2, 5], [3, 6], [4, 7
[[1, 4], [1, 6], [2, 3], [2, 4], [2, 5], [3, 7], [4, 6
[[1, 4], [1, 6], [2, 3], [2, 4], [2, 6], [3, 4], [5, 7
[[1, 4], [1, 6], [2, 3], [2, 4], [2, 7], [3, 6], [4, 5
[[1, 4], [1, 6], [2, 3], [2, 5], [2, 6], [3, 4], [4, 7
[[1, 4], [1, 6], [2, 3], [2, 5], [2, 7], [3, 4], [4, 6
[[1, 4], [1, 6], [2, 3], [2, 6], [2, 7], [3, 4], [4, 5
[[1, 4], [1, 6], [2, 4], [2, 5], [2, 6], [3, 4], [3, 7
[[1, 4], [1, 6], [2, 4], [2, 5], [2, 7], [3, 4], [3, 6
[[1, 4], [1, 6], [2, 4], [2, 6], [2, 7], [3, 4], [3, 5
[[1, 4], [1, 7], [2, 3], [2, 4], [2, 5], [3, 6], [4, 6
[[1, 4], [1, 7], [2, 3], [2, 4], [2, 6], [3, 4], [5, 6
[[1, 4], [1, 7], [2, 3], [2, 4], [2, 6], [3, 5], [4, 6
[[1, 4], [1, 7], [2, 3], [2, 4], [2, 6], [3, 6], [4, 5
[[1, 4], [1, 7], [2, 3], [2, 5], [2, 6], [3, 4], [4, 6
[[1, 4], [1, 7], [2, 4], [2, 5], [2, 6], [3, 4], [3, 6
[[1, 5], [1, 6], [2, 3], [2, 4], [2, 6], [3, 4], [4, 7
[[1, 5], [1, 6], [2, 3], [2, 4], [2, 7], [3, 4], [4, 6
[[1, 5], [1, 7], [2, 3], [2, 4], [2, 6], [3, 4], [4, 6
[[1, 6], [1, 7], [2, 3], [2, 4], [2, 5], [3, 4], [4, 6
[[1, 6], [1, 7], [2, 3], [2, 4], [2, 6], [3, 4], [4, 5]]]

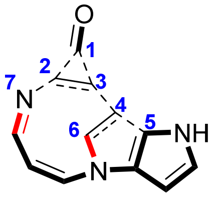

B-x3

[[1, 2], [1, 3], [2, 3], [2, 4], [3, 4], [4, 6], [5, 7
[[1, 2], [1, 3], [2, 3], [2, 4], [3, 5], [4, 6], [4, 7
[[1, 2], [1, 3], [2, 3], [2, 4], [3, 7], [4, 5], [4, 6
[[1, 2], [1, 3], [2, 3], [2, 5], [3, 4], [4, 6], [4, 7
[[1, 2], [1, 3], [2, 3], [2, 7], [3, 4], [4, 5], [4, 6
[[1, 2], [1, 3], [2, 4], [2, 5], [3, 4], [3, 6], [4, 7
[[1, 2], [1, 3], [2, 4], [2, 7], [3, 4], [3, 6], [4, 5

[[1, 2], [1, 4], [2, 3], [2, 4], [3, 5], [3, 7], [4, 6
[[1, 2], [1, 4], [2, 3], [2, 5], [3, 4], [3, 7], [4, 6
[[1, 2], [1, 4], [2, 3], [2, 7], [3, 4], [3, 5], [4, 6
[[1, 2], [1, 4], [2, 4], [2, 5], [3, 4], [3, 6], [3, 7
[[1, 2], [1, 4], [2, 4], [2, 7], [3, 4], [3, 5], [3, 6
[[1, 2], [1, 5], [2, 3], [2, 4], [3, 4], [3, 7], [4, 6
[[1, 2], [1, 7], [2, 3], [2, 4], [3, 4], [3, 5], [4, 6
[[1, 3], [1, 4], [2, 3], [2, 4], [2, 5], [3, 7], [4, 6
[[1, 3], [1, 4], [2, 3], [2, 4], [2, 7], [3, 5], [4, 6
[[1, 3], [1, 4], [2, 3], [2, 5], [2, 7], [3, 4], [4, 6
[[1, 3], [1, 4], [2, 4], [2, 5], [2, 7], [3, 4], [3, 6
[[1, 3], [1, 5], [2, 3], [2, 4], [2, 7], [3, 4], [4, 6
[[1, 3], [1, 7], [2, 3], [2, 4], [2, 5], [3, 4], [4, 6

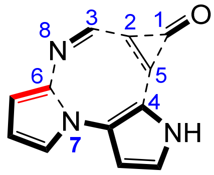

B-n

[[1, 2], [1, 3], [2, 4], [2, 5], [5, 6], [5, 7], [6, 8
[[1, 2], [1, 3], [2, 4], [2, 5], [5, 6], [5, 8], [6, 7
[[1, 2], [1, 3], [2, 5], [2, 6], [4, 5], [5, 6], [7, 8
[[1, 2], [1, 3], [2, 5], [2, 6], [4, 5], [5, 7], [6, 8
[[1, 2], [1, 3], [2, 5], [2, 6], [4, 5], [5, 8], [6, 7
[[1, 2], [1, 3], [2, 5], [2, 6], [4, 6], [5, 7], [5, 8
[[1, 2], [1, 3], [2, 5], [2, 6], [4, 7], [5, 6], [5, 8
[[1, 2], [1, 3], [2, 5], [2, 6], [4, 8], [5, 6], [5, 7
[[1, 2], [1, 3], [2, 5], [2, 7], [4, 5], [5, 6], [6, 8
[[1, 2], [1, 3], [2, 5], [2, 7], [4, 6], [5, 6], [5, 8
[[1, 2], [1, 3], [2, 5], [2, 8], [4, 5], [5, 6], [6, 7
[[1, 2], [1, 3], [2, 5], [2, 8], [4, 6], [5, 6], [5, 7
[[1, 2], [1, 4], [2, 3], [2, 5], [5, 6], [5, 7], [6, 8
[[1, 2], [1, 4], [2, 3], [2, 5], [5, 6], [5, 8], [6, 7
[[1, 2], [1, 4], [2, 5], [2, 6], [3, 5], [5, 6], [7, 8
[[1, 2], [1, 4], [2, 5], [2, 6], [3, 5], [5, 7], [6, 8
[[1, 2], [1, 4], [2, 5], [2, 6], [3, 5], [5, 8], [6, 7
[[1, 2], [1, 4], [2, 5], [2, 6], [3, 6], [5, 7], [5, 8
[[1, 2], [1, 4], [2, 5], [2, 6], [3, 7], [5, 6], [5, 8
[[1, 2], [1, 4], [2, 5], [2, 7], [3, 5], [5, 6], [6, 8
[[1, 2], [1, 4], [2, 5], [2, 7], [3, 6], [5, 6], [5, 8
[[1, 2], [1, 4], [2, 5], [2, 8], [3, 5], [5, 6], [6, 7
[[1, 2], [1, 4], [2, 5], [2, 8], [3, 6], [5, 6], [5, 7
[[1, 2], [1, 5], [2, 3], [2, 5], [4, 5], [6, 7], [6, 8
[[1, 2], [1, 5], [2, 3], [2, 5], [4, 6], [5, 6], [7, 8
[[1, 2], [1, 5], [2, 3], [2, 5], [4, 6], [5, 7], [6, 8
[[1, 2], [1, 5], [2, 3], [2, 5], [4, 7], [5, 6], [6, 8
[[1, 2], [1, 5], [2, 3], [2, 5], [4, 8], [5, 6], [6, 7
[[1, 2], [1, 5], [2, 4], [2, 5], [3, 5], [6, 7], [6, 8
[[1, 2], [1, 5], [2, 4], [2, 5], [3, 6], [5, 6], [7, 8
[[1, 2], [1, 5], [2, 4], [2, 5], [3, 6], [5, 7], [6, 8
[[1, 2], [1, 5], [2, 4], [2, 5], [3, 6], [5, 8], [6, 7
[[1, 2], [1, 5], [2, 4], [2, 5], [3, 7], [5, 6], [6, 8
[[1, 2], [1, 5], [2, 5], [2, 6], [3, 4], [5, 6], [7, 8
[[1, 2], [1, 5], [2, 5], [2, 6], [3, 4], [5, 7], [6, 8
[[1, 2], [1, 5], [2, 5], [2, 6], [3, 4], [5, 8], [6, 7
[[1, 2], [1, 5], [2, 5], [2, 6], [3, 5], [4, 6], [7, 8
[[1, 2], [1, 5], [2, 5], [2, 6], [3, 5], [4, 7], [6, 8
[[1, 2], [1, 5], [2, 5], [2, 6], [3, 5], [4, 8], [6, 7
[[1, 2], [1, 5], [2, 5], [2, 6], [3, 6], [4, 5], [7, 8
[[1, 2], [1, 5], [2, 5], [2, 6], [3, 6], [4, 7], [5, 8
[[1, 2], [1, 5], [2, 5], [2, 6], [3, 6], [4, 8], [5, 7
[[1, 2], [1, 5], [2, 5], [2, 6], [3, 7], [4, 5], [6, 8
[[1, 2], [1, 5], [2, 5], [2, 6], [3, 7], [4, 6], [5, 8
[[1, 2], [1, 5], [2, 5], [2, 6], [3, 7], [4, 8], [5, 6
[[1, 2], [1, 5], [2, 5], [2, 7], [3, 4], [5, 6], [6, 8
[[1, 2], [1, 5], [2, 5], [2, 7], [3, 5], [4, 6], [6, 8
[[1, 2], [1, 5], [2, 5], [2, 7], [3, 6], [4, 5], [6, 8
[[1, 2], [1, 5], [2, 5], [2, 7], [3, 6], [4, 6], [5, 8
[[1, 2], [1, 5], [2, 5], [2, 7], [3, 6], [4, 8], [5, 6
[[1, 2], [1, 5], [2, 5], [2, 8], [3, 4], [5, 6], [6, 7
[[1, 2], [1, 5], [2, 5], [2, 8], [3, 6], [4, 5], [6, 7
[[1, 2], [1, 5], [2, 5], [2, 8], [3, 6], [4, 6], [5, 7

[[1, 2], [1, 5], [2, 5], [2, 8], [3, 6], [4, 7], [5, 6
[[1, 2], [1, 5], [2, 5], [2, 8], [3, 7], [4, 6], [5, 6
[[1, 2], [1, 6], [2, 3], [2, 5], [4, 5], [5, 6], [7, 8
[[1, 2], [1, 6], [2, 3], [2, 5], [4, 5], [5, 7], [6, 8
[[1, 2], [1, 6], [2, 3], [2, 5], [4, 5], [5, 8], [6, 7
[[1, 2], [1, 6], [2, 3], [2, 5], [4, 6], [5, 7], [5, 8
[[1, 2], [1, 6], [2, 3], [2, 5], [4, 7], [5, 6], [5, 8
[[1, 2], [1, 6], [2, 3], [2, 5], [4, 8], [5, 6], [5, 7
[[1, 2], [1, 6], [2, 4], [2, 5], [3, 5], [5, 6], [7, 8
[[1, 2], [1, 6], [2, 4], [2, 5], [3, 5], [5, 7], [6, 8
[[1, 2], [1, 6], [2, 4], [2, 5], [3, 5], [5, 8], [6, 7
[[1, 2], [1, 6], [2, 4], [2, 5], [3, 6], [5, 7], [5, 8
[[1, 2], [1, 6], [2, 4], [2, 5], [3, 7], [5, 6], [5, 8
[[1, 2], [1, 6], [2, 5], [2, 6], [3, 4], [5, 7], [5, 8
[[1, 2], [1, 6], [2, 5], [2, 6], [3, 5], [4, 5], [7, 8
[[1, 2], [1, 6], [2, 5], [2, 6], [3, 5], [4, 7], [5, 8
[[1, 2], [1, 6], [2, 5], [2, 6], [3, 5], [4, 8], [5, 7
[[1, 2], [1, 6], [2, 5], [2, 6], [3, 7], [4, 5], [5, 8
[[1, 2], [1, 6], [2, 5], [2, 7], [3, 4], [5, 6], [5, 8
[[1, 2], [1, 6], [2, 5], [2, 7], [3, 5], [4, 5], [6, 8
[[1, 2], [1, 6], [2, 5], [2, 7], [3, 5], [4, 6], [5, 8
[[1, 2], [1, 6], [2, 5], [2, 7], [3, 5], [4, 8], [5, 6
[[1, 2], [1, 6], [2, 5], [2, 7], [3, 6], [4, 5], [5, 8
[[1, 2], [1, 6], [2, 5], [2, 8], [3, 4], [5, 6], [5, 7
[[1, 2], [1, 6], [2, 5], [2, 8], [3, 5], [4, 5], [6, 7
[[1, 2], [1, 6], [2, 5], [2, 8], [3, 5], [4, 6], [5, 7
[[1, 2], [1, 6], [2, 5], [2, 8], [3, 5], [4, 7], [5, 6
[[1, 2], [1, 6], [2, 5], [2, 8], [3, 6], [4, 5], [5, 7
[[1, 2], [1, 6], [2, 5], [2, 8], [3, 7], [4, 5], [5, 6
[[1, 2], [1, 7], [2, 3], [2, 5], [4, 5], [5, 6], [6, 8
[[1, 2], [1, 7], [2, 3], [2, 5], [4, 6], [5, 6], [5, 8
[[1, 2], [1, 7], [2, 4], [2, 5], [3, 5], [5, 6], [6, 8
[[1, 2], [1, 7], [2, 4], [2, 5], [3, 6], [5, 6], [5, 8
[[1, 2], [1, 7], [2, 5], [2, 6], [3, 4], [5, 6], [5, 8
[[1, 2], [1, 7], [2, 5], [2, 6], [3, 5], [4, 5], [6, 8
[[1, 2], [1, 7], [2, 5], [2, 6], [3, 5], [4, 6], [5, 8
[[1, 2], [1, 7], [2, 5], [2, 6], [3, 5], [4, 8], [5, 6
[[1, 2], [1, 7], [2, 5], [2, 6], [3, 6], [4, 5], [5, 8
[[1, 2], [1, 7], [2, 5], [2, 8], [3, 5], [4, 6], [5, 6
[[1, 2], [1, 7], [2, 5], [2, 8], [3, 6], [4, 5], [5, 6
[[1, 2], [1, 8], [2, 3], [2, 5], [4, 5], [5, 6], [6, 7
[[1, 2], [1, 8], [2, 3], [2, 5], [4, 6], [5, 6], [5, 7
[[1, 2], [1, 8], [2, 4], [2, 5], [3, 5], [5, 6], [6, 7
[[1, 2], [1, 8], [2, 4], [2, 5], [3, 6], [5, 6], [5, 7
[[1, 2], [1, 8], [2, 5], [2, 6], [3, 4], [5, 6], [5, 7
[[1, 2], [1, 8], [2, 5], [2, 6], [3, 5], [4, 5], [6, 7
[[1, 2], [1, 8], [2, 5], [2, 6], [3, 5], [4, 6], [5, 7
[[1, 2], [1, 8], [2, 5], [2, 6], [3, 5], [4, 7], [5, 6
[[1, 2], [1, 8], [2, 5], [2, 6], [3, 6], [4, 5], [5, 7
[[1, 2], [1, 8], [2, 5], [2, 6], [3, 7], [4, 5], [5, 6
[[1, 2], [1, 8], [2, 5], [2, 7], [3, 5], [4, 6], [5, 6
[[1, 2], [1, 8], [2, 5], [2, 7], [3, 6], [4, 5], [5, 6
[[1, 3], [1, 4], [2, 5], [2, 6], [2, 7], [5, 6], [5, 8
[[1, 3], [1, 4], [2, 5], [2, 6], [2, 8], [5, 6], [5, 7
[[1, 3], [1, 5], [2, 4], [2, 5], [2, 6], [5, 6], [7, 8
[[1, 3], [1, 5], [2, 4], [2, 5], [2, 6], [5, 7], [6, 8
[[1, 3], [1, 5], [2, 4], [2, 5], [2, 6], [5, 8], [6, 7
[[1, 3], [1, 5], [2, 4], [2, 5], [2, 7], [5, 6], [6, 8
[[1, 3], [1, 5], [2, 4], [2, 5], [2, 8], [5, 6], [6, 7
[[1, 3], [1, 5], [2, 5], [2, 6], [2, 7], [4, 5], [6, 8
[[1, 3], [1, 5], [2, 5], [2, 6], [2, 7], [4, 6], [5, 8
[[1, 3], [1, 5], [2, 5], [2, 6], [2, 7], [4, 8], [5, 6
[[1, 3], [1, 5], [2, 5], [2, 6], [2, 8], [4, 5], [6, 7
[[1, 3], [1, 5], [2, 5], [2, 6], [2, 8], [4, 6], [5, 7
[[1, 3], [1, 5], [2, 5], [2, 6], [2, 8], [4, 7], [5, 6
[[1, 3], [1, 5], [2, 5], [2, 7], [2, 8], [4, 6], [5, 6
[[1, 3], [1, 6], [2, 4], [2, 5], [2, 6], [5, 7], [5, 8
[[1, 3], [1, 6], [2, 4], [2, 5], [2, 7], [5, 6], [5, 8
[[1, 3], [1, 6], [2, 4], [2, 5], [2, 8], [5, 6], [5, 7
[[1, 3], [1, 6], [2, 5], [2, 6], [2, 7], [4, 5], [5, 8
[[1, 3], [1, 6], [2, 5], [2, 6], [2, 8], [4, 5], [5, 7
[[1, 3], [1, 6], [2, 5], [2, 7], [2, 8], [4, 5], [5, 6
[[1, 3], [1, 7], [2, 4], [2, 5], [2, 6], [5, 6], [5, 8
[[1, 3], [1, 7], [2, 5], [2, 6], [2, 8], [4, 5], [5, 6
[[1, 4], [1, 5], [2, 3], [2, 5], [2, 6], [5, 6], [7, 8
[[1, 4], [1, 5], [2, 3], [2, 5], [2, 6], [5, 7], [6, 8
[[1, 4], [1, 5], [2, 3], [2, 5], [2, 6], [5, 8], [6, 7

[[1, 4], [1, 5], [2, 3], [2, 5], [2, 7], [5, 6], [6, 8
[[1, 4], [1, 5], [2, 3], [2, 5], [2, 8], [5, 6], [6, 7
[[1, 4], [1, 5], [2, 5], [2, 6], [2, 7], [3, 5], [6, 8
[[1, 4], [1, 5], [2, 5], [2, 6], [2, 7], [3, 6], [5, 8
[[1, 4], [1, 5], [2, 5], [2, 6], [2, 8], [3, 5], [6, 7
[[1, 4], [1, 5], [2, 5], [2, 6], [2, 8], [3, 6], [5, 7
[[1, 4], [1, 5], [2, 5], [2, 6], [2, 8], [3, 7], [5, 6
[[1, 4], [1, 5], [2, 5], [2, 7], [2, 8], [3, 6], [5, 6
[[1, 4], [1, 6], [2, 3], [2, 5], [2, 6], [5, 7], [5, 8
[[1, 4], [1, 6], [2, 3], [2, 5], [2, 7], [5, 6], [5, 8
[[1, 4], [1, 6], [2, 3], [2, 5], [2, 8], [5, 6], [5, 7
[[1, 4], [1, 6], [2, 5], [2, 6], [2, 7], [3, 5], [5, 8
[[1, 4], [1, 6], [2, 5], [2, 6], [2, 8], [3, 5], [5, 7
[[1, 4], [1, 6], [2, 5], [2, 7], [2, 8], [3, 5], [5, 6
[[1, 4], [1, 7], [2, 3], [2, 5], [2, 6], [5, 6], [5, 8
[[1, 4], [1, 7], [2, 5], [2, 6], [2, 8], [3, 5], [5, 6
[[1, 4], [1, 8], [2, 3], [2, 5], [2, 6], [5, 6], [5, 7
[[1, 4], [1, 8], [2, 5], [2, 6], [2, 7], [3, 5], [5, 6
[[1, 5], [1, 6], [2, 3], [2, 4], [2, 5], [5, 6], [7, 8
[[1, 5], [1, 6], [2, 3], [2, 4], [2, 5], [5, 7], [6, 8
[[1, 5], [1, 6], [2, 3], [2, 4], [2, 5], [5, 8], [6, 7
[[1, 5], [1, 6], [2, 3], [2, 5], [2, 6], [4, 5], [7, 8
[[1, 5], [1, 6], [2, 3], [2, 5], [2, 6], [4, 7], [5, 8
[[1, 5], [1, 6], [2, 3], [2, 5], [2, 6], [4, 8], [5, 7
[[1, 5], [1, 6], [2, 3], [2, 5], [2, 7], [4, 5], [6, 8
[[1, 5], [1, 6], [2, 3], [2, 5], [2, 7], [4, 6], [5, 8
[[1, 5], [1, 6], [2, 3], [2, 5], [2, 7], [4, 8], [5, 6
[[1, 5], [1, 6], [2, 3], [2, 5], [2, 8], [4, 5], [6, 7
[[1, 5], [1, 6], [2, 3], [2, 5], [2, 8], [4, 6], [5, 7
[[1, 5], [1, 6], [2, 3], [2, 5], [2, 8], [4, 7], [5, 6
[[1, 5], [1, 6], [2, 4], [2, 5], [2, 6], [3, 5], [7, 8
[[1, 5], [1, 6], [2, 4], [2, 5], [2, 6], [3, 7], [5, 8
[[1, 5], [1, 6], [2, 4], [2, 5], [2, 7], [3, 5], [6, 8
[[1, 5], [1, 6], [2, 4], [2, 5], [2, 7], [3, 6], [5, 8
[[1, 5], [1, 6], [2, 4], [2, 5], [2, 8], [3, 5], [6, 7
[[1, 5], [1, 6], [2, 4], [2, 5], [2, 8], [3, 6], [5, 7
[[1, 5], [1, 6], [2, 4], [2, 5], [2, 8], [3, 7], [5, 6
[[1, 5], [1, 6], [2, 5], [2, 6], [2, 7], [3, 4], [5, 8
[[1, 5], [1, 6], [2, 5], [2, 6], [2, 7], [3, 5], [4, 8
[[1, 5], [1, 6], [2, 5], [2, 6], [2, 8], [3, 4], [5, 7
[[1, 5], [1, 6], [2, 5], [2, 6], [2, 8], [3, 5], [4, 7
[[1, 5], [1, 6], [2, 5], [2, 6], [2, 8], [3, 7], [4, 5
[[1, 5], [1, 6], [2, 5], [2, 7], [2, 8], [3, 4], [5, 6
[[1, 5], [1, 6], [2, 5], [2, 7], [2, 8], [3, 5], [4, 6
[[1, 5], [1, 6], [2, 5], [2, 7], [2, 8], [3, 6], [4, 5
[[1, 5], [1, 7], [2, 3], [2, 4], [2, 5], [5, 6], [6, 8
[[1, 5], [1, 7], [2, 3], [2, 5], [2, 6], [4, 5], [6, 8
[[1, 5], [1, 7], [2, 3], [2, 5], [2, 6], [4, 6], [5, 8
[[1, 5], [1, 7], [2, 3], [2, 5], [2, 6], [4, 8], [5, 6
[[1, 5], [1, 7], [2, 3], [2, 5], [2, 8], [4, 6], [5, 6
[[1, 5], [1, 7], [2, 4], [2, 5], [2, 6], [3, 5], [6, 8
[[1, 5], [1, 7], [2, 4], [2, 5], [2, 6], [3, 6], [5, 8
[[1, 5], [1, 7], [2, 4], [2, 5], [2, 8], [3, 6], [5, 6
[[1, 5], [1, 7], [2, 5], [2, 6], [2, 8], [3, 4], [5, 6
[[1, 5], [1, 7], [2, 5], [2, 6], [2, 8], [3, 5], [4, 6
[[1, 5], [1, 7], [2, 5], [2, 6], [2, 8], [3, 6], [4, 5
[[1, 5], [1, 8], [2, 3], [2, 4], [2, 5], [5, 6], [6, 7
[[1, 5], [1, 8], [2, 3], [2, 5], [2, 6], [4, 5], [6, 7
[[1, 5], [1, 8], [2, 3], [2, 5], [2, 6], [4, 6], [5, 7
[[1, 5], [1, 8], [2, 3], [2, 5], [2, 6], [4, 7], [5, 6
[[1, 5], [1, 8], [2, 3], [2, 5], [2, 7], [4, 6], [5, 6
[[1, 5], [1, 8], [2, 4], [2, 5], [2, 6], [3, 5], [6, 7
[[1, 5], [1, 8], [2, 4], [2, 5], [2, 6], [3, 6], [5, 7
[[1, 5], [1, 8], [2, 4], [2, 5], [2, 6], [3, 7], [5, 6
[[1, 5], [1, 8], [2, 4], [2, 5], [2, 7], [3, 6], [5, 6
[[1, 5], [1, 8], [2, 5], [2, 6], [2, 7], [3, 4], [5, 6
[[1, 5], [1, 8], [2, 5], [2, 6], [2, 7], [3, 5], [4, 6
[[1, 5], [1, 8], [2, 5], [2, 6], [2, 7], [3, 6], [4, 5
[[1, 6], [1, 7], [2, 3], [2, 4], [2, 5], [5, 6], [5, 8
[[1, 6], [1, 7], [2, 3], [2, 5], [2, 6], [4, 5], [5, 8
[[1, 6], [1, 7], [2, 3], [2, 5], [2, 8], [4, 5], [5, 6
[[1, 6], [1, 7], [2, 4], [2, 5], [2, 6], [3, 5], [5, 8
[[1, 6], [1, 7], [2, 4], [2, 5], [2, 8], [3, 5], [5, 6
[[1, 6], [1, 7], [2, 5], [2, 6], [2, 8], [3, 5], [4, 5
[[1, 6], [1, 8], [2, 3], [2, 4], [2, 5], [5, 6], [5, 7
[[1, 6], [1, 8], [2, 3], [2, 5], [2, 6], [4, 5], [5, 7
[[1, 6], [1, 8], [2, 3], [2, 5], [2, 7], [4, 5], [5, 6

[[1, 6], [1, 8], [2, 4], [2, 5], [2, 6], [3, 5], [5, 7
[[1, 6], [1, 8], [2, 4], [2, 5], [2, 7], [3, 5], [5, 6
[[1, 6], [1, 8], [2, 5], [2, 6], [2, 7], [3, 5], [4, 5
[[1, 7], [1, 8], [2, 3], [2, 5], [2, 6], [4, 5], [5, 6
[[1, 7], [1, 8], [2, 4], [2, 5], [2, 6], [3, 5], [5, 6]]]

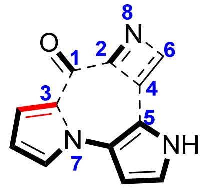

B-o

[[1, 2], [1, 3], [2, 3], [4, 5], [4, 6], [4, 7], [6, 8
[[1, 2], [1, 3], [2, 3], [4, 5], [4, 6], [4, 8], [6, 7
[[1, 2], [1, 3], [2, 3], [4, 6], [4, 7], [4, 8], [5, 6
[[1, 2], [1, 3], [2, 4], [3, 4], [4, 6], [5, 6], [7, 8
[[1, 2], [1, 3], [2, 4], [3, 4], [4, 6], [5, 7], [6, 8
[[1, 2], [1, 3], [2, 4], [3, 4], [4, 6], [5, 8], [6, 7
[[1, 2], [1, 3], [2, 4], [3, 5], [4, 6], [4, 7], [6, 8
[[1, 2], [1, 3], [2, 4], [3, 5], [4, 6], [4, 8], [6, 7
[[1, 2], [1, 3], [2, 4], [3, 6], [4, 5], [4, 6], [7, 8
[[1, 2], [1, 3], [2, 4], [3, 6], [4, 6], [4, 7], [5, 8
[[1, 2], [1, 3], [2, 4], [3, 6], [4, 6], [4, 8], [5, 7
[[1, 2], [1, 3], [2, 4], [3, 7], [4, 5], [4, 6], [6, 8
[[1, 2], [1, 3], [2, 4], [3, 7], [4, 6], [4, 8], [5, 6
[[1, 2], [1, 3], [2, 4], [3, 8], [4, 5], [4, 6], [6, 7
[[1, 2], [1, 3], [2, 4], [3, 8], [4, 6], [4, 7], [5, 6
[[1, 2], [1, 3], [2, 5], [3, 4], [4, 6], [4, 7], [6, 8
[[1, 2], [1, 3], [2, 5], [3, 4], [4, 6], [4, 8], [6, 7
[[1, 2], [1, 3], [2, 5], [3, 6], [4, 6], [4, 7], [4, 8
[[1, 2], [1, 3], [2, 6], [3, 4], [4, 5], [4, 6], [7, 8
[[1, 2], [1, 3], [2, 6], [3, 4], [4, 6], [4, 7], [5, 8
[[1, 2], [1, 3], [2, 6], [3, 4], [4, 6], [4, 8], [5, 7
[[1, 2], [1, 3], [2, 6], [3, 5], [4, 6], [4, 7], [4, 8
[[1, 2], [1, 3], [2, 6], [3, 7], [4, 5], [4, 6], [4, 8
[[1, 2], [1, 3], [2, 6], [3, 8], [4, 5], [4, 6], [4, 7
[[1, 2], [1, 3], [2, 7], [3, 4], [4, 5], [4, 6], [6, 8
[[1, 2], [1, 3], [2, 7], [3, 4], [4, 6], [4, 8], [5, 6
[[1, 2], [1, 3], [2, 7], [3, 6], [4, 5], [4, 6], [4, 8
[[1, 2], [1, 4], [2, 3], [3, 4], [4, 6], [5, 6], [7, 8
[[1, 2], [1, 4], [2, 3], [3, 4], [4, 6], [5, 7], [6, 8
[[1, 2], [1, 4], [2, 3], [3, 4], [4, 6], [5, 8], [6, 7
[[1, 2], [1, 4], [2, 3], [3, 5], [4, 6], [4, 7], [6, 8
[[1, 2], [1, 4], [2, 3], [3, 5], [4, 6], [4, 8], [6, 7
[[1, 2], [1, 4], [2, 3], [3, 6], [4, 5], [4, 6], [7, 8
[[1, 2], [1, 4], [2, 3], [3, 6], [4, 6], [4, 7], [5, 8
[[1, 2], [1, 4], [2, 3], [3, 6], [4, 6], [4, 8], [5, 7
[[1, 2], [1, 4], [2, 3], [3, 7], [4, 5], [4, 6], [6, 8
[[1, 2], [1, 4], [2, 3], [3, 7], [4, 6], [4, 8], [5, 6
[[1, 2], [1, 4], [2, 3], [3, 8], [4, 5], [4, 6], [6, 7
[[1, 2], [1, 4], [2, 3], [3, 8], [4, 6], [4, 7], [5, 6
[[1, 2], [1, 4], [2, 4], [3, 5], [3, 6], [4, 6], [7, 8
[[1, 2], [1, 4], [2, 4], [3, 5], [3, 8], [4, 6], [6, 7
[[1, 2], [1, 4], [2, 4], [3, 6], [3, 7], [4, 6], [5, 8
[[1, 2], [1, 4], [2, 4], [3, 6], [3, 8], [4, 6], [5, 7
[[1, 2], [1, 4], [2, 4], [3, 7], [3, 8], [4, 6], [5, 6
[[1, 2], [1, 4], [2, 5], [3, 4], [3, 6], [4, 6], [7, 8
[[1, 2], [1, 4], [2, 5], [3, 4], [3, 7], [4, 6], [6, 8
[[1, 2], [1, 4], [2, 5], [3, 4], [3, 8], [4, 6], [6, 7
[[1, 2], [1, 4], [2, 5], [3, 6], [3, 7], [4, 6], [4, 8
[[1, 2], [1, 4], [2, 5], [3, 6], [3, 8], [4, 6], [4, 7
[[1, 2], [1, 4], [2, 6], [3, 4], [3, 5], [4, 6], [7, 8
[[1, 2], [1, 4], [2, 6], [3, 4], [3, 7], [4, 6], [5, 8
[[1, 2], [1, 4], [2, 6], [3, 4], [3, 8], [4, 6], [5, 7
[[1, 2], [1, 4], [2, 6], [3, 5], [3, 8], [4, 6], [4, 7
[[1, 2], [1, 4], [2, 6], [3, 7], [3, 8], [4, 5], [4, 6
[[1, 2], [1, 4], [2, 7], [3, 4], [3, 5], [4, 6], [6, 8
[[1, 2], [1, 4], [2, 7], [3, 4], [3, 6], [4, 6], [5, 8
[[1, 2], [1, 4], [2, 7], [3, 4], [3, 8], [4, 6], [5, 6
[[1, 2], [1, 4], [2, 7], [3, 5], [3, 6], [4, 6], [4, 8
[[1, 2], [1, 4], [2, 7], [3, 6], [3, 8], [4, 5], [4, 6
[[1, 2], [1, 5], [2, 3], [3, 4], [4, 6], [4, 7], [6, 8

[[1, 2], [1, 5], [2, 3], [3, 4], [4, 6], [4, 8], [6, 7
[[1, 2], [1, 5], [2, 3], [3, 6], [4, 6], [4, 7], [4, 8
[[1, 2], [1, 5], [2, 4], [3, 4], [3, 6], [4, 6], [7, 8
[[1, 2], [1, 5], [2, 4], [3, 4], [3, 7], [4, 6], [6, 8
[[1, 2], [1, 5], [2, 4], [3, 4], [3, 8], [4, 6], [6, 7
[[1, 2], [1, 5], [2, 4], [3, 6], [3, 7], [4, 6], [4, 8
[[1, 2], [1, 5], [2, 4], [3, 6], [3, 8], [4, 6], [4, 7
[[1, 2], [1, 5], [2, 6], [3, 4], [3, 7], [4, 6], [4, 8
[[1, 2], [1, 5], [2, 6], [3, 4], [3, 8], [4, 6], [4, 7
[[1, 2], [1, 5], [2, 7], [3, 4], [3, 6], [4, 6], [4, 8
[[1, 2], [1, 6], [2, 3], [3, 4], [4, 5], [4, 6], [7, 8
[[1, 2], [1, 6], [2, 3], [3, 4], [4, 6], [4, 7], [5, 8
[[1, 2], [1, 6], [2, 3], [3, 4], [4, 6], [4, 8], [5, 7
[[1, 2], [1, 6], [2, 3], [3, 5], [4, 6], [4, 7], [4, 8
[[1, 2], [1, 6], [2, 3], [3, 7], [4, 5], [4, 6], [4, 8
[[1, 2], [1, 6], [2, 3], [3, 8], [4, 5], [4, 6], [4, 7
[[1, 2], [1, 6], [2, 4], [3, 4], [3, 5], [4, 6], [7, 8
[[1, 2], [1, 6], [2, 4], [3, 4], [3, 7], [4, 6], [5, 8
[[1, 2], [1, 6], [2, 4], [3, 4], [3, 8], [4, 6], [5, 7
[[1, 2], [1, 6], [2, 4], [3, 5], [3, 8], [4, 6], [4, 7
[[1, 2], [1, 6], [2, 4], [3, 7], [3, 8], [4, 5], [4, 6
[[1, 2], [1, 6], [2, 5], [3, 4], [3, 7], [4, 6], [4, 8
[[1, 2], [1, 6], [2, 5], [3, 4], [3, 8], [4, 6], [4, 7
[[1, 2], [1, 6], [2, 7], [3, 4], [3, 5], [4, 6], [4, 8
[[1, 2], [1, 6], [2, 7], [3, 4], [3, 8], [4, 5], [4, 6
[[1, 2], [1, 7], [2, 3], [3, 4], [4, 5], [4, 6], [6, 8
[[1, 2], [1, 7], [2, 3], [3, 4], [4, 6], [4, 8], [5, 6
[[1, 2], [1, 7], [2, 3], [3, 6], [4, 5], [4, 6], [4, 8
[[1, 2], [1, 7], [2, 4], [3, 4], [3, 5], [4, 6], [6, 8
[[1, 2], [1, 7], [2, 4], [3, 4], [3, 6], [4, 6], [5, 8
[[1, 2], [1, 7], [2, 4], [3, 4], [3, 8], [4, 6], [5, 6
[[1, 2], [1, 7], [2, 4], [3, 5], [3, 6], [4, 6], [4, 8
[[1, 2], [1, 7], [2, 4], [3, 6], [3, 8], [4, 5], [4, 6
[[1, 2], [1, 7], [2, 5], [3, 4], [3, 6], [4, 6], [4, 8
[[1, 2], [1, 7], [2, 6], [3, 4], [3, 5], [4, 6], [4, 8
[[1, 2], [1, 7], [2, 6], [3, 4], [3, 8], [4, 5], [4, 6
[[1, 2], [1, 8], [2, 3], [3, 4], [4, 5], [4, 6], [6, 7
[[1, 2], [1, 8], [2, 3], [3, 4], [4, 6], [4, 7], [5, 6
[[1, 2], [1, 8], [2, 3], [3, 6], [4, 5], [4, 6], [4, 7
[[1, 2], [1, 8], [2, 4], [3, 4], [3, 5], [4, 6], [6, 7
[[1, 2], [1, 8], [2, 4], [3, 4], [3, 6], [4, 6], [5, 7
[[1, 2], [1, 8], [2, 4], [3, 4], [3, 7], [4, 6], [5, 6
[[1, 2], [1, 8], [2, 4], [3, 5], [3, 6], [4, 6], [4, 7
[[1, 2], [1, 8], [2, 4], [3, 6], [3, 7], [4, 5], [4, 6
[[1, 2], [1, 8], [2, 5], [3, 4], [3, 6], [4, 6], [4, 7
[[1, 2], [1, 8], [2, 6], [3, 4], [3, 5], [4, 6], [4, 7
[[1, 2], [1, 8], [2, 6], [3, 4], [3, 7], [4, 5], [4, 6
[[1, 2], [1, 8], [2, 7], [3, 4], [3, 6], [4, 5], [4, 6
[[1, 3], [1, 4], [2, 3], [2, 4], [4, 6], [5, 6], [7, 8
[[1, 3], [1, 4], [2, 3], [2, 4], [4, 6], [5, 7], [6, 8
[[1, 3], [1, 4], [2, 3], [2, 4], [4, 6], [5, 8], [6, 7
[[1, 3], [1, 4], [2, 3], [2, 5], [4, 6], [4, 7], [6, 8
[[1, 3], [1, 4], [2, 3], [2, 5], [4, 6], [4, 8], [6, 7
[[1, 3], [1, 4], [2, 3], [2, 6], [4, 5], [4, 6], [7, 8
[[1, 3], [1, 4], [2, 3], [2, 6], [4, 6], [4, 7], [5, 8
[[1, 3], [1, 4], [2, 3], [2, 6], [4, 6], [4, 8], [5, 7
[[1, 3], [1, 4], [2, 3], [2, 7], [4, 5], [4, 6], [6, 8
[[1, 3], [1, 4], [2, 3], [2, 7], [4, 6], [4, 8], [5, 6
[[1, 3], [1, 4], [2, 4], [2, 5], [3, 6], [4, 6], [7, 8
[[1, 3], [1, 4], [2, 4], [2, 5], [3, 7], [4, 6], [6, 8
[[1, 3], [1, 4], [2, 4], [2, 5], [3, 8], [4, 6], [6, 7
[[1, 3], [1, 4], [2, 4], [2, 6], [3, 5], [4, 6], [7, 8
[[1, 3], [1, 4], [2, 4], [2, 6], [3, 7], [4, 6], [5, 8
[[1, 3], [1, 4], [2, 4], [2, 6], [3, 8], [4, 6], [5, 7
[[1, 3], [1, 4], [2, 4], [2, 7], [3, 5], [4, 6], [6, 8
[[1, 3], [1, 4], [2, 4], [2, 7], [3, 6], [4, 6], [5, 8
[[1, 3], [1, 4], [2, 4], [2, 7], [3, 8], [4, 6], [5, 6
[[1, 3], [1, 4], [2, 5], [2, 6], [3, 4], [4, 6], [7, 8
[[1, 3], [1, 4], [2, 5], [2, 6], [3, 7], [4, 6], [4, 8
[[1, 3], [1, 4], [2, 5], [2, 6], [3, 8], [4, 6], [4, 7
[[1, 3], [1, 4], [2, 5], [2, 7], [3, 4], [4, 6], [6, 8
[[1, 3], [1, 4], [2, 5], [2, 7], [3, 6], [4, 6], [4, 8
[[1, 3], [1, 4], [2, 6], [2, 7], [3, 4], [4, 6], [5, 8
[[1, 3], [1, 4], [2, 6], [2, 7], [3, 5], [4, 6], [4, 8
[[1, 3], [1, 4], [2, 6], [2, 7], [3, 8], [4, 5], [4, 6
[[1, 3], [1, 5], [2, 3], [2, 4], [4, 6], [4, 7], [6, 8
[[1, 3], [1, 5], [2, 3], [2, 4], [4, 6], [4, 8], [6, 7

[[1, 3], [1, 5], [2, 3], [2, 6], [4, 6], [4, 7], [4, 8
[[1, 3], [1, 5], [2, 4], [2, 6], [3, 4], [4, 6], [7, 8
[[1, 3], [1, 5], [2, 4], [2, 6], [3, 8], [4, 6], [4, 7
[[1, 3], [1, 5], [2, 4], [2, 7], [3, 4], [4, 6], [6, 8
[[1, 3], [1, 5], [2, 4], [2, 7], [3, 6], [4, 6], [4, 8
[[1, 3], [1, 5], [2, 6], [2, 7], [3, 4], [4, 6], [4, 8
[[1, 3], [1, 6], [2, 3], [2, 4], [4, 5], [4, 6], [7, 8
[[1, 3], [1, 6], [2, 3], [2, 4], [4, 6], [4, 7], [5, 8
[[1, 3], [1, 6], [2, 3], [2, 4], [4, 6], [4, 8], [5, 7
[[1, 3], [1, 6], [2, 3], [2, 5], [4, 6], [4, 7], [4, 8
[[1, 3], [1, 6], [2, 3], [2, 7], [4, 5], [4, 6], [4, 8
[[1, 3], [1, 6], [2, 4], [2, 5], [3, 4], [4, 6], [7, 8
[[1, 3], [1, 6], [2, 4], [2, 5], [3, 7], [4, 6], [4, 8
[[1, 3], [1, 6], [2, 4], [2, 5], [3, 8], [4, 6], [4, 7
[[1, 3], [1, 6], [2, 4], [2, 7], [3, 4], [4, 6], [5, 8
[[1, 3], [1, 6], [2, 4], [2, 7], [3, 5], [4, 6], [4, 8
[[1, 3], [1, 6], [2, 4], [2, 7], [3, 8], [4, 5], [4, 6
[[1, 3], [1, 6], [2, 5], [2, 7], [3, 4], [4, 6], [4, 8
[[1, 3], [1, 7], [2, 3], [2, 4], [4, 5], [4, 6], [6, 8
[[1, 3], [1, 7], [2, 3], [2, 4], [4, 6], [4, 8], [5, 6
[[1, 3], [1, 7], [2, 3], [2, 6], [4, 5], [4, 6], [4, 8
[[1, 3], [1, 7], [2, 4], [2, 5], [3, 4], [4, 6], [6, 8
[[1, 3], [1, 7], [2, 4], [2, 5], [3, 6], [4, 6], [4, 8
[[1, 3], [1, 7], [2, 4], [2, 6], [3, 4], [4, 6], [5, 8
[[1, 3], [1, 7], [2, 4], [2, 6], [3, 8], [4, 5], [4, 6
[[1, 3], [1, 7], [2, 5], [2, 6], [3, 4], [4, 6], [4, 8
[[1, 3], [1, 8], [2, 3], [2, 4], [4, 5], [4, 6], [6, 7
[[1, 3], [1, 8], [2, 3], [2, 4], [4, 6], [4, 7], [5, 6
[[1, 3], [1, 8], [2, 3], [2, 6], [4, 5], [4, 6], [4, 7
[[1, 3], [1, 8], [2, 4], [2, 5], [3, 4], [4, 6], [6, 7
[[1, 3], [1, 8], [2, 4], [2, 5], [3, 6], [4, 6], [4, 7
[[1, 3], [1, 8], [2, 4], [2, 6], [3, 4], [4, 6], [5, 7
[[1, 3], [1, 8], [2, 4], [2, 6], [3, 5], [4, 6], [4, 7
[[1, 3], [1, 8], [2, 4], [2, 6], [3, 7], [4, 5], [4, 6
[[1, 3], [1, 8], [2, 4], [2, 7], [3, 4], [4, 6], [5, 6
[[1, 3], [1, 8], [2, 4], [2, 7], [3, 6], [4, 5], [4, 6
[[1, 3], [1, 8], [2, 5], [2, 6], [3, 4], [4, 6], [4, 7
[[1, 3], [1, 8], [2, 6], [2, 7], [3, 4], [4, 5], [4, 6
[[1, 4], [1, 5], [2, 3], [2, 4], [3, 6], [4, 6], [7, 8
[[1, 4], [1, 5], [2, 3], [2, 4], [3, 7], [4, 6], [6, 8
[[1, 4], [1, 5], [2, 3], [2, 4], [3, 8], [4, 6], [6, 7
[[1, 4], [1, 5], [2, 3], [2, 6], [3, 4], [4, 6], [7, 8
[[1, 4], [1, 5], [2, 3], [2, 6], [3, 7], [4, 6], [4, 8
[[1, 4], [1, 5], [2, 3], [2, 6], [3, 8], [4, 6], [4, 7
[[1, 4], [1, 5], [2, 3], [2, 7], [3, 4], [4, 6], [6, 8
[[1, 4], [1, 5], [2, 3], [2, 7], [3, 6], [4, 6], [4, 8
[[1, 4], [1, 5], [2, 4], [2, 6], [3, 7], [3, 8], [4, 6
[[1, 4], [1, 5], [2, 4], [2, 7], [3, 6], [3, 8], [4, 6
[[1, 4], [1, 5], [2, 6], [2, 7], [3, 4], [3, 8], [4, 6
[[1, 4], [1, 6], [2, 3], [2, 4], [3, 5], [4, 6], [7, 8
[[1, 4], [1, 6], [2, 3], [2, 4], [3, 7], [4, 6], [5, 8
[[1, 4], [1, 6], [2, 3], [2, 4], [3, 8], [4, 6], [5, 7
[[1, 4], [1, 6], [2, 3], [2, 5], [3, 4], [4, 6], [7, 8
[[1, 4], [1, 6], [2, 3], [2, 5], [3, 7], [4, 6], [4, 8
[[1, 4], [1, 6], [2, 3], [2, 5], [3, 8], [4, 6], [4, 7
[[1, 4], [1, 6], [2, 3], [2, 7], [3, 4], [4, 6], [5, 8
[[1, 4], [1, 6], [2, 3], [2, 7], [3, 5], [4, 6], [4, 8
[[1, 4], [1, 6], [2, 3], [2, 7], [3, 8], [4, 5], [4, 6
[[1, 4], [1, 6], [2, 4], [2, 5], [3, 7], [3, 8], [4, 6
[[1, 4], [1, 6], [2, 4], [2, 7], [3, 5], [3, 8], [4, 6
[[1, 4], [1, 6], [2, 5], [2, 7], [3, 4], [3, 8], [4, 6
[[1, 4], [1, 7], [2, 3], [2, 4], [3, 5], [4, 6], [6, 8
[[1, 4], [1, 7], [2, 3], [2, 4], [3, 6], [4, 6], [5, 8
[[1, 4], [1, 7], [2, 3], [2, 4], [3, 8], [4, 6], [5, 6
[[1, 4], [1, 7], [2, 3], [2, 5], [3, 4], [4, 6], [6, 8
[[1, 4], [1, 7], [2, 3], [2, 5], [3, 6], [4, 6], [4, 8
[[1, 4], [1, 7], [2, 3], [2, 6], [3, 4], [4, 6], [5, 8
[[1, 4], [1, 7], [2, 3], [2, 6], [3, 5], [4, 6], [4, 8
[[1, 4], [1, 7], [2, 3], [2, 6], [3, 8], [4, 5], [4, 6
[[1, 4], [1, 7], [2, 4], [2, 5], [3, 6], [3, 8], [4, 6
[[1, 4], [1, 7], [2, 4], [2, 6], [3, 5], [3, 8], [4, 6
[[1, 4], [1, 7], [2, 5], [2, 6], [3, 4], [3, 8], [4, 6
[[1, 4], [1, 8], [2, 3], [2, 4], [3, 5], [4, 6], [6, 7
[[1, 4], [1, 8], [2, 3], [2, 4], [3, 6], [4, 6], [5, 7
[[1, 4], [1, 8], [2, 3], [2, 4], [3, 7], [4, 6], [5, 6
[[1, 4], [1, 8], [2, 3], [2, 5], [3, 4], [4, 6], [6, 7
[[1, 4], [1, 8], [2, 3], [2, 5], [3, 6], [4, 6], [4, 7

[[1, 4], [1, 8], [2, 3], [2, 6], [3, 4], [4, 6], [5, 7
[[1, 4], [1, 8], [2, 3], [2, 6], [3, 5], [4, 6], [4, 7
[[1, 4], [1, 8], [2, 3], [2, 6], [3, 7], [4, 5], [4, 6
[[1, 4], [1, 8], [2, 3], [2, 7], [3, 4], [4, 6], [5, 6
[[1, 4], [1, 8], [2, 3], [2, 7], [3, 6], [4, 5], [4, 6
[[1, 4], [1, 8], [2, 4], [2, 5], [3, 6], [3, 7], [4, 6
[[1, 4], [1, 8], [2, 4], [2, 7], [3, 5], [3, 6], [4, 6
[[1, 4], [1, 8], [2, 5], [2, 6], [3, 4], [3, 7], [4, 6
[[1, 4], [1, 8], [2, 5], [2, 7], [3, 4], [3, 6], [4, 6
[[1, 4], [1, 8], [2, 6], [2, 7], [3, 4], [3, 5], [4, 6
[[1, 5], [1, 6], [2, 3], [2, 4], [3, 4], [4, 6], [7, 8
[[1, 5], [1, 6], [2, 3], [2, 4], [3, 7], [4, 6], [4, 8
[[1, 5], [1, 6], [2, 3], [2, 4], [3, 8], [4, 6], [4, 7
[[1, 5], [1, 6], [2, 3], [2, 7], [3, 4], [4, 6], [4, 8
[[1, 5], [1, 6], [2, 4], [2, 7], [3, 4], [3, 8], [4, 6
[[1, 5], [1, 7], [2, 3], [2, 4], [3, 4], [4, 6], [6, 8
[[1, 5], [1, 7], [2, 3], [2, 4], [3, 6], [4, 6], [4, 8
[[1, 5], [1, 7], [2, 3], [2, 6], [3, 4], [4, 6], [4, 8
[[1, 5], [1, 7], [2, 4], [2, 6], [3, 4], [3, 8], [4, 6
[[1, 5], [1, 8], [2, 3], [2, 4], [3, 4], [4, 6], [6, 7
[[1, 5], [1, 8], [2, 3], [2, 4], [3, 6], [4, 6], [4, 7
[[1, 5], [1, 8], [2, 3], [2, 6], [3, 4], [4, 6], [4, 7
[[1, 5], [1, 8], [2, 4], [2, 6], [3, 4], [3, 7], [4, 6
[[1, 5], [1, 8], [2, 4], [2, 7], [3, 4], [3, 6], [4, 6
[[1, 6], [1, 7], [2, 3], [2, 4], [3, 4], [4, 6], [5, 8
[[1, 6], [1, 7], [2, 3], [2, 4], [3, 5], [4, 6], [4, 8
[[1, 6], [1, 7], [2, 3], [2, 4], [3, 8], [4, 5], [4, 6
[[1, 6], [1, 7], [2, 3], [2, 5], [3, 4], [4, 6], [4, 8
[[1, 6], [1, 7], [2, 4], [2, 5], [3, 4], [3, 8], [4, 6
[[1, 6], [1, 8], [2, 3], [2, 4], [3, 4], [4, 6], [5, 7
[[1, 6], [1, 8], [2, 3], [2, 4], [3, 5], [4, 6], [4, 7
[[1, 6], [1, 8], [2, 3], [2, 4], [3, 7], [4, 5], [4, 6
[[1, 6], [1, 8], [2, 3], [2, 5], [3, 4], [4, 6], [4, 7
[[1, 6], [1, 8], [2, 3], [2, 7], [3, 4], [4, 5], [4, 6
[[1, 6], [1, 8], [2, 4], [2, 5], [3, 4], [3, 7], [4, 6
[[1, 6], [1, 8], [2, 4], [2, 7], [3, 4], [3, 5], [4, 6
[[1, 7], [1, 8], [2, 3], [2, 4], [3, 4], [4, 6], [5, 6
[[1, 7], [1, 8], [2, 3], [2, 4], [3, 6], [4, 5], [4, 6
[[1, 7], [1, 8], [2, 3], [2, 6], [3, 4], [4, 5], [4, 6
[[1, 7], [1, 8], [2, 4], [2, 5], [3, 4], [3, 6], [4, 6
[[1, 7], [1, 8], [2, 4], [2, 6], [3, 4], [3, 5], [4, 6

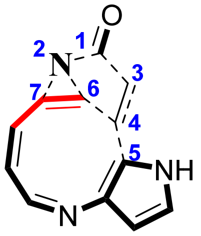

B-x22

[[1, 2], [1, 3], [2, 4], [2, 6], [3, 4], [4, 6], [5, 7
[[1, 2], [1, 3], [2, 4], [2, 6], [3, 4], [4, 7], [5, 6
[[1, 2], [1, 3], [2, 4], [2, 7], [3, 4], [4, 6], [5, 6
[[1, 2], [1, 3], [2, 5], [2, 6], [3, 4], [4, 6], [4, 7
[[1, 2], [1, 3], [2, 6], [2, 7], [3, 4], [4, 5], [4, 6
[[1, 2], [1, 4], [2, 3], [2, 6], [3, 4], [4, 6], [5, 7
[[1, 2], [1, 4], [2, 3], [2, 6], [3, 4], [4, 7], [5, 6
[[1, 2], [1, 4], [2, 3], [2, 7], [3, 4], [4, 6], [5, 6
[[1, 2], [1, 4], [2, 4], [2, 6], [3, 4], [3, 6], [5, 7
[[1, 2], [1, 4], [2, 4], [2, 6], [3, 4], [3, 7], [5, 6
[[1, 2], [1, 4], [2, 4], [2, 7], [3, 4], [3, 6], [5, 6
[[1, 2], [1, 4], [2, 5], [2, 6], [3, 4], [3, 6], [4, 7
[[1, 2], [1, 4], [2, 5], [2, 6], [3, 4], [3, 7], [4, 6
[[1, 2], [1, 4], [2, 5], [2, 7], [3, 4], [3, 6], [4, 6
[[1, 2], [1, 4], [2, 6], [2, 7], [3, 4], [3, 5], [4, 6
[[1, 2], [1, 4], [2, 6], [2, 7], [3, 4], [3, 6], [4, 5
[[1, 2], [1, 5], [2, 3], [2, 6], [3, 4], [4, 6], [4, 7
[[1, 2], [1, 5], [2, 4], [2, 6], [3, 4], [3, 6], [4, 7
[[1, 2], [1, 5], [2, 4], [2, 6], [3, 4], [3, 7], [4, 6
[[1, 2], [1, 5], [2, 4], [2, 7], [3, 4], [3, 6], [4, 6
[[1, 2], [1, 6], [2, 3], [2, 4], [3, 4], [4, 6], [5, 7
[[1, 2], [1, 6], [2, 3], [2, 4], [3, 4], [4, 7], [5, 6

[[1, 2], [1, 6], [2, 3], [2, 5], [3, 4], [4, 6], [4, 7
[[1, 2], [1, 6], [2, 3], [2, 6], [3, 4], [4, 5], [4, 7
[[1, 2], [1, 6], [2, 3], [2, 7], [3, 4], [4, 5], [4, 6
[[1, 2], [1, 6], [2, 4], [2, 5], [3, 4], [3, 6], [4, 7
[[1, 2], [1, 6], [2, 4], [2, 5], [3, 4], [3, 7], [4, 6
[[1, 2], [1, 6], [2, 4], [2, 6], [3, 4], [3, 5], [4, 7
[[1, 2], [1, 6], [2, 4], [2, 6], [3, 4], [3, 7], [4, 5
[[1, 2], [1, 6], [2, 4], [2, 7], [3, 4], [3, 5], [4, 6
[[1, 2], [1, 6], [2, 4], [2, 7], [3, 4], [3, 6], [4, 5
[[1, 2], [1, 7], [2, 3], [2, 4], [3, 4], [4, 6], [5, 6
[[1, 2], [1, 7], [2, 3], [2, 6], [3, 4], [4, 5], [4, 6
[[1, 2], [1, 7], [2, 4], [2, 5], [3, 4], [3, 6], [4, 6
[[1, 2], [1, 7], [2, 4], [2, 6], [3, 4], [3, 5], [4, 6
[[1, 2], [1, 7], [2, 4], [2, 6], [3, 4], [3, 6], [4, 5
[[1, 3], [1, 4], [2, 4], [2, 6], [2, 7], [3, 4], [5, 6
[[1, 3], [1, 4], [2, 5], [2, 6], [2, 7], [3, 4], [4, 6
[[1, 3], [1, 5], [2, 4], [2, 6], [2, 7], [3, 4], [4, 6
[[1, 3], [1, 6], [2, 4], [2, 5], [2, 6], [3, 4], [4, 7
[[1, 3], [1, 6], [2, 4], [2, 5], [2, 7], [3, 4], [4, 6
[[1, 3], [1, 6], [2, 4], [2, 6], [2, 7], [3, 4], [4, 5
[[1, 3], [1, 7], [2, 4], [2, 5], [2, 6], [3, 4], [4, 6
[[1, 4], [1, 5], [2, 3], [2, 6], [2, 7], [3, 4], [4, 6
[[1, 4], [1, 5], [2, 4], [2, 6], [2, 7], [3, 4], [3, 6
[[1, 4], [1, 6], [2, 3], [2, 4], [2, 6], [3, 4], [5, 7
[[1, 4], [1, 6], [2, 3], [2, 4], [2, 7], [3, 4], [5, 6
[[1, 4], [1, 6], [2, 3], [2, 5], [2, 6], [3, 4], [4, 7
[[1, 4], [1, 6], [2, 3], [2, 5], [2, 7], [3, 4], [4, 6
[[1, 4], [1, 6], [2, 3], [2, 6], [2, 7], [3, 4], [4, 5
[[1, 4], [1, 6], [2, 4], [2, 5], [2, 6], [3, 4], [3, 7
[[1, 4], [1, 6], [2, 4], [2, 5], [2, 7], [3, 4], [3, 6
[[1, 4], [1, 6], [2, 4], [2, 6], [2, 7], [3, 4], [3, 5
[[1, 4], [1, 7], [2, 3], [2, 4], [2, 6], [3, 4], [5, 6
[[1, 4], [1, 7], [2, 3], [2, 5], [2, 6], [3, 4], [4, 6
[[1, 4], [1, 7], [2, 4], [2, 5], [2, 6], [3, 4], [3, 6
[[1, 5], [1, 6], [2, 3], [2, 4], [2, 6], [3, 4], [4, 7
[[1, 5], [1, 6], [2, 3], [2, 4], [2, 7], [3, 4], [4, 6
[[1, 5], [1, 7], [2, 3], [2, 4], [2, 6], [3, 4], [4, 6
[[1, 6], [1, 7], [2, 3], [2, 4], [2, 5], [3, 4], [4, 6
[[1, 6], [1, 7], [2, 3], [2, 4], [2, 6], [3, 4], [4, 5]]]

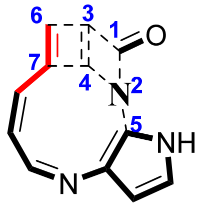

B-x23

[[1, 2], [1, 3], [2, 3], [2, 4], [3, 4], [4, 6], [5, 7
[[1, 2], [1, 3], [2, 3], [2, 4], [3, 4], [4, 7], [5, 6
[[1, 2], [1, 3], [2, 3], [2, 5], [3, 4], [4, 6], [4, 7
[[1, 2], [1, 3], [2, 3], [2, 6], [3, 4], [4, 5], [4, 7
[[1, 2], [1, 3], [2, 3], [2, 7], [3, 4], [4, 5], [4, 6
[[1, 2], [1, 3], [2, 4], [2, 5], [3, 4], [3, 6], [4, 7
[[1, 2], [1, 3], [2, 4], [2, 5], [3, 4], [3, 7], [4, 6
[[1, 2], [1, 3], [2, 4], [2, 6], [3, 4], [3, 5], [4, 7
[[1, 2], [1, 3], [2, 4], [2, 6], [3, 4], [3, 7], [4, 5
[[1, 2], [1, 3], [2, 4], [2, 7], [3, 4], [3, 5], [4, 6
[[1, 2], [1, 3], [2, 4], [2, 7], [3, 4], [3, 6], [4, 5
[[1, 2], [1, 4], [2, 3], [2, 4], [3, 4], [3, 6], [5, 7
[[1, 2], [1, 4], [2, 3], [2, 4], [3, 4], [3, 7], [5, 6
[[1, 2], [1, 4], [2, 3], [2, 5], [3, 4], [3, 6], [4, 7
[[1, 2], [1, 4], [2, 3], [2, 5], [3, 4], [3, 7], [4, 6
[[1, 2], [1, 4], [2, 3], [2, 6], [3, 4], [3, 5], [4, 7
[[1, 2], [1, 4], [2, 3], [2, 6], [3, 4], [3, 7], [4, 5
[[1, 2], [1, 4], [2, 3], [2, 7], [3, 4], [3, 5], [4, 6
[[1, 2], [1, 4], [2, 3], [2, 7], [3, 4], [3, 6], [4, 5
[[1, 2], [1, 4], [2, 4], [2, 5], [3, 4], [3, 6], [3, 7
[[1, 2], [1, 4], [2, 4], [2, 6], [3, 4], [3, 5], [3, 7
[[1, 2], [1, 4], [2, 4], [2, 7], [3, 4], [3, 5], [3, 6
[[1, 2], [1, 5], [2, 3], [2, 4], [3, 4], [3, 6], [4, 7
[[1, 2], [1, 5], [2, 3], [2, 4], [3, 4], [3, 7], [4, 6
[[1, 2], [1, 6], [2, 3], [2, 4], [3, 4], [3, 5], [4, 7

[[1, 2], [1, 6], [2, 3], [2, 4], [3, 4], [3, 7], [4, 5
[[1, 2], [1, 7], [2, 3], [2, 4], [3, 4], [3, 5], [4, 6
[[1, 2], [1, 7], [2, 3], [2, 4], [3, 4], [3, 6], [4, 5
[[1, 3], [1, 4], [2, 3], [2, 4], [2, 6], [3, 4], [5, 7
[[1, 3], [1, 4], [2, 3], [2, 4], [2, 7], [3, 4], [5, 6
[[1, 3], [1, 4], [2, 3], [2, 5], [2, 6], [3, 4], [4, 7
[[1, 3], [1, 4], [2, 3], [2, 5], [2, 7], [3, 4], [4, 6
[[1, 3], [1, 4], [2, 3], [2, 6], [2, 7], [3, 4], [4, 5
[[1, 3], [1, 4], [2, 4], [2, 5], [2, 6], [3, 4], [3, 7
[[1, 3], [1, 4], [2, 4], [2, 5], [2, 7], [3, 4], [3, 6
[[1, 3], [1, 4], [2, 4], [2, 6], [2, 7], [3, 4], [3, 5
[[1, 3], [1, 5], [2, 3], [2, 4], [2, 6], [3, 4], [4, 7
[[1, 3], [1, 5], [2, 3], [2, 4], [2, 7], [3, 4], [4, 6
[[1, 3], [1, 6], [2, 3], [2, 4], [2, 5], [3, 4], [4, 7
[[1, 3], [1, 6], [2, 3], [2, 4], [2, 7], [3, 4], [4, 5
[[1, 3], [1, 7], [2, 3], [2, 4], [2, 5], [3, 4], [4, 6
[[1, 3], [1, 7], [2, 3], [2, 4], [2, 6], [3, 4], [4, 5
[[1, 4], [1, 5], [2, 3], [2, 4], [2, 6], [3, 4], [3, 7
[[1, 4], [1, 5], [2, 3], [2, 4], [2, 7], [3, 4], [3, 6
[[1, 4], [1, 6], [2, 3], [2, 4], [2, 5], [3, 4], [3, 7
[[1, 4], [1, 6], [2, 3], [2, 4], [2, 7], [3, 4], [3, 5
[[1, 4], [1, 7], [2, 3], [2, 4], [2, 5], [3, 4], [3, 6
[[1, 4], [1, 7], [2, 3], [2, 4], [2, 6], [3, 4], [3, 5]]]

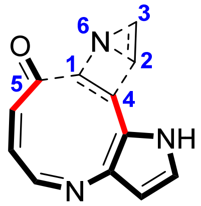

B-i1

[[1, 2], [1, 3], [1, 6], [2, 4], [2, 6], [3, 4], [5, 6
[[1, 2], [1, 3], [1, 6], [2, 5], [2, 6], [3, 4], [4, 6
[[1, 2], [1, 4], [1, 6], [2, 3], [2, 6], [3, 4], [5, 6
[[1, 2], [1, 4], [1, 6], [2, 5], [2, 6], [3, 4], [3, 6
[[1, 2], [1, 5], [1, 6], [2, 3], [2, 6], [3, 4], [4, 6
[[1, 2], [1, 5], [1, 6], [2, 4], [2, 6], [3, 4], [3, 6
[[1, 3], [1, 4], [1, 6], [2, 3], [2, 4], [2, 6], [5, 6
[[1, 3], [1, 4], [1, 6], [2, 4], [2, 5], [2, 6], [3, 6
[[1, 3], [1, 5], [1, 6], [2, 3], [2, 4], [2, 6], [4, 6
[[1, 4], [1, 5], [1, 6], [2, 3], [2, 4], [2, 6], [3, 6]]]

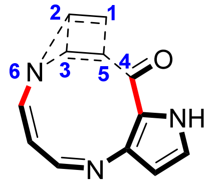

B-k1

[[1, 2], [1, 3], [2, 3], [2, 5], [3, 5], [4, 6], [5, 6
[[1, 2], [1, 3], [2, 3], [2, 6], [3, 5], [4, 5], [5, 6
[[1, 2], [1, 3], [2, 4], [2, 5], [3, 5], [3, 6], [5, 6
[[1, 2], [1, 3], [2, 5], [2, 6], [3, 4], [3, 5], [5, 6
[[1, 2], [1, 3], [2, 5], [2, 6], [3, 5], [3, 6], [4, 5
[[1, 2], [1, 4], [2, 3], [2, 5], [3, 5], [3, 6], [5, 6
[[1, 2], [1, 5], [2, 3], [2, 4], [3, 5], [3, 6], [5, 6
[[1, 2], [1, 5], [2, 3], [2, 5], [3, 5], [3, 6], [4, 6
[[1, 2], [1, 5], [2, 3], [2, 6], [3, 4], [3, 5], [5, 6
[[1, 2], [1, 5], [2, 3], [2, 6], [3, 5], [3, 6], [4, 5
[[1, 2], [1, 5], [2, 5], [2, 6], [3, 4], [3, 5], [3, 6
[[1, 2], [1, 6], [2, 3], [2, 5], [3, 4], [3, 5], [5, 6
[[1, 2], [1, 6], [2, 3], [2, 5], [3, 5], [3, 6], [4, 5
[[1, 3], [1, 4], [2, 3], [2, 5], [2, 6], [3, 5], [5, 6
[[1, 3], [1, 5], [2, 3], [2, 4], [2, 5], [3, 6], [5, 6
[[1, 3], [1, 5], [2, 3], [2, 5], [2, 6], [3, 4], [5, 6
[[1, 3], [1, 5], [2, 3], [2, 5], [2, 6], [3, 5], [4, 6
[[1, 3], [1, 5], [2, 3], [2, 5], [2, 6], [3, 6], [4, 5
[[1, 3], [1, 5], [2, 4], [2, 5], [2, 6], [3, 5], [3, 6

[[1, 3], [1, 6], [2, 3], [2, 4], [2, 5], [3, 5], [5, 6
[[1, 3], [1, 6], [2, 3], [2, 5], [2, 6], [3, 5], [4, 5
[[1, 5], [1, 6], [2, 3], [2, 4], [2, 5], [3, 5], [3, 6
[[1, 5], [1, 6], [2, 3], [2, 5], [2, 6], [3, 4], [3, 5]]]

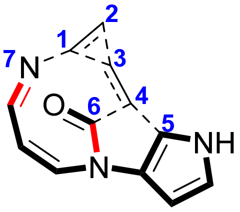

B-l1

[[1, 2], [1, 3], [1, 4], [2, 3], [3, 4], [4, 5], [6, 7
[[1, 2], [1, 3], [1, 4], [2, 3], [3, 4], [4, 6], [5, 7
[[1, 2], [1, 3], [1, 4], [2, 3], [3, 4], [4, 7], [5, 6
[[1, 2], [1, 3], [1, 4], [2, 4], [3, 4], [3, 5], [6, 7
[[1, 2], [1, 3], [1, 4], [2, 4], [3, 4], [3, 6], [5, 7
[[1, 2], [1, 3], [1, 4], [2, 4], [3, 4], [3, 7], [5, 6
[[1, 2], [1, 3], [1, 4], [2, 5], [3, 4], [3, 6], [4, 7
[[1, 2], [1, 3], [1, 4], [2, 5], [3, 4], [3, 7], [4, 6
[[1, 2], [1, 3], [1, 4], [2, 6], [3, 4], [3, 5], [4, 7
[[1, 2], [1, 3], [1, 4], [2, 6], [3, 4], [3, 7], [4, 5
[[1, 2], [1, 3], [1, 4], [2, 7], [3, 4], [3, 5], [4, 6
[[1, 2], [1, 3], [1, 4], [2, 7], [3, 4], [3, 6], [4, 5
[[1, 2], [1, 3], [1, 5], [2, 3], [3, 4], [4, 6], [4, 7
[[1, 2], [1, 3], [1, 5], [2, 4], [3, 4], [3, 6], [4, 7
[[1, 2], [1, 3], [1, 5], [2, 4], [3, 4], [3, 7], [4, 6
[[1, 2], [1, 3], [1, 6], [2, 3], [3, 4], [4, 5], [4, 7
[[1, 2], [1, 3], [1, 6], [2, 4], [3, 4], [3, 5], [4, 7
[[1, 2], [1, 3], [1, 6], [2, 4], [3, 4], [3, 7], [4, 5
[[1, 2], [1, 3], [1, 7], [2, 3], [3, 4], [4, 5], [4, 6
[[1, 2], [1, 3], [1, 7], [2, 4], [3, 4], [3, 5], [4, 6
[[1, 2], [1, 3], [1, 7], [2, 4], [3, 4], [3, 6], [4, 5
[[1, 2], [1, 4], [1, 5], [2, 3], [3, 4], [3, 6], [4, 7
[[1, 2], [1, 4], [1, 5], [2, 3], [3, 4], [3, 7], [4, 6
[[1, 2], [1, 4], [1, 5], [2, 4], [3, 4], [3, 6], [3, 7
[[1, 2], [1, 4], [1, 6], [2, 3], [3, 4], [3, 5], [4, 7
[[1, 2], [1, 4], [1, 6], [2, 3], [3, 4], [3, 7], [4, 5
[[1, 2], [1, 4], [1, 6], [2, 4], [3, 4], [3, 5], [3, 7
[[1, 2], [1, 4], [1, 7], [2, 3], [3, 4], [3, 5], [4, 6
[[1, 2], [1, 4], [1, 7], [2, 3], [3, 4], [3, 6], [4, 5
[[1, 2], [1, 4], [1, 7], [2, 4], [3, 4], [3, 5], [3, 6
[[1, 3], [1, 4], [1, 5], [2, 3], [2, 4], [3, 6], [4, 7
[[1, 3], [1, 4], [1, 5], [2, 3], [2, 4], [3, 7], [4, 6
[[1, 3], [1, 4], [1, 5], [2, 4], [2, 6], [3, 4], [3, 7
[[1, 3], [1, 4], [1, 5], [2, 4], [2, 7], [3, 4], [3, 6
[[1, 3], [1, 4], [1, 6], [2, 3], [2, 4], [3, 4], [5, 7
[[1, 3], [1, 4], [1, 6], [2, 3], [2, 4], [3, 5], [4, 7
[[1, 3], [1, 4], [1, 6], [2, 3], [2, 4], [3, 7], [4, 5
[[1, 3], [1, 4], [1, 6], [2, 4], [2, 5], [3, 4], [3, 7
[[1, 3], [1, 4], [1, 6], [2, 4], [2, 7], [3, 4], [3, 5
[[1, 3], [1, 4], [1, 7], [2, 3], [2, 4], [3, 4], [5, 6
[[1, 3], [1, 4], [1, 7], [2, 3], [2, 4], [3, 5], [4, 6
[[1, 3], [1, 4], [1, 7], [2, 3], [2, 4], [3, 6], [4, 5
[[1, 3], [1, 4], [1, 7], [2, 4], [2, 5], [3, 4], [3, 6
[[1, 3], [1, 4], [1, 7], [2, 4], [2, 6], [3, 4], [3, 5
[[1, 3], [1, 5], [1, 6], [2, 3], [2, 4], [3, 4], [4, 7
[[1, 3], [1, 5], [1, 7], [2, 3], [2, 4], [3, 4], [4, 6
[[1, 3], [1, 6], [1, 7], [2, 3], [2, 4], [3, 4], [4, 5
[[1, 4], [1, 5], [1, 6], [2, 3], [2, 4], [3, 4], [3, 7
[[1, 4], [1, 5], [1, 7], [2, 3], [2, 4], [3, 4], [3, 6
[[1, 4], [1, 6], [1, 7], [2, 3], [2, 4], [3, 4], [3, 5]]]

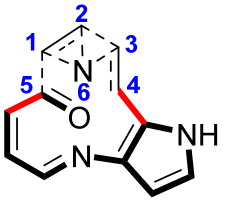

B-j1

[[1, 2], [1, 3], [1, 6], [2, 3], [2, 6], [3, 4], [5, 6
[[1, 2], [1, 3], [1, 6], [2, 5], [2, 6], [3, 4], [3, 6
[[1, 2], [1, 5], [1, 6], [2, 3], [2, 6], [3, 4], [3, 6
[[1, 3], [1, 5], [1, 6], [2, 3], [2, 4], [2, 6], [3, 6]]]

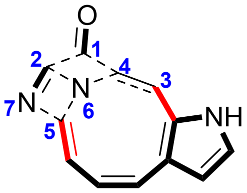

C-p

[[1, 2], [1, 4], [2, 5], [3, 4], [4, 6], [5, 6], [6, 7
[[1, 2], [1, 4], [2, 6], [3, 4], [4, 5], [5, 6], [6, 7
[[1, 2], [1, 4], [2, 6], [3, 4], [4, 6], [5, 6], [5, 7
[[1, 2], [1, 5], [2, 4], [3, 4], [4, 6], [5, 6], [6, 7
[[1, 2], [1, 5], [2, 6], [3, 4], [4, 5], [4, 6], [6, 7
[[1, 2], [1, 5], [2, 6], [3, 4], [4, 6], [4, 7], [5, 6
[[1, 2], [1, 6], [2, 4], [3, 4], [4, 5], [5, 6], [6, 7
[[1, 2], [1, 6], [2, 4], [3, 4], [4, 6], [5, 6], [5, 7
[[1, 2], [1, 6], [2, 5], [3, 4], [4, 5], [4, 6], [6, 7
[[1, 2], [1, 6], [2, 5], [3, 4], [4, 6], [4, 7], [5, 6
[[1, 2], [1, 6], [2, 6], [3, 4], [4, 5], [4, 6], [5, 7
[[1, 2], [1, 6], [2, 6], [3, 4], [4, 5], [4, 7], [5, 6
[[1, 2], [1, 7], [2, 6], [3, 4], [4, 5], [4, 6], [5, 6
[[1, 4], [1, 5], [2, 4], [2, 6], [3, 4], [5, 6], [6, 7
[[1, 4], [1, 5], [2, 5], [2, 6], [3, 4], [4, 6], [6, 7
[[1, 4], [1, 6], [2, 4], [2, 5], [3, 4], [5, 6], [6, 7
[[1, 4], [1, 6], [2, 4], [2, 6], [3, 4], [5, 6], [5, 7
[[1, 4], [1, 6], [2, 5], [2, 6], [3, 4], [4, 5], [6, 7
[[1, 4], [1, 6], [2, 5], [2, 6], [3, 4], [4, 6], [5, 7
[[1, 4], [1, 6], [2, 5], [2, 6], [3, 4], [4, 7], [5, 6
[[1, 4], [1, 7], [2, 5], [2, 6], [3, 4], [4, 6], [5, 6
[[1, 5], [1, 6], [2, 4], [2, 5], [3, 4], [4, 6], [6, 7
[[1, 5], [1, 6], [2, 4], [2, 6], [3, 4], [4, 5], [6, 7
[[1, 5], [1, 6], [2, 4], [2, 6], [3, 4], [4, 6], [5, 7
[[1, 5], [1, 6], [2, 4], [2, 6], [3, 4], [4, 7], [5, 6
[[1, 5], [1, 6], [2, 5], [2, 6], [3, 4], [4, 6], [4, 7
[[1, 5], [1, 7], [2, 4], [2, 6], [3, 4], [4, 6], [5, 6
[[1, 6], [1, 7], [2, 4], [2, 5], [3, 4], [4, 6], [5, 6
[[1, 6], [1, 7], [2, 4], [2, 6], [3, 4], [4, 5], [5, 6
[[1, 6], [1, 7], [2, 5], [2, 6], [3, 4], [4, 5], [4, 6]]]

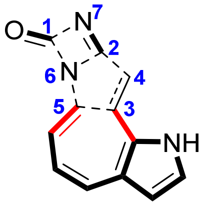

C-q

[[1, 2], [1, 3], [2, 5], [3, 4], [4, 6], [5, 6], [6, 7
[[1, 2], [1, 3], [2, 6], [3, 4], [4, 5], [5, 6], [6, 7
[[1, 2], [1, 3], [2, 6], [3, 4], [4, 6], [5, 6], [5, 7
[[1, 2], [1, 4], [2, 5], [3, 4], [3, 6], [5, 6], [6, 7
[[1, 2], [1, 4], [2, 6], [3, 4], [3, 5], [5, 6], [6, 7
[[1, 2], [1, 4], [2, 6], [3, 4], [3, 6], [5, 6], [5, 7
[[1, 2], [1, 5], [2, 3], [3, 4], [4, 6], [5, 6], [6, 7
[[1, 2], [1, 5], [2, 4], [3, 4], [3, 6], [5, 6], [6, 7

[[1, 2], [1, 5], [2, 5], [3, 4], [3, 6], [4, 6], [6, 7
[[1, 2], [1, 5], [2, 6], [3, 4], [3, 5], [4, 6], [6, 7
[[1, 2], [1, 5], [2, 6], [3, 4], [3, 6], [4, 5], [6, 7
[[1, 2], [1, 5], [2, 6], [3, 4], [3, 6], [4, 6], [5, 7
[[1, 2], [1, 5], [2, 6], [3, 4], [3, 6], [4, 7], [5, 6
[[1, 2], [1, 5], [2, 6], [3, 4], [3, 7], [4, 6], [5, 6
[[1, 2], [1, 6], [2, 3], [3, 4], [4, 5], [5, 6], [6, 7
[[1, 2], [1, 6], [2, 3], [3, 4], [4, 6], [5, 6], [5, 7
[[1, 2], [1, 6], [2, 4], [3, 4], [3, 5], [5, 6], [6, 7
[[1, 2], [1, 6], [2, 4], [3, 4], [3, 6], [5, 6], [5, 7
[[1, 2], [1, 6], [2, 5], [3, 4], [3, 5], [4, 6], [6, 7
[[1, 2], [1, 6], [2, 5], [3, 4], [3, 6], [4, 5], [6, 7
[[1, 2], [1, 6], [2, 5], [3, 4], [3, 6], [4, 6], [5, 7
[[1, 2], [1, 6], [2, 5], [3, 4], [3, 6], [4, 7], [5, 6
[[1, 2], [1, 6], [2, 5], [3, 4], [3, 7], [4, 6], [5, 6
[[1, 2], [1, 6], [2, 6], [3, 4], [3, 5], [4, 6], [5, 7
[[1, 2], [1, 6], [2, 6], [3, 4], [3, 5], [4, 7], [5, 6
[[1, 2], [1, 6], [2, 6], [3, 4], [3, 6], [4, 5], [5, 7
[[1, 2], [1, 6], [2, 6], [3, 4], [3, 7], [4, 5], [5, 6
[[1, 2], [1, 7], [2, 5], [3, 4], [3, 6], [4, 6], [5, 6
[[1, 2], [1, 7], [2, 6], [3, 4], [3, 5], [4, 6], [5, 6
[[1, 2], [1, 7], [2, 6], [3, 4], [3, 6], [4, 5], [5, 6
[[1, 3], [1, 4], [2, 5], [2, 6], [3, 4], [5, 6], [6, 7
[[1, 3], [1, 5], [2, 4], [2, 6], [3, 4], [5, 6], [6, 7
[[1, 3], [1, 5], [2, 5], [2, 6], [3, 4], [4, 6], [6, 7
[[1, 3], [1, 6], [2, 4], [2, 5], [3, 4], [5, 6], [6, 7
[[1, 3], [1, 6], [2, 4], [2, 6], [3, 4], [5, 6], [5, 7
[[1, 3], [1, 6], [2, 5], [2, 6], [3, 4], [4, 5], [6, 7
[[1, 3], [1, 6], [2, 5], [2, 6], [3, 4], [4, 6], [5, 7
[[1, 3], [1, 6], [2, 5], [2, 6], [3, 4], [4, 7], [5, 6
[[1, 3], [1, 7], [2, 5], [2, 6], [3, 4], [4, 6], [5, 6
[[1, 4], [1, 5], [2, 3], [2, 6], [3, 4], [5, 6], [6, 7
[[1, 4], [1, 5], [2, 5], [2, 6], [3, 4], [3, 6], [6, 7
[[1, 4], [1, 6], [2, 3], [2, 5], [3, 4], [5, 6], [6, 7
[[1, 4], [1, 6], [2, 3], [2, 6], [3, 4], [5, 6], [5, 7
[[1, 4], [1, 6], [2, 5], [2, 6], [3, 4], [3, 5], [6, 7
[[1, 4], [1, 6], [2, 5], [2, 6], [3, 4], [3, 6], [5, 7
[[1, 4], [1, 6], [2, 5], [2, 6], [3, 4], [3, 7], [5, 6
[[1, 4], [1, 7], [2, 5], [2, 6], [3, 4], [3, 6], [5, 6
[[1, 5], [1, 6], [2, 3], [2, 4], [3, 4], [5, 6], [6, 7
[[1, 5], [1, 6], [2, 3], [2, 5], [3, 4], [4, 6], [6, 7
[[1, 5], [1, 6], [2, 3], [2, 6], [3, 4], [4, 5], [6, 7
[[1, 5], [1, 6], [2, 3], [2, 6], [3, 4], [4, 6], [5, 7
[[1, 5], [1, 6], [2, 3], [2, 6], [3, 4], [4, 7], [5, 6
[[1, 5], [1, 6], [2, 4], [2, 5], [3, 4], [3, 6], [6, 7
[[1, 5], [1, 6], [2, 4], [2, 6], [3, 4], [3, 5], [6, 7
[[1, 5], [1, 6], [2, 4], [2, 6], [3, 4], [3, 6], [5, 7
[[1, 5], [1, 6], [2, 4], [2, 6], [3, 4], [3, 7], [5, 6
[[1, 5], [1, 6], [2, 5], [2, 6], [3, 4], [3, 6], [4, 7
[[1, 5], [1, 6], [2, 5], [2, 6], [3, 4], [3, 7], [4, 6
[[1, 5], [1, 7], [2, 3], [2, 6], [3, 4], [4, 6], [5, 6
[[1, 5], [1, 7], [2, 4], [2, 6], [3, 4], [3, 6], [5, 6
[[1, 5], [1, 7], [2, 5], [2, 6], [3, 4], [3, 6], [4, 6
[[1, 6], [1, 7], [2, 3], [2, 5], [3, 4], [4, 6], [5, 6
[[1, 6], [1, 7], [2, 3], [2, 6], [3, 4], [4, 5], [5, 6
[[1, 6], [1, 7], [2, 4], [2, 5], [3, 4], [3, 6], [5, 6
[[1, 6], [1, 7], [2, 4], [2, 6], [3, 4], [3, 5], [5, 6
[[1, 6], [1, 7], [2, 5], [2, 6], [3, 4], [3, 5], [4, 6
[[1, 6], [1, 7], [2, 5], [2, 6], [3, 4], [3, 6], [4, 5]]]

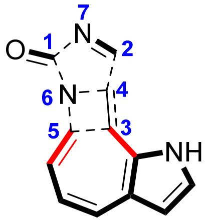

C-x4

[[1, 2], [1, 3], [3, 4], [4, 5], [4, 6], [5, 6], [6, 7
[[1, 2], [1, 4], [3, 4], [3, 5], [4, 6], [5, 6], [6, 7
[[1, 2], [1, 4], [3, 4], [3, 6], [4, 5], [5, 6], [6, 7
[[1, 2], [1, 4], [3, 4], [3, 6], [4, 6], [5, 6], [5, 7
[[1, 2], [1, 5], [3, 4], [3, 6], [4, 5], [4, 6], [6, 7

[[1, 2], [1, 5], [3, 4], [3, 6], [4, 6], [4, 7], [5, 6
[[1, 2], [1, 6], [3, 4], [3, 5], [4, 5], [4, 6], [6, 7
[[1, 2], [1, 6], [3, 4], [3, 5], [4, 6], [4, 7], [5, 6
[[1, 2], [1, 6], [3, 4], [3, 6], [4, 5], [4, 6], [5, 7
[[1, 2], [1, 6], [3, 4], [3, 6], [4, 5], [4, 7], [5, 6
[[1, 2], [1, 6], [3, 4], [3, 7], [4, 5], [4, 6], [5, 6
[[1, 3], [1, 4], [2, 5], [3, 4], [4, 6], [5, 6], [6, 7
[[1, 3], [1, 4], [2, 6], [3, 4], [4, 5], [5, 6], [6, 7
[[1, 3], [1, 4], [2, 6], [3, 4], [4, 6], [5, 6], [5, 7
[[1, 3], [1, 5], [2, 4], [3, 4], [4, 6], [5, 6], [6, 7
[[1, 3], [1, 5], [2, 6], [3, 4], [4, 5], [4, 6], [6, 7
[[1, 3], [1, 5], [2, 6], [3, 4], [4, 6], [4, 7], [5, 6
[[1, 3], [1, 6], [2, 4], [3, 4], [4, 5], [5, 6], [6, 7
[[1, 3], [1, 6], [2, 4], [3, 4], [4, 6], [5, 6], [5, 7
[[1, 3], [1, 6], [2, 5], [3, 4], [4, 5], [4, 6], [6, 7
[[1, 3], [1, 6], [2, 5], [3, 4], [4, 6], [4, 7], [5, 6
[[1, 3], [1, 6], [2, 6], [3, 4], [4, 5], [4, 6], [5, 7
[[1, 3], [1, 6], [2, 6], [3, 4], [4, 5], [4, 7], [5, 6
[[1, 3], [1, 7], [2, 6], [3, 4], [4, 5], [4, 6], [5, 6
[[1, 4], [1, 5], [2, 3], [3, 4], [4, 6], [5, 6], [6, 7
[[1, 4], [1, 5], [2, 4], [3, 4], [3, 6], [5, 6], [6, 7
[[1, 4], [1, 5], [2, 5], [3, 4], [3, 6], [4, 6], [6, 7
[[1, 4], [1, 5], [2, 6], [3, 4], [3, 5], [4, 6], [6, 7
[[1, 4], [1, 5], [2, 6], [3, 4], [3, 6], [4, 5], [6, 7
[[1, 4], [1, 5], [2, 6], [3, 4], [3, 6], [4, 6], [5, 7
[[1, 4], [1, 5], [2, 6], [3, 4], [3, 6], [4, 7], [5, 6
[[1, 4], [1, 5], [2, 6], [3, 4], [3, 7], [4, 6], [5, 6
[[1, 4], [1, 6], [2, 3], [3, 4], [4, 5], [5, 6], [6, 7
[[1, 4], [1, 6], [2, 3], [3, 4], [4, 6], [5, 6], [5, 7
[[1, 4], [1, 6], [2, 4], [3, 4], [3, 5], [5, 6], [6, 7
[[1, 4], [1, 6], [2, 4], [3, 4], [3, 6], [5, 6], [5, 7
[[1, 4], [1, 6], [2, 5], [3, 4], [3, 5], [4, 6], [6, 7
[[1, 4], [1, 6], [2, 5], [3, 4], [3, 6], [4, 5], [6, 7
[[1, 4], [1, 6], [2, 5], [3, 4], [3, 6], [4, 6], [5, 7
[[1, 4], [1, 6], [2, 5], [3, 4], [3, 6], [4, 7], [5, 6
[[1, 4], [1, 6], [2, 5], [3, 4], [3, 7], [4, 6], [5, 6
[[1, 4], [1, 6], [2, 6], [3, 4], [3, 5], [4, 5], [6, 7
[[1, 4], [1, 6], [2, 6], [3, 4], [3, 5], [4, 6], [5, 7
[[1, 4], [1, 6], [2, 6], [3, 4], [3, 5], [4, 7], [5, 6
[[1, 4], [1, 6], [2, 6], [3, 4], [3, 6], [4, 5], [5, 7
[[1, 4], [1, 6], [2, 6], [3, 4], [3, 7], [4, 5], [5, 6
[[1, 4], [1, 7], [2, 5], [3, 4], [3, 6], [4, 6], [5, 6
[[1, 4], [1, 7], [2, 6], [3, 4], [3, 5], [4, 6], [5, 6
[[1, 4], [1, 7], [2, 6], [3, 4], [3, 6], [4, 5], [5, 6
[[1, 5], [1, 6], [2, 3], [3, 4], [4, 5], [4, 6], [6, 7
[[1, 5], [1, 6], [2, 3], [3, 4], [4, 6], [4, 7], [5, 6
[[1, 5], [1, 6], [2, 4], [3, 4], [3, 5], [4, 6], [6, 7
[[1, 5], [1, 6], [2, 4], [3, 4], [3, 6], [4, 5], [6, 7
[[1, 5], [1, 6], [2, 4], [3, 4], [3, 6], [4, 6], [5, 7
[[1, 5], [1, 6], [2, 4], [3, 4], [3, 6], [4, 7], [5, 6
[[1, 5], [1, 6], [2, 4], [3, 4], [3, 7], [4, 6], [5, 6
[[1, 5], [1, 6], [2, 5], [3, 4], [3, 6], [4, 6], [4, 7
[[1, 5], [1, 6], [2, 6], [3, 4], [3, 5], [4, 6], [4, 7
[[1, 5], [1, 6], [2, 6], [3, 4], [3, 6], [4, 5], [4, 7
[[1, 5], [1, 6], [2, 6], [3, 4], [3, 7], [4, 5], [4, 6
[[1, 5], [1, 7], [2, 4], [3, 4], [3, 6], [4, 6], [5, 6
[[1, 5], [1, 7], [2, 6], [3, 4], [3, 6], [4, 5], [4, 6
[[1, 6], [1, 7], [2, 3], [3, 4], [4, 5], [4, 6], [5, 6
[[1, 6], [1, 7], [2, 4], [3, 4], [3, 5], [4, 6], [5, 6
[[1, 6], [1, 7], [2, 4], [3, 4], [3, 6], [4, 5], [5, 6
[[1, 6], [1, 7], [2, 5], [3, 4], [3, 6], [4, 5], [4, 6
[[1, 6], [1, 7], [2, 6], [3, 4], [3, 5], [4, 5], [4, 6]]]

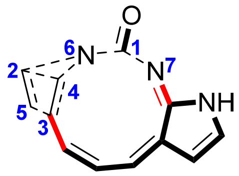

C-r

[[1, 2], [1, 3], [2, 4], [3, 4], [4, 6], [5, 6], [6, 7
[[1, 2], [1, 3], [2, 6], [3, 4], [4, 5], [4, 6], [6, 7
[[1, 2], [1, 3], [2, 6], [3, 4], [4, 6], [4, 7], [5, 6
[[1, 2], [1, 4], [2, 3], [3, 4], [4, 6], [5, 6], [6, 7

[[1, 2], [1, 4], [2, 4], [3, 4], [3, 6], [5, 6], [6, 7
[[1, 2], [1, 4], [2, 6], [3, 4], [3, 5], [4, 6], [6, 7
[[1, 2], [1, 4], [2, 6], [3, 4], [3, 6], [4, 5], [6, 7
[[1, 2], [1, 4], [2, 6], [3, 4], [3, 6], [4, 6], [5, 7
[[1, 2], [1, 4], [2, 6], [3, 4], [3, 6], [4, 7], [5, 6
[[1, 2], [1, 4], [2, 6], [3, 4], [3, 7], [4, 6], [5, 6
[[1, 2], [1, 4], [2, 7], [3, 4], [3, 6], [4, 6], [5, 6
[[1, 2], [1, 5], [2, 4], [3, 4], [3, 6], [4, 6], [6, 7
[[1, 2], [1, 5], [2, 6], [3, 4], [3, 6], [4, 6], [4, 7
[[1, 2], [1, 6], [2, 3], [3, 4], [4, 5], [4, 6], [6, 7
[[1, 2], [1, 6], [2, 3], [3, 4], [4, 6], [4, 7], [5, 6
[[1, 2], [1, 6], [2, 4], [3, 4], [3, 5], [4, 6], [6, 7
[[1, 2], [1, 6], [2, 4], [3, 4], [3, 6], [4, 5], [6, 7
[[1, 2], [1, 6], [2, 4], [3, 4], [3, 6], [4, 6], [5, 7
[[1, 2], [1, 6], [2, 4], [3, 4], [3, 6], [4, 7], [5, 6
[[1, 2], [1, 6], [2, 4], [3, 4], [3, 7], [4, 6], [5, 6
[[1, 2], [1, 6], [2, 6], [3, 4], [3, 5], [4, 6], [4, 7
[[1, 2], [1, 6], [2, 6], [3, 4], [3, 6], [4, 5], [4, 7
[[1, 2], [1, 6], [2, 6], [3, 4], [3, 7], [4, 5], [4, 6
[[1, 2], [1, 6], [2, 7], [3, 4], [3, 6], [4, 5], [4, 6
[[1, 2], [1, 7], [2, 4], [3, 4], [3, 6], [4, 6], [5, 6
[[1, 2], [1, 7], [2, 6], [3, 4], [3, 6], [4, 5], [4, 6
[[1, 3], [1, 4], [2, 4], [2, 6], [3, 4], [5, 6], [6, 7
[[1, 3], [1, 4], [2, 6], [2, 7], [3, 4], [4, 6], [5, 6
[[1, 3], [1, 5], [2, 4], [2, 6], [3, 4], [4, 6], [6, 7
[[1, 3], [1, 6], [2, 4], [2, 6], [3, 4], [4, 5], [6, 7
[[1, 3], [1, 6], [2, 4], [2, 6], [3, 4], [4, 6], [5, 7
[[1, 3], [1, 6], [2, 4], [2, 6], [3, 4], [4, 7], [5, 6
[[1, 3], [1, 6], [2, 4], [2, 7], [3, 4], [4, 6], [5, 6
[[1, 3], [1, 6], [2, 6], [2, 7], [3, 4], [4, 5], [4, 6
[[1, 3], [1, 7], [2, 4], [2, 6], [3, 4], [4, 6], [5, 6
[[1, 4], [1, 5], [2, 3], [2, 6], [3, 4], [4, 6], [6, 7
[[1, 4], [1, 5], [2, 4], [2, 6], [3, 4], [3, 6], [6, 7
[[1, 4], [1, 5], [2, 6], [2, 7], [3, 4], [3, 6], [4, 6
[[1, 4], [1, 6], [2, 3], [2, 4], [3, 4], [5, 6], [6, 7
[[1, 4], [1, 6], [2, 3], [2, 6], [3, 4], [4, 5], [6, 7
[[1, 4], [1, 6], [2, 3], [2, 6], [3, 4], [4, 6], [5, 7
[[1, 4], [1, 6], [2, 3], [2, 6], [3, 4], [4, 7], [5, 6
[[1, 4], [1, 6], [2, 3], [2, 7], [3, 4], [4, 6], [5, 6
[[1, 4], [1, 6], [2, 4], [2, 6], [3, 4], [3, 5], [6, 7
[[1, 4], [1, 6], [2, 4], [2, 6], [3, 4], [3, 6], [5, 7
[[1, 4], [1, 6], [2, 4], [2, 6], [3, 4], [3, 7], [5, 6
[[1, 4], [1, 6], [2, 4], [2, 7], [3, 4], [3, 6], [5, 6
[[1, 4], [1, 6], [2, 6], [2, 7], [3, 4], [3, 5], [4, 6
[[1, 4], [1, 6], [2, 6], [2, 7], [3, 4], [3, 6], [4, 5
[[1, 4], [1, 7], [2, 3], [2, 6], [3, 4], [4, 6], [5, 6
[[1, 4], [1, 7], [2, 4], [2, 6], [3, 4], [3, 6], [5, 6
[[1, 5], [1, 6], [2, 3], [2, 4], [3, 4], [4, 6], [6, 7
[[1, 5], [1, 6], [2, 3], [2, 6], [3, 4], [4, 6], [4, 7
[[1, 5], [1, 6], [2, 4], [2, 6], [3, 4], [3, 6], [4, 7
[[1, 5], [1, 6], [2, 4], [2, 6], [3, 4], [3, 7], [4, 6
[[1, 5], [1, 6], [2, 4], [2, 7], [3, 4], [3, 6], [4, 6
[[1, 5], [1, 7], [2, 4], [2, 6], [3, 4], [3, 6], [4, 6
[[1, 6], [1, 7], [2, 3], [2, 4], [3, 4], [4, 6], [5, 6
[[1, 6], [1, 7], [2, 3], [2, 6], [3, 4], [4, 5], [4, 6
[[1, 6], [1, 7], [2, 4], [2, 6], [3, 4], [3, 5], [4, 6
[[1, 6], [1, 7], [2, 4], [2, 6], [3, 4], [3, 6], [4, 5]]]

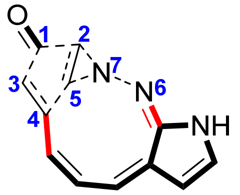

C-x5

[[1, 2], [1, 3], [2, 6], [3, 4], [4, 5], [5, 6], [6, 7
[[1, 2], [1, 3], [2, 6], [3, 4], [4, 6], [5, 6], [5, 7
[[1, 2], [1, 4], [2, 6], [3, 4], [3, 5], [5, 6], [6, 7
[[1, 2], [1, 4], [2, 6], [3, 4], [3, 6], [5, 6], [5, 7
[[1, 2], [1, 5], [2, 3], [3, 4], [4, 6], [5, 6], [6, 7
[[1, 2], [1, 5], [2, 4], [3, 4], [3, 6], [5, 6], [6, 7
[[1, 2], [1, 5], [2, 6], [3, 4], [3, 5], [4, 6], [6, 7
[[1, 2], [1, 5], [2, 6], [3, 4], [3, 6], [4, 5], [6, 7

[[1, 2], [1, 5], [2, 6], [3, 4], [3, 6], [4, 6], [5, 7
[[1, 2], [1, 5], [2, 6], [3, 4], [3, 6], [4, 7], [5, 6
[[1, 2], [1, 5], [2, 6], [3, 4], [3, 7], [4, 6], [5, 6
[[1, 2], [1, 5], [2, 7], [3, 4], [3, 6], [4, 6], [5, 6
[[1, 2], [1, 6], [2, 3], [3, 4], [4, 5], [5, 6], [6, 7
[[1, 2], [1, 6], [2, 3], [3, 4], [4, 6], [5, 6], [5, 7
[[1, 2], [1, 6], [2, 4], [3, 4], [3, 5], [5, 6], [6, 7
[[1, 2], [1, 6], [2, 4], [3, 4], [3, 6], [5, 6], [5, 7
[[1, 2], [1, 6], [2, 6], [3, 4], [3, 5], [4, 5], [6, 7
[[1, 2], [1, 6], [2, 6], [3, 4], [3, 5], [4, 6], [5, 7
[[1, 2], [1, 6], [2, 6], [3, 4], [3, 5], [4, 7], [5, 6
[[1, 2], [1, 6], [2, 6], [3, 4], [3, 6], [4, 5], [5, 7
[[1, 2], [1, 6], [2, 6], [3, 4], [3, 7], [4, 5], [5, 6
[[1, 2], [1, 6], [2, 7], [3, 4], [3, 5], [4, 6], [5, 6
[[1, 2], [1, 6], [2, 7], [3, 4], [3, 6], [4, 5], [5, 6
[[1, 2], [1, 7], [2, 6], [3, 4], [3, 5], [4, 6], [5, 6
[[1, 2], [1, 7], [2, 6], [3, 4], [3, 6], [4, 5], [5, 6
[[1, 3], [1, 5], [2, 4], [2, 6], [3, 4], [5, 6], [6, 7
[[1, 3], [1, 5], [2, 6], [2, 7], [3, 4], [4, 6], [5, 6
[[1, 3], [1, 6], [2, 4], [2, 6], [3, 4], [5, 6], [5, 7
[[1, 3], [1, 6], [2, 6], [2, 7], [3, 4], [4, 5], [5, 6
[[1, 4], [1, 5], [2, 3], [2, 6], [3, 4], [5, 6], [6, 7
[[1, 4], [1, 5], [2, 6], [2, 7], [3, 4], [3, 6], [5, 6
[[1, 4], [1, 6], [2, 3], [2, 6], [3, 4], [5, 6], [5, 7
[[1, 4], [1, 6], [2, 6], [2, 7], [3, 4], [3, 5], [5, 6
[[1, 5], [1, 6], [2, 3], [2, 4], [3, 4], [5, 6], [6, 7
[[1, 5], [1, 6], [2, 3], [2, 6], [3, 4], [4, 5], [6, 7
[[1, 5], [1, 6], [2, 3], [2, 6], [3, 4], [4, 6], [5, 7
[[1, 5], [1, 6], [2, 3], [2, 6], [3, 4], [4, 7], [5, 6
[[1, 5], [1, 6], [2, 3], [2, 7], [3, 4], [4, 6], [5, 6
[[1, 5], [1, 6], [2, 4], [2, 6], [3, 4], [3, 5], [6, 7
[[1, 5], [1, 6], [2, 4], [2, 6], [3, 4], [3, 6], [5, 7
[[1, 5], [1, 6], [2, 4], [2, 6], [3, 4], [3, 7], [5, 6
[[1, 5], [1, 6], [2, 4], [2, 7], [3, 4], [3, 6], [5, 6
[[1, 5], [1, 6], [2, 6], [2, 7], [3, 4], [3, 5], [4, 6
[[1, 5], [1, 6], [2, 6], [2, 7], [3, 4], [3, 6], [4, 5
[[1, 5], [1, 7], [2, 3], [2, 6], [3, 4], [4, 6], [5, 6
[[1, 5], [1, 7], [2, 4], [2, 6], [3, 4], [3, 6], [5, 6
[[1, 6], [1, 7], [2, 3], [2, 6], [3, 4], [4, 5], [5, 6
[[1, 6], [1, 7], [2, 4], [2, 6], [3, 4], [3, 5], [5, 6

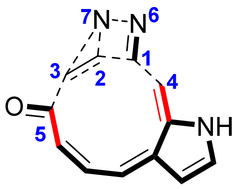

C-x6

[[1, 2], [1, 3], [2, 3], [2, 4], [3, 7], [5, 7], [6, 7
[[1, 2], [1, 3], [2, 3], [2, 5], [3, 7], [4, 7], [6, 7
[[1, 2], [1, 3], [2, 3], [2, 6], [3, 7], [4, 7], [5, 7
[[1, 2], [1, 3], [2, 3], [2, 7], [3, 4], [5, 7], [6, 7
[[1, 2], [1, 3], [2, 3], [2, 7], [3, 5], [4, 7], [6, 7
[[1, 2], [1, 3], [2, 3], [2, 7], [3, 6], [4, 7], [5, 7
[[1, 2], [1, 3], [2, 3], [2, 7], [3, 7], [4, 6], [5, 7
[[1, 2], [1, 3], [2, 3], [2, 7], [3, 7], [4, 7], [5, 6
[[1, 2], [1, 4], [2, 3], [2, 7], [3, 5], [3, 7], [6, 7
[[1, 2], [1, 4], [2, 3], [2, 7], [3, 6], [3, 7], [5, 7
[[1, 2], [1, 5], [2, 3], [2, 7], [3, 4], [3, 7], [6, 7
[[1, 2], [1, 5], [2, 3], [2, 7], [3, 6], [3, 7], [4, 7
[[1, 2], [1, 7], [2, 3], [2, 4], [3, 5], [3, 7], [6, 7
[[1, 2], [1, 7], [2, 3], [2, 4], [3, 6], [3, 7], [5, 7
[[1, 2], [1, 7], [2, 3], [2, 5], [3, 4], [3, 7], [6, 7
[[1, 2], [1, 7], [2, 3], [2, 5], [3, 6], [3, 7], [4, 7
[[1, 2], [1, 7], [2, 3], [2, 6], [3, 4], [3, 7], [5, 7
[[1, 2], [1, 7], [2, 3], [2, 6], [3, 5], [3, 7], [4, 7
[[1, 2], [1, 7], [2, 3], [2, 7], [3, 4], [3, 5], [6, 7
[[1, 2], [1, 7], [2, 3], [2, 7], [3, 4], [3, 6], [5, 7
[[1, 2], [1, 7], [2, 3], [2, 7], [3, 4], [3, 7], [5, 6
[[1, 2], [1, 7], [2, 3], [2, 7], [3, 5], [3, 6], [4, 7
[[1, 2], [1, 7], [2, 3], [2, 7], [3, 5], [3, 7], [4, 6
[[1, 3], [1, 4], [2, 3], [2, 5], [2, 7], [3, 7], [6, 7
[[1, 3], [1, 4], [2, 3], [2, 6], [2, 7], [3, 7], [5, 7
[[1, 3], [1, 5], [2, 3], [2, 4], [2, 7], [3, 7], [6, 7

[[1, 3], [1, 5], [2, 3], [2, 6], [2, 7], [3, 7], [4, 7
[[1, 3], [1, 7], [2, 3], [2, 4], [2, 5], [3, 7], [6, 7
[[1, 3], [1, 7], [2, 3], [2, 4], [2, 6], [3, 7], [5, 7
[[1, 3], [1, 7], [2, 3], [2, 4], [2, 7], [3, 5], [6, 7
[[1, 3], [1, 7], [2, 3], [2, 4], [2, 7], [3, 6], [5, 7
[[1, 3], [1, 7], [2, 3], [2, 4], [2, 7], [3, 7], [5, 6
[[1, 3], [1, 7], [2, 3], [2, 5], [2, 6], [3, 7], [4, 7
[[1, 3], [1, 7], [2, 3], [2, 5], [2, 7], [3, 4], [6, 7
[[1, 3], [1, 7], [2, 3], [2, 5], [2, 7], [3, 6], [4, 7
[[1, 3], [1, 7], [2, 3], [2, 5], [2, 7], [3, 7], [4, 6
[[1, 3], [1, 7], [2, 3], [2, 6], [2, 7], [3, 4], [5, 7
[[1, 3], [1, 7], [2, 3], [2, 6], [2, 7], [3, 5], [4, 7
[[1, 4], [1, 7], [2, 3], [2, 5], [2, 7], [3, 6], [3, 7
[[1, 4], [1, 7], [2, 3], [2, 6], [2, 7], [3, 5], [3, 7
[[1, 5], [1, 7], [2, 3], [2, 4], [2, 7], [3, 6], [3, 7
[[1, 5], [1, 7], [2, 3], [2, 6], [2, 7], [3, 4], [3, 7

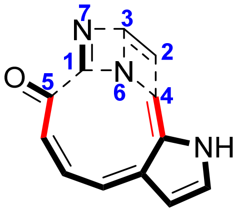

C-p1

[[1, 2], [1, 3], [2, 3], [3, 4], [4, 6], [5, 6], [6, 7
[[1, 2], [1, 3], [2, 3], [3, 6], [4, 5], [4, 6], [6, 7
[[1, 2], [1, 3], [2, 3], [3, 6], [4, 6], [4, 7], [5, 6
[[1, 2], [1, 4], [2, 3], [3, 4], [3, 6], [5, 6], [6, 7
[[1, 2], [1, 4], [2, 3], [3, 5], [3, 6], [4, 6], [6, 7
[[1, 2], [1, 4], [2, 3], [3, 6], [3, 7], [4, 6], [5, 6
[[1, 2], [1, 5], [2, 3], [3, 4], [3, 6], [4, 6], [6, 7
[[1, 2], [1, 6], [2, 3], [3, 4], [3, 5], [4, 6], [6, 7
[[1, 2], [1, 6], [2, 3], [3, 4], [3, 6], [4, 5], [6, 7
[[1, 2], [1, 6], [2, 3], [3, 4], [3, 6], [4, 6], [5, 7
[[1, 2], [1, 6], [2, 3], [3, 4], [3, 6], [4, 7], [5, 6
[[1, 2], [1, 6], [2, 3], [3, 4], [3, 7], [4, 6], [5, 6
[[1, 2], [1, 6], [2, 3], [3, 5], [3, 6], [4, 6], [4, 7
[[1, 2], [1, 6], [2, 3], [3, 6], [3, 7], [4, 5], [4, 6
[[1, 3], [1, 4], [2, 3], [2, 4], [3, 6], [5, 6], [6, 7
[[1, 3], [1, 4], [2, 3], [2, 5], [3, 6], [4, 6], [6, 7
[[1, 3], [1, 4], [2, 3], [2, 6], [3, 4], [5, 6], [6, 7
[[1, 3], [1, 4], [2, 3], [2, 6], [3, 5], [4, 6], [6, 7
[[1, 3], [1, 4], [2, 3], [2, 6], [3, 6], [4, 5], [6, 7
[[1, 3], [1, 4], [2, 3], [2, 6], [3, 6], [4, 6], [5, 7
[[1, 3], [1, 4], [2, 3], [2, 6], [3, 6], [4, 7], [5, 6
[[1, 3], [1, 4], [2, 3], [2, 6], [3, 7], [4, 6], [5, 6
[[1, 3], [1, 4], [2, 3], [2, 7], [3, 6], [4, 6], [5, 6
[[1, 3], [1, 5], [2, 3], [2, 4], [3, 6], [4, 6], [6, 7
[[1, 3], [1, 5], [2, 3], [2, 6], [3, 4], [4, 6], [6, 7
[[1, 3], [1, 5], [2, 3], [2, 6], [3, 6], [4, 6], [4, 7
[[1, 3], [1, 6], [2, 3], [2, 4], [3, 4], [5, 6], [6, 7
[[1, 3], [1, 6], [2, 3], [2, 4], [3, 5], [4, 6], [6, 7
[[1, 3], [1, 6], [2, 3], [2, 4], [3, 6], [4, 5], [6, 7
[[1, 3], [1, 6], [2, 3], [2, 4], [3, 6], [4, 6], [5, 7
[[1, 3], [1, 6], [2, 3], [2, 4], [3, 6], [4, 7], [5, 6
[[1, 3], [1, 6], [2, 3], [2, 4], [3, 7], [4, 6], [5, 6
[[1, 3], [1, 6], [2, 3], [2, 5], [3, 4], [4, 6], [6, 7
[[1, 3], [1, 6], [2, 3], [2, 5], [3, 6], [4, 6], [4, 7
[[1, 3], [1, 6], [2, 3], [2, 6], [3, 4], [4, 5], [6, 7
[[1, 3], [1, 6], [2, 3], [2, 6], [3, 4], [4, 6], [5, 7
[[1, 3], [1, 6], [2, 3], [2, 6], [3, 4], [4, 7], [5, 6
[[1, 3], [1, 6], [2, 3], [2, 6], [3, 5], [4, 6], [4, 7
[[1, 3], [1, 6], [2, 3], [2, 6], [3, 6], [4, 5], [4, 7
[[1, 3], [1, 6], [2, 3], [2, 6], [3, 7], [4, 5], [4, 6
[[1, 3], [1, 6], [2, 3], [2, 7], [3, 4], [4, 6], [5, 6
[[1, 3], [1, 6], [2, 3], [2, 7], [3, 6], [4, 5], [4, 6
[[1, 4], [1, 5], [2, 3], [2, 6], [3, 4], [3, 6], [6, 7
[[1, 4], [1, 5], [2, 3], [2, 6], [3, 6], [3, 7], [4, 6
[[1, 4], [1, 6], [2, 3], [2, 4], [3, 5], [3, 6], [6, 7
[[1, 4], [1, 6], [2, 3], [2, 4], [3, 6], [3, 7], [5, 6
[[1, 4], [1, 6], [2, 3], [2, 5], [3, 4], [3, 6], [6, 7
[[1, 4], [1, 6], [2, 3], [2, 5], [3, 6], [3, 7], [4, 6
[[1, 4], [1, 6], [2, 3], [2, 6], [3, 4], [3, 5], [6, 7

[[1, 4], [1, 6], [2, 3], [2, 6], [3, 4], [3, 6], [5, 7
[[1, 4], [1, 6], [2, 3], [2, 6], [3, 4], [3, 7], [5, 6
[[1, 4], [1, 6], [2, 3], [2, 6], [3, 5], [3, 6], [4, 7
[[1, 4], [1, 6], [2, 3], [2, 6], [3, 5], [3, 7], [4, 6
[[1, 4], [1, 6], [2, 3], [2, 6], [3, 6], [3, 7], [4, 5
[[1, 4], [1, 6], [2, 3], [2, 7], [3, 4], [3, 6], [5, 6
[[1, 4], [1, 6], [2, 3], [2, 7], [3, 5], [3, 6], [4, 6
[[1, 5], [1, 6], [2, 3], [2, 4], [3, 4], [3, 6], [6, 7
[[1, 5], [1, 6], [2, 3], [2, 4], [3, 6], [3, 7], [4, 6
[[1, 5], [1, 6], [2, 3], [2, 6], [3, 4], [3, 6], [4, 7
[[1, 5], [1, 6], [2, 3], [2, 6], [3, 4], [3, 7], [4, 6
[[1, 5], [1, 6], [2, 3], [2, 7], [3, 4], [3, 6], [4, 6

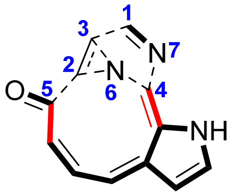

C-x7

[[1, 2], [2, 3], [2, 4], [3, 4], [3, 6], [5, 6], [6, 7
[[1, 2], [2, 3], [2, 4], [3, 5], [3, 6], [4, 6], [6, 7
[[1, 2], [2, 3], [2, 4], [3, 6], [3, 7], [4, 6], [5, 6
[[1, 2], [2, 3], [2, 5], [3, 4], [3, 6], [4, 6], [6, 7
[[1, 2], [2, 3], [2, 6], [3, 4], [3, 5], [4, 6], [6, 7
[[1, 2], [2, 3], [2, 6], [3, 4], [3, 6], [4, 5], [6, 7
[[1, 2], [2, 3], [2, 6], [3, 4], [3, 6], [4, 6], [5, 7
[[1, 2], [2, 3], [2, 6], [3, 4], [3, 6], [4, 7], [5, 6
[[1, 2], [2, 3], [2, 6], [3, 4], [3, 7], [4, 6], [5, 6
[[1, 2], [2, 3], [2, 6], [3, 5], [3, 6], [4, 6], [4, 7
[[1, 2], [2, 3], [2, 6], [3, 6], [3, 7], [4, 5], [4, 6
[[1, 2], [2, 3], [2, 7], [3, 4], [3, 6], [4, 6], [5, 6
[[1, 3], [2, 3], [2, 4], [2, 5], [3, 6], [4, 6], [6, 7
[[1, 3], [2, 3], [2, 4], [2, 6], [3, 4], [5, 6], [6, 7
[[1, 3], [2, 3], [2, 4], [2, 6], [3, 5], [4, 6], [6, 7
[[1, 3], [2, 3], [2, 4], [2, 6], [3, 6], [4, 5], [6, 7
[[1, 3], [2, 3], [2, 4], [2, 6], [3, 6], [4, 6], [5, 7
[[1, 3], [2, 3], [2, 4], [2, 6], [3, 6], [4, 7], [5, 6
[[1, 3], [2, 3], [2, 4], [2, 6], [3, 7], [4, 6], [5, 6
[[1, 3], [2, 3], [2, 4], [2, 7], [3, 6], [4, 6], [5, 6
[[1, 3], [2, 3], [2, 5], [2, 6], [3, 4], [4, 6], [6, 7
[[1, 3], [2, 3], [2, 5], [2, 6], [3, 6], [4, 6], [4, 7
[[1, 3], [2, 3], [2, 6], [2, 7], [3, 4], [4, 6], [5, 6
[[1, 3], [2, 3], [2, 6], [2, 7], [3, 6], [4, 5], [4, 6
[[1, 4], [2, 3], [2, 4], [2, 6], [3, 5], [3, 6], [6, 7
[[1, 4], [2, 3], [2, 4], [2, 6], [3, 6], [3, 7], [5, 6
[[1, 4], [2, 3], [2, 5], [2, 6], [3, 4], [3, 6], [6, 7
[[1, 4], [2, 3], [2, 5], [2, 6], [3, 6], [3, 7], [4, 6
[[1, 4], [2, 3], [2, 6], [2, 7], [3, 4], [3, 6], [5, 6
[[1, 4], [2, 3], [2, 6], [2, 7], [3, 5], [3, 6], [4, 6
[[1, 5], [2, 3], [2, 4], [2, 6], [3, 4], [3, 6], [6, 7
[[1, 5], [2, 3], [2, 4], [2, 6], [3, 6], [3, 7], [4, 6
[[1, 5], [2, 3], [2, 6], [2, 7], [3, 4], [3, 6], [4, 6
[[1, 6], [2, 3], [2, 4], [2, 5], [3, 4], [3, 6], [6, 7
[[1, 6], [2, 3], [2, 4], [2, 5], [3, 6], [3, 7], [4, 6
[[1, 6], [2, 3], [2, 4], [2, 6], [3, 4], [3, 5], [6, 7
[[1, 6], [2, 3], [2, 4], [2, 6], [3, 4], [3, 6], [5, 7
[[1, 6], [2, 3], [2, 4], [2, 6], [3, 4], [3, 7], [5, 6
[[1, 6], [2, 3], [2, 4], [2, 6], [3, 5], [3, 6], [4, 7
[[1, 6], [2, 3], [2, 4], [2, 6], [3, 5], [3, 7], [4, 6
[[1, 6], [2, 3], [2, 4], [2, 6], [3, 6], [3, 7], [4, 5
[[1, 6], [2, 3], [2, 4], [2, 7], [3, 4], [3, 6], [5, 6
[[1, 6], [2, 3], [2, 4], [2, 7], [3, 5], [3, 6], [4, 6
[[1, 6], [2, 3], [2, 5], [2, 6], [3, 4], [3, 6], [4, 7
[[1, 6], [2, 3], [2, 5], [2, 6], [3, 4], [3, 7], [4, 6
[[1, 6], [2, 3], [2, 5], [2, 7], [3, 4], [3, 6], [4, 6
[[1, 6], [2, 3], [2, 6], [2, 7], [3, 4], [3, 5], [4, 6
[[1, 6], [2, 3], [2, 6], [2, 7], [3, 4], [3, 6], [4, 5

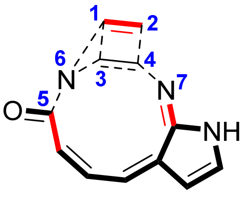

C-r1

[[1, 3], [1, 4], [2, 3], [3, 4], [4, 6], [5, 6], [6, 7
[[1, 3], [1, 4], [2, 4], [3, 4], [3, 6], [5, 6], [6, 7
[[1, 3], [1, 4], [2, 5], [3, 4], [3, 6], [4, 6], [6, 7
[[1, 3], [1, 4], [2, 6], [3, 4], [3, 5], [4, 6], [6, 7
[[1, 3], [1, 4], [2, 6], [3, 4], [3, 6], [4, 5], [6, 7
[[1, 3], [1, 4], [2, 6], [3, 4], [3, 6], [4, 7], [5, 6
[[1, 3], [1, 4], [2, 6], [3, 4], [3, 7], [4, 6], [5, 6
[[1, 3], [1, 4], [2, 7], [3, 4], [3, 6], [4, 6], [5, 6
[[1, 3], [1, 5], [2, 4], [3, 4], [3, 6], [4, 6], [6, 7
[[1, 3], [1, 5], [2, 6], [3, 4], [3, 6], [4, 6], [4, 7
[[1, 3], [1, 6], [2, 3], [3, 4], [4, 5], [4, 6], [6, 7
[[1, 3], [1, 6], [2, 3], [3, 4], [4, 6], [4, 7], [5, 6
[[1, 3], [1, 6], [2, 4], [3, 4], [3, 5], [4, 6], [6, 7
[[1, 3], [1, 6], [2, 4], [3, 4], [3, 6], [4, 5], [6, 7
[[1, 3], [1, 6], [2, 4], [3, 4], [3, 6], [4, 7], [5, 6
[[1, 3], [1, 6], [2, 4], [3, 4], [3, 7], [4, 6], [5, 6
[[1, 3], [1, 6], [2, 5], [3, 4], [3, 6], [4, 6], [4, 7
[[1, 3], [1, 6], [2, 6], [3, 4], [3, 5], [4, 6], [4, 7
[[1, 3], [1, 6], [2, 6], [3, 4], [3, 6], [4, 5], [4, 7
[[1, 3], [1, 6], [2, 6], [3, 4], [3, 7], [4, 5], [4, 6
[[1, 3], [1, 6], [2, 7], [3, 4], [3, 6], [4, 5], [4, 6
[[1, 3], [1, 7], [2, 4], [3, 4], [3, 6], [4, 6], [5, 6
[[1, 3], [1, 7], [2, 6], [3, 4], [3, 6], [4, 5], [4, 6
[[1, 4], [1, 5], [2, 3], [3, 4], [3, 6], [4, 6], [6, 7
[[1, 4], [1, 5], [2, 6], [3, 4], [3, 6], [3, 7], [4, 6
[[1, 4], [1, 6], [2, 3], [3, 4], [3, 5], [4, 6], [6, 7
[[1, 4], [1, 6], [2, 3], [3, 4], [3, 6], [4, 5], [6, 7
[[1, 4], [1, 6], [2, 3], [3, 4], [3, 6], [4, 7], [5, 6
[[1, 4], [1, 6], [2, 3], [3, 4], [3, 7], [4, 6], [5, 6
[[1, 4], [1, 6], [2, 4], [3, 4], [3, 5], [3, 6], [6, 7
[[1, 4], [1, 6], [2, 4], [3, 4], [3, 6], [3, 7], [5, 6
[[1, 4], [1, 6], [2, 5], [3, 4], [3, 6], [3, 7], [4, 6
[[1, 4], [1, 6], [2, 6], [3, 4], [3, 5], [3, 6], [4, 7
[[1, 4], [1, 6], [2, 6], [3, 4], [3, 5], [3, 7], [4, 6
[[1, 4], [1, 6], [2, 6], [3, 4], [3, 6], [3, 7], [4, 5
[[1, 4], [1, 6], [2, 7], [3, 4], [3, 5], [3, 6], [4, 6
[[1, 4], [1, 7], [2, 3], [3, 4], [3, 6], [4, 6], [5, 6
[[1, 4], [1, 7], [2, 6], [3, 4], [3, 5], [3, 6], [4, 6
[[1, 5], [1, 6], [2, 3], [3, 4], [3, 6], [4, 6], [4, 7
[[1, 5], [1, 6], [2, 4], [3, 4], [3, 6], [3, 7], [4, 6
[[1, 6], [1, 7], [2, 3], [3, 4], [3, 6], [4, 5], [4, 6
[[1, 6], [1, 7], [2, 4], [3, 4], [3, 5], [3, 6], [4, 6]]]

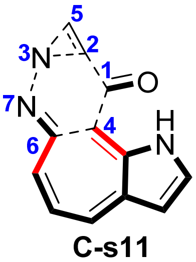

C-s11

[[1, 2], [1, 3], [2, 3], [2, 5], [3, 4], [4, 6], [5, 7
[[1, 2], [1, 3], [2, 3], [2, 5], [3, 4], [4, 7], [5, 6
[[1, 2], [1, 3], [2, 3], [2, 5], [3, 6], [4, 5], [4, 7
[[1, 2], [1, 3], [2, 3], [2, 5], [3, 7], [4, 5], [4, 6
[[1, 2], [1, 3], [2, 4], [2, 5], [3, 4], [3, 6], [5, 7
[[1, 2], [1, 3], [2, 4], [2, 5], [3, 4], [3, 7], [5, 6
[[1, 2], [1, 3], [2, 4], [2, 5], [3, 5], [3, 6], [4, 7
[[1, 2], [1, 3], [2, 4], [2, 5], [3, 5], [3, 7], [4, 6
[[1, 2], [1, 3], [2, 4], [2, 5], [3, 6], [3, 7], [4, 5
[[1, 2], [1, 3], [2, 5], [2, 6], [3, 4], [3, 5], [4, 7
[[1, 2], [1, 3], [2, 5], [2, 6], [3, 4], [3, 7], [4, 5

[[1, 2], [1, 3], [2, 5], [2, 7], [3, 4], [3, 5], [4, 6
[[1, 2], [1, 3], [2, 5], [2, 7], [3, 4], [3, 6], [4, 5
[[1, 2], [1, 4], [2, 3], [2, 5], [3, 4], [3, 6], [5, 7
[[1, 2], [1, 4], [2, 3], [2, 5], [3, 4], [3, 7], [5, 6
[[1, 2], [1, 4], [2, 3], [2, 5], [3, 5], [3, 6], [4, 7
[[1, 2], [1, 4], [2, 3], [2, 5], [3, 5], [3, 7], [4, 6
[[1, 2], [1, 4], [2, 3], [2, 5], [3, 6], [3, 7], [4, 5
[[1, 2], [1, 4], [2, 4], [2, 5], [3, 5], [3, 6], [3, 7
[[1, 2], [1, 4], [2, 5], [2, 6], [3, 4], [3, 5], [3, 7
[[1, 2], [1, 4], [2, 5], [2, 7], [3, 4], [3, 5], [3, 6
[[1, 2], [1, 5], [2, 3], [2, 5], [3, 4], [3, 6], [4, 7
[[1, 2], [1, 5], [2, 3], [2, 5], [3, 4], [3, 7], [4, 6
[[1, 2], [1, 5], [2, 4], [2, 5], [3, 4], [3, 6], [3, 7
[[1, 2], [1, 6], [2, 3], [2, 5], [3, 4], [3, 5], [4, 7
[[1, 2], [1, 6], [2, 3], [2, 5], [3, 4], [3, 7], [4, 5
[[1, 2], [1, 6], [2, 4], [2, 5], [3, 4], [3, 5], [3, 7
[[1, 2], [1, 7], [2, 3], [2, 5], [3, 4], [3, 5], [4, 6
[[1, 2], [1, 7], [2, 3], [2, 5], [3, 4], [3, 6], [4, 5
[[1, 2], [1, 7], [2, 4], [2, 5], [3, 4], [3, 5], [3, 6
[[1, 3], [1, 4], [2, 3], [2, 4], [2, 5], [3, 6], [5, 7
[[1, 3], [1, 4], [2, 3], [2, 4], [2, 5], [3, 7], [5, 6
[[1, 3], [1, 4], [2, 3], [2, 5], [2, 6], [3, 4], [5, 7
[[1, 3], [1, 4], [2, 3], [2, 5], [2, 6], [3, 5], [4, 7
[[1, 3], [1, 4], [2, 3], [2, 5], [2, 6], [3, 7], [4, 5
[[1, 3], [1, 4], [2, 3], [2, 5], [2, 7], [3, 4], [5, 6
[[1, 3], [1, 4], [2, 3], [2, 5], [2, 7], [3, 5], [4, 6
[[1, 3], [1, 4], [2, 3], [2, 5], [2, 7], [3, 6], [4, 5
[[1, 3], [1, 4], [2, 4], [2, 5], [2, 6], [3, 5], [3, 7
[[1, 3], [1, 4], [2, 4], [2, 5], [2, 7], [3, 5], [3, 6
[[1, 3], [1, 4], [2, 5], [2, 6], [2, 7], [3, 4], [3, 5
[[1, 3], [1, 5], [2, 3], [2, 4], [2, 5], [3, 6], [4, 7
[[1, 3], [1, 5], [2, 3], [2, 4], [2, 5], [3, 7], [4, 6
[[1, 3], [1, 5], [2, 3], [2, 5], [2, 6], [3, 4], [4, 7
[[1, 3], [1, 5], [2, 3], [2, 5], [2, 7], [3, 4], [4, 6
[[1, 3], [1, 5], [2, 4], [2, 5], [2, 6], [3, 4], [3, 7
[[1, 3], [1, 5], [2, 4], [2, 5], [2, 7], [3, 4], [3, 6
[[1, 3], [1, 6], [2, 3], [2, 4], [2, 5], [3, 4], [5, 7
[[1, 3], [1, 6], [2, 3], [2, 4], [2, 5], [3, 5], [4, 7
[[1, 3], [1, 6], [2, 3], [2, 4], [2, 5], [3, 7], [4, 5
[[1, 3], [1, 6], [2, 3], [2, 5], [2, 7], [3, 4], [4, 5
[[1, 3], [1, 6], [2, 4], [2, 5], [2, 7], [3, 4], [3, 5
[[1, 3], [1, 7], [2, 3], [2, 4], [2, 5], [3, 4], [5, 6
[[1, 3], [1, 7], [2, 3], [2, 4], [2, 5], [3, 5], [4, 6
[[1, 3], [1, 7], [2, 3], [2, 4], [2, 5], [3, 6], [4, 5
[[1, 3], [1, 7], [2, 3], [2, 5], [2, 6], [3, 4], [4, 5
[[1, 3], [1, 7], [2, 4], [2, 5], [2, 6], [3, 4], [3, 5
[[1, 4], [1, 5], [2, 3], [2, 4], [2, 5], [3, 6], [3, 7
[[1, 4], [1, 5], [2, 3], [2, 5], [2, 6], [3, 4], [3, 7
[[1, 4], [1, 5], [2, 3], [2, 5], [2, 7], [3, 4], [3, 6
[[1, 4], [1, 6], [2, 3], [2, 4], [2, 5], [3, 5], [3, 7
[[1, 4], [1, 6], [2, 3], [2, 5], [2, 7], [3, 4], [3, 5
[[1, 4], [1, 7], [2, 3], [2, 4], [2, 5], [3, 5], [3, 6
[[1, 4], [1, 7], [2, 3], [2, 5], [2, 6], [3, 4], [3, 5
[[1, 5], [1, 6], [2, 3], [2, 4], [2, 5], [3, 4], [3, 7
[[1, 5], [1, 7], [2, 3], [2, 4], [2, 5], [3, 4], [3, 6

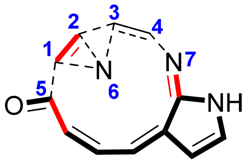

C-x8

1[[1, 3], [1, 4], [2, 3], [2, 6], [3, 4], [5, 6], [6, 7
[[1, 3], [1, 4], [2, 5], [2, 6], [3, 4], [3, 6], [6, 7
[[1, 3], [1, 4], [2, 6], [2, 7], [3, 4], [3, 6], [5, 6
[[1, 3], [1, 5], [2, 3], [2, 6], [3, 4], [4, 6], [6, 7
[[1, 3], [1, 5], [2, 4], [2, 6], [3, 4], [3, 6], [6, 7
[[1, 3], [1, 5], [2, 6], [2, 7], [3, 4], [3, 6], [4, 6
[[1, 3], [1, 6], [2, 3], [2, 4], [3, 4], [5, 6], [6, 7
[[1, 3], [1, 6], [2, 3], [2, 5], [3, 4], [4, 6], [6, 7
[[1, 3], [1, 6], [2, 3], [2, 6], [3, 4], [4, 5], [6, 7
[[1, 3], [1, 6], [2, 3], [2, 6], [3, 4], [4, 7], [5, 6
[[1, 3], [1, 6], [2, 3], [2, 7], [3, 4], [4, 6], [5, 6

[[1, 3], [1, 6], [2, 4], [2, 5], [3, 4], [3, 6], [6, 7
[[1, 3], [1, 6], [2, 4], [2, 6], [3, 4], [3, 5], [6, 7
[[1, 3], [1, 6], [2, 4], [2, 6], [3, 4], [3, 7], [5, 6
[[1, 3], [1, 6], [2, 4], [2, 7], [3, 4], [3, 6], [5, 6
[[1, 3], [1, 6], [2, 5], [2, 6], [3, 4], [3, 6], [4, 7
[[1, 3], [1, 6], [2, 5], [2, 6], [3, 4], [3, 7], [4, 6
[[1, 3], [1, 6], [2, 5], [2, 7], [3, 4], [3, 6], [4, 6
[[1, 3], [1, 6], [2, 6], [2, 7], [3, 4], [3, 5], [4, 6
[[1, 3], [1, 6], [2, 6], [2, 7], [3, 4], [3, 6], [4, 5
[[1, 3], [1, 7], [2, 3], [2, 6], [3, 4], [4, 6], [5, 6
[[1, 3], [1, 7], [2, 4], [2, 6], [3, 4], [3, 6], [5, 6
[[1, 3], [1, 7], [2, 5], [2, 6], [3, 4], [3, 6], [4, 6
[[1, 4], [1, 5], [2, 3], [2, 6], [3, 4], [3, 6], [6, 7
[[1, 4], [1, 6], [2, 3], [2, 5], [3, 4], [3, 6], [6, 7
[[1, 4], [1, 6], [2, 3], [2, 6], [3, 4], [3, 5], [6, 7
[[1, 4], [1, 6], [2, 3], [2, 6], [3, 4], [3, 7], [5, 6
[[1, 4], [1, 6], [2, 3], [2, 7], [3, 4], [3, 6], [5, 6
[[1, 4], [1, 6], [2, 5], [2, 6], [3, 4], [3, 6], [3, 7
[[1, 4], [1, 6], [2, 6], [2, 7], [3, 4], [3, 5], [3, 6
[[1, 4], [1, 7], [2, 3], [2, 6], [3, 4], [3, 6], [5, 6
[[1, 5], [1, 6], [2, 3], [2, 4], [3, 4], [3, 6], [6, 7
[[1, 5], [1, 6], [2, 3], [2, 6], [3, 4], [3, 6], [4, 7
[[1, 5], [1, 6], [2, 3], [2, 6], [3, 4], [3, 7], [4, 6
[[1, 5], [1, 6], [2, 3], [2, 7], [3, 4], [3, 6], [4, 6
[[1, 5], [1, 6], [2, 4], [2, 6], [3, 4], [3, 6], [3, 7
[[1, 5], [1, 7], [2, 3], [2, 6], [3, 4], [3, 6], [4, 6
[[1, 6], [1, 7], [2, 3], [2, 4], [3, 4], [3, 6], [5, 6
[[1, 6], [1, 7], [2, 3], [2, 5], [3, 4], [3, 6], [4, 6
[[1, 6], [1, 7], [2, 3], [2, 6], [3, 4], [3, 5], [4, 6
[[1, 6], [1, 7], [2, 3], [2, 6], [3, 4], [3, 6], [4, 5
[[1, 6], [1, 7], [2, 4], [2, 6], [3, 4], [3, 5], [3, 6]]]

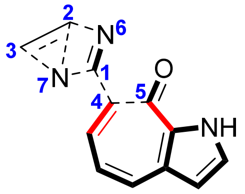

C-q1

[[1, 2], [1, 3], [2, 3], [2, 4], [4, 7], [5, 7], [6, 7
[[1, 2], [1, 3], [2, 3], [2, 7], [4, 5], [4, 7], [6, 7
[[1, 2], [1, 3], [2, 3], [2, 7], [4, 6], [4, 7], [5, 7
[[1, 2], [1, 4], [2, 3], [2, 4], [3, 7], [5, 7], [6, 7
[[1, 2], [1, 4], [2, 3], [2, 5], [3, 7], [4, 7], [6, 7
[[1, 2], [1, 4], [2, 3], [2, 6], [3, 7], [4, 7], [5, 7
[[1, 2], [1, 4], [2, 3], [2, 7], [3, 4], [5, 7], [6, 7
[[1, 2], [1, 4], [2, 3], [2, 7], [3, 5], [4, 7], [6, 7
[[1, 2], [1, 4], [2, 3], [2, 7], [3, 6], [4, 7], [5, 7
[[1, 2], [1, 4], [2, 3], [2, 7], [3, 7], [4, 5], [6, 7
[[1, 2], [1, 4], [2, 3], [2, 7], [3, 7], [4, 6], [5, 7
[[1, 2], [1, 4], [2, 3], [2, 7], [3, 7], [4, 7], [5, 6
[[1, 2], [1, 5], [2, 3], [2, 4], [3, 7], [4, 7], [6, 7
[[1, 2], [1, 5], [2, 3], [2, 7], [3, 4], [4, 7], [6, 7
[[1, 2], [1, 5], [2, 3], [2, 7], [3, 7], [4, 6], [4, 7
[[1, 2], [1, 7], [2, 3], [2, 4], [3, 4], [5, 7], [6, 7
[[1, 2], [1, 7], [2, 3], [2, 4], [3, 5], [4, 7], [6, 7
[[1, 2], [1, 7], [2, 3], [2, 4], [3, 6], [4, 7], [5, 7
[[1, 2], [1, 7], [2, 3], [2, 4], [3, 7], [4, 5], [6, 7
[[1, 2], [1, 7], [2, 3], [2, 4], [3, 7], [4, 6], [5, 7
[[1, 2], [1, 7], [2, 3], [2, 4], [3, 7], [4, 7], [5, 6
[[1, 2], [1, 7], [2, 3], [2, 5], [3, 4], [4, 7], [6, 7
[[1, 2], [1, 7], [2, 3], [2, 5], [3, 7], [4, 6], [4, 7
[[1, 2], [1, 7], [2, 3], [2, 6], [3, 4], [4, 7], [5, 7
[[1, 2], [1, 7], [2, 3], [2, 6], [3, 7], [4, 5], [4, 7
[[1, 2], [1, 7], [2, 3], [2, 7], [3, 4], [4, 5], [6, 7
[[1, 2], [1, 7], [2, 3], [2, 7], [3, 4], [4, 6], [5, 7
[[1, 2], [1, 7], [2, 3], [2, 7], [3, 4], [4, 7], [5, 6
[[1, 2], [1, 7], [2, 3], [2, 7], [3, 5], [4, 6], [4, 7
[[1, 2], [1, 7], [2, 3], [2, 7], [3, 6], [4, 5], [4, 7
[[1, 2], [1, 7], [2, 3], [2, 7], [3, 7], [4, 5], [4, 6
[[1, 3], [1, 4], [2, 3], [2, 4], [2, 7], [5, 7], [6, 7
[[1, 3], [1, 4], [2, 3], [2, 5], [2, 7], [4, 7], [6, 7
[[1, 3], [1, 4], [2, 3], [2, 6], [2, 7], [4, 7], [5, 7

[[1, 3], [1, 5], [2, 3], [2, 4], [2, 7], [4, 7], [6, 7
[[1, 3], [1, 7], [2, 3], [2, 4], [2, 5], [4, 7], [6, 7
[[1, 3], [1, 7], [2, 3], [2, 4], [2, 6], [4, 7], [5, 7
[[1, 3], [1, 7], [2, 3], [2, 4], [2, 7], [4, 5], [6, 7
[[1, 3], [1, 7], [2, 3], [2, 4], [2, 7], [4, 6], [5, 7
[[1, 3], [1, 7], [2, 3], [2, 4], [2, 7], [4, 7], [5, 6
[[1, 3], [1, 7], [2, 3], [2, 5], [2, 7], [4, 6], [4, 7
[[1, 3], [1, 7], [2, 3], [2, 6], [2, 7], [4, 5], [4, 7
[[1, 4], [1, 5], [2, 3], [2, 4], [2, 7], [3, 7], [6, 7
[[1, 4], [1, 5], [2, 3], [2, 6], [2, 7], [3, 7], [4, 7
[[1, 4], [1, 7], [2, 3], [2, 4], [2, 5], [3, 7], [6, 7
[[1, 4], [1, 7], [2, 3], [2, 4], [2, 6], [3, 7], [5, 7
[[1, 4], [1, 7], [2, 3], [2, 4], [2, 7], [3, 5], [6, 7
[[1, 4], [1, 7], [2, 3], [2, 4], [2, 7], [3, 6], [5, 7
[[1, 4], [1, 7], [2, 3], [2, 4], [2, 7], [3, 7], [5, 6
[[1, 4], [1, 7], [2, 3], [2, 5], [2, 6], [3, 7], [4, 7
[[1, 4], [1, 7], [2, 3], [2, 5], [2, 7], [3, 4], [6, 7
[[1, 4], [1, 7], [2, 3], [2, 5], [2, 7], [3, 6], [4, 7
[[1, 4], [1, 7], [2, 3], [2, 5], [2, 7], [3, 7], [4, 6
[[1, 4], [1, 7], [2, 3], [2, 6], [2, 7], [3, 4], [5, 7
[[1, 4], [1, 7], [2, 3], [2, 6], [2, 7], [3, 5], [4, 7
[[1, 4], [1, 7], [2, 3], [2, 6], [2, 7], [3, 7], [4, 5
[[1, 5], [1, 7], [2, 3], [2, 4], [2, 6], [3, 7], [4, 7
[[1, 5], [1, 7], [2, 3], [2, 4], [2, 7], [3, 4], [6, 7
[[1, 5], [1, 7], [2, 3], [2, 4], [2, 7], [3, 6], [4, 7
[[1, 5], [1, 7], [2, 3], [2, 4], [2, 7], [3, 7], [4, 6
[[1, 5], [1, 7], [2, 3], [2, 6], [2, 7], [3, 4], [4, 7

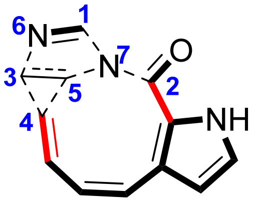

C-x9

[[1, 2], [3, 4], [3, 5], [3, 7], [4, 5], [5, 7], [6, 7
[[1, 2], [3, 4], [3, 5], [3, 7], [4, 7], [5, 6], [5, 7
[[1, 2], [3, 5], [3, 6], [3, 7], [4, 5], [4, 7], [5, 7
[[1, 3], [2, 3], [3, 5], [4, 5], [4, 7], [5, 7], [6, 7
[[1, 3], [2, 4], [3, 5], [3, 7], [4, 5], [5, 7], [6, 7
[[1, 3], [2, 4], [3, 5], [3, 7], [4, 7], [5, 6], [5, 7
[[1, 3], [2, 5], [3, 4], [3, 5], [4, 7], [5, 7], [6, 7
[[1, 3], [2, 5], [3, 5], [3, 7], [4, 5], [4, 7], [6, 7
[[1, 3], [2, 5], [3, 5], [3, 7], [4, 6], [4, 7], [5, 7
[[1, 3], [2, 6], [3, 5], [3, 7], [4, 5], [4, 7], [5, 7
[[1, 3], [2, 7], [3, 4], [3, 5], [4, 5], [5, 7], [6, 7
[[1, 3], [2, 7], [3, 4], [3, 5], [4, 7], [5, 6], [5, 7
[[1, 3], [2, 7], [3, 5], [3, 6], [4, 5], [4, 7], [5, 7
[[1, 3], [2, 7], [3, 5], [3, 7], [4, 5], [4, 6], [5, 7
[[1, 3], [2, 7], [3, 5], [3, 7], [4, 5], [4, 7], [5, 6
[[1, 4], [2, 3], [3, 5], [3, 7], [4, 5], [5, 7], [6, 7
[[1, 4], [2, 3], [3, 5], [3, 7], [4, 7], [5, 6], [5, 7
[[1, 4], [2, 5], [3, 4], [3, 5], [3, 7], [5, 7], [6, 7
[[1, 4], [2, 5], [3, 5], [3, 6], [3, 7], [4, 7], [5, 7
[[1, 4], [2, 7], [3, 4], [3, 5], [3, 7], [5, 6], [5, 7
[[1, 4], [2, 7], [3, 5], [3, 6], [3, 7], [4, 5], [5, 7
[[1, 5], [2, 3], [3, 4], [3, 5], [4, 7], [5, 7], [6, 7
[[1, 5], [2, 3], [3, 5], [3, 7], [4, 5], [4, 7], [6, 7
[[1, 5], [2, 3], [3, 5], [3, 7], [4, 6], [4, 7], [5, 7
[[1, 5], [2, 4], [3, 4], [3, 5], [3, 7], [5, 7], [6, 7
[[1, 5], [2, 4], [3, 5], [3, 6], [3, 7], [4, 7], [5, 7
[[1, 5], [2, 5], [3, 4], [3, 5], [3, 7], [4, 7], [6, 7
[[1, 5], [2, 6], [3, 4], [3, 5], [3, 7], [4, 7], [5, 7
[[1, 5], [2, 7], [3, 4], [3, 5], [3, 6], [4, 7], [5, 7
[[1, 5], [2, 7], [3, 4], [3, 5], [3, 7], [4, 5], [6, 7
[[1, 5], [2, 7], [3, 4], [3, 5], [3, 7], [4, 6], [5, 7
[[1, 5], [2, 7], [3, 4], [3, 5], [3, 7], [4, 7], [5, 6
[[1, 5], [2, 7], [3, 5], [3, 6], [3, 7], [4, 5], [4, 7
[[1, 7], [2, 3], [3, 4], [3, 5], [4, 5], [5, 7], [6, 7
[[1, 7], [2, 3], [3, 4], [3, 5], [4, 7], [5, 6], [5, 7
[[1, 7], [2, 3], [3, 5], [3, 6], [4, 5], [4, 7], [5, 7
[[1, 7], [2, 3], [3, 5], [3, 7], [4, 5], [4, 6], [5, 7

[[1, 7], [2, 3], [3, 5], [3, 7], [4, 5], [4, 7], [5, 6
[[1, 7], [2, 4], [3, 4], [3, 5], [3, 7], [5, 6], [5, 7
[[1, 7], [2, 4], [3, 5], [3, 6], [3, 7], [4, 5], [5, 7
[[1, 7], [2, 5], [3, 4], [3, 5], [3, 6], [4, 7], [5, 7
[[1, 7], [2, 5], [3, 4], [3, 5], [3, 7], [4, 5], [6, 7
[[1, 7], [2, 5], [3, 4], [3, 5], [3, 7], [4, 6], [5, 7
[[1, 7], [2, 5], [3, 4], [3, 5], [3, 7], [4, 7], [5, 6
[[1, 7], [2, 5], [3, 5], [3, 6], [3, 7], [4, 5], [4, 7
[[1, 7], [2, 6], [3, 4], [3, 5], [3, 7], [4, 5], [5, 7
[[1, 7], [2, 7], [3, 4], [3, 5], [3, 6], [4, 5], [5, 7
[[1, 7], [2, 7], [3, 4], [3, 5], [3, 7], [4, 5], [5, 6

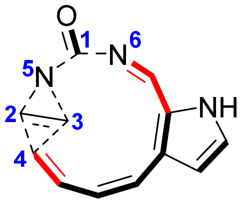

C-p11

[[1, 2], [1, 3], [2, 3], [2, 4], [3, 5], [4, 5], [5, 6
[[1, 2], [1, 3], [2, 3], [2, 5], [3, 4], [4, 5], [5, 6
[[1, 2], [1, 3], [2, 3], [2, 5], [3, 5], [4, 5], [4, 6
[[1, 2], [1, 4], [2, 3], [2, 5], [3, 4], [3, 5], [5, 6
[[1, 2], [1, 4], [2, 3], [2, 5], [3, 5], [3, 6], [4, 5
[[1, 2], [1, 5], [2, 3], [2, 4], [3, 4], [3, 5], [5, 6
[[1, 2], [1, 5], [2, 3], [2, 4], [3, 5], [3, 6], [4, 5
[[1, 2], [1, 5], [2, 3], [2, 5], [3, 4], [3, 5], [4, 6
[[1, 2], [1, 5], [2, 3], [2, 5], [3, 4], [3, 6], [4, 5
[[1, 2], [1, 5], [2, 3], [2, 6], [3, 4], [3, 5], [4, 5
[[1, 2], [1, 6], [2, 3], [2, 5], [3, 4], [3, 5], [4, 5
[[1, 3], [1, 4], [2, 3], [2, 4], [2, 5], [3, 5], [5, 6
[[1, 3], [1, 4], [2, 3], [2, 5], [2, 6], [3, 5], [4, 5
[[1, 3], [1, 5], [2, 3], [2, 4], [2, 5], [3, 4], [5, 6
[[1, 3], [1, 5], [2, 3], [2, 4], [2, 5], [3, 5], [4, 6
[[1, 3], [1, 5], [2, 3], [2, 4], [2, 5], [3, 6], [4, 5
[[1, 3], [1, 5], [2, 3], [2, 4], [2, 6], [3, 5], [4, 5
[[1, 3], [1, 5], [2, 3], [2, 5], [2, 6], [3, 4], [4, 5
[[1, 3], [1, 6], [2, 3], [2, 4], [2, 5], [3, 5], [4, 5
[[1, 4], [1, 5], [2, 3], [2, 4], [2, 5], [3, 5], [3, 6
[[1, 4], [1, 5], [2, 3], [2, 5], [2, 6], [3, 4], [3, 5
[[1, 5], [1, 6], [2, 3], [2, 4], [2, 5], [3, 4], [3, 5]]]

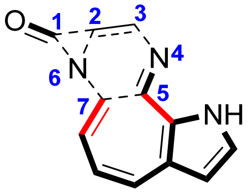

C-q11

[[1, 2], [1, 3], [2, 3], [2, 6], [4, 6], [5, 7], [6, 7
[[1, 2], [1, 3], [2, 3], [2, 6], [4, 7], [5, 6], [6, 7
[[1, 2], [1, 3], [2, 3], [2, 7], [4, 6], [5, 6], [6, 7
[[1, 2], [1, 4], [2, 3], [2, 6], [3, 6], [5, 7], [6, 7
[[1, 2], [1, 4], [2, 3], [2, 6], [3, 7], [5, 6], [6, 7
[[1, 2], [1, 4], [2, 3], [2, 7], [3, 6], [5, 6], [6, 7
[[1, 2], [1, 5], [2, 3], [2, 6], [3, 6], [4, 7], [6, 7
[[1, 2], [1, 5], [2, 3], [2, 6], [3, 7], [4, 6], [6, 7
[[1, 2], [1, 5], [2, 3], [2, 7], [3, 6], [4, 6], [6, 7
[[1, 2], [1, 6], [2, 3], [2, 4], [3, 6], [5, 7], [6, 7
[[1, 2], [1, 6], [2, 3], [2, 4], [3, 7], [5, 6], [6, 7
[[1, 2], [1, 6], [2, 3], [2, 5], [3, 6], [4, 7], [6, 7
[[1, 2], [1, 6], [2, 3], [2, 5], [3, 7], [4, 6], [6, 7
[[1, 2], [1, 6], [2, 3], [2, 6], [3, 4], [5, 7], [6, 7
[[1, 2], [1, 6], [2, 3], [2, 6], [3, 5], [4, 7], [6, 7
[[1, 2], [1, 6], [2, 3], [2, 6], [3, 7], [4, 6], [5, 7
[[1, 2], [1, 6], [2, 3], [2, 6], [3, 7], [4, 7], [5, 6
[[1, 2], [1, 6], [2, 3], [2, 7], [3, 4], [5, 6], [6, 7
[[1, 2], [1, 6], [2, 3], [2, 7], [3, 5], [4, 6], [6, 7

[[1, 2], [1, 6], [2, 3], [2, 7], [3, 6], [4, 6], [5, 7
[[1, 2], [1, 6], [2, 3], [2, 7], [3, 6], [4, 7], [5, 6
[[1, 2], [1, 6], [2, 3], [2, 7], [3, 7], [4, 6], [5, 6
[[1, 2], [1, 7], [2, 3], [2, 4], [3, 6], [5, 6], [6, 7
[[1, 2], [1, 7], [2, 3], [2, 5], [3, 6], [4, 6], [6, 7
[[1, 2], [1, 7], [2, 3], [2, 6], [3, 4], [5, 6], [6, 7
[[1, 2], [1, 7], [2, 3], [2, 6], [3, 5], [4, 6], [6, 7
[[1, 2], [1, 7], [2, 3], [2, 6], [3, 6], [4, 6], [5, 7
[[1, 2], [1, 7], [2, 3], [2, 6], [3, 6], [4, 7], [5, 6
[[1, 2], [1, 7], [2, 3], [2, 6], [3, 7], [4, 6], [5, 6
[[1, 2], [1, 7], [2, 3], [2, 7], [3, 6], [4, 6], [5, 6
[[1, 3], [1, 4], [2, 3], [2, 6], [2, 7], [5, 6], [6, 7
[[1, 3], [1, 5], [2, 3], [2, 6], [2, 7], [4, 6], [6, 7
[[1, 3], [1, 6], [2, 3], [2, 4], [2, 6], [5, 7], [6, 7
[[1, 3], [1, 6], [2, 3], [2, 4], [2, 7], [5, 6], [6, 7
[[1, 3], [1, 6], [2, 3], [2, 5], [2, 6], [4, 7], [6, 7
[[1, 3], [1, 6], [2, 3], [2, 5], [2, 7], [4, 6], [6, 7
[[1, 3], [1, 6], [2, 3], [2, 6], [2, 7], [4, 6], [5, 7
[[1, 3], [1, 6], [2, 3], [2, 6], [2, 7], [4, 7], [5, 6
[[1, 3], [1, 7], [2, 3], [2, 4], [2, 6], [5, 6], [6, 7
[[1, 3], [1, 7], [2, 3], [2, 5], [2, 6], [4, 6], [6, 7
[[1, 3], [1, 7], [2, 3], [2, 6], [2, 7], [4, 6], [5, 6
[[1, 4], [1, 5], [2, 3], [2, 6], [2, 7], [3, 6], [6, 7
[[1, 4], [1, 6], [2, 3], [2, 5], [2, 6], [3, 7], [6, 7
[[1, 4], [1, 6], [2, 3], [2, 5], [2, 7], [3, 6], [6, 7
[[1, 4], [1, 6], [2, 3], [2, 6], [2, 7], [3, 5], [6, 7
[[1, 4], [1, 6], [2, 3], [2, 6], [2, 7], [3, 6], [5, 7
[[1, 4], [1, 6], [2, 3], [2, 6], [2, 7], [3, 7], [5, 6
[[1, 4], [1, 7], [2, 3], [2, 5], [2, 6], [3, 6], [6, 7
[[1, 4], [1, 7], [2, 3], [2, 6], [2, 7], [3, 6], [5, 6
[[1, 5], [1, 6], [2, 3], [2, 4], [2, 6], [3, 7], [6, 7
[[1, 5], [1, 6], [2, 3], [2, 4], [2, 7], [3, 6], [6, 7
[[1, 5], [1, 6], [2, 3], [2, 6], [2, 7], [3, 4], [6, 7
[[1, 5], [1, 6], [2, 3], [2, 6], [2, 7], [3, 6], [4, 7
[[1, 5], [1, 6], [2, 3], [2, 6], [2, 7], [3, 7], [4, 6
[[1, 5], [1, 7], [2, 3], [2, 4], [2, 6], [3, 6], [6, 7
[[1, 5], [1, 7], [2, 3], [2, 6], [2, 7], [3, 6], [4, 6
[[1, 6], [1, 7], [2, 3], [2, 4], [2, 5], [3, 6], [6, 7
[[1, 6], [1, 7], [2, 3], [2, 4], [2, 6], [3, 5], [6, 7
[[1, 6], [1, 7], [2, 3], [2, 4], [2, 6], [3, 6], [5, 7
[[1, 6], [1, 7], [2, 3], [2, 4], [2, 6], [3, 7], [5, 6
[[1, 6], [1, 7], [2, 3], [2, 4], [2, 7], [3, 6], [5, 6
[[1, 6], [1, 7], [2, 3], [2, 5], [2, 6], [3, 4], [6, 7
[[1, 6], [1, 7], [2, 3], [2, 5], [2, 6], [3, 6], [4, 7
[[1, 6], [1, 7], [2, 3], [2, 5], [2, 6], [3, 7], [4, 6
[[1, 6], [1, 7], [2, 3], [2, 5], [2, 7], [3, 6], [4, 6
[[1, 6], [1, 7], [2, 3], [2, 6], [2, 7], [3, 4], [5, 6
[[1, 6], [1, 7], [2, 3], [2, 6], [2, 7], [3, 5], [4, 6

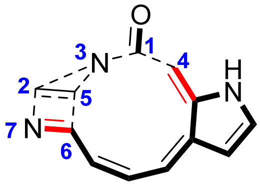

C-s12

[[1, 2], [1, 3], [2, 3], [2, 5], [3, 4], [5, 6], [5, 7
[[1, 2], [1, 3], [2, 3], [2, 5], [3, 5], [4, 6], [5, 7
[[1, 2], [1, 3], [2, 3], [2, 5], [3, 5], [4, 7], [5, 6
[[1, 2], [1, 3], [2, 3], [2, 5], [3, 6], [4, 5], [5, 7
[[1, 2], [1, 3], [2, 3], [2, 5], [3, 7], [4, 5], [5, 6
[[1, 2], [1, 3], [2, 4], [2, 5], [3, 5], [3, 6], [5, 7
[[1, 2], [1, 3], [2, 4], [2, 5], [3, 5], [3, 7], [5, 6
[[1, 2], [1, 3], [2, 5], [2, 6], [3, 4], [3, 5], [5, 7
[[1, 2], [1, 3], [2, 5], [2, 6], [3, 5], [3, 7], [4, 5
[[1, 2], [1, 3], [2, 5], [2, 7], [3, 4], [3, 5], [5, 6
[[1, 2], [1, 3], [2, 5], [2, 7], [3, 5], [3, 6], [4, 5
[[1, 2], [1, 4], [2, 3], [2, 5], [3, 5], [3, 6], [5, 7
[[1, 2], [1, 4], [2, 3], [2, 5], [3, 5], [3, 7], [5, 6
[[1, 2], [1, 5], [2, 3], [2, 5], [3, 4], [3, 6], [5, 7
[[1, 2], [1, 5], [2, 3], [2, 5], [3, 4], [3, 7], [5, 6
[[1, 2], [1, 5], [2, 3], [2, 5], [3, 5], [3, 6], [4, 7
[[1, 2], [1, 5], [2, 3], [2, 5], [3, 5], [3, 7], [4, 6

[[1, 2], [1, 5], [2, 3], [2, 5], [3, 6], [3, 7], [4, 5
[[1, 2], [1, 5], [2, 4], [2, 5], [3, 5], [3, 6], [3, 7
[[1, 2], [1, 5], [2, 5], [2, 6], [3, 4], [3, 5], [3, 7
[[1, 2], [1, 5], [2, 5], [2, 7], [3, 4], [3, 5], [3, 6
[[1, 2], [1, 6], [2, 3], [2, 5], [3, 4], [3, 5], [5, 7
[[1, 2], [1, 6], [2, 3], [2, 5], [3, 5], [3, 7], [4, 5
[[1, 2], [1, 7], [2, 3], [2, 5], [3, 4], [3, 5], [5, 6
[[1, 2], [1, 7], [2, 3], [2, 5], [3, 5], [3, 6], [4, 5
[[1, 3], [1, 4], [2, 3], [2, 5], [2, 6], [3, 5], [5, 7
[[1, 3], [1, 4], [2, 3], [2, 5], [2, 7], [3, 5], [5, 6
[[1, 3], [1, 5], [2, 3], [2, 4], [2, 5], [3, 6], [5, 7
[[1, 3], [1, 5], [2, 3], [2, 4], [2, 5], [3, 7], [5, 6
[[1, 3], [1, 5], [2, 3], [2, 5], [2, 6], [3, 4], [5, 7
[[1, 3], [1, 5], [2, 3], [2, 5], [2, 6], [3, 5], [4, 7
[[1, 3], [1, 5], [2, 3], [2, 5], [2, 6], [3, 7], [4, 5
[[1, 3], [1, 5], [2, 3], [2, 5], [2, 7], [3, 4], [5, 6
[[1, 3], [1, 5], [2, 3], [2, 5], [2, 7], [3, 5], [4, 6
[[1, 3], [1, 5], [2, 3], [2, 5], [2, 7], [3, 6], [4, 5
[[1, 3], [1, 5], [2, 4], [2, 5], [2, 6], [3, 5], [3, 7
[[1, 3], [1, 5], [2, 4], [2, 5], [2, 7], [3, 5], [3, 6
[[1, 3], [1, 5], [2, 5], [2, 6], [2, 7], [3, 4], [3, 5
[[1, 3], [1, 6], [2, 3], [2, 4], [2, 5], [3, 5], [5, 7
[[1, 3], [1, 6], [2, 3], [2, 5], [2, 7], [3, 5], [4, 5
[[1, 3], [1, 7], [2, 3], [2, 4], [2, 5], [3, 5], [5, 6
[[1, 3], [1, 7], [2, 3], [2, 5], [2, 6], [3, 5], [4, 5
[[1, 4], [1, 5], [2, 3], [2, 5], [2, 6], [3, 5], [3, 7
[[1, 4], [1, 5], [2, 3], [2, 5], [2, 7], [3, 5], [3, 6
[[1, 5], [1, 6], [2, 3], [2, 4], [2, 5], [3, 5], [3, 7
[[1, 5], [1, 6], [2, 3], [2, 5], [2, 7], [3, 4], [3, 5
[[1, 5], [1, 7], [2, 3], [2, 4], [2, 5], [3, 5], [3, 6
[[1, 5], [1, 7], [2, 3], [2, 5], [2, 6], [3, 4], [3, 5]]]

4. The structures converted from the data groups

The 150 unique structures assembled by self-created program code in Python before the inflection pointing in the most important trend of the total energy of mechanic force field were shown below.

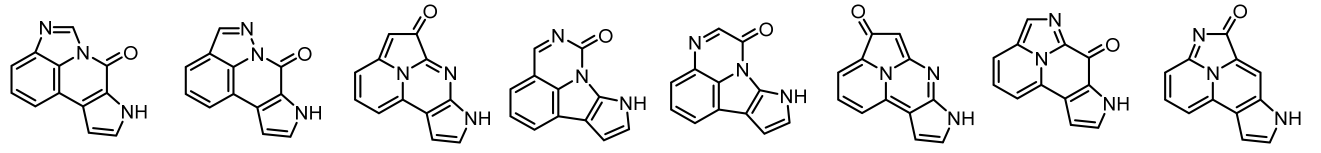

G-64 G-61 H-41 G-77 G-72 F-36 H-89 H11-99

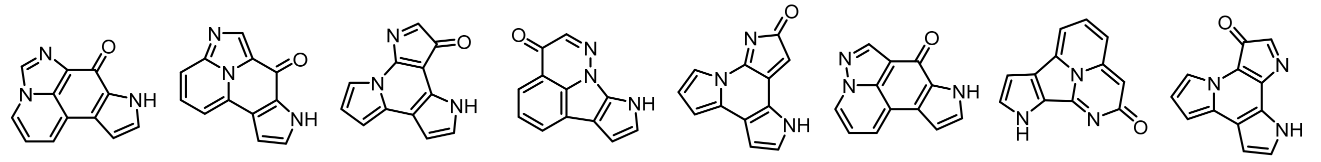

A-205 H11-144 A-2 G-13 B-15 A-43 H-58 A-7

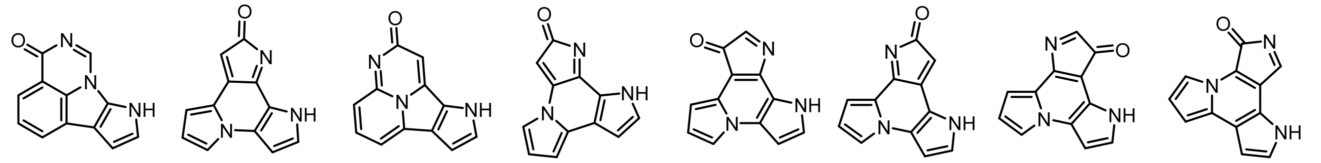

G-32 o-249 H11-107 B-36 n-112 o-246 n-116 A-77

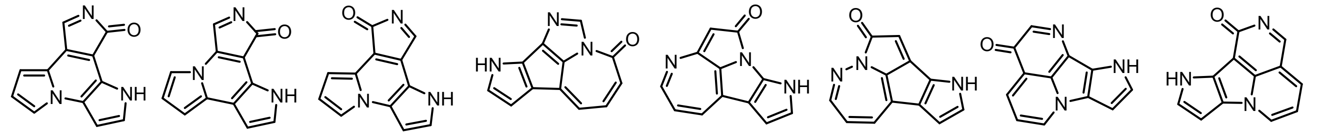

n-188 A-79 n-187 G1-79 C-43 C-39 n-123 n-198

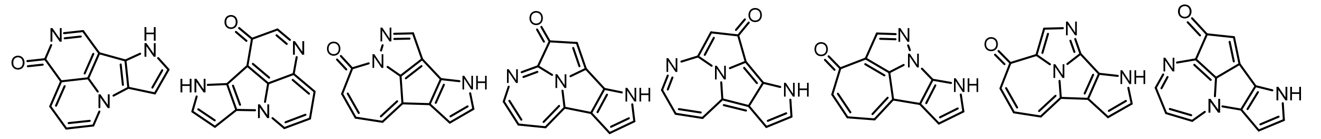

n-210 n-113 G1-55 A2-61 D-16 G1-38 H1-75 I-65

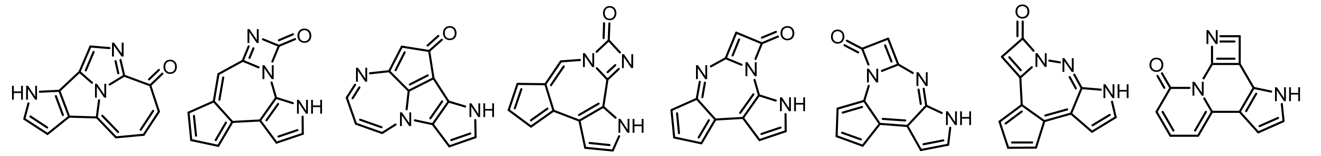

H1-51 H-148 I-66 H-149 H11-68 F-44 F-39 G1-108

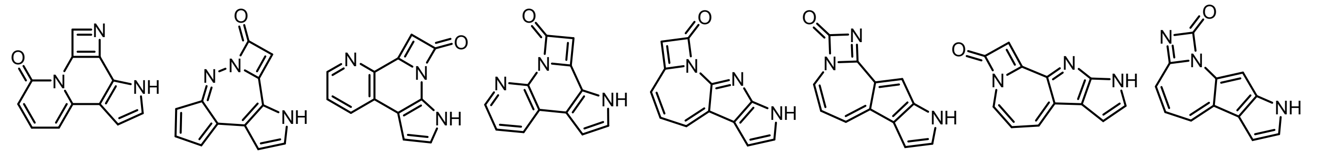

G1-80 H11-60 C-42 C-44 H-52 B-138 B-40 H11-2

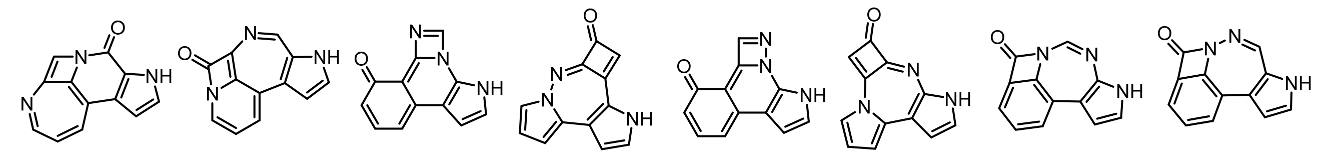

C-66 A-159 G1-97 B-13 G1-60 B-30 G-19 G-18

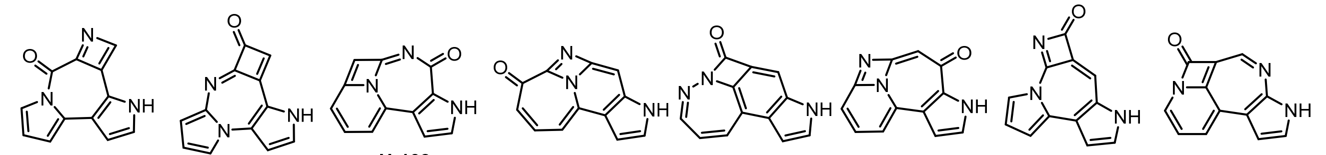

B-190 o-81 H-103 H1-53 C-17 H11-156 B-128 A-50

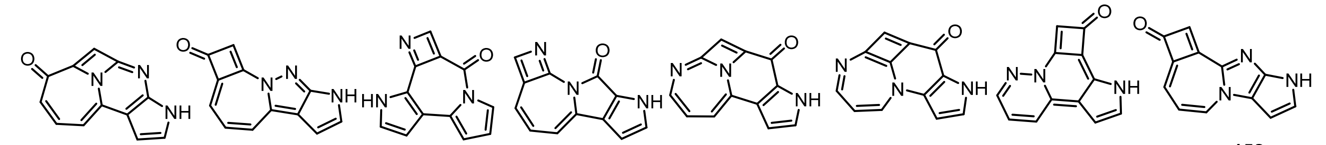

H1-139 F-34 B-177 H-96 A2-65 I-85 D-15 o-152

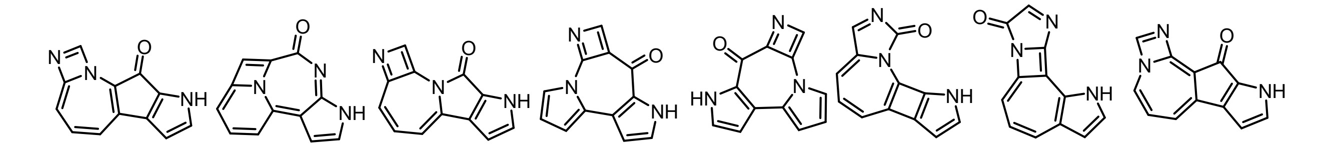

G-37 F-32 H11-82 B-175 B-194 G-82 x4-8 A-193

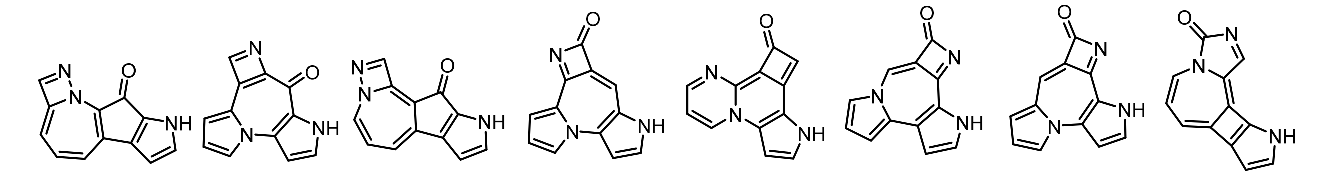

G-36 n-130 A-100 o-212 I-62 B-212 o-220 A-93

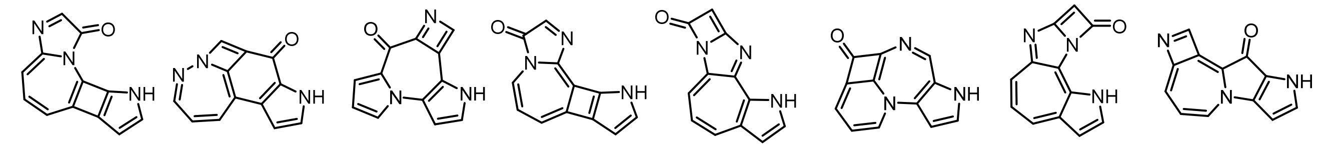

G-74 C-13 o-12 A-29 q11-33 n-168 s11-45 n-129

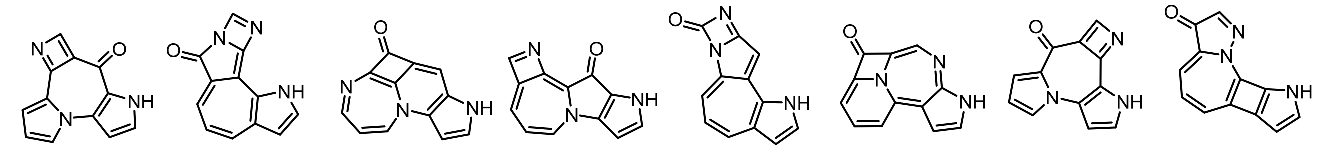

o-177 x4-58 I-37 n-136 q-65 F-11 o-120 G-14

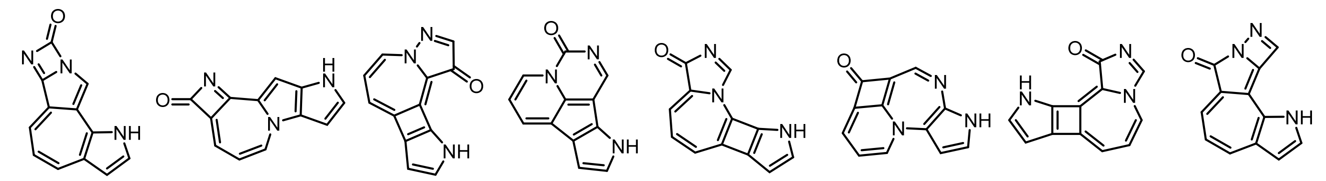

q-66 o-165 A-5 A-91 G-33 n-169 A-214 x4-52

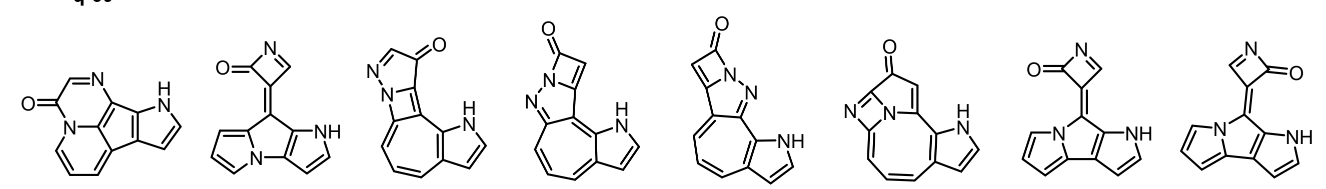

A-31 n-192 x4-2 s11-43 q11-37 q-6 A-137 A-53

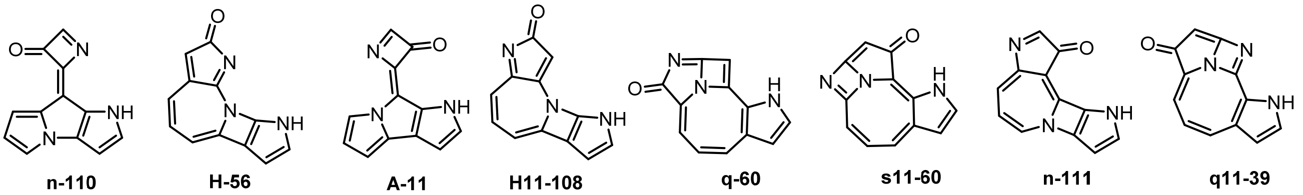

n-110 H-56 A-11 H11-108 q-60 s11-60 n-111 q11-39

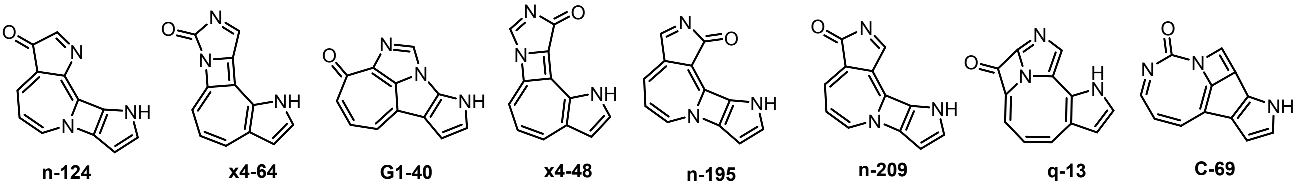

n-124 x4-64 G1-40 x4-48 n-195 n-209 q-13 C-69

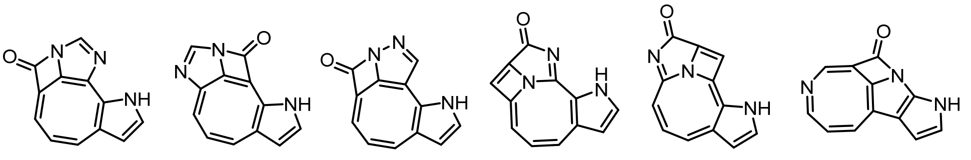

x4-60 x4-22 x4-50 q11-5 s11-29 C-21

5. Calculated detail for DFT-GIAO

The quantum chemical calculations were carried out in the serial programs including Gaussian 16 software in Supercomputing Center in Pilot National Laboratory for Marine Science and Technology (Qingdao) [33]. The procedures were described in the previous paper [35]. In brief, the input geometries were built in the Chemdraw Pro 14.1 software with an MM2 force field. The lowest energy conformers within 10 kcal/mol were subjected to further DFT calculations at the B3LYP/6-31G* level in the gas phase, and all minima displayed no imaginary frequencies by vibrational frequency analysis at the same level. Thermal corrections to Gibbs energies were obtained from frequency calculations at 298 K. The population of each conformer was calculated by Boltzmann distribution based on Gibbs free energy with the Shermo [34]. GIAO calculations of NMR shielding constants with spin-spin interactions were accomplished for all stable conformers using the DFT method at the mPW1PW91/6-31+G** level in chloroform and DMSO. The shielding constants (including C and H) obtained were directly statistically analyzed with experimental chemical shifts.

Table S1. The calculated ^{13}C NMR data as well as the calculated chemical shifts after linear scaling (mscalcd.) of the 150 structures.

NO	EXP	G-64			G-61			H-41			G-77			G-72		
		calcd.	mscalcd.	Δ	calcd.	mscalcd.	Δ	calcd.	mscalcd.	Δ	calcd.	mscalcd.	Δ	calcd.	mscalcd.	Δ
1C	168.800	151.949	153.390	-15.410	151.781	154.569	-14.231	173.777	152.993	-15.807	170.837	160.005	-8.795	155.259	153.046	-15.754
2C	133.700	142.145	142.797	9.097	144.890	146.866	13.166	152.215	141.119	7.419	152.364	145.803	12.103	151.076	149.156	15.456
3C	133.300	142.014	142.655	9.355	135.551	136.425	3.125	145.738	137.552	4.252	139.388	135.827	2.527	133.673	132.973	-0.327
4C	132.400	130.789	130.527	-1.873	128.905	128.996	-3.404	142.288	135.652	3.252	134.948	132.414	0.014	131.262	130.731	-1.669
5C	128.600	129.785	129.442	0.842	128.389	128.419	-0.181	138.867	133.768	5.168	129.559	128.271	-0.329	129.276	128.884	0.284
6C	125.200	129.603	129.245	4.045	125.858	125.590	0.390	135.714	132.032	6.832	125.207	124.925	-0.275	125.903	125.747	0.547
7C	124.400	125.672	124.998	0.598	125.579	125.278	0.878	130.027	128.900	4.500	124.490	124.374	-0.026	125.900	125.745	1.345
8C	122.500	123.792	122.966	0.466	124.900	124.519	2.019	112.243	119.106	-3.394	122.377	122.749	0.249	124.943	124.855	2.355
9C	122.400	121.468	120.455	-1.945	124.749	124.350	1.950	108.885	117.257	-5.143	119.787	120.758	-1.642	123.681	123.681	1.281
10C	121.000	121.205	120.171	-0.829	123.587	123.051	2.051	105.402	115.339	-5.661	114.902	117.003	-3.997	120.972	121.162	0.162
11C	116.200	116.723	115.328	-0.872	115.963	114.528	-1.672	102.439	113.707	-2.493	113.356	115.814	-0.386	115.191	115.786	-0.414
12C	105.100	104.091	101.679	-3.421	103.827	100.961	-4.139	88.639	106.107	1.007	100.228	105.721	0.621	100.140	101.790	-3.310
R2		0.8244			0.8306			0.8014			0.9017			0.799		
MAE		4.06			3.93			5.41			2.58			3.58		
TAD		48.75			47.20			64.93			30.97			42.90		

F-36			H-89			H11-99			A-205			H11-144			A-2		
176.132	152.956	-15.844	159.924	162.575	-6.225	173.804	156.597	-12.203	168.645	168.202	-0.598	162.027	157.399	-11.401	194.857	157.752	-11.048
157.428	142.945	9.245	138.382	139.019	5.319	155.839	145.135	11.435	133.684	133.109	-0.591	146.572	143.275	9.575	164.178	144.045	10.345
147.205	137.474	4.174	133.620	133.811	0.511	137.705	133.566	0.266	133.315	132.738	-0.562	142.094	139.182	5.882	162.024	143.082	9.782
140.520	133.896	1.496	133.059	133.198	0.798	134.505	131.524	-0.876	133.088	132.510	0.110	132.534	130.445	-1.955	134.483	130.777	-1.623
139.920	133.575	4.975	130.688	130.605	2.005	134.376	131.442	2.842	129.548	128.957	0.357	130.907	128.958	0.358	123.437	125.842	-2.758
135.805	131.373	6.173	127.529	127.151	1.951	131.217	129.426	4.226	126.560	125.958	0.758	129.554	127.722	2.522	120.728	124.631	-0.569
123.935	125.020	0.620	127.047	126.624	2.224	129.596	128.392	3.992	125.697	125.091	0.691	127.644	125.976	1.576	118.819	123.778	-0.622
122.997	124.518	2.018	125.601	125.043	2.543	122.969	124.164	1.664	124.149	123.537	1.037	127.560	125.899	3.399	115.857	122.455	-0.045
114.051	119.730	-2.670	122.607	121.769	-0.631	107.970	114.595	-7.805	123.498	122.884	0.484	123.513	122.201	-0.199	108.523	119.178	-3.222
104.854	114.808	-6.192	118.705	117.502	-3.498	107.226	114.120	-6.880	121.236	120.613	-0.387	115.688	115.050	-5.950	100.930	115.786	-5.214
101.286	112.898	-3.302	116.289	114.860	-1.340	105.104	112.766	-3.434	117.167	116.529	0.329	113.450	113.004	-3.196	97.168	114.105	-2.095
85.477	104.437	-0.663	103.986	101.407	-3.693	103.642	111.834	6.734	104.141	103.453	-1.647	104.213	104.563	-0.537	92.911	112.203	7.103
0.8109			0.9546			0.8068			0.9974			0.8714			0.8314		
4.78			2.56			5.20			0.63			3.88			4.54		
57.37			30.74			62.36			7.55			46.55			54.43		
**G-13**			**B-15**			**A-43**			**H-58**			**A-7**			**G-32**		
172.914	163.688	-5.112	192.999	159.113	-9.687	168.976	163.475	-5.325	170.844	157.858	-10.942	185.475	157.500	-11.300	170.668	162.850	-5.950
147.028	142.685	8.985	175.831	150.325	16.625	144.409	141.559	7.859	145.388	139.433	5.733	165.670	146.858	13.158	146.259	142.473	8.773
136.100	133.818	0.518	139.234	131.591	-1.709	138.201	136.021	2.721	142.105	137.057	3.757	149.524	138.183	4.883	139.438	136.779	3.479
135.682	133.478	1.078	131.755	127.762	-4.638	132.994	131.376	-1.024	135.372	132.183	-0.217	136.448	131.158	-1.242	133.673	131.966	-0.434
126.958	126.400	-2.200	130.408	127.073	-1.527	129.788	128.516	-0.084	134.278	131.391	2.791	130.680	128.058	-0.542	127.555	126.859	-1.741
126.734	126.218	1.018	122.872	123.215	-1.985	126.604	125.675	0.475	129.789	128.142	2.942	130.216	127.809	2.609	125.457	125.108	-0.092
123.988	123.990	-0.410	122.383	122.965	-1.435	125.416	124.616	0.216	129.673	128.058	3.658	122.188	123.496	-0.904	125.362	125.028	0.628
122.576	122.844	0.344	120.027	121.759	-0.741	125.379	124.583	2.083	129.294	127.784	5.284	114.848	119.552	-2.948	122.374	122.534	0.034
120.005	120.758	-1.642	117.184	120.303	-2.097	121.740	121.336	-1.064	122.424	122.811	0.411	114.802	119.527	-2.873	119.570	120.193	-2.207
119.098	120.022	-0.978	117.090	120.255	-0.745	116.598	116.749	-4.251	111.125	114.633	-6.367	110.441	117.184	-3.816	118.421	119.234	-1.766
112.503	114.671	-1.529	106.742	114.958	-1.242	113.553	114.033	-2.167	102.721	108.550	-7.650	102.523	112.930	-3.270	115.012	116.388	0.188
100.705	105.098	-0.002	105.318	114.229	9.129	104.155	105.649	0.549	98.740	105.669	0.569	99.564	111.340	6.240	100.437	104.221	-0.879
0.9526			0.8057			0.9498			0.8724			0.8365			0.9461		
1.98			4.30			2.32			4.19			4.48			2.18		
23.82			51.56			27.82			50.32			53.79			26.17		
**o-249**			**H11-107**			**B-36**			**n-112**			**o-246**			**n-116**		
195.181	160.621	-8.179	171.050	157.821	-10.979	193.001	160.248	-8.552	194.005	157.935	-10.865	195.795	159.012	-9.788	194.683	159.259	-9.541
169.914	147.211	13.511	149.320	141.865	8.165	166.260	145.778	12.078	166.710	145.257	11.557	176.515	149.487	15.787	165.152	145.190	11.490
145.653	134.336	1.036	139.064	134.334	1.034	151.807	137.958	4.658	153.691	139.209	5.909	142.526	132.697	-0.603	156.734	141.180	7.880
138.946	130.777	-1.623	139.053	134.326	1.926	138.119	130.551	-1.849	135.786	130.893	-1.507	134.845	128.902	-3.498	127.873	127.431	-4.969
136.234	129.337	0.737	137.044	132.850	4.250	133.094	127.832	-0.768	134.175	130.144	1.544	128.967	125.999	-2.601	124.052	125.610	-2.990
126.838	124.351	-0.849	130.826	128.285	3.085	127.849	124.994	-0.206	127.488	127.038	1.838	127.825	125.435	0.235	122.129	124.694	-0.506
124.818	123.279	-1.121	129.130	127.039	2.639	127.422	124.763	0.363	116.677	122.016	-2.384	126.722	124.890	0.490	120.962	124.138	-0.262
122.157	121.867	-0.633	127.439	125.797	3.297	119.058	120.237	-2.263	115.604	121.518	-0.982	124.762	123.921	1.421	117.541	122.509	0.009
114.668	117.892	-4.508	119.318	119.834	-2.566	115.878	118.517	-3.883	110.898	119.332	-3.068	115.360	119.277	-3.123	115.816	121.687	-0.713
113.343	117.189	-3.811	108.951	112.222	-8.778	113.986	117.493	-3.507	101.350	114.897	-6.103	113.699	118.456	-2.544	105.422	116.735	-4.265
112.391	116.684	0.484	108.891	112.178	-4.022	107.273	113.860	-2.340	97.143	112.943	-3.257	106.152	114.728	-1.472	98.524	113.449	-2.751

99.954	110.084	4.984	101.807	106.976	1.876	102.702	111.387	6.287	95.978	112.402	7.302	98.198	110.799	5.699	94.989	111.765	6.665
0.8756			0.8658			0.873			0.8378			0.8356			0.8465		
3.46			4.38			3.90			4.69			3.94			4.34		
41.48			52.62			46.75			56.32			47.26			52.04		
**A-77**			**n-188**			**A-79**			**n-187**			**G1-79**			**C-43**		
186.081	154.511	-14.289	191.813	154.808	-13.992	190.887	155.802	-12.998	191.868	155.724	-13.076	162.597	156.139	-12.661	166.208	146.345	-22.455
182.430	152.806	19.106	186.645	152.591	18.891	181.706	151.629	17.929	183.837	152.176	18.476	144.566	139.158	5.458	162.732	144.497	10.797
132.855	129.650	-3.650	129.709	128.165	-5.135	135.585	130.667	-2.633	130.449	128.589	-4.711	143.827	138.462	5.162	162.605	144.429	11.129
130.941	128.756	-3.644	128.928	127.830	-4.570	132.778	129.392	-3.008	128.816	127.868	-4.532	138.523	133.466	1.066	141.578	133.247	0.847
124.781	125.878	-2.722	127.730	127.316	-1.284	129.955	128.109	-0.491	128.519	127.737	-0.863	138.124	133.091	4.491	133.798	129.110	0.510
123.972	125.500	0.300	126.929	126.973	1.773	124.014	125.408	0.208	126.105	126.670	1.470	137.049	132.078	6.878	131.100	127.675	2.475
120.750	123.995	-0.405	117.346	122.861	-1.539	122.618	124.774	0.374	116.706	122.518	-1.882	132.181	127.494	3.094	124.299	124.058	-0.342
117.398	122.430	-0.070	116.556	122.523	0.023	116.496	121.991	-0.509	115.545	122.005	-0.495	125.224	120.941	-1.559	122.831	123.278	0.778
116.824	122.161	-0.239	115.366	122.012	-0.388	110.630	119.325	-3.075	113.772	121.221	-1.179	123.289	119.119	-3.281	120.735	122.163	-0.237
112.003	119.910	-1.090	109.782	119.616	-1.384	107.963	118.113	-2.887	112.651	120.726	-0.274	120.130	116.144	-4.856	117.937	120.675	-0.325
101.739	115.115	-1.085	99.616	115.255	-0.945	101.727	115.279	-0.921	99.351	114.850	-1.350	119.295	115.358	-0.842	100.078	111.177	-5.023
96.948	112.877	7.777	95.731	113.589	8.489	96.952	113.109	8.009	96.171	113.445	8.345	105.216	102.098	-3.002	92.148	106.960	1.860
0.7376			0.7314			0.7677			0.7501			0.8656			0.6923		
4.53			4.87			4.42			4.72			4.36			4.73		
54.38			58.41			53.04			56.65			52.35			56.78		
**C-39**			**n-123**			**n-198**			**n-210**			**n-113**			**G1-55**		
164.378	157.626	-11.174	173.487	162.006	-6.794	169.595	151.978	-16.822	172.716	155.205	-13.595	169.285	151.875	-16.925	161.533	155.553	-13.247
145.725	140.469	6.769	146.503	141.210	7.510	162.296	147.726	14.026	157.085	145.373	11.673	165.295	149.430	15.730	148.339	143.162	9.462
142.049	137.088	3.788	135.879	133.022	-0.278	138.758	134.016	0.716	142.426	136.153	2.853	135.207	130.995	-2.305	141.086	136.351	3.051
139.201	134.469	2.069	134.152	131.691	-0.709	135.688	132.227	-0.173	135.337	131.694	-0.706	133.259	129.802	-2.598	137.346	132.839	0.439
136.685	132.154	3.554	129.952	128.454	-0.146	133.278	130.823	2.223	134.461	131.143	2.543	133.079	129.692	1.092	137.341	132.834	4.234
135.292	130.873	5.673	129.075	127.778	2.578	129.946	128.883	3.683	128.794	127.578	2.378	132.838	129.544	4.344	136.942	132.459	7.259
128.912	125.005	0.605	128.254	127.145	2.745	126.757	127.025	2.625	127.149	126.544	2.144	128.494	126.882	2.482	125.879	122.070	-2.330
125.383	121.759	-0.741	125.187	124.782	2.282	121.645	124.047	1.547	121.478	122.977	0.477	127.473	126.257	3.757	124.858	121.111	-1.389
122.864	119.442	-2.958	120.997	121.552	-0.848	117.283	121.506	-0.894	120.543	122.389	-0.011	122.566	123.250	0.850	123.170	119.526	-2.874
122.793	119.377	-1.623	116.581	118.149	-2.851	113.756	119.452	-1.548	117.788	120.656	-0.344	114.763	118.469	-2.531	122.505	118.902	-2.098
116.370	113.469	-2.731	115.327	117.183	0.983	95.062	108.563	-7.637	98.692	108.644	-7.556	104.632	112.262	-3.938	120.391	116.916	0.716
103.706	101.820	-3.280	93.815	100.603	-4.497	92.879	107.291	2.191	93.329	105.271	0.171	93.128	105.214	0.114	104.446	101.942	-3.158
0.8958			0.9399			0.7738			0.841			0.7602			0.8521		
3.75			2.68			4.51			3.70			4.72			4.19		
44.97			32.22			54.08			44.45			56.66			50.26		
**A2-61**			**D-16**			**G1-38**			**H1-75**			**l-65**			**H1-51**		
178.722	158.157	-10.643	177.659	158.302	-10.498	179.579	162.537	-6.263	176.566	163.604	-5.196	198.437	151.701	-17.099	173.248	162.427	-6.373
150.688	140.325	6.625	154.654	142.996	9.296	146.392	137.119	3.419	143.658	136.125	2.425	178.804	144.264	10.564	147.317	140.542	6.842
139.446	133.174	-0.126	152.895	141.826	8.526	145.092	136.123	2.823	138.87	132.127	-1.173	149.739	133.254	-0.046	138.683	133.254	-0.046
138.503	132.574	0.174	134.718	129.733	-2.667	142.614	134.225	1.825	138.145	131.522	-0.878	147.716	132.488	0.088	137.409	132.179	-0.221
135.602	130.728	2.128	133.571	128.970	0.370	138.211	130.853	2.253	137.203	130.736	2.136	147.324	132.339	3.739	135.167	130.287	1.687
134.948	130.312	5.112	129.390	126.188	0.988	136.987	129.915	4.715	135.49	129.305	4.105	143.44	130.868	5.668	131.006	126.775	1.575

132.048	128.468	4.068	125.633	123.689	-0.711	132.144	126.206	1.806	133.957	128.025	3.625	140.43	129.728	5.328	128.763	124.882	0.482
129.638	126.935	4.435	124.036	122.626	0.126	126.234	121.680	-0.820	129.894	124.632	2.132	132.482	126.717	4.217	128.252	124.451	1.951
126.269	124.792	2.392	122.774	121.787	-0.613	126.100	121.577	-0.823	128.795	123.715	1.315	121.968	122.734	0.334	126.78	123.208	0.808
108.679	113.603	-7.397	119.185	119.399	-1.601	118.499	115.755	-5.245	125.776	121.194	0.194	98.832	113.971	-7.029	125.524	122.148	1.148
101.600	109.100	-7.100	108.010	111.964	-4.236	115.609	113.542	-2.658	112.115	109.787	-6.413	91.163	111.066	-5.134	111.712	110.491	-5.709
95.910	105.480	0.380	99.141	106.064	0.964	103.200	104.038	-1.062	103.695	102.756	-2.344	73.91	104.530	-0.570	102.859	103.019	-2.081
0.8681			0.8819			0.9489			0.9518			0.7746			0.9466		
4.22			3.38			2.81			2.66			4.98			2.41		
50.58			40.60			33.71			31.94			59.82			28.92		
**H-148**			**l-66**			**H-149**			**H11-68**			**F-44**			**F-39**		
144.514	143.243	-25.557	191.375	157.889	-10.911	171.627	157.226	-11.574	200.656	152.969	-15.831	166.725	143.154	-25.646	177.821	155.195	-13.605
143.163	142.032	8.332	161.03	142.307	8.607	160.462	148.742	15.042	161.008	138.815	5.115	163.413	141.707	8.007	166.802	148.718	15.018
139.695	138.924	5.624	160.749	142.163	8.863	142.841	135.352	2.052	153.804	136.243	2.943	162.067	141.120	7.820	141.655	133.937	0.637
133.966	133.789	1.389	138.943	130.965	-1.435	140.737	133.753	1.353	148.57	134.374	1.974	161.446	140.848	8.448	137.134	131.279	-1.121
133.642	133.498	4.898	135.636	129.267	0.667	129.989	125.586	-3.014	145.956	133.441	4.841	142.83	132.719	4.119	133.285	129.017	0.417
131.722	131.777	6.577	134.619	128.745	3.545	127.538	123.723	-1.477	137.295	130.349	5.149	136.187	129.818	4.618	130.279	127.250	2.050
127.672	128.147	3.747	124.129	123.358	-1.042	126.829	123.184	-1.216	127.733	126.936	2.536	123.611	124.326	-0.074	127.157	125.415	1.015
124.834	125.604	3.104	122.776	122.663	0.163	126.149	122.668	0.168	126.133	126.364	3.864	113.818	120.049	-2.451	123.855	123.474	0.974
124.775	125.551	3.151	113.957	118.135	-4.265	124.948	121.755	-0.645	109.769	120.523	-1.877	113.06	119.718	-2.682	123.24	123.112	0.712
111.907	114.017	-6.983	110.88	116.555	-4.445	121.207	118.912	-2.088	104.233	118.546	-2.454	105.163	116.270	-4.730	112.659	116.893	-4.107
108.25	110.739	-5.461	106.202	114.153	-2.047	114.447	113.775	-2.425	103.087	118.137	1.937	100.854	114.388	-1.812	106.893	113.504	-2.696
103.288	106.292	1.192	93.019	107.383	2.283	108.041	108.907	3.807	43.563	96.887	-8.213	89.771	109.548	4.448	93.701	105.749	0.649
0.631			0.868			0.8408			0.8262			0.6254			0.8253		
6.33			4.02			3.74			4.73			6.24			3.58		
76.01			48.27			44.86			56.74			74.86			43.00		
**G1-108**			**G1-80**			**H11-60**			**C-42**			**C-44**			**H-52**		
206.824	161.711	-7.089	204.157	164.830	-3.970	176.501	152.857	-15.943	200.139	153.170	-15.630	166.520	144.959	-23.841	197.561	150.657	-18.143
162.555	141.547	7.847	156.589	140.029	6.329	169.800	149.079	15.379	152.808	136.599	2.899	166.417	144.911	11.211	167.221	139.953	6.253
158.051	139.495	6.195	143.534	133.222	-0.078	141.972	133.393	0.093	148.195	134.984	1.684	151.688	138.022	4.722	166.159	139.578	6.278
141.516	131.964	-0.436	136.933	129.780	-2.620	138.226	131.281	-1.119	142.283	132.914	0.514	146.039	135.380	2.980	156.245	136.080	3.680
137.424	130.100	1.500	134.866	128.702	0.102	137.242	130.726	2.126	136.937	131.043	2.443	135.796	130.590	1.990	134.035	128.245	-0.355
125.918	124.859	-0.341	131.69	127.046	1.846	130.654	127.013	1.813	133.094	129.697	4.497	134.992	130.214	5.014	133.780	128.155	2.955
121.639	122.910	-1.490	130.525	126.439	2.039	128.426	125.757	1.357	127.952	127.897	3.497	133.374	129.457	5.057	127.067	125.786	1.386
120.758	122.508	0.008	130.333	126.339	3.839	128.308	125.690	3.190	125.363	126.991	4.491	117.524	122.044	-0.456	126.105	125.447	2.947
110.937	118.035	-4.365	120.696	121.314	-1.086	118.700	120.274	-2.126	122.993	126.161	3.761	114.025	120.407	-1.993	124.627	124.925	2.525
103.273	114.544	-6.456	105.721	113.506	-7.494	116.649	119.118	-1.882	111.271	122.057	1.057	113.570	120.195	-0.805	115.922	121.854	0.854
102.226	114.067	-2.133	104.069	112.645	-3.555	107.772	114.114	-2.086	99.782	118.035	1.835	104.461	115.934	-0.266	99.450	116.043	-0.157
97.303	111.825	6.725	98.425	109.702	4.602	90.319	104.276	-0.824	31.254	94.043	-11.057	73.660	101.529	-3.571	45.167	96.892	-8.208
0.8955			0.9306			0.7933			0.8207			0.6859			0.7971		
3.72			3.13			3.99			4.45			5.16			4.48		
44.59			37.56			47.94			53.36			61.91			53.74		
**B-138**			**B-40**			**H11-2**			**C-66**			**A-159**			**G1-97**		
177.278	155.213	-13.587	172.792	147.477	-21.323	177.980	150.321	-18.479	158.864	151.879	-16.921	219.588	168.554	-0.246	183.736	150.494	-18.306

161.106	145.351	11.651	162.822	142.543	8.843	161.432	142.148	8.448	151.974	146.067	12.367	155.129	137.059	3.359	176.963	147.403	13.703
143.483	134.605	1.305	160.310	141.299	7.999	159.432	141.160	7.860	142.418	138.007	4.707	147.225	133.197	-0.103	165.730	142.275	8.975
141.692	133.513	1.113	143.971	133.213	0.813	143.326	133.206	0.806	138.734	134.899	2.499	146.726	132.953	0.553	145.059	132.838	0.438
137.648	131.047	2.447	139.278	130.891	2.291	142.791	132.941	4.341	136.913	133.363	4.763	133.618	126.549	-2.051	139.790	130.433	1.833
136.254	130.197	4.997	136.850	129.689	4.489	139.692	131.411	6.211	127.239	125.203	0.003	129.531	124.552	-0.648	127.285	124.725	-0.475
129.948	126.351	1.951	133.964	128.261	3.861	126.908	125.097	0.697	125.787	123.978	-0.422	128.453	124.025	-0.375	119.099	120.988	-3.412
125.283	123.507	1.007	128.234	125.425	2.925	122.175	122.759	0.259	125.457	123.700	1.200	124.105	121.901	-0.599	116.922	119.994	-2.506
118.319	119.260	-3.140	125.173	123.910	1.510	119.868	121.620	-0.780	122.403	121.124	-1.276	123.378	121.545	-0.855	112.341	117.903	-4.497
115.245	117.385	-3.615	107.594	115.210	-5.790	109.212	116.357	-4.643	117.328	116.843	-4.157	120.908	120.339	-0.661	111.720	117.619	-3.381
101.502	109.005	-7.195	96.756	109.847	-6.353	102.813	113.196	-3.004	112.498	112.769	-3.431	108.065	114.064	-2.136	107.705	115.786	-0.414
100.087	108.142	3.042	88.522	105.772	0.672	82.925	103.374	-1.726	104.113	105.696	0.596	97.572	108.937	3.837	102.012	113.187	8.087
0.8255			0.7153			0.7766			0.7941			0.9853			0.7156		
4.59			5.57			4.77			4.36			1.29			5.50		
55.05			66.87			57.25			52.34			15.42			66.03		
**B-13**			**G1-60**			**B-30**			**G-19**			**G-18**			**B-190**		
185.013	153.779	-15.021	182.933	153.977	-14.823	188.382	152.678	-16.122	161.988	151.229	-17.571	163.868	152.961	-15.839	191.167	166.400	-2.400
168.663	145.148	11.448	173.514	148.949	15.249	179.427	148.470	14.770	160.981	150.473	16.773	163.760	152.876	19.176	156.051	143.101	9.401
166.731	144.128	10.828	148.506	135.600	2.300	164.500	141.456	8.156	139.287	134.194	0.894	137.052	131.878	-1.422	134.928	129.086	-4.214
139.922	129.976	-2.424	144.964	133.709	1.309	138.604	129.287	-3.113	136.253	131.917	-0.483	133.597	129.162	-3.238	134.379	128.721	-3.679
134.577	127.154	-1.446	139.005	130.528	1.928	134.176	127.206	-1.394	132.397	129.024	0.424	131.214	127.288	-1.312	131.719	126.957	-1.643
130.704	125.110	-0.090	133.052	127.350	2.150	133.249	126.771	1.571	132.284	128.939	3.739	127.645	124.482	-0.718	128.715	124.963	-0.237
129.992	124.734	0.334	121.818	121.353	-3.047	119.862	120.480	-3.920	126.524	124.617	0.217	126.109	123.275	-1.125	128.387	124.746	0.346
126.603	122.945	0.445	121.011	120.923	-1.577	119.191	120.165	-2.335	124.814	123.333	0.833	125.232	122.585	0.085	128.134	124.578	2.078
119.136	119.003	-3.397	117.679	119.144	-3.256	118.942	120.048	-2.352	121.666	120.971	-1.429	124.568	122.063	-0.337	123.750	121.669	-0.731
113.319	115.932	-5.068	116.106	118.304	-2.696	112.690	117.110	-3.890	115.250	116.157	-4.843	123.491	121.217	0.217	115.716	116.339	-4.661
107.817	113.028	-3.172	105.809	112.808	-3.392	109.271	115.503	-0.697	111.453	113.307	-2.893	117.595	116.581	0.381	113.891	115.128	-1.072
107.186	112.694	7.594	102.468	111.024	5.924	107.087	114.477	9.377	106.395	109.512	4.412	108.194	109.190	4.090	109.041	111.910	6.810
0.7688			0.7858			0.7289			0.7404			0.7434			0.9203		
5.11			4.80			5.64			4.54			3.99			3.11		
61.27			57.65			67.70			54.51			47.94			37.27		
**o-81**			**H-103**			**H1-53**			**C-17**			**H11-156**			**B-128**		
189.291	153.085	-15.715	165.004	144.129	-24.671	171.082	149.432	-19.368	160.162	149.672	-19.129	175.850	147.725	-21.075	188.519	156.192	-12.608
174.125	145.717	12.017	163.201	143.309	9.609	168.974	148.249	14.549	155.541	146.206	12.506	167.984	144.054	10.354	180.540	152.252	18.552
168.613	143.039	9.739	158.949	141.374	8.074	150.251	137.742	4.442	143.109	136.882	3.582	167.139	143.660	10.360	134.004	129.272	-4.028
141.018	129.634	-2.766	149.113	136.897	4.497	136.609	130.086	-2.314	137.190	132.443	0.042	146.951	134.240	1.840	125.822	125.232	-7.168
138.192	128.261	-0.339	141.033	133.220	4.620	134.495	128.900	0.300	135.822	131.417	2.817	138.506	130.300	1.700	125.119	124.885	-3.715
138.122	128.227	3.027	132.855	129.498	4.298	134.357	128.822	3.622	132.427	128.870	3.670	137.100	129.644	4.444	125.080	124.866	-0.334
132.974	125.726	1.326	122.995	125.011	0.611	134.321	128.802	4.402	131.722	128.342	3.942	124.727	123.871	-0.529	121.165	122.932	-1.468
122.827	120.796	-1.704	115.313	121.515	-0.985	126.389	124.351	1.851	125.953	124.015	1.515	116.462	120.014	-2.486	119.120	121.922	-0.578
116.966	117.949	-4.451	109.327	118.791	-3.609	117.548	119.389	-3.011	125.448	123.636	1.236	108.383	116.245	-6.155	118.028	121.383	-1.017
113.330	116.183	-4.817	108.543	118.434	-2.566	106.735	113.321	-7.679	115.530	116.198	-4.802	106.513	115.372	-5.628	114.009	119.399	-1.601
109.994	114.562	-1.638	105.917	117.239	1.039	105.771	112.780	-3.420	109.955	112.016	-4.184	104.519	114.442	-1.758	112.308	118.559	2.359
101.561	110.465	5.365	77.288	104.210	-0.890	104.030	111.803	6.703	99.151	103.913	-1.187	103.650	114.036	8.936	108.458	116.658	11.558

0.7707			0.6657			0.6958			0.7567			0.6678			0.7126		
5.24			5.46			5.97			4.88			6.27			5.42		
62.90			65.47			71.66			58.61			75.27			64.99		
**A-50**			**H1-139**			**F-34**			**B-177**			**H-96**			**A2-65**		
207.806	162.957	-5.843	174.952	150.298	-18.502	184.501	151.269	-17.531	210.363	163.565	-5.235	175.055	150.655	-18.145	161.733	154.386	-14.414
151.303	136.705	3.005	161.007	143.080	9.380	177.907	148.025	14.325	169.306	143.866	10.166	169.483	147.635	13.935	141.420	138.410	4.710
146.659	134.548	1.248	157.790	141.415	8.115	160.306	139.366	6.066	146.424	132.887	-0.413	156.155	140.411	7.111	140.756	137.888	4.588
146.581	134.512	2.112	141.745	133.110	0.710	151.980	135.269	2.869	143.998	131.723	-0.677	148.062	136.025	3.625	137.597	135.403	3.003
138.422	130.721	2.121	140.100	132.259	3.659	137.351	128.072	-0.528	143.484	131.477	2.877	136.292	129.645	1.045	134.318	132.824	4.224
133.058	128.229	3.029	138.160	131.255	6.055	133.805	126.327	1.127	128.531	124.302	-0.898	123.165	122.530	-2.670	129.594	129.109	3.909
131.288	127.406	3.006	124.564	124.217	-0.183	127.521	123.235	-1.165	127.246	123.686	-0.714	123.058	122.472	-1.928	127.987	127.845	3.445
121.667	122.936	0.436	124.339	124.101	1.601	124.691	121.843	-0.657	120.176	120.293	-2.207	122.063	121.933	-0.567	125.158	125.620	3.120
117.466	120.985	-1.415	110.092	116.727	-5.673	121.485	120.266	-2.134	119.847	120.136	-2.264	118.050	119.758	-2.642	124.305	124.949	2.549
109.990	117.511	-3.489	104.739	113.956	-7.044	116.627	117.875	-3.125	111.424	116.094	-4.906	110.985	115.929	-5.071	109.301	113.148	-7.852
106.644	115.957	-0.243	100.833	111.934	-4.266	110.803	115.010	-1.190	109.483	115.163	-1.037	110.679	115.763	-0.437	105.979	110.535	-5.665
74.884	101.201	-3.899	99.440	111.213	6.113	94.542	107.010	1.910	99.591	110.417	5.317	101.613	110.849	5.749	97.033	103.499	-1.601
0.96			0.7296			0.7715			0.9195			0.738			0.8356		
2.49			5.94			4.39			3.06			5.24			4.92		
29.85			71.30			52.63			36.71			62.92			59.08		
**l-85**			**D-15**			**o-152**			**G-37**			**F-32**			**H11-82**		
162.866	152.623	-16.177	178.591	150.862	-17.938	186.683	152.615	-16.185	179.954	157.844	-10.956	159.992	145.283	-23.517	176.903	156.753	-12.047
148.964	141.947	8.247	173.862	148.471	14.771	175.632	147.178	13.478	155.131	141.939	8.239	154.595	142.079	8.379	161.314	146.807	13.107
145.131	139.004	5.704	151.743	137.290	3.990	158.798	138.896	5.596	153.839	141.112	7.812	151.894	140.475	7.175	147.390	137.924	4.624
144.957	138.870	6.470	137.796	130.240	-2.160	146.077	132.637	0.237	146.683	136.527	4.127	149.071	138.798	6.398	134.980	130.006	-2.394
130.357	127.659	-0.941	135.136	128.895	0.295	139.585	129.443	0.843	132.493	127.435	-1.165	140.860	133.923	5.323	132.019	128.117	-0.483
129.748	127.191	1.991	134.604	128.626	3.426	131.447	125.439	0.239	128.046	124.586	-0.614	136.646	131.420	6.220	131.273	127.641	2.441
129.113	126.704	2.304	128.224	125.401	1.001	130.569	125.007	0.607	127.503	124.238	-0.162	121.650	122.516	-1.884	126.628	124.678	0.278
124.021	122.794	0.294	126.911	124.738	2.238	128.924	124.198	1.698	121.740	120.546	-1.954	119.365	121.159	-1.341	123.893	122.933	0.433
123.110	122.094	-0.306	125.013	123.778	1.378	125.892	122.706	0.306	120.613	119.824	-2.576	113.678	117.782	-4.618	121.146	121.180	-1.220
121.980	121.226	0.226	112.153	117.277	-3.723	113.982	116.846	-4.154	120.407	119.692	-1.308	108.969	114.986	-6.014	113.925	116.573	-4.427
108.482	110.861	-5.339	99.662	110.963	-5.237	107.582	113.697	-2.503	105.392	110.072	-6.128	106.323	113.415	-2.785	104.643	110.651	-5.549
97.693	102.576	-2.524	91.875	107.027	1.927	89.677	104.888	-0.212	104.933	109.778	4.678	103.588	111.791	6.691	104.131	110.325	5.225
0.823			0.7538			0.8021			0.8664			0.6464			0.8308		
4.21			4.84			3.84			4.14			6.70			4.35		
50.52			58.08			46.06			49.72			80.34			52.23		
**B-175**			**B-194**			**G-82**			**x4-8**			**A-193**			**G-36**		
218.663	163.765	-5.035	192.778	162.464	-6.336	164.944	146.120	-22.680	157.556	152.655	-16.145	174.911	150.440	-18.360	172.398	154.472	-14.328
172.055	143.733	10.033	169.037	148.379	14.679	159.559	142.968	9.268	148.766	143.837	10.137	167.284	146.183	12.483	157.165	144.450	10.750
158.902	138.080	4.780	137.167	129.470	-3.830	152.547	138.864	5.564	145.175	140.235	6.935	154.552	139.077	5.777	144.703	136.251	2.951
137.122	128.719	-3.681	133.138	127.080	-5.320	150.979	137.946	5.546	139.001	134.041	1.641	150.757	136.959	4.559	142.052	134.507	2.107
132.024	126.528	-2.072	131.114	125.879	-2.721	150.439	137.630	9.030	136.183	131.214	2.614	136.484	128.994	0.394	136.277	130.708	2.108
122.844	122.582	-2.618	130.075	125.262	0.062	133.292	127.594	2.394	133.104	128.125	2.925	134.136	127.683	2.483	134.070	129.256	4.056
121.130	121.846	-2.554	126.654	123.233	-1.167	127.460	124.180	-0.220	128.161	123.166	-1.234	126.307	123.314	-1.086	131.271	127.414	3.014

117.577	120.319	-2.181	125.064	122.289	-0.211	120.414	120.056	-2.444	127.416	122.419	-0.081	124.424	122.263	-0.237	127.610	125.006	2.506
115.286	119.334	-3.066	123.133	121.144	-1.256	117.503	118.353	-4.047	127.038	122.040	-0.360	121.659	120.720	-1.680	116.343	117.593	-4.807
113.364	118.508	-2.492	118.499	118.394	-2.606	114.408	116.541	-4.459	125.882	120.880	-0.120	118.709	119.073	-1.927	110.275	113.601	-7.399
107.450	115.966	-0.234	115.422	116.569	0.369	107.119	112.275	-3.925	112.890	107.846	-8.354	101.478	109.457	-6.743	107.021	111.460	-4.740
103.506	114.271	9.171	110.229	113.488	8.388	105.117	111.103	6.003	112.248	107.202	2.102	101.424	109.427	4.327	103.084	108.870	3.770
0.8879			0.8479			0.668			0.801			0.7537			0.8091		
3.99			3.91			6.30			4.39			5.00			5.21		
47.92			46.94			75.58			52.65			60.06			62.54		
**n-130**			**A-100**			**o-212**			**l-62**			**B-212**			**o-220**		
193.605	164.473	-4.327	173.733	154.310	-14.490	191.614	157.019	-11.781	184.498	152.173	-16.627	185.100	155.048	-13.752	188.197	154.796	-14.004
161.646	145.150	11.450	155.091	142.554	8.854	181.500	152.031	18.331	170.436	145.565	11.865	180.028	152.401	18.701	181.811	151.665	17.965
138.437	131.118	-2.182	138.863	132.321	-0.979	134.453	128.827	-4.473	158.958	140.171	6.871	135.828	129.328	-3.972	140.889	131.601	-1.699
137.103	130.311	-2.089	136.595	130.891	-1.509	133.079	128.150	-4.250	141.608	132.019	-0.381	134.847	128.816	-3.584	134.646	128.540	-3.860
131.040	126.646	-1.954	135.779	130.376	1.776	129.697	126.482	-2.118	134.870	128.852	0.252	130.064	126.319	-2.281	133.660	128.056	-0.544
128.818	125.302	0.102	135.136	129.971	4.771	126.427	124.869	-0.331	131.666	127.347	2.147	128.982	125.755	0.555	131.997	127.241	2.041
126.106	123.663	-0.737	134.786	129.750	5.350	122.921	123.140	-1.260	130.020	126.573	2.173	127.161	124.804	0.404	126.651	124.620	0.220
123.250	121.936	-0.564	133.644	129.030	6.530	121.083	122.233	-0.267	129.096	126.139	3.639	118.844	120.463	-2.037	121.173	121.934	-0.566
123.189	121.899	-0.501	126.236	124.358	1.958	120.616	122.003	-0.397	114.279	119.177	-3.223	118.083	120.065	-2.335	113.512	118.178	-4.222
116.357	117.768	-3.232	118.317	119.365	-1.635	115.320	119.391	-1.609	102.177	113.490	-7.510	115.157	118.538	-2.462	111.926	117.400	-3.600
115.530	117.268	1.068	101.381	108.685	-7.515	109.629	116.584	0.384	97.209	111.156	-5.044	111.472	116.614	0.414	110.350	116.628	0.428
100.433	108.141	3.041	90.856	102.048	-3.052	102.205	112.923	7.823	96.790	110.959	5.859	109.245	115.452	10.352	102.719	112.886	7.786
0.9273			0.8184			0.7703			0.7583			0.7259			0.7507		
2.60			4.87			4.42			5.47			5.07			4.74		
31.25			58.42			53.02			65.59			60.85			56.93		
**A-93**			**G-74**			**C-13**			**o-12**			**A-29**			**q11-33**		
165.033	147.024	-21.776	167.627	148.450	-20.350	167.281	160.167	-8.633	194.260	160.401	-8.399	161.411	148.482	-20.318	198.908	149.142	-19.658
164.244	146.567	12.867	160.697	144.270	10.570	142.861	139.568	5.868	171.145	147.753	14.053	150.847	141.209	7.509	170.387	139.805	6.105
150.249	138.464	5.164	156.632	141.819	8.519	139.597	136.815	3.515	138.875	130.094	-3.206	147.089	138.622	5.322	157.314	135.525	2.225
139.248	132.095	-0.305	149.866	137.738	5.338	139.216	136.494	4.094	135.242	128.106	-4.294	140.051	133.776	1.376	150.087	133.158	0.758
138.127	131.446	2.846	135.696	129.192	0.592	132.370	130.719	2.119	132.027	126.347	-2.253	139.467	133.374	4.774	136.015	128.551	-0.049
135.835	130.118	4.918	133.948	128.138	2.938	128.916	127.806	2.606	131.206	125.898	0.698	136.144	131.086	5.886	133.816	127.831	2.631
134.173	129.156	4.756	130.221	125.890	1.490	126.342	125.634	1.234	130.835	125.695	1.295	132.734	128.738	4.338	132.963	127.552	3.152
120.860	121.448	-1.052	120.418	119.978	-2.522	123.112	122.910	0.410	130.540	125.533	3.033	132.048	128.266	5.766	130.767	126.833	4.333
120.253	121.096	-1.304	116.951	117.887	-4.513	120.386	120.611	-1.789	128.679	124.515	2.115	118.326	118.818	-3.582	129.894	126.547	4.147
108.275	114.161	-6.839	111.597	114.658	-6.342	112.302	113.792	-7.208	111.258	114.982	-6.018	107.994	111.705	-9.295	125.327	125.052	4.052
103.188	111.216	-4.984	109.924	113.649	-2.551	109.268	111.233	-4.967	110.252	114.432	-1.768	107.467	111.342	-4.858	108.833	119.652	3.452
102.379	110.747	5.647	107.200	112.006	6.906	105.239	107.834	2.734	101.736	109.772	4.672	102.899	108.197	3.097	30.521	94.013	-11.087
0.6741			0.701			0.9061			0.8508			0.7081			0.7506		
6.04			6.05			3.76			4.32			6.34			5.14		
72.46			72.63			45.18			51.80			76.12			61.65		
**n-168**			**s11-45**			**n-129**			**o-177**			**x4-58**			**l-37**		
187.389	155.612	-13.188	199.374	149.351	-19.449	168.610	145.945	-22.855	209.896	163.354	-5.446	159.418	154.155	-14.645	189.826	157.266	-11.534
173.124	147.641	13.941	170.264	139.786	6.086	167.696	145.494	11.794	169.973	144.179	10.479	152.559	147.392	13.692	168.586	145.357	11.657

156.976	138.617	5.317	157.347	135.541	2.241	157.182	140.308	7.008	158.057	138.456	5.156	140.162	135.169	1.869	154.670	137.554	4.254
147.585	133.369	0.969	150.601	133.324	0.924	154.622	139.045	6.645	138.563	129.093	-3.307	137.548	132.591	0.191	146.497	132.972	0.572
137.273	127.607	-0.993	135.870	128.484	-0.116	135.484	129.604	1.004	127.303	123.685	-4.915	135.051	130.129	1.529	140.552	129.639	1.039
135.051	126.365	1.165	133.995	127.868	2.668	129.291	126.549	1.349	126.246	123.177	-2.023	134.857	129.938	4.738	137.280	127.804	2.604
129.383	123.198	-1.202	132.360	127.330	2.930	124.046	123.962	-0.438	124.455	122.317	-2.083	129.630	124.784	0.384	127.635	122.396	-2.004
124.786	120.629	-1.871	130.536	126.731	4.231	121.326	122.620	0.120	124.020	122.108	-0.392	128.059	123.235	0.735	127.543	122.344	-0.156
122.264	119.220	-3.180	130.384	126.681	4.281	121.303	122.609	0.209	121.414	120.856	-1.544	125.577	120.788	-1.612	123.086	119.845	-2.555
119.016	117.405	-3.595	124.915	124.884	3.884	109.109	116.593	-4.407	117.702	119.073	-1.927	120.447	115.730	-5.270	121.643	119.036	-1.964
116.313	115.895	-0.305	108.973	119.646	3.446	99.788	111.995	-4.205	112.169	116.416	0.216	115.843	111.190	-5.010	115.861	115.794	-0.406
102.293	108.060	2.960	31.011	94.027	-11.073	93.564	108.925	3.825	100.529	110.825	5.725	113.036	108.423	3.323	94.212	103.656	-1.444
0.8281			0.7548			0.6812			0.9021			0.8041			0.8772		
4.06			5.11			5.32			3.60			4.42			3.35		
48.69			61.33			63.86			43.21			53.00			40.19		
**n-136**			**q-65**			**F-11**			**o-120**			**G-14**			**q-66**		
183.123	152.880	-15.920	152.438	145.735	-23.065	162.786	145.698	-23.102	212.523	161.943	-6.857	167.634	146.018	-22.782	144.873	149.026	-19.774
166.710	144.465	10.765	152.277	145.605	11.905	158.076	142.770	9.070	169.663	142.356	8.656	162.633	143.366	9.666	140.496	143.480	9.780
155.510	138.723	5.423	135.575	132.140	-1.160	151.290	138.550	5.250	157.220	136.670	3.370	160.871	142.432	9.132	138.970	141.547	8.247
153.198	137.538	5.138	135.551	132.120	-0.280	145.197	134.761	2.361	148.876	132.856	0.456	148.422	135.831	3.431	133.402	134.492	2.092
133.148	127.258	-1.342	134.787	131.504	2.904	144.248	134.171	5.571	141.571	129.518	0.918	148.378	135.808	7.208	130.534	130.859	2.259
131.804	126.569	1.369	132.779	129.885	4.685	143.002	133.397	8.197	141.182	129.340	4.140	131.118	126.657	1.457	129.645	129.732	4.532
126.995	124.103	-0.297	132.327	129.521	5.121	135.488	128.724	4.324	124.027	121.500	-2.900	124.170	122.973	-1.427	126.425	125.652	1.252
126.476	123.837	1.337	129.703	127.406	4.906	124.120	121.656	-0.844	117.821	118.664	-3.836	118.701	120.073	-2.427	125.442	124.407	1.907
123.565	122.345	-0.055	128.412	126.365	3.965	115.619	116.370	-6.030	114.307	117.058	-5.342	114.650	117.925	-4.475	123.139	121.489	-0.911
120.264	120.652	-0.348	121.777	121.016	0.016	114.361	115.588	-5.412	112.683	116.316	-4.684	114.541	117.868	-3.132	117.496	114.339	-6.661
96.665	108.553	-7.647	109.090	110.787	-5.413	108.903	112.194	-4.006	109.811	115.004	-1.196	104.386	112.483	-3.717	116.397	112.947	-3.253
93.083	106.717	1.617	97.588	101.514	-3.586	104.897	109.703	4.603	103.982	112.340	7.240	103.794	112.170	7.070	110.604	105.607	0.507
0.8063			0.6796			0.6576			0.8902			0.6601			0.7456		
4.27			5.58			6.56			4.13			6.33			5.10		
51.26			67.01			78.77			49.59			75.92			61.18		
**o-165**			**A-5**			**A-91**			**G-33**			**n-169**			**A-214**		
190.411	154.047	-14.753	177.703	157.223	-11.577	151.882	145.689	-23.111	172.090	149.280	-19.520	187.680	155.425	-13.375	177.683	154.101	-14.699
181.215	149.818	16.118	153.327	141.556	7.856	151.384	145.306	11.606	164.306	145.312	11.612	172.411	147.129	13.429	161.092	144.505	10.805
142.401	131.967	-1.333	140.892	133.564	0.264	147.199	142.092	8.792	151.725	138.898	5.598	154.877	137.603	4.303	148.960	137.487	4.187
141.675	131.633	-0.767	138.602	132.093	-0.307	136.349	133.758	1.358	146.497	136.233	3.833	152.396	136.255	3.855	138.920	131.680	-0.720
134.717	128.433	-0.167	137.639	131.474	2.874	131.274	129.860	1.260	143.768	134.842	6.242	137.549	128.188	-0.412	138.095	131.203	2.603
132.852	127.576	2.376	137.490	131.378	6.178	130.291	129.105	3.905	129.215	127.423	2.223	132.904	125.665	0.465	137.211	130.692	5.492
131.369	126.894	2.494	135.315	129.980	5.580	125.453	125.388	0.988	119.002	122.216	-2.184	127.235	122.585	-1.815	134.095	128.890	4.490
127.977	125.334	2.834	128.268	125.451	2.951	121.247	122.158	-0.342	116.592	120.988	-1.512	123.561	120.589	-1.911	121.527	121.620	-0.880
115.677	119.677	-2.723	119.796	120.006	-2.394	120.623	121.679	-0.721	114.867	120.108	-2.292	121.076	119.239	-3.161	113.509	116.983	-5.417
106.061	115.254	-5.746	109.087	113.123	-7.877	120.380	121.492	0.492	103.250	114.186	-6.814	117.729	117.420	-3.580	106.999	113.217	-7.783
104.967	114.751	-1.449	103.706	109.665	-6.535	105.571	110.117	-6.083	101.425	113.255	-2.945	113.269	114.997	-1.203	105.832	112.542	-3.658
90.912	108.287	3.187	101.204	108.057	2.957	101.514	107.001	1.901	96.693	110.843	5.743	101.407	108.552	3.452	102.499	110.614	5.514
0.7825			0.8419			0.6821			0.7219			0.8283			0.7864		

4.50			4.78			5.05			5.88			4.25			5.52		
53.94			57.35			60.56			70.52			50.96			66.25		
**x4-52**			**A-31**			**n-192**			**x4-2**			**s11-43**			**q11-37**		
158.211	154.055	-14.745	151.915	157.334	-11.466	209.061	159.891	-8.909	168.926	158.969	-9.831	202.712	151.436	-17.364	207.233	152.826	-15.974
153.255	149.045	15.345	137.912	140.369	6.669	185.882	150.091	16.391	148.793	141.244	7.544	155.958	136.232	2.532	147.866	133.496	-0.204
138.915	134.548	1.248	132.576	133.905	0.605	139.672	130.553	-2.747	147.293	139.924	6.624	144.550	132.522	-0.778	145.078	132.588	-0.712
136.624	132.233	-0.167	132.322	133.597	1.197	135.558	128.814	-3.586	141.557	134.874	2.474	142.068	131.715	-0.685	144.530	132.410	0.010
133.671	129.247	0.647	131.404	132.485	3.885	127.264	125.307	-3.293	136.401	130.334	1.734	139.695	130.943	2.343	139.338	130.719	2.119
133.549	129.124	3.924	128.585	129.070	3.870	125.262	124.461	-0.739	129.353	124.129	-1.071	137.274	130.156	4.956	135.851	129.584	4.384
128.798	124.321	-0.079	126.159	126.131	1.731	122.409	123.255	-1.145	128.506	123.384	-1.016	135.189	129.477	5.077	131.581	128.194	3.794
127.785	123.297	0.797	123.539	122.956	0.456	121.864	123.024	0.524	127.693	122.668	0.168	129.548	127.643	5.143	130.841	127.953	5.453
122.971	118.431	-3.969	121.412	120.380	-2.020	113.434	119.460	-2.940	126.842	121.919	-0.481	121.602	125.059	2.659	127.957	127.014	4.614
122.111	117.561	-3.439	121.100	120.002	-0.998	113.102	119.320	-1.680	123.240	118.747	-2.253	117.950	123.871	2.871	120.624	124.626	3.626
118.036	113.442	-2.758	121.035	119.923	3.723	110.272	118.123	1.923	112.006	108.857	-7.343	110.749	121.530	5.330	111.228	121.567	5.367
112.891	108.241	3.141	102.474	97.436	-7.664	94.052	111.265	6.165	111.702	108.589	3.489	23.048	93.009	-12.091	22.475	92.669	-12.431
0.7968			0.8863			0.8289			0.8897			0.7711			0.7868		
4.19			3.69			4.17			3.67			5.15			4.89		
50.26			44.28			50.04			44.03			61.83			58.69		
**q-6**			**A-137**			**A-53**			**n-110**			**H-56**			**A-11**		
184.639	153.564	-15.236	208.904	161.142	-7.658	207.260	160.071	-8.729	193.677	155.018	-13.782	188.636	158.586	-10.214	191.309	155.997	-12.803
177.173	149.645	15.945	181.862	149.282	15.582	184.046	150.038	16.338	182.190	149.907	16.207	162.547	143.605	9.905	180.210	150.689	16.989
154.618	137.806	4.506	135.349	128.881	-4.419	135.796	129.184	-4.116	152.470	136.681	3.381	149.402	136.058	2.758	145.328	134.009	0.709
145.999	133.282	0.882	131.337	127.121	-5.279	130.973	127.100	-5.300	137.659	130.090	-2.310	147.623	135.036	2.636	135.115	129.125	-3.275
130.937	125.376	-3.224	130.433	126.725	-1.875	130.340	126.826	-1.774	135.871	129.295	0.695	144.802	133.416	4.816	129.893	126.628	-1.972
130.201	124.990	-0.210	128.953	126.076	0.876	128.969	126.233	1.033	124.296	124.144	-1.056	130.736	125.340	0.140	128.234	125.834	0.634
128.317	124.001	-0.399	121.499	122.806	-1.594	119.815	122.277	-2.123	123.884	123.960	-0.440	128.767	124.209	-0.191	126.604	125.055	0.655
120.580	119.939	-2.561	119.835	122.077	-0.423	118.301	121.623	-0.877	118.231	121.445	-1.055	120.457	119.437	-3.063	118.771	121.309	-1.191
118.795	119.002	-3.398	116.014	120.401	-1.999	114.921	120.162	-2.238	109.844	117.713	-4.687	120.423	119.418	-2.982	118.182	121.028	-1.372
116.247	117.665	-3.335	113.122	119.132	-1.868	113.186	119.412	-1.588	109.365	117.499	-3.501	118.840	118.509	-2.491	111.848	117.999	-3.001
110.265	114.525	-1.675	106.056	116.033	-0.167	106.423	116.489	0.289	109.188	117.421	1.221	105.651	110.936	-5.264	102.319	113.442	-2.758
108.853	113.784	8.684	101.136	113.875	8.775	101.198	114.231	9.131	93.367	110.380	5.280	102.347	109.039	3.939	100.343	112.497	7.397
0.7537			0.8266			0.8076			0.789			0.8787			0.7861		
5.00			4.21			4.46			4.47			4.03			4.40		
60.05			50.51			53.54			53.61			48.40			52.76		
**H11-108**			**q-60**			**s11-60**			**n-111**			**q11-39**			**n-124**		
189.822	151.178	-17.622	184.949	153.048	-15.752	183.414	149.779	-19.021	185.585	155.027	-13.773	187.805	153.547	-15.253	184.752	153.909	-14.891
175.492	145.134	11.434	181.476	151.300	17.600	174.475	145.928	12.228	157.232	140.278	6.578	174.549	147.193	13.493	169.242	146.044	12.344
158.000	137.755	4.455	150.647	135.781	2.481	158.568	139.075	5.775	151.854	137.480	4.180	162.364	141.353	8.053	148.158	135.352	2.052
149.272	134.074	1.674	134.697	127.751	-4.649	148.021	134.531	2.131	144.435	133.621	1.221	140.032	130.649	-1.751	141.372	131.911	-0.489
148.407	133.709	5.109	134.076	127.439	-1.161	142.557	132.178	3.578	140.130	131.382	2.782	134.640	128.065	-0.535	134.553	128.453	-0.147
143.970	131.838	6.638	133.187	126.991	1.791	132.986	128.054	2.854	136.192	129.333	4.133	129.030	125.376	0.176	133.846	128.094	2.894
121.682	122.436	-1.964	126.966	123.860	-0.540	125.368	124.773	0.373	134.723	128.569	4.169	125.716	123.788	-0.612	132.275	127.298	2.898
118.134	120.940	-1.560	124.658	122.698	0.198	117.838	121.529	-0.971	132.337	127.328	4.828	123.625	122.785	0.285	130.906	126.603	4.103

116.567	120.279	-2.121	117.315	119.001	-3.399	116.536	120.968	-1.432	121.250	121.560	-0.840	119.849	120.976	-1.424	122.965	122.577	0.177
104.776	115.306	-5.694	115.051	117.862	-3.138	112.933	119.416	-1.584	110.303	115.866	-5.134	114.232	118.283	-2.717	116.119	119.105	-1.895
103.500	114.767	-1.433	108.399	114.513	-1.687	88.438	108.863	-7.337	94.870	107.837	-8.363	99.306	111.129	-5.071	93.579	107.675	-8.525
83.319	106.255	1.155	106.165	113.388	8.288	87.696	108.543	3.443	90.061	105.336	0.236	98.011	110.509	5.409	91.420	106.580	1.480
0.7713			0.7309			0.7474			0.8368			0.7843			0.8068		
5.07			5.06			5.06			4.69			4.56			4.32		
60.86			60.68			60.73			56.24			54.78			51.90		
**x4-64**			**G1-40**			**x4-48**			**n-195**			**n-209**			**q-13**		
159.589	152.668	-16.132	178.123	162.748	-6.052	171.651	163.092	-5.708	185.628	151.238	-17.562	186.162	153.283	-15.517	167.408	152.683	-16.117
151.912	145.477	11.777	143.847	135.313	1.613	146.422	140.618	6.918	178.699	148.129	14.429	171.881	146.636	12.936	164.551	150.751	17.051
147.868	141.689	8.389	143.309	134.883	1.583	142.386	137.022	3.722	152.352	136.310	3.010	151.681	137.233	3.933	142.343	135.734	2.434
140.868	135.132	2.732	141.680	133.579	1.179	137.743	132.886	0.486	140.006	130.772	-1.628	138.788	131.231	-1.169	139.857	134.053	1.653
134.390	129.065	0.465	136.488	129.423	0.823	132.680	128.376	-0.224	137.688	129.732	1.132	135.001	129.468	0.868	130.269	127.570	-1.030
129.097	124.107	-1.093	136.411	129.361	4.161	129.386	125.442	0.242	135.963	128.958	3.758	134.106	129.051	3.851	126.126	124.768	-0.432
128.499	123.546	-0.854	135.918	128.967	4.567	128.205	124.390	-0.010	134.556	128.327	3.927	133.027	128.549	4.149	123.068	122.701	-1.699
128.474	123.523	1.023	133.253	126.834	4.334	127.297	123.581	1.081	121.930	122.663	0.163	119.982	122.477	-0.023	120.166	120.738	-1.762
123.923	119.260	-3.140	123.943	119.382	-3.018	125.908	122.344	-0.056	120.614	122.072	-0.328	115.795	120.528	-1.872	119.192	120.080	-2.320
123.721	119.071	-1.929	119.349	115.705	-5.295	115.524	113.094	-7.906	108.470	116.625	-4.375	108.783	117.263	-3.737	117.316	118.811	-2.189
115.898	111.743	-4.457	118.341	114.898	-1.302	114.998	112.625	-3.575	93.813	110.050	-6.150	93.039	109.935	-6.265	111.154	114.644	-1.556
112.241	108.318	3.218	102.836	102.488	-2.612	112.255	110.182	5.082	90.991	108.784	3.684	88.685	107.908	2.808	105.961	111.133	6.033
0.7938			0.9423			0.9225			0.752			0.7943			0.7577		
4.60			3.04			2.92			5.01			4.76			4.52		
55.21			36.54			35.01			60.15			57.13			54.28		
**C-69**			**x4-60**			**x4-22**			**x4-50**			**q11-5**			**s11-29**		
162.209	151.092	-17.708	152.048	154.619	-14.181	187.194	146.677	-22.123	160.698	151.410	-17.390	172.259	155.532	-13.268	173.804	157.901	-10.899
154.196	144.907	11.207	143.276	143.854	10.154	166.802	139.326	5.626	155.658	147.444	13.744	159.830	146.520	12.820	147.643	139.544	5.844
147.931	140.071	6.771	142.313	142.673	9.373	161.977	137.587	4.287	149.274	142.421	9.121	152.519	141.219	7.919	146.451	138.708	5.408
140.716	134.502	2.102	133.149	131.426	-0.974	150.710	133.525	1.125	138.715	134.112	1.712	140.913	132.803	0.403	141.204	135.026	2.626
139.154	133.296	4.696	131.606	129.533	0.933	150.087	133.300	4.700	127.986	125.669	-2.931	131.475	125.960	-2.640	138.829	133.359	4.759
132.669	128.290	3.090	130.053	127.627	2.427	147.918	132.518	7.318	126.648	124.616	-0.584	129.054	124.204	-0.996	131.233	128.029	2.829
123.710	121.375	-3.025	127.378	124.344	-0.056	127.077	125.005	0.605	125.668	123.845	-0.555	128.707	123.952	-0.448	126.257	124.538	0.138
123.105	120.908	-1.592	126.528	123.301	0.801	124.712	124.153	1.653	121.990	120.951	-1.549	124.170	120.663	-1.837	125.233	123.819	1.319
122.886	120.739	-1.661	122.232	118.029	-4.371	118.997	122.092	-0.308	119.884	119.294	-3.106	120.434	117.954	-4.446	121.139	120.946	-1.454
122.539	120.471	-0.529	120.666	116.107	-4.893	118.156	121.789	0.789	117.047	117.061	-3.939	117.452	115.791	-5.209	111.701	114.324	-6.676
112.725	112.895	-3.305	116.741	111.291	-4.909	117.544	121.569	5.369	114.909	115.379	-0.821	116.723	115.263	-0.937	106.505	110.678	-5.522
102.571	105.058	-0.042	116.369	110.834	5.734	46.707	96.032	-9.068	109.862	111.407	6.307	114.656	113.764	8.664	100.885	106.734	1.634
0.7847			0.8029			0.7121			0.7422			0.7883			0.8813		
4.64			4.90			5.25			5.15			4.97			4.09		
55.73			58.80			62.97			61.76			59.59			49.11		

**C-21**		
168.022	155.522	-13.278
150.937	142.554	8.854
144.967	138.023	4.723

137.165	132.101	-0.299
133.118	129.030	0.430
132.571	128.614	3.414
128.549	125.562	1.162
127.667	124.892	2.392
126.845	124.268	1.868
123.581	121.791	0.791
107.209	109.365	-6.835
97.408	101.926	-3.174
0.8594		
3.94		
47.22		

6. Experimental NMR data of aaptourinamine

Table S2. ^{1}H, ^{13}C NMR and key 2D NMR data of aaptourinamine [a]

No	C	H	HMQC	HMBC (H→C)	^{1}H-^{1}H COSY
2	124.4	8.71, d (6.85)	2, 11	3, 3a, 9a	3
3	116.2	7.29, t (6.82)	3	2, 9b	2, 3a
3a	122.5	8.03, d (6.85)	3a	2, 3, 9a, 10 (w)	3
4		12.33, s			5, 6
5	125.2	7.25, t (2.57)	5	6, 6a, 7	4, 6
6	105.1	6.95, dd (2.35, 2.05)	6	5, 6a, 7	4, 5
6a	122.4				
7	133.3				
8	168.8				
9	128.5				
9a	132.4				
9b	121.0				
11	133.7	8.71, s		10	

w: weak cross peak

[a] ^{1}H-NMR: δ_H (ppm, J in Hz), (500 MHz, DMSO-d_6), ^{13}C-NMR: δ_C (ppm), (126 MHz, DMSO-d_6)

7. Experimental spectra of aaptourinamine

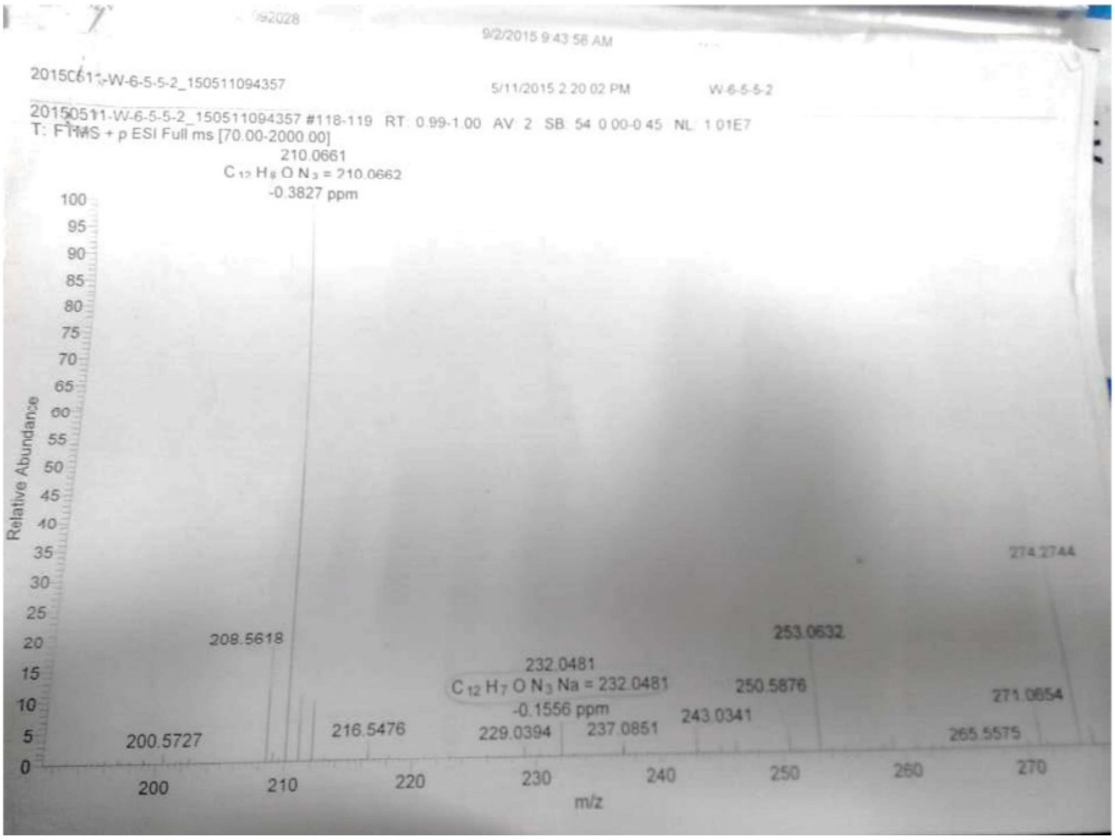

Figure S6. HRESIMS of aaptourinamine

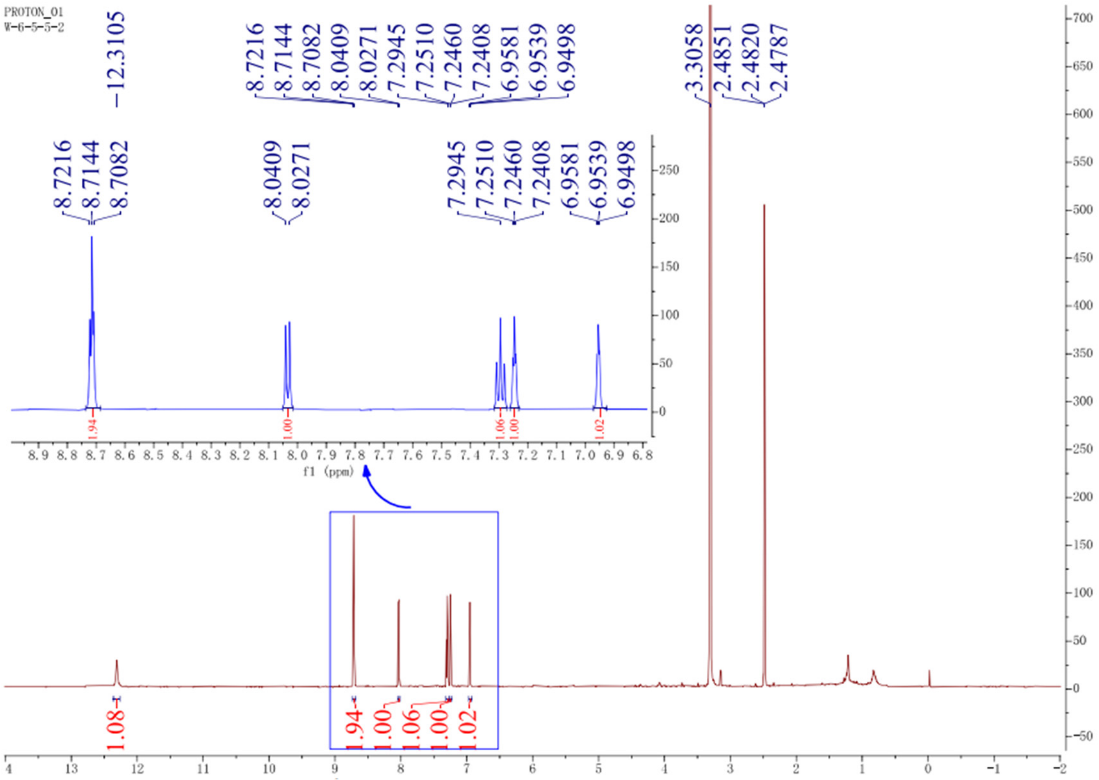

Figure S7. ^{1}H NMR of aaptourinamine (500 MHz, DMSO)

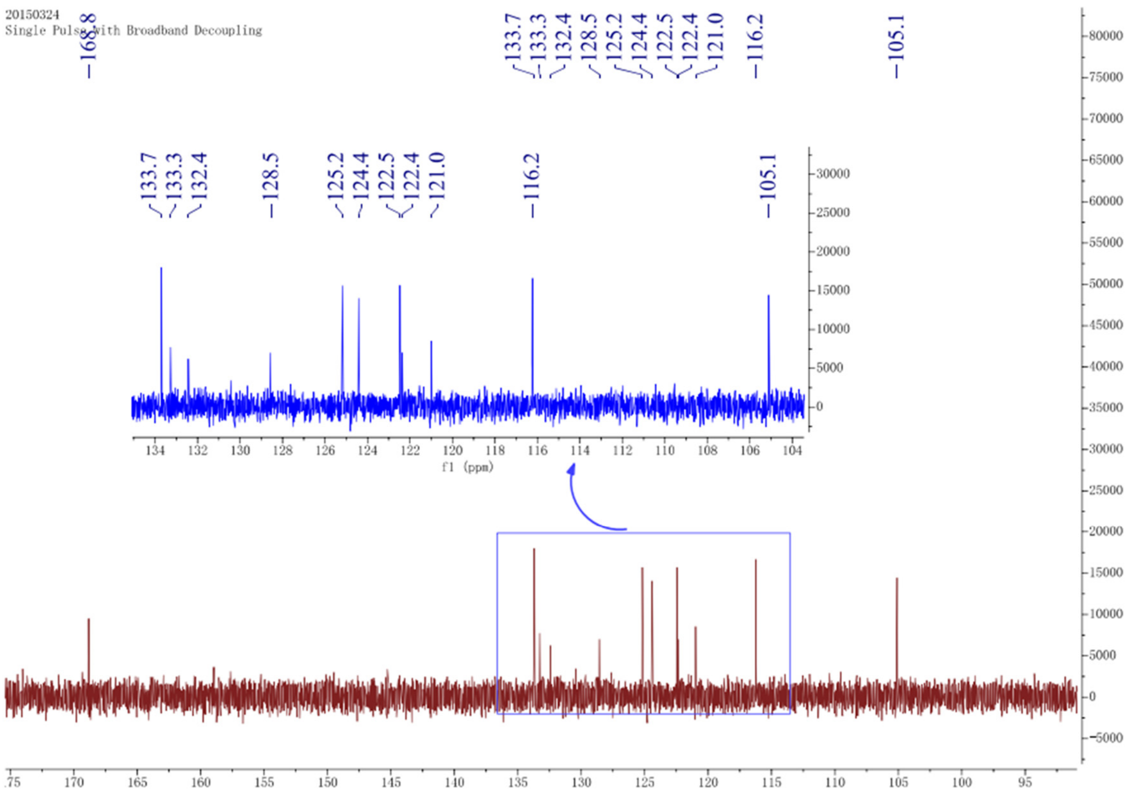

Figure S8. ^{13}C NMR of aaptourinamine (125 MHz, DMSO)

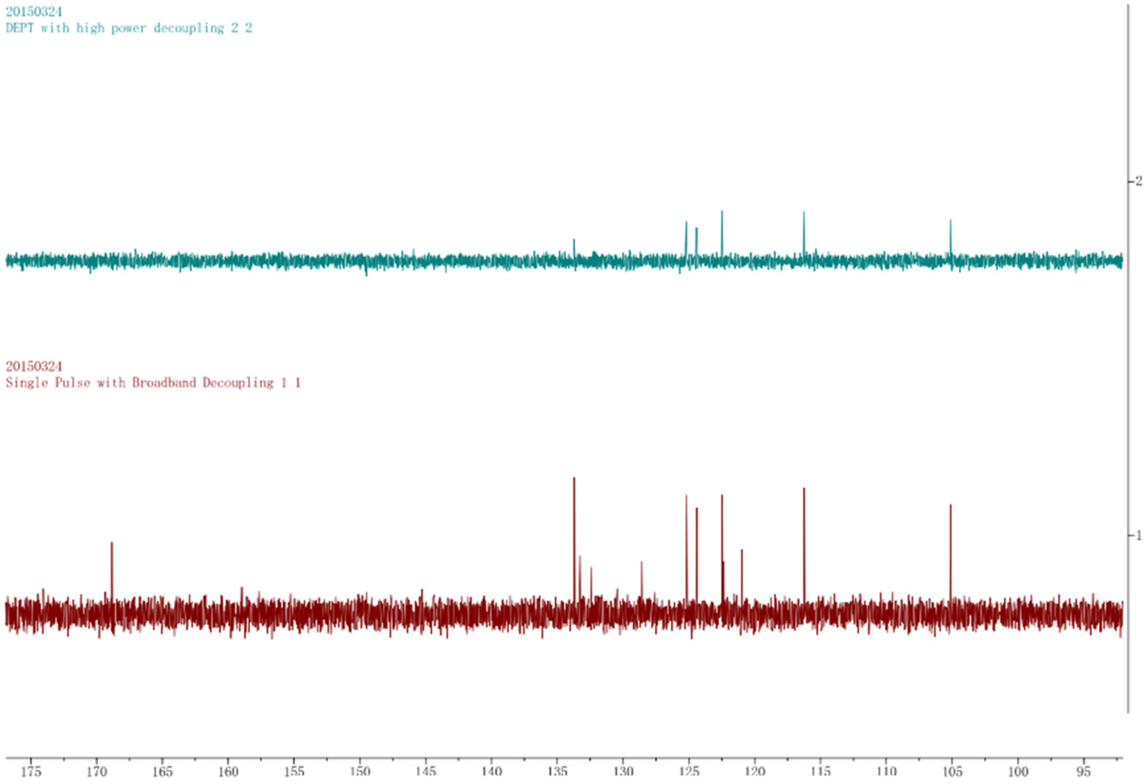

Figure S9. DEPT of aaptourinamine (DMSO)

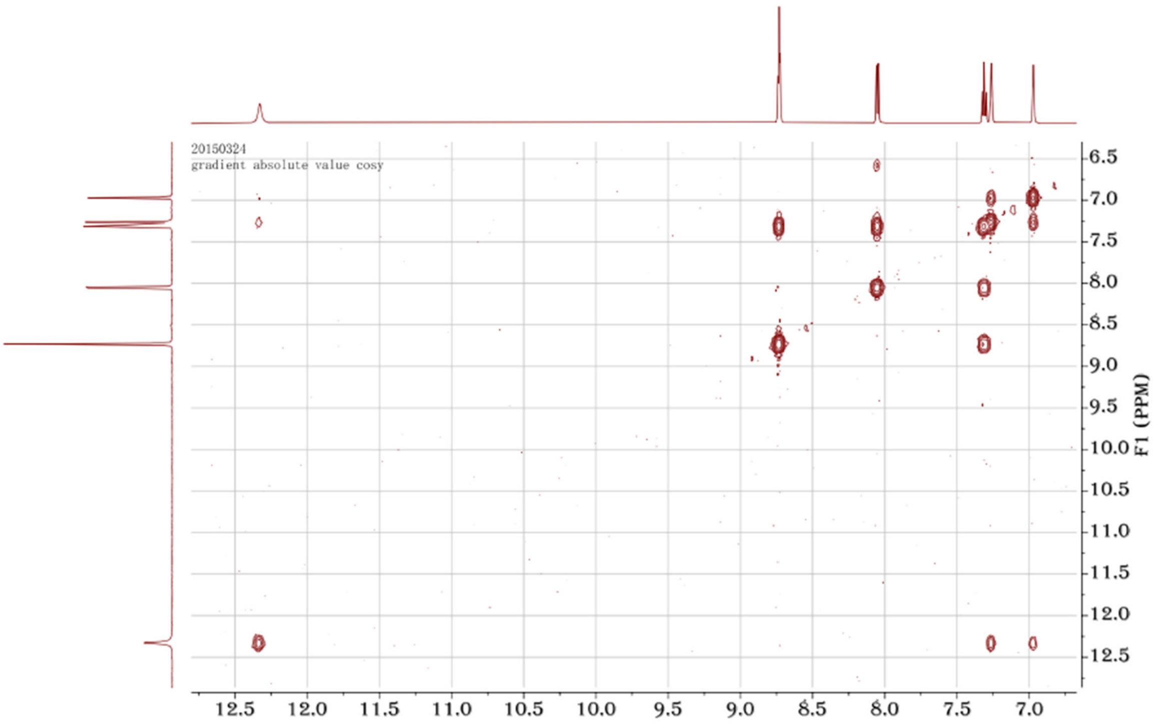

Figure S10. ^{1}H-^{1}H COSY of aaptourinamine (DMSO)

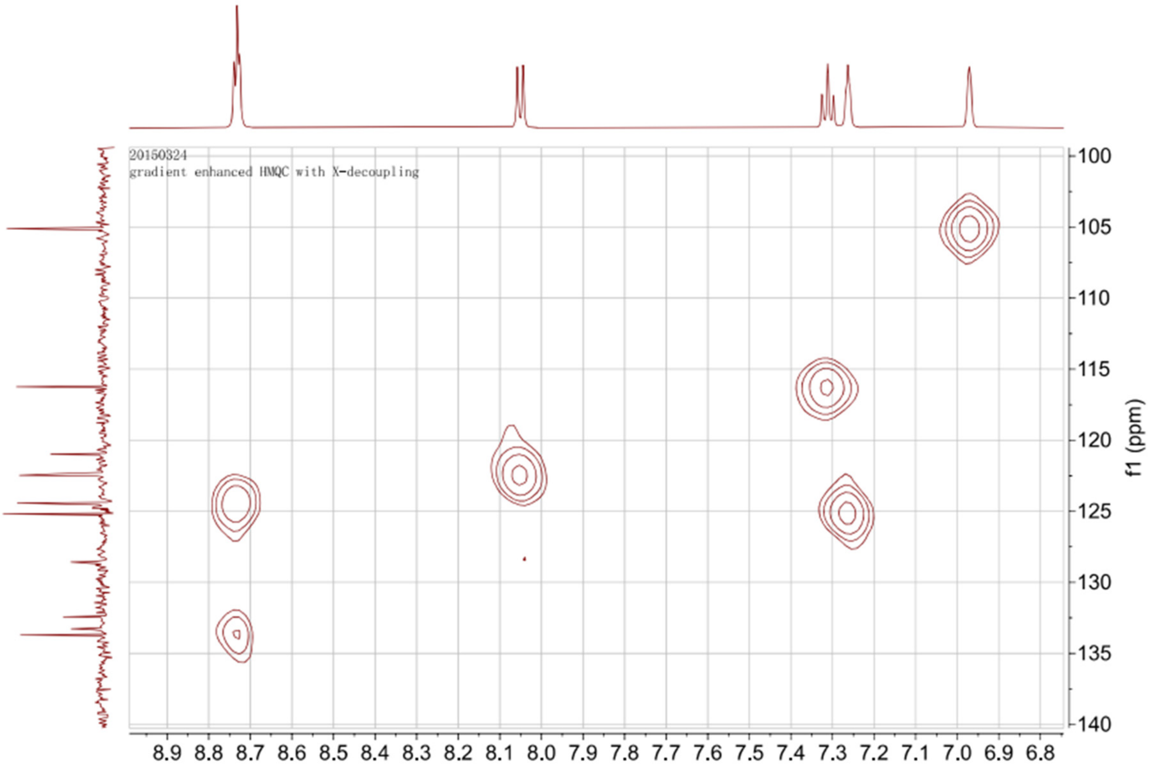

Figure S11. HMQC of aaptourinamine (DMSO)

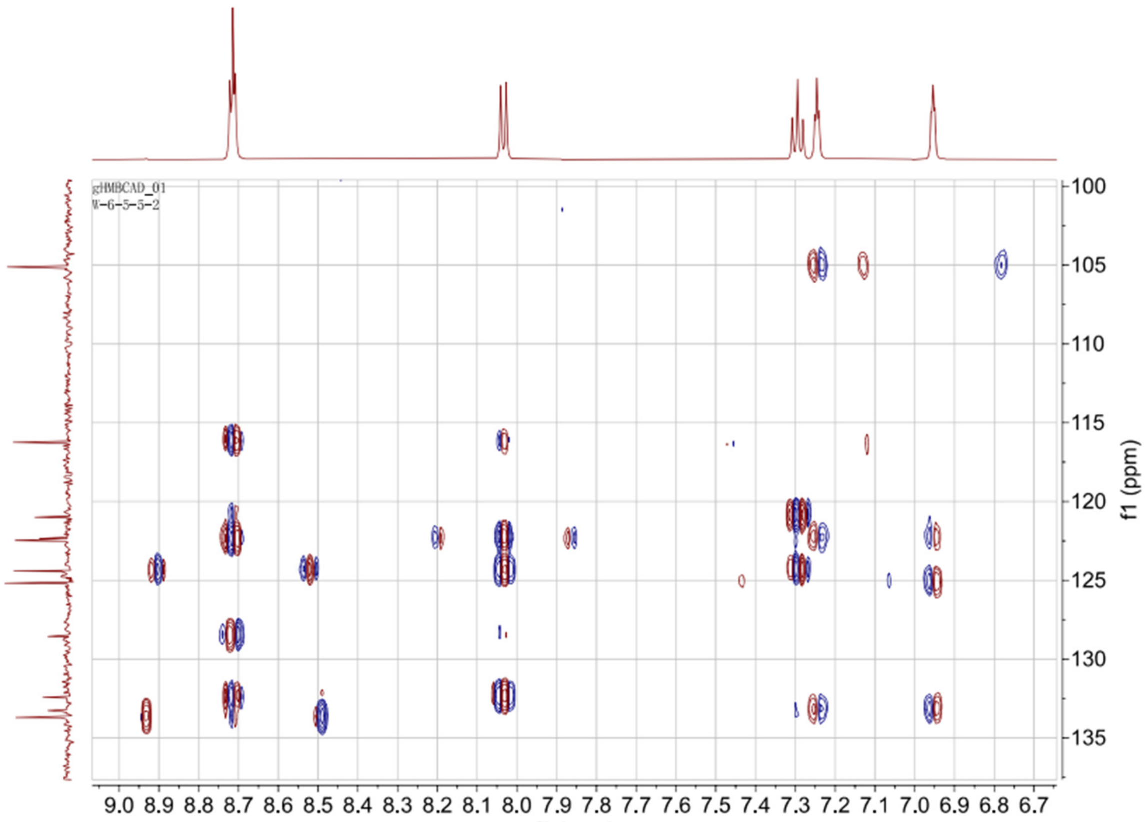

Figure S12. HMBC of aaptourinamine (DMSO)

Reference

32. Schrödinger. Available online: https://www.schrodinger.com/products/macromodel (accessed on 15 March 2021).

33. Frisch, M.; Trucks, G.; Schlegel, H.; Scuseria, G.; Robb, M.; Cheeseman, J.; Scalmani, G.; Barone, V.; Petersson, G.; Nakatsuji, H. Gaussian; Gaussian, Inc.: Wallingford, CT, USA, 2016.

34. Shermo: A General Code for Calculating Molecular Thermochemistry Properties. Available online: http://bbs.keinsci.com/thread-17494–1-1.html (accessed on 11 January 2021).

35. Jin, T.; Li, P.; Wang, C.; Tang, X.; Cheng, M.; Zong, Y.; Luo, L.; Ou, H.; Liu, K.; Li, G. Racemic Bisindole Alkaloids: Structure, Bioactivity, and Computational Study. Chinese Journal of Chemistry. *Chin. J. Chem.* **2021**, *39*, 2588-2598
